# Confinement Catalysis Enables Macrocyclization at Up to 0.6 M: Selective Formation of Mono- and Dimeric Glycosidic Macrocycles

**DOI:** 10.1021/jacs.6c03730

**Published:** 2026-03-18

**Authors:** Sudip Guria, Julia Bechter, Alessandro Prescimone, Konrad Tiefenbacher

**Affiliations:** † Department of Chemistry, 27209 University of Basel, Mattenstrasse 22, 4058 Basel, Switzerland; ‡ Department of Biosystems Science and Engineering, ETH Zurich, Schanzenstrasse 44, 4056 Basel, Switzerland

## Abstract

Macrocyclic compounds are widespread in nature and frequently found among bioactive natural products. Their conformational preorganization allows them to effectively engage large binding surfaces, making them valuable in drug discovery, especially for modulating protein–protein interactions. Consequently, macrocyclization strategies have received significant attention; however, they still typically rely on high-dilution conditions (0.10–10 mM) to favor intramolecular ring closure over intermolecular oligomerization. Here, we report a solution to this long-standing challenge using a catalytic confined-space approach that operates efficiently even at the substrate solubility limit (600 mM), thereby eliminating the need for high-dilution conditions. The capsular catalyst enables the high-yielding formation of medium- and large-sized glycosidic macrocycles with excellent β-selectivity. Moreover, the method addresses a second persistent challenge in macrocyclization: the selective formation of macrocyclic dimers. Whereas such dimers are inaccessible under traditional high-dilution conditions, they are obtained in high yields when two substrates fit into the capsule’s cavity, again at high substrate concentrations and with excellent β,β-selectivity. Control experiments establish the indispensability of the capsule as conventional conditions afford substantially lower yields and predominantly α-selectivity. The method’s utility is further demonstrated in the selective synthesis of the dimeric core structure of glucolipsin A and cycloviracin B1. This work establishes confinement catalysis as a powerful tool to overcome key limitations in macrocyclization chemistry.

## Graphical abstract

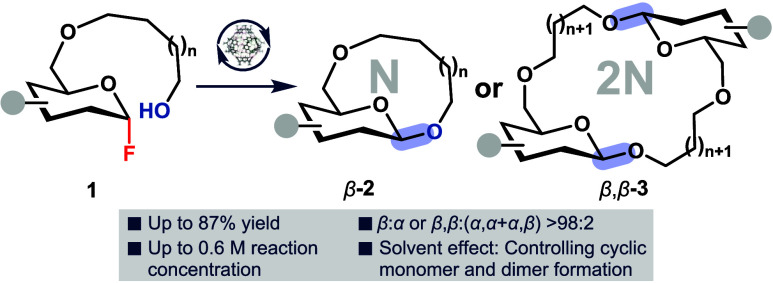

## Introduction

Macrocyclic compounds are highly prevalent in nature and frequently found among bioactive natural products.^1–4^ They also play a crucial role in drug discovery, particularly in addressing challenging targets that are often inaccessible to conventional small molecules. Their inherent conformational preorganization allows them to engage large binding surfaces with a minimal entropic penalty, making them ideal candidates for modulating protein–protein interactions and other complex biological interfaces.^1–4^ Despite their importance, the formation of medium- (8–11-membered) and large rings (≥12-membered) from flexible acyclic substrates remains a significant challenge. Medium-sized ring formation is disfavored due to transannular strain, which imposes an enthalpic penalty.^5,6^ In contrast, macrocyclization is primarily limited by entropic factors, as it requires a substantial loss of conformational freedom in the acyclic precursor.^5,6^ Significant efforts have been devoted in recent decades to developing efficient and versatile macrocyclization strategies.^7–10^ Despite significant advances, macrocyclization generally still requires high-dilution conditions (0.10–10 mM) to favor intramolecular ring closure over undesired intermolecular oligomerization (challenge #1, Figure 1A). An even greater synthetic hurdle is the selective formation of macrocyclic dimers (challenge #2, Figure 1A). Because these species arise from a linear dimer intermediate, the use of high dilution becomes counterproductive, as it suppresses formation of this prerequisite species. While templating has been successfully used to obtain macrocyclic dimers,^7^ no general, template-independent strategy currently exists for their reliable and selective synthesis.

**Figure 1:**
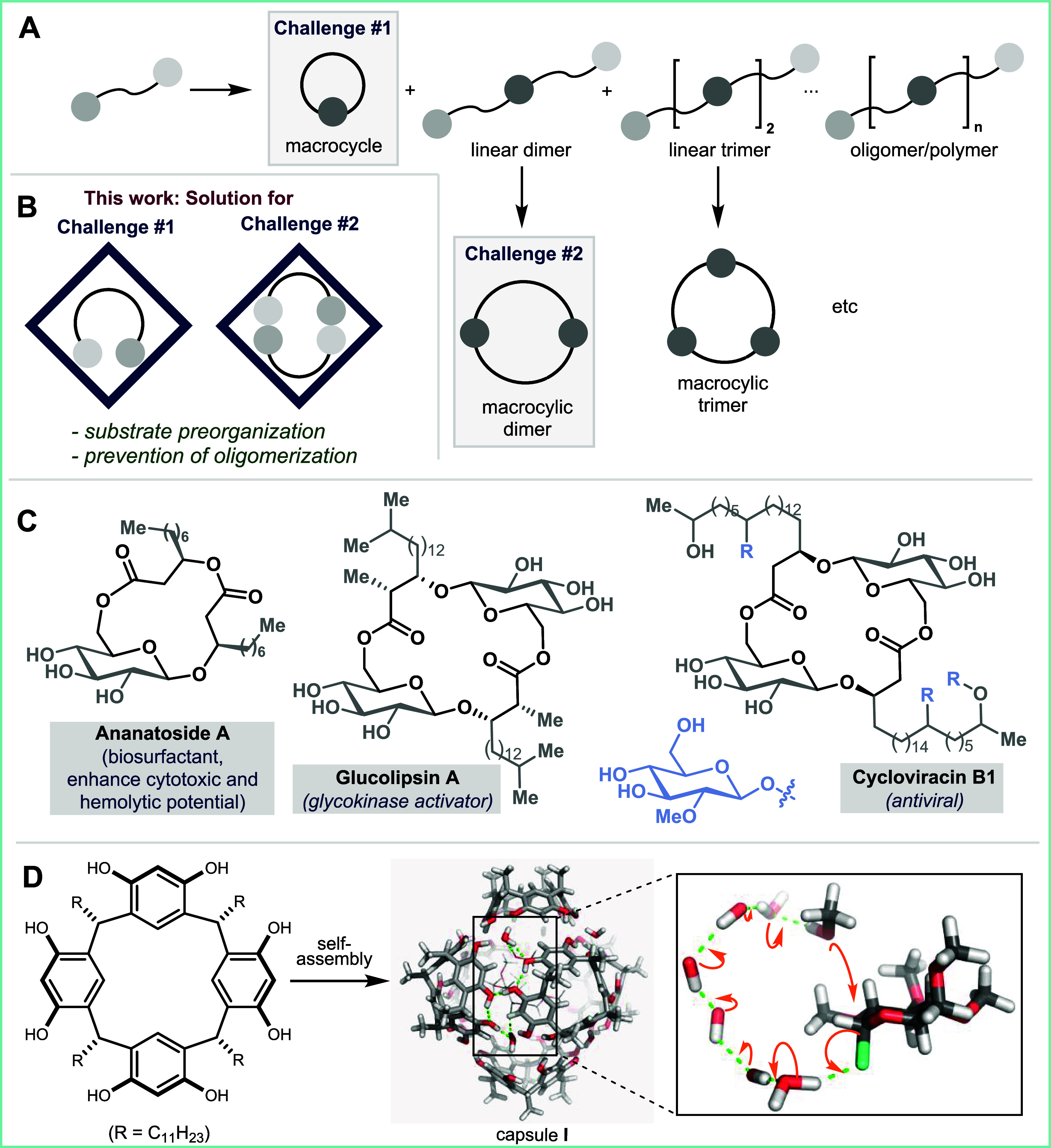
(A) General challenge of macrocyclization vs intermolecular oligomerization. (B) This work: A confinement-based solution for both challenges in the context of macroglycosylation. (C) Importance of macro-glycosides: natural products and biologically active molecules. (D) Self-assembly of resorcin[4]­arene (RS) to form the hexameric capsule **I**. A proton wire on the capsule surface simultaneously activates an alcohol (nucleophile) and a glycosyl halide (electrophile).

Supramolecular container-mediated catalysis has attracted significant interest due to its ability to replicate key features of enzymatic function, particularly substrate encapsulation, and selective activation within a defined microenvironment.^11–29^ Confinement of the reaction environment has been shown to promote selectivities that are otherwise challenging to achieve under standard solution-phase conditions. A seemingly obvious application of confinement catalysis is macrocyclization, where the encapsulation of a substrate within a closed nanoenvironment can significantly facilitate macrocyclization through multiple mechanisms. (1) Entropic preorganization: the confined space can fold the flexible substrate into a conformation that more closely resembles the transition state, effectively bringing the two reactive ends into proximity. (2) Suppression of side reactions: confinement can help prevent undesired intermolecular oligomerization, which typically competes with macrocyclization. While traditional macrocyclizations rely on high-dilution conditions, nanoconfinement may enable efficient ring closure at much higher, synthetically practical concentrations. (3) Furthermore, if two substrate molecules fit within the cavity, this may even promote the selective formation of macrocyclic dimers (challenge #2, Figure 1A).

Despite the promise of confinement-based strategies in addressing the challenges of macrocyclization, reported examples remain surprisingly limited.^6^ Heterogeneous porous materials have been explored to facilitate such transformations. For instance, ruthenium-based metathesis catalysts immobilized on mesocellular siliceous foam outperformed their solution-phase counterparts, particularly for ring sizes larger than 20 atoms.^30^ Nevertheless, reactions still required dilute conditions (5 mM), as increasing the concentration to 20 mM reduced macrocyclization yields due to competing cross-metathesis. Related approaches involving immobilization of olefin metathesis catalysts in mesoporous silica,^31–33^ or covalent organic frameworks,^34^ have also been reported. In exceptional cases, macrocyclization selectivity exceeded 50% even at 0.1 M concentration.^33^ Another heterogeneous example is the efficient Suzuki–Miyaura macrocyclization inside single-layered porous nanosheets, though again only at very low concentrations (10 μM).^35^ In contrast, homogeneous supramolecular containers have so far been applied exclusively in (super)­stoichiometric transformations. The Rebek group has reported several examples employing stoichiometric amounts of cavitands, leveraging the hydrophobic effect to achieve efficient substrate binding and orientation.^36–40^ In these examples, the molecular container functioned as a chaperone, folding the substrate into a suitable conformation, without acting as a catalyst or promoter itself. Furthermore, the Gibb group reported the use of stoichiometric amounts of a dimeric capsule to facilitate thioether and amine cyclizations.^41,42^ In all reported examples involving homogeneous supramolecular containers, very low concentrations (1–2 mM) were utilized, highlighting the persistent challenge of performing macrocyclizations at higher concentrations.

We aimed to explore the potential of catalytic macrocyclization within a confined space in the context of glycosylation chemistry. Macrocyclic glucosides are of interest due to their occurrence in bioactive natural products. Representative examples include ananatoside A,^43,44^ glucolipsin A,^45^ and cycloviracin B1 ^46,47^ (Figure 1C) that exclusively contain β-glycosidic bonds. While macroglycosylation reactions have been explored, they are typically performed at rather dilute concentrations (approximately 10–20 mM) and predominantly furnish the α-glycosidic bond when the substrate for cyclization is flexible.^48–50^ In earlier work, our group demonstrated that intermolecular glycosylation is catalyzed by the readily accessible resorcin[4]­arene capsule **I** ^51–54^ through an enzyme-like proton-wire mechanism (Figure 1D), which enables dual activation of both the glycosyl halide electrophile and the nucleophile.^55–57^ This unique mode of activation enables intermolecular glycosylation with high β-selectivity.

Here, we demonstrate that the resorcin[4]­arene capsule **I** can catalyze the even more challenging formation of medium- and large-sized glycosidic macrocycles as well as macrocyclic dimers in good yields (up to 87%) and excellent β- and β,β-selectivity. To our knowledge, such a catalyst-controlled β-selective glycosylation to form macroglycosides has not been reported before. Existing strategies for the synthesis of such products typically rely on ring-closing metathesis or macrolactonization under high-dilution conditions.^58–60^ In contrast, the methodology presented herein affords macrocyclic products in good yields even at substrate concentrations as high as 0.60 M.

## Results and Discussion

We selected the hydroxyalkyl-substituted, glucose-derived cyclization precursor **1c** (Table 1) as a test substrate. It features a fluoride leaving group that is sufficiently stable to be carried through a multistep sequence, including column chromatography, while remaining activatable within capsule **I**.^55^ Precursor **1c** can be synthesized in seven linear steps (see SI, section 10 for details) and serves as a precursor to a 12-membered macroglycoside, distantly related to the natural product ananatoside A (Figure 1C). Substrate **1c** was treated with 10 mol % of capsule **I** at 30 °C under standard (0.10 M in CDCl_3_), nonhigh dilution conditions in the presence of neutral alumina to scavenge hydrofluoric acid released during the course of the reaction. The reaction afforded macrocycle **2c** and cyclic dimer **3c** (**2c**:**3c** = 42:58) with high overall yield and excellent β- and β,β-selectivity (>98:2, Table 1, entry 1). When neutral alumina was replaced with basic alumina, the yield and the **2c**:**3c** ratio improved slightly (entry 2). However, without alumina as an HF scavenger, the yield and especially the β-selectivity decreased dramatically due acid-catalyzed epimerization and decomposition (entry 3). Subsequently, various solvents were screened to evaluate their effect on improving the selectivity and yield for either the macrocyclic monomer or dimer (for more details, see SI, section 3.1). Interestingly, the bulky solvent 1,2-dichlorobenzene (1,2-DCB) significantly increased the yield of the macrocyclic monomer **2c** relative to the dimer, affording an isolated yield of 70% while maintaining excellent β-selectivity (entry 4). In contrast, the smaller solvent dichloromethane (DCM) favored the formation of the macrocyclic dimer **3c**, providing a good isolated yield of 52% with excellent β,β-selectivity (entry 5). It should be noted that the macrocyclic monomer and dimer can be readily separated by silica gel chromatography due to their polarity difference.

**Table 1:**
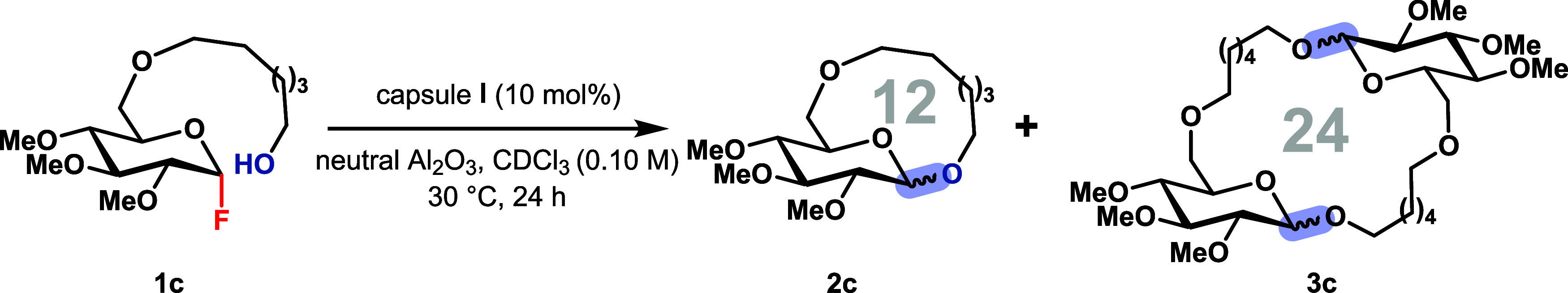
Summary of Reaction Condition Screening and Control Experiments^f^

entry	deviation	conversion (%)	Comb. yield^a^ (%)	**2c**/**3c**	β/α (**2c**)	β,β/(α,β + α,α) (**3c**)
1	none	>99	89	42:58	>98:2	>98:2
2	basic Al_2_O_3_ instead of neutral	>99	91	44:56	>98:2	>98:2
3	no Al_2_O_3_	>99	56	27:73	65:35	70:30
4	1,2-DCB instead of CDCl_3_	>99	79 (70)^b^	**89**:11	>98:2	>98:2
5	DCM instead of CDCl_3_	>99	69 (52)^c^	20:**80**	>98:2	>98:2
6	1.0 equiv. *n*Bu_4_NBr added	19	-	-	-	-
7	no capsule **I**	25	-	-	-	-
8^d^	2.0 equiv. TMSOTf as a promotor	>99	27	19:81	37:63	18:82
9^e^	2.0 equiv. BF_3_·Et_2_O as a promotor	>99	41	14:86	13:87	13:87

a NMR yield determined with internal standard (dimethyl terephthalate).

b Isolated yield of **2c**.

c Isolated yield of **3c**.

d Separate experiment without capsule **I**; 30 °C in DCM for 3 h.

e Separate experiment without capsule **I**; 30 °C in CDCl_3_ for 3 h.

f Standard reaction condition: Compound **1c** (50.0 μmol), capsule **I** (10 mol %), and 50 mg basic Al_2_O_3_ in 0.5 mL chloroform-*d* (CDCl_3_) at 30 °C for 24 h.

To investigate the importance of capsule **I** in determining reaction selectivity, a series of control experiments was conducted. When stoichiometric amounts of tetrabutylammonium bromide, a known high-affinity guest for the capsule,^61^ were added to block the capsule cavity, neither **2c** nor **3c** was detectable by ^1^H NMR (entry 6). Similarly, no product was detected in the absence of capsule **I** under otherwise identical reaction conditions. Furthermore, the substrate bearing the β-configured fluoride leaving group exhibited no reactivity under these conditions (see SI, section 4.1). Finally, for comparison, when the reaction was carried out under traditional, noncapsular, glycosylation conditions, using 2.0 equiv of either TMSOTf or BF_3_·Et_2_O as promoters, the macrocyclic monomer and dimer were formed only in low yields and predominantly with α-selectivity (entries 8 and 9). According to LC-MS analysis, the poor yield was attributed to intermolecular oligomerization and substrate hydrolysis.

### Substrates Scope

Next, the substrate scope of the reaction was explored. A series of ten substrates (**1a**–**1j**, Figure 2A), varying in side-chain length and sugar moieties, was synthesized with the potential to form, upon glycosylation, 10- to 28-membered macrocyclic monomers or 20- to 56-membered macrocyclic dimers. All substrates were converted under the optimized conditions, either using chloroform, DCM, or 1,2-DCB as solvent, affording the desired products in good yields and with excellent β- and β,β-selectivity.

**Figure 2:**
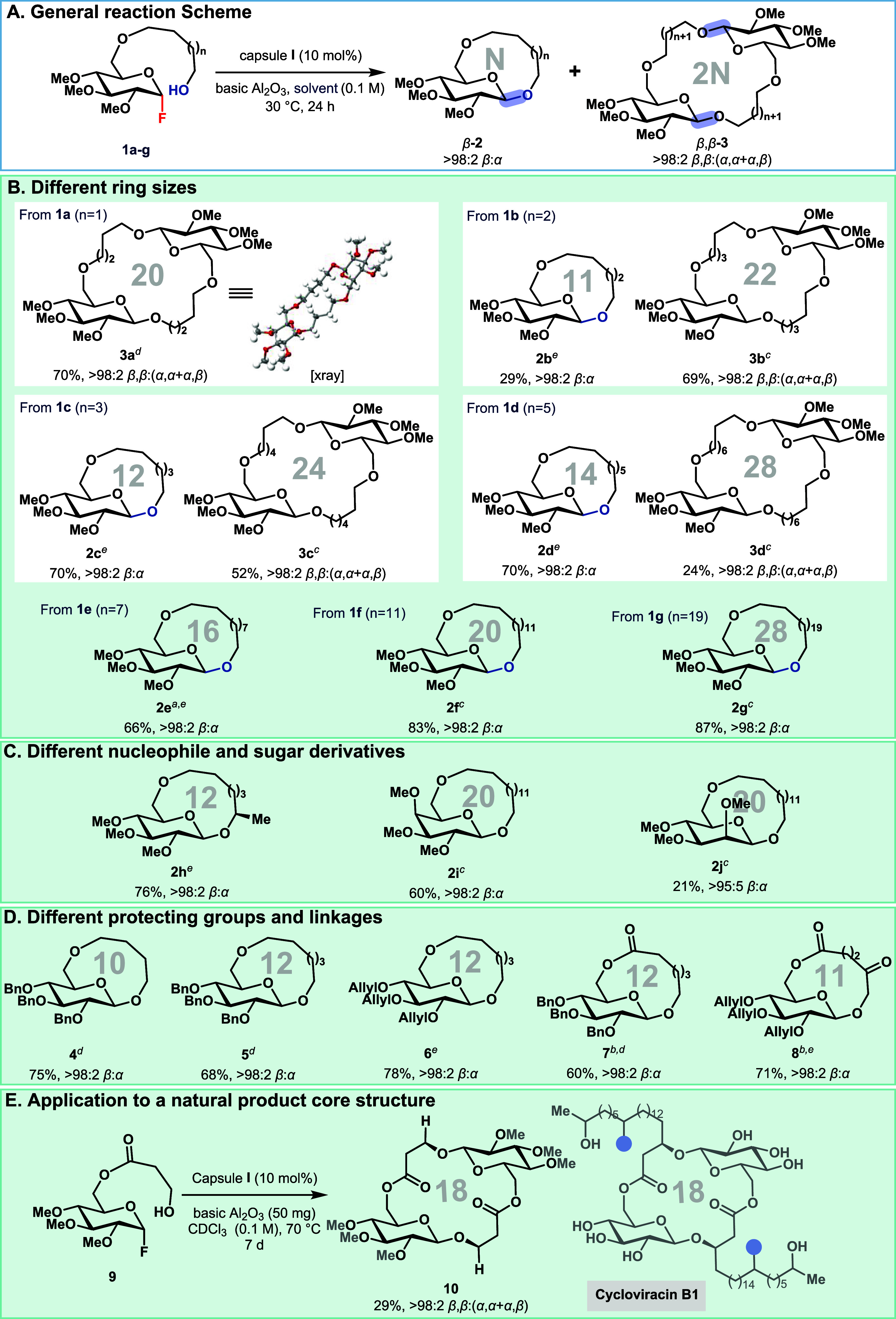
Substrate scope of β or β,β-selective macroglycosylation. Yields refer to isolated material. (A) General reaction scheme for synthesizing macrocyclic glycosides. (B) Scope for different ring sizes using methyl-protected sugar derivatives. (C) Secondary alcohol nucleophile and two different sugar electrophiles. (D) Exploration of different protecting groups and ester-linked sugar derivatives. (E) Synthesis of the core of the natural product cycloviracin B1 and glucolipsin A (see Figure 1C). Reaction condition: Compound **1** (100 μmol), capsule **I** (10 mol %), and 100 mg basic Al_2_O_3_ in 1.0 mL solvent at 30 °C for 24 h. ^*a*^The reaction was completed in 48 h. ^*b*^At 50 °C for 7 d. ^*c*^DCM as solvent. ^*d*^CDCl_3_ as solvent. ^*e*^1,2-DCB as solvent.

The selectivity between monomer and dimer formation was influenced by the choice of solvent, in line with the trends observed in the initial screening (Table 1, entries 4 and 5). Substrates **1a** and **1b** primarily afforded cyclic dimers, with isolated yields of 69% and 70%, respectively. However, when the bulky solvent 1,2-DCB was used, the macrocyclic monomer **2b** was obtained in 29% isolated yield (Figure 2B). For the larger substrates **1c** and **1d**, macrocyclic dimer formation decreased, accompanied by a corresponding increase in the yield of the macrocyclic monomers. For the largest substrates studied (**1e**–**1g**), the macrocyclic monomers were formed almost exclusively with excellent β-selectivity. To demonstrate the generality of our method, a chiral secondary alcohol as well as galactose- and mannose-derived substrates were evaluated under the reaction conditions. In 1,2-DCB, the chiral secondary alcohol afforded the highly β-selective macroglycoside **2h** in 76% isolated yield (Figure 2C). Additionally, galactose and mannose derivatives cyclized in dichloromethane to give the cyclic monomers **2i** and **2j**, respectively in excellent β-selectivity.

To further expand the utility of the developed methodology, we investigated cleavable protecting groups on the glucose core. Accordingly, benzyl- and allyl-protected derivatives were synthesized and evaluated under the glycosylation conditions (Figure 2D). These substrates are larger than the previously studied methylated analogs, a feature that generally favors the formation of macrocyclic monomers inside the capsule. Notably, even the 10- and 12-membered glycosides **4**–**6** were obtained in high yields (68–78%).

Additionally, we explored ester linkages at the primary alcohol of the glucose substrate, motivated by the presence of similar functionalities in related natural products (Figure 1C). Although these substrates were less reactive, good yields (60–71%) and excellent β-selectivity were achieved for products **7** and **8** at slightly elevated temperatures (50 °C) and extended reaction times, demonstrating the application potential of the methodology developed.

Encouraged by these results, we aimed to apply this novel methodology to the synthesis of a natural product derivative. We chose the dimeric core motif of *glucolipsin A* and *cycloviracin B1* (Figure 1C) as a target. Substrate **9** was synthesized in seven linear steps (see SI, section 10.9), and upon exposure to the reaction conditions, was converted with excellent β,β-selectivity into the desired core structure **10** of the natural products (Figure 2E).

We next sought to rationalize the observed solvent effects. The results of all solvent screenings corresponding to the reactions in Figure 2B are summarized in Figure 3A. Two trends emerge:

(a) Substrate size is the primary determinant of the cyclization outcome. Larger substrates exhibit a stronger tendency to form monomeric macroglycosides. For example, substrates **1f** and **1g**, which form monomeric rings of ≥20 atoms, did not yield any detectable macrocyclic dimers by ^1^H NMR analysis. This trend is consistent with the occupancy model proposed by Rebek and Mecozzi, which suggests an optimal cavity occupancy of approximately 55% for stable host–guest complexes, close to the typical packing efficiency observed in many organic liquids.^62^ In our previous work, we identified an occupancy threshold of approximately 58% as the upper limit for the largest products formed via intermolecular glycosylation (product size ∼800 Å^3^; capsule volume^51^ ∼1375 Å^3^).^57^ Binding of two substrate molecules **1f**, capable of forming a 40-membered dimeric macrocycle, would significantly exceed the optimal occupancy, reaching approximately 70% (for details see SI, section 11). Conversely, for the smaller substrates **1a**–**e**, macrocyclic dimers were formed to varying extents. For the smallest substrates, **1a** and **1b**, the dimeric product was predominant. This trend can again be rationalized by occupancy considerations. Binding of a single molecule of substrate **1a** results in an occupancy of only 22%, excluding the contribution of solvent molecules that help fill the remaining space. In contrast, binding of two substrate molecules yields an occupancy of approximately 44%, which is much closer to the ideal value.
(b) Interestingly, the solvent also exerts a significant influence on monomer/dimer selectivity, with the most striking effect observed for substrate **1c**. When using the bulky solvent 1,2-DCB, the macrocyclic monomer was obtained in 70% isolated yield. In contrast, the smaller solvent dichloromethane (DCM) predominantly afforded the macrocyclic dimer in 52% isolated yield. Binding of a single molecule of **1c** results in an occupancy of only 25%, excluding solvent contributions. With 1,2-DCB, just three solvent molecules are sufficient to increase the occupancy to a reasonable 52%. In contrast, achieving a similar occupancy with DCM would require six to seven molecules. However, when two molecules of **1c** bind simultaneously, the occupancy increases to 49%, already close to the optimal value. In this case, the addition of a single DCM molecule brings the occupancy to 54%, while one molecule of 1,2-DCB would overshoot the ideal threshold (58%). This observation provides a rationale for the solvent-dependent selectivity, with DCM favoring dimerization and 1,2-DCB promoting monomeric macrocycle formation. Additional solvent effects were observed at the extremes of substrate size. For the smallest substrate, **1a**, only chloroform afforded the product in good yield; neither DCM nor 1,2-DCB was effective. This unexpected result may be attributed to more efficient capsule packing in the case of chloroform.

**Figure 3:**
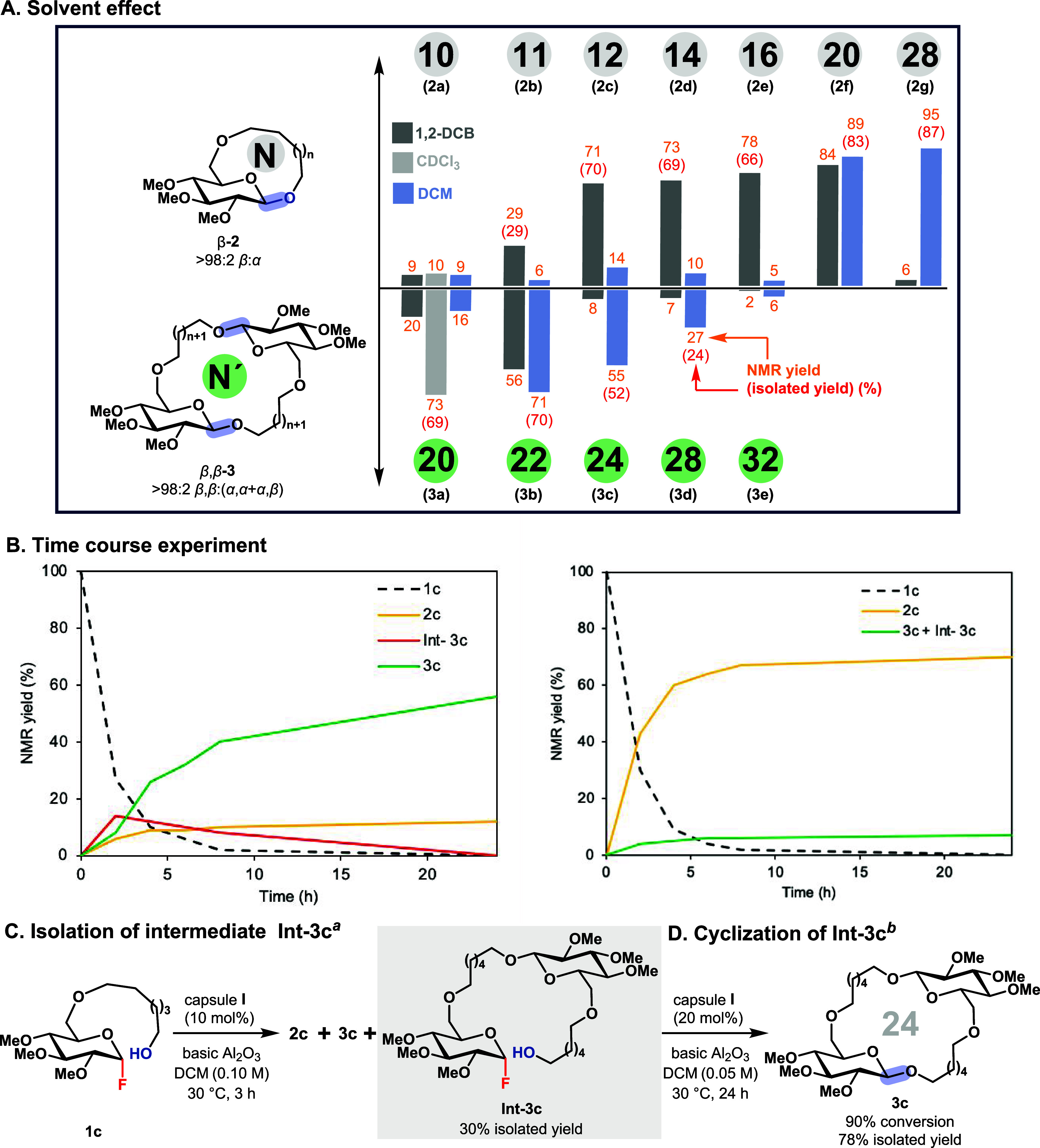
(A) Overview of the solvent effects observed for the cyclization of substrates depicted in Figure 2B. The respective ring sizes are indicated by large black numbers inside the colored circles for ease of identification. (B) Mechanistic insight into cyclic dimer formation. Time-course studies for substrate **1c** (left = reaction in dichloromethane, right = reaction in 1,2-DCB). (C) Isolation of β-linked linear dimer (**Int-3c**). (D) Conversion of the β-linked linear dimer (**Int-3c**) to the β,β- dimer **3c**. Reaction condition: ^*a*^Compound **1c** (500 μmol), capsule **I** (10 mol %), and 500 mg basic Al_2_O_3_ in 5.0 mL DCM at 30 °C for 3 h. ^*b*^Compound **Int-3c** (12.5 μmol), capsule **I** (20 mol %), and 25 mg basic Al_2_O_3_ in 0.25 mL DCM at 30 °C for 24 h.

Overall, the data suggest that efficient formation of macrocyclic dimers requires not only favorable packing of two substrates within the capsule but also a slower rate of monomer cyclization. Evidence for the coencapsulation of two substrate molecules was obtained for substrate **9** (see SI, section 5 for details).

At the opposite size extreme, the formation of the 28-membered macrocyclic monomer was successful only in DCM. Although 1,2-DCB can offer a similar occupancy, yields were low, suggesting that occupancy alone does not fully account for reactivity; factors such as substrate mobility may also play a role.

### Cyclization at the Solubility Limit

Finally, we sought to explore the concentration limits of this methodology. The standard substrate concentration of 0.10 M was already high compared to typical macrocyclization conditions, which are often conducted at concentrations ranging from 0.10 to 10 mM.^7–10^ Nevertheless, we pushed the system to the solubility limit in 1,2-DCB (0.60 M) for substrate **1c**. Macrocyclizations at such high concentrations are rare and typically require a template and/or high preorganization. Remarkably, even under these extreme conditions, the macrocyclic products were obtained in a high combined yield (73%, versus 79% at 0.10 M, Table 2). Importantly, excellent β-selectivity was maintained. This result corresponds to an *Emac* score of 8.4 (overall for monomer and dimer) or 8.0 for the monomer alone, which, according to the established scale (*Emac* 8–9), already indicates a successfully optimized process.^63^ Notably, no specific optimization was carried out for this specific example, highlighting the intrinsic potential of macrocyclizations catalyzed within a supramolecular container. Control experiments were conducted at this concentration. When the capsule was blocked with the high-affinity guest tetrabutylammonium bromide,,^61^ no product formation was observed (entry 3). Using conventional reaction promoters, only trace amounts of the desired products were detected (entries 4 and 5). These results again highlight the unique potential of macrocyclizations catalyzed within a supramolecular container.

**Table 2:**
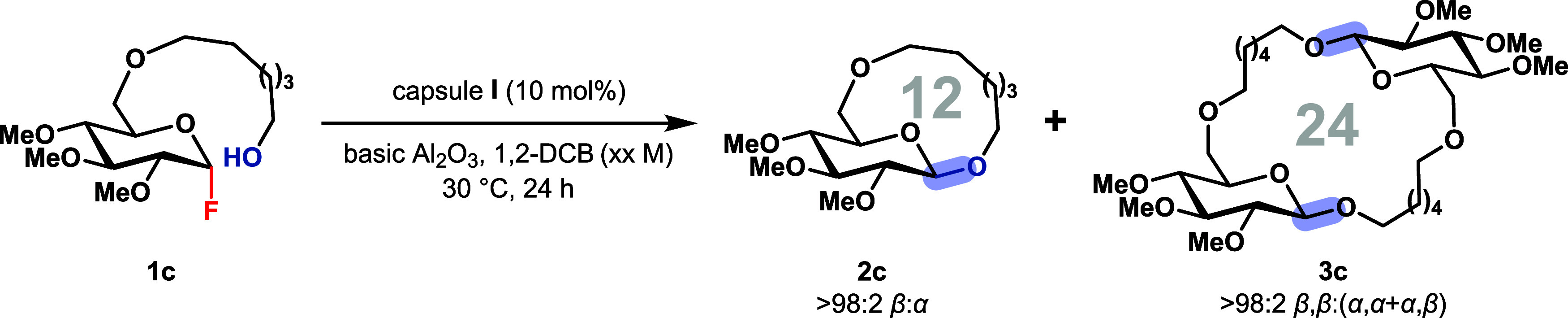
Exploring Macrocyclization at the Solubility Limit (0.60 M)

entry	concentration (M)	conversion (%)	Comb. NMR yield (%)	*Emac*	**2c**/**3c**	β/α (**2c**)	β,β/(α,β + α,α) (**3c**)
1^a^	0.10	>99	79	7.7	89:11	>98:2	>98:2
2^b^	0.60	>99	73	8.4	77:23	>98:2	>98:2
3^b^ ^,^ ^c^	0.60	25	-	-	-	-	-
4^d^ ^,^ ^e^	0.60	>99	<5	-	-	-	-
5^d^ ^,^ ^f^	0.60	>99	<5	-	-	-	-

a Reaction condition: Compound **1c** (100 μmol), capsule **I** (10 mol %), and 100 mg basic Al_2_O_3_ in 1.0 mL 1,2-DCB at 30 °C for 24 h.

b Compound **1c** (200 μmol), capsule **I** (10 mol %), and 200 mg basic Al_2_O_3_ in 0.33 mL 1,2-DCB at 30 °C for 24 h.

c Using 1.0 equiv. tetrabutyl ammonium bromide to block the capsule **I**.

d Compound **1c** (50 μmol), promoter (2.0 equiv) in 85 μL dry 1,2-DCB at 30 °C for 3 h.

e Using TMSOTf as a promoter.

f Using BF_3_·Et_2_O as a promoter.

### Mechanistic Insights

Having explored the scope of the reaction, to gain a deeper understanding of the macroglycosylation, detailed time-course experiments were performed in 1,2-dichlorobenzene and dichloromethane (Figure 3B; for details, see SI Section 6). The results confirm that the β-selectivity is observed from the onset and is not due to potential postreaction equilibration. Interestingly, in dichloromethane, a distinct new signal appeared at ∼4.23 ppm, which was isolated and identified as the linear dimer **Int-3c** that was linked β-selectively (Figure 3C). This intermediate was successfully isolated in 30% yield when the reaction was worked up after 3 h.

Importantly, when the isolated linear dimer **Int-3c** was resubmitted to the original catalytic conditions, it cleanly cyclized to afford the β,β-cyclic dimer **3c** in 78% isolated yield (90% conversion) after 24 h at 30 °C. These experiments indicate that the cyclic dimer is likely formed via a stepwise mechanism: initial intermolecular dimerization to generate a linear intermediate, followed by intramolecular cyclization as the rate-determining step (Figure 3D).

Several independent observations support that both the macrocyclization and, in the case of macrocyclic dimer formation, the intermolecular dimerization occur inside the capsule rather than in bulk solution:

(1) Spectroscopic evidence for encapsulation. Encapsulation of substrates is evidenced by the emergence of new upfield signals, resulting from anisotropic shielding of the respective nuclei by the capsule walls (see SI section 5 for details).
(2) Control experiments clearly demonstrate the essential role of the capsule in the cyclization (Table 1). In the absence of the capsule, or when the capsule cavity is blocked by a known high-affinity guest for the capsule,^61^ no macrocyclization products were detected. The lack of product formation with the blocked capsule rules out productive reactivity in bulk solution or at the outer surface of the capsule, providing very strong evidence that the transformation requires access to the capsule interior.
(3) Concentration effects. While at 100 mM substrate concentration, some diastereomeric mixtures of macrocyclic products were still detected with conventional promotors (Table 1, entries 8 and 9), only traces were detected at 600 mM (Table 2, entries 4 and 5). This observation further underscores the critical role of the capsule, as at increased concentration, standard activation methods fail to induce macrocyclization.
(4) Stereochemical evidence. The pronounced β-selectivity in both the linear dimer intermediate and the final cyclic products is unprecedented under conventional bulk conditions (Table 1, entries 8–9) without an acyl neighboring-group effect.^64^ Such selectivity therefore rules out a solution-phase pathway with confidence.
(5) Size-selectivity effects further support a confined reaction environment. The observed size-selectivity patterns (Figures 2 and 3A) are fully consistent with a reaction occurring within a confined cavity. A solution-phase process would be incompatible with the selective formation of cyclic monomers or dimers depending on the substrate size. For instance, substrates **2f** and **2g** did not yield any traces of a cyclic dimer, as the capsule is too small to encapsulate two substrates of this size.
(6) Consistency across conditions. All observed trends remain unchanged across a wide range of substrates, further supporting a capsule-confined mechanism rather than a coincidental solution-phase pathway.
(7) Altered kinetics and concentration dependence. The intermolecular reaction was investigated in detail.^55^ An approximately zero-order dependence on nucleophile concentration was observed, consistent with the formation of a host–substrate complex preceding the glycosylation step. Collectively, these complementary findings provide compelling evidence that both key bond-forming steps, intermolecular (for cyclic dimer formation) and intramolecular (for macrocyclization), occur within the capsule cavity rather than in bulk solution.

Finally, we examined why product inhibition is not significant, as reactions typically reach completion within 24 h using 10 mol % of capsule, in contrast to most previous (super)­stoichiometric macrocyclization strategies in supramolecular host systems. Binding constants were measured for both substrate and product. Most substrates proved too reactive for detailed encapsulation studies, so the deactivated ester-containing substrate **9** was chosen for its stability (see SI Section 5). A 1:1 substrate–capsule complex exhibited a binding constant of ∼1.4 × 10^4^ M^–1^, whereas the product **10** bound more weakly, with *K* ≈ 5 × 10^3^ M^–1^. Therefore, despite a slowdown in conversion toward the end of the reaction, full conversion can be obtained using 10 mol % of capsule **I**.

## Summary

In this study, we report a novel and highly effective strategy for the catalytic construction of macroglycosides via glycosylation within a self-assembled supramolecular container. Capsule **I** (i) efficiently catalyzed the formation of medium and large glycosidic macrocycles in yields of up to 87%. (ii) Notably, the method also enabled the selective formation of macrocyclic dimers, architectures that are typically even more synthetically challenging than their monomeric counterparts. The observed selectivity between monomer and dimer formation can be controlled by varying the substrate size and solvent, aligning with the concept of optimal capsule occupancy. (iii) In all cases, the products were obtained with excellent β- and β,β-selectivity, without requiring a C2-directing group on the glucose unit, which is traditionally required for such stereocontrol. The control experiments underscore the vital role of the supramolecular container, as traditional noncapsular conditions result in poor yields and unfavorable stereoselectivity (predominantly α-anomers). (iv) Time-course experiments show that the reaction is intrinsically β-selective from the outset and does not rely on postreaction equilibration. The cyclic dimer arises via a β-linked linear dimer intermediate, with this selectivity preserved during cyclization, resulting in exclusive formation of the β,β-macroglycoside. (v) Most strikingly, the capsule-catalyzed glycosylation proceeded efficiently under very high concentrations (up to 0.60 M), circumventing the need for the high-dilution conditions typically required for macrocyclization. These findings highlight the utility of supramolecular containers in overcoming key limitations in macrocyclization chemistry. In contrast to earlier (super)­stoichiometric cavitand and capsule approaches, the present system benefits from two key factors: (1) the capsule itself functions as the catalyst, actively accelerating the reaction within its confined interior; and (2) product inhibition does not impede turnover, enabling the capsule to be employed in truly catalytic amounts. We anticipate that this work will stimulate a comprehensive investigation into the potential of confinement catalysis across the broad and timely field of macrocyclizations.

## Supplementary Material



## Notes

Experimental details and NMR spectra of new compounds (PDF)
