# Supplementary material for: Confinement Catalysis Enables Macrocyclization at Up to 0.6 M: Selective Formation of Mono- and Dimeric Glycosidic Macrocycles
Source: J Am Chem Soc. 2026 Mar 18;148(12):13473–82. doi: 10.1021/jacs.6c03730 (PMC13047676; doi:10.1021/jacs.6c03730)

Confinement Catalysis Enables Macrocyclization at Up to 0.6 M: Selective Formation of Mono- and Dimeric Glycosidic Macrocycles

Sudip Guria,[1] Julia Bechter,[1] Alessandro Prescimone,[1] Konrad Tiefenbacher*,[1,2]

[1] Department of Chemistry, University of Basel, Mattenstrasse 22, 4058 Basel, Switzerland
[2] Department of Biosystems Science and Engineering, ETH Zurich, Schanzenstrasse 44, 4056 Basel, Switzerland

Table of Contents

1. General information 2

2. Synthesis of catalyst 4

3. Reaction optimization 4

General procedure for the macrocyclization (GP1) 4

3.1. Screening of solvents 4

4. Control experiments 5

4.1. Using β-fluoro glycosyl donor for the catalysis experiment 5

4.2. Capsule blocking experiment 5

4.3. Macroglycosylation by using different promoters 6

5. NMR encapsulation study 6

5.1 Binding study of product 10 8

6. Time-course experiments 10

6.1. Using 1,2-dichlorobenzene as a solvent (condition for selective cyclic monomer) 10

6.2. Using dichloromethane as a solvent (condition for selective cyclic dimer) 11

7. Mechanistic insights 12

7.1. Isolation of linear dimer intermediate (Int-3c) 12

7.2. Catalysis experiment using linear dimer intermediate (Int-3c) 13

8. Crude NMR of cyclization reactions 15

8.1. For compounds 3a 15

8.2. For compounds 2b and 3b 16

8.3. For compounds 2c and 3c 16

8.4. For compounds 2d and 3d 17

8.5. For compound 2e 17

8.6. For compound 2f 18

8.7. For compound 2g 18

8.8. For compound 2h 19

8.9. For compound 2i 19

8.10. For compound 2j 20
8.11. For compound 4 20
8.12. For compound 5 21
8.13. For compound 6 21
8.14. For compound 7 22
8.15. For compound 8 22
8.16. For compound 10 23
9. Products isolated 24
10. Synthesis of cyclization substrates 34
10.1. Synthesis of ether-linked glucose derivatives for the cyclization 34
General procedure for the synthesis of compound S5a-g (GP2) 35
General procedure for the synthesis of compound 1a-g (GP3) 35
10.2. Synthesis of compound 1g 42
10.3. Synthesis of cyclization substrate with secondary alcohol as nucleophile 45
10.4. Synthesis of ether-linked galactose and mannose derivatives for the cyclization 46
10.5. Synthesis of cyclization substrates with benzyl-protected glucose 53
General procedure for the alkylation of primary alcohols (GP4) 53
General procedure for TBS-deprotection (GP5) 54
10.6. Synthesis of common allyl-protected intermediate S23 59
10.7. Synthesis of cyclization substrates with allyl-protected glucose 61
10.8. Synthesis of substrate S27 63
10.9. Synthesis of dimeric compound 10 64
11. Occupancy estimations 66
12. References 68
Appendix A: Crystallographic details 69
Appendix B: NMR spectra of substrates 76

1. General information

Experimental: Reactions were carried out under an argon atmosphere in dried glassware unless otherwise indicated. For the cyclization reactions, no precaution against air was taken. Analytical thin-layer chromatography (TLC) was performed on Merck silica gel 60 F_{254} aluminum sheets, which were analyzed after exposure to standard staining solutions (CAM: cerium ammonium molybdate, anisaldehyde or basic $KMnO_4$). All NMR experiments were performed on a Bruker Avance Neo and a Bruker Avance III HD NMR spectrometer operating at 500 MHz and 600 MHz proton frequency, respectively. The instruments were equipped with a direct observe 5-mm BBFO smart probe (500 MHz) or a five-channel cryogenic 5 mm QCI probe (600 MHz). All probes were equipped with actively shielded z-gradients (10 A). The experiments were performed at 298 K. Chemical shifts of ^{1}H NMR and ^{13}C NMR are given in ppm. The following solvent residual signals of the deuterated solvents were used as reference: $CDCl_3$: 7.26 ppm (δ^{1}H), 77.16 ppm (δ^{13}C), acetone-*d6*: 2.09 ppm (δ^{1}H), 30.60 ppm ($\delta^{13}CH_3$) or 207.07 ppm (δ^{13}CO). Coupling constants (*J*) are reported in Hertz (Hz). Standard

abbreviations indicating multiplicity were used as follows: s (singlet), d (doublet), t (triplet), dd (doublet of doublets), m (multiplet). Infrared spectra were recorded on a Perkin Elmer Spectrum Two FT-IR Spectrometer. High-resolution mass spectra were obtained on a Thermo Scientific LTQ-FT Ultra via electrospray ionization (ESI) or a Finnigan MAT 8200 (EI) (ESI source parameters for positive polarity mode were: spray voltage, 4.0 kV; capillary temperature, 275 °C; capillary voltage, 48 V; and tube lens, –120 V).

Sources of chemicals: Deuterated chloroform ($CDCl_3$, 99.8%) was purchased from Cambridge Isotope Laboratories (stabilized over silver foil). Acetic acid, aluminum oxide (activated, basic, Brockmann I), benzene-d_6, ethanol (99.9%), ethylmagnesium bromide (3.0 M sol. in diethyl ether), hexylmagnesium bromide (0.8 M sol. in THF), iso-butylmagnesium bromide (2.0 M sol. in diethyl ether), *n*-hexane (HPLC grade), methanol, methylmagnesium bromide (3.0 M sol. in diethyl ether), pentylmagnesium bromide (2.0 M sol. in diethyl ether), phenol red, semicarbazide hydrochloride, tetrabutylammonium bromide, trimethylamine were purchased from Sigma-Aldrich. Anhydrous diethyl ether (Et_2O), tetrahydrofuran (THF), dimethyl formamide (DMF) and anhydrous DCM were purchased from Acros Organics. Silica gel (0.040-0.063 mm, 230-400 mesh ASTM) was purchased from Merck KGaA. *n*-Decane was purchased from Fluka. All chemicals were used as received. Transfer of liquids with a volume ranging from 1 to 10 µL or from 10 to 100 µL was performed with a microman M1 pipette (Gilson, systematic error: 1.40% - 1.60%) equipped with 10 µL or 100 µL pipette tips, respectively.

2. Synthesis of catalyst

Synthesis of resorcin[4]arene: Synthesis of resorcin[4]arene was carried out as previously described.[1]

3. Reaction optimization

General procedure for the macrocyclization (GP1)

To a stirred solution of resorcin[4]arene (33.2 mg, 30.0 µmol, corresponding to 5.00 µmol of the capsule **I**, 10 mol%) in 0.5 mL of the respective solvent, two balls of 4 Å MS (ca. 66 mg) were added, and the mixture was kept in an airtight vessel for 2 d. To the gel formed, compound **1c** was added (16.2 mg, 50.0 µmol, 1.0 equiv.) and stirred for 2 minutes to break down the gel and produce a clear solution. Immediately, this mixture (without the molecular sieves) was transferred via a glass pipette to a separate 2 mL vial containing 50 mg dry basic Al_2O_3. Then, the vial was placed in a preheated metal block (30 °C) and stirred for 24 h. To estimate the material loss in the transfer operation, 0.5 mL of acetone-d_6 was added to the first empty vial, and dimethyl terephthalate (approx. 10 mg, but the exact amount was noted) was added to it as an internal standard. Afterward, the crude NMR was measured (t = 0) to determine how much starting material (**1c**) was lost during the compound transfer.

After 24 h, the reaction was stopped by adding 0.3 mL of acetone and 50 mg of $NaHCO_3$, stirring for five minutes. All solid components were removed by filtration and concentrated under reduced pressure. A ^{1}H NMR of the crude mixture was recorded (t = 24 h) in acetone-d_6 using dimethyl terephthalate (approx. 10 mg, but the exact amount was noted) as an internal standard to determine the conversion and yield of the reaction.

Note 1: *As the ^{1}H NMR signals were broad in some cases (likely traces of HF), 25 mg solid $NaHCO_3$ was added to the NMR tube for both t = 0 and t = 24 h ^{1}H NMR measurements.*

Note 2: *The catalytic experiments for the substrate scope were conducted on a 100 µmol scale in 1.0 mL of the indicated solvent.*

3.1. Screening of solvents

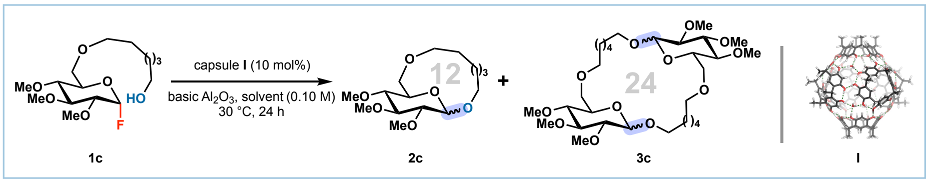

Entry	Solvent	Conversion (%)	Comb. Yield (%)	2c/3c	*β*/*α* (2c)	*β*,*β*/(*α*,*β* + *α*,*α*) (3c)
1	chloroform-*d* ($CDCl_3$)	>99	91	44:56	>98:2	>98:2
**2**	**dichloromethane (DCM)**	**>99**	**69 (52)[c]**	**20:80**	**>98:2**	**>98:2**
3	1,1,2,2-tetrachloro ethane	73	37	35:65	>98:2	>98:2
4	toluene	>99	32	53:47	>98:2	>98:2
5[a,b]	α,α,α-trifluoro toluene ($PhCF_3$)	70	47	17:83	>98:2	>98:2
6	mesitylene	48	22	18:82	>98:2	>98:2
7	fluorobenzene	>99	86	44:56	>98:2	>98:2
8	chlorobenzene	>99	83	61:39	>98:2	>98:2
9[a]	1,2-difluorobenzene (1,2-DFB)	>99	87	69:31	>98:2	>98:2
**10**	**1,2-dichlorobenzene (1,2-DCB)**	**>99**	**79 (70)[d]**	**89:11**	**>98:2**	**>98:2**
11	1,3-dichlorobenzene 1,3-DCB	>99	69	83:17	>98:2	>98:2

Standard reaction condition: Compound **1c** (50.0 µmol), capsule **I** (10 mol%), and 50 mg basic Al_2O_3 in 0.5 mL solvent at 30 °C for 24 h. (a) Bad solubility (b) After 3 d, 85% conversion. (c) Isolated yield of **3c**. (d) Isolated yield of **2c**.

4. Control experiments

4.1. Using *β*-fluoro glycosyl donor for the catalysis experiment

The β-fluoro glycosyl donor **S8** was prepared with benzyl protecting groups, as the methylated version proved unstable. No significant conversion to the macrocycle was observed, indicating that only the *α*-fluoride is reactive inside the capsule.

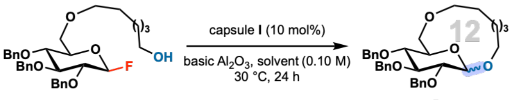

S8 **5**

According to the **GP1**, substrate **S8** (27.6 mg, 50.0 µmol, 1.0 equiv.) in 0.5 mL solvent was treated with resorcin[4]arene (33.2 mg, 30.0 µmol, corresponding to 5.00 µmol of the capsule **I**, 10 mol%). After 24 h, the reaction was stopped by adding 0.3 mL of acetone and 50 mg $NaHCO_3$, stirring for five minutes. All solid components were removed by filtration and concentrated under reduced pressure. A ^{1}H NMR of the crude mixture was recorded (t = 24 h) in acetone-d_6 using dimethyl terephthalate (approx. 10 mg, but the exact amount was noted) as an internal standard to determine the conversion and yield of the reaction.

Entry	Solvent	Conversion (%)	NMR Yield (%)
1	dichloromethane	25	<2
2	chloroform-*d*	20	<2
3	1,2-dichlorobenzene	14	<1

4.2. Capsule blocking experiment

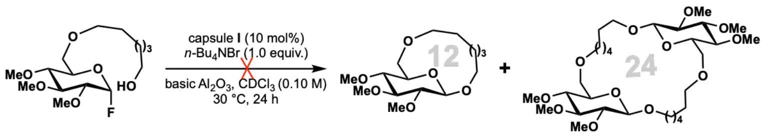

1c **2c** **3c**

To a stirred solution of resorcin[4]arene (33.2 mg, 30.0 µmol, corresponding to 5.00 µmol of the capsule **I**, 10 mol%) in 0.5 mL of chloroform-*d*, two balls of 4 Å MS (ca. 66 mg) were added, and the mixture was kept in an airtight vessel for 2 days. To the gel formed, compound **1c** was added (16.2 mg, 50.0 µmol, 1.0 equiv.) and stirred for 2 minutes to break down the gel and produce a clear solution. Immediately, this mixture (without the molecular sieves) was transferred via a glass pipette to a separate 2 mL vial containing 50 mg dry basic Al_2O_3 and tetrabutylammonium bromide salt (16.1 mg, 50.0 µmol, 1.0 equiv.). Then, the vial was placed in a preheated metal block (30 °C) and stirred for 24 h. To estimate the material loss in the transfer operation, 0.5 mL of acetone-d_6 was added to the first empty vial, and dimethyl terephthalate (approx. 10 mg, but the exact amount was noted) was added to it as an internal standard. Afterward, the crude NMR was measured (t = 0) to determine how much starting material (**1c**) was lost during the compound transfer.

After 24 hours, the reaction was stopped by adding 0.3 mL of acetone-d_6 and 50 mg of $NaHCO_3$, stirring for five minutes. Dimethyl terephthalate (approx. 10 mg, but the exact amount was noted) was then added to the vial as an internal standard and stirred for an additional five minutes. A ^{1}H NMR of the crude mixture was recorded (t = 24 h) in acetone-d_6, which indicated no desired product formation.

Note: *As the ^{1}H NMR signals were broad in some cases (likely traces of HF), 25 mg solid $NaHCO_3$ was added to the NMR tube for both t = 0 and t = 24 h NMR measurements.*

4.3. Macroglycosylation by using different promoters

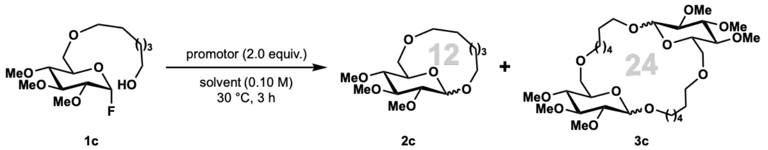

Compound **1c** (16.2 mg, 50.0 µmol, 1.0 equiv.) was dissolved in 0.5 mL of dry chloroform-*d* or dichloromethane. Promoter $BF_3.Et_2O$ (12.3 µL, 100 µmol, 2.0 equiv.) or TMSOTf (18.1 µL, 100 µmol, 2.0 equiv.) was added at room temperature and stirred at 30 °C for 3 h. After the completion of the reaction, a saturated $NaHCO_3$ solution was added to destroy the excess reagent and then diluted with dichloromethane. Dimethyl terephthalate (approx. 10 mg, but the exact amount was noted) was then added to the vial as an internal standard and stirred for five more minutes. Afterward, the organic phase was filtered through a glass pipette filled with $MgSO_4$ to remove any remaining water. Then the solvent was evaporated under reduced pressure, redissolved in acetone-d_6, and proton NMR was recorded to determine the yield and selectivity of the product.

Entry	promotor	solvent	overall yield (%)	2c/3c	β/α (2c)	$\beta,\beta/(\alpha,\beta + \alpha,\alpha)$ (3c)
1	$BF_3.Et_2O$	chloroform-*d*	41	14:86	13:87	13:87
2	TMSOTf	dichloromethane	27	19:81	37:63	18:82

The control experiments produced the desired macrocycles only in poor yield and insufficient selectivity (mostly α-selective). According to LC-MS analysis, the low yield mainly results from substrate hydrolysis and linear oligomerization.

5. NMR encapsulation study

Most substrates are too reactive for the encapsulation study. Thus, the deactivated, ester-containing substrate **9** was chosen for this study.

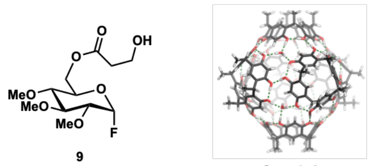

9
Guest **(G)**

Capsule **I**
Host **(H)**

An NMR tube was charged with resorcin[4]arene (33.2 mg, 30.0 µmol, corresponding to 5.00 µmol of capsule **I**, 10 mol% capsule **I** (**H**)) in 500 µL of water-saturated $CDCl_3$ (10 mM). In a 4 mL vial, a stock solution was prepared using substrate **9** (42.7 mg, 144 µmol, 200 mM) and resorcin[4]arene (47.8 mg, 43.2 µmol, corresponding to 7.19 µmol of the capsule **I** (**H**), 10 mM) in 719 µL of water-saturated $CDCl_3$. Afterward, the NMR tube was inserted into an NMR spectrometer (600 MHz), and ^{1}H NMR spectra were recorded, adding from 0-5.00 equivalents of guest, maintaining the host concentration constant (Fig S1).

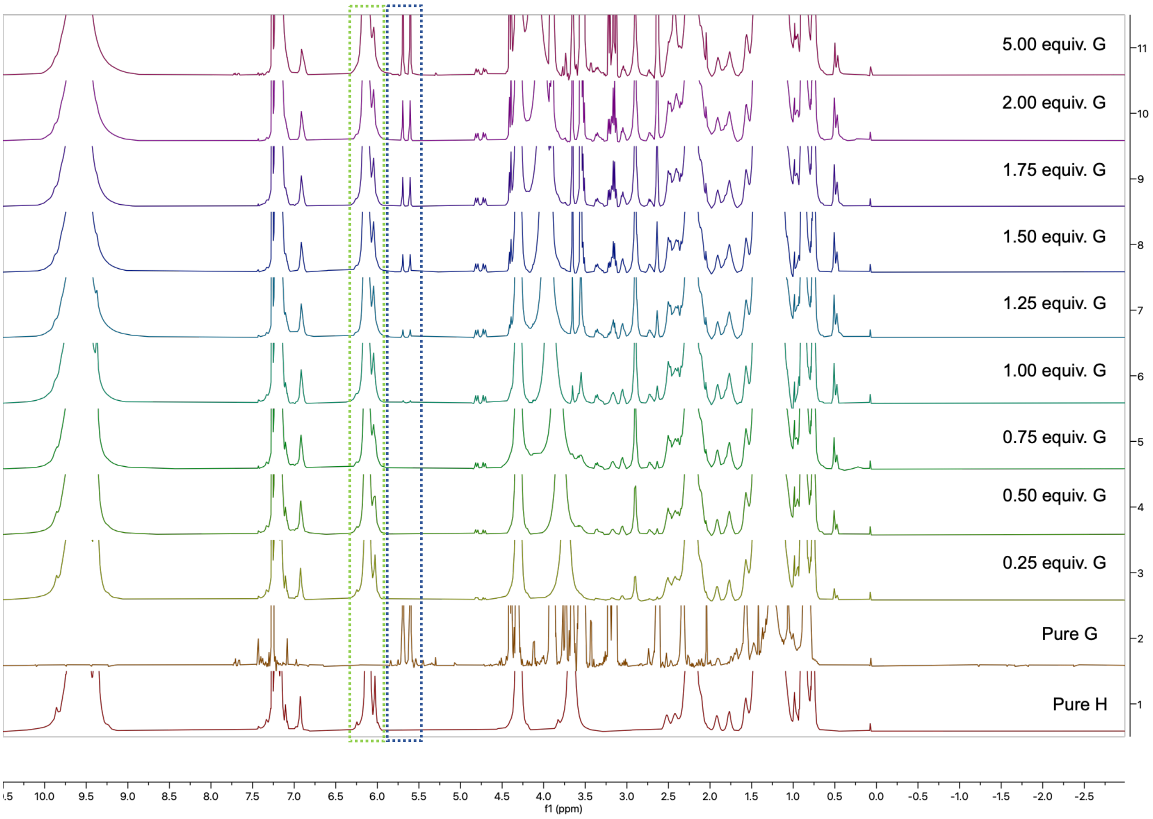

***Figure S1**: Stacked ^{1}H NMR spectra* (at 298K) *of free host (Capsule **I**, H), free guest (**9**, G), and host-guest complex (HG) by varying the amount of guest **9** from 0 to 5.00 equivalents relative to the host molecule.*

The ^{1}H NMR spectrum of each measurement was analyzed in the following way: the integral of the free guest signal (1H, C-H at 5.65 ppm marked as blue box), and the integral of the host signal (24H, C-H at 6.12 ppm marked as green box) as the internal reference are used to calculate the concentration of the free guest [G] (Table 1). Encapsulated guest signals, due to the slow rotation of the guest inside the capsule, are often difficult to identify and integrate. Therefore, we base our binding studies exclusively on the free guest.

Entry	Guest equivalent	Unbound guest (mM)	Calculated host-guest complex (mM)
1	0	0	0
2	0.25	0	2.5
3	0.50	0.2	4.8
4	0.75	0.2	7.3
5	1.00	0.8	9.2
6	1.25	1.2	11.3
7	1.50	3.7	11.3
8	1.75	6.0	11.5
9	2.00	7.9	12.1
10	5.00	34.4	15.6

***Table 1:** Data for the calculated host-guest complex based on the amount of guest and the residual unbound guest concentration from the ^{1}H NMR study.*

A plot was created comparing the calculated host-guest complex with the amount of guest (Fig S2). It indicates a higher binding constant for the 1:1 H-G complex, as compared to the 1:2 complex.

Nevertheless, all data points above 1.00 equiv. of guest, indicate that the encapsulation of two guests is observed.

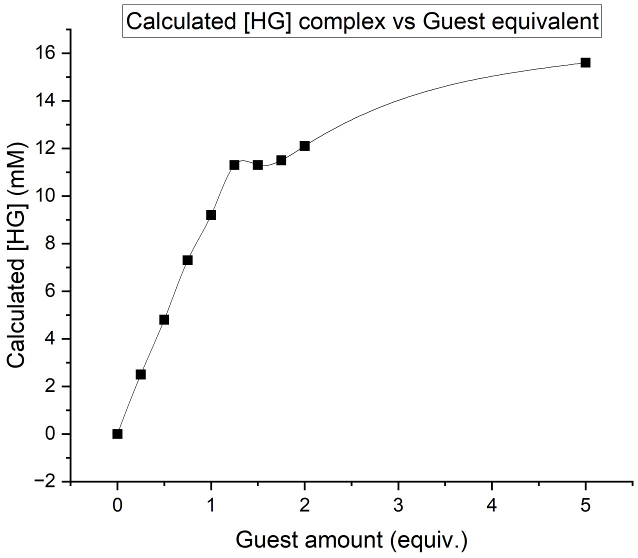

Figure S2: *Plot between calculated host-guest complex and guest equivalent.*

This enables the determination of the binding constant for 1:1 binding via:

$$[HG] = [G]_0 - [G]$$

$$[H] = [H]_0 - [HG]$$

$$K = \frac{[HG]}{[G] \times [H]}$$

The binding constants for the 1:1 host-guest measured in this section are summarized as follows using the above formula:

Compound **9** (**G**)	0.75 equiv.	1.00 equiv.
K_1 (M^{-1})	13518	14375

5.1 Binding study of product 10

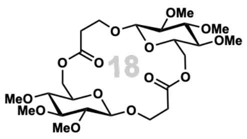

10
Guest **(G)**

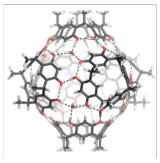

Capsule **I**
Host **(H)**

In a 4 mL vial, resorcin[4]arene (14.4 mg, 13.0 µmol, corresponding to 2.17 µmol of capsule **I**) and compound **10** (1.20 mg, 2.17 µmol) was dissolved in 435 µL of water-saturated $CDCl_3$ (5.00 mM)and stirred for 30 minutes. Then the mixture was transferred into an NMR tube and ^{1}H NMR spectra were recorded (Fig S3).

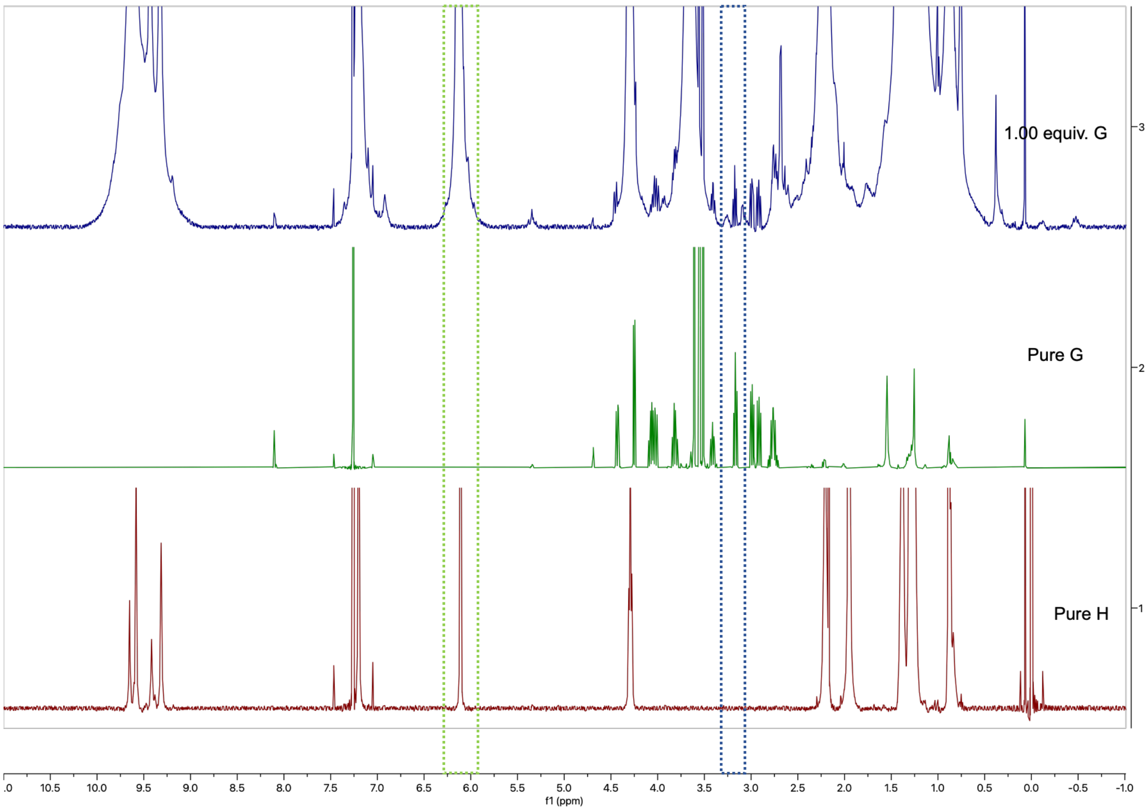

***Figure S3**: Stacked ^1H NMR spectra* (at 298K) *of free host (Capsule **I**, H), free guest (**10**, G), and host-guest complex (HG) by mixing 1:1 host (H) and guest (G) molecules.*

The ^{1}H NMR spectrum of the host-guest complex was analyzed in the following way: the integral of the free guest signal (1H, C-H at 3.17 ppm marked as blue box), and the integral of the host signal (24H, C-H at 6.12 ppm marked as green box) as the internal reference are used to calculate the concentration of the free guest [G] (Table 2). Using the above-mentioned formula, the calculated binding constant of the compound is 5062 M^{-1}.

Unbound guest (mM)	Calculated host-guest complex (mM)	Binding constant K_2 (M^{-1})
0.90	4.10	5062

***Table 2:** Data used for calculating the binding constant of the compound **10** from the ^1H NMR study.*

The ^{1}H NMR binding studies show that the substrate exhibits a binding constant approximately threefold higher than that of the product. Thus, although the catalyst maintains a 3-fold thermodynamic preference for the substrate, conversion will slow towards the end of the reaction. Nevertheless, full conversion was observed within 7 days for this specific example.

6. Time-course experiments

6.1. Using 1,2-dichlorobenzene as a solvent (condition for selective cyclic monomer)

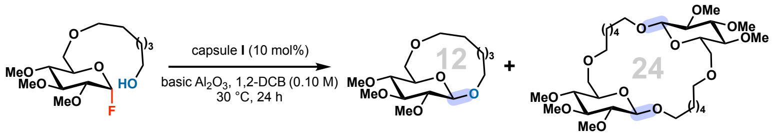

1c 2c 3c

To a stirred solution of resorcin[4]arene (33.2 mg, 30.0 μmol, corresponding to 5.00 μmol of the capsule **I**, 10 mol%) in 0.5 mL of 1,2-dichlorobenzene, two balls of 4 Å MS (ca. 66 mg) were added, and the mixture was kept in an airtight vessel for 2 days. To the gel formed, compound **1c** was added (16.2 mg, 50.0 μmol, 1.0 equiv.), and the mixture was stirred for 2 minutes to break down the gel and produce a clear solution. Immediately, this mixture (without the molecular sieves) was transferred via a glass pipette to a separate 2 mL vial containing 50 mg dry basic Al_2O_3. Then, the vial was placed in a preheated metal block (30 °C) and stirred. Then, in an NMR tube containing approximately 25 mg of $NaHCO_3$, 20 μL of the reaction mixture was taken, dissolved in 0.4 mL of acetone-d_6, and ^{1}H NMR was recorded at t = 0, 2, 4, 6, 8, and 24 hours. All ^{1}H NMR spectra were stacked and compared with pure *α*/*β*-monomer **2c**, and *β*,*β*-/*α*,*β*-dimer **3c**; Fig S4). These spectra clearly suggested that the highly *β*- or *β*,*β*-selectivity arises from the beginning of the reaction, and not due to post-reaction equilibration.

Note-1: *As the ^{1}H NMR signals were broad in some cases (likely traces of HF), 25 mg solid $NaHCO_3$ was added to the NMR tube for all ^{1}H NMR measurements.*

Note-2: *Compound α-***2c** contains slightly *β*-**2c** as an impurity.

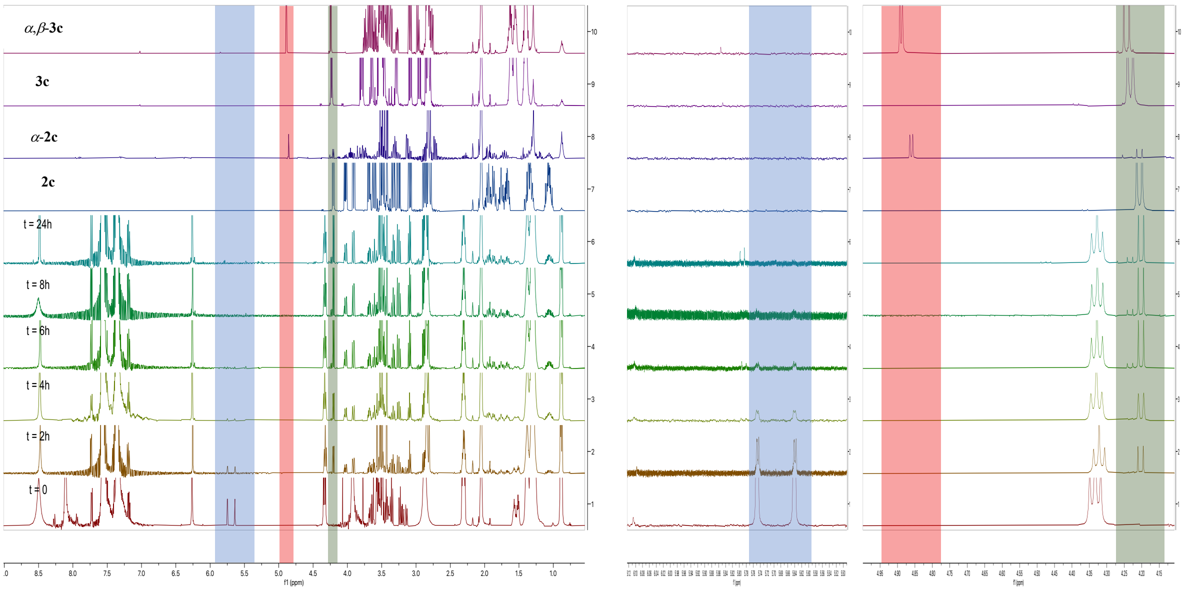

Figure S4: *Stacked ^{1}H NMR spectra of the time-course experiments in 1,2-dichlorobenzene, shown alongside spectra of the pure compounds for comparison.*

6.2. Using dichloromethane as a solvent (condition for selective cyclic dimer)

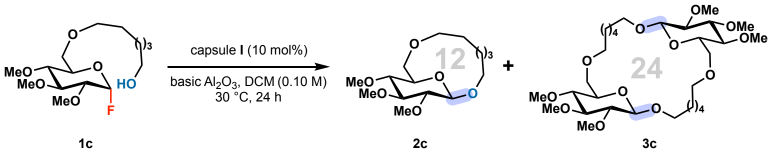

To a stirred solution of resorcin[4]arene (33.2 mg, 30.0 µmol, corresponding to 5.00 µmol of the capsule **I**, 10 mol%) in 0.5 mL of dichloromethane, two balls of 4 Å MS (ca. 66 mg) were added, and the mixture was kept in an airtight vessel for 2 days. To the dry catalyst, compound **1c** was added (16.2 mg, 50.0 µmol, 1.0 equiv.). The mixture was then stirred for 2 minutes to produce a clear solution. Immediately, this mixture (without the molecular sieves) was transferred via a glass pipette to a separate 2 mL vial containing 50 mg dry basic Al_2O_3. Then, the vial was placed in a preheated metal block (30 °C) and stirred. Then, in a 4 mL vial, 20 µL of the reaction mixture was taken and dissolved in 0.4 mL of acetone. Remove the solvent mixture under reduced pressure (to avoid a large solvent signal in the ^{1}H NMR spectra), redissolve in 0.5 mL of acetone-d_6, and transferred into an NMR tube containing approximately 25 mg of $NaHCO_3$. ^{1}H NMR was recorded at t = 0, 2, 4, 6, 8, and 24 hours. All ^{1}H NMR spectra were stacked and compared with pure *α*/*β*-monomer **2c**, and *β*,*β*-/*α*,*β*-dimer **3c**; Fig S5). These spectra clearly suggested that the highly *β*- or *β*,*β*-selectivity arises from the beginning of the reaction, and not due to post-reaction equilibration.

Note-1: *As the ^{1}H NMR signals were broad in some cases (likely traces of HF), 25 mg solid $NaHCO_3$ was added to the NMR tube for all ^{1}H NMR measurements.*

Note-2: *Compound α-***2c** contains slightly *β*-**2c** as an impurity.

Note-3: *During the formation of the cyclic dimer, the initial linear dimer is formed, which causes overlaps in the anomeric proton signal region (for both the starting material and the cyclic dimer).*

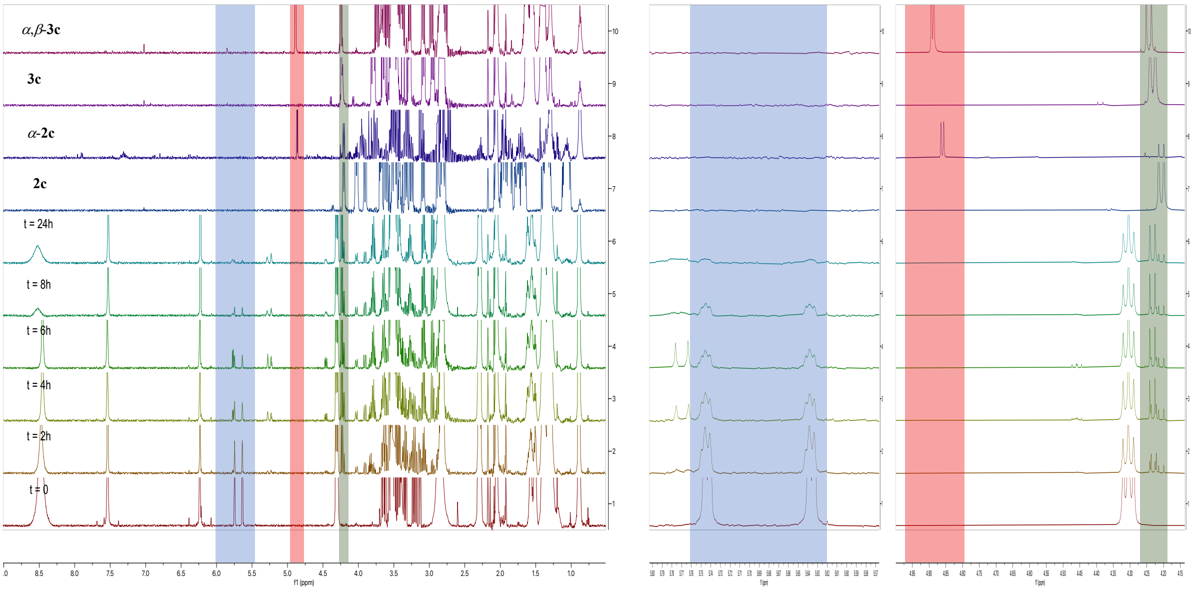

Figure S5: *Stacked ¹H NMR spectra of the time-course experiments in dichloromethane, shown alongside spectra of the pure compounds for comparison.*

7. Mechanistic insights

7.1. Isolation of linear dimer intermediate (Int-3c)

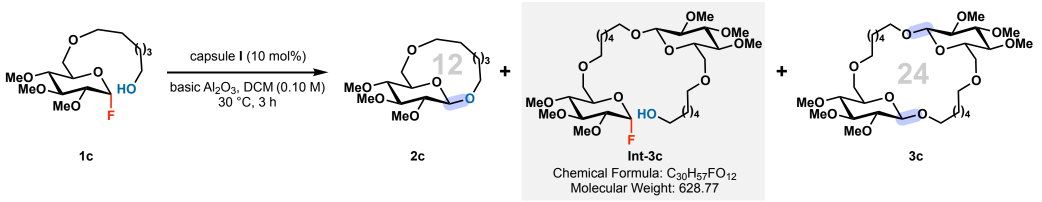

The catalytic reaction was performed using the **GP1** and the substrate **1c** (155 mg, 478 µmol, 1.0 equiv.) in 5 mL dichloromethane. After 3 h, the reaction was stopped by adding 2.0 mL of acetone and 100 mg of $NaHCO_3$, stirring for five minutes. All solid components were removed by filtration and concentrated under reduced pressure. A ^{1}H NMR of the crude mixture was recorded (t = 3 h) in acetone-d_6. Afterward, all crude material was collected in a 50 mL round-bottom flask and dried properly. Then, 20 mL of acetonitrile was added, and the mixture was sonicated for 5 minutes. The flask was then kept at -20 °C for 15 minutes. Then, all solids were filtered off, and the filtrate was dried under reduced pressure. This process was repeated one more time. Afterward, the crude material was purified using silica gel column chromatography (20-80% EtOAc in CyH) to yield the linear dimer intermediate **int-3c** (45.6 mg, 72.5 µmol, 30% yield) as a colourless gummy liquid.

^{1}H NMR spectra of the crude reaction mixture were stacked and compared with pure *β*-monomer **2c**, *β,β*-dimer **3c** as well as linear dimer **Int-3c** (Fig S6). These spectra clearly suggested that the highly *β,β*-selective **3c** forms through a highly *β*-selective linear dimer **Int-3c** intermediate.

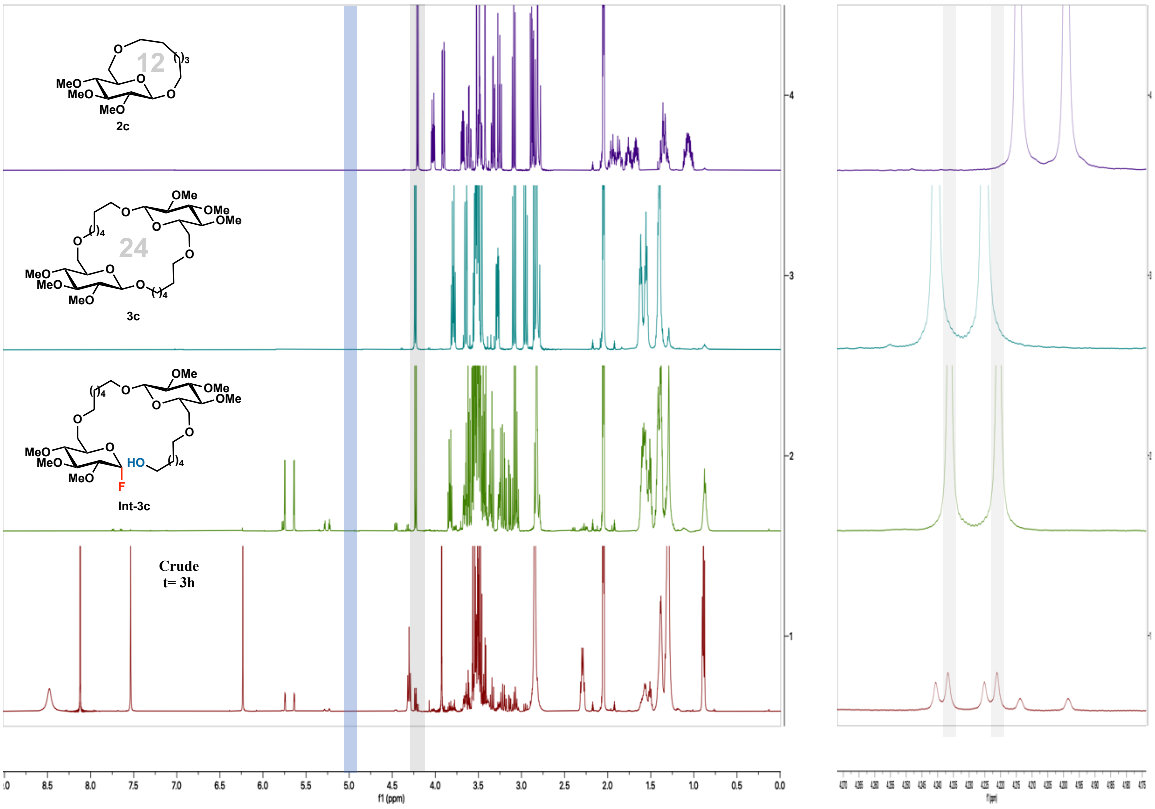

Figure S6: *Stacked ^{1}H NMR spectrum after 3h in dichloromethane , compared with spectra of the isolated intermediate **Int-3c** and pure **3c** and **2c**.*

R$_f$ 0.28 (3:7 cyclohexane: EtOAc)

^{1}H NMR (500 MHz, acetone-d_6, 298K) ^{1}H NMR (500 MHz, Acetone) δ 5.69 (dd, J = 53.7, 2.7 Hz, 1H), 4.23 (d, J = 7.8 Hz, 1H), 3.83 (dt, J = 9.6, 6.4 Hz, 1H), 3.68 – 3.57 (m, 4H), 3.57 (s, 3H), 3.55 (q, J = 1.2 Hz, 1H), 3.54 (s, 3H), 3.54 – 3.52 (m, 2H), 3.51 (s, 3H), 3.50 (s, 3H), 3.49 (s, 3H), 3.48 (d, J = 4.5 Hz, 2H), 3.47 (s, 3H), 3.46 – 3.41 (m, 3H), 3.38 – 3.31 (m, 2H), 3.28 – 3.24 (m, 1H), 3.20 (s, 1H), 3.16 (ddd, J = 26.0, 9.6, 2.7 Hz, 1H), 3.07 (dd, J = 9.1, 7.8 Hz, 2H), 1.65 – 1.47 (m, 9H), 1.47 – 1.35 (m, 8H).

^{19}F NMR (470 MHz, acetone-d_6, 298K) δ -150.40.

^{13}C NMR (126 MHz, acetone-d_6, 298K) δ 105.9 (d, J = 224.7 Hz), 104.1, 87.4, 85.0, 83.9, 82.2 (d, J = 24.8 Hz), 80.5, 79.3, 75.5, 73.8 (d, J = 4.1 Hz), 72.0, 71.9, 70.5, 69.9, 69.7, 62.4, 60.9, 60.8, 60.7, 60.4, 58.9, 33.8, 30.7, 30.5, 30.5, 26.9, 26.7, 26.7, 26.5 (one carbon signal is missing due to overlap).

HRMS (ESI$^+$) m/z calculated for $C_{30}H_{61}FNO_{12}$ [M + NH_4^+]: 646.4172; found 646.4185.

7.2. Catalysis experiment using linear dimer intermediate (Int-3c)

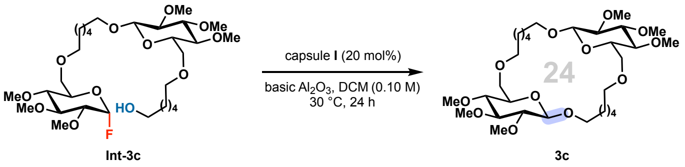

To a stirred solution of resorcin[4]arene (16.6 mg, 15.0 µmol, corresponding to 2.50 µmol of the capsule **I**, 20 mol%) in 0.25 mL of the respective solvent, two balls of 4 Å MS (ca. 66 mg) were added, and the mixture was kept in an airtight vessel for 2 d. To the dry catalyst, intermediate **Int-3c** was added (8.1 mg, 12.9 µmol, 1.0 equiv.) and stirred for 2 minutes to break down the gel and produce a clear solution. Immediately, this mixture (without the molecular sieves) was transferred via a glass pipette to a separate 2 mL vial containing 25 mg of dry basic Al_2O_3. Then, the vial was placed in a preheated metal block (30 °C) and stirred for 24 h. To estimate the material loss in the transfer operation, 0.5 mL of acetone was added to the first empty vial, and dimethyl terephthalate (approx. 5 mg, but the exact amount was noted) was added to it as an internal standard. Then the solvent was evaporated and the crude NMR was measured (t = 0) to determine, using acetone-d_6, how much starting material (**Int-3c**) was lost during the compound transfer (resulting in 12.2 µmol of reactive **Int-3c** present after transfer).

After 24 h, the reaction was stopped by adding 0.5 mL of acetone and 50 mg of $NaHCO_3$, stirring for five minutes. All solid components were removed by filtration and concentrated under reduced pressure. A ^{1}H NMR of the crude mixture was recorded (t = 24 h) in acetone-d_6 using dimethyl terephthalate (approx. 5 mg, but the exact amount was noted) as an internal standard. ^{1}H NMR analysis indicated 90% conversion of the reaction, affording the cyclic dimer **2c** in 78% isolated yield.

Note 1: *As the ^{1}H NMR signals were broad in some cases (likely traces of HF), 25 mg solid $NaHCO_3$ was added to the NMR tube for both t = 0 and t = 24 h ^{1}H NMR measurements.*

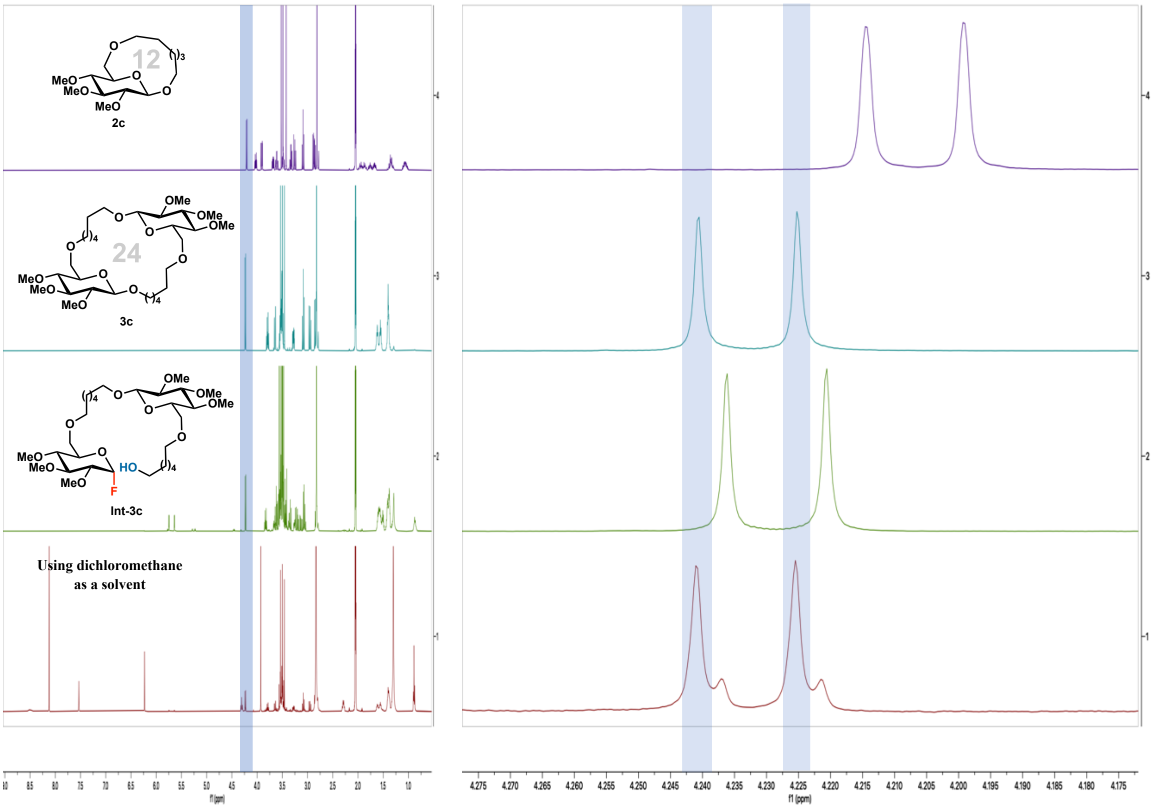

Figure S7: *Crude ¹H NMR spectrum of catalysis experiment after 24h in dichloromethane , compared with spectra of the isolated intermediate* ***Int-3c*** *and pure* ***3c*** *and* ***2c****.*

8. Crude NMR of cyclization reactions

From Figures S4 and S5, it is evident that the anomeric proton of the α-isomer (both for the cyclic monomer and the cyclic dimer) consistently appears in the approximately 4.8-5.0 ppm range. In all crude ^{1}H-NMR spectra, it was evident that there is no signal in that range, indicating high β-selectivity in all catalysis experiments.

8.1. For compounds 3a

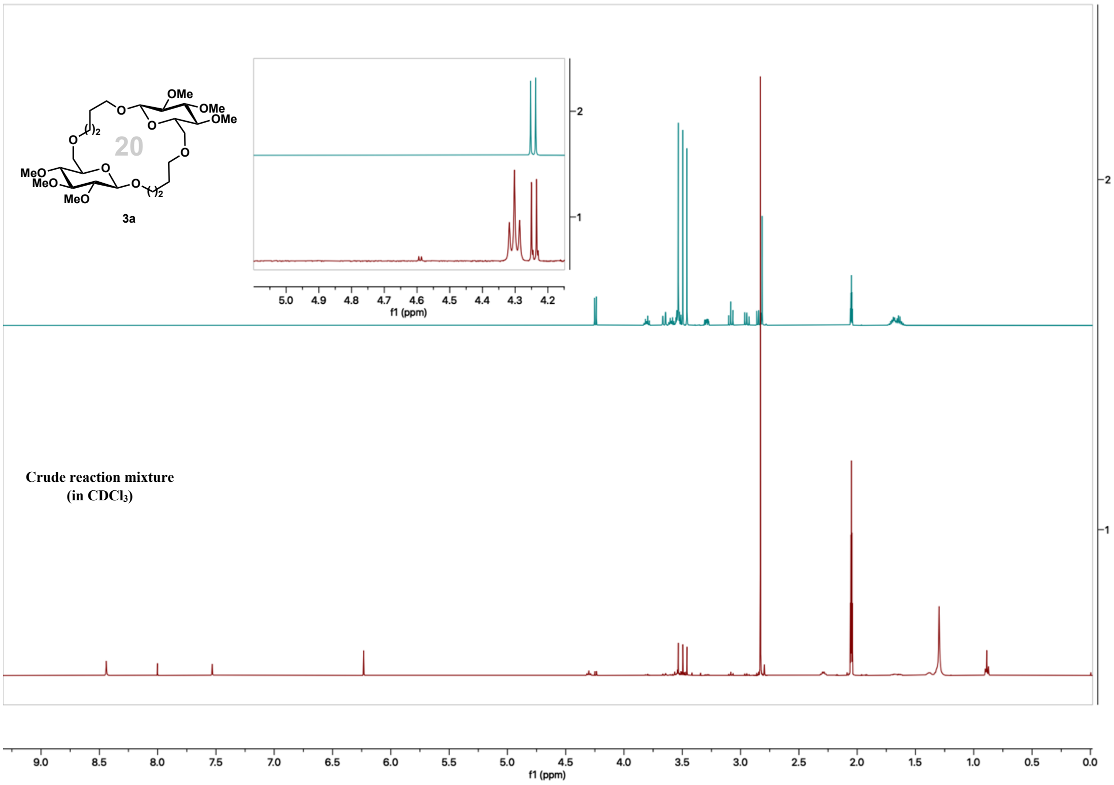

8.2. For compounds 2b and 3b

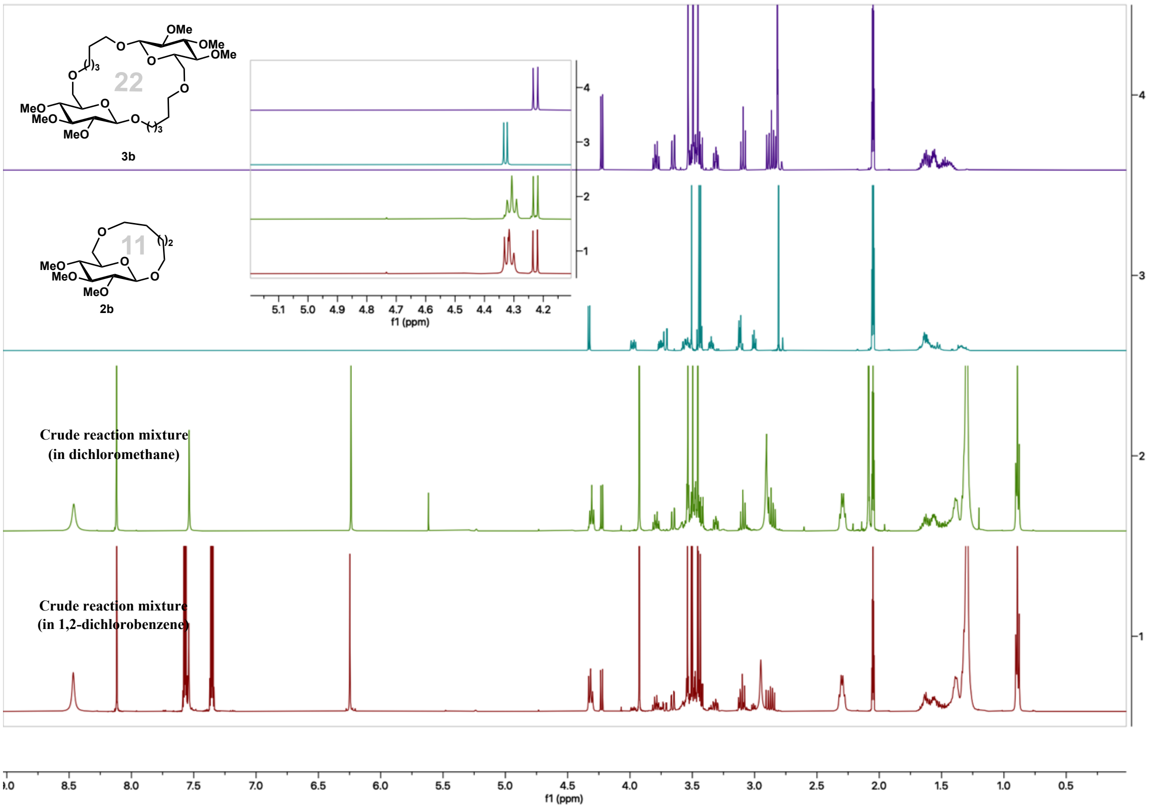

8.3. For compounds 2c and 3c

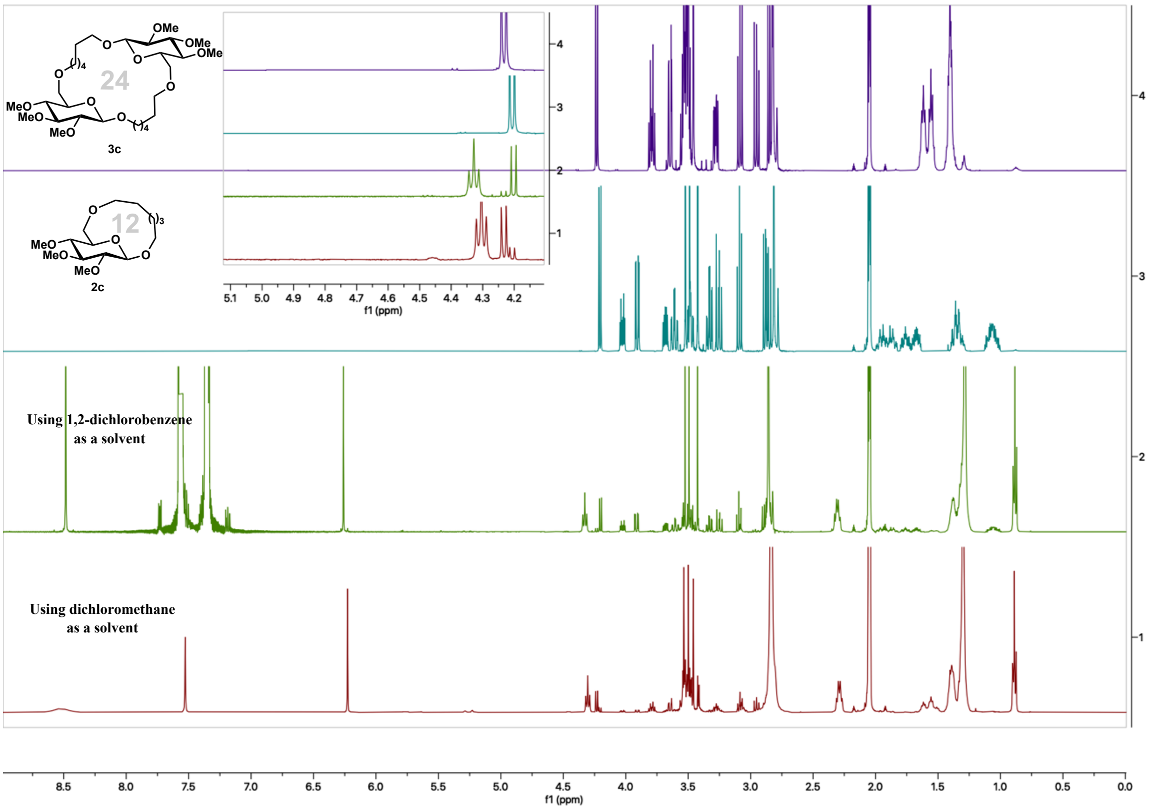

8.4. For compounds 2d and 3d

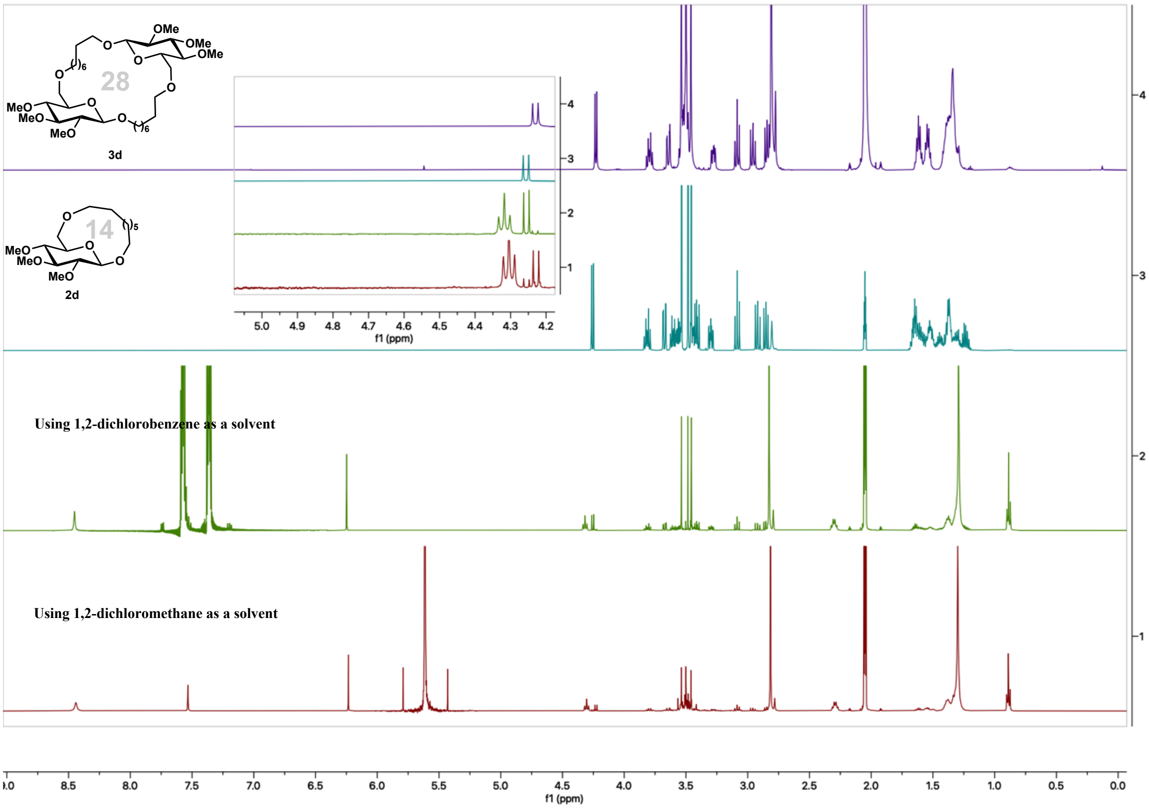

8.5. For compound 2e

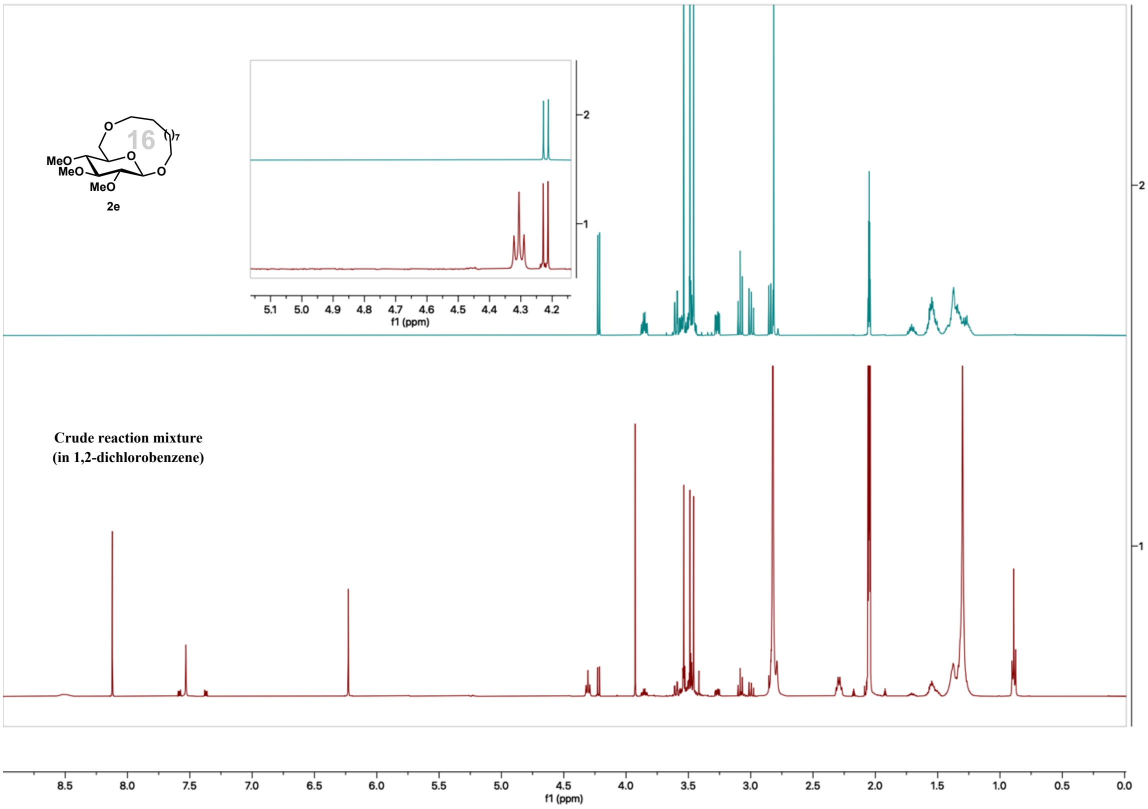

8.6. For compound 2f

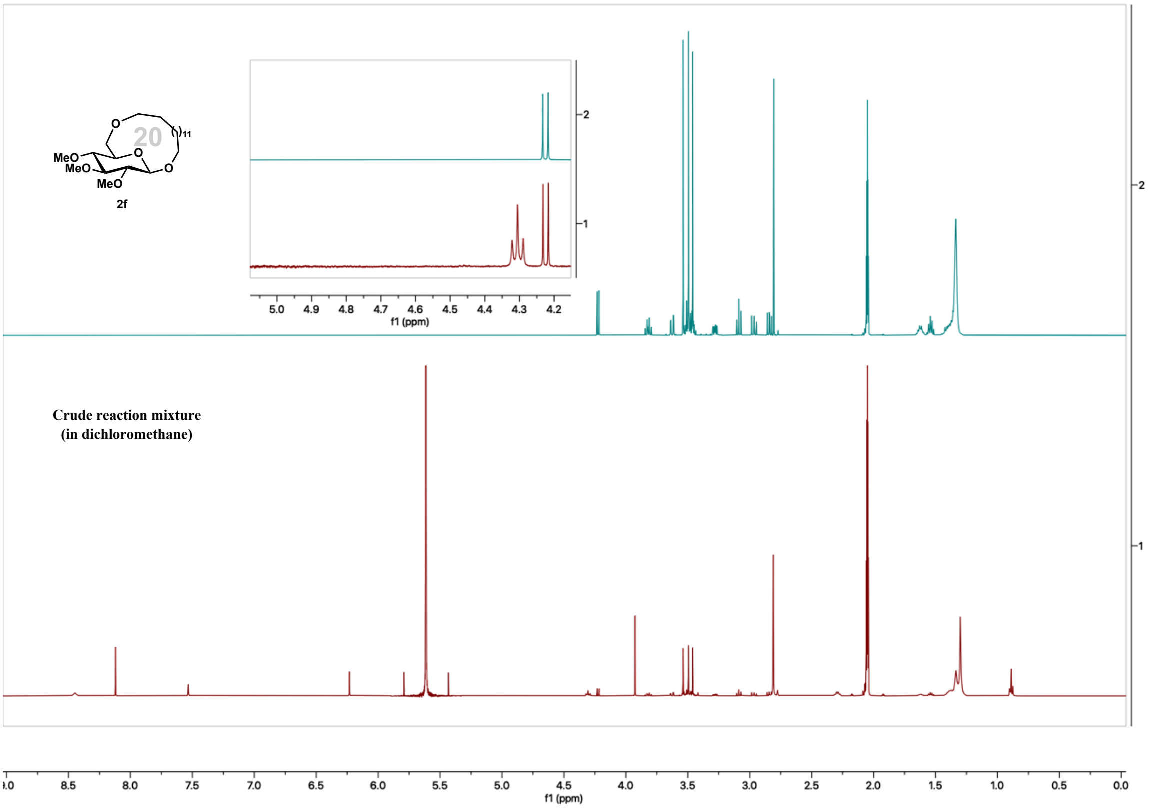

8.7. For compound 2g

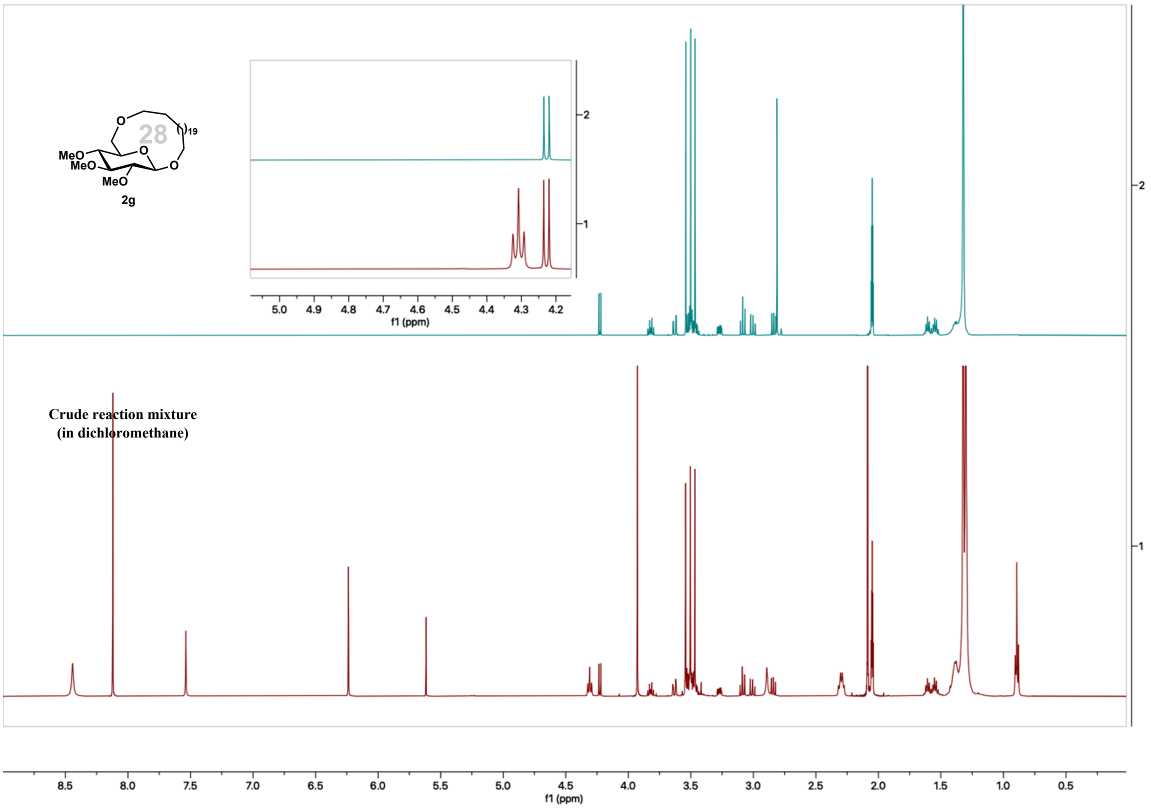

8.8. For compound 2h

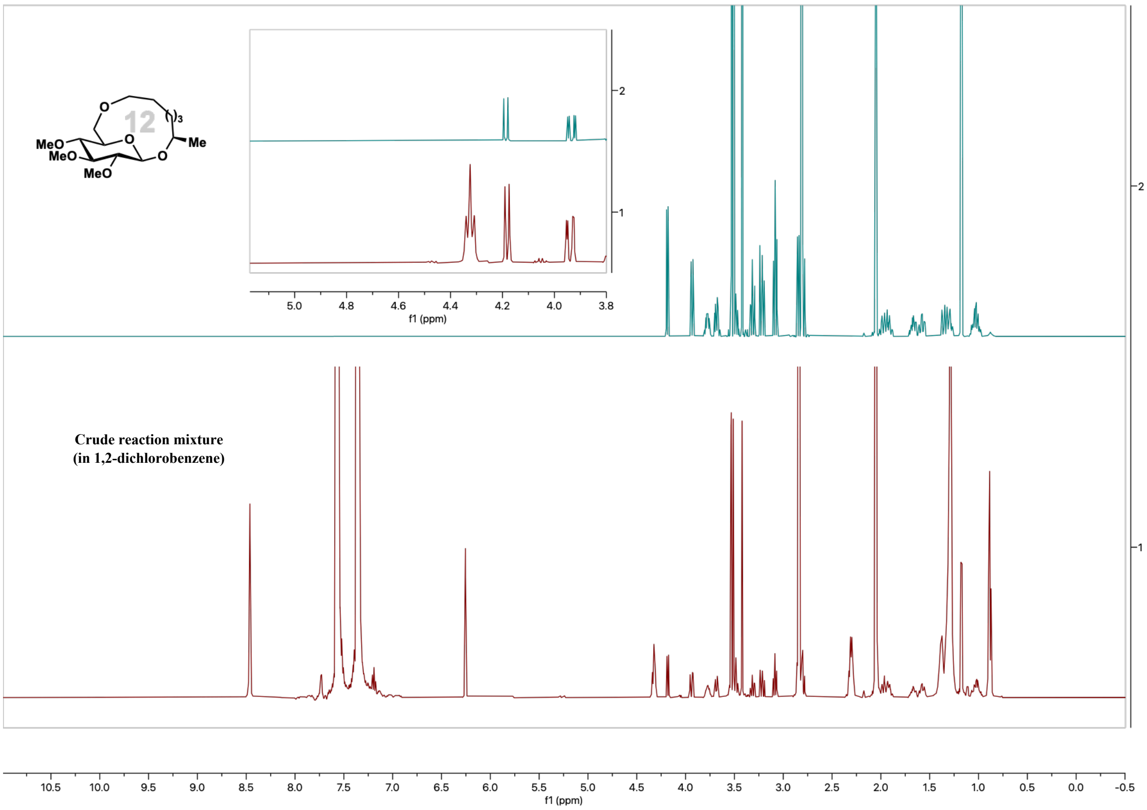

8.9. For compound 2i

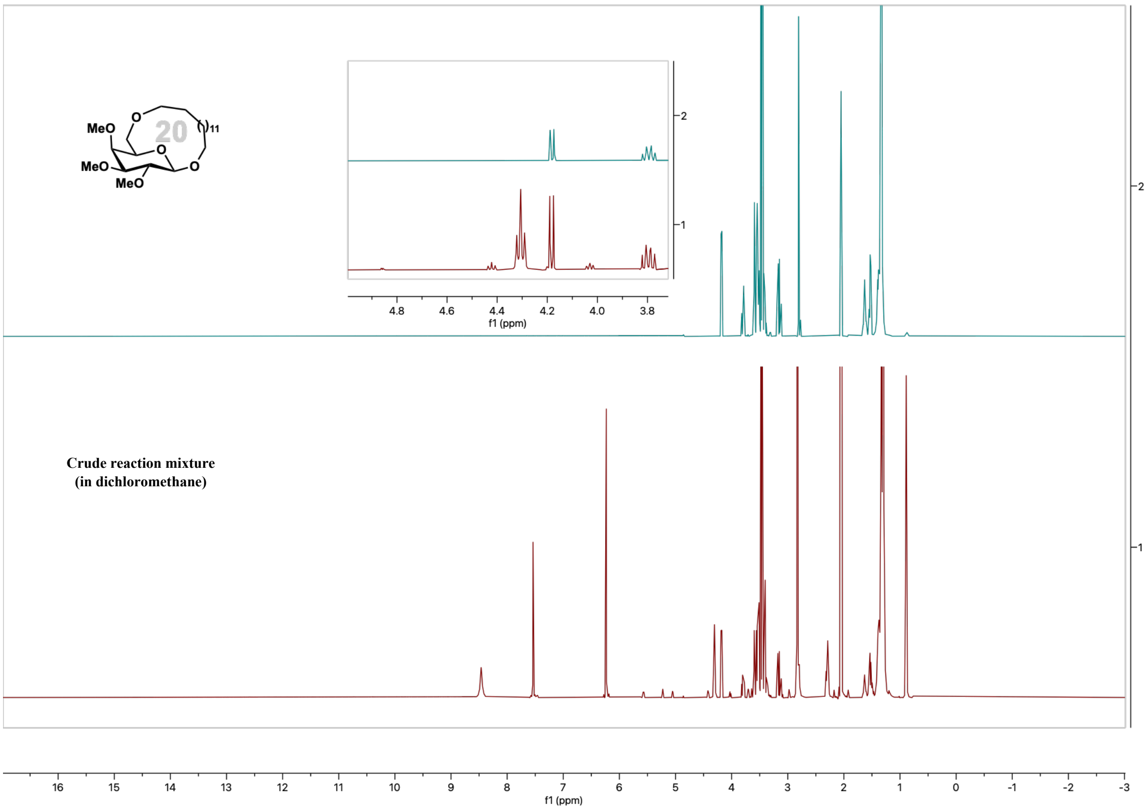

8.10. For compound 2j

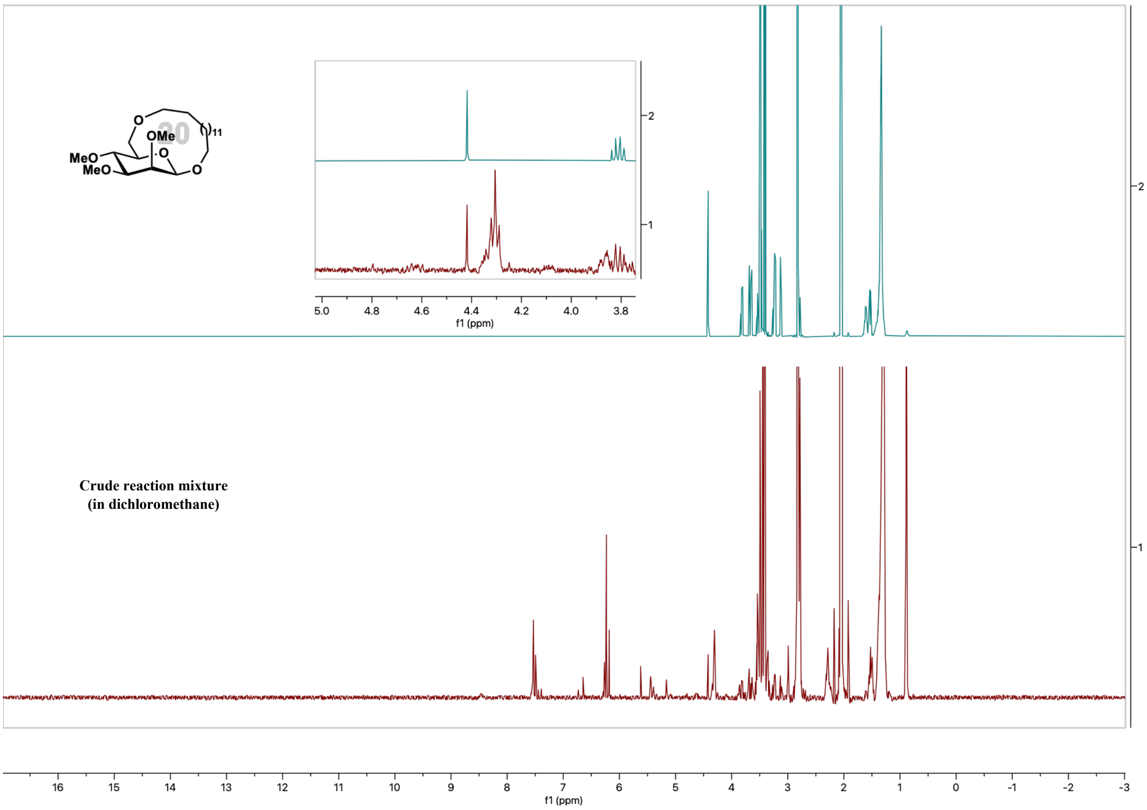

8.11. For compound 4

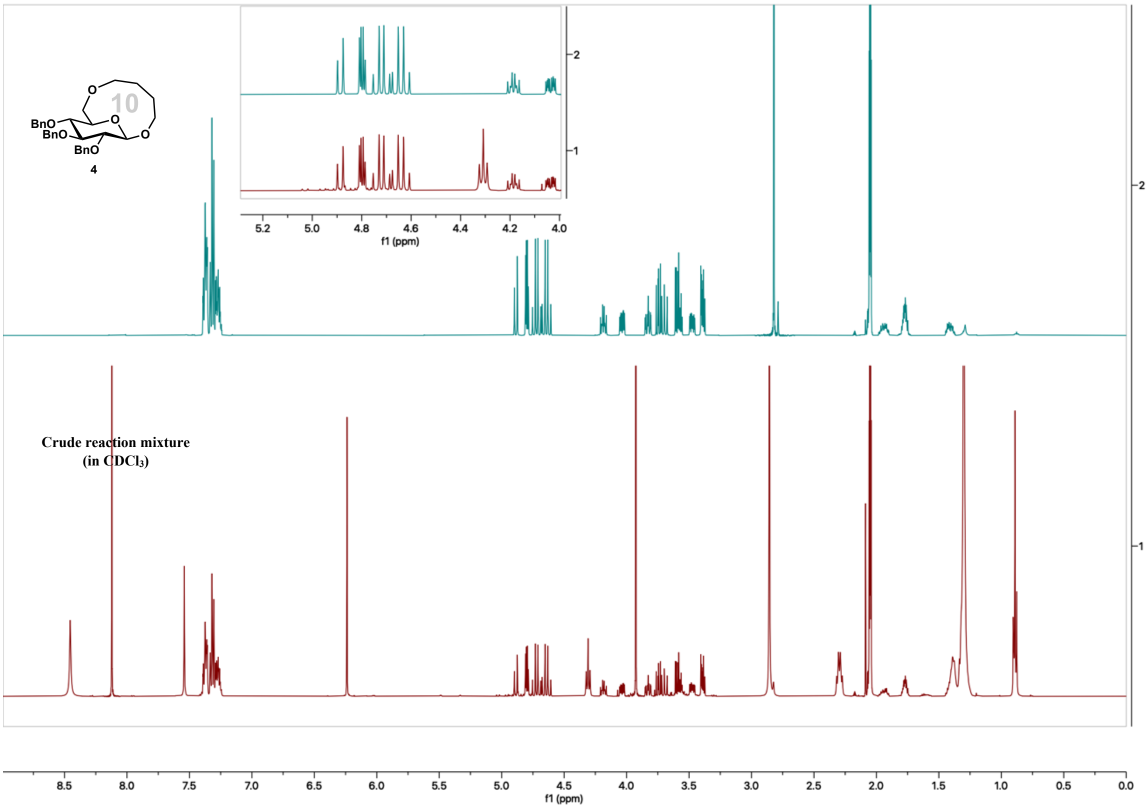

8.12. For compound 5

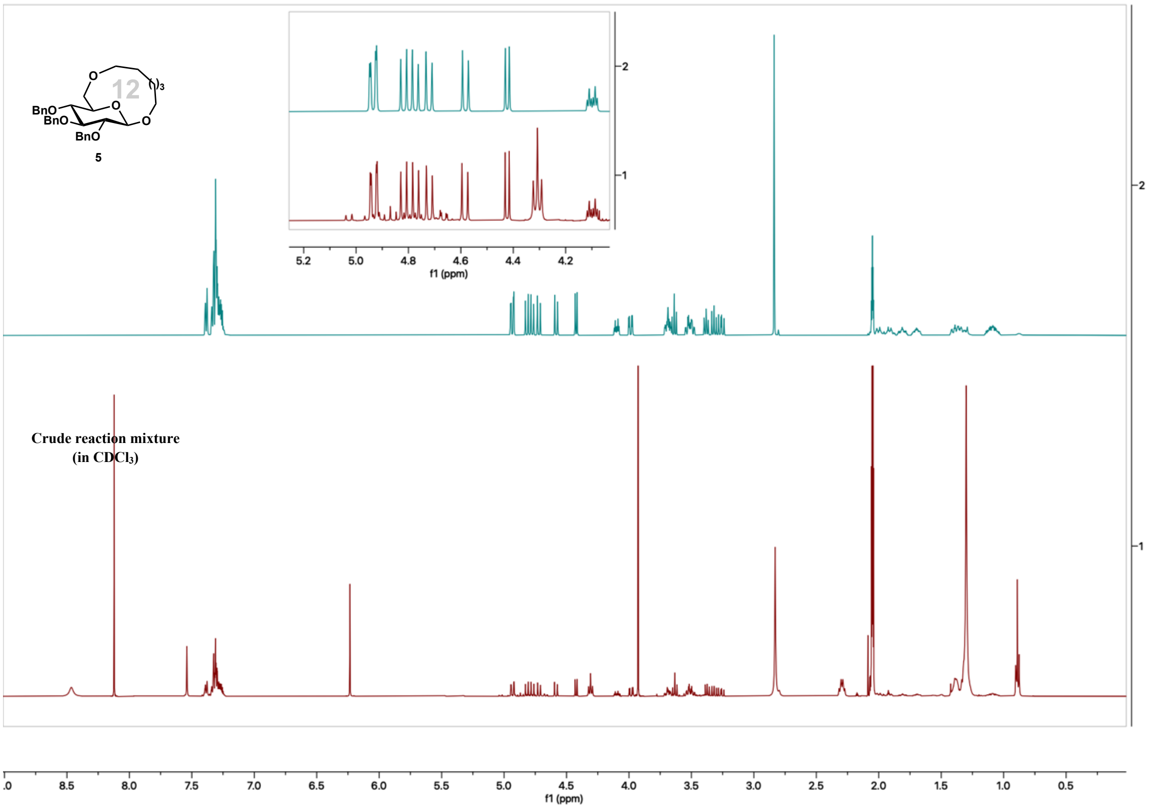

8.13. For compound 6

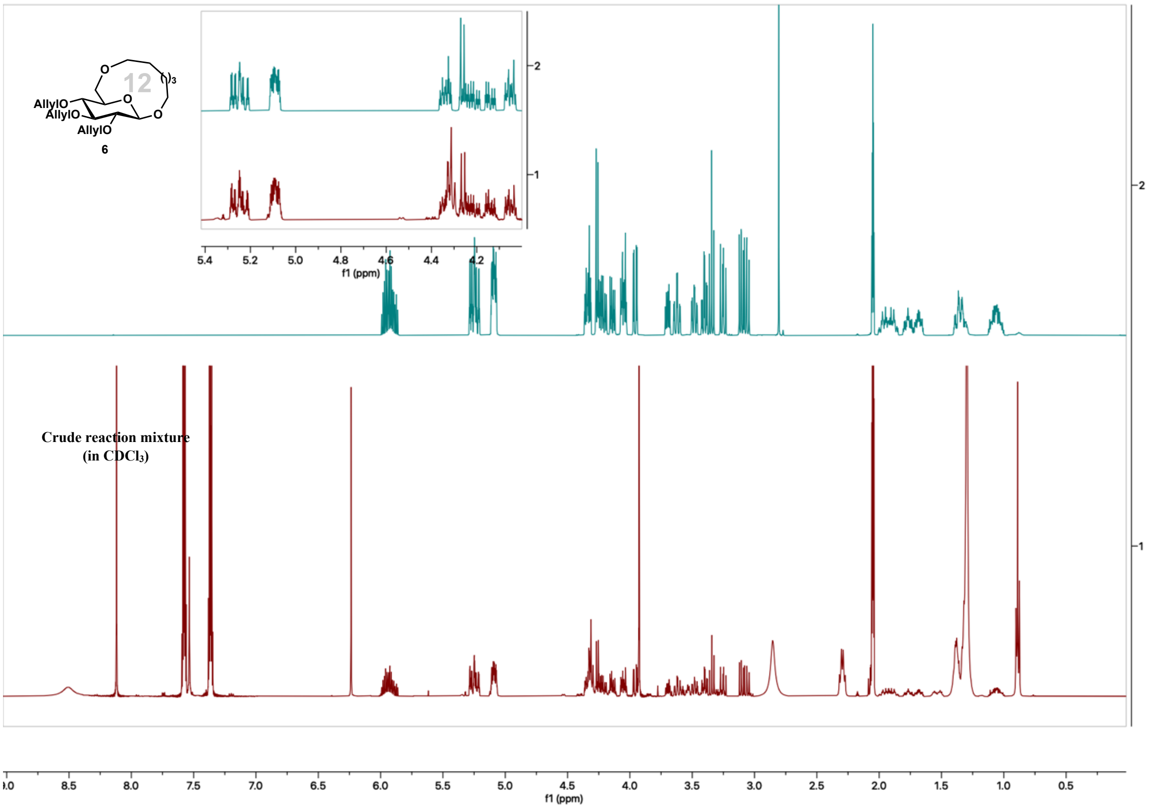

8.14. For compound 7

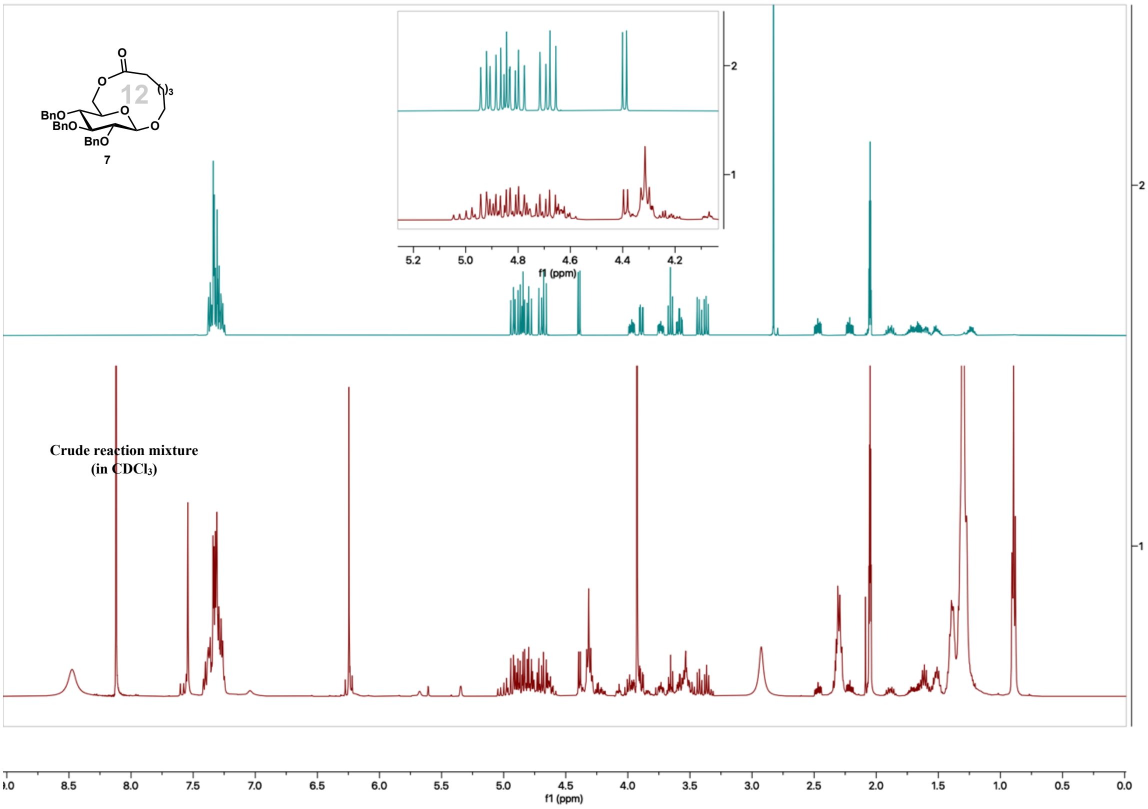

8.15. For compound 8

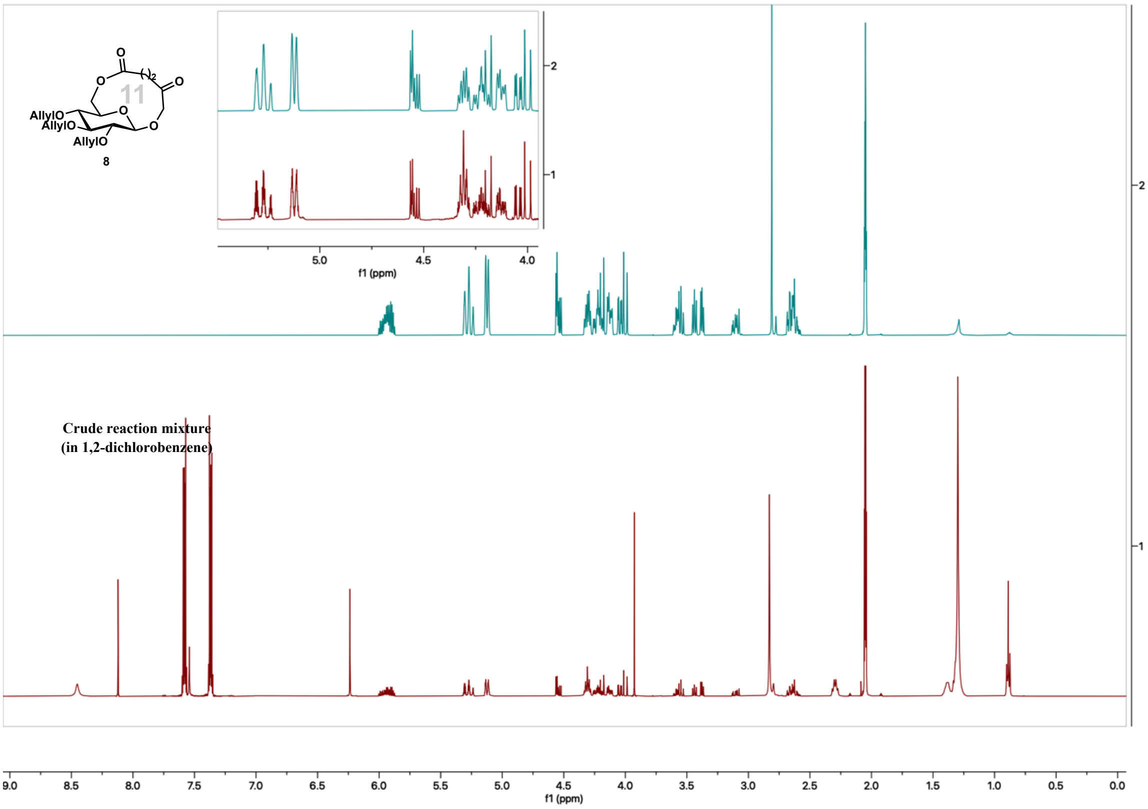

8.16. For compound 10

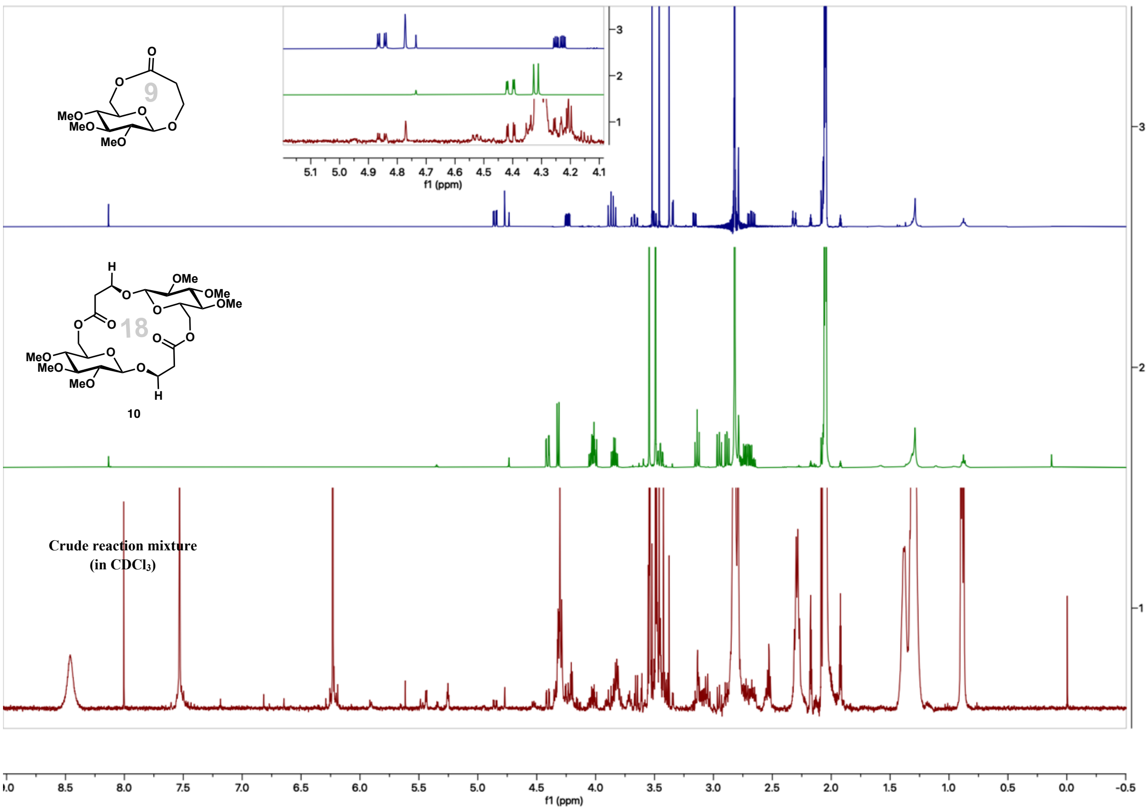

9. Products isolated

(1*R*,9*R*,10*R*,11*S*,12*R*,13*R*,21*R*,22*R*,23*S*,24*R*)-10,11,12,22,23,24-hexamethoxy-2,7,14,19,25,26-hexaoxatricyclo[19.3.1.1^{9,13}]hexacosane (3a)

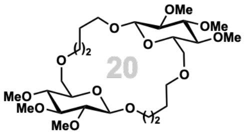

Chemical Formula: $C_{26}H_{48}O_{12}$
Molecular Weight: 552.66

Compound **3a** was synthesized using the **GP1** and substrate **1a** (30.8 mg, 104 µmol, 1.0 equiv.) in $CDCl_3$. The crude material was purified using silica gel column chromatography (0-35% EtOAc in CyH) to yield **3a** (19.7 mg, 35.7 µmol, 69% yield) as a white solid.

R*f*: 0.25 (1:1 cyclohexane/ethyl acetate).

mp: 152 – 157 °C

^{1}H NMR (500 MHz, acetone-*d*$_6$, 298K) δ 4.24 (d, *J* = 7.7 Hz, 2H), 3.81 (dt, *J* = 10.0, 6.5 Hz, 2H), 3.66 (dd, *J* = 11.5, 1.8 Hz, 2H), 3.59 (dt, *J* = 10.0, 6.4 Hz, 2H), 3.56 – 3.51 (m, 12H), 3.50 (s, 6H), 3.46 (s, 6H), 3.29 (ddd, *J* = 9.9, 6.2, 1.8 Hz, 2H), 3.08 (t, *J* = 8.9 Hz, 2H), 2.95 (dd, *J* = 9.9, 8.8 Hz, 2H), 2.84 (dd, *J* = 9.0, 7.8 Hz, 2H), 1.76 – 1.57 (m, 8H).

^{13}C NMR (126 MHz, acetone-*d*$_6$, 298K) δ 104.2, 87.4, 85.0, 80.8, 75.8, 71.4, 70.7, 69.6, 60.7, 60.4, 60.3, 27.3, 26.7.

HRMS (ESI$^+$) *m/z* calculated for $C_{26}H_{48}NaO_{12}$ [M + Na$^+$]: 575.3038; found 575.3048.

IR ν_{max}(thin film)/cm^{-1} 2942w, 2831w, 1466w, 1377m, 1305w, 1188w, 1137m, 1072s, 1038s, 981s, 947s, 630s.

(1*R*,10*R*,11*R*,12*S*,13*R*)-11,12,13-trimethoxy-2,8,14-trioxabicyclo[8.3.1]tetradecane (2b)

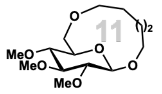

Chemical Formula: $C_{14}H_{26}O_6$
Molecular Weight: 290.36

Compound **2b** was synthesized using the **GP1** and substrate **1b** (29.9 mg, 96.3 µmol, 1.0 equiv.) in 1,2-dichlorobenzene. The crude material was purified using silica gel column chromatography (0-20% EtOAc in CyH) to yield **2b** (8.20 mg, 28.2 µmol, 29% yield) as a white solid.

R*f*: 0.26 (7:3 cyclohexane/ethyl acetate).

mp: 64 – 65 °C

^{1}H NMR (500 MHz, acetone-*d*$_6$, 298K) δ 4.33 (d, *J* = 6.2 Hz, 1H), 3.97 (ddd, *J* = 11.9, 6.6, 1.9 Hz, 1H), 3.75 (ddd, *J* = 10.0, 5.5, 3.2 Hz, 1H), 3.72 (dd, *J* = 12.5, 2.2 Hz, 1H), 3.59 – 3.52 (m, 2H), 3.51 (s, 3H), 3.46 – 3.42 (m, 7H), 3.35 (tt, *J* = 7.5, 2.2 Hz, 1H), 3.17 – 3.08 (m, 2H), 3.05 – 2.96 (m, 1H), 1.72 – 1.50 (m, 5H), 1.38 – 1.30 (m, 1H).

^{13}C NMR (126 MHz, acetone-*d*$_6$, 298K) δ 104.3, 87.3, 84.9, 80.8, 77.1, 72.3, 71.0, 70.8, 60.2, 60.0, 59.4, 30.5, 30.1, 22.5.

HRMS (ESI$^+$) *m/z* calculated for $C_{15}H_{26}NaO_6$ [M + Na$^+$]: 313.1622; found 313.1616.

IR ν_{max}(thin film)/cm^{-1} 2949m, 2914m, 2864m, 2831w, 1445w, 1429w, 1405w, 1372w, 1290w, 1182w, 1125s, 1095s, 1043s, 970s, 605m.

(1*R*,10*R*,11*R*,12*S*,13*R*,14*R*,23*R*,24*R*,25*S*,26*R*)-11,12,13,24,25,26-hexamethoxy-2,8,15,21,27,28-hexaoxatricyclo[21.3.1.1^{10,14}]octacosane (3b)

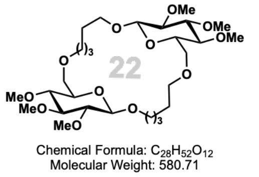

Compound **3b** was synthesized using the **GP1** and substrate **1b** (30.6 mg, 98.6 µmol, 1.0 equiv.) in dichloromethane. The crude material was purified using silica gel column chromatography (0-40% EtOAc in CyH) to yield **3b** (19.9 mg, 34.3 µmol, 70% yield) as a white solid.

R*f*: 0.22 (1:1 cyclohexane/ethyl acetate).

mp: 130 – 132 °C

^{1}H NMR (500 MHz, acetone-*d*$_6$, 298K) δ 4.23 (d, *J* = 7.8 Hz, 2H), 3.79 (dt, *J* = 9.3, 7.2 Hz, 2H), 3.65 (dd, *J* = 10.7, 1.8 Hz, 2H), 3.53 (s, 6H), 3.52 – 3.41 (m, 20H), 3.31 (ddd, *J* = 9.8, 7.3, 1.8 Hz, 2H), 3.09 (t, *J* = 8.9 Hz, 2H), 2.99 – 2.83 (m, 4H), 1.71 – 1.59 (m, 4H), 1.59 – 1.52 (m, 4H), 1.52 – 1.38 (m, 4H).

^{13}C NMR (126 MHz, acetone-*d*$_6$, 298K) δ 103.7, 87.4, 84.9, 81.2, 75.3, 71.5, 70.8, 69.9, 60.7, 60.4, 60.3, 30.3, 30.2, 23.8.

HRMS (ESI$^+$) *m/z* calculated for $C_{28}H_{52}NaO_{12}$ [M + Na$^+$]: 603.3351; found 603.3357.

IR ν_{max}(thin film)/cm^{-1} 2930w, 2859w, 2830w, 1462w, 1374w, 1313w, 1125s, 1070s, 1040s, 994s, 962s, 894w, 730w, 659w.

(1*R*,11*R*,12*R*,13*S*,14*R*)-12,13,14-trimethoxy-2,9,15-trioxabicyclo[9.3.1]pentadecane (2c)

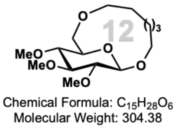

Compound **2c** was synthesized using the **GP1** and substrate **1c** (34.1 mg, 105 µmol, 1.0 equiv.) in 1,2-dichlorobenzene. The crude material was purified using silica gel column chromatography (0-20% EtOAc in CyH) to yield **2c** (22.3 mg, 73.3 µmol, 70% yield) as a white solid.

R*f*: 0.22 (4:1 cyclohexane/ethyl acetate).

mp: 64 – 66 °C

^{1}H NMR (500 MHz, acetone-*d*$_6$, 298K) δ 4.21 (d, *J* = 7.6 Hz, 1H), 4.06 – 3.98 (m, 1H), 3.91 (dd, *J* = 11.9, 2.6 Hz, 1H), 3.68 (ddd, *J* = 9.5, 4.8, 3.6 Hz, 1H), 3.61 (td, *J* = 11.4, 2.6 Hz, 1H), 3.52 (s, 3H), 3.50–3.48 (m, 4H), 3.42 (s, 3H), 3.33 (td, *J* = 9.5, 2.6 Hz, 1H), 3.25 (dd, *J* = 11.9, 9.4 Hz, 1H), 3.09 (t, *J* = 8.6 Hz, 1H), 2.91 – 2.84 (m, 2H), 2.00 – 1.90 (m, 1H), 1.90 – 1.82 (m, 1H), 1.81 – 1.72 (m, 1H), 1.67 (tdt, *J* = 10.3, 6.9, 2.5 Hz, 1H), 1.40 – 1.27 (m, 2H), 1.17 – 1.00 (m, 2H).

^{13}C NMR (126 MHz, acetone-*d*$_6$, 298K) δ 106.0, 87.6, 84.7, 82.5, 76.1, 72.1, 71.4, 70.7, 60.6, 60.3, 60.0, 30.5, 30.3, 22.8, 22.1.

HRMS (ESI$^+$) *m/z* calculated for $C_{15}H_{28}NaO_6$ [M + Na$^+$]: 327.1778; found 327.1775.

IR ν_{max}(thin film)/cm^{-1} 2892m, 2864w, 2830w, 1467w, 1441w, 1373w, 1303w, 1273w, 1156s, 1110s, 1079s, 1059s, 1038s, 1011s, 987s, 934m, 812m, 631m.

(1*R*,11*R*,12*R*,13*S*,14*R*,15*R*,25*R*,26*R*,27*S*,28*R*)-12,13,14,26,27,28-hexamethoxy-2,9,16,23,29,30-hexaoxatricyclo[23.3.1.1^{11,15}]triacontane (3c)

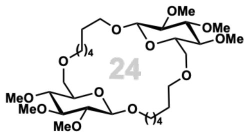

Chemical Formula: $C_{30}H_{56}O_{12}$
Molecular Weight: 608.77

Compound **3c** was synthesized using the **GP1** and substrate **1c** (34.4 mg, 106 µmol, 1.0 equiv.) in dichloromethane. The crude material was purified using silica gel column chromatography (0-40% EtOAc in CyH) to yield **3c** (16.8 mg, 27.6 µmol, 52% yield) as a white solid.

R*f*: 0.28 (1:1 cyclohexane/ethyl acetate).

mp: 140 – 145 °C

^{1}H NMR (500 MHz, acetone-*d$_6$*, 298K) δ 4.23 (d, *J* = 7.7 Hz, 2H), 3.79 (dt, *J* = 9.6, 7.0 Hz, 2H), 3.65 (dd, *J* = 11.4, 1.8 Hz, 2H), 3.57 – 3.48 (m, 20H), 3.46 (s, 6H), 3.28 (ddd, *J* = 9.9, 6.2, 1.8 Hz, 2H), 3.09 (t, *J* = 8.9 Hz, 2H), 2.95 (dd, *J* = 9.9, 8.8 Hz, 2H), 2.85 (dd, *J* = 9.0, 7.8 Hz, 2H), 1.62 (tt, *J* = 7.0, 3.1 Hz, 4H), 1.59 – 1.52 (m, 4H), 1.46 – 1.34 (m, 8H).

^{13}C NMR (126 MHz, acetone-*d$_6$*, 298K) δ 104.0, 87.4, 85.0, 80.8, 75.8, 71.9, 70.6, 69.9, 60.7, 60.4, 60.3, 30.8, 30.5, 26.4, 26.3.

HRMS (ESI$^+$) *m/z* calculated for $C_{30}H_{56}NaO_{12}$ [M + Na$^+$]: 631.3664; found 631.3664.

IR ν_{max}(thin film)/cm^{-1} 2932m, 2832w, 1466w, 1440w, 1376w, 1303w, 1274w, 1186w, 1111s, 1073s, 1034s, 980s, 932m, 730w, 629m.

(1*R*,13*R*,14*R*,15*S*,16*R*)-14,15,16-trimethoxy-2,11,17-trioxabicyclo[11.3.1]heptadecane (2d)

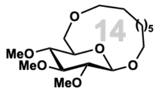

Chemical Formula: $C_{17}H_{32}O_6$
Molecular Weight: 332.44

Compound **2d** was synthesized using the **GP1** and substrate **1d** (37.4 mg, 106 µmol, 1.0 equiv.) in 1,2-dichlorobenzene. The crude material was purified using silica gel column chromatography (0-15% EtOAc in CyH) to yield **2d** (24.4 mg, 73.4 µmol, 69% yield) as a gummy liquid.

R*f*: 0.36 (4:1 cyclohexane/ethyl acetate).

^{1}H NMR (500 MHz, acetone-*d$_6$*, 298K) δ 4.26 (d, *J* = 7.6 Hz, 1H), 3.81 (dt, *J* = 10.9, 6.8 Hz, 1H), 3.67 (dd, *J* = 10.3, 2.0 Hz, 1H), 3.64 – 3.54 (m, 2H), 3.54 (s, 3H), 3.48 (s, 3H), 3.47 – 3.39 (m, 5H), 3.30 (ddd, *J* = 9.3, 7.0, 2.0 Hz, 1H), 3.09 (t, *J* = 8.9 Hz, 1H), 2.92 (dd, *J* = 9.9, 8.8 Hz, 1H), 2.85 (dd, *J* = 9.0, 7.9 Hz, 1H), 1.69 – 1.56 (m, 4H), 1.56 – 1.48 (m, 2H), 1.48 – 1.42 (m, 1H), 1.41 – 1.20 (m, 5H).

^{13}C NMR (126 MHz, acetone-*d$_6$*, 298K) δ 104.9, 87.6, 85.1, 81.2, 74.8, 70.7, 69.9, 69.9, 60.7, 60.4, 60.2, 29.3, 28.3, 26.9, 26.3, 24.5, 23.8.

HRMS (ESI$^+$) *m/z* calculated for $C_{17}H_{32}NaO_6$ [M + Na$^+$]: 355.2091; found 355.2097.

(1*R*,13*R*,14*R*,15*S*,16*R*,17*R*,29*R*,30*R*,31*S*,32*R*)-14,15,16,30,31,32-hexamethoxy-2,11,18,27,33,34-hexaoxatricyclo[27.3.1.1^{13,17}]tetratriacontane (3d)

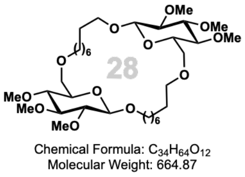

Compound **3d** was synthesized using the **GP1** and substrate **1d** (35.0 mg, 99.3 µmol, 1.0 equiv.) in dichloromethane. The crude material was purified using silica gel column chromatography (0-30% EtOAc in CyH) to yield **3d** (8.00 mg, 12.0 µmol, 24% yield) as a white solid.

R*f*: 0.43 (1:1 cyclohexane/ethyl acetate).

mp: 134 – 136 °C

^{1}H NMR (500 MHz, acetone-*d$_6$*, 298K) δ 4.23 (d, *J* = 7.8 Hz, 2H), 3.80 (dt, *J* = 9.6, 6.9 Hz, 2H), 3.64 (dd, *J* = 11.3, 1.8 Hz, 2H), 3.54 (s, 6H), 3.53 – 3.48 (m, 14H), 3.46 (s, 6H), 3.28 (ddd, *J* = 9.8, 6.1, 1.8 Hz, 2H), 3.09 (t, *J* = 8.9 Hz, 2H), 2.96 (dd, *J* = 9.9, 8.8 Hz, 2H), 2.84 (dd, *J* = 9.0, 7.8 Hz, 2H), 1.62 (p, *J* = 6.9 Hz, 4H), 1.54 (q, *J* = 6.7 Hz, 4H), 1.42 – 1.30 (m, 16H).

^{13}C NMR (126 MHz, acetone-*d$_6$*, 298K) δ 104.0, 87.4, 85.0, 80.8, 75.8, 71.9, 70.6, 70.0, 60.7, 60.4, 60.3, 30.8, 30.6, 30.2, 30.1, 26.9, 26.8.

HRMS (ESI$^+$) *m/z* calculated for $C_{34}H_{64}NaO_{12}$ [M + Na$^+$]: 687.4290; found 687.4302.

IR ν_{max}(thin film)/cm^{-1} 2925m, 2848m, 1466m, 1376m, 1315w, 1272w, 1112s, 1073s, 1042s, 980s, 944m, 933m, 726w, 628m.

(1*R*,15*R*,16*R*,17*S*,18*R*)-16,17,18-trimethoxy-2,13,19-trioxabicyclo[13.3.1]nonadecane (2e)

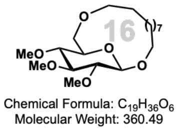

Compound **2e** was synthesized using the **GP1** and substrate **1e** (38.8 mg, 102 µmol, 1.0 equiv.) in 1,2-dichlorobenzene. The crude material was purified by using silica gel column chromatography (0-20% EtOAc in CyH) to yield **2e** (24.4 mg, 67.7 µmol, 66% yield) as a gummy liquid.

R*f*: 0.46 (7:3 cyclohexane/ethyl acetate).

^{1}H NMR (500 MHz, acetone-*d$_6$*, 298K) δ 4.22 (d, *J* = 7.8 Hz, 1H), 3.85 (td, *J* = 9.4, 5.4 Hz, 1H), 3.60 (dd, *J* = 10.1, 2.0 Hz, 1H), 3.57 – 3.53 (m, 4H), 3.52 – 3.43 (m, 9H), 3.27 (ddd, *J* = 9.8, 5.9, 2.0 Hz, 1H), 3.08 (t, *J* = 8.9 Hz, 1H), 3.00 (dd, *J* = 9.8, 8.8 Hz, 1H), 2.84 (dd, *J* = 8.9, 7.8 Hz, 1H), 1.76 – 1.65 (m, 1H), 1.63 – 1.46 (m, 5H), 1.46 – 1.21 (m, 10H).

^{13}C NMR (126 MHz, acetone-*d$_6$*, 298K) δ 103.5, 87.4, 84.9, 80.9, 75.0, 70.6, 70.2, 67.6, 60.8, 60.3, 60.2, 29.6, 28.9, 28.5, 27.2, 27.0, 26.0, 25.4, 25.0.

HRMS (ESI$^+$) *m/z* calculated for $C_{19}H_{36}NaO_6$ [M + Na$^+$]: 383.2404; found 383.2405.

IR ν_{max}(thin film)/cm^{-1} 2923m, 2852m, 1457w, 1371w, 1303w, 1085s, 990m, 936w, 635w.

(1*R*,19*R*,20*R*,21*S*,22*R*)-20,21,22-trimethoxy-2,17,23-trioxabicyclo[17.3.1]tricosane (2f)

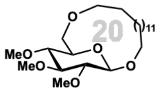

Compound **2f** was synthesized using the **GP1** and substrate **1f** (44.5 mg, 102 µmol, 1.0 equiv.) in dichloromethane. The crude material was purified by using silica gel column chromatography (0-40% EtOAc in CyH) to yield **2f** (35.1 mg, 84.3 µmol, 83% yield) as a gummy liquid.

R*f*: 0.50 (7:3 cyclohexane/ethyl acetate).

^{1}H NMR (500 MHz, acetone-*d$_6$*, 298K) δ 4.23 (d, *J* = 7.8 Hz, 1H), 3.82 (dt, *J* = 9.3, 7.5 Hz, 1H), 3.63 (dd, *J* = 10.8, 1.8 Hz, 1H), 3.54 (s, 3H), 3.52 – 3.44 (m, 10H), 3.28 (ddd, *J* = 9.8, 6.2, 1.8 Hz, 1H), 3.09 (t, *J* = 8.9 Hz, 1H), 2.97 (dd, *J* = 9.9, 8.8 Hz, 1H), 2.84 (dd, *J* = 9.0, 7.8 Hz, 1H), 1.67 – 1.58 (m, 2H), 1.58 – 1.50 (m, 2H), 1.46 – 1.25 (m, 20H).

^{13}C NMR (126 MHz, acetone-*d$_6$*, 298K) δ 103.9, 87.4, 85.0, 80.9, 75.5, 71.8, 70.6, 69.7, 60.8, 60.4, 60.3, 30.3, 30.0, 29.2, 28.6, 28.3, 28.2, 27.9, 27.7, 27.4, 27.3, 26.4, 25.8.

HRMS (ESI$^+$) *m/z* calculated for $C_{23}H_{44}NaO_6$ [M + Na$^+$]: 439.3030; found 439.3033.

IR ν_{max}(thin film)/cm^{-1} 2920m, 2851m, 1458w, 1370w, 1304w, 1084s, 988m, 936w, 722w, 640w.

(1*R*,27*R*,28*R*,29*S*,30*R*)-28,29,30-trimethoxy-2,25,31-trioxabicyclo[25.3.1]hentriacontane (2g)

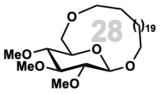

Compound **2g** was synthesized using the **GP1** and substrate **1g** (54.8 mg, 100 µmol, 1.0 equiv.) in dichloromethane. The crude material was purified using silica gel column chromatography (0-15% EtOAc in CyH) to yield **2g** (46.0 mg, 87.0 µmol, 87% yield) as a gummy liquid, which solidified after being kept in the freezer.

R*f*: 0.20 (9:1 cyclohexane/ethyl acetate).

mp: 44 – 46 °C

^{1}H NMR (500 MHz, acetone-*d$_6$*, 298K) δ 4.23 (d, *J* = 7.7 Hz, 1H), 3.82 (dt, *J* = 9.5, 6.7 Hz, 1H), 3.63 (dd, *J* = 11.0, 1.8 Hz, 1H), 3.54 (s, 3H), 3.53 – 3.48 (m, 6H), 3.48 – 3.45 (m, 4H), 3.27 (ddd, *J* = 9.8, 5.6, 1.8 Hz, 1H), 3.09 (t, *J* = 8.9 Hz, 1H), 3.00 (dd, *J* = 9.8, 8.8 Hz, 1H), 2.84 (dd, *J* = 9.0, 7.8 Hz, 1H), 1.68 – 1.58 (m, 2H), 1.54 (dt, *J* = 7.7, 6.3 Hz, 2H), 1.39 (tdd, *J* = 8.8, 5.5, 2.8 Hz, 4H), 1.35 – 1.29 (m, 32H).

^{13}C NMR (126 MHz, acetone-*d$_6$*, 298K) δ 104.0, 87.4, 85.0, 80.7, 75.6, 72.0, 70.5, 70.0, 60.8, 60.4, 60.3, 30.6, 30.0, 29.9, 29.9, 29.8, 29.6, 29.5, 29.4, 29.2, 29.1, 28.9, 28.9, 28.7, 28.7, 28.6, 26.9, 26.7 (some carbon signals are missing due to overlap with other signals).

HRMS (ESI$^+$) *m/z* calculated for $C_{31}H60NaO_6$ [M + Na$^+$]: 551.4282; found 551.4290.

IR ν_{max}(thin film)/cm^{-1} 2912s, 2845s, 1465w, 1375w, 1313w, 1279w, 1152s, 1123s, 1084s, 984m, 935m, 719w, 639w.

(1*R*,3*R*,11*R*,12*R*,13*S*,14*R*)-12,13,14-trimethoxy-3-methyl-2,9,15-trioxabicyclo[9.3.1]pentadecane (2h)

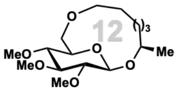

Chemical Formula: $C_{16}H_{30}O_6$
Molecular Weight: 318.41

Compound **2j** was synthesized using the **GP1** and substrate **1h** (17.5 mg, 51.7 µmol, 1.0 equiv.) in 1,2-dichlorobenzene. The crude material was purified using silica gel column chromatography (0-30% EtOAc in CyH) to yield **2h** (12.5 mg, 39.3 µmol, 76% yield) as a white solid.

R*f*: 0.43 (6:4 cyclohexane/ethyl acetate).

mp: 107 – 111 °C

^{1}H NMR (500 MHz, acetone-d_6, 298K) δ 4.19 (d, *J* = 7.8 Hz, 1H), 3.93 (dd, *J* = 12.1, 2.8 Hz, 1H), 3.78 (dqd, *J* = 10.5, 6.2, 2.6 Hz, 1H), 3.72 – 3.65 (m, 1H), 3.53 (s, 3H), 3.51 (s, 3H), 3.51 – 3.46 (m, 1H), 3.42 (s, 3H), 3.31 (td, *J* = 9.8, 2.9 Hz, 1H), 3.22 (dd, *J* = 12.1, 9.9 Hz, 1H), 3.08 (t, *J* = 8.7 Hz, 1H), 2.84 (dd, *J* = 8.8, 7.9 Hz, 1H), 2.80 – 2.78 (m, 1H), 2.04 – 1.86 (m, 2H), 1.67 (dddt, *J* = 14.3, 10.9, 5.1, 3.5 Hz, 1H), 1.58 (dddd, *J* = 14.1, 10.5, 5.0, 3.0 Hz, 1H), 1.44 – 1.25 (m, 2H), 1.18 (d, *J* = 6.3 Hz, 3H), 1.11 – 0.95 (m, 2H).

^{13}C NMR (126 MHz, acetone-d_6, 298K) δ 105.7, 87.7, 84.8, 82.9, 77.8, 76.1, 72.1, 71.4, 60.7, 60.3, 60.2, 37.3, 30.6, 23.3, 22.7, 22.6 (the missing carbon signal is due to overlap with other signals).

HRMS (ESI$^+$) *m/z* calculated for $C_{16}H_{30}NaO_6$ [M + Na$^+$]: 341.1935; found 341.1931.

IR ν_{max}(thin film)/cm^{-1} 2913m, 2868w, 2834w, 1441w, 1372s, 1227w, 1127s, 1113, 1083s, 1044s, 1013s, 637m.

(1*R*,19*R*,20*S*,21*S*,22*R*)-20,21,22-trimethoxy-2,17,23-trioxabicyclo[17.3.1]tricosane (2i)

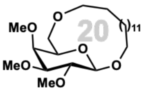

Chemical Formula: $C_{23}H_{44}O_6$
Molecular Weight: 416.60

Compound **2i** was synthesized using the **GP1** and substrate **1i** (43.7 mg, 100 µmol, 1.0 equiv.) in dichloromethane. The crude material was purified using silica gel column chromatography (0-30% EtOAc in CyH) to yield **2i** (24.9 mg, 59.8 µmol, 60% yield) as a gummy liquid.

R*f*: 0.33 (3:2 cyclohexane/ethyl acetate).

^{1}H NMR (500 MHz, acetone-d_6, 298K) δ 4.18 (d, *J* = 7.3 Hz, 1H), 3.88 – 3.74 (m, 1H), 3.63 – 3.57 (m, 2H), 3.57 – 3.49 (m, 4H), 3.48 (s, 3H), 3.46 (s, 3H), 3.45 (s, 3H), 3.43 – 3.38 (m, 1H), 3.21 – 3.10 (m, 2H), 1.68 – 1.58 (m, 2H), 1.53 (p, *J* = 6.6 Hz, 2H), 1.44 – 1.31 (m, 20H).

^{13}C NMR (126 MHz, acetone-d_6, 298K) δ 104.5, 85.1, 81.7, 77.4, 74.9, 71.5, 71.5, 69.7, 61.1, 60.5, 58.5, 29.9, 29.1, 28.5, 28.4, 28.1, 28.1, 27.9, 27.7, 27.3, 26.2, 25.6.

HRMS (ESI$^+$) *m/z* calculated for $C_{23}H_{44}NaO_6$ [M + Na$^+$]: 439.3030; found 439.3037.

IR ν_{max}(thin film)/cm^{-1} 2924s, 2854m, 1460w, 1371m, 1194w, 1000s, 981m, 879w, 764w, 698w.

(1*R*,19*R*,20*R*,21*S*,22*S*)-20,21,22-trimethoxy-2,17,23-trioxabicyclo[17.3.1]tricosane (2j)

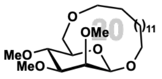

Compound **2j** was synthesized using the **GP1** and substrate **1j** (43.0 mg, 98.5 µmol, 1.0 equiv.) in dichloromethane. The crude material was purified using silica gel column chromatography (0-30% EtOAc in CyH) to yield **2j** (8.50 mg,20.4 µmol, 21% yield) as a white solid.

R*f*: 0.35 (6:4 cyclohexane/ethyl acetate).

mp: 41 – 44 °C

^{1}H NMR (500 MHz, acetone-*d*$_6$, 298K) δ 4.42 (d, *J* = 0.7 Hz, 1H), 3.81 (dt, *J* = 9.3, 7.6 Hz, 1H), 3.69 (dd, *J* = 3.1, 0.8 Hz, 1H), 3.65 (dd, *J* = 10.7, 1.7 Hz, 1H), 3.56 – 3.43 (m, 7H), 3.42 (s, 3H), 3.40 (s, 3H), 3.29 – 3.20 (m, 2H), 3.16 – 3.09 (m, 1H), 1.67 – 1.57 (m, 2H), 1.54 (ddt, *J* = 8.9, 7.0, 5.3 Hz, 2H), 1.45 – 1.27 (m, 20H).

^{13}C NMR (126 MHz, acetone-*d*$_6$, 298K) δ 102.2, 85.0, 77.7, 77.6, 76.5, 71.8, 71.2, 69.7, 60.9, 60.6, 56.8, 30.5, 30.0, 29.2, 28.6, 28.3, 28.1, 27.9, 27.7, 27.3, 27.1, 26.5, 25.8.

HRMS (ESI$^+$) *m/z* calculated for $C_{23}H_{44}NaO_6$ [M + Na$^+$]: 439.3030; found 439.3031.

IR ν_{max}(thin film)/cm^{-1} 2924m, 2856m,. 1459w, 1373w, 1311w, 1260w, 1100s, 1067s, 1004m, 980m, 856w, 773m, 714w, 607w.

(1*R*,9*R*,10*R*,11*S*,12*R*)-10,11,12-tris(benzyloxy)-2,7,13-trioxabicyclo[7.3.1]tridecane (4)

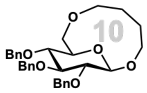

Compound **4** was synthesized using the **GP1** and substrate **S16a** (53.5 mg, 102 µmol, 1.0 equiv.) in $CDCl_3$. The crude material was purified using silica gel column chromatography (0-20% EtOAc in CyH) to yield **4** (38.8 mg,76.9 µmol, 75% yield) as a white solid.

R*f*: 0.50 (7:3 cyclohexane/ethyl acetate).

mp: 66 – 70 °C

^{1}H NMR (500 MHz, acetone-*d*$_6$, 298K) δ 7.46 – 7.34 (m, 6H), 7.34 – 7.24 (m, 9H), 4.89 (d, *J* = 11.3 Hz, 1H), 4.84 – 4.78 (m, 2H), 4.78 – 4.68 (m, 2H), 4.64 (q, *J* = 11.7 Hz, 2H), 4.24 – 4.15 (m, 1H), 4.04 (ddd, *J* = 11.4, 4.5, 2.8 Hz, 1H), 3.83 (ddd, *J* = 11.2, 8.8, 2.5 Hz, 1H), 3.78 – 3.66 (m, 2H), 3.63 – 3.54 (m, 2H), 3.48 (ddd, *J* = 11.1, 6.6, 2.6 Hz, 1H), 3.43 – 3.36 (m, 2H), 1.99 – 1.89 (m, 1H), 1.77 (tdd, *J* = 6.4, 4.6, 3.2 Hz, 2H), 1.46 – 1.35 (m, 1H).

^{13}C NMR (126 MHz, acetone-*d*$_6$, 298K) δ 139.3, 138.8, 138.7, 128.2, 128.1, 128.0, 127.8, 127.7, 127.7, 127.4, 127.3, 127.2, 102.2, 81.9, 81.1, 79.8, 77.4, 74.3, 72.4, 71.8, 71.2, 70.3, 65.0, 27.9, 24.8.

HRMS (ESI$^+$) *m/z* calculated for $C_{31}H_{36}NaO_6$ [M + Na$^+$]: 527.2404; found 527.2394.

IR ν_{max}(thin film)/cm^{-1} 3023w, 2907m, 2857m, 1603w, 1494w, 1450m, 1396w, 1353m, 1307w, 1206w, 1135m, 1095s, 1061s, 1035s, 974ss, 832w, 730s, 692s, 672m.

(1*R*,11*R*,12*R*,13*S*,14*R*)-12,13,14-tris(benzyloxy)-2,9,15-trioxabicyclo[9.3.1]pentadecane (5)

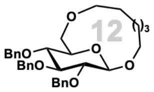

Compound **5** was synthesized using the **GP1** and substrate **S16b** (56.4 mg, 102 µmol, 1.0 equiv.) in $CDCl_3$. The crude material was purified using silica gel column chromatography (0-15% EtOAc in CyH) to yield **5** (36.9 mg, 69.3 µmol, 68% yield) as a white solid.

R*f*: 0.57 (7:3 cyclohexane/ethyl acetate).

mp: 114 – 116 °C

^{1}H NMR (500 MHz, acetone-*d6*, 298K) δ 7.43 – 7.36 (m, 2H), 7.36 – 7.30 (m, 8H), 7.29 – 7.22 (m, 5H), 4.93 (dd, *J* = 11.3, 2.2 Hz, 2H), 4.82 (d, *J* = 11.2 Hz, 1H), 4.77 (d, *J* = 11.2 Hz, 1H), 4.72 (d, *J* = 11.4 Hz, 1H), 4.58 (d, *J* = 11.2 Hz, 1H), 4.42 (d, *J* = 7.6 Hz, 1H), 4.10 (dt, *J* = 11.3, 3.8 Hz, 1H), 3.99 (dd, *J* = 12.1, 2.8 Hz, 1H), 3.74 – 3.66 (m, 2H), 3.64 (t, *J* = 8.7 Hz, 1H), 3.51 (dtd, *J* = 14.5, 9.7, 2.7 Hz, 2H), 3.38 (dd, *J* = 8.7, 7.6 Hz, 1H), 3.35 – 3.29 (m, 1H), 3.26 (dd, *J* = 12.1, 9.5 Hz, 1H), 2.03 – 1.95 (m, 1H), 1.95 – 1.86 (m, 1H), 1.81 (dddd, *J* = 14.8, 11.9, 8.1, 4.1 Hz, 1H), 1.70 (tdd, *J* = 13.8, 6.2, 3.6 Hz, 1H), 1.45 – 1.30 (m, 2H), 1.18 – 1.01 (m, 2H).

^{13}C NMR (126 MHz, acetone-*d6*, 298K) δ 140.1, 140.1, 139.6, 129.1, 129.0, 129.0, 128.7, 128.7, 128.5, 128.4, 128.2, 128.1, 106.3, 85.8, 83.2, 80.7, 76.2, 75.7, 75.2, 74.6, 72.2, 71.4, 70.9, 30.5, 30.3, 22.7, 22.1.

HRMS (ESI^+) *m/z* calculated for $C_{33}H_{40}NaO_6$ [M + Na^+]: 555.2717; found 555.2714.

IR ν_{max}(thin film)/cm^{-1} 3025w, 2892w, 2860w, 1495w, 1451w, 1350m, 1301w, 1275w, 1211w, 1172w, 1141m, 1111s, 1080s, 1059s, 1040s, 1028s, 1011s, 950m, 933w, 908w, 815w, 729s, 693s.

(1R,11R,12R,13S,14R)-12,13,14-tris(allyloxy)-2,9,15-trioxabicyclo[9.3.1]pentadecane (6)

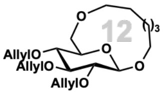

Compound **6** was synthesized using the **GP1** and substrate **S25** (41.9 mg, 104 µmol, 1.0 equiv.) in 1,2-dichlorobenzene. The crude material was purified by using silica gel column chromatography (0-15% EtOAc in CyH) to yield **6** (31.0 mg, 81.1 µmol, 78% yield) as a white solid.

R*f*: 0.60 (7:3 cyclohexane/ethyl acetate).

mp: 85 – 87 °C

^{1}H NMR (500 MHz, acetone-*d6*, 298K) δ 6.08 – 5.73 (m, 3H), 5.28 (dq, *J* = 7.8, 1.8 Hz, 1H), 5.26 – 5.20 (m, 2H), 5.14 – 5.04 (m, 3H), 4.34 (dtt, *J* = 13.4, 5.1, 1.6 Hz, 2H), 4.29 – 4.23 (m, 2H), 4.21 (ddt, *J* = 12.8, 5.6, 1.6 Hz, 1H), 4.14 (ddt, *J* = 13.0, 5.6, 1.6 Hz, 1H), 4.10 – 4.01 (m, 2H), 3.96 (dd, *J* = 12.1, 2.8 Hz, 1H), 3.70 (ddd, *J* = 9.5, 4.6, 3.6 Hz, 1H), 3.62 (td, *J* = 11.5, 2.5 Hz, 1H), 3.48 (ddd, *J* = 10.5, 9.5, 2.6 Hz, 1H), 3.40 (td, *J* = 9.7, 2.8 Hz, 1H), 3.34 (t, *J* = 8.8 Hz, 1H), 3.25 (dd, *J* = 12.1, 9.5 Hz, 1H), 3.11 (dd, *J* = 8.8, 7.7 Hz, 1H), 3.06 (dd, *J* = 9.8, 8.7 Hz, 1H), 2.01 – 1.84 (m, 2H), 1.83 – 1.73 (m, 1H),

1.69 (dddt, J = 14.1, 10.5, 5.4, 3.5 Hz, 1H), 1.42 – 1.26 (m, 2H), 1.06 (tddd, J = 21.7, 8.9, 7.1, 4.2 Hz, 2H).

^{13}C NMR (126 MHz, acetone-d_6, 298K) δ 136.9, 136.8, 136.4, 116.5, 115.9, 115.9, 106.2, 85.5, 82.7, 80.4, 76.1, 74.6, 74.0, 73.5, 72.2, 71.4, 70.9, 30.5, 22.7, 22.0 (one carbon signal is missing due to the overlap with solvent signal).

Note: DEPT 135 spectra have been attached as evidence of the missing carbon overlapped with the solvent signal.

HRMS (ESI$^+$) *m/z* calculated for $C_{21}H_{34}NaO_6$ [M + Na$^+$]: 405.2248; found 405.2254.

IR ν_{max}(thin film)/cm^{-1} 2903m, 2848m, 1646w, 1465w, 1425w, 1346w, 1277w, 1240w, 1063s, 1039s, 1016s, 999s, 921s, 811w, 683w, 624w.

(1*R*,11*R*,12*R*,13*S*,14*R*)-12,13,14-tris(benzyloxy)-2,9,15-trioxabicyclo[9.3.1]pentadecan-8-one (7)

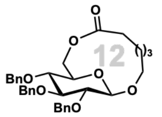

Chemical Formula: $C_{33}H_{38}O_7$
Molecular Weight: 546.66

Compound **7** was synthesized using the **GP1** and substrate **S18** (57.2 mg, 101 µmol, 1.0 equiv.) in $CDCl_3$ at 50 °C for 7 d. The crude material was purified by using silica gel column chromatography (0-20% EtOAc in CyH) to yield **7** (33.0 mg,60.4 µmol, 60% yield) as a white solid.

R*f*: 0.60 (7:3 cyclohexane/ethyl acetate).

mp: 113 – 116 °C

^{1}H NMR (500 MHz, acetone-d_6, 298K) δ 7.42 – 7.15 (m, 15H), 4.91 (dd, J = 17.7, 11.2 Hz, 2H), 4.88 – 4.77 (m, 3H), 4.69 (dd, J = 19.0, 11.3 Hz, 2H), 4.39 (d, J = 7.7 Hz, 1H), 3.97 (ddd, J = 11.6, 7.4, 3.3 Hz, 1H), 3.89 (dd, J = 11.2, 3.4 Hz, 1H), 3.73 (ddd, J = 11.7, 7.7, 3.0 Hz, 1H), 3.66 (t, J = 8.7 Hz, 1H), 3.59 (td, J = 10.0, 3.4 Hz, 1H), 3.42 (dd, J = 9.8, 8.7 Hz, 1H), 3.37 (dd, J = 8.7, 7.8 Hz, 1H), 2.47 (ddd, J = 13.0, 8.6, 4.1 Hz, 1H), 2.21 (ddd, J = 13.2, 8.3, 4.5 Hz, 1H), 1.89 (ddt, J = 16.2, 13.3, 7.9 Hz, 1H), 1.78 – 1.56 (m, 3H), 1.56 – 1.47 (m, 1H), 1.28 – 1.18 (m, 1H).

^{13}C NMR (126 MHz, acetone-d_6, 298K) δ 173.4, 140.0, 140.0, 139.5, 129.1, 129.0, 129.0, 128.9, 128.7, 128.6, 128.5, 128.2, 105.7, 85.8, 83.1, 80.4, 75.8, 75.2, 74.7, 73.3, 71.9, 63.5, 34.3, 29.5, 24.4, 23.2 (missing carbon signal is due to the overlap with other carbon signal).

HRMS (ESI$^+$) *m/z* calculated for $C_{33}H_{38}NaO_7$ [M + Na$^+$]: 569.2510; found 569.2507.

IR ν_{max}(thin film)/cm^{-1} 3025w, 2858w, 1742s, 1494w, 1451m, 1351w, 1314w, 1298w, 1257w, 1232w, 1208m, 1141s, 1109s, 1065s, 1023s, 1001s, 906w, 742s, 705s, 660m.

(1*R*,10*R*,11*R*,12*S*,13*R*)-11,12,13-tris(allyloxy)-2,8,14-trioxabicyclo[8.3.1]tetradecane-4,7-dione (8)

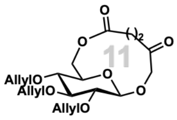

Chemical Formula: $C_{20}H_{28}O_8$
Molecular Weight: 396.44

Compound **9** was synthesized using the **GP1** and substrate **S27** (41.5 mg, 99.6 µmol, 1.0 equiv.) in 1,2-dichlorobenzene at 50 °C for 7 d. The crude material was purified by using silica gel column chromatography (0-30% EtOAc in CyH) to yield **9** (28.0 mg,70.6 µmol, 71% yield).

R*f*: 0.37 (3:2 cyclohexane/ethyl acetate).

^{1}H NMR (500 MHz, acetone-d_6, 298K) δ 6.25 – 5.77 (m, 3H), 5.44 – 5.20 (m, 3H), 5.12 (dtd, *J* = 10.1, 1.9, 0.9 Hz, 3H), 4.58 – 4.50 (m, 2H), 4.31 (dtt, *J* = 12.9, 5.6, 1.5 Hz, 2H), 4.27 – 4.17 (m, 3H), 4.16 – 4.10 (m, 2H), 4.08 – 4.03 (m, 1H), 4.00 (d, *J* = 14.1 Hz, 1H), 3.63 – 3.52 (m, 2H), 3.44 (dd, *J* = 8.2, 6.6 Hz, 1H), 3.38 (ddd, *J* = 6.1, 5.1, 0.9 Hz, 1H), 3.15 – 3.06 (m, 1H), 2.71 – 2.57 (m, 3H).

^{13}C NMR (126 MHz, acetone-d_6, 298K) δ 206.8, 172.3, 136.5, 136.3, 136.2, 116.9, 116.6, 116.3, 103.4, 84.9, 82.4, 77.6, 75.3, 74.0, 73.7, 73.6, 72.5, 63.3, 36.5, 31.2.

HRMS (ESI^{+}) *m/z* calculated for $C_{20}H_{28}NaO_8$ [M + Na^{+}]: 419.1676; found 419.1674.

IR ν_{max}(thin film)/cm^{-1} 2910w, 1741s, 1644w, 1423w, 1342w, 1247m, 1133s, 1073s, 1017m, 995m, 924m, 605w.

10. Synthesis of cyclization substrates

10.1. Synthesis of ether-linked glucose derivatives for the cyclization

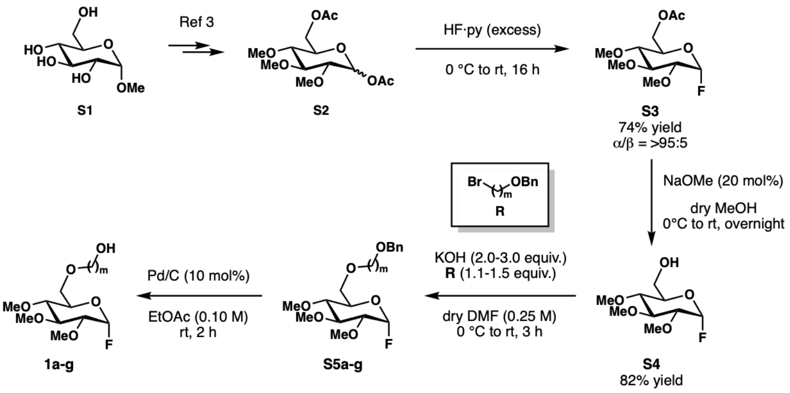

((2*R*,3*R*,4*S*,5*R*,6*R*)-6-fluoro-3,4,5-trimethoxytetrahydro-2*H*-pyran-2-yl)methyl acetate (S3)

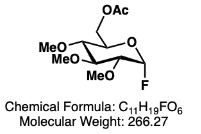

Compound **S3** was synthesized according to the modified procedure of the Miller group.[2] To a 100 mL plastic vial was added compound **S2**[3] (8.15 g, 26.6 mmol, 1.0 equiv.) and a magnetic stir bar. The vial was then placed in an ice-water bath, and a cold (freshly removed from a –20 °C freezer) solution of 70% HF•pyridine (36 mL) was added to the solid compound via a plastic syringe. The vial was capped, and the suspension was stirred for 14 h. Over the course of the reaction, the ice bath was allowed to warm to rt. The reaction mixture was then diluted with ice-cold H_2O (25 mL) followed by CH_2Cl_2 (25 mL), and the resulting biphasic mixture was stirred for 5 min. The mixture was transferred to a 500 mL separatory funnel, rinsed with CH_2Cl_2, and the layers were separated. The organic layer was collected, and the aqueous layer was extracted with CH_2Cl_2 (3x 40 mL). The combined organic layers were then washed with a saturated aqueous $NaHCO_3$ solution (1x 50 mL) followed by brine solution (1x 50 mL), dried over anhydrous $MgSO_4$, filtered, and evaporated to dryness in a 500 mL round-bottom flask. The resulting crude material was purified via silica gel column chromatography (10-40% EtOAc in CyH) to obtain the title compound **S3** (α:β>95:5) as colourless gummy liquid (5.26 g, 19.8 mmol, 74% yield).

R*f*: 0.40 (1:1 cyclohexane/ethyl acetate).

^{1}H NMR (500 MHz, $CDCl_3$, 298K) δ 5.65 (dd, *J* = 53.0, 2.7 Hz, 1H), 4.33 (dd, *J* = 12.1, 2.1 Hz, 1H), 4.26 (dd, *J* = 12.1, 4.7 Hz, 1H), 3.90 (ddd, *J* = 10.2, 4.8, 2.1 Hz, 1H), 3.65 (s, 3H), 3.55 (s, 3H), 3.55 – 3.49 (m, 4H), 3.24 – 3.10 (m, 2H), 2.10 (s, 3H).

^{19}F NMR (470 MHz, $CDCl_3$, 298K) δ -149.4.

^{13}C NMR (126 MHz, $CDCl_3$, 298K) δ 170.8, 104.9 (d, *J* = 227.1 Hz), 83.0, 81.5 (d, *J* = 24.7 Hz), 78.9, 71.3 (d, *J* = 4.4 Hz), 62.8, 61.2, 60.9, 59.4, 21.0.

HRMS (ESI^+) *m/z* calculated for $C_{11}H_{19}FNaO_6$ [M + Na^+]: 289.1058; found 289.1061.

IR ν_{max}(thin film)/cm^{-1} 2933w, 2829w, 1738s, 1445w, 1367m, 1231s, 1185m, 1157s, 1099s, 1021s, 997s, 942m, 889m, 756m, 687w, 633w, 603w.

((2*R*,3*R*,4*S*,5*R*,6*R*)-6-fluoro-3,4,5-trimethoxytetrahydro-2*H*-pyran-2-yl)methanol (S4)

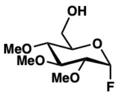

A 100 mL two-neck round bottom flask was charged with α-D-glucopyranosyl fluoride **S3** (4.90 g, 18.4 mmol, 1.0 equiv.) and dry MeOH (55 mL). The solution was stirred and cooled to 0 °C. After 10 minutes, solid NaOMe (0.20 g, 3.68 mmol, 0.20 equiv.) was added in one portion and kept at 0 °C for 10 minutes. Subsequently, the reaction mixture was allowed to warm up to rt and stirred for 3 h. After completion of the reaction, the solvent was removed under reduced pressure and worked up using an ethyl acetate/water mixture. The combined organic layer was dried over anhydrous $MgSO_4$ and concentrated under reduced pressure. The crude material was purified by silica gel column chromatography (10-60% EtOAc in CyH) to yield the corresponding alcohol **S4** (3.38 g, 15.1 mmol, 82% yield) as a colourless gummy liquid that solidified upon freezing.

R_f: 0.25 (2:1 cyclohexane: EtOAc).

mp: 40 – 42 °C

^{1}H NMR (500 MHz, $CDCl_3$, 298K) δ 5.66 (dd, J = 53.2, 2.7 Hz, 1H), 3.90 – 3.82 (m, 1H), 3.80 – 3.72 (m, 2H), 3.65 (s, 3H), 3.58 (s, 3H), 3.56 (s, 3H), 3.52 (t, J = 9.3 Hz, 1H), 3.26 (ddd, J = 9.9, 9.0, 0.7 Hz, 1H), 3.17 (ddd, J = 25.6, 9.5, 2.7 Hz, 1H), 1.75 (dd, J = 8.1, 4.7 Hz, 1H).

^{19}F NMR (470 MHz, $CDCl_3$, 298K) δ -148.41.

^{13}C NMR (126 MHz, $CDCl_3$, 298K) δ 104.9 (d, J = 226.8 Hz), 82.8, 81.4 (d, J = 24.6 Hz), 78.4, 73.3 (d, J = 3.5 Hz), 61.3, 61.0, 60.8, 59.3.

HRMS (ESI^+) *m/z* calculated for $C_9H_{17}FNaO_5$ [M + Na^+]: 247.0952; found 247.0953.

IR ν_{max}(thin film)/cm^{-1} 3459br, 2931m, 2832w, 2356w, 2147w, 1455w, 1380w, 1186m, 1159s, 1103s, 1016s, 890w, 757w, 698w.

General procedure for the synthesis of compound S5a-g (GP2)

A 50 mL two-neck dry round bottom flask was charged with compound **S4** (1.0 equiv.) and dissolved in dry DMF (0.25 M). The solution was cooled to 0 °C using an ice bath, and finely ground KOH (2.0-3.0 equiv.) was added. The reaction mixture was stirred at the same temperature for 45 minutes, and a solution of the corresponding bromide **R** (1.1-1.5 equiv.) in dry DMF (1 mL) was added dropwise. After 15 minutes, the ice bath was removed, and the reaction was stirred for 3 h at room temperature. After completion of the reaction (monitored by TLC), it was stopped by the addition of water, and the mixture was extracted with EtOAc (4x 15 mL). The combined organic phases were washed with water (4x 20 mL), and brine solution (1x 20 mL), dried over anhydrous $MgSO_4$, and removed under reduced pressure. The crude material was purified by flash column chromatography (5-15% EtOAc in CyH) to yield **S5a-g** as a colourless oil.

General procedure for the synthesis of compound 1a-g (GP3)

A 50 mL one-neck round-bottom flask was charged with **S5a-g** (1.0 equiv.) and dissolved in EtOAc (0.10 M). Palladium on carbon (5% or 10% Pd, 0.10 equiv.) was added, and the solution was purged with H_2 for 30 minutes. After the reaction was completed (monitored by TLC), the reaction mixture was filtered through a celite bed, and the filtrate was concentrated under reduced pressure. The crude

mixture was purified by flash column chromatography (20-70% EtOAc in CyH) to yield compound **1a-g**.

(2*R*,3*R*,4*S*,5*R*,6*R*)-2-((4-(benzyloxy)butoxy)methyl)-6-fluoro-3,4,5-trimethoxytetrahydro-2*H*-pyran (S5a)

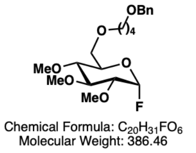

Compound **S5a** was synthesized according to the **GP2** using compound **S4** (336 mg, 1.50 mmol, 1.0 equiv.), KOH (252 mg, 4.50 mmol, 3.0 equiv.) and the corresponding bromide (802 mg, 3.30 mmol, 2.2 equiv.) in 6 mL dry DMF. The crude material was purified by silica gel column chromatography (0-25% EtOAc in CyH) to yield the product **S5a** (500 mg, 1.29 mmol, 86% yield) as a colourless liquid.

^{1}H NMR (500 MHz, $CDCl_3$, 298K) δ 7.37 – 7.31 (m, 4H), 7.30 – 7.26 (m, 1H), 5.66 (dd, *J* = 53.4, 2.7 Hz, 1H), 4.50 (s, 2H), 3.89 – 3.72 (m, 1H), 3.69 – 3.60 (m, 5H), 3.58 – 3.54 (m, 7H), 3.52 – 3.42 (m, 4H), 3.31 (dd, *J* = 10.1, 9.1 Hz, 1H), 3.20 (ddd, *J* = 25.7, 9.6, 2.7 Hz, 1H), 1.76 – 1.64 (m, 4H).

^{19}F NMR (470 MHz, $CDCl_3$, 298K) δ -149.42.

^{13}C NMR (126 MHz, $CDCl_3$, 298K) δ 138.7, 128.5, 127.7, 127.7, 105.2 (d, *J* = 226.2 Hz), 83.1, 81.5 (d, *J* = 24.8 Hz), 78.5, 73.0, 72.8 (d, *J* = 4.1 Hz), 71.6, 70.2, 68.7, 61.1, 60.8, 59.3, 26.6, 26.5.

HRMS (ESI^{+}) *m/z* calculated for $C_{20}H_{31}FNaO_6$ [M + Na^{+}]: 409.1997; found 409.2004.

4-(((2*R*,3*R*,4*S*,5*R*,6*R*)-6-fluoro-3,4,5-trimethoxytetrahydro-2*H*-pyran-2-yl)methoxy)butan-1-ol (1a)

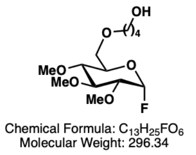

Compound **1a** was synthesized according to the **GP3** using compound **S5a** (475 mg, 1.23 mmol, 1.0 equiv.) using palladium on carbon (5%, 262 mg, 123 μmol, 0.10 equiv.) The crude material was purified by silica gel column chromatography (10-70% EtOAc in CyH) to yield the product **1a** (336 mg, 1.13 mmol, 92% yield) as a colourless gummy liquid.

R*f* 0.18 (3:7 cyclohexane: EtOAc).

^{1}H NMR (500 MHz, $CDCl_3$, 298K) δ 5.67 (dd, *J* = 53.2, 2.7 Hz, 1H), 3.78 (dddd, *J* = 10.2, 3.2, 2.2, 0.6 Hz, 1H), 3.74 – 3.62 (m, 7H), 3.59 – 3.54 (m, 7H), 3.53 – 3.46 (m, 2H), 3.29 (ddd, *J* = 10.1, 9.1, 0.7 Hz, 1H), 3.20 (ddd, *J* = 25.8, 9.6, 2.7 Hz, 1H), 2.08 (s, 1H), 1.83 – 1.62 (m, 4H).

^{19}F NMR (470 MHz, $CDCl_3$, 298K) δ -149.43.

^{13}C NMR (126 MHz, $CDCl_3$, 298K) δ 105.1 (d, *J* = 226.4 Hz), 83.1, 81.5 (d, *J* = 24.8 Hz), 78.5, 72.6 (d, *J* = 4.1 Hz), 71.7, 68.9, 62.9, 61.1, 60.8, 59.4, 30.3, 26.8.

HRMS (ESI^{+}) *m/z* calculated for $C_{13}H_{25}FNaO_6$ [M + Na^{+}]: 319.1527; found 319.1533.

IR ν_{max}(thin film)/cm^{-1} 3442br, 2929m, 1444w, 1377w, 1158s, 1099s, 1051s, 1021s, 995s, 890m, 850w, 757m, 690w.

(2*R*,3*R*,4*S*,5*R*,6*R*)-2-(((5-(benzyloxy)pentyl)oxy)methyl)-6-fluoro-3,4,5-trimethoxytetrahydro-2*H*-pyran (S5b)

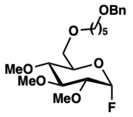

Chemical Formula: $C_{21}H_{33}FO_6$
Molecular Weight: 400.49

Compound **S5b** was synthesized according to the **GP2** using compound **S4** (336 mg, 1.50 mmol, 1.0 equiv.), KOH (252 mg, 4.50 mmol, 3.0 equiv.) and corresponding bromide (849 mg, 3.30 mmol, 2.2 equiv.) in 6 mL dry DMF. The crude material was purified by silica gel column chromatography (0-25% EtOAc in CyH) to deliver the product **S5b** (532 mg, 1.33 mmol, 89% yield) as a colourless liquid.

^{1}H NMR (500 MHz, $CDCl_3$, 298K) δ 7.37 – 7.31 (m, 4H), 7.28 (ddd, *J* = 6.9, 3.4, 2.1 Hz, 1H), 5.67 (dd, *J* = 53.4, 2.7 Hz, 1H), 4.50 (s, 2H), 3.88 – 3.73 (m, 1H), 3.68 – 3.60 (m, 5H), 3.58 – 3.50 (m, 7H), 3.50 – 3.41 (m, 4H), 3.31 (dd, *J* = 10.1, 9.1 Hz, 1H), 3.20 (ddd, *J* = 25.8, 9.6, 2.7 Hz, 1H), 1.71 – 1.56 (m, 4H), 1.52 – 1.36 (m, 2H).

^{19}F NMR (470 MHz, $CDCl_3$, 298K) δ -149.44.

^{13}C NMR (126 MHz, $CDCl_3$, 298K) δ 138.8, 128.5, 127.8, 127.6, 105.2 (d, *J* = 226.1 Hz), 83.1, 81.5 (d, *J* = 24.8 Hz), 78.5, 73.0, 72.8 (d, *J* = 4.1 Hz), 71.7, 70.4, 68.7, 61.1, 60.7, 59.3, 29.7, 29.5, 23.0.

HRMS (ESI^+) *m/z* calculated for $C_{21}H_{33}FNaO_6$ [M + Na^+]: 423.2153; found 423.2160.

5-(((2*R*,3*R*,4*S*,5*R*,6*R*)-6-fluoro-3,4,5-trimethoxytetrahydro-2*H*-pyran-2-yl)methoxy)pentan-1-ol (1b)

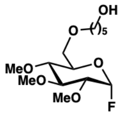

Chemical Formula: $C_{14}H_{27}FO_6$
Molecular Weight: 310.36

Compound **1b** was synthesized according to the **GP3** using compound **S5b** (505 mg, 1.26 mmol, 1.0 equiv.) using palladium on carbon (5%, 268 mg, 126 μmol, 0.10 equiv.) The crude material was purified by silica gel column chromatography (10-70% EtOAc in CyH) to yield the product **1b** (368 mg, 1.19 mmol, 94% yield) as a colourless gummy liquid.

R*f* 0.20 (4:6 cyclohexane: EtOAc).

^{1}H NMR (500 MHz, $CDCl_3$, 298K) δ 5.67 (dd, *J* = 53.3, 2.7 Hz, 1H), 3.83 – 3.73 (m, 1H), 3.68 – 3.60 (m, 7H), 3.58 – 3.52 (m, 7H), 3.52 – 3.41 (m, 2H), 3.30 (dd, *J* = 10.1, 9.1 Hz, 1H), 3.20 (ddd, *J* = 25.7, 9.6, 2.7 Hz, 1H), 1.72 – 1.53 (m, 4H), 1.52 – 1.35 (m, 2H), 1.33 (s, 1H).

^{19}F NMR (470 MHz, $CDCl_3$, 298K) δ -149.45.

^{13}C NMR (126 MHz, $CDCl_3$, 298K) δ 105.0 (d, *J* = 226.3 Hz), 83.0, 81.3 (d, *J* = 24.8 Hz), 78.4, 72.6 (d, *J* = 4.1 Hz), 71.6, 68.6, 62.8, 61.0, 60.6, 59.2, 32.5, 29.3, 22.3.

HRMS (ESI^+) *m/z* calculated for $C_{14}H_{27}FNaO_6$ [M + Na^+]: 333.1684; found 333.1689.

IR ν_{max}(thin film)/cm^{-1} 3444br, 2929m, 1454w, 1378w, 1158s, 1099s, 1020s, 995s, 937w, 890m, 850w, 758m, 690w.

(2*R*,3*R*,4*S*,5*R*,6*R*)-2-(((6-(benzyloxy)hexyl)oxy)methyl)-6-fluoro-3,4,5-trimethoxytetrahydro-2*H*-pyran (S5c)

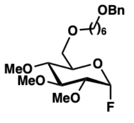

Chemical Formula: $C_{22}H_{35}FO_6$
Molecular Weight: 414.51

Compound **S5c** was synthesized according to the **GP2** using compound **S4** (224 mg, 1.00 mmol, 1.0 equiv.), KOH (168 mg, 3.00 mmol, 3.0 equiv.) and corresponding bromide (407 mg, 1.50 mmol, 1.5 equiv.) in 4 mL dry DMF. The crude material was purified by silica gel column chromatography (0-20% EtOAc in CyH) to yield the product **S5c** (403 mg, 0.972 mmol, 97% yield) as a colourless liquid.

$\mathbf{R}_f$ 0.22 (4:1 cyclohexane: EtOAc).

^{1}H NMR (500 MHz, $CDCl_3$, 298K) δ 7.37 – 7.32 (m, 4H), 7.30 – 7.26 (m, 1H), 5.67 (dd, *J* = 53.2, 2.7 Hz, 1H), 4.50 (s, 2H), 3.80 – 3.74 (m, 1H), 3.68 – 3.58 (m, 5H), 3.55-351 (m, 7H), 3.51 – 3.44 (m, 3H), 3.41 (dt, *J* = 9.5, 6.9 Hz, 1H), 3.31 (ddd, *J* = 10.1, 9.2, 0.7 Hz, 1H), 3.20 (ddd, *J* = 25.7, 9.6, 2.7 Hz, 1H), 1.67 – 1.58 (m, 4H), 1.44 – 1.31 (m, 4H).

^{19}F NMR (470 MHz, $CDCl_3$, 298K) δ -149.44.

^{13}C NMR (126 MHz, $CDCl_3$, 298K) δ 138.8, 128.5, 127.8, 127.6, 105.2 (d, *J* = 226.2 Hz), 83.1, 81.5 (d, *J* = 24.8 Hz), 78.5, 73.0, 72.8 (d, *J* = 4.1 Hz), 71.8, 70.5, 68.7, 61.1, 60.7, 59.3, 29.9, 29.7, 26.2, 26.2.

HRMS (ESI$^+$) *m/z* calculated for $C_{22}H_{35}FNaO_6$ [M + Na$^+$]: 437.2310; found 437.2313.

IR ν_{max}(thin film)/cm^{-1} 2928m, 2853m, 1452w, 1362w, 1159s, 1099s, 1022s, 890m, 850w, 735m, 696m, 609w.

(((2*R*,3*R*,4*S*,5*R*,6*R*)-6-fluoro-3,4,5-trimethoxytetrahydro-2*H*-pyran-2-yl)methoxy)methanol (1c)

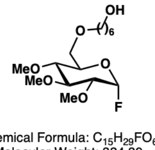

Chemical Formula: $C_{15}H_{29}FO_6$
Molecular Weight: 324.39

Compound **1c** was synthesized according to the **GP3** using compound **S5c** (387 mg, 0.934 mmol, 1.0 equiv.) using palladium on carbon (5%, 199 mg, 93.4 μmol, 0.10 equiv.) The crude material was purified by silica gel column chromatography (10-70% EtOAc in CyH) to yield the product **1c** (277 mg, 0.854 mmol, 91% yield) as a colourless gummy liquid.

$\mathbf{R}_f$ 0.28 (3:7 cyclohexane: EtOAc)

^{1}H NMR (500 MHz, $CDCl_3$, 298K) δ 5.67 (dd, *J* = 53.3, 2.7 Hz, 1H), 3.80 – 3.74 (m, 1H), 3.66 – 3.60 (m, 7H), 3.57 – 3.46 (m, 8H), 3.42 (dtd, *J* = 9.3, 6.8, 2.1 Hz, 1H), 3.35 – 3.27 (m, 1H), 3.20 (ddt, *J* = 25.7, 9.6, 2.5 Hz, 1H), 1.68 – 1.52 (m, 4H), 1.45 – 1.31 (m, 4H).

^{19}F NMR (470 MHz, $CDCl_3$, 298K) δ -149.47.

^{13}C NMR (126 MHz, $CDCl_3$, 298K) δ 105.0 (d, *J* = 226.2 Hz), 83.0, 81.3 (d, *J* = 24.8 Hz), 78.4, 72.6 (d, *J* = 4.1 Hz), 71.6, 68.6, 62.9, 61.0, 60.6, 59.2, 32.7, 29.5, 26.0, 25.6.

HRMS (ESI^+) *m/z* calculated for $C_{15}H_{29}FNaO_6$ [M + Na^+]: 347.1840; found 347.1843.

IR ν_{max}(thin film)/cm^{-1} 3446br, 2927m, 2856m, 1455w, 1378w, 1158s, 1100s, 1053s, 1020s, 938w, 889m, 850w, 757m, 690w.

(2*R*,3*R*,4*S*,5*R*,6*R*)-2-(((8-(benzyloxy)octyl)oxy)methyl)-6-fluoro-3,4,5-trimethoxytetrahydro-2*H*-pyran (S5d)

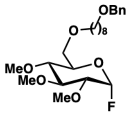

Chemical Formula: $C_{24}H_{39}FO_6$
Molecular Weight: 442.57

Compound **S5d** was synthesized according to the **GP2** using compound **S4** (336 mg, 1.50 mmol, 1.0 equiv.), KOH (252 mg, 4.50 mmol, 3.0 equiv.) and corresponding bromide[4] (673 mg, 2.25 mmol, 1.5 equiv.) in 6 mL dry DMF. The crude material was purified by silica gel column chromatography (0-20% EtOAc in CyH) to yield the product **S5d** (602 mg, 1.36 mmol, 91% yield) as a colourless liquid.

^{1}H NMR (500 MHz, $CDCl_3$, 298K) δ 7.38 – 7.32 (m, 4H), 7.31 – 7.27 (m, 1H), 5.67 (dd, *J* = 53.3, 2.7 Hz, 1H), 4.50 (s, 2H), 3.82 – 3.73 (m, 1H), 3.65 (s, 3H), 3.64 – 3.60 (m, 2H), 3.55 (s, 3H), 3.54 (s, 3H), 3.54 – 3.50 (m, 1H), 3.50 – 3.44 (m, 3H), 3.41 (dt, *J* = 9.4, 6.9 Hz, 1H), 3.32 (dd, *J* = 10.1, 9.2 Hz, 1H), 3.21 (ddd, *J* = 25.8, 9.6, 2.7 Hz, 1H), 1.60 (tdd, *J* = 8.8, 6.7, 4.4 Hz, 4H), 1.41 – 1.26 (m, 8H).

^{19}F NMR (470 MHz, $CDCl_3$, 298K) δ -149.45.

^{13}C NMR (126 MHz, $CDCl_3$, 298K) δ 138.9, 128.5, 127.8, 127.6, 105.2 (d, *J* = 226.2 Hz), 83.1, 81.5 (d, *J* = 24.9 Hz), 78.5, 73.0, 72.8 (d, *J* = 4.1 Hz), 71.9, 70.6, 68.7, 61.2, 60.8, 59.3, 29.9, 29.7, 29.6, 29.6, 26.3, 26.2.

HRMS (ESI^+) *m/z* calculated for $C_{24}H_{39}FNaO_6$ [M + Na^+]: 465.2623; found 465.2630.

8-(((2*R*,3*R*,4*S*,5*R*,6*R*)-6-fluoro-3,4,5-trimethoxytetrahydro-2*H*-pyran-2-yl)methoxy)octan-1-ol (1d)

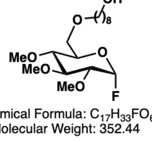

Chemical Formula: $C_{17}H_{33}FO_6$
Molecular Weight: 352.44

Compound **1d** was synthesized according to the **GP3** using compound **S5d** (582 mg, 1.31 mmol, 1.0 equiv.) using palladium on carbon (5%, 279 mg, 131 μmol, 0.10 equiv.) The crude material was purified by silica gel column chromatography (10-70% EtOAc in CyH) to yield the product **1d** (395 mg, 1.12 mmol, 85% yield) as a colourless gummy liquid.

R_f: 0.38 (3:7 cyclohexane/ethyl acetate).

^{1}H NMR (500 MHz, $CDCl_3$, 298K) δ 5.67 (dd, *J* = 53.3, 2.7 Hz, 1H), 3.83 – 3.74 (m, 1H), 3.69 – 3.59 (m, 7H), 3.55 (s, 3H), 3.54 (s, 3H), 3.54 – 3.47 (m, 2H), 3.41 (dt, *J* = 9.4, 6.9 Hz, 1H), 3.32 (ddd, *J* = 10.1, 9.1, 0.7 Hz, 1H), 3.21 (ddd, *J* = 25.7, 9.6, 2.7 Hz, 1H), 1.65 – 1.51 (m, 4H), 1.41 – 1.28 (m, 9H).

^{19}F NMR (470 MHz, $CDCl_3$, 298K) δ -149.47.

^{13}C NMR (126 MHz, $CDCl_3$, 298K) δ 105.2 (d, *J* = 226.2 Hz), 83.1, 81.5 (d, *J* = 24.8 Hz), 78.5, 72.8 (d, *J* = 4.1 Hz), 71.9, 68.7, 63.2, 61.2, 60.7, 59.3, 32.9, 29.7, 29.5, 29.5, 26.2, 25.8.

HRMS (ESI^+) *m/z* calculated for $C_{17}H_{33}FNaO_6$ [M + Na^+]: 375.2153; found 375.2156.

IR ν_{max}(thin film)/cm^{-1} 3414br, 2924m, 2851m, 1458w, 1378w, 1158s, 1100s, 1054s, 1021s, 890m, 850w, 758m, 690m.

(2*R*,3*R*,4*S*,5*R*,6*R*)-2-(((10-(benzyloxy)decyl)oxy)methyl)-6-fluoro-3,4,5-trimethoxytetrahydro-2*H*-pyran (S5e)

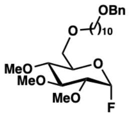

Chemical Formula: $C_{26}H_{43}FO_6$
Molecular Weight: 470.62

Compound **S5e** was synthesized according to the **GP2** using compound **S4** (565 mg, 2.52 mmol, 1.0 equiv.), KOH (424 mg, 7.56 mmol, 3.0 equiv.) and the corresponding bromide[5] (1.65 g, 5.04 mmol, 2.0 equiv.) in 10 mL dry DMF. The crude material was purified by silica gel column chromatography (0-15% EtOAc in CyH) to yield the product **S5e** (1.03 g, 2.19 mmol, 87% yield) as a colourless liquid.

R_f 0.50 (7:3 cyclohexane: EtOAc).

^{1}H NMR (500 MHz, $CDCl_3$, 298K) δ 7.36-7.32 (d, *J* = 4.4 Hz, 4H), 7.30 – 7.26 (m, 1H), 5.68 (dd, *J* = 53.4, 2.7 Hz, 1H), 4.50 (s, 2H), 3.81 – 3.75 (m, 1H), 3.68 – 3.59 (m, 5H), 3.56 (s, 3H), 3.54 (s, 3H), 3.54 – 3.50 (m, 1H), 3.50 – 3.44 (m, 3H), 3.41 (dt, *J* = 9.5, 7.0 Hz, 1H), 3.32 (ddd, *J* = 10.0, 9.1, 0.7 Hz, 1H), 3.21 (ddd, *J* = 25.8, 9.6, 2.7 Hz, 1H), 1.65 – 1.55 (m, 4H), 1.42 – 1.23 (m, 12H).

^{19}F NMR (470 MHz, $CDCl_3$, 298K) δ -149.44.

^{13}C NMR (126 MHz, $CDCl_3$, 298K) δ 138.9, 128.5, 127.8, 127.6, 105.2 (d, *J* = 226.3 Hz), 83.1, 81.5 (d, *J* = 24.9 Hz), 78.5, 73.0, 72.8, 71.9, 70.7, 68.7, 61.2, 60.8, 59.3, 29.9, 29.7, 29.7, 29.7, 29.6, 29.6, 26.3, 26.3.

HRMS (ESI^+) *m/z* calculated for $C_{26}H_{43}FNaO_6$ [M + Na^+]: 493.2936; found 493.2931.

IR ν_{max}(thin film)/cm^{-1} 2922m, 1849m, 1452w, 1361w, 1159s, 1100s, 1023s, 891m, 850w, 734m, 696m, 608w.

10-(((2*R*,3*R*,4*S*,5*R*,6*R*)-6-fluoro-3,4,5-trimethoxytetrahydro-2*H*-pyran-2-yl)methoxy)decan-1-ol (1e)

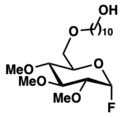

Chemical Formula: $C_{19}H_{37}FO_6$
Molecular Weight: 380.50

Compound **1e** was synthesized according to the **GP3** from compound **S5e** (800 mg, 1.70 mmol, 1.0 equiv.) using palladium on carbon (10%, 181 mg, 170 µmol, 0.10 equiv.) The crude material was purified by silica gel column chromatography (20-70% EtOAc in CyH) to yield the product **1e** (600 mg, 1.58 mmol, 93% yield) as a colourless gummy liquid.

R_f 0.25 (3:2 cyclohexane: EtOAc)

^{1}H NMR (500 MHz, $CDCl_3$, 298K) δ 5.68 (dd, *J* = 53.3, 2.6 Hz, 1H), 3.77 (dddd, *J* = 10.1, 3.1, 2.2, 0.6 Hz, 1H), 3.67 – 3.60 (m, 7H), 3.56 (s, 3H), 3.55 (s, 3H), 3.54 – 3.47 (m, 2H), 3.41 (dt, *J* = 9.4, 6.9 Hz, 1H), 3.32 (ddd, *J* = 10.0, 9.1, 0.7 Hz, 1H), 3.21 (ddd, *J* = 25.7, 9.6, 2.7 Hz, 1H), 1.67 – 1.52 (m, 4H), 1.40 – 1.26 (m, 12H), 1.26 – 1.21 (m, 1H).

^{19}F NMR (470 MHz, $CDCl_3$, 298K) δ -149.45.

^{13}C NMR (126 MHz, $CDCl_3$, 298K) δ 105.0 (d, *J* = 226.2 Hz), 83.0, 81.3 (d, *J* = 24.8 Hz), 78.4, 72.6 (d, *J* = 4.1 Hz), 71.8, 68.5, 63.1, 61.0, 60.6, 59.2, 32.8, 29.6, 29.5, 29.5, 29.4, 29.4, 26.1, 25.7.

HRMS (ESI^+) *m/z* calculated for $C_{19}H_{37}FNaO_6$ [M + Na^+]: 403.2466; found 403.2469.

IR ν_{max}(thin film)/cm^{-1} 3444br, 2921m, 2849m, 1460w, 1378w, 1158s, 1101s, 1054s, 1021s, 891m, 850w, 758m, 690w.

(2*R*,3*R*,4*S*,5*R*,6*R*)-2-(((14-(benzyloxy)tetradecyl)oxy)methyl)-6-fluoro-3,4,5-trimethoxytetrahydro-2*H*-pyran (S5f)

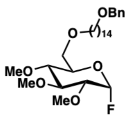

Chemical Formula: $C_{30}H_{51}FO_6$
Molecular Weight: 526.73

Compound **S5f** was synthesized according to the **GP2** using compound **S4** (275 mg, 1.23 mmol, 1.0 equiv.), KOH (166 mg, 2.95 mmol, 2.4 equiv.) and the corresponding bromide[4] (709 mg, 1.85 mmol, 1.5 equiv.) in 5 mL dry DMF. The crude material was purified by silica gel column chromatography (0-10% EtOAc in CyH) to yield the product **S5f** (575 mg, 1.10 mmol, 90% yield) as a colourless liquid.

R_f 0.10 (19:1 cyclohexane: EtOAc).

^{1}H NMR (500 MHz, $CDCl_3$, 298K) δ 7.36 – 7.32 (m, 4H), 7.28 (dd, *J* = 4.9, 3.8 Hz, 1H), 5.68 (dd, *J* = 53.3, 2.7 Hz, 1H), 4.50 (s, 2H), 3.77 (dt, *J* = 10.1, 2.8 Hz, 1H), 3.68 – 3.59 (m, 5H), 3.56 (s, 3H), 3.55 (s, 3H), 3.54 – 3.50 (m, 1H), 3.50 – 3.45 (m, 3H), 3.41 (dt, *J* = 9.4, 6.9 Hz, 1H), 3.33 (t, *J* = 9.6 Hz, 1H), 3.21 (ddd, *J* = 25.7, 9.5, 2.7 Hz, 1H), 1.65 – 1.55 (m, 4H), 1.39 – 1.32 (m, 5.6 Hz, 4H), 1.26 – 1.25 (m, 16H).

^{19}F NMR (470 MHz, $CDCl_3$, 298K) δ -149.44.

^{13}C NMR (126 MHz, $CDCl_3$, 298K) δ 138.9, 128.5, 127.8, 127.6, 105.2 (d, *J* = 226.2 Hz), 83.1, 81.5 (d, *J* = 24.8 Hz), 78.5, 73.0, 72.8 (d, *J* = 4.1 Hz), 71.9, 70.7, 68.6, 61.1, 60.7, 59.3, 29.9, 29.8, 29.8, 29.8, 29.8, 29.7, 29.6, 29.6, 26.3, 26.3 (two carbons is missing due to overlap with other signals).

HRMS (ESI^+) *m/z* calculated for $C_{30}H_{51}FNaO_6$ [M + Na^+]: 549.3562; found 549.3568.

IR ν_{max}(thin film)/cm^{-1} 2919s, 2848s, 1452m, 1361m, 1159s, 1101s, 1023s, 891m, 851w, 733s, 696s.

14-(((2*R*,3*R*,4*S*,5*R*,6*R*)-6-fluoro-3,4,5-trimethoxytetrahydro-2*H*-pyran-2-yl)methoxy)tetradecan-1-ol (1f)

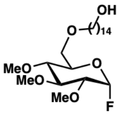

Compound **1f** was synthesized according to the **GP3** using compound **S5f** (575 mg, 1.10 mmol, 1.0 equiv.) using palladium on carbon (10%, 117 mg, 110 µmol, 0.10 equiv.) The crude material was purified by silica gel column chromatography (20-50% EtOAc in CyH) to yield the product **1f** (408 mg, 0.93 mmol, 85% yield) as a white solid.

$\mathbf{R}_f$ 0.30 (3:2 cyclohexane: EtOAc).

mp: 48 – 50 °C

^{1}**H NMR** (500 MHz, $CDCl_3$, 298K) δ 5.67 (dd, *J* = 53.3, 2.7 Hz, 1H), 3.81 – 3.74 (m, 1H), 3.68 – 3.58 (m, 7H), 3.55 (s, 3H), 3.54 (s, 3H), 3.53 – 3.46 (m, 2H), 3.41 (dt, *J* = 9.4, 6.9 Hz, 1H), 3.36 – 3.29 (m, 1H), 3.21 (ddd, *J* = 25.8, 9.5, 2.7 Hz, 1H), 1.66 – 1.51 (m, 4H), 1.39 – 1.22 (m, 20H).

^{19}**F NMR** (470 MHz, $CDCl_3$, 298K) δ -149.45.

^{13}**C NMR** (126 MHz, $CDCl_3$, 298K) δ 105.2 (d, *J* = 226.2 Hz), 83.1, 81.5 (d, *J* = 24.8 Hz), 78.5, 72.8 (d, *J* = 4.1 Hz), 71.9, 68.6, 63.2, 61.1, 60.7, 59.3, 33.0, 29.8, 29.8, 29.7, 29.7, 29.7, 29.7, 29.6, 29.6, 26.3, 25.9 (one carbon is missing due to overlap with other signals).

HRMS (ESI^+) *m/z* calculated for $C_{23}H_{45}FNaO_6$ [M + Na^+]: 459.3092; found 459.3098.

IR ν_{max}(thin film)/cm^{-1} 3546m, 2910s, 2843s, 1460w, 1381w, 1366w, 1336w, 1164s, 1132s, 1101s, 1073s, 1012m, 997m, 969m, 936m, 880m, 852w, 752m, 612w.

10.2. Synthesis of compound 1g

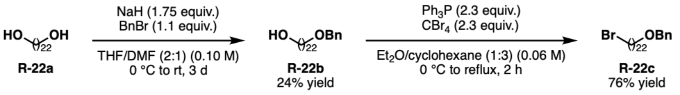

22-(benzyloxy)docosan-1-ol (R-22b)

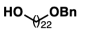

In a 250 mL dry two-neck round bottom flask, docosan-1,22-diol **R-22a** (4.80 g, 14.0 mmol, 1.0 equiv.) was dissolved in dry THF (95 mL) and dry DMF (45 mL) under an argon atmosphere. The reaction mixture was cooled to 0 °C and stirred for 5 minutes. Then sodium hydride (616 mg, 15.4 mmol, 1.1 equiv.; 60% dispersed in mineral oil) was added in one portion, and stirring was continued at 0 °C for 45 minutes. After that, benzyl bromide (1.85 mL, 15.4 mmol, 1.1 equiv.) was added dropwise at the same temperature, and the reaction was continued for 1 d at room temperature. After 1 d, an excess of sodium hydride (364 mg, 9.10 mmol, 0.65 equiv.; 60% dispersed in mineral oil) was added at room temperature, and the reaction continued for 2 d. Then, the excess sodium hydride was destroyed by the addition of water at 0 °C. The reaction mixture was transferred to a separatory funnel and worked up with ethyl acetate (4x 125 mL). The combined organic phase was washed with water (4x 125 mL) and brine solution (1x 150 mL), dried over anhydrous $MgSO_4$, and concentrated under reduced pressure. The crude material was purified by silica gel column chromatography (0-30% EtOAc in CyH) to yield the corresponding alkylated product **R-22b** (1.45 g, 3.35 mmol, 24% yield) as a white solid.

$\mathbf{R}_f$ 0.54 (3:2 cyclohexane: EtOAc).

mp: 71 – 75 °C

^{1}H NMR (500 MHz, $CDCl_3$, 298K) δ 7.38 – 7.31 (m, 4H), 7.30 – 7.26 (m, 1H), 4.50 (s, 2H), 3.64 (td, J = 6.5, 4.6 Hz, 2H), 3.46 (t, J = 6.7 Hz, 2H), 1.65 – 1.59 (m, 2H), 1.59 – 1.53 (m, 2H), 1.38 – 1.23 (m, 37H).

^{13}C NMR (126 MHz, $CDCl_3$, 298K) δ 138.9, 128.5, 127.8, 127.6, 73.0, 70.7, 63.3, 33.0, 29.9, 29.8, 29.8, 29.8, 29.8, 29.8, 29.8, 29.6, 29.6, 26.35, 25.9 (other carbon peaks are missing due to overlap).

HRMS (ESI$^+$) *m/z* calculated for $C_{29}H_{52}AgO_2$ [M + Ag$^+$]: 539.3013; found 539.3014.

IR ν_{max}(thin film)/cm^{-1} 3314br, 2912s, 2843s, 1460m, 1360w, 1101m, 1060m, 1020w, 912w, 746m, 729m, 719m, 696m, 613w.

(((22-bromodocosyl)oxy)methyl)benzene (R-22c)

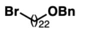

Chemical Formula: $C_{29}H_{51}BrO$
Molecular Weight: 495.63

In a 100 mL two-neck dry round bottom flask, a solution of triphenylphosphine (1.16 g, 4.42 mmol, 2.3 equiv.) in 6 mL diethyl ether at 0 ˚C was treated with carbon tetrabromide (1.46 g, 4.42 mmol, 2.3 equiv.), whereupon a yellow precipitate was formed. The mixture was allowed to react for 30 minutes at 0 ˚C. A solution of the corresponding alcohol **R-22b** (0.83 g, 1.92 mmol, 1.0 equiv.) in Et_2O/cyclohexane (2 mL/24 mL) was then added dropwise, and the mixture was heated to reflux for 2 hours. The suspension was cooled to room temperature, and cyclohexane was added. After filtration, the filtrate was concentrated under reduced pressure. The obtained residue was then purified by flash chromatography (0-5% EtOAc in CyH) to afford the bromide **R-22c** (0.72 g, 1.45 mmol, 76% yield) as a white solid.

R*f* 0.60 (20:1 cyclohexane: EtOAc).

mp: 41 – 43 °C

^{1}H NMR (500 MHz, $CDCl_3$, 298K) δ 7.36 – 7.32 (m, 4H), 7.30 – 7.26 (m, 1H), 4.50 (s, 2H), 3.46 (t, J = 6.7 Hz, 2H), 3.41 (t, J = 6.9 Hz, 2H), 1.88 – 1.82 (m, 2H), 1.61 (dq, J = 8.3, 6.7 Hz, 2H), 1.46 – 1.40 (m, 2H), 1.35 (ddd, J = 9.9, 6.4, 2.2 Hz, 2H), 1.32 – 1.22 (m, 32H).

^{13}C NMR (126 MHz, $CDCl_3$, 298K) δ 138.9, 128.5, 127.8, 127.6, 73.0, 70.7, 34.2, 33.0, 29.9, 29.9, 29.8, 29.8, 29.8, 29.8, 29.8, 29.8, 29.7, 29.6, 29.6, 28.9, 28.3, 26.4 (some carbon signal are missing due to overlap with other signals).

HRMS (ESI$^+$) *m/z* calculated for $C_{29}H_{52}BrO$ [M + H$^+$]: 495.3196; found 495.3194.

IR ν_{max}(thin film)/cm^{-1} 2010s, 2843s, 1492w, 1468s, 1452m, 1363m, 1266w, 1235w, 1220w, 1206w, 1113s, 1073m, 1026m, 991w, 990w, 732s, 717s, 694s, 638m.

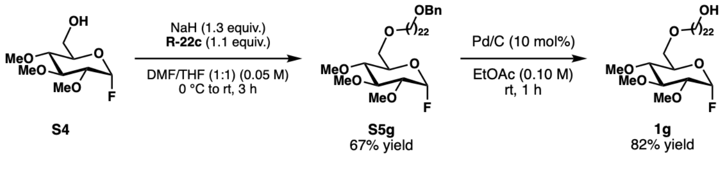

(2*R*,3*R*,4*S*,5*R*,6*R*)-2-(((22-(benzyloxy)docosyl)oxy)methyl)-6-fluoro-3,4,5-trimethoxytetrahydro-2*H*-pyran (S5g)

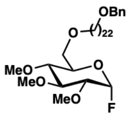

Chemical Formula: $C_{38}H_{67}FO_6$
Molecular Weight: 638.95

In a 50 mL dry two-neck round bottom flask, compound **S4** (168 mg, 0.750 mmol, 1.0 equiv.) was dissolved in dry DMF (8 mL) under an argon atmosphere. The reaction mixture was cooled to 0 °C and stirred for 5 minutes. Then sodium hydride (39.0 mg, 0.975 mmol, 1.3 equiv. ; 60% dispersed in mineral oil) was added in one portion, and stirring was continued at 0 °C for 45 minutes. After that, a solution of bromide **R-22c** (409 mg, 0.825 mmol, 1.1 equiv.) in dry THF (8 mL) was added dropwise at the same temperature, and the reaction was continued for 3 h at room temperature. After the completion of the reaction (monitored by TLC checking), the excess sodium hydride was destroyed by the addition of water. The reaction mixture was transferred to a separatory funnel and worked up with ethyl acetate (4x 20 mL). The combined organic phase was washed with water (4x 20 mL) and brine solution (1x 25 mL), dried over anhydrous $MgSO_4$, and concentrated under reduced pressure. The crude material was purified by silica gel column chromatography (0-10% EtOAc in CyH) to yield the product **S5g** (320 mg, 0.500 mmol, 67% yield) as a white solid.

R_f 0.55 (3:7 cyclohexane: EtOAc)

mp: 43 – 45 °C

^{1}H NMR (500 MHz, $CDCl_3$, 298K) δ 7.36-7.32 (m, 4H), 7.30 – 7.26 (m, 1H), 5.68 (dd, *J* = 53.3, 2.7 Hz, 1H), 4.50 (s, 2H), 3.86 – 3.73 (m, 1H), 3.70 – 3.60 (m, 5H), 3.56 (s, 3H), 3.55 (s, 3H), 3.54 – 3.51 (m, 1H), 3.50 – 3.44 (m, 3H), 3.41 (dt, *J* = 9.4, 6.9 Hz, 1H), 3.33 (ddd, *J* = 10.0, 9.1, 0.7 Hz, 1H), 3.21 (ddd, *J* = 25.7, 9.6, 2.7 Hz, 1H), 1.78 – 1.51 (m, 4H), 1.42 – 1.32 (m, 4H), 1.29-1.22 (m, 32H).

^{19}F NMR (470 MHz, $CDCl_3$, 298K) δ -149.44.

^{13}C NMR (126 MHz, $CDCl_3$, 298K) δ 138.9, 128.5, 127.8, 127.6, 105.2 (d, *J* = 226.1 Hz), 83.1, 81.5 (d, *J* = 24.8 Hz), 78.5, 73.0, 72.8 (d, *J* = 4.1 Hz), 71.9, 70.7, 68.6, 61.1, 60.7, 59.3, 29.9, 29.9, 29.9, 29.8, 29.8, 29.8, 29.8, 29.8, 29.8, 29.7, 29.6, 29.6, 26.3, 26.3 (other carbon signal was not observed due to overlap).

HRMS (ESI^+) *m/z* calculated for $C_{38}H_{67}FNaO_6$ [M + Na^+]: 661.4814; found 661.4818.

IR ν_{max}(thin film)/cm^{-1} 2912s, 2844s, 1466m, 1364m, 1161m, 1103s, 1015m, 886m, 848w, 752m, 731m, 695m, 608w.

22-(((2*R*,3*R*,4*S*,5*R*,6*R*)-6-fluoro-3,4,5-trimethoxytetrahydro-2*H*-pyran-2-yl)methoxy)docosan-1-ol (1g)

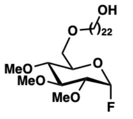

Chemical Formula: $C_{31}H_{61}FO_6$
Molecular Weight: 548.82

Compound **1g** was synthesized according to the **GP3** using compound **S5g** (288 mg, 0.450 mmol, 1.0 equiv.) palladium on carbon (10%, 48.0 mg, 45.0 µmol, 0.10 equiv.) The crude material was purified by silica gel column chromatography (10-50% EtOAc in CyH) to yield compound **1g** (205 mg, 0.374 mmol, 83%) as a white solid.

R_f 0.60 (3:7 cyclohexane: EtOAc).

mp: 75 – 78 °C

^{1}H NMR (500 MHz, $CDCl_3$, 298K) δ 5.67 (dd, *J* = 53.3, 2.7 Hz, 1H), 3.80 – 3.74 (m, 1H), 3.66 – 3.59 (m, 7H), 3.55 (s, 3H), 3.54 (s, 3H), 3.54 – 3.47 (m, 2H), 3.41 (dt, *J* = 9.4, 6.9 Hz, 1H), 3.32 (t, *J* = 9.6 Hz, 1H), 3.21 (ddd, *J* = 25.7, 9.6, 2.7 Hz, 1H), 1.69 – 1.52 (m, 4H), 1.38-1.25 (m, 36H).

^{19}F NMR (470 MHz, $CDCl_3$, 298K) δ -149.44.

^{13}C NMR (126 MHz, $CDCl_3$, 298K) δ 105.2 (d, *J* = 226.1 Hz), 83.1, 81.5 (d, *J* = 24.8 Hz), 78.5, 72.8 (d, *J* = 4.0 Hz), 71.9, 68.6, 63.2, 61.1, 60.7, 59.3, 33.0, 29.8, 29.8, 29.8, 29.8, 29.8, 29.8, 29.8, 29.7, 29.7, 29.6, 29.6, 16.3, 25.9 (other carbon signal was not observed due to overlap).

HRMS (ESI^+) *m/z* calculated for $C_{31}H_{61}FNaO_6$ [M + Na^+]: 571.4344; found 571.4355.

IR ν_{max}(thin film)/cm^{-1} 3547m, 2919s, 2843s, 1469m, 1460m, 1381m, 1336w, 1165s, 1133s, 1102s, 1074s, 1014m, 994s, 976m, 936w, 907w, 881m, 851w, 753m, 718w.

10.3. Synthesis of cyclization substrate with secondary alcohol as nucleophile

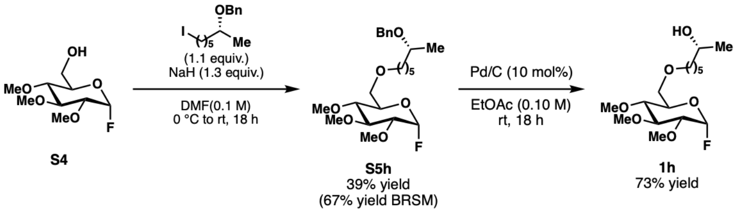

(2*R*,3*R*,4*S*,5*R*,6*R*)-2-((((*R*)-6-(benzyloxy)heptyl)oxy)methyl)-6-fluoro-3,4,5-trimethoxytetrahydro-2*H*-pyran (S5h)

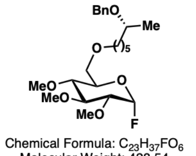

In a 50 mL dry two-neck round-bottom flask, compound **S4** (168 mg, 0.750 mmol, 1.0 equiv.) was dissolved in dry DMF (6.5 mL) under an argon atmosphere. The reaction mixture was cooled to 0 °C and stirred for 5 minutes. Then sodium hydride (39.0 mg, 0.975 mmol, 1.3 equiv. ; 60% dispersed in mineral oil) was added in one portion, and stirring was continued at 0 °C for 45 minutes. After that, a solution of the corresponding iodide (274 mg, 0.825 mmol, 1.1 equiv.) in dry DMF (1 mL) was added dropwise at the same temperature, and the reaction was continued for 18 h at room temperature. Afterward, the excess sodium hydride was destroyed by the addition of water. The reaction mixture was transferred to a separatory funnel and worked up with ethyl acetate (4x 20 mL). The combined organic phase was washed with water (4x 20 mL) and brine solution (1x 25 mL), dried over anhydrous $MgSO_4$, and concentrated under reduced pressure. The crude material was purified by silica gel column chromatography (0-15% EtOAc in CyH) to yield the product **S5h** (125 mg, 0.292 mmol, 39% yield) as a gummy liquid. Also, 70 mg of starting material was recovered, indicating the actual yield of the reaction is 67%.

R_f 0.33 (3:1 cyclohexane: EtOAc)

^{1}H NMR (500 MHz, $CDCl_3$, 298K) δ 7.38 – 7.31 (m, 4H), 7.30 – 7.22 (m, 1H), 5.67 (dd, *J* = 53.3, 2.7 Hz, 1H), 4.56 (d, *J* = 11.8 Hz, 1H), 4.45 (d, *J* = 11.8 Hz, 1H), 3.91 – 3.75 (m, 1H), 3.65 (s, 3H), 3.63 (t, *J* = 2.8 Hz, 2H), 3.55 (s, 3H), 3.54 (s, 3H), 3.54 – 3.47 (m, 3H), 3.41 (dt, *J* = 9.5, 6.9 Hz, 1H), 3.32 (ddd, *J* = 10.1, 9.1, 0.7 Hz, 1H), 3.21 (ddd, *J* = 25.7, 9.6, 2.7 Hz, 1H), 1.87 – 1.49 (m, 3H), 1.51 – 1.41 (m, 2H), 1.40 – 1.28 (m, 3H), 1.19 (d, *J* = 6.1 Hz, 3H).

^{19}**F NMR** (470 MHz, $CDCl_3$, 298K) δ -149.44.

^{13}**C NMR** (126 MHz, $CDCl_3$, 298K) δ 139.3, 128.5, 127.8, 127.5, 105.2 (d, *J* = 226.2 Hz), 83.1, 81.5 (d, *J* = 24.8 Hz), 78.5, 74.9, 72.8 (d, *J* = 4.1 Hz), 71.8, 70.4, 68.7, 61.2, 60.7, 59.3, 36.8, 29.7, 26.4, 25.5, 19.8.

HRMS (ESI$^+$) *m/z* calculated for $C_{23}H_{37}FNaO_6$ [M + Na$^+$]: 451.2466; found 451.2463.

IR ν_{max}(thin film)/cm^{-1} 2934m, 2861m, 1454w, 1375w, 1186m, 1163s, 1106s, 1027m, 893w, 737w, 698w.

(*R*)-7-(((2*R*,3*R*,4*S*,5*R*,6*R*)-6-fluoro-3,4,5-trimethoxytetrahydro-2*H*-pyran-2-yl)methoxy)heptan-2-ol (1h)

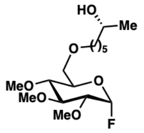

Chemical Formula: $C_{16}H_{31}FO_6$
Molecular Weight: 338.42

Compound **1h** was synthesized according to the **GP3** using compound **S5h** (117 mg, 0.273 mmol, 1.0 equiv.) and palladium on carbon (10%, 29.1 mg, 27.3 µmol, 0.10 equiv.) for 18 h. The crude material was purified by silica gel column chromatography (10-50% EtOAc in CyH) to yield compound **1h** (67.3 mg, 0.199 mmol, 73%) as a gummy liquid.

$\mathbf{R}_f$ 0.60 (3:7 cyclohexane: EtOAc).

^{1}**H NMR** (500 MHz, acetone-d_6, 298K) δ 5.69 (dd, *J* = 53.7, 2.7 Hz, 1H), 3.76 – 3.60 (m, 3H), 3.59 – 3.54 (m, 4H), 3.52 – 3.47 (m, 7H), 3.43 (dt, *J* = 9.3, 6.6 Hz, 1H), 3.40 – 3.31 (m, 2H), 3.25 – 3.20 (m, 1H), 3.16 (ddd, *J* = 25.9, 9.6, 2.7 Hz, 1H), 1.57 (dddd, *J* = 13.0, 7.9, 6.6, 1.9 Hz, 2H), 1.50 – 1.27 (m, 6H), 1.10 (d, *J* = 6.1 Hz, 3H).

^{19}**F NMR** (470 MHz, acetone-d_6, 298K) δ -150.44.

^{13}**C NMR** (126 MHz, acetone-d_6, 298K) δ 105.9 (d, *J* = 224.7 Hz), 83.9, 82.2 (d, *J* = 24.8 Hz), 79.3, 73.8 (d, *J* = 4.2 Hz), 72.0, 69.7, 67.5, 60.9, 60.7, 58.9, 40.3, 30.6, 27.1, 26.4, 24.1.

HRMS (ESI$^+$) *m/z* calculated for $C_{16}H_{31}FNaO_6$ [M + Na$^+$]: 361.1997; found 361.1993.

IR ν_{max}(thin film)/cm^{-1} 3445br, 2932m, 2860w, 1459w, 1375w, 1160s, 1102s, 1157s, 937w, 892m, 851w, 759m, 691w.

10.4. Synthesis of ether-linked galactose and mannose derivatives for the cyclization

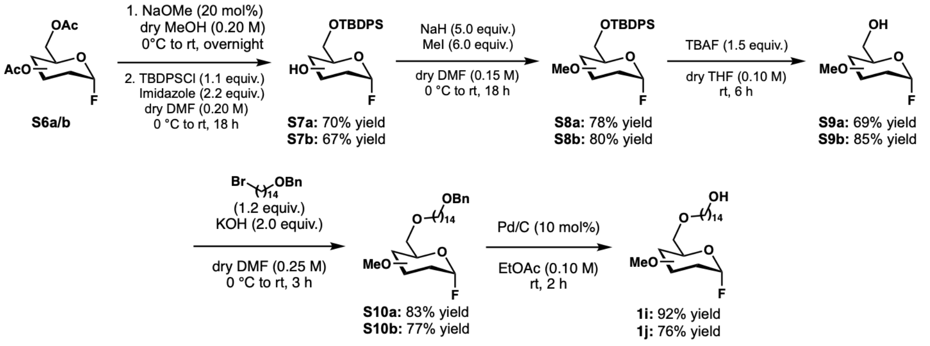

(2*R*,3*R*,4*S*,5*R*,6*R*)-2-(((*tert*-butyldiphenylsilyl)oxy)methyl)-6-fluorotetrahydro-2*H*-pyran-3,4,5-triol (S7a)

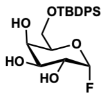

Chemical Formula: $C_{22}H_{29}FO_5Si$
Molecular Weight: 420.55

A 100 mL two-neck round bottom flask was charged with α-D-glycopyranosyl fluoride **S6a** (1.42 g, 4.05 mmol, 1.0 equiv.) in 20 mL dry MeOH. The solution was stirred and cooled down to 0 °C. After 10 minutes, solid NaOMe (43.8 mg, 0.810 mmol, 0.20 equiv.) was added in one portion and kept at 0 °C for 10 min. Subsequently, the ice bath was removed, and the reaction mixture was allowed to warm up to rt and stirred overnight. After the reaction was complete, the solvent was removed under reduced pressure. The crude material was used directly in the next step without further purification.

In a 100 mL dry two-neck round bottom flask, the crude material (1.0 equiv.) was dissolved in 20 mL dry DMF under an argon atmosphere. The mixture was cooled down to 0 °C and stirred for 5 minutes. Then imidazole (607 mg, 8.91 mmol, 2.2 equiv.) was added in one portion, followed by *tert*-butyldiphenylchlorosilane (1.16 mL, 4.46 mmol, 1.1 equiv.) at the same temperature, and stirring was continued for 18 h at room temperature. After the reaction was complete (as indicated by TLC), it was stopped by adding water. The reaction mixture was transferred to a separatory funnel and worked up with ethyl acetate (4x 40 mL). The combined organic phase was washed with water (4x 40 mL) and brine solution (1x 40 mL), dried over anhydrous $MgSO_4$, and concentrated under reduced pressure. The crude material was purified by silica gel column chromatography (30-70% EtOAc in CyH) to yield the compound **S7a** (1.20 g, 2.85 mmol, 70% yield) as a white foam.

R*f* 0.45 (9:1 DCM / MeOH)

^{1}H NMR (500 MHz, CD_3OD_SPE, 298K) δ 7.86 – 7.57 (m, 4H), 7.57 – 7.27 (m, 6H), 5.69 – 5.36 (m, 1H), 4.06 – 3.99 (m, 2H), 3.90 (dd, *J* = 10.3, 6.2 Hz, 1H), 3.84 – 3.72 (m, 3H), 1.05 (s, 9H).

^{19}F NMR (470 MHz, CD_3OD_SPE, 298K) δ -153.79.

^{13}C NMR (126 MHz, CD_3OD_SPE, 298K) δ 136.7, 134.5, 134.5, 131.0, 130.9, 128.8, 128.8, 109.3 (d, *J* = 224.1 Hz), 74.7 (d, *J* = 3.2 Hz), 71.0, 70.2, 69.9 (d, *J* = 24.8 Hz), 64.1, 27.3, 20.0.

HRMS (ESI$^+$) *m/z* calculated for $C_{22}H_{29}FNaO_5Si$ [M + Na$^+$]: 443.1660; found 443.1652.

IR ν_{max}(thin film)/cm^{-1} 3378br, 2931w, 2857w, 1471w, 1427m, 1068s, 822m, 739m, 699s, 611s.

***tert*-butyl(((2*R*,3*S*,4*S*,5*R*,6*R*)-6-fluoro-3,4,5-trimethoxytetrahydro-2*H*-pyran-2-yl)methoxy)diphenylsilane (S8a)**

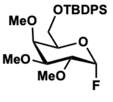

Chemical Formula: $C_{25}H_{35}FO_5Si$
Molecular Weight: 462.63

In a 50 mL dry two-neck round-bottom flask, compound **S7a** (980 mg, 2.33 mmol, 1.0 equiv.) was dissolved in 15 mL dry DMF under an argon atmosphere. The reaction mixture was cooled to 0 °C and stirred for 5 minutes. Then sodium hydride (468 mg, 11.7 mmol, 5.0 equiv.; 60% dispersed in mineral oil) was added in one portion, and stirring was continued at 0 °C for 45 minutes. After that, methyl iodide (0.87 mL, 14.0 mmol, 6.0 equiv.) was added dropwise at the same temperature, and the reaction was continued for 24 h at room temperature. After the completion of the reaction (monitored by TLC checking), the excess sodium hydride was destroyed by the addition of water. The reaction mixture was

transferred to a separatory funnel and worked up with ethyl acetate (4x 25 mL). The combined organic phase was washed with water (4x 25 mL) and brine solution (1x 25 mL), dried over anhydrous $MgSO_4$, and concentrated under reduced pressure. The crude material was purified by silica gel column chromatography (0-20% EtOAc in CyH) to yield the corresponding allylated product **S8a** (842 mg, 1.82 mmol, 78% yield) as a colourless gummy liquid.

R*f* 0.40 (3:1 cyclohexane: EtOAc)

^{1}H NMR (500 MHz, $CDCl_3$, 298K) δ 7.78 – 7.59 (m, 4H), 7.54 – 7.36 (m, 6H), 5.66 (dd, *J* = 53.9, 2.6 Hz, 1H), 3.98 (ddd, *J* = 8.8, 5.5, 1.1 Hz, 1H), 3.94 (dd, *J* = 2.7, 1.3 Hz, 1H), 3.85 (ddd, *J* = 9.9, 8.8, 1.2 Hz, 1H), 3.74 (dd, *J* = 9.7, 5.4 Hz, 1H), 3.63 (ddd, *J* = 24.5, 10.1, 2.6 Hz, 1H), 3.58 (d, *J* = 6.3 Hz, 7H), 3.55 (s, 3H), 1.08 (s, 9H).

^{19}F NMR (470 MHz, $CDCl_3$, 298K) δ -150.26.

^{13}C NMR (126 MHz, $CDCl_3$, 298K) δ 135.7, 135.7, 133.2, 133.2, 130.0, 130.0, 128.0, 127.9, 105.6 (d, *J* = 225.9 Hz), 80.2, 77.8 (d, *J* = 23.9 Hz), 74.8, 72.9 (d, *J* = 3.0 Hz), 61.5, 61.4, 59.4, 58.4, 27.0, 19.4.

HRMS (ESI^+) *m/z* calculated for $C_{25}H_{35}FNaO_5Si$ [M + Na^+]: 485.2130; found 485.2123.

IR ν_{max}(thin film)/cm^{-1} 2929m, 2857w, 1468w, 1169m, 1146m, 1111s, 1045w, 838w, 824w, 739m.

((2*R*,3*S*,4*S*,5*R*,6*R*)-6-fluoro-3,4,5-trimethoxytetrahydro-2*H*-pyran-2-yl)methanol (S9a)

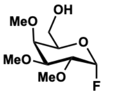

Chemical Formula: $C_9H_{17}FO_5$
Molecular Weight: 224.23

In a 50 mL dry two-neck round bottom flask, compound **S8a** (486 mg, 1.05 mmol, 1.0 equiv.) was dissolved in 10 mL dry THF under an argon atmosphere. The reaction mixture was cooled to 0 °C and stirred for 5 minutes. Then TBAF (1.58 mL, 1.58 mmol, 1.5 equiv., 1.0 M in THF) was added dropwise at the same temperature, and the reaction was continued overnight at room temperature. After the completion of the reaction (monitored by TLC checking), the reaction mixture was passed through a plug of neutral Al_2O_3, and the flask was washed with ethyl acetate (3x 15 mL). Then the combined organic mixture was concentrated under reduced pressure, purified by silica gel column chromatography (10-70% EtOAc in CyH) to yield the corresponding alcohol **S9a** (163 mg, 0.727 mmol, 69% yield) as a white solid.

R*f* 0.30 (1:4 cyclohexane: EtOAc)

mp: 98 – 101 °C

^{1}H NMR (500 MHz, CD_3OD_SPE, 298K) δ 5.79 – 5.60 (m, 1H), 3.93 – 3.85 (m, 2H), 3.67 (dd, *J* = 6.6, 0.5 Hz, 2H), 3.62 – 3.55 (m, 1H), 3.55 (s, 4H), 3.51 (s, 3H), 3.51 (s, 3H).

^{19}F NMR (470 MHz, CD_3OD_SPE, 298K) δ -151.86.

^{13}C NMR (126 MHz, CD_3OD_SPE, 298K) δ 106.7 (d, *J* = 224.7 Hz), 81.2, 78.9 (d, *J* = 24.0 Hz), 76.5, 74.8 (d, *J* = 3.2 Hz), 61.6, 61.6, 59.3, 58.2.

HRMS (ESI^+) *m/z* calculated for $C_9H_{17}FNaO_5$ [M + Na^+]: 247.0952; found 247.0951.

IR ν_{max}(thin film)/cm^{-1} 3426br, 2988w, 2934w, 2830w, 1446w, 1340w, 1153m, 1130m, 1099s, 1078s, 1063s, 1038s, 965s, 913m, 832m, 739s, 699m, 657m.

(2*R*,3*S*,4*S*,5*R*,6*R*)-2-(((14-(benzyloxy)tetradecyl)oxy)methyl)-6-fluoro-3,4,5-trimethoxytetrahydro-2*H*-pyran (S10a)

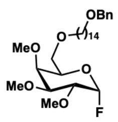

Chemical Formula: $C_{30}H_{51}FO_6$
Molecular Weight: 526.73

Compound **S10a** was synthesized according to the **GP2** using compound **S9a** (135 mg, 0.600 mmol, 1.0 equiv.), KOH (67.3 mg, 1.20 mmol, 2.0 equiv.) and the corresponding bromide[4] (276 mg, 0.720 mmol, 1.2 equiv.) in 2.5 mL dry DMF. The crude material was purified by silica gel column chromatography (0-15% EtOAc in CyH) to yield the product **S10a** (263 mg, 0.500 mmol, 83% yield) as a white solid.

R*f* 0.33 (3:1 cyclohexane: EtOAc).

mp: 34 – 36 °C

^{1}H NMR (500 MHz, $CDCl_3$, 298K) δ 7.34 (d, *J* = 4.4 Hz, 4H), 7.31 – 7.25 (m, 1H), 5.71 (dd, *J* = 53.7, 2.7 Hz, 1H), 4.50 (s, 2H), 4.15 – 4.00 (m, 1H), 3.80 (dd, *J* = 2.8, 1.2 Hz, 1H), 3.68 – 3.60 (m, 1H), 3.59 – 3.57 (m, 4H), 3.57 – 3.52 (m, 8H), 3.51 – 3.42 (m, 4H), 1.69 – 1.50 (m, 4H), 1.47 – 1.16 (m, 20H).

^{19}F NMR (470 MHz, $CDCl_3$, 298K) δ -150.48.

^{13}C NMR (126 MHz, $CDCl_3$, 298K) δ 138.9, 128.5, 127.8, 127.6, 105.6 (d, *J* = 226.0 Hz), 79.9, 77.6 (d, *J* = 23.7 Hz), 75.3, 73.0, 71.9, 71.5 (d, *J* = 3.1 Hz), 70.7, 68.4, 61.5, 59.4, 58.3, 29.9, 29.8, 29.8, 29.8, 29.6, 29.6, 26.4, 26.3 (other carbon signal was not observed due to overlap).

HRMS (ESI$^+$) *m/z* calculated for $C_{30}H_{51}FNaO_6$ [M + Na$^+$]: 549.3562; found 549.3564.

IR ν_{max}(thin film)/cm^{-1} 2919s, 2849s, 1497w, 1467m, 1365m, 1241w, 1174s, 1140s, 1119s, 1104s, 1090s, 1072, 1037s, 960m, 910m, 875m, 831m,, 754m, 732s, 697m, 655m.

14-(((2*R*,3*S*,4*S*,5*R*,6*R*)-6-fluoro-3,4,5-trimethoxytetrahydro-2*H*-pyran-2-yl)methoxy)tetradecan-1-ol (1i)

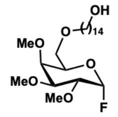

Chemical Formula: $C_{23}H_{45}FO_6$
Molecular Weight: 436.61

Compound **1i** was synthesized according to the **GP3** using compound **S10a** (233 mg, 0.442 mmol, 1.0 equiv.) using palladium on carbon (10%, 47.0 mg, 44.2 μmol, 0.10 equiv.) The crude material was purified by silica gel column chromatography (20-50% EtOAc in CyH) to yield the product **1i** (178 mg, 0.408 mmol, 92% yield) as a white solid.

R*f* 0.50 (1:4 cyclohexane: EtOAc).

mp: 72 – 75 °C

^{1}H NMR (500 MHz, acetone-*d*$_6$, 298K) δ 5.90 – 5.43 (m, 1H), 4.19 – 3.91 (m, 1H), 3.91 – 3.78 (m, 1H), 3.61 – 3.52 (m, 4H), 3.52 – 3.50 (m, 4H), 3.49 (s, 3H), 3.47 (d, *J* = 0.5 Hz, 3H), 3.47 – 3.41 (m, 2H), 3.35 (t, *J* = 5.3 Hz, 1H), 1.56 (dq, *J* = 8.3, 6.5 Hz, 2H), 1.53 – 1.47 (m, 2H), 1.38 – 1.30 (m, 20H).

^{19}**F NMR** (470 MHz, acetone-d_6, 298K) δ -150.72.

^{13}**C NMR** (126 MHz, acetone-d_6, 298K) δ 106.7 (d, J = 224.5 Hz), 80.7, 78.4 (d, J = 23.9 Hz), 76.4, 72.5 (d, J = 3.2 Hz), 71.9, 69.6, 62.5, 61.3, 59.1, 58.2, 33.8, 30.5, 30.4, 30.4, 30.4, 30.4, 30.4, 30.4, 30.4, 30.2, 26.9, 26.7.

HRMS (ESI$^+$) *m/z* calculated for $C_{23}H_{45}FNaO_6$ [M + Na$^+$]: 459.3092; found 459.3095.

IR ν_{max}(thin film)/cm^{-1} 3347br, 2919s, 2851s, 1467m, 1366w, 1257w, 1173s, 1136m, 1113s, 1074s, 1042s, 967s, 911w, 834m, 754m, 659m.

(2*R*,3*S*,4*S*,5*S*,6*R*)-2-(((*tert*-butyldiphenylsilyl)oxy)methyl)-6-fluorotetrahydro-2*H*-pyran-3,4,5-triol (S7b)

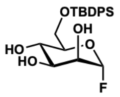

Chemical Formula: $C_{22}H_{29}FO_5Si$
Molecular Weight: 420.55

A 100 mL two-neck round-bottom flask was charged with α-D-mannosyl fluoride **S6b** (2.13 g, 6.08 mmol, 1.0 equiv.) in 30 mL dry MeOH. The solution was stirred and cooled down to 0 °C. After 10 minutes, solid NaOMe (65.9 mg, 1.22 mmol, 0.20 equiv.) was added in one portion and kept at 0 °C for 10 min. Subsequently, the ice bath was removed, and the reaction mixture was allowed to warm up to rt and stirred overnight. After the reaction was complete, the solvent was removed under reduced pressure. The crude material was used directly in the next step without further purification.

In a 100 mL dry two-neck round-bottom flask, the crude material (1.0 equiv.) was dissolved in 30 mL dry DMF under an argon atmosphere. The mixture was cooled down to 0 °C and stirred for 5 minutes. Then imidazole (912 mg, 13.4 mmol, 2.2 equiv.) was added in one portion, followed by *tert*-butyldiphenylchlorosilane (1.74 mL, 6.69 mmol, 1.1 equiv.) at the same temperature, and stirring was continued for 18 h at room temperature. After the reaction was complete (as indicated by TLC), it was stopped by adding water. The reaction mixture was transferred to a separatory funnel and worked up with ethyl acetate (4x 40 mL). The combined organic phase was washed with water (4x 40 mL) and brine solution (1x 40 mL), dried over anhydrous $MgSO_4$, and concentrated under reduced pressure. The crude material was purified by silica gel column chromatography (30-70% EtOAc in CyH) to yield the compound **S7b** (1.71 g, 4.07 mmol, 67% yield) as a white foam.

R*f* 0.50 (9:1 DCM / MeOH)

^{1}**H NMR** (500 MHz, CD_3OD_SPE, 298K) δ 7.78 – 7.67 (m, 4H), 7.43 – 7.35 (m, 6H), 5.56 (dd, J = 50.5, 1.9 Hz, 1H), 3.98 (dd, J = 11.2, 2.0 Hz, 1H), 3.95 (dd, J = 3.4, 1.6 Hz, 1H), 3.92 (dd, J = 11.2, 5.1 Hz, 1H), 3.83 (t, J = 9.7 Hz, 1H), 3.74 (ddd, J = 9.9, 5.2, 2.0 Hz, 1H), 3.70 (ddd, J = 9.5, 3.5, 2.2 Hz, 1H), 1.05 (s, 9H).

^{19}**F NMR** (470 MHz, CD_3OD_SPE, 298K) δ -139.01.

^{13}**C NMR** (126 MHz, CD_3OD_SPE, 298K) δ 136.9, 136.8, 134.9, 134.8, 130.7, 130.7, 128.7, 128.7, 109.8 (d, J = 219.2 Hz), 77.4 (d, J = 2.4 Hz), 72.1 (d, J = 2.1 Hz), 70.6 (d, J = 36.5 Hz), 67.5, 64.6, 27.3, 20.2.

HRMS (ESI$^+$) *m/z* calculated for $C_{22}H_{29}FNaO_5Si$ [M + Na$^+$]: 443.1660; found 443.1652.

IR ν_{max}(thin film)/cm^{-1} 3391br, 2931m, 2857m, 1658w, 1427m, 1176w, 1139m, 1104s, 966s, 821m, 740m, 700s, 609s.

tert-butyl(((2R,3R,4S,5S,6R)-6-fluoro-3,4,5-trimethoxytetrahydro-2H-pyran-2-yl)methoxy)diphenylsilane (S8b)

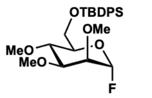

Chemical Formula: $C_{25}H_{35}FO_5Si$
Molecular Weight: 462.63

In a 50 mL dry two-neck round-bottom flask, compound **S7b** (1.44 g, 3.42 mmol, 1.0 equiv.) was dissolved in 21 mL dry DMF under an argon atmosphere. The reaction mixture was cooled to 0 °C and stirred for 5 minutes. Then sodium hydride (684 mg, 17.1 mmol, 5.0 equiv.; 60% dispersed in mineral oil) was added in one portion, and stirring was continued at 0 °C for 45 minutes. After that, methyl iodide (1.28 mL, 20.5 mmol, 6.0 equiv.) was added dropwise at the same temperature, and the reaction was continued for 24 h at room temperature. After the completion of the reaction (monitored by TLC checking), the excess sodium hydride was destroyed by the addition of water. The reaction mixture was transferred to a separatory funnel and worked up with ethyl acetate (4x 25 mL). The combined organic phase was washed with water (4x 25 mL) and brine solution (1x 25 mL), dried over anhydrous $MgSO_4$, and concentrated under reduced pressure. The crude material was purified by silica gel column chromatography (0-20% EtOAc in CyH) to yield the corresponding allylated product **S8b** (1.27 g, 2.74 mmol, 80% yield) as a colourless gummy liquid.

R_f 0.46 (3:1 cyclohexane: EtOAc)

^{1}H NMR (500 MHz, $CDCl_3$, 298K) δ 7.76 – 7.68 (m, 4H), 7.53 – 7.30 (m, 6H), 5.71 (dd, J = 50.7, 2.0 Hz, 1H), 3.96 (dd, J = 11.5, 3.2 Hz, 1H), 3.90 – 3.80 (m, 2H), 3.73 (ddd, J = 3.1, 2.1, 0.8 Hz, 1H), 3.67 – 3.61 (m, 1H), 3.58 – 3.51 (m, 10H), 1.06 (s, 9H).

^{19}F NMR (470 MHz, $CDCl_3$, 298K) δ -139.85.

^{13}C NMR (126 MHz, $CDCl_3$, 298K) δ 136.1, 135.8, 134.0, 133.5, 129.7, 129.7, 127.8, 127.6, 105.8 (d, J = 220.6 Hz), 80.6 (d, J = 1.8 Hz), 75.9 (d, J = 34.8 Hz), 75.2 (d, J = 1.7 Hz), 75.0, 62.6, 60.9, 59.1, 58.1, 26.9, 19.6.

HRMS (ESI$^+$) *m/z* calculated for $C_{25}H_{35}FNaO_5Si$ [M + Na$^+$]: 485.2130; found 485.2126.

IR ν_{max}(thin film)/cm^{-1} 2931m, 2857w,. 1472w, 1420w, 1388w, 1361w, 1292w, 1187m, 1100s, 1044m, 1018s, 951m, 823m, 793m, 741m, 701s, 648m, 609m.

((2R,3R,4S,5S,6R)-6-fluoro-3,4,5-trimethoxytetrahydro-2H-pyran-2-yl)methanol (S9b)

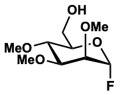

Chemical Formula: $C_9H_{17}FO_5$
Molecular Weight: 224.23

In a 50 mL dry two-neck round-bottom flask, compound **S8b** (1.22 g, 2.64 mmol, 1.0 equiv.) was dissolved in 25 mL dry THF under an argon atmosphere. The reaction mixture was cooled to 0 °C and stirred for 5 minutes. Then TBAF (3.96 mL, 3.96 mmol, 1.5 equiv., 1.0 M in THF) was added dropwise at the same temperature, and the reaction was continued overnight at room temperature. After the completion of the reaction (monitored by TLC checking), the reaction mixture was passed through a plug of neutral Al_2O_3, and the flask was washed with ethyl acetate (3x 15 mL). Then the combined organic mixture was concentrated under reduced pressure, purified by silica gel column chromatography (10-70% EtOAc in CyH) to yield the corresponding alcohol **S9a** (502 mg, 2.24 mmol, 85% yield) as a white solid.

R$_f$ 0.30 (3:7 cyclohexane: EtOAc)

mp: 50 – 52 °C

^{1}H NMR (500 MHz, CD_3OD_SPE, 298K) δ 5.66 (dd, *J* = 50.5, 2.2 Hz, 1H), 3.79 (ddd, *J* = 3.3, 2.1, 1.2 Hz, 1H), 3.76 (dd, *J* = 12.1, 2.1 Hz, 1H), 3.68 (dd, *J* = 12.1, 4.6 Hz, 1H), 3.62 – 3.53 (m, 1H), 3.52 (s, 3H), 3.51 – 3.46 (s, 8H).

^{19}F NMR (470 MHz, CD_3OD_SPE, 298K) δ -140.99.

^{13}C NMR (126 MHz, CD_3OD_SPE, 298K) δ 107.2 (d, *J* = 220.7 Hz), 82.1 (d, *J* = 1.9 Hz), 76.8 (d, *J* = 34.7 Hz), 76.5, 76.2 (d, *J* = 2.2 Hz), 61.9, 60.9, 59.7, 58.0.

HRMS (ESI$^+$) *m/z* calculated for $C_9H_{17}FNaO_5$ [M + Na$^+$]: 247.0952; found 247.0952.

IR ν_{max}(thin film)/cm^{-1} 3492br, 2929m, 1451w, 1381w, 1293w, 1186m, 1122s, 1109s, 1085s, 1030s, 937s, 909m, 796m, 656m.

(2*R*,3*R*,4*S*,5*S*,6*R*)-2-(((14-(benzyloxy)tetradecyl)oxy)methyl)-6-fluoro-3,4,5-trimethoxytetrahydro-2*H*-pyran (S10b)

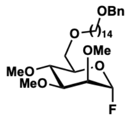

Chemical Formula: $C_{30}H_{51}FO_6$
Molecular Weight: 526.73

Compound **S10a** was synthesized according to the **GP2** using compound **S9b** (224 mg, 1.00 mmol, 1.0 equiv.), KOH (112 mg, 2.00 mmol, 2.0 equiv.) and the corresponding bromide[4] (460 mg, 1.20 mmol, 1.2 equiv.) in 2.5 mL dry DMF. The crude material was purified by silica gel column chromatography (0-15% EtOAc in CyH) to yield the product **S10a** (406 mg, 0.771 mmol, 77% yield) as a gummy liquid.

R$_f$ 0.38 (3:1 cyclohexane: EtOAc).

^{1}H NMR (500 MHz, $CDCl_3$, 298K) δ 7.36 – 7.32 (m, 4H), 7.30 – 7.26 (m, 1H), 5.68 (dd, *J* = 50.3, 2.0 Hz, 1H), 4.50 (s, 2H), 3.81 – 3.72 (m, 1H), 3.69 (ddd, *J* = 3.0, 2.1, 0.9 Hz, 1H), 3.68 – 3.61 (m, 2H), 3.57 – 3.51 (m, 9H), 3.51 (s, 3H), 3.46 (t, *J* = 6.7 Hz, 2H), 3.44 – 3.39 (m, 1H), 1.70 – 1.56 (m, 4H), 1.41 – 1.21 (m, 20H).

^{19}F NMR (470 MHz, $CDCl_3$, 298K) δ -140.23.

^{13}C NMR (126 MHz, $CDCl_3$, 298K) δ 138.9, 128.5, 127.8, 127.6, 105.7 (d, *J* = 221.8 Hz), 80.7, 76.0 (d, *J* = 34.6 Hz), 75.7, 74.1 (d, *J* = 2.4 Hz), 73.0, 72.0, 70.7, 69.5, 60.8, 59.6, 58.2, 29.9, 29.8, 29.8, 29.8, 29.8, 29.7, 29.6, 26.4, 26.3(other carbon signal was not observed due to overlap).

HRMS (ESI$^+$) *m/z* calculated for $C_{30}H_{51}FNaO_6$ [M + Na$^+$]: 549.3562; found 549.3565.

IR ν_{max}(thin film)/cm^{-1} 2923s, 2852s, 1455m, 1362m, 1292w, 1186s, 1094s, 952s, 795m, 735m, 697m, 656m.

14-(((2*R*,3*R*,4*S*,5*S*,6*R*)-6-fluoro-3,4,5-trimethoxytetrahydro-2*H*-pyran-2-yl)methoxy)tetradecan-1-ol (1j)

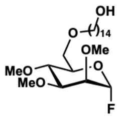

Compound **1j** was synthesized according to the **GP3** using compound **S10b** (375 mg, 0.712 mmol, 1.0 equiv.) using palladium on carbon (10%, 75.8 mg, 71.2 μmol, 0.10 equiv.) The crude material was purified by silica gel column chromatography (20-50% EtOAc in CyH) to yield the product **1i** (236 mg, 0.541 mmol, 76% yield) as a white solid.

R_f 0.53 (1:4 cyclohexane: EtOAc).

mp: 29 – 31 °C

^{1}H NMR (500 MHz, acetone-d_6, 298K) δ 5.62 (dd, J = 50.9, 2.2 Hz, 1H), 3.76 (ddd, J = 3.0, 2.2, 1.5 Hz, 1H), 3.64 – 3.60 (m, 2H), 3.59 – 3.55 (m, 1H), 3.55 – 3.49 (m, 3H), 3.48 (s, 3H), 3.48 (s, 3H), 3.46 (s, 3H), 3.45 – 3.41 (m, 2H), 3.35 (t, J = 5.3 Hz, 1H), 1.61 – 1.53 (m, 2H), 1.53 – 1.44 (m, 2H), 1.38 – 1.30 (d, J = 5.5 Hz, 20H).

^{19}F NMR (470 MHz, acetone-d_6, 298K) δ -139.42.

^{13}C NMR (126 MHz, acetone-d_6, 298K) δ 107.3 (d, J = 219.7 Hz), 81.8 (d, J = 1.9 Hz), 76.4 (d, J = 35.3 Hz), 76.3, 75.1 (d, J = 2.3 Hz), 72.0, 70.3, 62.5, 60.6, 59.7, 57.7, 33.8, 30.5, 30.4, 30.4, 30.4, 30.4, 30.4, 30.2, 26.9, 26.7.

HRMS (ESI$^+$) *m/z* calculated for $C_{23}H_{45}FNaO_6$ [M + Na$^+$]: 459.3092; found 459.3101.

IR ν_{max}(thin film)/cm^{-1} 3515w, 3386w, 2914s, 2849s, 1468m, 1383m, 1297m, 1187s, 1130m, 1086s, 1034m, 991m, 941s, 882m, 838m, 797m, 657m.

10.5. Synthesis of cyclization substrates with benzyl-protected glucose

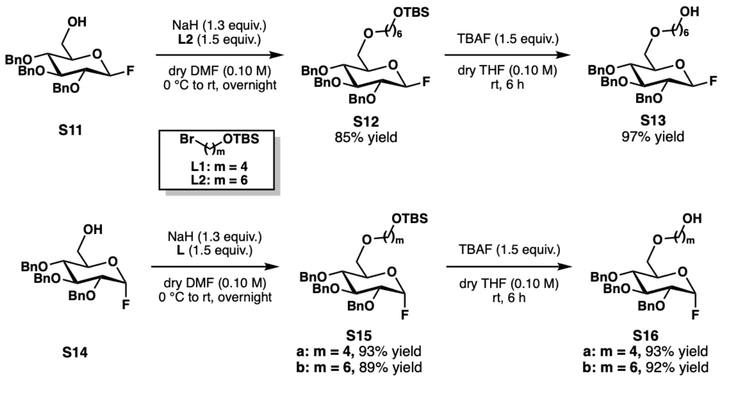

General procedure for the alkylation of primary alcohols (GP4)

In a 50 mL dry two-neck round bottom flask, compound **S11** or **S14** (1.0 equiv.)[6] was dissolved in dry DMF (0.10 M) under an argon atmosphere. The reaction mixture was cooled to 0 °C and stirred for 5 minutes. Then sodium hydride (1.3 equiv.; 60% dispersed in mineral oil) was added in one portion, and stirring was continued at 0 °C for 45 minutes. After that, a solution of the corresponding bromide **L** (1.5 equiv., dissolved in 1 mL dry DMF) was added dropwise at the same temperature, and the reaction was continued for 18 h at room temperature. After the completion of the reaction (monitored by TLC checking), the excess sodium hydride was destroyed by the addition of water. The reaction mixture was transferred to a separatory funnel and washed with ethyl acetate (4x 20 mL). The combined organic

phase was washed with water (4x 20 mL) and brine solution (1x 25 mL), dried over anhydrous $MgSO_4$, and concentrated under reduced pressure. The crude material was purified by silica gel column chromatography (1-5% EtOAc in CyH) to obtain the corresponding alkylated product **S12**/**S15** as colourless gummy oil.

General procedure for TBS-deprotection (GP5)

In a 50 mL dry two-neck round bottom flask, compound **S12** or **S15** (1.0 equiv.) was dissolved in dry THF (0.10 M) under an argon atmosphere. The reaction mixture was cooled to 0 °C and stirred for 5 minutes. Then TBAF (1.5 equiv., 1.0 M in THF) was added dropwise at the same temperature, and the reaction was continued for 6 h at room temperature. After the completion of the reaction (monitored by TLC checking), the reaction mixture was passed through a plug of neutral Al_2O_3, and the flask was washed with ethyl acetate (3x 15 mL). Then the combined organic mixture was concentrated under reduced pressure, purified by silica gel column chromatography (10-60% EtOAc in CyH) to obtain the corresponding alcohol **S13/S16** as colourless gummy oil.

***tert*-butyldimethyl((6-(((2*R*,3*R*,4*S*,5*R*,6*S*)-3,4,5-tris(benzyloxy)-6-fluorotetrahydro-2*H*-pyran-2-yl)methoxy)hexyl)oxy)silane (S12)**

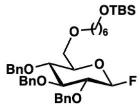

Chemical Formula: $C_{39}H_{55}FO_6Si$
Molecular Weight: 666.95

Compound **S12** was synthesized according to **GP4** using the β-fluoride **S11** (149 mg, 0.330 mmol, 1.0 equiv.) in 3 mL dry DMF, sodium hydride (17.2 mg, 0.430 mmol, 1.3 equiv.; 60% dispersed in mineral oil) and the bromide **L2** (146 mg, 0.495 mmol, 1.5 equiv.). Silica gel column chromatography was performed (0-20% EtOAc in CyH) to yield the corresponding alkylated product **S12** (187 mg, 0.280 mmol, 85% yield) as colourless gummy oil.

R_f 0.33 (9:1 cyclohexane: EtOAc)

^{1}H NMR (500 MHz, $CDCl_3$, 298K) δ 7.41 – 7.27 (m, 15H), 5.25 (dd, *J* = 52.9, 6.7 Hz, 1H), 4.90 (d, *J* = 11.0 Hz, 1H), 4.87 – 4.83 (m, 2H), 4.79 (d, *J* = 11.0 Hz, 1H), 4.71 (d, *J* = 11.1 Hz, 1H), 4.63 (d, *J* = 10.9 Hz, 1H), 3.78 – 3.63 (m, 4H), 3.63 – 3.53 (m, 4H), 3.50 (td, *J* = 7.0, 1.9 Hz, 1H), 3.42 (ddd, *J* = 9.9, 8.1, 6.5 Hz, 1H), 1.59 (h, *J* = 5.2 Hz, 2H), 1.54 – 1.47 (m, 2H), 1.34 (tq, *J* = 7.4, 4.8 Hz, 4H), 0.89 (s, 9H), 0.04 (d, *J* = 1.1 Hz, 6H).

^{19}F NMR (470 MHz, $CDCl_3$, 298K) δ -137.90.

^{13}C NMR (126 MHz, $CDCl_3$, 298K) δ 138.4, 138.2, 137.9, 128.6, 128.6, 128.6, 128.3, 128.1, 128.0, 128.0, 128.0, 127.9, 110.0 (d, *J* = 215.7 Hz), 83.6 (d, *J* = 11.3 Hz), 81.8, 81.6, 75.6, 75.1, 75.0 (d, *J* = 5.0 Hz), 74.6, 72.0, 69.2, 63.3, 32.9, 29.8, 26.1, 26.1, 25.8, 18.5, -5.1.

HRMS (ESI^+) *m/z* calculated for $C_{39}H_{55}FNaO_6Si$ [M + Na^+]: 689.3644; found 689.3647.

IR ν_{max}(thin film)/cm^{-1} 3027w, 2924m, 2853m, 2357w, 1495w, 1452w, 1358w, 1253w, 1095s, 1027m, 834m, 775w, 734w, 697m.

6-(((2*R*,3*R*,4*S*,5*R*,6*S*)-3,4,5-tris(benzyloxy)-6-fluorotetrahydro-2*H*-pyran-2-yl)methoxy)hexan-1-ol (S13)

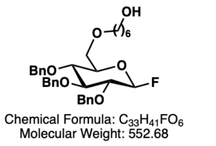

Compound **S13** was synthesized according to **GP5** using the β-fluoride **S12** (170 mg, 0.255 mmol, 1.0 equiv.) and TBAF (383 µL, 0.383 mmol, 1.5 equiv.; 1.0 M in THF) in 2.5 mL dry THF. Silica gel column chromatography was performed (10-50% EtOAc in CyH) to yield the corresponding alkylated product **S13** (137 mg, 0.248 mmol, 97%) as colourless gummy oil.

R*f* 0.40 (1:1 cyclohexane: EtOAc)

^{1}H NMR (500 MHz, $CDCl_3$, 298K) δ 7.39 – 7.26 (m, 15H), 5.25 (dd, *J* = 52.9, 6.8 Hz, 1H), 4.90 (d, *J* = 11.0 Hz, 1H), 4.85 (d, *J* = 11.0 Hz, 2H), 4.79 (d, *J* = 11.0 Hz, 1H), 4.71 (d, *J* = 11.1 Hz, 1H), 4.63 (d, *J* = 10.9 Hz, 1H), 3.79 – 3.65 (m, 4H), 3.64 – 3.54 (m, 4H), 3.54 – 3.49 (m, 1H), 3.43 (dt, *J* = 9.4, 6.7 Hz, 1H), 1.65 – 1.53 (m, 4H), 1.44 – 1.33 (m, 4H), 1.22 (s, 1H).

^{19}F NMR (470 MHz, $CDCl_3$, 298K) δ -137.81.

^{13}C NMR (126 MHz, $CDCl_3$, 298K) δ 138.3, 138.1, 137.7, 128.5, 128.5, 128.4, 128.2, 127.9, 127.9, 127.9, 127.8, 109.9 (d, *J* = 215.8 Hz), 83.5 (d, *J* = 11.4 Hz), 81.6, 81.4, 75.5, 75.0, 74.9 (d, *J* = 5.0 Hz), 74.5 (d, *J* = 2.4 Hz), 71.7, 69.1, 62.9, 32.7, 29.6, 26.0, 25.6 (one carbon is missing due to overlap with other signal).

HRMS (ESI^+) *m/z* calculated for $C_{33}H_{41}FNaO_6$ [M + Na^+]: 575.2779; found 575.2790.

IR ν_{max}(thin film)/cm^{-1} 3432br, 3026w, 2925m, 2857m, 1494w, 1452m, 1356m, 1306w, 1208w, 1088s, 1057s, 1026s, 909w, 734s, 695s, 607w.

***tert*-butyldimethyl(4-(((2*R*,3*R*,4*S*,5*R*,6*R*)-3,4,5-tris(benzyloxy)-6-fluorotetrahydro-2*H*-pyran-2-yl)methoxy)butoxy)silane (S15a)**

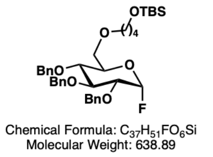

Compound **S15a** was synthesized according to **GP4** using the α-fluoride **S14** (407 mg, 0.900 mmol, 1.0 equiv.) in 9 mL dry DMF, sodium hydride (46.8 mg, 1.17 mmol, 1.3 equiv. ; 60% dispersed in mineral oil) and the bromide **L1** (361 mg, 1.35 mmol, 1.5 equiv.). Silica gel column chromatography was performed (1-15% EtOAc in CyH) to yield the corresponding alkylated product **S15a** (534 mg, 0.836 mmol, 93% yield) as colourless gummy oil.

R*f* 0.62 (7:3 cyclohexane: EtOAc)

^{1}H NMR (500 MHz, $CDCl_3$, 298K) δ 7.39 – 7.27 (m, 15H), 5.54 (dd, *J* = 53.2, 2.6 Hz, 1H), 4.96 (d, *J* = 10.9 Hz, 1H), 4.87 (dd, *J* = 12.6, 10.9 Hz, 2H), 4.80 (d, *J* = 11.9 Hz, 1H), 4.70 (d, *J* = 11.9 Hz, 1H), 4.63 (d, *J* = 10.9 Hz, 1H), 3.98 (t, *J* = 9.4 Hz, 1H), 3.91 (dt, *J* = 10.3, 2.5 Hz, 1H), 3.80 – 3.67 (m, 2H), 3.64 – 3.53 (m, 4H), 3.53 – 3.47 (m, 1H), 3.38 (dt, *J* = 9.4, 6.7 Hz, 1H), 1.66 – 1.60 (m, 2H), 1.60 – 1.48 (m, 2H), 0.88 (s, 9H), 0.03 (s, 6H).

^{19}F NMR (470 MHz, $CDCl_3$, 298K) δ -149.60.

^{13}C NMR (126 MHz, $CDCl_3$, 298K) δ 138.6, 138.3, 137.8, 128.7, 128.6, 128.6, 128.2, 128.2, 128.1, 128.0, 127.9, 127.9, 105.7 (d, J = 226.8 Hz), 81.6, 79.5 (d, J = 24.8 Hz), 76.8, 76.0, 75.3, 73.7, 72.8 (d, J = 4.0 Hz), 71.7, 68.7, 63.0, 29.7, 26.3, 26.1, 18.5, -5.2.

HRMS (ESI^+) *m/z* calculated for $C_{37}H_{51}FNaO_6Si$ [M + Na^+]: 661.3331; found 661.3330.

IR ν_{max}(thin film)/cm^{-1} 3026w, 2923s, 2853s, 2355w, 1494w, 1452m, 1358m, 1252m, 1207w, 1160s, 1009s, 1027s, 906w, 834s, 774s, 736s, 696s.

4-(((2*R*,3*R*,4*S*,5*R*,6*R*)-3,4,5-tris(benzyloxy)-6-fluorotetrahydro-2*H*-pyran-2-yl)methoxy)butan-1-ol (S16a)

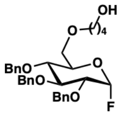

Chemical Formula: $C_{31}H_{37}FO_6$
Molecular Weight: 524.63

Compound **S16a** was synthesized according to **GP5** using the α-fluoride **S15a** (524 mg, 0.820 mmol, 1.0 equiv.) and TBAF (1.23 mL, 1.23 mmol, 1.5 equiv.; 1.0 M in THF) in 8.2 mL dry THF. Silica gel column chromatography was performed (10-50% EtOAc in CyH) to yield the corresponding alkylated product **S16a** (402 mg, 0.766 mmol, 93% yield) as colourless gummy oil.

R_f 0.47 (3:7 cyclohexane: EtOAc)

^{1}H NMR (500 MHz, $CDCl_3$, 298K) δ 7.37 – 7.27 (m, 15H), 5.56 (dd, J = 53.1, 2.7 Hz, 1H), 4.96 (d, J = 10.9 Hz, 1H), 4.90 (d, J = 11.0 Hz, 1H), 4.85 (d, J = 10.9 Hz, 1H), 4.80 (d, J = 11.8 Hz, 1H), 4.71 (d, J = 11.8 Hz, 1H), 4.62 (d, J = 11.0 Hz, 1H), 3.98 (t, J = 9.4 Hz, 1H), 3.92 (dt, J = 10.2, 2.6 Hz, 1H), 3.75 – 3.67 (m, 2H), 3.66 – 3.48 (m, 5H), 3.41 (dtd, J = 9.5, 4.9, 1.7 Hz, 1H), 1.91 (t, J = 5.8 Hz, 1H), 1.74 – 1.54 (m, 4H).

^{19}F NMR (470 MHz, $CDCl_3$, 298K) δ -149.51.

^{13}C NMR (126 MHz, $CDCl_3$, 298K) δ 138.6, 138.3, 137.8, 128.7, 128.6, 128.6, 128.2, 128.1, 128.1, 128.0, 127.9, 127.9, 105.6 (d, J = 227.1 Hz), 81.6, 79.6 (d, J = 24.8 Hz), 76.8, 76.0, 75.3, 73.7, 72.7 (d, J = 4.0 Hz), 71.7, 68.8, 62.9, 30.2, 26.7.

HRMS (ESI^+) *m/z* calculated for $C_{31}H_{37}FNaO_6$ [M + Na^+]: 547.2466; found 547.2465.

IR ν_{max}(thin film)/cm^{-1} 3406br, 3026w, 2863w, 1494w, 1452m, 1357w, 1208w, 1157m, 1068, 1025s, 907m, 850w, 733s, 694s.

***tert*-butyldimethyl((6-(((2*R*,3*R*,4*S*,5*R*,6*R*)-3,4,5-tris(benzyloxy)-6-fluorotetrahydro-2*H*-pyran-2-yl)methoxy)hexyl)oxy)silane (S15b)**

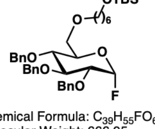

Chemical Formula: $C_{39}H_{55}FO_6Si$
Molecular Weight: 666.95

Compound **S15b** was synthesized according to **GP1** using the α-fluoride **S14** (321 mg, 0.710 mmol, 1.0 equiv.) in 7 mL dry DMF, sodium hydride (37.0 mg, 0.923 mmol, 1.3 equiv. ; 60% dispersed in mineral oil) and the bromide **L2** (316 mg, 1.07 mmol, 1.5 equiv.). Silica gel column chromatography

was (1-15% EtOAc in CyH) to yield the corresponding alkylated product **S15b** (423 mg, 0.634 mmol, 89%) as colourless gummy oil.

R*f* 0.45 (9:1 cyclohexane: EtOAc)

^{1}H NMR (500 MHz, $CDCl_3$, 298K) δ 7.38 – 7.26 (m, 15H), 5.54 (dd, *J* = 53.2, 2.6 Hz, 1H), 4.96 (d, *J* = 10.9 Hz, 1H), 4.87 (dd, *J* = 13.3, 10.9 Hz, 2H), 4.80 (d, *J* = 11.9 Hz, 1H), 4.71 (d, *J* = 11.8 Hz, 1H), 4.63 (d, *J* = 10.9 Hz, 1H), 3.98 (t, *J* = 9.4 Hz, 1H), 3.91 (ddd, *J* = 10.2, 3.3, 2.0 Hz, 1H), 3.76 – 3.66 (m, 2H), 3.62 (dd, *J* = 11.0, 2.0 Hz, 1H), 3.60 – 3.52 (m, 3H), 3.49 (ddd, *J* = 9.4, 7.1, 5.9 Hz, 1H), 3.35 (dt, *J* = 9.4, 7.0 Hz, 1H), 1.69 – 1.56 (m, 2H), 1.52 – 1.46 (m, 2H), 1.41 – 1.27 (m, 4H), 0.89 (s, 9H), 0.03 (s, 6H).

^{19}F NMR (470 MHz, $CDCl_3$, 298K) δ -149.61.

^{13}C NMR (126 MHz, $CDCl_3$, 298K) δ 138.6, 138.3, 137.8, 128.7, 128.6, 128.6, 128.2, 128.2, 128.1, 128.0, 127.9, 127.9, 105.7 (d, *J* = 226.7 Hz), 81.6, 79.5 (d, *J* = 24.8 Hz), 76.8, 76.0, 75.3, 73.7, 72.8 (d, *J* = 3.9 Hz), 71.9, 68.7, 63.3, 32.9, 29.7, 26.1, 25.8, 18.5, -5.1 (one carbon is missing due to overlap).

HRMS (ESI^+) *m/z* calculated for $C_{39}H_{55}FNaO_6Si$ [M + Na^+]: 689.3644; found 689.3648.

IR ν_{max}(thin film)/cm^{-1} 3026w, 2923m, 2852m, 1494w, 1452m, 1358m, 1251m, 1208w, 1158m, 1087s, 1026s, 906w, 832s, 772s, 732s, 694s, 661w.

6-(((2*R*,3*R*,4*S*,5*R*,6*R*)-3,4,5-tris(benzyloxy)-6-fluorotetrahydro-2*H*-pyran-2-yl)methoxy)hexan-1-ol (S16b)

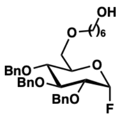

Chemical Formula: $C_{33}H_{41}FO_6$
Molecular Weight: 552.68

Compound **S16b** was synthesized according to **GP5** using the α-fluoride **S15b** (407 mg, 0.610 mmol, 1.0 equiv.) and TBAF (915 µL, 0.915 mmol, 1.5 equiv.; 1.0 M in THF) in 6 mL dry THF. Silica gel column chromatography was performed (10-50% EtOAc in CyH) to yield the corresponding alkylated product **S16b** (311 mg, 0.563 mmol, 92% yield) as colourless gummy oil.

R*f* 0.40 (1:1 cyclohexane: EtOAc)

^{1}H NMR (500 MHz, $CDCl_3$, 298K) δ 7.38 – 7.27 (m, 15H), 5.54 (dd, *J* = 53.2, 2.7 Hz, 1H), 4.95 (d, *J* = 10.8 Hz, 1H), 4.87 (dd, *J* = 17.1, 10.9 Hz, 2H), 4.80 (d, *J* = 11.9 Hz, 1H), 4.71 (d, *J* = 11.9 Hz, 1H), 4.63 (d, *J* = 11.0 Hz, 1H), 3.98 (t, *J* = 9.4 Hz, 1H), 3.91 (ddd, *J* = 10.0, 3.3, 1.9 Hz, 1H), 3.78 – 3.67 (m, 2H), 3.67 – 3.53 (m, 4H), 3.53 – 3.46 (m, 1H), 3.36 (dt, *J* = 9.5, 6.9 Hz, 1H), 1.73 – 1.50 (m, 4H), 1.44 – 1.30 (m, 4H), 1.18 (s, 1H).

^{19}F NMR (470 MHz, $CDCl_3$, 298K) δ -149.62.

^{13}C NMR (126 MHz, $CDCl_3$, 298K) δ 138.5, 138.2, 137.7, 128.6, 128.5, 128.5, 128.1, 128.0, 128.0, 127.8, 127.8, 127.8, 105.6 (d, *J* = 226.8 Hz), 81.5, 79.3 (d, *J* = 24.9 Hz), 76.7, 75.9, 75.2, 73.6, 72.7, 71.6, 68.6, 62.9, 32.7, 29.5, 26.0, 25.6.

HRMS (ESI^+) *m/z* calculated for $C_{33}H_{41}FNaO_6$ [M + Na^+]: 575.2779; found 575.2791.

IR ν_{max}(thin film)/cm^{-1} 3414br, 3026w, 2925m, 2856m, 1494w, 1452m, 1357m, 1208w, 1157s, 1068s, 1025s, 906m, 849w, 734s, 694s, 605w.

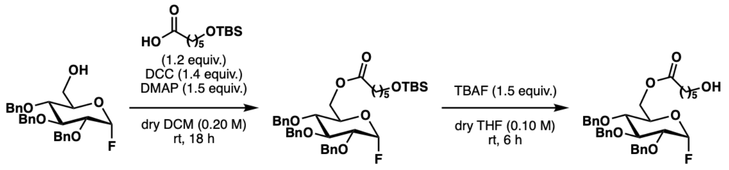

S14 S17 75% yield S18 89% yield

((2*R*,3*R*,4*S*,5*R*,6*R*)-3,4,5-tris(benzyloxy)-6-fluorotetrahydro-2*H*-pyran-2-yl)methyl 6-((*tert*-butyldimethylsilyl)oxy)hexanoate (S17)

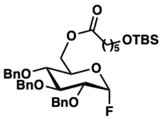

Chemical Formula: $C_{39}H_{53}FO_7Si$
Molecular Weight: 680.93

A suspension of D-glucose derivative **S14** (340 mg, 0.751 mmol, 1.0 equiv.), 6-((*tert*-butyldimethylsilyl)oxy)hexanoic acid[7] (222 mg, 0.901 mmol, 1.2 equiv.), dicyclohexylcarbodiimide (DCC; 217 mg, 1.05 mmol, 1.4 equiv.) and N,N-(di-methylamino) pyridine (DMAP; 138 mg, 1.13 mmol, 1.5 equiv.) in 4.0 mL dry dichloromethane was stirred at room temperature for 18 h. After completion of the reaction (monitored by TLC), the urea byproduct was removed by filtration, and the filtrate was evaporated. The crude material was purified by column chromatography (0-30% EtOAc in CyH) to yield the corresponding ester product **S17** (386 mg, 0.567 mmol, 75% yield) as colourless gummy oil.

R*f* 0.62 (7:3 cyclohexane: EtOAc)

^{1}H NMR (500 MHz, $CDCl_3$, 298K) δ 7.38 – 7.29 (m, 13H), 7.29 – 7.25 (m, 2H), 5.51 (dd, *J* = 52.9, 2.6 Hz, 1H), 4.98 (d, *J* = 10.8 Hz, 1H), 4.87 (dd, *J* = 12.7, 10.8 Hz, 2H), 4.80 (d, *J* = 11.8 Hz, 1H), 4.72 (d, *J* = 11.8 Hz, 1H), 4.57 (d, *J* = 10.8 Hz, 1H), 4.29 (qd, *J* = 12.2, 3.2 Hz, 2H), 4.20 – 3.93 (m, 2H), 3.65 – 3.46 (m, 4H), 2.29 (td, *J* = 7.5, 4.0 Hz, 2H), 1.62 (p, *J* = 7.6 Hz, 2H), 1.54 – 1.47 (m, 2H), 1.39 – 1.29 (m, 2H), 0.88 (s, 9H), 0.03 (s, 6H).

^{19}F NMR (470 MHz, $CDCl_3$, 298K) δ -149.53.

^{13}C NMR (126 MHz, $CDCl_3$, 298K) δ 173.4, 138.4, 137.7, 128.7, 128.7, 128.6, 128.3, 128.2, 128.2, 128.1, 128.1, 128.0, 105.3 (d, *J* = 226.8 Hz), 81.5, 79.6 (d, *J* = 25.2 Hz), 76.6, 76.1, 75.4, 73.7, 71.4, 63.1, 62.3, 34.2, 32.6, 26.1, 25.5, 24.8, 18.5, -5.1(one carbon is missing due to overlap).

IR ν_{max}(thin film)/cm^{-1} 3026w, 2924s, 2853m, 2360w, 1737s, 1495w, 1452m, 1358m, 1253m, 1208m, 1156s, 1096s, 1026s, 910w, 834s, 774s, 738s, 697s.

HRMS (ESI$^+$) *m/z* calculated for $C_{39}H_{53}FNaO_7Si$ [M + Na$^+$]: 703.3437; found 703.3442.

((2*R*,3*R*,4*S*,5*R*,6*R*)-3,4,5-tris(benzyloxy)-6-fluorotetrahydro-2*H*-pyran-2-yl)methyl 6-hydroxyhexanoate (S18)

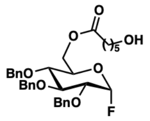

Chemical Formula: $C_{33}H_{39}FO_7$
Molecular Weight: 566.67

Compound **S18** was synthesized according to **GP5** using the α-fluoride **S17** (368 mg, 0.540 mmol, 1.0 equiv.) and TBAF (810 µL, 0.81 mmol, 1.5 equiv.; 1.0 M in THF) in 6 mL dry THF. Silica gel column chromatography was performed (10-70% EtOAc in CyH) to yield the corresponding deprotected product **S18** (272 mg, 0.480 mmol, 89% yield) as colourless gummy oil.

R_f 0.30 (1:1 cyclohexane: EtOAc)

^{1}H NMR (500 MHz, $CDCl_3$, 298 K) δ 7.40 – 7.23 (m, 15H), 5.52 (dd, *J* = 53.0, 2.6 Hz, 1H), 4.98 (d, *J* = 10.7 Hz, 1H), 4.87 (dd, *J* = 15.8, 10.8 Hz, 2H), 4.80 (d, *J* = 11.9 Hz, 1H), 4.72 (d, *J* = 11.9 Hz, 1H), 4.58 (d, *J* = 10.8 Hz, 1H), 4.33 (dd, *J* = 12.2, 2.2 Hz, 1H), 4.27 (dd, *J* = 12.2, 4.2 Hz, 1H), 4.06 – 3.97 (m, 2H), 3.61 (td, *J* = 6.0, 2.7 Hz, 2H), 3.60 – 3.49 (m, 2H), 2.31 (td, *J* = 7.4, 3.2 Hz, 2H), 1.69 – 1.59 (m, 2H), 1.60 – 1.51 (m, 2H), 1.46 – 1.32 (m, 2H), 1.26 (s, 1H).

^{19}F NMR (470 MHz, $CDCl_3$, 298 K) δ -149.55.

^{13}C NMR (126 MHz, $CDCl_3$, 298 K) δ 173.3, 138.4, 137.7, 128.7, 128.7, 128.7, 128.3, 128.2, 128.2, 128.2, 128.1, 128.0, 105.3 (d, *J* = 227.7 Hz), 81.5, 79.5 (d, *J* = 24.8 Hz), 76.6, 76.1, 75.4, 73.7, 71.4 (d, *J* = 4.3 Hz), 62.7, 62.4, 34.1, 32.4, 25.4, 24.7(one carbon is missing due to overlap).

HRMS (ESI^+) *m/z* calculated for $C_{33}H_{39}FNaO_7$ [M + Na^+]: 589.2572; found 589.2581.

IR ν_{max}(thin film)/cm^{-1} 3038br, 3026w, 2925w, 1733m, 1494w, 1452m, 1357m, 1207m, 1154s, 1068s, 1016s, 909m, 735s, 695s, 606w.

10.6. Synthesis of common allyl-protected intermediate S23

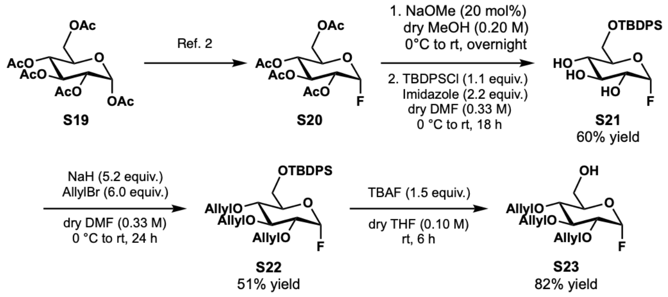

Compound **S20** was prepared from commercially available α-D-glucose pentaacetate **S19** using the reported procedure of the Miller group[2] with high α-selectivity.

(2*R*,3*S*,4*S*,5*R*,6*R*)-2-(((*tert*-butyldiphenylsilyl)oxy)methyl)-6-fluorotetrahydro-2*H*-pyran-3,4,5-triol (S21)

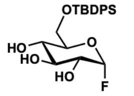

Chemical Formula: $C_{22}H_{29}FO_5Si$
Molecular Weight: 420.55

A 100 mL two-neck round bottom flask was charged with α-D-glucopyranosyl fluoride **S20** (2.72 g, 7.77 mmol, 1.0 equiv.) in 38 mL dry MeOH. The solution was stirred and cooled down to 0 °C. After 10 minutes, solid NaOMe (84.0 mg, 1.55 mmol, 0.20 equiv.) was added in one portion and kept at 0 °C for 10 min. Subsequently, the ice bath was removed, and the reaction mixture was allowed to warm up to rt and stirred overnight. After completion of the reaction, the solvent was removed under reduced pressure. The crude material was directly used for the next step without further purification.

In a 100 mL dry two-neck round bottom flask, the crude material (1.0 equiv.) was dissolved in 25 mL dry DMF under an argon atmosphere. The mixture was cooled down to 0 °C and stirred for 5 minutes. Then imidazole (1.16 g, 17.1 mmol, 2.2 equiv.) was added in one portion, followed by *tert*-butyldiphenylchlorosilane (2.22 mL, 8.55 mmol, 1.1 equiv.) at the same temperature, and stirring was continued for 18 h at room temperature. After the completion of the reaction (monitored by TLC checking), the reaction was stopped by the addition of water. The reaction mixture was transferred to a separatory funnel and worked up with ethyl acetate (4x 40 mL). The combined organic phase was washed with water (4x 40 mL) and brine solution (1x 40 mL), dried over anhydrous $MgSO_4$, and concentrated under reduced pressure. The crude material was purified by silica gel column chromatography (30-70% EtOAc in CyH) to yield the compound **S21** (1.96 g, 4.66 mmol, 60% yield) as a white solid.

R*f* 0.28 (cyclohexane / EtOAc, 1:4)

mp: 106 – 112 °C

^{1}H NMR (500 MHz, CD_3OD, 298K) δ 7.88 – 7.61 (m, 4H), 7.61 – 7.26 (m, 6H), 5.56 (dd, *J* = 53.9, 2.8 Hz, 1H), 3.98 – 3.88 (m, 2H), 3.76 (ddd, *J* = 9.7, 4.0, 2.1 Hz, 1H), 3.70 – 3.57 (m, 2H), 3.43 (ddd, *J* = 25.9, 9.4, 2.8 Hz, 1H), 1.04 (s, 9H).

^{19}F NMR (470 MHz, CD_3OD, 298K) δ -151.57.

^{13}C NMR (126 MHz, CD_3OD, 298K) δ 136.8, 136.8, 134.8, 134.7, 130.8, 130.8, 128.7, 128.7, 109.1 (d, *J* = 224.1 Hz), 76.3 (d, *J* = 3.4 Hz), 74.6, 73.3 (d, *J* = 25.3 Hz), 70.4, 63.9, 27.3, 20.2.

HRMS (ESI$^+$) *m/z* calculated for $C_{22}H_{29}FNaO_5Si$ [M + Na$^+$]: 443.1660; found 443.1661.

IR ν_{max}(thin film)/cm^{-1} 3326br, 3066w, 2926m, 2853m, 1470w, 1425m, 1388w, 1168m, 1111s, 1035s, 987w, 953w, 890w, 823w, 762m, 740m, 699s, 614m.

tert-butyldiphenyl(((2*R*,3*R*,4*S*,5*R*,6*R*)-3,4,5-tris(allyloxy)-6-fluorotetrahydro-2*H*-pyran-2-yl)methoxy)silane (S22)

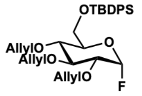

Chemical Formula: $C_{31}H_{41}FO_5Si$
Molecular Weight: 540.75

In a 50 mL dry two-neck round bottom flask, compound **S21** (1.68 g, 4.00 mmol, 1.0 equiv.) was dissolved in 12 mL dry DMF under an argon atmosphere. The reaction mixture was cooled to 0 °C and stirred for 5 minutes. Then sodium hydride (832 mg, 20.8 mmol, 5.2 equiv.; 60% dispersed in mineral oil) was added in one portion, and stirring was continued at 0 °C for 45 minutes. After that, allyl bromide (2.10 mL, 24.0 mmol, 6.0 equiv.) was added dropwise at the same temperature, and the reaction was continued for 24 h at room temperature. After the completion of the reaction (monitored by TLC checking), the excess sodium hydride was destroyed by the addition of water. The reaction mixture was transferred to a separatory funnel and worked up with ethyl acetate (4x 25 mL). The combined organic phase was washed with water (4x 25 mL) and brine solution (1x 25 mL), dried over anhydrous $MgSO_4$, and concentrated under reduced pressure. The crude material was purified by silica gel column chromatography (0-20% EtOAc in CyH) to yield the corresponding allylated product **S22** (1.09 g, 2.02 mmol, 51% yield) as a colourless gummy liquid.

R*f* 0.55 (9:1 cyclohexane: EtOAc)

^{1}H NMR (500 MHz, $CDCl_3$, 298K) δ 7.72 – 7.62 (m, 4H), 7.48 – 7.32 (m, 6H), 6.04 – 5.83 (m, 3H), 5.63 (dd, *J* = 53.5, 2.7 Hz, 1H), 5.33 (ddq, *J* = 17.0, 15.3, 1.6 Hz, 2H), 5.26 – 5.16 (m, 3H), 5.13 (dq, *J* = 10.4, 1.3 Hz, 1H), 4.37 (ddq, *J* = 12.2, 5.7, 1.6 Hz, 2H), 4.34 – 4.24 (m, 2H), 4.24 – 4.13 (m, 2H), 3.93 (dd, *J* = 11.6, 2.7 Hz, 1H), 3.86 (dd, *J* = 11.6, 1.7 Hz, 1H), 3.80 – 3.72 (m, 2H), 3.70 – 3.61 (m, 1H), 3.38 (ddd, *J* = 25.6, 9.5, 2.7 Hz, 1H), 1.06 (s, 9H).

^{19}F NMR (470 MHz, $CDCl_3$, 298K) δ -148.55.

^{13}C NMR (126 MHz, $CDCl_3$, 298K) δ 135.9, 135.7, 135.2, 134.9, 134.6, 133.7, 133.3, 129.8, 129.8, 127.8, 127.7, 117.9, 117.2, 116.9, 105.9 (d, *J* = 225.7 Hz), 81.1, 79.5 (d, *J* = 24.9 Hz), 76.3, 74.7, 74.2, 73.9 (d, *J* = 3.4 Hz), 72.9, 62.1, 27.0, 19.5.

HRMS (ESI^+) *m/z* calculated for $C_{31}H_{41}FNaO_5Si$ [M + Na^+]: 563.2600; found 563.2605.

IR ν_{max}(thin film)/cm^{-1} 2925m, 2853m, 1645w, 1470w, 1425m, 1388w, 1358w, 1157m, 1104s, 1075s, 1032s, 992s, 921s, 893w, 821m, 739m, 699s, 608m.

((2*R*,3*R*,4*S*,5*R*,6*R*)-3,4,5-tris(allyloxy)-6-fluorotetrahydro-2*H*-pyran-2-yl)methanol (S23)

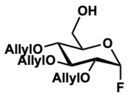

Chemical Formula: $C_{15}H_{23}FO_5$
Molecular Weight: 302.34

In a 50 mL dry two-neck round bottom flask, compound **S22** (990 mg, 1.83 mmol, 1.0 equiv.) was dissolved in 20 mL dry THF under an argon atmosphere. The reaction mixture was cooled to 0 °C and stirred for 5 minutes. Then TBAF (2.75 mL, 2.75 mmol, 1.5 equiv., 1.0 M in THF) was added dropwise at the same temperature, and the reaction was continued overnight at room temperature. After the completion of the reaction (monitored by TLC checking), the reaction mixture was passed through a plug of neutral Al_2O_3, and the flask was washed with ethyl acetate (3x 15 mL). Then the combined organic mixture was concentrated under reduced pressure, purified by silica gel column chromatography (10-70% EtOAc in CyH) to yield the corresponding alcohol **S23** (452 mg, 1.50 mmol, 82% yield) as a white solid.

R_f 0.48 (1:1 cyclohexane: EtOAc)

mp: 152 – 157 °C

^{1}H NMR (500 MHz, $CDCl_3$, 298K) δ 6.04 – 5.77 (m, 3H), 5.60 (dd, *J* = 53.1, 2.7 Hz, 1H), 5.35 – 5.25 (m, 3H), 5.24 – 5.12 (m, 3H), 4.35 (dddt, *J* = 12.6, 8.4, 5.7, 1.5 Hz, 2H), 4.31 – 4.22 (m, 2H), 4.17 (dddd, *J* = 13.8, 7.5, 2.9, 1.4 Hz, 2H), 3.85 (ddd, *J* = 11.6, 5.1, 2.9 Hz, 1H), 3.82 – 3.71 (m, 3H), 3.45 (t, *J* = 9.6 Hz, 1H), 3.35 (dddd, *J* = 25.7, 9.6, 2.9, 1.2 Hz, 1H), 1.76 (dd, *J* = 8.1, 4.9 Hz, 1H).

^{19}F NMR (470 MHz, $CDCl_3$, 298K) δ -148.39.

^{13}C NMR (126 MHz, $CDCl_3$, 298K) δ 135.1, 134.7, 134.5, 118.1, 117.5, 116.9, 105.7 (d, *J* = 227.0 Hz), 80.8, 79.2 (d, *J* = 24.6 Hz), 76.3, 74.5, 74.1, 73.5 (d, *J* = 3.5 Hz), 72.9, 61.4.

HRMS (ESI^+) *m/z* calculated for $C_{15}H_{23}FNaO_5$ [M + Na^+]: 325.1422; found 325.1415.

IR ν_{max}(thin film)/cm^{-1} 3358br, 3037w, 2977w, 2909w, 1646w, 1459w, 1425w, 1346m, 1206w, 1164s, 1114s, 1075s, 989s, 916s, 884m, 849m, 771s, 700w.

10.7. Synthesis of cyclization substrates with allyl-protected glucose

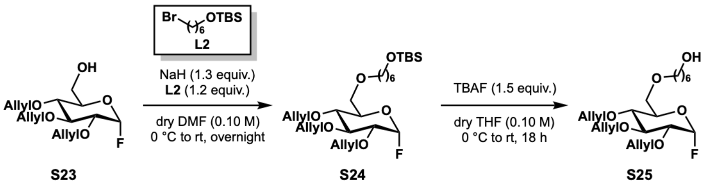

S23 **S24** 82% yield **S25** 74% yield

***tert*-butyldimethyl((6-(((2*R*,3*R*,4*S*,5*R*,6*R*)-3,4,5-tris(allyloxy)-6-fluorotetrahydro-2*H*-pyran-2-yl)methoxy)hexyl)oxy)silane (S24)**

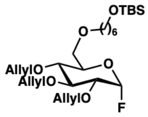

Chemical Formula: $C_{27}H_{49}FO_6Si$
Molecular Weight: 516.77

Compound **S24** was synthesized according to **GP4** using the α-fluoride **S23** (227 mg, 0.751 mmol, 1.0 equiv.) in 7 mL dry DMF, sodium hydride (39.0 mg, 0.976 mmol, 1.3 equiv.; 60% dispersed in mineral oil) and the bromide **L2** (266 mg, 0.901 mmol, 1.2 equiv.) in 7 mL dry THF was added. Silica gel column chromatography was performed (0-15% EtOAc in CyH) to yield the corresponding alkylated product **S24** (318 mg, 0.615 mmol, 82% yield) as colourless oil.

$\mathbf{R}_f$ 0.48 (9:1 cyclohexane: EtOAc)

^{1}H NMR (500 MHz, $CDCl_3$, 298K) δ 6.02 – 5.86 (m, 3H), 5.62 (dd, *J* = 53.3, 2.7 Hz, 1H), 5.31 (dq, *J* = 4.4, 1.6 Hz, 1H), 5.29 – 5.24 (m, 2H), 5.20 (dq, *J* = 10.3, 1.3 Hz, 1H), 5.17 (dq, *J* = 3.0, 1.3 Hz, 1H), 5.15 (dq, *J* = 3.0, 1.3 Hz, 1H), 4.38 – 4.26 (m, 3H), 4.23 (ddt, *J* = 12.7, 5.4, 1.4 Hz, 1H), 4.14 (dddt, *J* = 18.0, 12.3, 5.7, 1.4 Hz, 2H), 3.91 – 3.78 (m, 1H), 3.71 (t, *J* = 9.4 Hz, 1H), 3.68 – 3.56 (m, 4H), 3.56 – 3.47 (m, 2H), 3.45 – 3.32 (m, 2H), 1.67 – 1.56 (m, 2H), 1.51 (dq, *J* = 8.4, 6.6 Hz, 2H), 1.42 – 1.28 (m, 4H), 0.89 (s, 9H), 0.04 (s, 6H).

^{19}F NMR (470 MHz, $CDCl_3$, 298K) δ -149.44.

^{13}C NMR (126 MHz, $CDCl_3$, 298K) δ 135.2, 134.9, 134.6, 118.1, 117.0, 116.9, 105.9 (d, *J* = 226.4 Hz), 81.0, 79.1 (d, *J* = 24.6 Hz), 76.5, 74.5, 74.1, 72.9, 72.8 (d, *J* = 4.1 Hz), 71.9, 68.6, 63.3, 32.9, 29.7, 26.1, 26.1, 25.8, 18.5, -5.1.

HRMS (ESI^+) *m/z* calculated for $C_{27}H_{49}FNaO_6Si$ [M + Na^+]: 539.3175; found 539.3180.

IR ν_{max}(thin film)/cm^{-1} 2924m, 2853m, 1645w, 1459w, 1358w, 1252m, 1158s, 1080s, 992s, 921s, 833s, 772s, 660w.

6-(((2*R*,3*R*,4*S*,5*R*,6*R*)-3,4,5-tris(allyloxy)-6-fluorotetrahydro-2*H*-pyran-2-yl)methoxy)hexan-1-ol (S25)

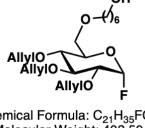

Chemical Formula: $C_{21}H_{35}FO_6$
Molecular Weight: 402.50

Compound **S25** was synthesized according to **GP5** using the compound **S24** (310 mg, 0.600 mmol, 1.0 equiv.) and TBAF (900 µL, 0.900 mmol, 1.5 equiv.; 1.0 M in THF) in 6 mL dry THF and stirred for 18 h. Silica gel column chromatography was performed (10-70% EtOAc in CyH) to yield the corresponding alkylated product **S25** (178 mg, 0.442 mmol, 74% yield) as colourless gummy oil.

R*f* 0.35 (1:1 cyclohexane: EtOAc)

^{1}H NMR (500 MHz, acetone-*d*$_6$, 298K) δ 6.02 – 5.89 (m, 3H), 5.67 (dd, *J* = 53.7, 2.7 Hz, 1H), 5.39 – 5.22 (m, 3H), 5.19 – 5.07 (m, 3H), 4.38 – 4.24 (m, 3H), 4.22 (dq, *J* = 5.5, 1.5 Hz, 2H), 4.16 (ddt, *J* = 12.7, 5.5, 1.5 Hz, 1H), 3.77 – 3.71 (m, 1H), 3.65 (dd, *J* = 11.0, 3.8 Hz, 1H), 3.63 – 3.56 (m, 2H), 3.56 – 3.48 (m, 3H), 3.48 – 3.41 (m, 2H), 3.41 – 3.32 (m, 2H), 1.63 – 1.54 (m, 2H), 1.54 – 1.48 (m, 2H), 1.44 – 1.32 (m, 4H).

^{19}F NMR (470 MHz, acetone-*d*$_6$, 298K) δ -150.25.

^{13}C NMR (126 MHz, acetone-*d*$_6$, 298K) δ 136.7, 136.4, 136.1, 116.9, 116.2, 116.0, 106.4 (d, *J* = 224.9 Hz), 81.6, 80.1 (d, *J* = 24.8 Hz), 77.4, 74.6, 74.3, 73.9 (d, *J* = 4.1 Hz), 72.6, 72.0, 69.6, 62.4, 33.8, 30.6, 26.9, 26.5.

HRMS (ESI$^+$) *m/z* calculated for $C_{21}H_{35}FNaO_6$ [M + Na$^+$]: 425.2310; found 425.2314.

IR ν_{max}(thin film)/cm^{-1} 3444br, 3072w, 2927s, 2868s, 2356m, 2337m, 1645w, 1457m, 1423m, 1349m, 1159s, 1106s, 1077s, 924s, 848s, 772m.

10.8. Synthesis of substrate S27

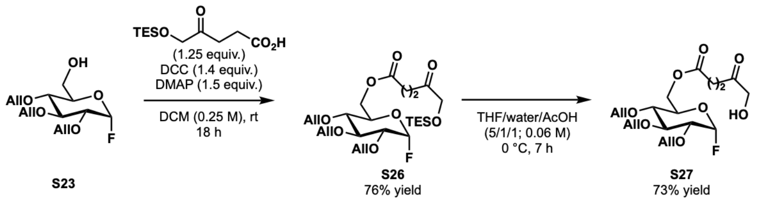

((2*R*,3*R*,4*S*,5*R*,6*R*)-6-fluoro-3,4,5-tris(((*E*)-prop-1-en-1-yl)oxy)tetrahydro-2*H*-pyran-2-yl)methyl 4-oxo-5-((triethylsilyl)oxy)pentanoate (S26)

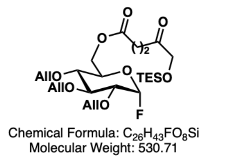

A suspension of D-glucose derivative **S23** (272 mg, 0.900 mmol, 1.0 equiv.), 4-oxo-5-((triethylsilyl)oxy)pentanoic acid[8] (278 mg, 1.13 mmol, 1.25 equiv.), dicyclohexylcarbodiimide (DCC; 260 mg, 1.26 mmol, 1.4 equiv.) and N,N-(di-methylamino) pyridine (DMAP; 165 mg, 1.35 mmol, 1.5 equiv.) in 4 mL dry dichloromethane was stirred at room temperature for 18 h. After completion of the reaction (monitored by TLC), the urea byproduct was removed by filtration, and the filtrate was evaporated. The crude material was purified by column chromatography (0-20% EtOAc in CyH) to yield the corresponding ester **S26** (363 mg, 0.684 mmol, 76%) as colourless gummy oil.

R*f* 0.40 (3:1 cyclohexane: EtOAc)

^{1}H NMR (500 MHz, $CDCl_3$, 298K) δ 6.17 – 5.81 (m, 3H), 5.59 (dd, *J* = 53.0, 2.7 Hz, 1H), 5.36 – 5.20 (m, 4H), 5.20 – 5.15 (m, 2H), 4.43 – 4.14 (m, 9H), 4.10 (ddt, *J* = 12.2, 6.1, 1.3 Hz, 1H), 3.97 – 3.90 (m, 1H), 3.73 (t, *J* = 9.4 Hz, 1H), 3.43 – 3.32 (m, 2H), 2.93 – 2.78 (m, 2H), 2.72 – 2.58 (m, 2H), 0.97 (t, *J* = 7.9 Hz, 9H), 0.63 (q, *J* = 8.0 Hz, 6H).

^{19}F NMR (470 MHz, $CDCl_3$, 298K) δ -149.35.

^{13}**C NMR** (126 MHz, $CDCl_3$, 298K) δ 209.2, 172.4, 134.9, 134.4, 134.3, 118.0, 117.5, 116.9, 105.4 (d, *J* = 227.4 Hz), 80.7, 78.9 (d, *J* = 24.6 Hz), 76.3, 74.4, 74.1, 72.7, 71.2 (d, *J* = 4.2 Hz), 68.9, 62.6, 33.1, 27.3, 6.7, 4.3.

HRMS (ESI^+) *m/z* calculated for $C_{26}H_{43}FNaO_8Si$ [M + Na^+]: 553.2603; found 553.2613.

IR ν_{max}(thin film)/cm^{-1} 2951w, 2872w, 1735m, 1645w, 1456w, 1412w, 1347w, 1152s, 1078s, 1002s, 923m, 811m, 729m.

((2*R*,3*R*,4*S*,5*R*,6*R*)-6-fluoro-3,4,5-tris(((*E*)-prop-1-en-1-yl)oxy)tetrahydro-2*H*-pyran-2-yl)methyl 5-hydroxy-4-oxopentanoate (S27)

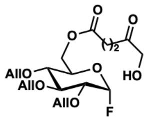

Chemical Formula: $C_{20}H_{29}FO_8$
Molecular Weight: 416.44

To a solution of ketoester **S26** (159 mg, 0.300 mmol, 1.0 equiv.) in 3.8 mL THF cooled to 0 °C, was added 0.8 mL water and 0.8 mL acetic acid. After stirring for 7 h at 0 °C (controlled by TLC), saturated NH_4Cl solution was added to stop the reaction and extracted with ethyl acetate (3x 25mL). The combined organic layer was extracted with brine (1x 30 mL) and dried over anhydrous magnesium sulfate, concentrated under reduced pressure. It was purified by column chromatography (20-70% EtOAc in CyH) to yield the corresponding alcohol **S27** (91.0 mg, 0.219 mmol, 73% yield) as colourless gummy oil.

$\mathbf{R}_f$ 0.50 (3:17 cyclohexane: EtOAc)

^{1}**H NMR** (500 MHz, acetone-d_6, 298K) δ 6.10 – 5.86 (m, 3H), 5.70 (dd, *J* = 53.4, 2.7 Hz, 1H), 5.51 – 5.23 (m, 3H), 5.21 – 4.96 (m, 3H), 4.35 (dddt, *J* = 12.6, 8.5, 5.5, 1.6 Hz, 2H), 4.30 – 4.25 (m, 3H), 4.23 (ddt, *J* = 5.3, 2.7, 1.5 Hz, 4H), 4.15 (ddt, *J* = 12.6, 5.8, 1.5 Hz, 1H), 3.90 (t, *J* = 5.5 Hz, 1H), 3.86 (dddd, *J* = 10.2, 4.2, 2.5, 0.6 Hz, 1H), 3.63 (t, *J* = 9.3 Hz, 1H), 3.54 – 3.35 (m, 2H), 2.80 (ddt, *J* = 7.0, 5.0, 0.6 Hz, 2H), 2.66 (td, *J* = 6.4, 1.4 Hz, 2H).

^{19}**F NMR** (470 MHz, acetone-d_6, 298K) δ -150.22.

^{13}**C NMR** (126 MHz, $CDCl_3$, 298K) δ 208.0, 172.0, 135.0, 134.5, 134.4, 118.2, 117.7, 117.1, 105.5 (d, *J* = 227.5 Hz), 80.8, 79.0 (d, *J* = 24.6 Hz), 76.4, 74.6, 74.2, 72.9, 71.2 (d, *J* = 4.4 Hz), 68.4, 63.1, 32.9, 27.8.

HRMS (ESI^+) *m/z* calculated for $C_{20}H_{29}FNaO_8$ [M + Na^+]: 439.1739; found 439.1737.

IR ν_{max}(thin film)/cm^{-1} 3460br, 2915w, 1723m, 1645w, 1409w, 1351m, 1152s, 1071s, 993s, 922s, 768m, 510m.

10.9. Synthesis of dimeric compound 10

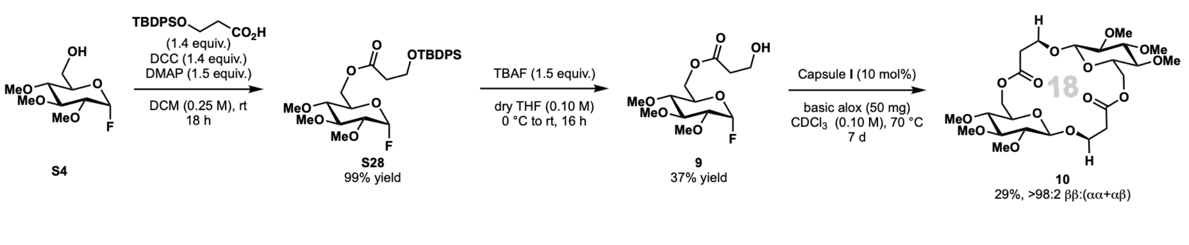

((2*R*,3*R*,4*S*,5*R*,6*R*)-6-fluoro-3,4,5-trimethoxytetrahydro-2*H*-pyran-2-yl)methyl 3-((*tert*-butyldiphenylsilyl)oxy)propanoate (S28)

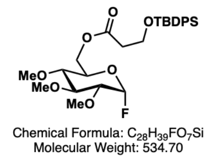

A suspension of D-glucose derivative **S4** (344 mg, 1.53 mmol, 1.0 equiv.), 3-((*tert*-butyldiphenylsilyl)oxy)propanoic acid[9] (703 mg, 2.14 mmol, 1.4 equiv.), dicyclohexylcarbodiimide (DCC; 442 mg, 2.14 mmol, 1.4 equiv.) and N,N-(di-methylamino) pyridine (DMAP; 281 mg, 2.3 mmol, 1.5 equiv.) in 4 mL dry dichloromethane was stirred at room temperature for 18 h. After completion of the reaction (monitored by TLC), the urea byproduct was removed by filtration, and the filtrate was evaporated. The crude material was purified by column chromatography (0-15% EtOAc in CyH) to yield the corresponding ester **S28** (810 mg, 1.51 mmol, 99% yield) as colourless gummy oil.

R*f* 0.28 (4:1 cyclohexane: EtOAc)

^{1}H NMR (500 MHz, $CDCl_3$, 298K) δ 7.70 – 7.63 (m, 4H), 7.46 – 7.35 (m, 6H), 5.62 (dd, *J* = 53.0, 2.7 Hz, 1H), 4.38 (dd, *J* = 12.1, 2.1 Hz, 1H), 4.29 (dd, *J* = 12.1, 4.6 Hz, 1H), 3.96 (td, *J* = 6.3, 2.5 Hz, 2H), 3.90 (ddd, *J* = 10.2, 4.7, 2.2 Hz, 1H), 3.65 (s, 3H), 3.55-3.50 (m, 7H), 3.25 – 3.08 (m, 2H), 2.61 (t, *J* = 6.3 Hz, 2H), 1.03 (s, 9H).

^{19}F NMR (470 MHz, $CDCl_3$, 298K) δ -149.33.

^{13}C NMR (126 MHz, $CDCl_3$, 298K) δ 171.5, 135.7, 135.7, 133.6, 133.6, 129.9, 129.9, 127.8, 104.8 (d, *J* = 227.1 Hz), 83.0, 81.5 (d, *J* = 24.7 Hz), 78.9, 71.3 (d, *J* = 4.4 Hz), 62.7, 61.2, 60.9, 59.9, 59.4, 37.8, 26.9, 19.3 (one aromatic carbon signal is missing due to overlap with other signals).

HRMS (ESI^+) *m/z* calculated for $C_{28}H_{39}FNaO_7Si$ [M + Na^+]: 557.2341; found 557.2335.

IR ν_{max}(thin film)/cm^{-1} 2927m, 2852m, 1739s, 1469m, 1425m, 1387m, 1360m, 1262m, 1156s, 1099s, 1016s, 938m, 891m, 821m, 737s, 700s, 686s, 611s.

((2*R*,3*R*,4*S*,5*R*,6*R*)-6-fluoro-3,4,5-trimethoxytetrahydro-2*H*-pyran-2-yl)methyl 3-hydroxypropanoate (9)

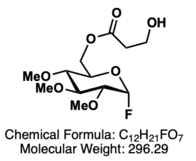

Compound **9** was synthesized according to **GP5** using the compound **S28** (603 mg, 1.13 mmol, 1.0 equiv.) and TBAF (1.70 mL, 1.70 mmol, 1.5 equiv.; 1.0 M in THF) in 12 mL dry THF and stirred for 18 h. Silica gel column chromatography (20-80% EtOAc in CyH) to yield the corresponding alcohol **9** (124 mg, 0.419 mmol, 37% yield) as colourless gummy oil.

R*f* 0.22 (3:7 cyclohexane: EtOAc)

^{1}H NMR (500 MHz, acetone-*d*$_6$, 298K) δ 5.72 (dd, *J* = 53.4, 2.7 Hz, 1H), 4.46 – 4.22 (m, 2H), 3.86 – 3.77 (m, 3H), 3.74 (t, *J* = 5.5 Hz, 1H), 3.57 (s, 3H), 3.51 (s, 3H), 3.50 (s, 3H), 3.38 (t, *J* = 9.3 Hz, 1H), 3.30 – 3.14 (m, 2H), 2.55 (td, *J* = 6.1, 2.1 Hz, 2H).

^{19}F NMR (470 MHz, acetone-*d*$_6$, 298K) δ -150.41.

^{13}C NMR (126 MHz, acetone-d_6, 298K) δ 172.1, 105.7 (d, J = 225.4 Hz), 83.8, 82.0 (d, J = 24.6 Hz), 79.3, 72.1 (d, J = 4.4 Hz), 63.1, 60.9, 60.8, 58.9, 58.5, 38.4.

HRMS (ESI$^+$) m/z calculated for $C_{12}H_{21}FNaO_6$ [M + Na$^+$]: 319.1164; found 319.1159.

IR ν_{max}(thin film)/cm^{-1} 3450br, 2933m, 2831w, 1732s, 1446m, 1364m, 1151s, 1088s, 1001s, 889m, 756m.

(1*R*,8*R*,9*R*,10*S*,11*R*,12*R*,19*R*,20*R*,21*S*,22*R*)-9,10,11,20,21,22-hexamethoxy-2,6,13,17,23,24-hexaoxatricyclo[17.3.1.1^{8,12}]tetracosane-5,16-dione (10)

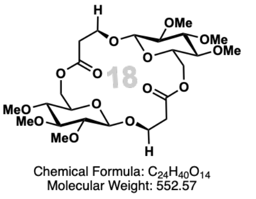

Compound **10** was synthesized using the **GP1** and substrate **9** (13.3 mg, 44.9 µmol, 1.0 equiv.) in $CDCl_3$ at 70 °C for 7 d. The crude material was dissolved in 2 mL of acetonitrile and kept at -20 °C for 15 minutes. Then, the white precipitate was filtered off and washed with cold acetonitrile. The filtrate was evaporated and purified using silica gel column chromatography (10-90% EtOAc in CyH) to yield **10** as a mixture with some catalyst. Finally, compound **10** was purified by preparative TLC using 90% EtOAc in CyH as a white solid (3.60 mg, 6.52 µmol, 29% yield).

R*f*: 0.44 (3:17 cyclohexane/ethyl acetate).

mp: 204 – 207 °C

^{1}H NMR (500 MHz, acetone-d_6, 298K) δ 4.41 (dd, J = 11.4, 2.2 Hz, 2H), 4.32 (d, J = 7.8 Hz, 2H), 4.13 – 3.97 (m, 4H), 3.84 (td, J = 10.2, 6.0 Hz, 2H), 3.55 (s, 6H), 3.49 (s, 12H), 3.45 (td, J = 9.5, 2.3 Hz, 2H), 3.14 (t, J = 8.9 Hz, 2H), 2.95 (dd, J = 9.9, 8.7 Hz, 2H), 2.88 (dd, J = 9.0, 7.8 Hz, 2H), 2.77 – 2.64 (m, 4H).

^{13}C NMR (126 MHz, acetone-d_6, 298K) δ 170.4, 103.9, 87.2, 84.7, 81.3, 73.4, 65.8, 64.5, 60.8, 60.5, 60.4, 36.1.

HRMS (ESI$^+$) m/z calculated for $C_{24}H_{40}NaO_{14}$ [M + Na$^+$]: 575.2310; found 575.2320.

IR ν_{max}(thin film)/cm^{-1} 2921w, 2834w, 1734s, 1465w, 1372w, 1345w, 1286m, 1255m, 1176m, 1155m, 1117s, 1086s, 1067s, 1037s, 1019s, 996s, 980s, 946s, 916s, 894s, 797m, 722m, 616m.

11. Occupancy estimations

The volume of capsule I (1375 Å^3) was reported in the literature.[10] The volumes of the guest molecules were calculated using the software Spartan'24, Wavefunction Inc.. The initial model of the respective guest molecule was geometry optimized using the semi-empirical PM6 method (gas phase). The volumes obtained are listed in the table below.

Entry	Guest molecule	Volume (Å^3)
1	dichloromethane (DCM/CH_2Cl_2)	60.99
2	chloroform ($CHCl_3$)	74.55
3	1,2-dichlorobenzene (1,2-DCB)	126.1
4	Substrate **1a**	301.6

5	Substrate **1b**	320.1
6	Substrate **1c**	338.0
7	Substrate **1d**	375.4
8	Substrate **1e**	412.3
9	Substrate **1f**	481.7
10	Substrate **1g**	630.1

12. References

(1) Köster, J. M.; Tiefenbacher, K. Elucidating the Importance of Hydrochloric Acid as a Cocatalyst for Resorcinarene-Capsule-Catalyzed Reactions. *ChemCatChem* **2018**, *10* (14), 2941–2944. https://doi.org/https://doi.org/10.1002/cctc.201800326.

(2) Pelletier, G.; Zwicker, A.; Allen, C. L.; Schepartz, A.; Miller, S. J. Aqueous Glycosylation of Unprotected Sucrose Employing Glycosyl Fluorides in the Presence of Calcium Ion and Trimethylamine. *J Am Chem Soc* **2016**, *138* (9), 3175–3182. https://doi.org/10.1021/jacs.5b13384.

(3) Łopatkiewicz, G.; Buda, S.; Mlynarski, J. Application of the EF and GH Fragments to the Synthesis of Idraparinux. *J Org Chem* **2017**, *82* (23), 12701–12714. https://doi.org/10.1021/acs.joc.7b02497.

(4) Lethu, S.; Matsuoka, S.; Murata, M. Highly Efficient Preparation of Selectively Isotope Cluster-Labeled Long Chain Fatty Acids via Two Consecutive Csp3–Csp3 Cross-Coupling Reactions. *Org Lett* **2014**, *16* (3), 844–847. https://doi.org/10.1021/ol4036159.

(5) Köster, J. M.; Tiefenbacher, K. Elucidating the Importance of Hydrochloric Acid as a Cocatalyst for Resorcinarene-Capsule-Catalyzed Reactions. *ChemCatChem* **2018**, *10* (14), 2941–2944. https://doi.org/https://doi.org/10.1002/cctc.201800326.

(6) López, J. C.; Bernal-Albert, P.; Uriel, C.; Valverde, S.; Gómez, A. M. IPy2BF4/HF-Pyridine: A New Combination of Reagents for the Transformation of Partially Unprotected Thioglycosides and n-Pentenyl Glycosides to Glycosyl Fluorides. *J Org Chem* **2007**, *72* (26), 10268–10271. https://doi.org/10.1021/jo7018653.

(7) Lusseau, J.; Uduagwu, D.; Robert, F.; Landais, Y. Copper-Mediated Decarboxylative Amidination: Synthesis of O-Methyl-N-Alkylnitroisoureas. *Adv Synth Catal* **2024**, *366* (10), 2209–2213. https://doi.org/https://doi.org/10.1002/adsc.202400091.

(8) Groß, T.; Herrmann, T.; Shi, B.; Jäger, A.; Chiu, P.; Metz, P. Further Studies on Sultones Derived from Carbene Cyclization Cycloaddition Cascades. *Tetrahedron* **2015**, *71* (35), 5925–5931. https://doi.org/https://doi.org/10.1016/j.tet.2015.05.095.

(9) Sano, S.; Kuroda, Y.; Saito, K.; Ose, Y.; Nagao, Y. Tandem Reduction–Olefination of Triethyl 2-Acyl-2-Fluoro-2-Phosphonoacetates and a Synthetic Approach to Cbz-Gly-Ψ[(Z)-CFC]-Gly Dipeptide Isostere. *Tetrahedron* **2006**, *62* (51), 11881–11890. https://doi.org/https://doi.org/10.1016/j.tet.2006.09.096.

(10) MacGillivray, L. R.; Atwood, J. L. A Chiral Spherical Molecular Assembly Held Together by 60 Hydrogen Bonds. *Nature* **1997**, *389* (6650), 469–472. https://doi.org/10.1038/38985.

Appendix A: Crystallographic details

Crystal Data and Experimental

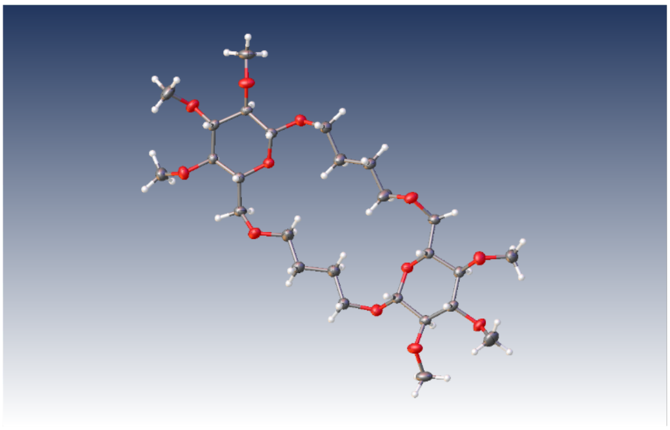

Experimental. Single colourless needle-shaped crystals of sug-157_150k_new were used as supplied. A suitable crystal with dimensions 0.30 × 0.13 × 0.04 mm³ was selected and mounted on a STOE STADIVARI diffractometer. The crystal was kept at a steady T = 150 K during data collection. The structure was solved with the ShelXT 2018/2 (Sheldrick, 2018) solution program using dual methods and by using Olex2 1.5 (Dolomanov et al., 2009) as the graphical interface. The model was refined with ShelXL 2018/3 (Sheldrick, 2015) using full matrix least squares minimisation on $\boldsymbol{F}^2$.

Crystal Data. $C_{26}H_{48}O_{12}$, M_r = 552.64, monoclinic, $C2$ (No. 5), a = 17.4489(9) Å, b = 4.32420(10) Å, c = 20.8071(11) Å, β = 113.940(4)°, $\alpha = \gamma$ = 90°, V = 1434.89(12) Å³, T = 150 K, Z = 2, Z' = 0.5, $\mu(GaK_\alpha)$ = 0.535, 9968 reflections measured, 2163 unique (R_{int} = 0.0497) which were used in all calculations. The final wR_2 was 0.1368 (all data) and R_1 was 0.0489 (I≥2 σ(I)).

**Compound**	sug-157_150k_new (**3a**)
Formula	$C_{26}H_{48}O_{12}$
$D_{calc.}$/ g cm^{-3}	1.279
μ/mm^{-1}	0.535
Formula Weight	552.64
Colour	colourless
Shape	needle-shaped
Size/mm³	0.30×0.13×0.04
T/K	150
Crystal System	monoclinic
Flack Parameter	0.0(4)
Hooft Parameter	-0.45(15)
Space Group	$C2$
a/Å	17.4489(9)
b/Å	4.32420(10)
c/Å	20.8071(11)
α/°	90
β/°	113.940(4)
γ/°	90
V/Å³	1434.89(12)
Z	2
Z'	0.5
Wavelength/Å	1.34143
Radiation type	GaK_α
Θ_{min}/°	4.410
Θ_{max}/°	55.880
Measured Refl's.	9968
Indep't Refl's	2163
Refl's I≥2 σ(I)	1991
R_{int}	0.0497
Parameters	175
Restraints	1
Largest Peak	0.296
Deepest Hole	-0.227
GooF	1.056
wR_2 (all data)	0.1368
wR_2	0.1315
R_1 (all data)	0.0537
R_1	0.0489

Structure Quality Indicators

**Reflections:**	d min (GaKα) 2Θ=111.8° 0.81	I/σ(I) 29.9	Rint m=4.61 4.97%	Full 107.2° 99% to 111.8° 99.8	
**Refinement:**	Shift 0.000	Max Peak 0.3	Min Peak -0.2	GooF 1.056	Hooft -.45(15)

A colourless needle-shaped-shaped crystal with dimensions 0.30 × 0.13 × 0.04 mm^3 was mounted. Data were collected using a STOE STADIVARI diffractometer equipped with an Oxford Cryosystems low-temperature device operating at T = 150 K.

Data were measured using rotation method, ω scans with GaK_{α} radiation. The diffraction pattern was indexed and the total number of runs and images was based on the strategy calculation from the program X-Area Pilatus3_SV 1.31.186.0 (STOE, 2022). The maximum resolution that was achieved was Θ = 55.880° (0.81 Å).

The unit cell was refined using X-Area Integrate 2.5.3.0 (STOE, 2021)X-Area LANA 2.7.5.0 (STOE, 2022) on 8497 reflections, 85% of the observed reflections.

Data reduction, scaling and absorption corrections were performed using X-Area Integrate 2.5.3.0 (STOE, 2021)X-Area LANA 2.7.5.0 (STOE, 2022). The final completeness is 99.80 % out to 55.880° in Θ. A multi-scan absorption correction was performed using STOE X-Red32, absorption correction by Gaussian integration, analogous to P. Coppens in: F. R. Ahmed (Editor), "Crystallographic Computing", Munksgaard, Copenhagen (1970), 255 - 270. Afterwards scaling of reflection intensities was performed within STOE LANA. J. Koziskova, F. Hahn, J. Richter, J. Kozisek, Acta Chimica Slovaca, vol. 9, no. 2, 2016, pp. 136 - 140. Finally a spherical absorption correction was done within STOE LANA.. The absorption coefficient μ of this material is 0.535 mm^{-1} at this wavelength (λ = 1.34143Å) and the minimum and maximum transmissions are 0.548 and 0.749.

The structure was solved and the space group C2 (# 5) determined by the ShelXT 2018/2 (Sheldrick, 2018) structure solution program using using dual methods and refined by full matrix least squares minimisation on $\boldsymbol{F}^2$ using version 2018/3 of ShelXL 2018/3 (Sheldrick, 2015). All non-hydrogen atoms were refined anisotropically. Hydrogen atom positions were calculated geometrically and refined using the riding model. Hydrogen atom positions were calculated geometrically and refined using the riding model.

_exptl_absorpt_process_details: STOE X-Red32, absorption correction by Gaussian integration, analogous toP. Coppens in: F. R. Ahmed (Editor), "Crystallographic Computing", Munksgaard,Copenhagen (1970), 255 - 270. Afterwards scaling of reflection intensities wasperformed within STOE LANA. J. Koziskova, F. Hahn, J. Richter, J. Kozisek, ActaChimica Slovaca, vol. 9, no. 2, 2016, pp. 136 - 140.Finally a spherical absorption correction was done within STOE LANA.

The value of Z' is 0.5. This means that only half of the formula unit is present in the asymmetric unit, with the other half consisting of symmetry equivalent atoms. The moiety formula is C26 H48 O12.

The Flack parameter was refined to 0.0(4). Determination of absolute structure using Bayesian statistics on Bijvoet differences using the Olex2 results in -0.45(15). Note: The Flack parameter is used to determine chirality of the crystal studied, the value should be near 0, a value of 1 means that the stereochemistry is wrong and the model should be inverted. A value of 0.5 means that the crystal consists of a racemic mixture of the two enantiomers.

Data Plots: Diffraction Data

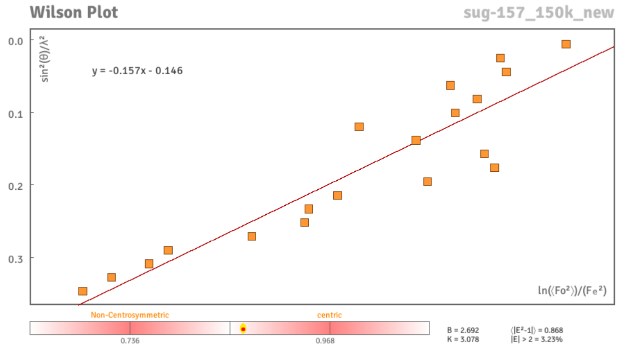

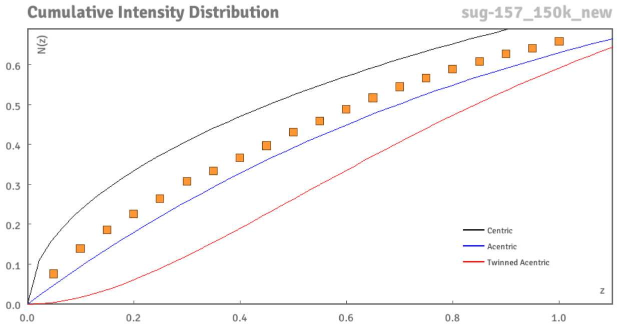

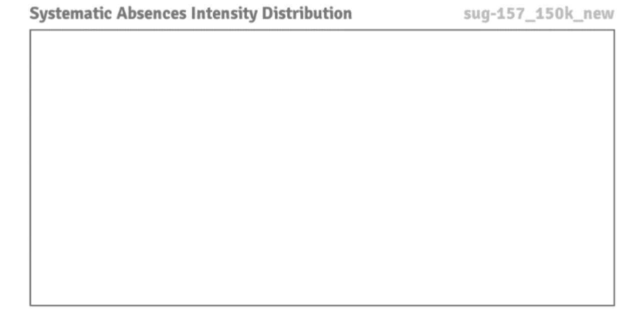

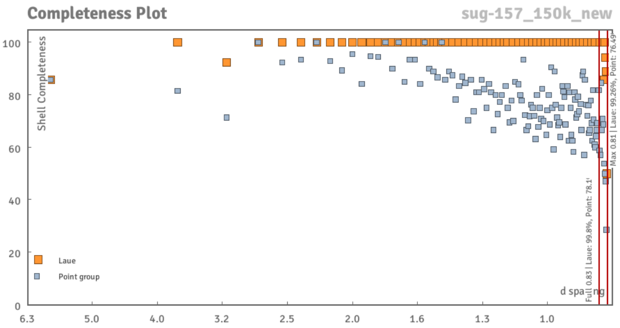

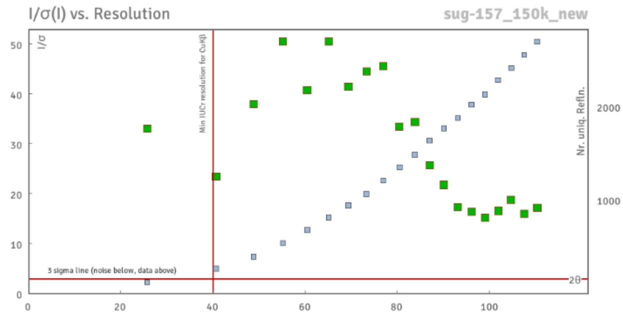

Data Plots: Refinement and Data

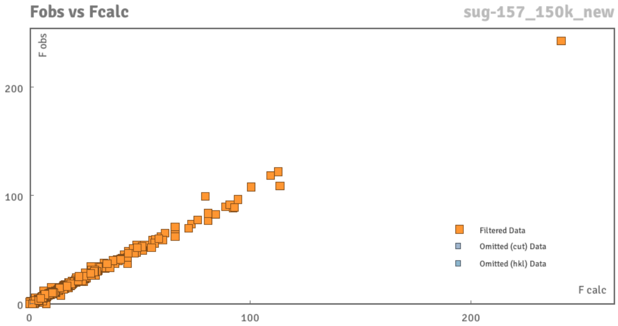

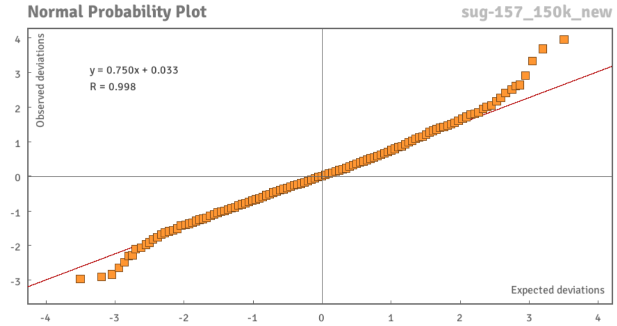

Reflection Statistics

Total reflections (after filtering)	9968	Unique reflections	2163
Completeness	0.765	Mean I/σ	30.79
hkl$_{max}$ collected	(21, 3, 23)	hkl$_{min}$ collected	(-21, -5, -25)
hkl$_{max}$ used	(19, 3, 25)	hkl$_{min}$ used	(-21, -5, 0)
Lim d$_{max}$ collected	100.0	Lim d$_{min}$ collected	0.67
d$_{max}$ used	8.72	d$_{min}$ used	0.81
Friedel pairs	683	Friedel pairs merged	0

Inconsistent equivalents	259	R_{int}	0.0497
R_{sigma}	0.0334	Intensity transformed	0
Omitted reflections	0	Omitted by user (OMIT hkl)	0
Multiplicity	(772, 971, 637, 457, 294, 185, 77, 37, 10, 1)	Maximum multiplicity	17
Removed systematic absences	0	Filtered off (Shel/OMIT)	0

Table 1: Fractional Atomic Coordinates (×10^4) and Equivalent Isotropic Displacement Parameters (Å^2×10^3) for sug-157_150k_new. U_{eq} is defined as 1/3 of the trace of the orthogonalised U_{ij}.

Atom	x	y	z	U_{eq}
O3	4733.3(10)	6414(4)	3101.1(8)	26.9(4)
O2	3315.4(11)	6624(5)	2771.9(10)	32.1(5)
O1	3514.8(12)	4604(5)	5764.3(9)	34.1(5)
O4	6042.2(12)	4161(6)	2189.0(10)	34.6(5)
O5	4425.4(12)	5731(5)	1052.6(9)	35.6(5)
O6	3073.8(12)	3868(5)	1457.2(10)	37.0(5)
C9	5374.2(15)	5709(6)	2279.9(12)	25.9(5)
C8	4535.1(16)	4614(6)	1728.5(12)	26.4(6)
C6	3945.9(14)	5077(7)	2649.3(12)	26.1(5)
C7	3790.6(15)	5621(6)	1882.8(12)	25.9(5)
C10	5424.1(14)	4960(6)	3013.8(12)	25.5(5)
C1	3762.3(16)	6127(7)	6419.3(13)	30.7(6)
C5	3075.5(16)	5279(8)	3293.6(13)	34.2(6)
C3	3309.5(17)	4991(8)	4564.9(13)	35.1(6)
C4	3662.7(17)	5999(9)	4041.8(14)	39.3(7)
C2	3872.5(19)	5755(11)	5311.0(14)	46.6(9)
C13	2478.7(18)	5457(10)	884.6(15)	44.9(8)
C11	6450.8(18)	5942(10)	1842.9(15)	44.6(8)
C12	4359(3)	3398(11)	558.3(16)	53.2(10)

Table 2: Anisotropic Displacement Parameters (×10^4) for sug-157_150k_new. The anisotropic displacement factor exponent takes the form: $-2\pi^2[h^2a^{*2} \times U_{11} + ... +2hka^* \times b^* \times U_{12}]$

Atom	U_{11}	U_{22}	U_{33}	U_{23}	U_{13}	U_{12}
O3	22.8(8)	33.4(10)	22.8(8)	-1.9(8)	7.6(7)	1.5(8)
O2	25.2(8)	43.5(11)	28.1(8)	4.5(9)	11.2(7)	4.6(8)
O1	33.0(9)	42.7(12)	22.9(8)	-2.0(8)	7.6(7)	-7.8(8)
O4	31.4(10)	42.2(12)	35.2(9)	1.0(9)	18.5(8)	6.6(9)
O5	42.8(10)	43.3(12)	19.9(8)	3.1(8)	11.8(7)	2.3(10)
O6	29.6(9)	39.5(11)	30.2(9)	1.8(9)	0.1(7)	-6.1(9)
C9	25.7(11)	28.1(13)	24.3(11)	0.2(10)	10.7(9)	2.8(10)
C8	31.7(12)	26.0(13)	19.3(10)	0.5(10)	8.2(9)	1.2(10)
C6	21.8(11)	30.3(12)	24.0(11)	3.5(10)	7.2(9)	2.3(10)
C7	24.4(11)	27.9(13)	21.0(11)	1.4(10)	4.6(9)	0.9(10)
C10	23.5(11)	27.9(12)	24.4(11)	1.0(10)	8.9(9)	2.0(10)
C1	24.6(11)	40.0(14)	24.0(11)	-0.6(12)	6.2(9)	-1.4(12)
C5	27.3(11)	49.7(16)	26.2(11)	0.5(12)	11.6(10)	-2.4(13)
C3	29.6(12)	46.8(16)	26.2(12)	-3.7(12)	8.7(10)	-4.0(12)
C4	31.0(12)	57.2(19)	28.9(12)	-4.0(14)	11.2(10)	-7.8(14)
C2	36.2(14)	76(3)	27.2(13)	-5.3(16)	12.1(11)	-20.0(17)
C13	29.3(13)	59(2)	32.4(13)	0.7(15)	-1.8(11)	4.5(15)
C11	31.9(13)	71(2)	34.4(13)	-1.2(16)	17.2(11)	-0.4(16)
C12	63(2)	70(3)	27.1(14)	-10.7(16)	19.2(14)	-6(2)

Table 1: Bond Lengths in Å for sug-157_150k_new.

Atom	Atom	Length/Å
O3	C6	1.435(3)
O3	C10	1.435(3)
O2	C6	1.397(3)
O2	C5	1.436(3)
O1	C1	1.414(3)
O1	C2	1.416(4)
O4	C9	1.422(3)
O4	C11	1.427(4)
O5	C8	1.425(3)
O5	C12	1.411(4)
O6	C7	1.423(3)
O6	C13	1.402(4)
C9	C8	1.523(3)
C9	C10	1.529(3)
C8	C7	1.523(4)
C6	C7	1.524(3)
C10	C1[1]	1.517(3)
C5	C4	1.509(4)
C3	C4	1.516(4)
C3	C2	1.499(4)

----
[1]1-x,+y,1-z

Table 3: Bond Angles in ° for sug-157_150k_new.

Atom	Atom	Atom	Angle/°
C6	O3	C10	111.77(18)
C6	O2	C5	115.9(2)
C1	O1	C2	115.7(2)
C9	O4	C11	114.7(3)
C12	O5	C8	114.5(3)
C13	O6	C7	115.4(2)
O4	C9	C8	110.0(2)
O4	C9	C10	108.40(19)
C8	C9	C10	109.5(2)
O5	C8	C9	109.9(2)
O5	C8	C7	109.1(2)
C7	C8	C9	113.2(2)
O3	C6	C7	109.9(2)
O2	C6	O3	107.6(2)
O2	C6	C7	107.37(19)
O6	C7	C8	109.0(2)
O6	C7	C6	107.9(2)
C8	C7	C6	111.83(19)
O3	C10	C9	108.87(19)
O3	C10	C1[1]	109.0(2)
C1[1]	C10	C9	111.2(2)
O1	C1	C10[1]	114.7(2)
O2	C5	C4	114.3(2)
C2	C3	C4	113.2(2)
C5	C4	C3	112.2(2)
O1	C2	C3	109.4(2)

----
[1]1-x,+y,1-z

Table 4: Torsion Angles in ° for sug-157_150k_new.

Atom	Atom	Atom	Atom	Angle/°
O3	C6	C7	O6	171.3(2)
O3	C6	C7	C8	51.4(3)
O2	C6	C7	O6	-72.0(3)
O2	C6	C7	C8	168.1(2)
O2	C5	C4	C3	170.4(3)
O4	C9	C8	O5	-70.2(3)
O4	C9	C8	C7	167.6(2)
O4	C9	C10	O3	-177.8(2)
O4	C9	C10	C1[1]	62.1(3)
O5	C8	C7	O6	72.2(3)
O5	C8	C7	C6	-168.5(2)
C9	C8	C7	O6	-165.0(2)
C9	C8	C7	C6	-45.8(3)
C8	C9	C10	O3	-57.7(3)
C8	C9	C10	C1[1]	-177.8(2)
C6	O3	C10	C9	67.2(3)
C6	O3	C10	C1[1]	-171.3(2)
C6	O2	C5	C4	79.6(3)
C10	O3	C6	O2	179.85(19)
C10	O3	C6	C7	-63.5(3)
C10	C9	C8	O5	170.8(2)
C10	C9	C8	C7	48.5(3)
C1	O1	C2	C3	-155.3(3)
C5	O2	C6	O3	-92.1(3)
C5	O2	C6	C7	149.7(2)

Atom	Atom	Atom	Atom	Angle/°
C4	C3	C2	O1	-177.2(3)
C2	O1	C1	C10[1]	-84.9(3)
C2	C3	C4	C5	-178.5(3)
C13	O6	C7	C8	-105.1(3)
C13	O6	C7	C6	133.3(2)
C11	O4	C9	C8	101.4(3)
C11	O4	C9	C10	-138.8(2)
C12	O5	C8	C9	117.8(3)
C12	O5	C8	C7	-117.5(3)

––––

[1]1-x,+y,1-z

Table 5: Hydrogen Fractional Atomic Coordinates (×10^4) and Equivalent Isotropic Displacement Parameters (Å^2×10^3) for sug-157_150k_new. U_{eq} is defined as 1/3 of the trace of the orthogonalised U_{ij}.

Atom	x	y	z	U_{eq}
H9	5429.71	7991.62	2234.1	31
H8	4542.26	2302.98	1716.31	32
H6	3936.03	2815.84	2746.4	31
H7	3677.67	7867.4	1769.35	31
H10	5387.07	2670.4	3061.73	31
H1A	3310.42	5893.82	6588.14	37
H1B	3822.53	8362.14	6347.48	37
H5A	3043.32	3006.04	3230.51	41
H5B	2507.59	6025.13	3213.84	41
H3A	2760.06	6007.3	4444.47	42
H3B	3213.93	2729.26	4523.37	42
H4A	3770.94	8253.25	4089.12	47
H4B	4204.39	4939.51	4152.07	47
H2A	4431.4	4814.01	5433.26	56
H2B	3944.12	8024.46	5366.95	56
H13A	2729.1	6051.48	557.78	67
H13B	2296.91	7315.8	1053.2	67
H13C	1994.25	4113.05	642.46	67
H11A	6093.41	6044.71	1338.79	67
H11B	6985.99	4966.41	1913.28	67
H11C	6553.73	8037.3	2039.11	67
H12A	4262.54	4349.07	104.53	80
H12B	3887.85	2031.76	503.48	80
H12C	4878.7	2192.57	723.68	80

Appendix B: NMR spectra of substrates

^{1}H NMR (500 MHz, acetone-d_6, 298K) of compound **3a**

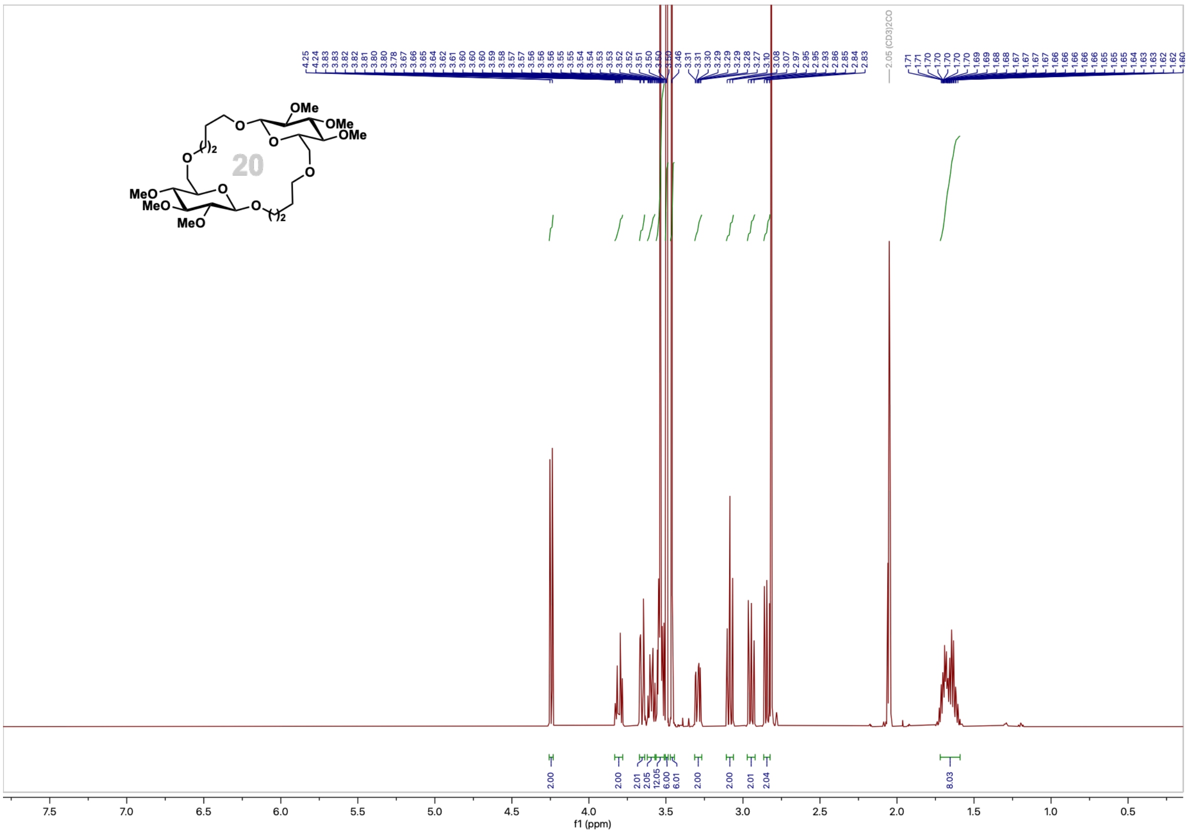

^{13}C NMR (126 MHz, acetone-d_6, 298K) of compound **3a**

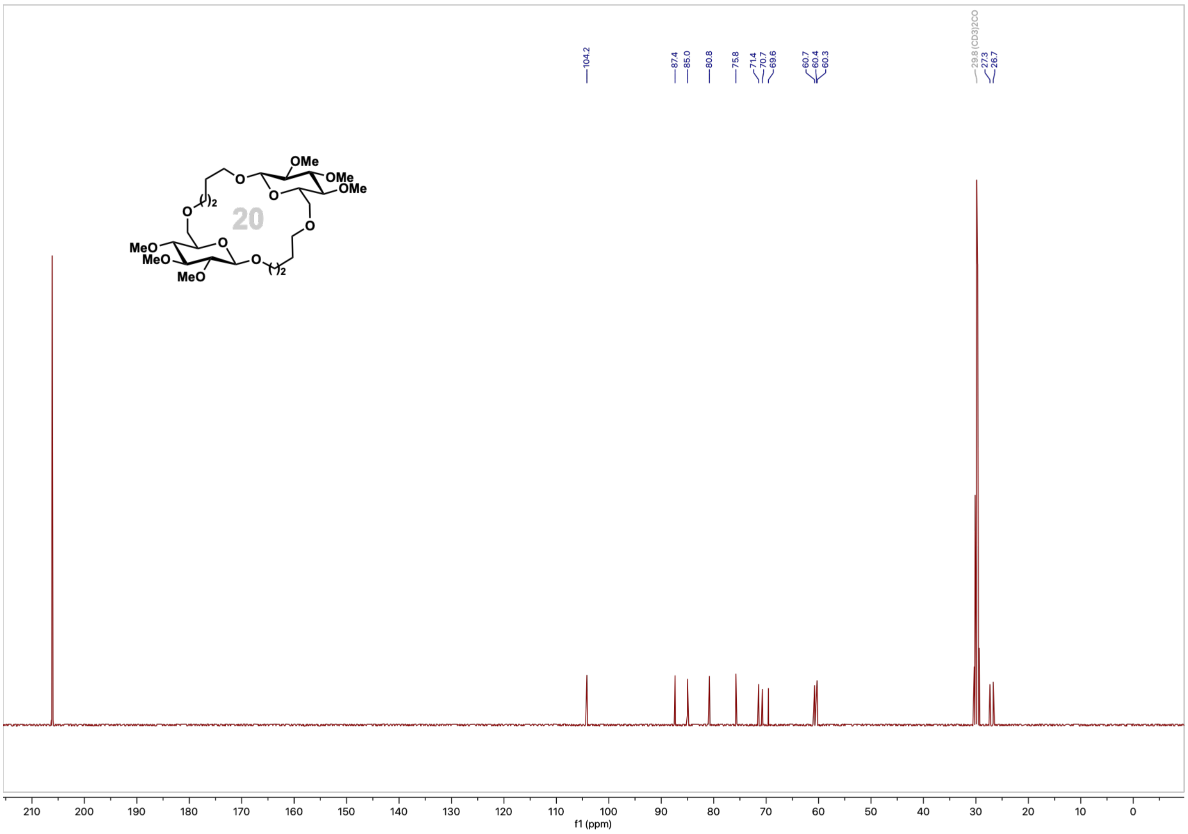

^{1}H NMR (500 MHz, acetone-d_6, 298K) of compound **2b**

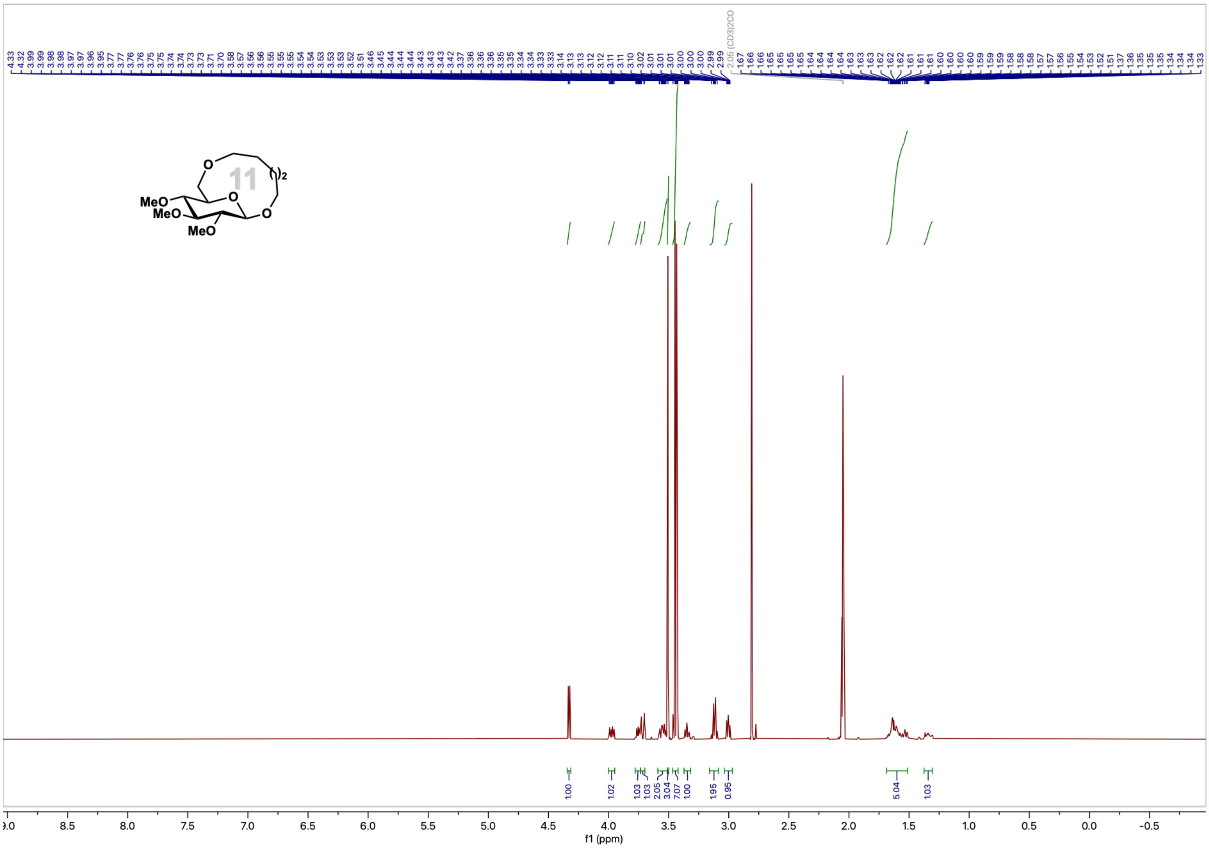

^{13}C NMR (126 MHz, acetone-d_6, 298K) of compound **2b**

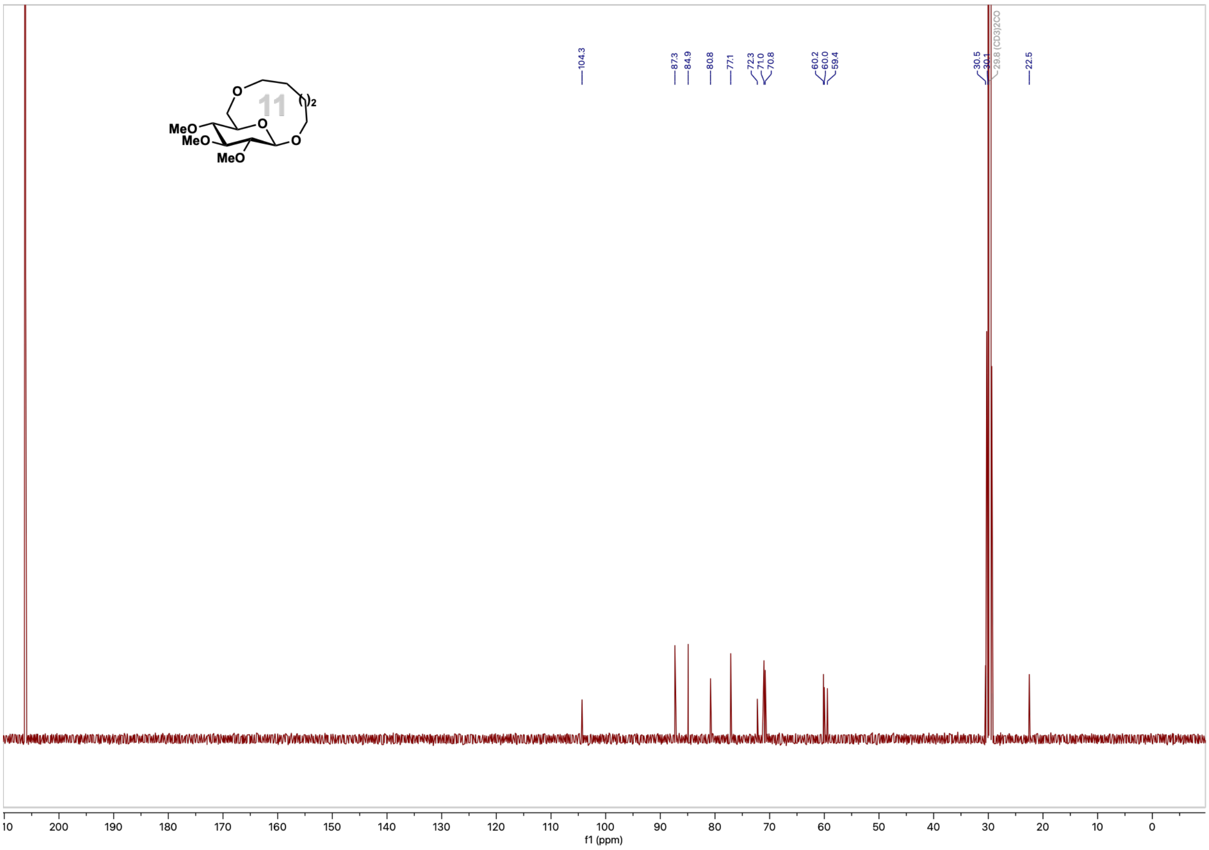

^{1}H NMR (500 MHz, acetone-d_6, 298K) of compound **3b**

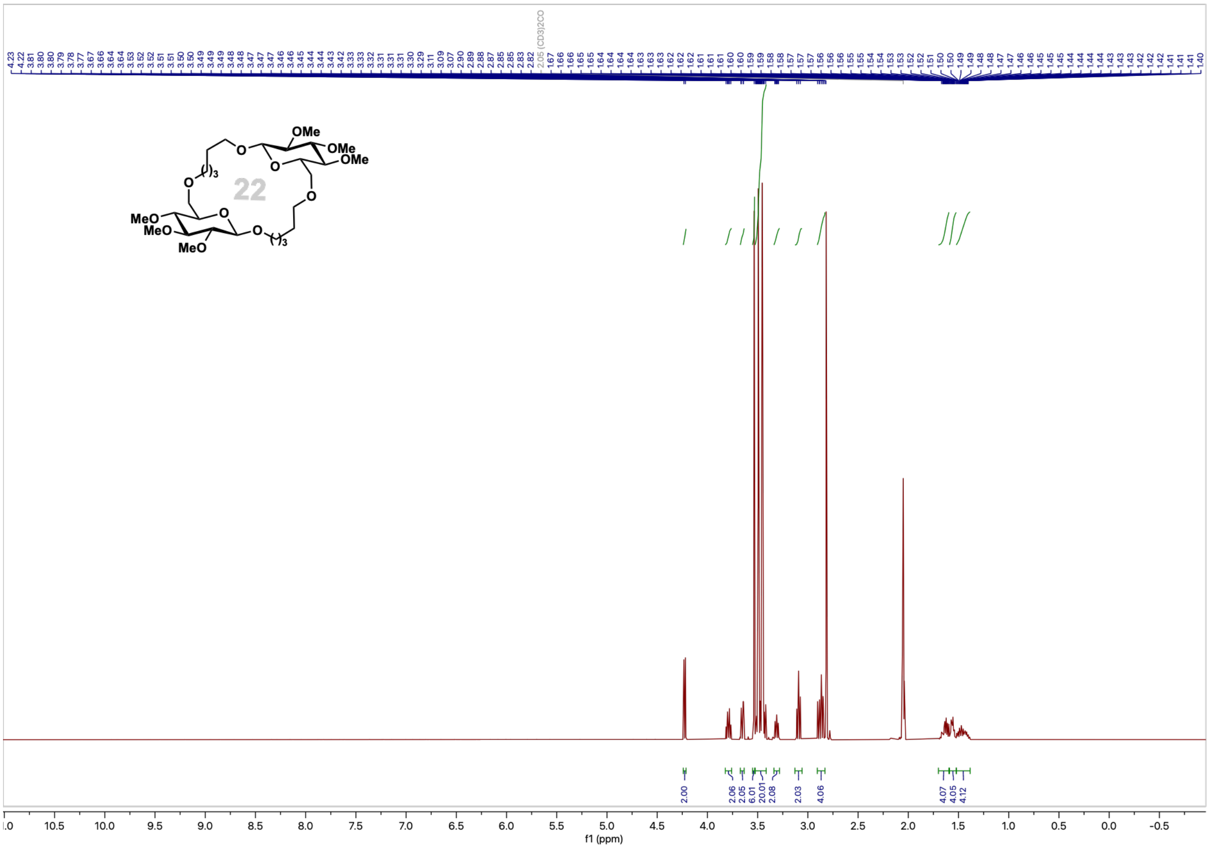

^{13}C NMR (126 MHz, acetone-d_6, 298K) of compound **3b**

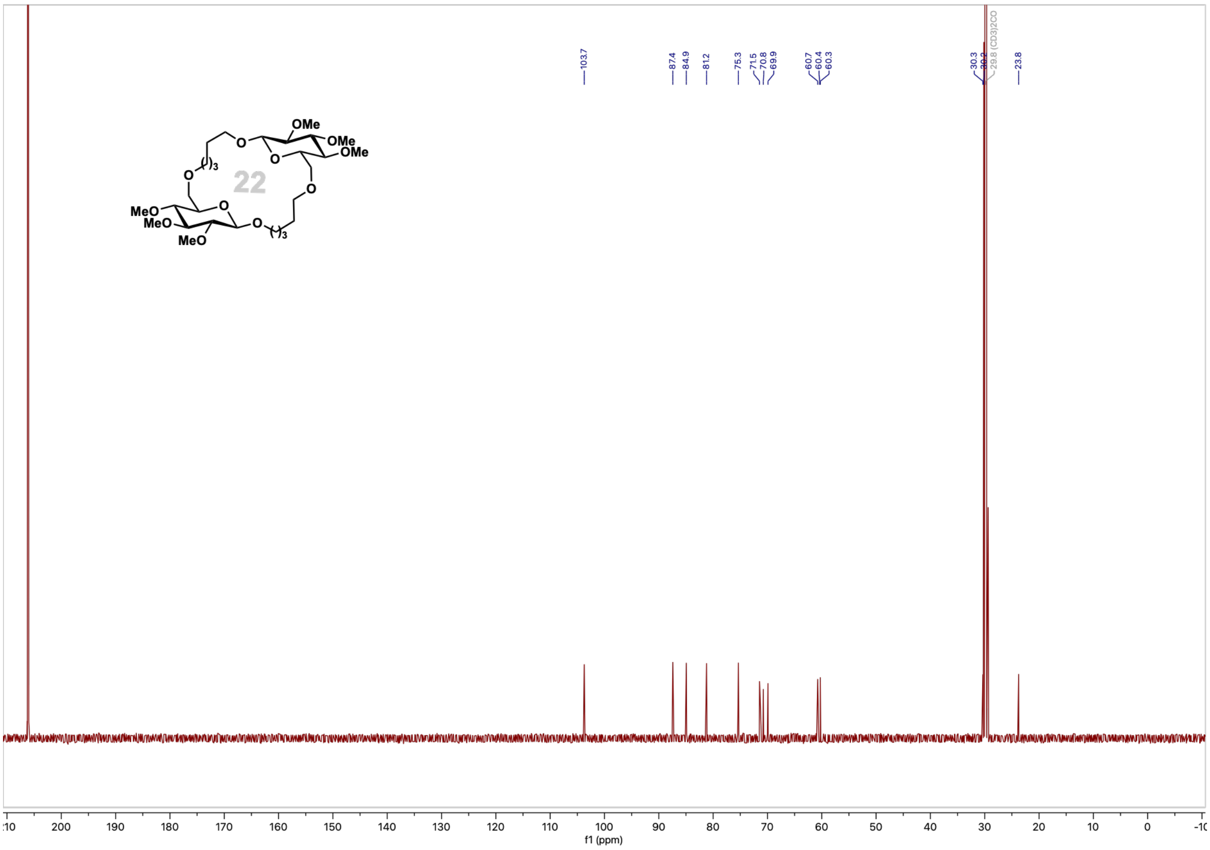

^{1}H NMR (500 MHz, acetone-d_6, 298K) of compound **2c**

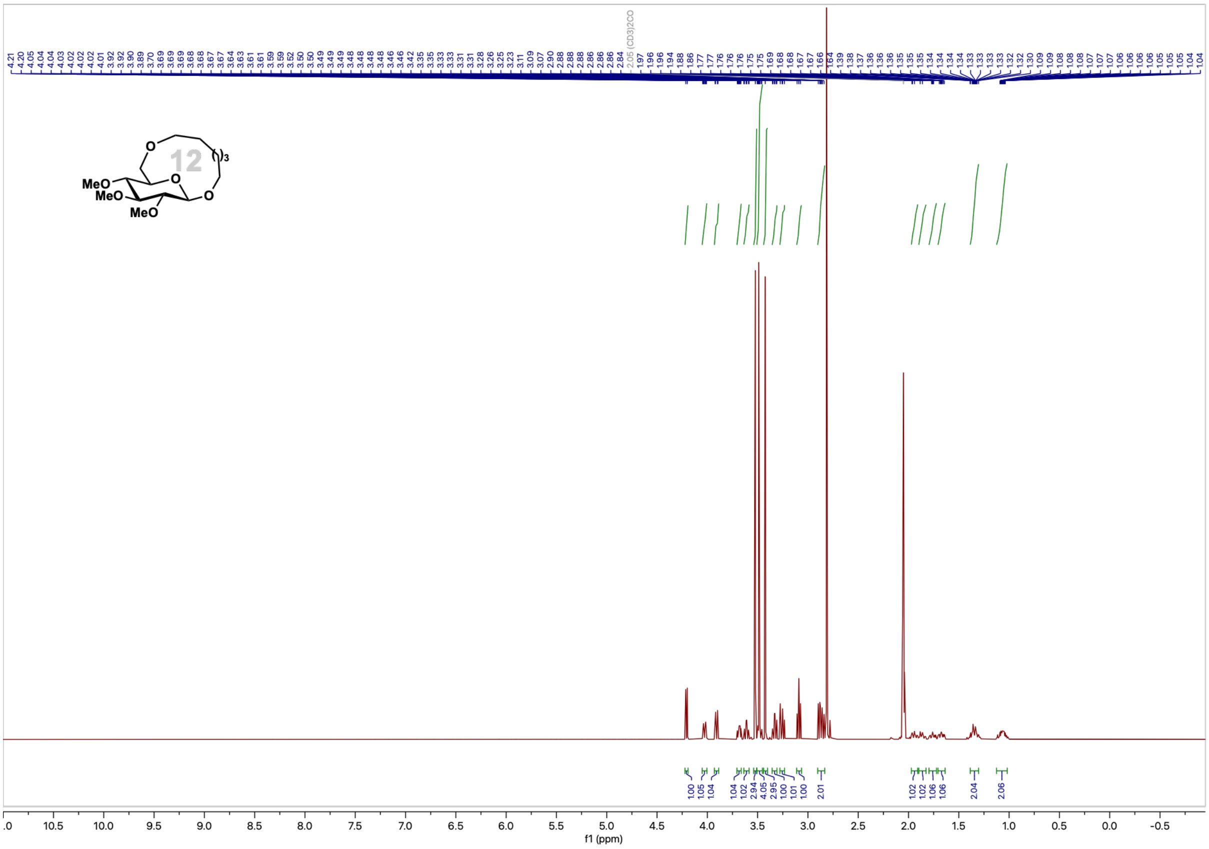

^{13}C NMR (126 MHz, acetone-d_6, 298K) of compound **2c**

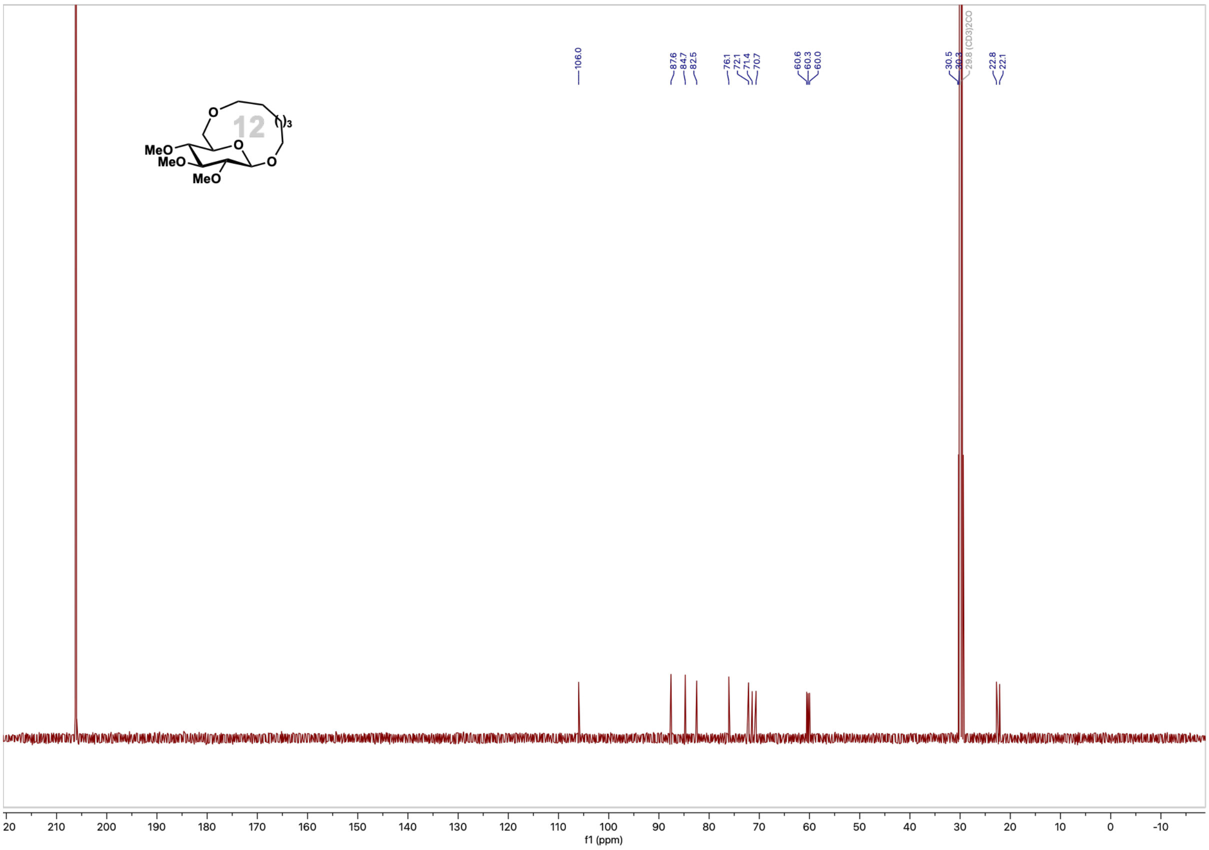

^{1}H-^{1}H COSY NMR (500 MHz, acetone-d_6, 298K) of compound **2c**

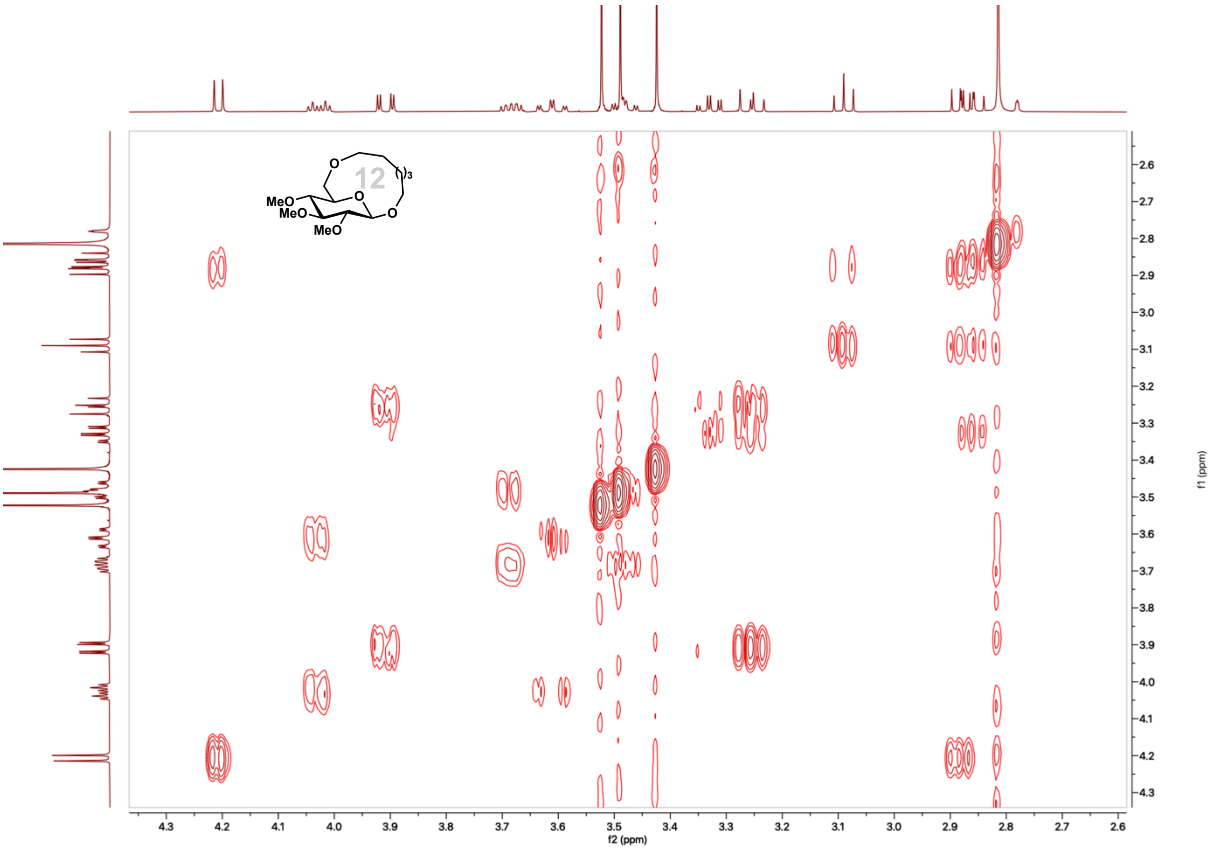

^{1}H-^{1}H NOESY NMR (500 MHz, acetone-d_6, 298K) of compound **2c**

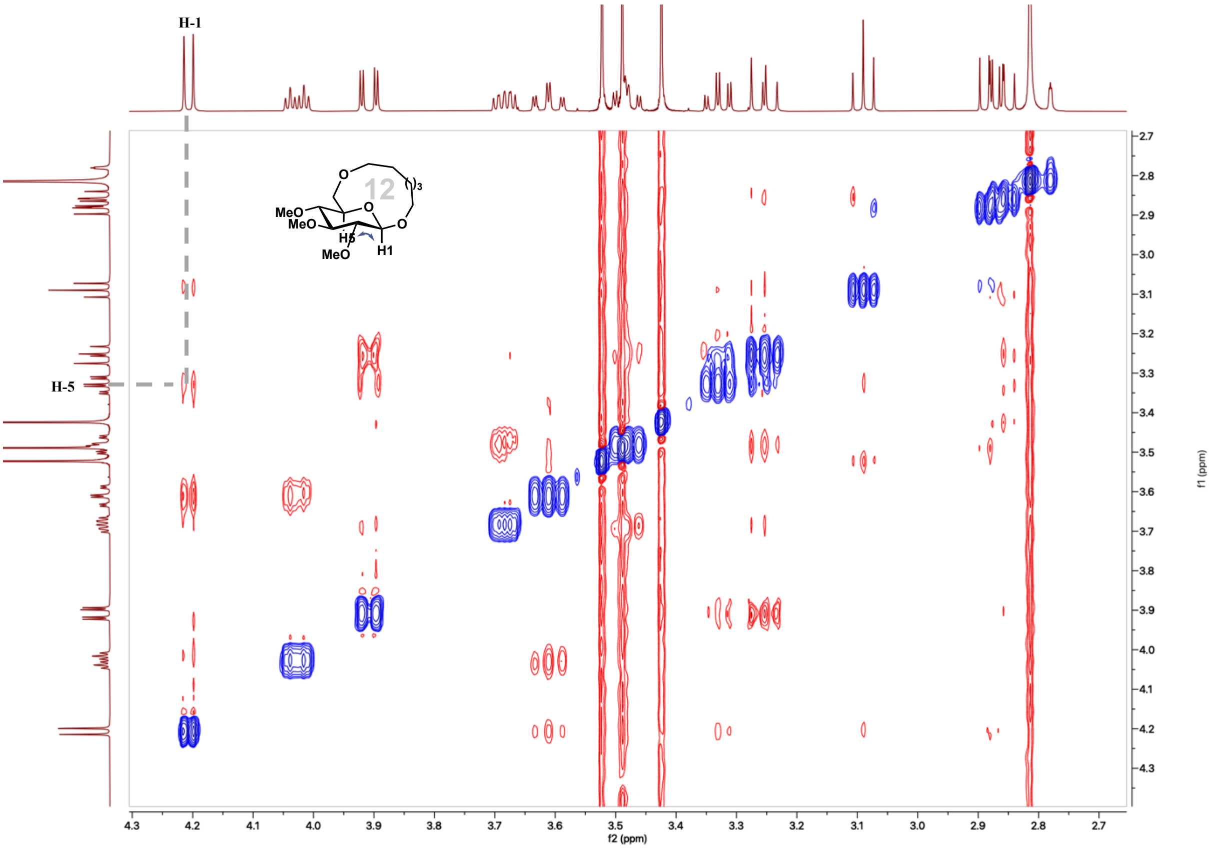

^{1}H NMR (500 MHz, acetone-d_6, 298K) of compound **Int-3c**

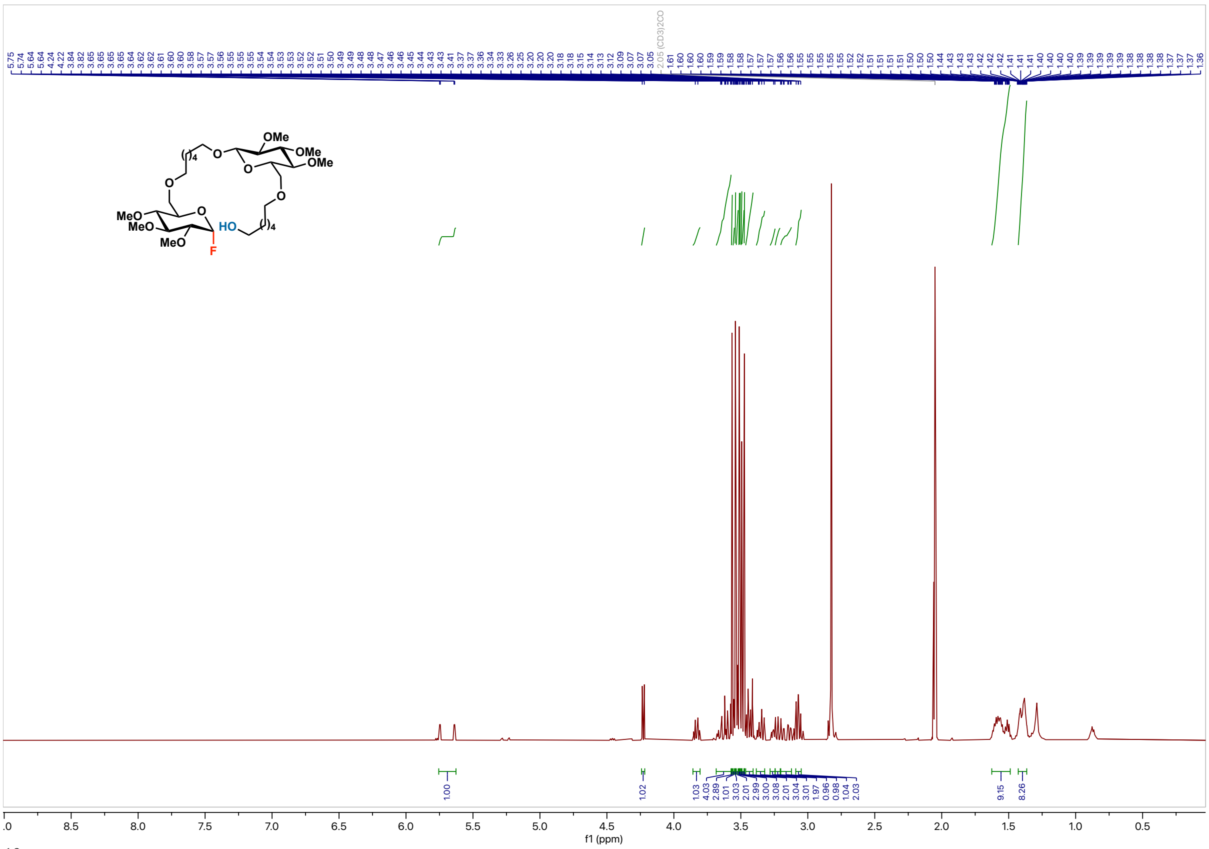

^{19}F NMR (470 MHz, acetone-d_6, 298K) of compound **Int-3c**

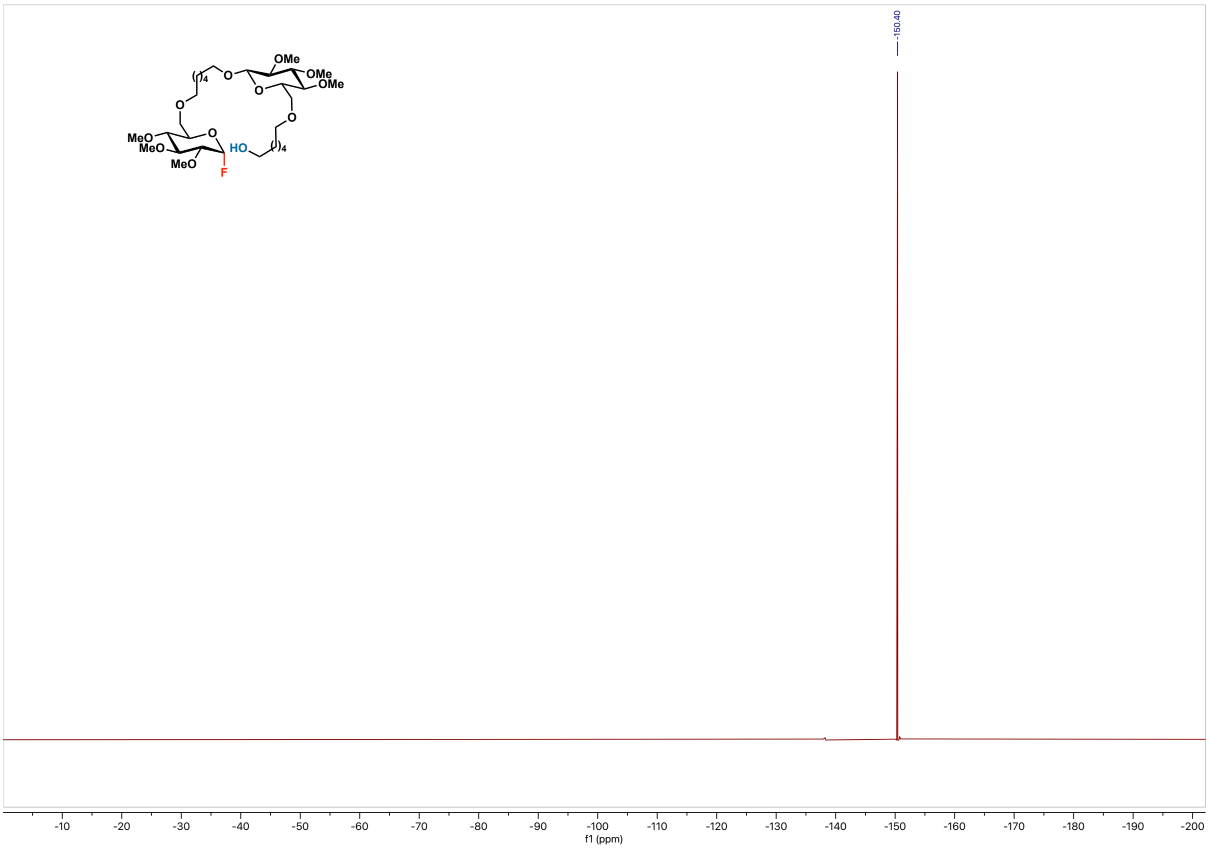

^{13}C NMR (126 MHz, acetone-d_6, 298K) of compound **Int-3c**

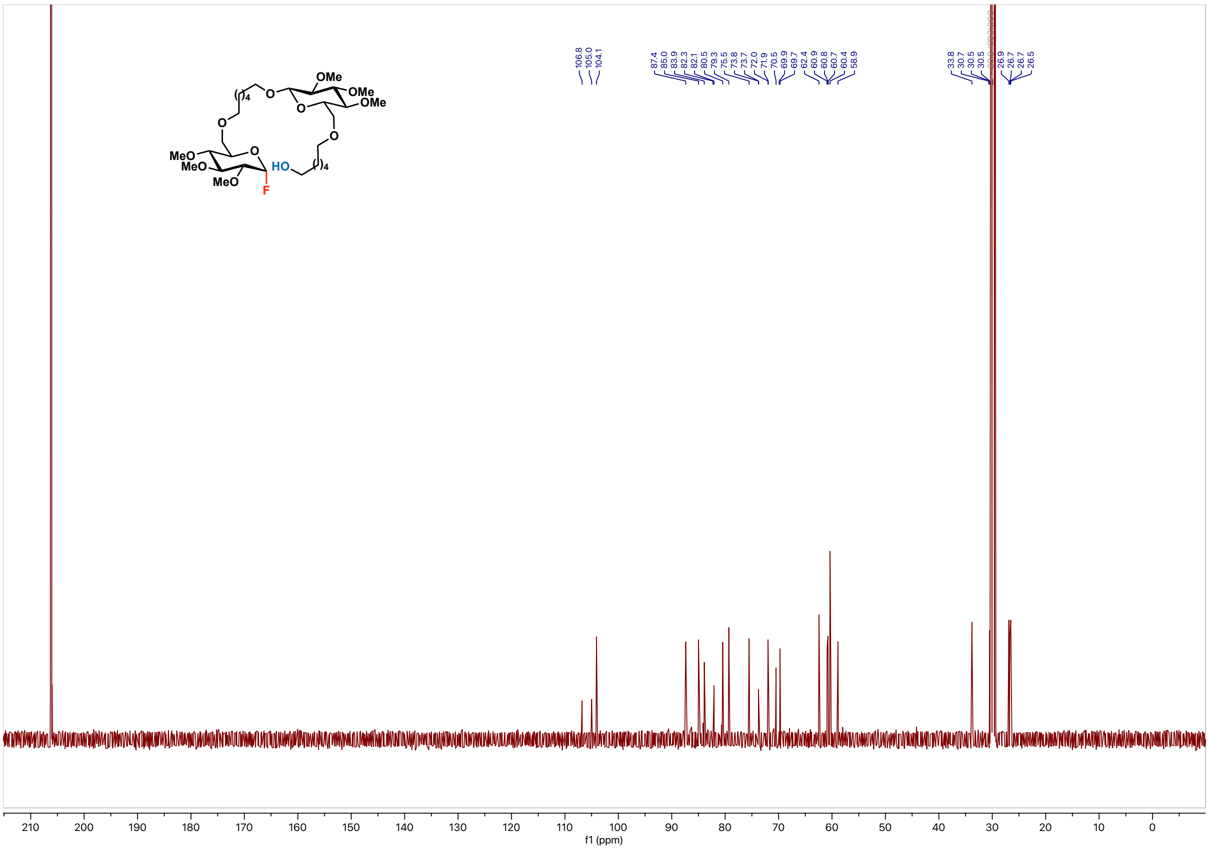

106.8
105.0
104.1
87.4
85.0
83.9
82.3
82.1
80.5
79.3
75.5
73.8
73.7
72.0
71.9
70.5
69.9
69.7
62.4
60.9
60.8
60.7
60.4
58.9
33.8
30.7
30.5
30.5
26.9
26.7
26.7
26.5
OMe
OMe
OMe
MeO
MeO
MeO
HO
F
f1 (ppm)

^{1}H NMR (500 MHz, acetone-d_6, 298K) of compound **3c**

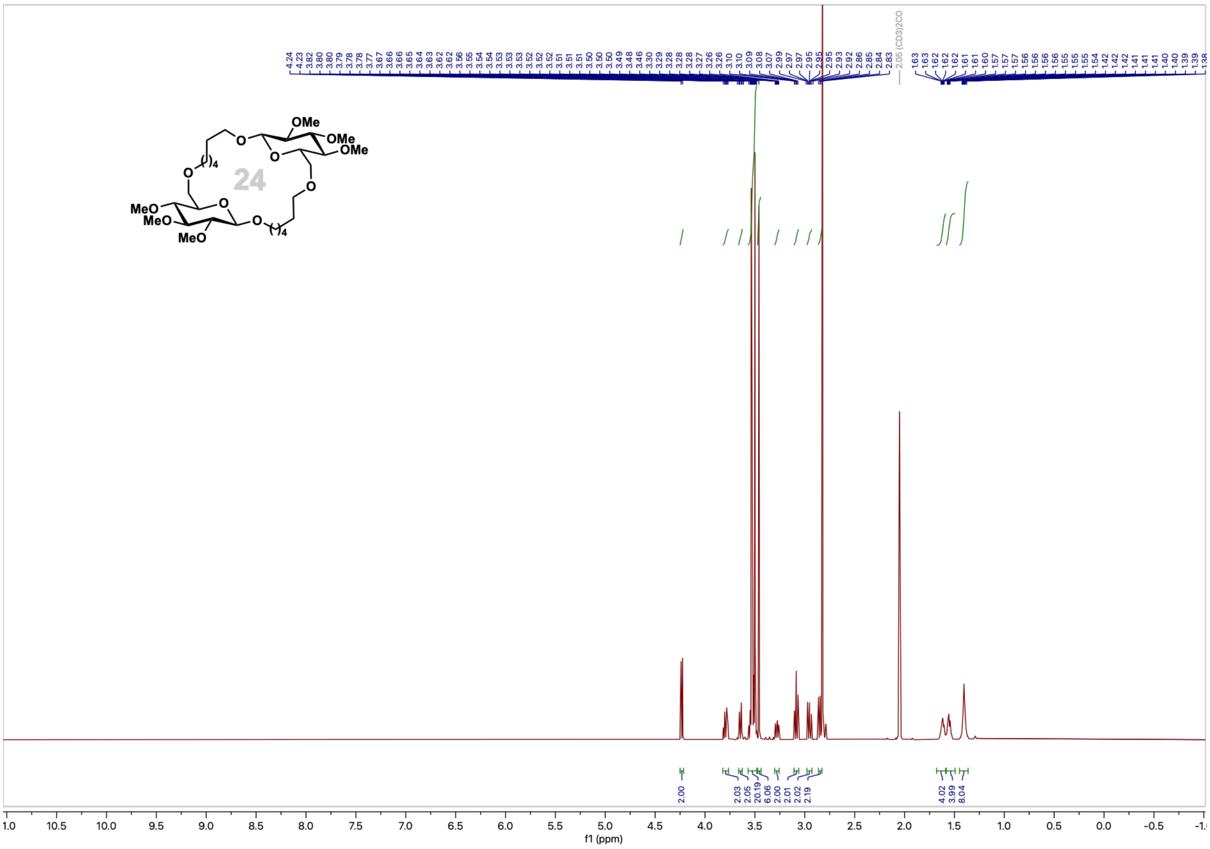

^{13}C NMR (126 MHz, acetone-d_6, 298K) of compound **3c**

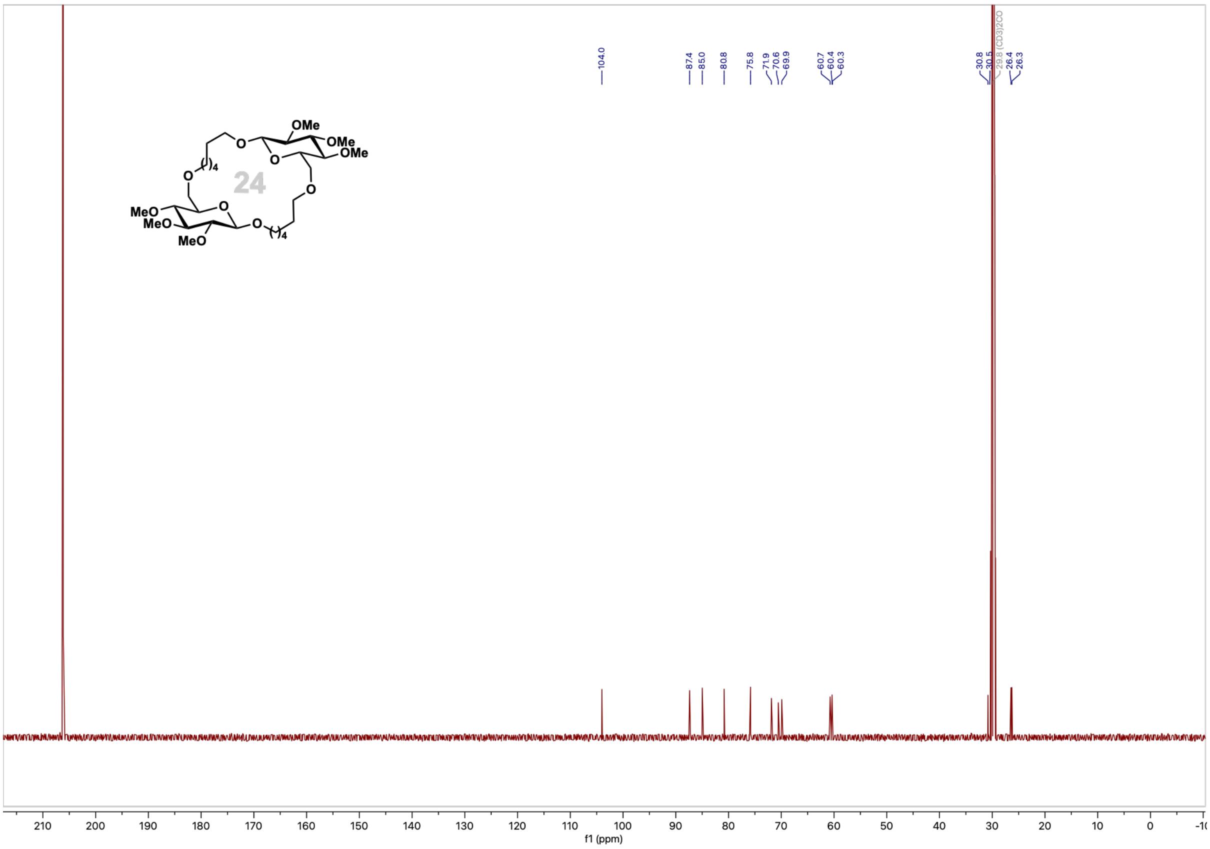

^{1}H-^{1}H COSY NMR (500 MHz, acetone-d_6, 298K) of compound **3c**

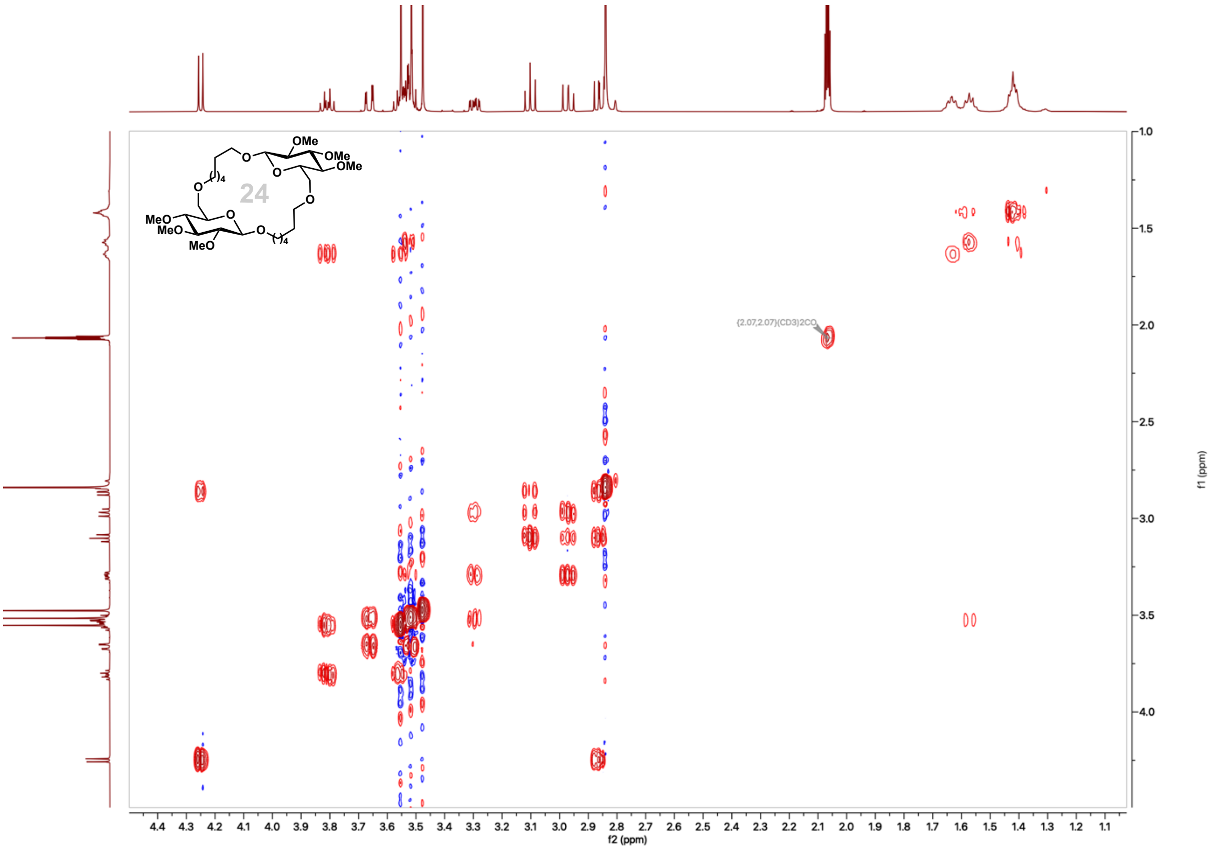

^{1}H-^{1}H NOESY NMR (500 MHz, acetone-d_6, 298K) of compound **3c**

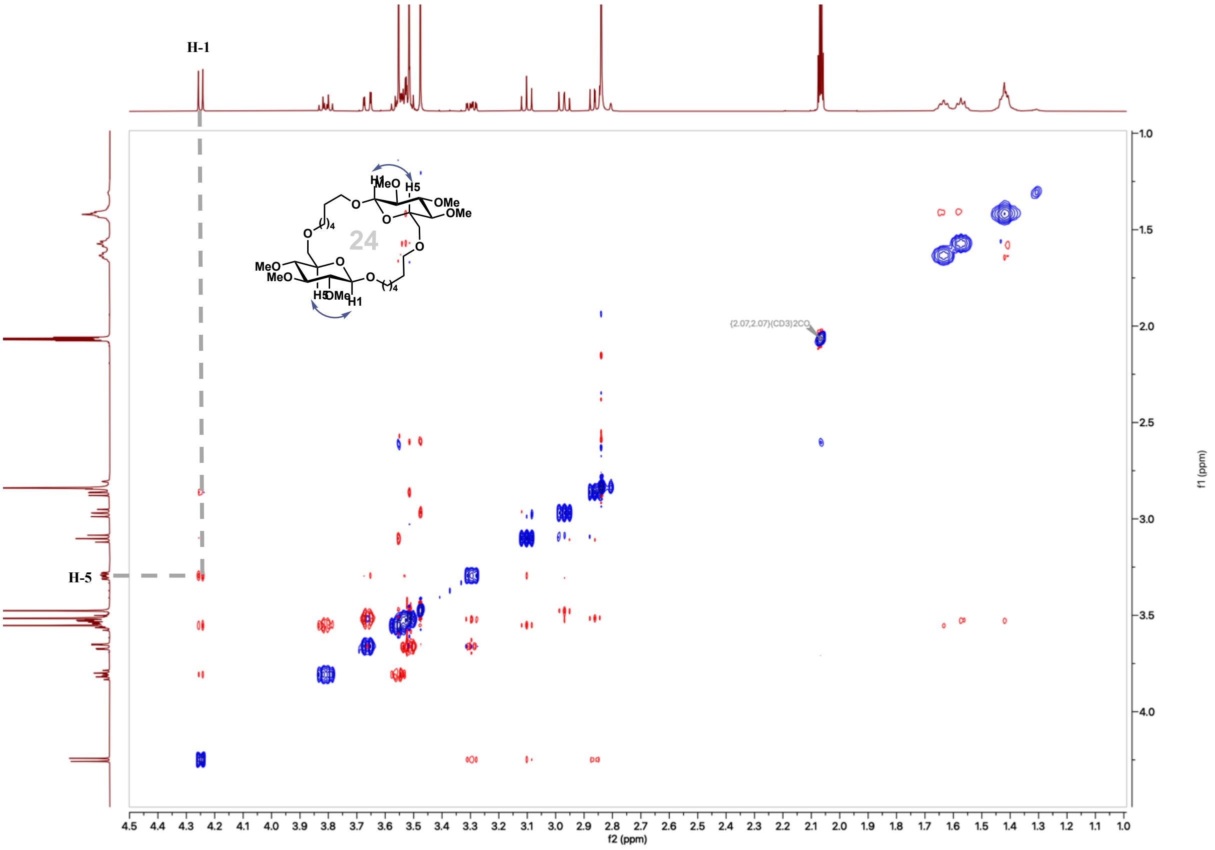

^{1}H NMR (500 MHz, acetone-d_6, 298K) of compound **2d**

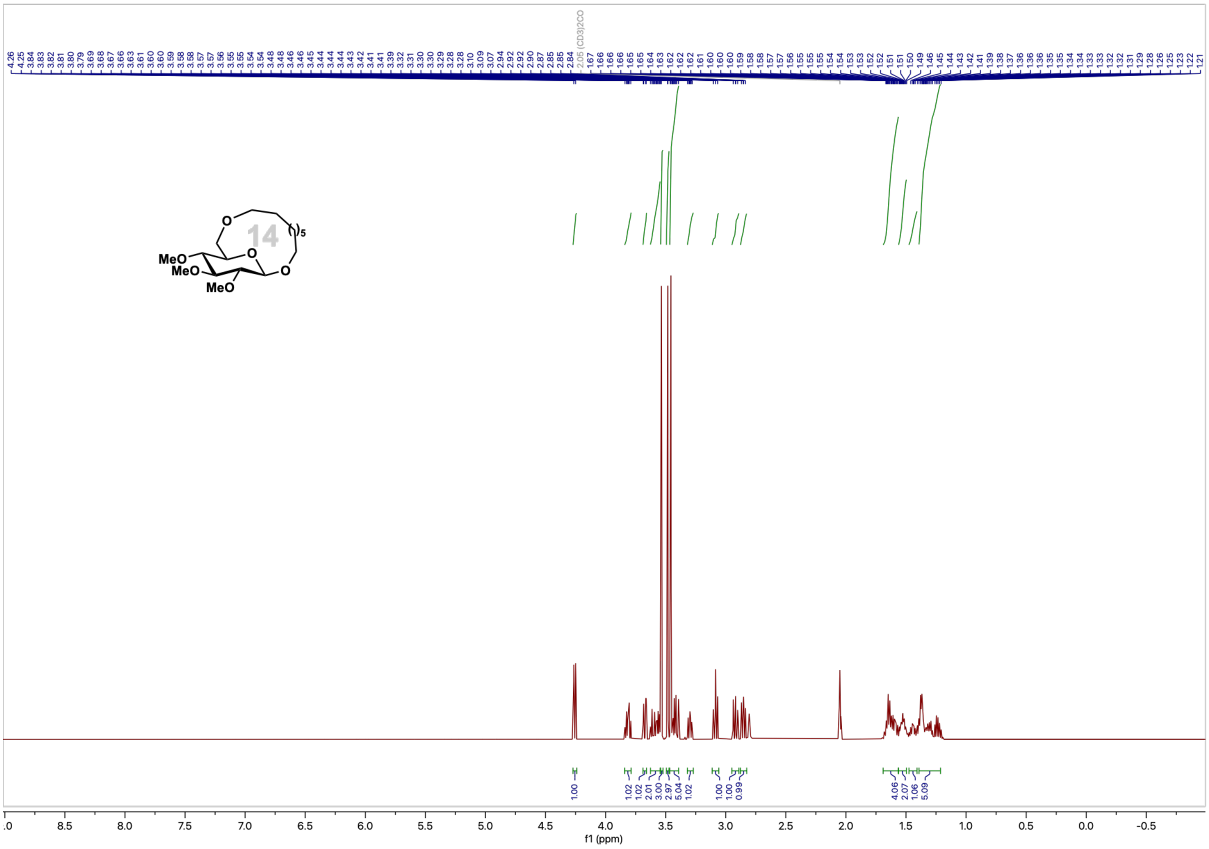

^{13}C NMR (126 MHz, acetone-d_6, 298K) of compound **2d**

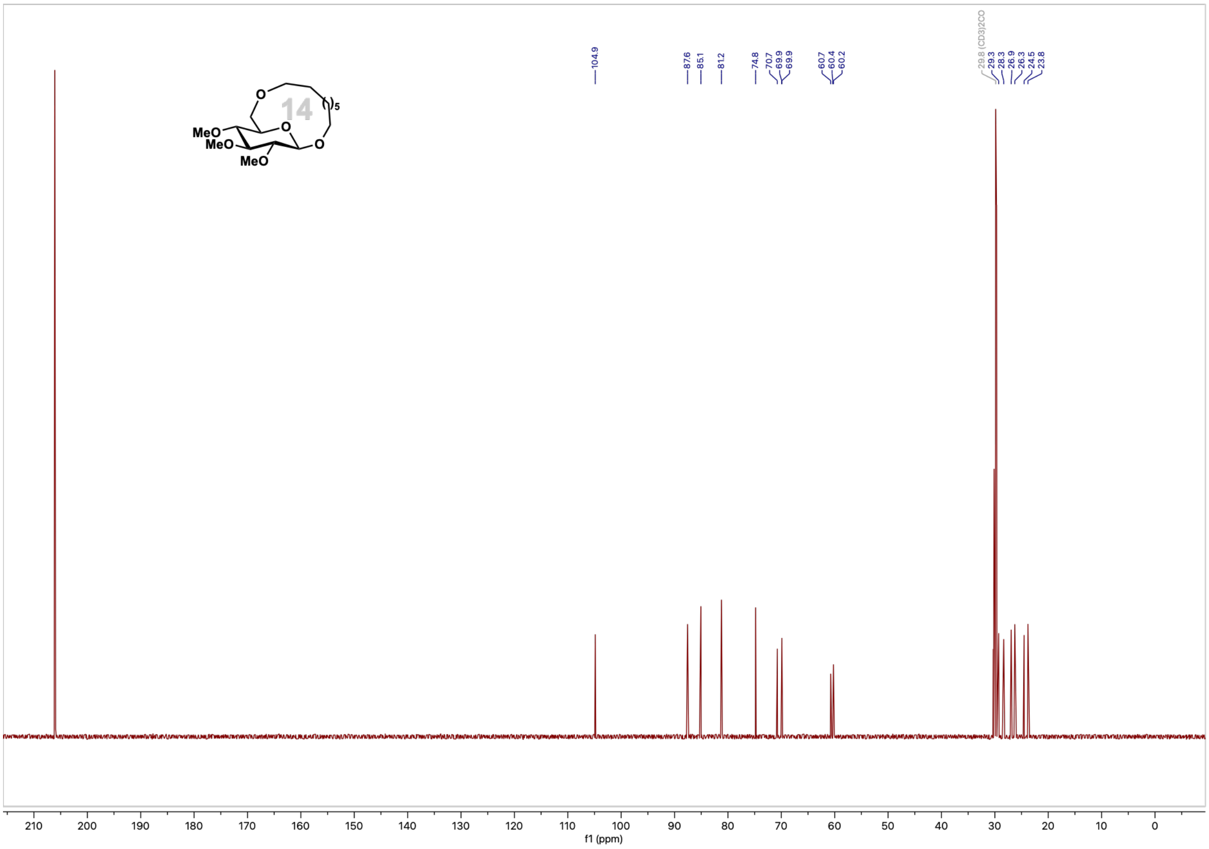

^{1}H NMR (500 MHz, acetone-d_6, 298K) of compound **3d**

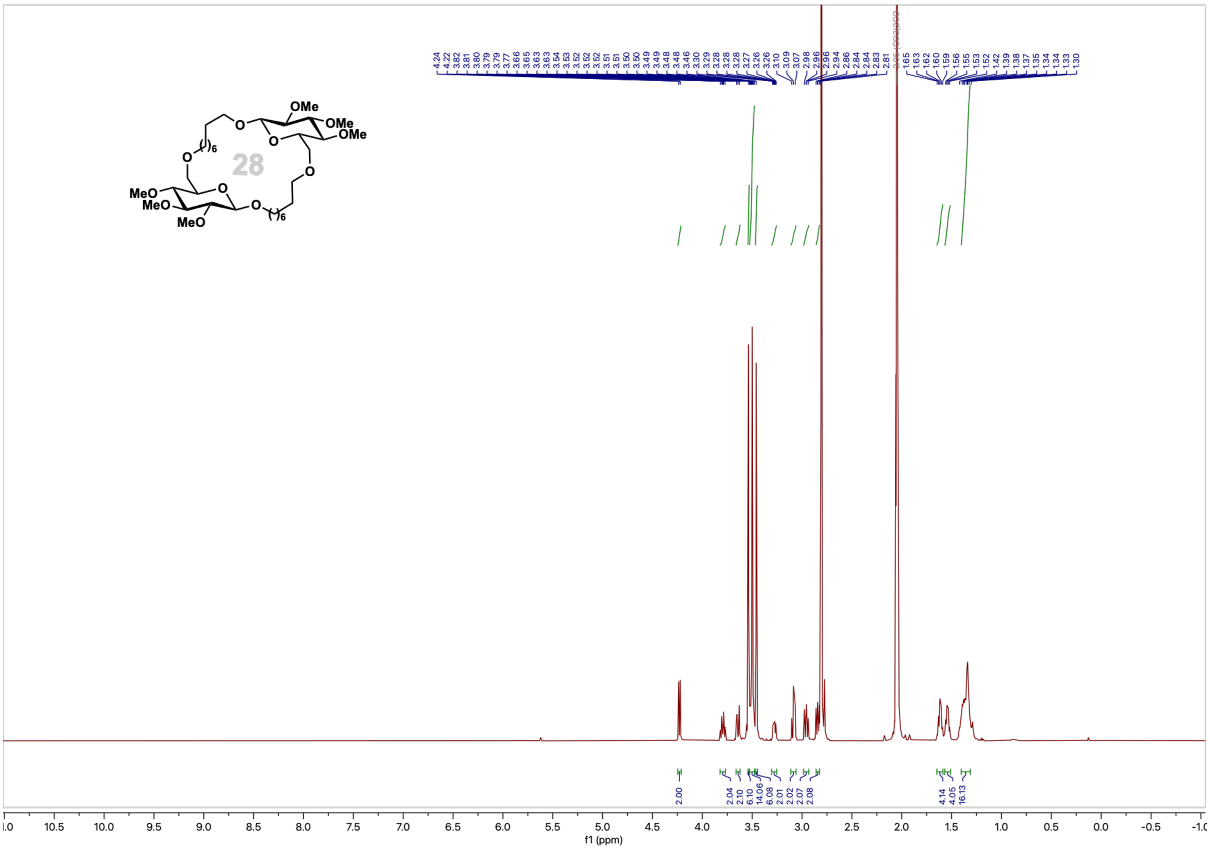

^{13}C NMR (126 MHz, acetone-d_6, 298K) of compound **3d**

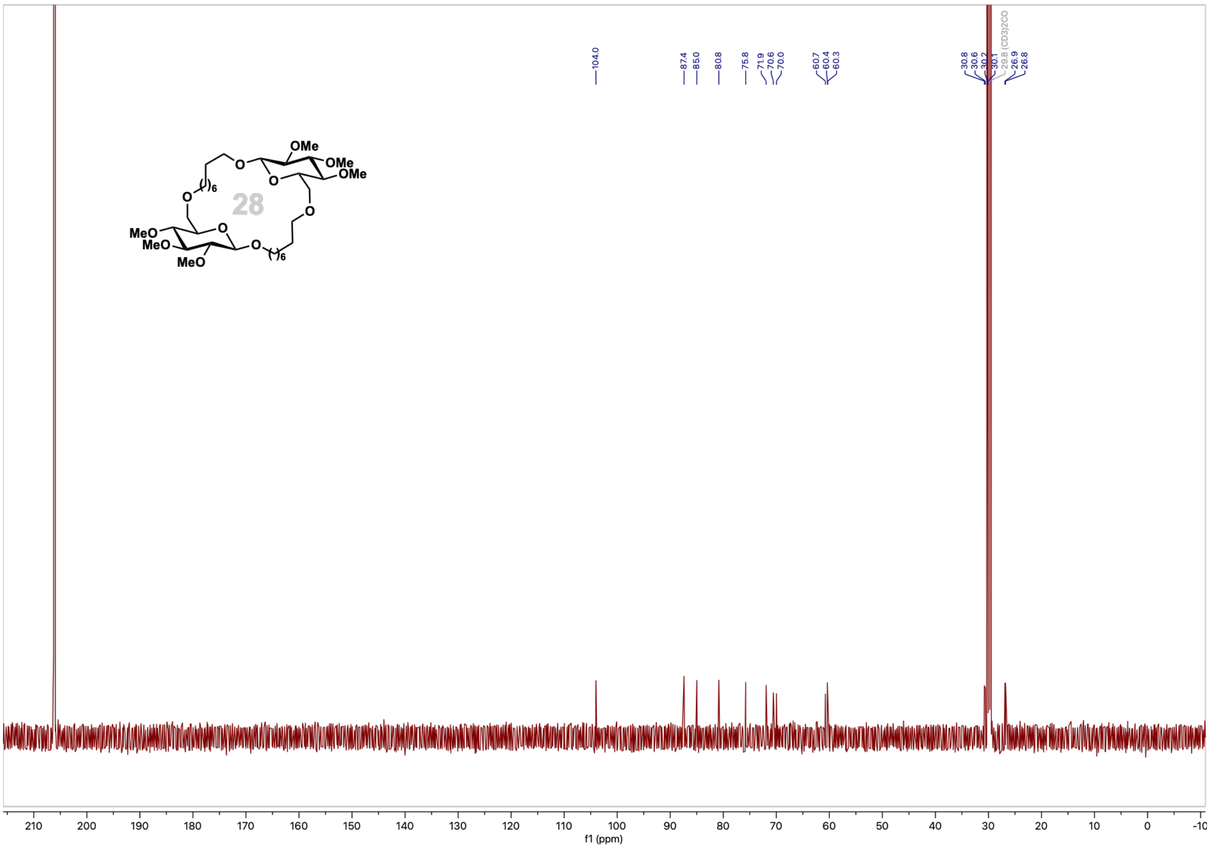

^{1}H NMR (500 MHz, acetone-d_6, 298K) of compound **2e**

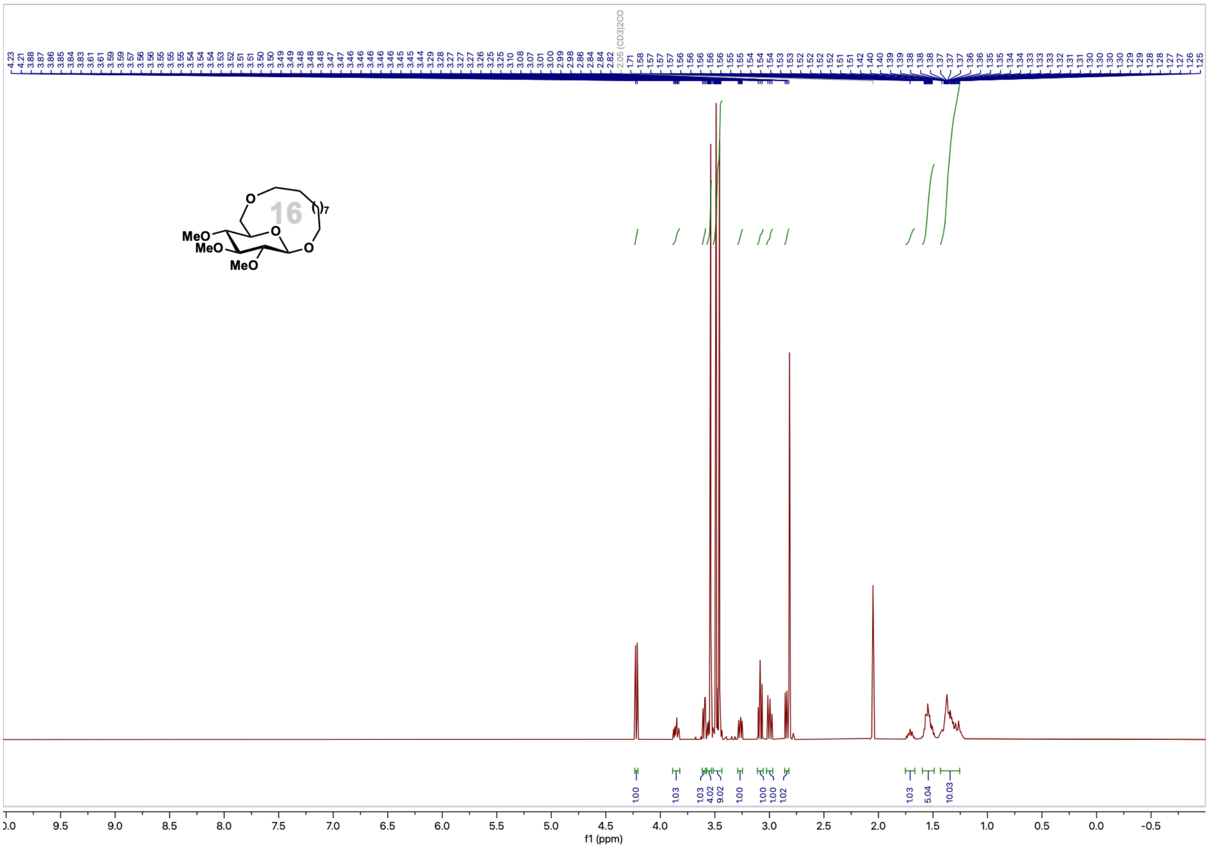

^{13}C NMR (126 MHz, acetone-d_6, 298K) of compound **2e**

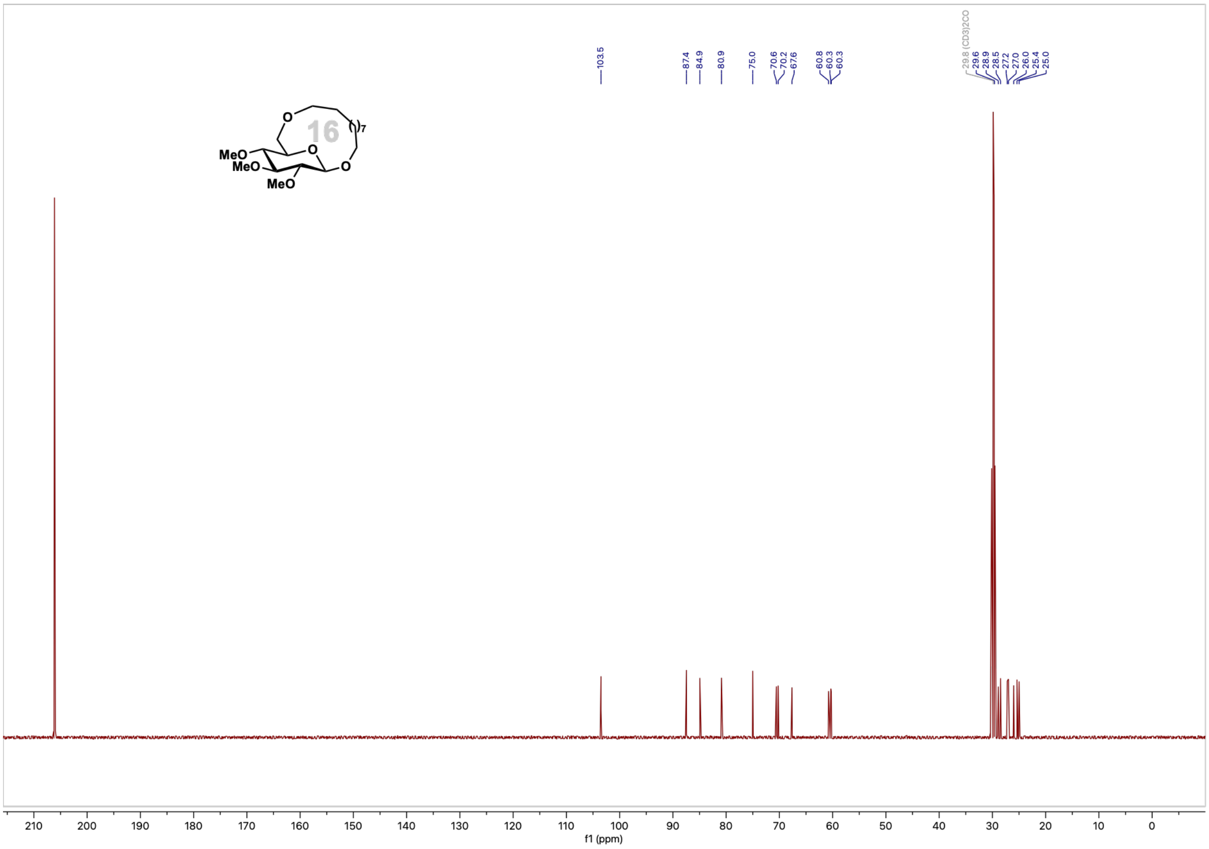

^{1}H NMR (500 MHz, acetone-d_6, 298K) of compound **2f**

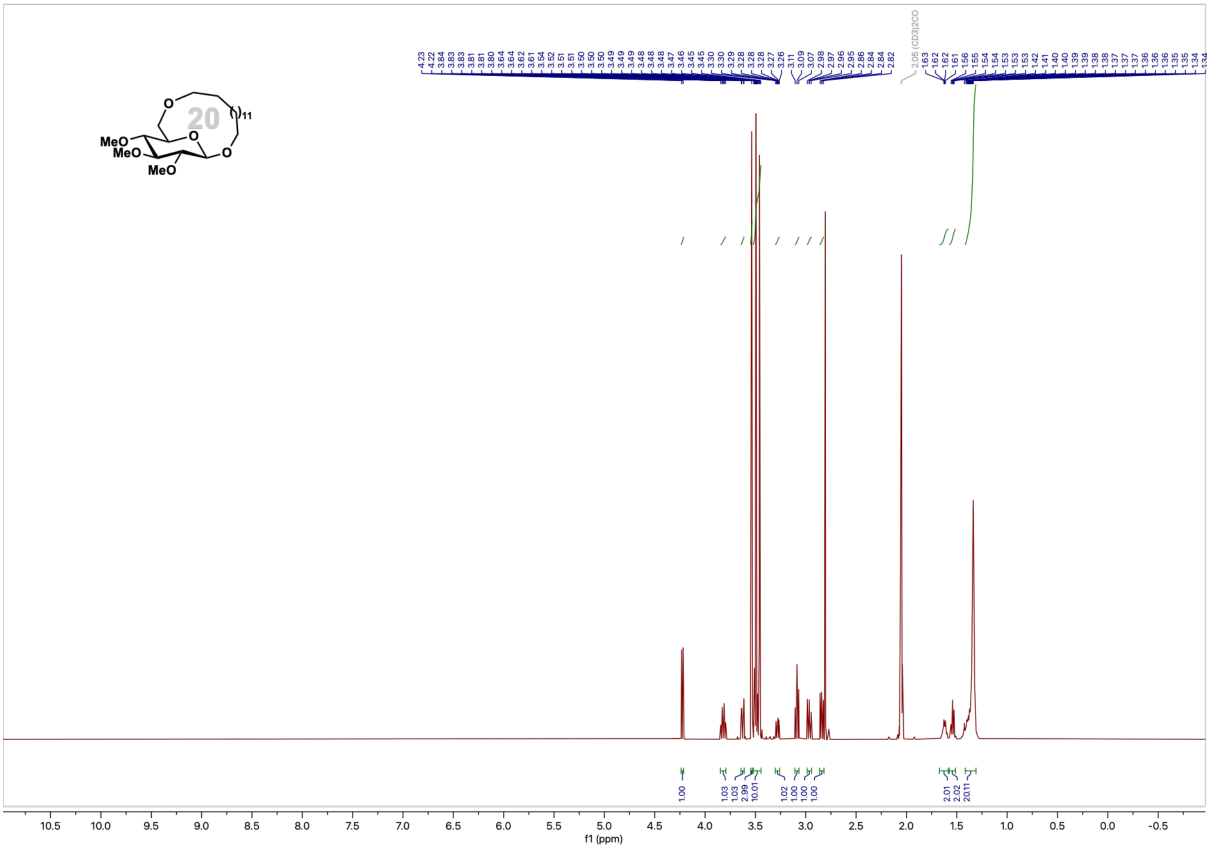

^{13}C NMR (126 MHz, acetone-d_6, 298K) of compound **2f**

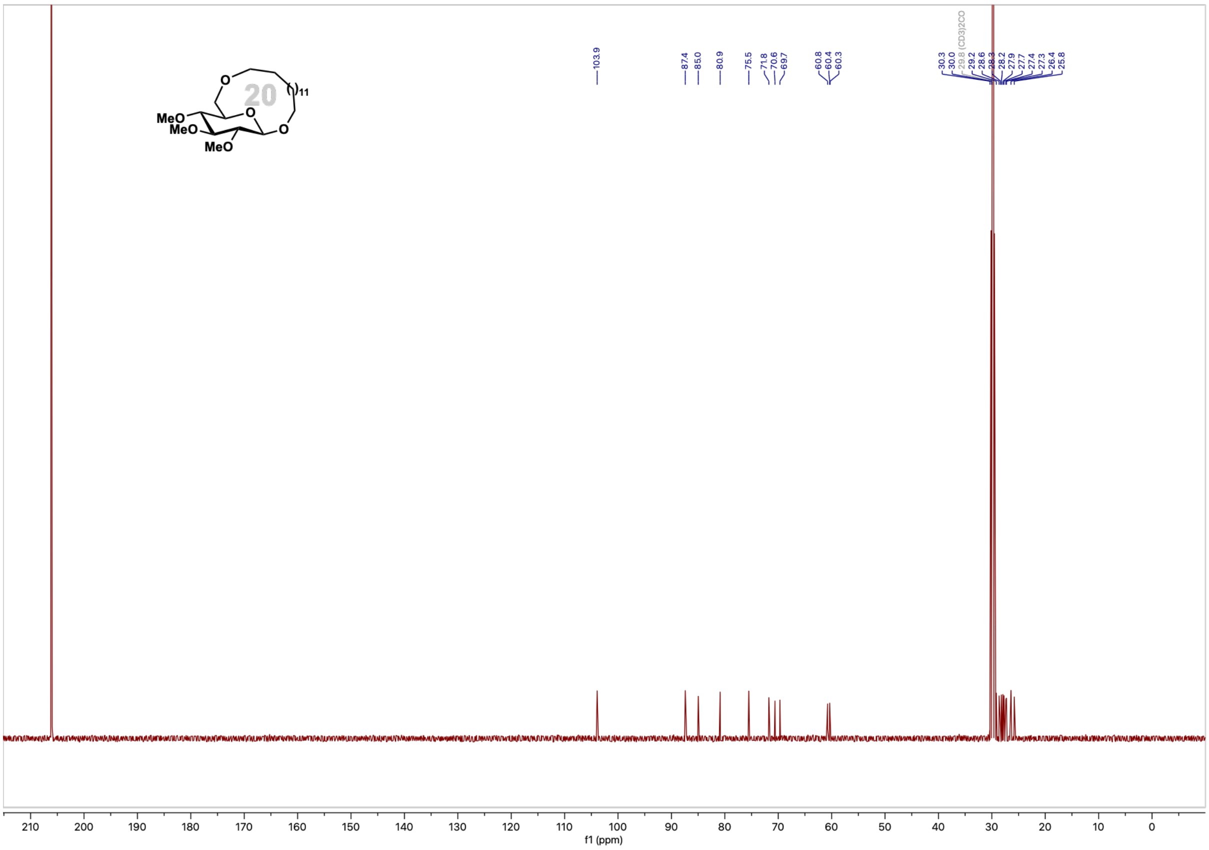

^{1}H NMR (500 MHz, acetone-d_6, 298K) of compound **2g**

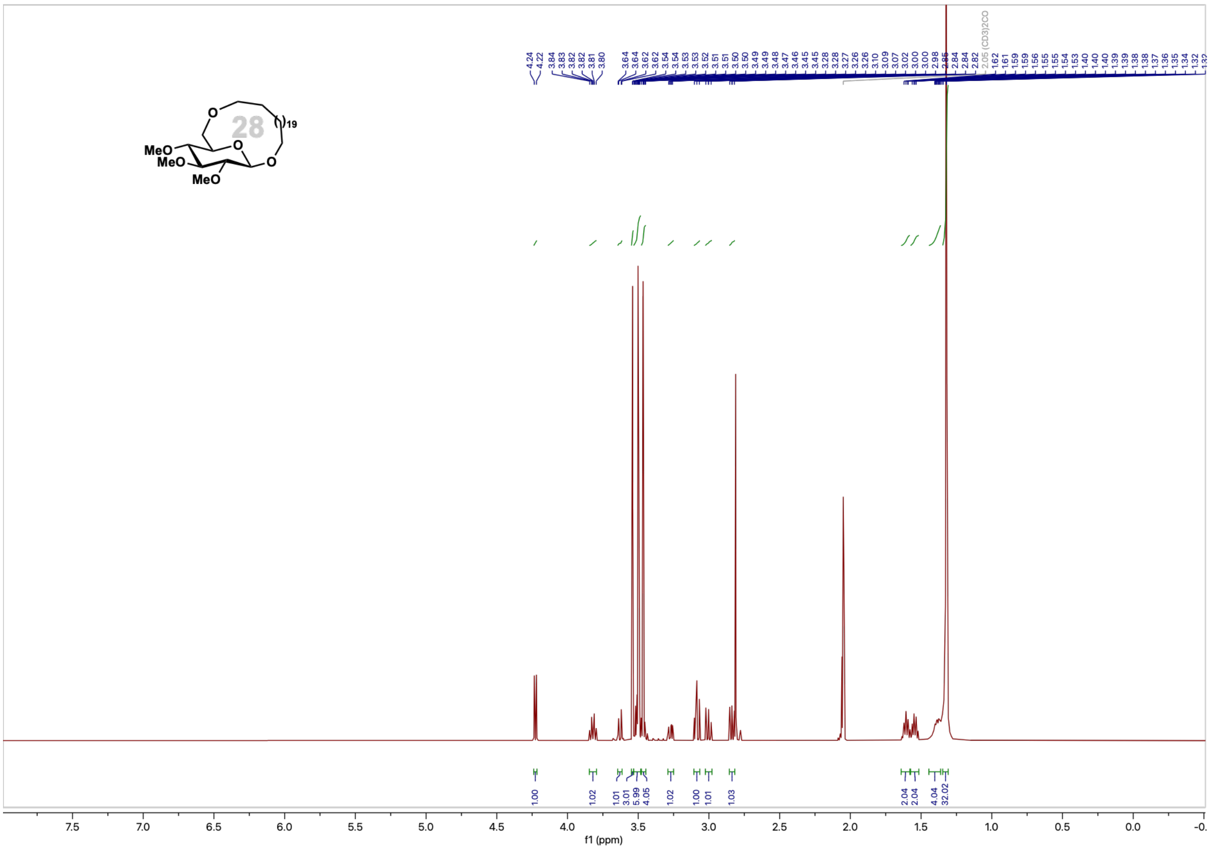

^{13}C NMR (126 MHz, acetone-d_6, 298K) of compound **2f**

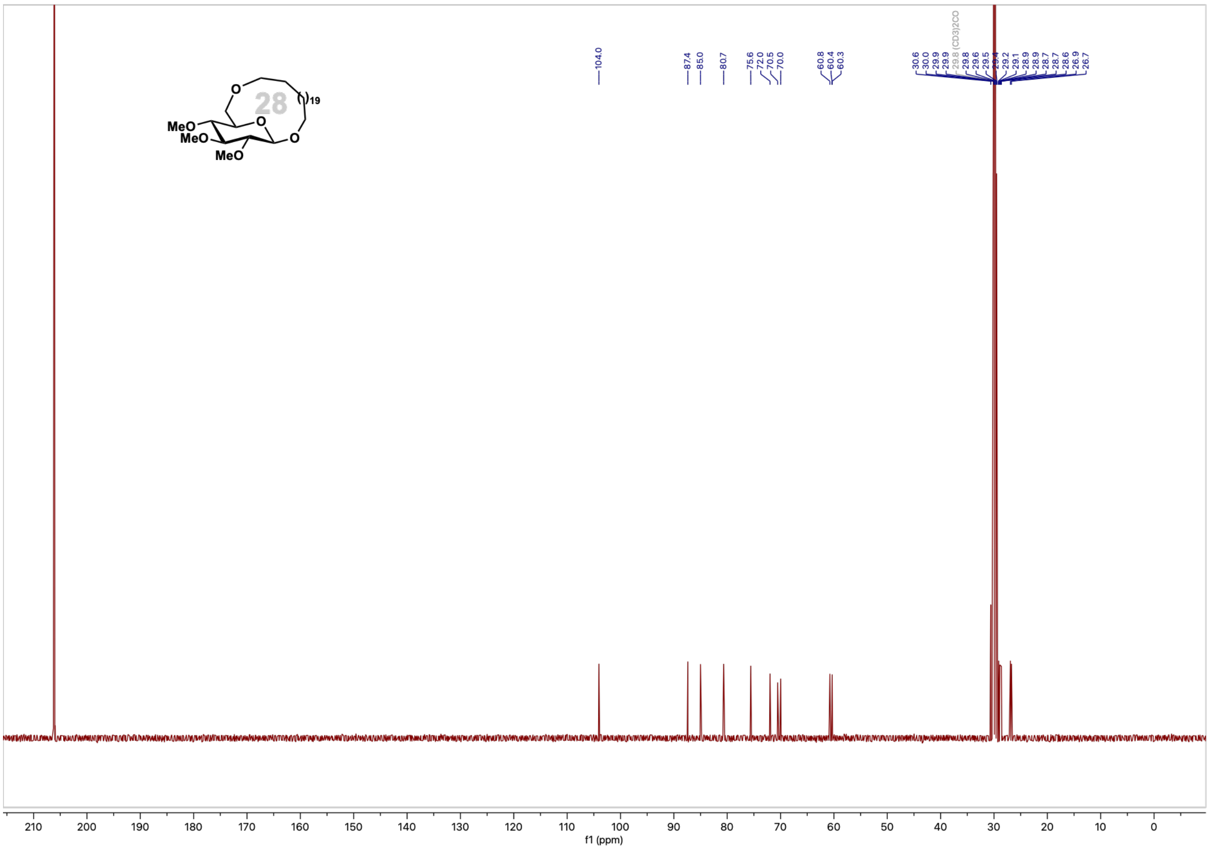

^{1}H NMR (500 MHz, acetone-d_6, 298K) of compound **2h**

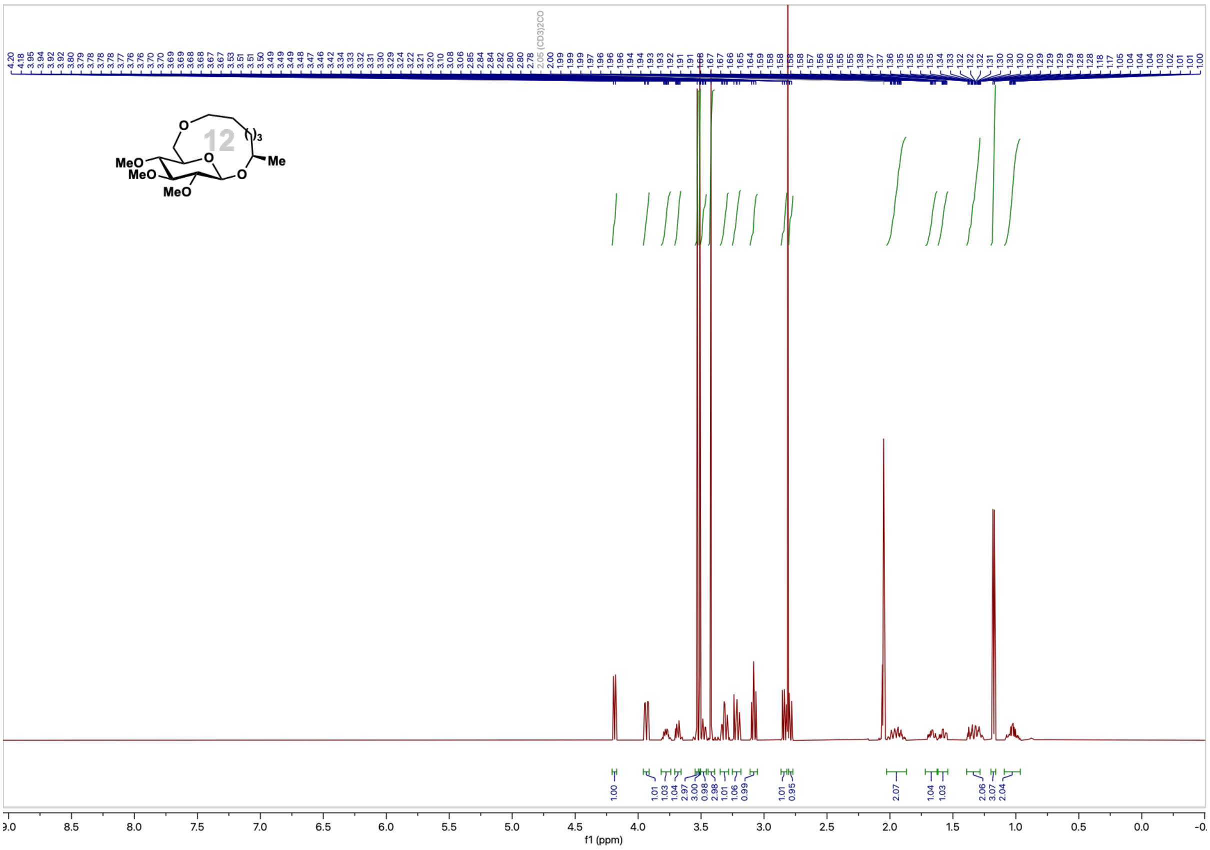

^{13}C NMR (126 MHz, acetone-d_6, 298K) of compound **2h**

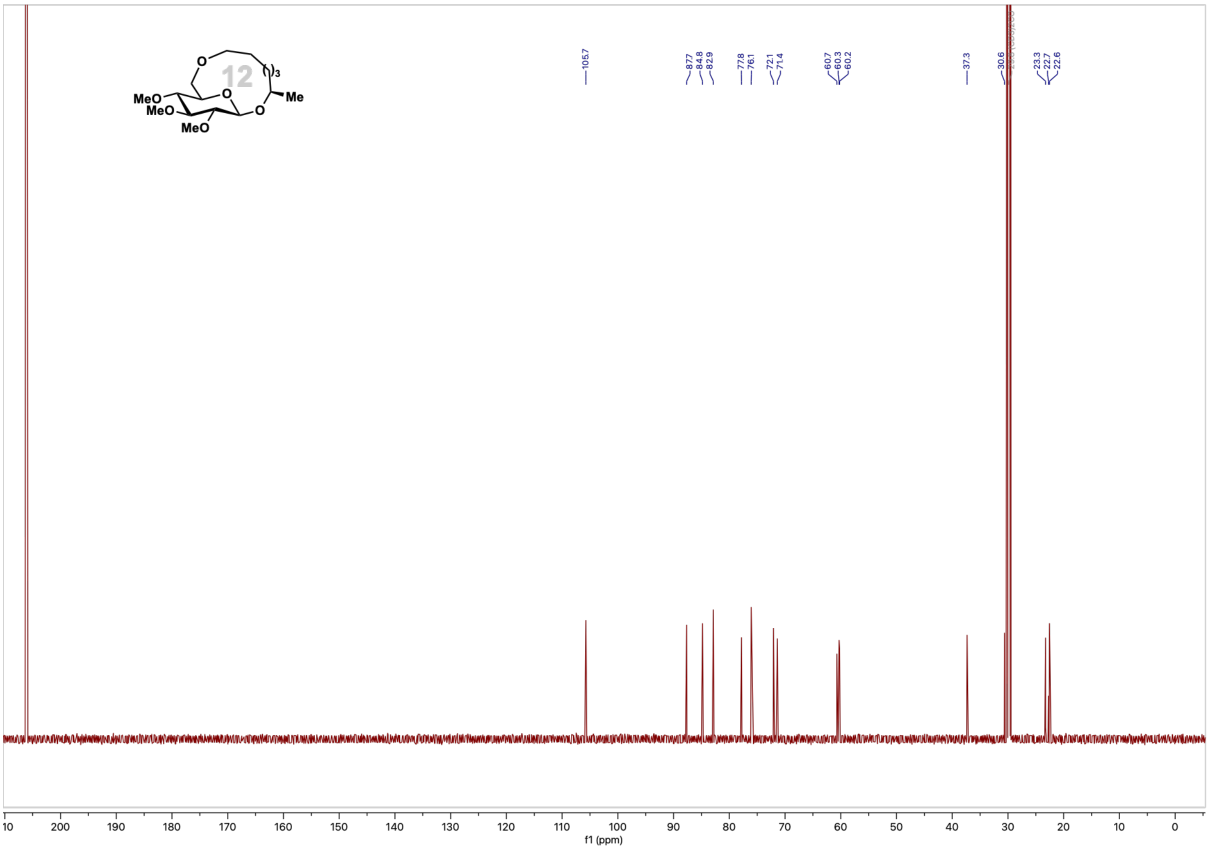

^{1}H NMR (500 MHz, acetone-d_6, 298K) of compound **2i**

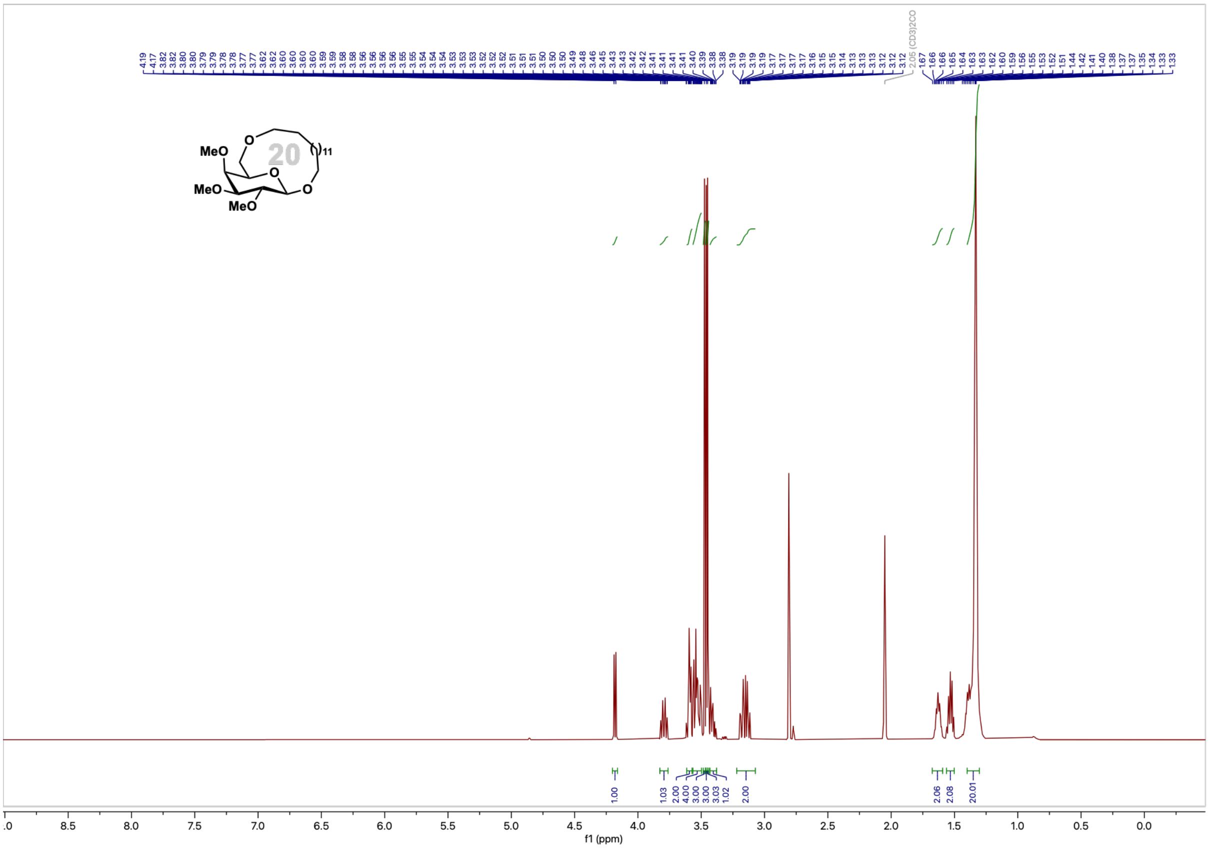

^{13}C NMR (126 MHz, acetone-d_6, 298K) of compound **2i**

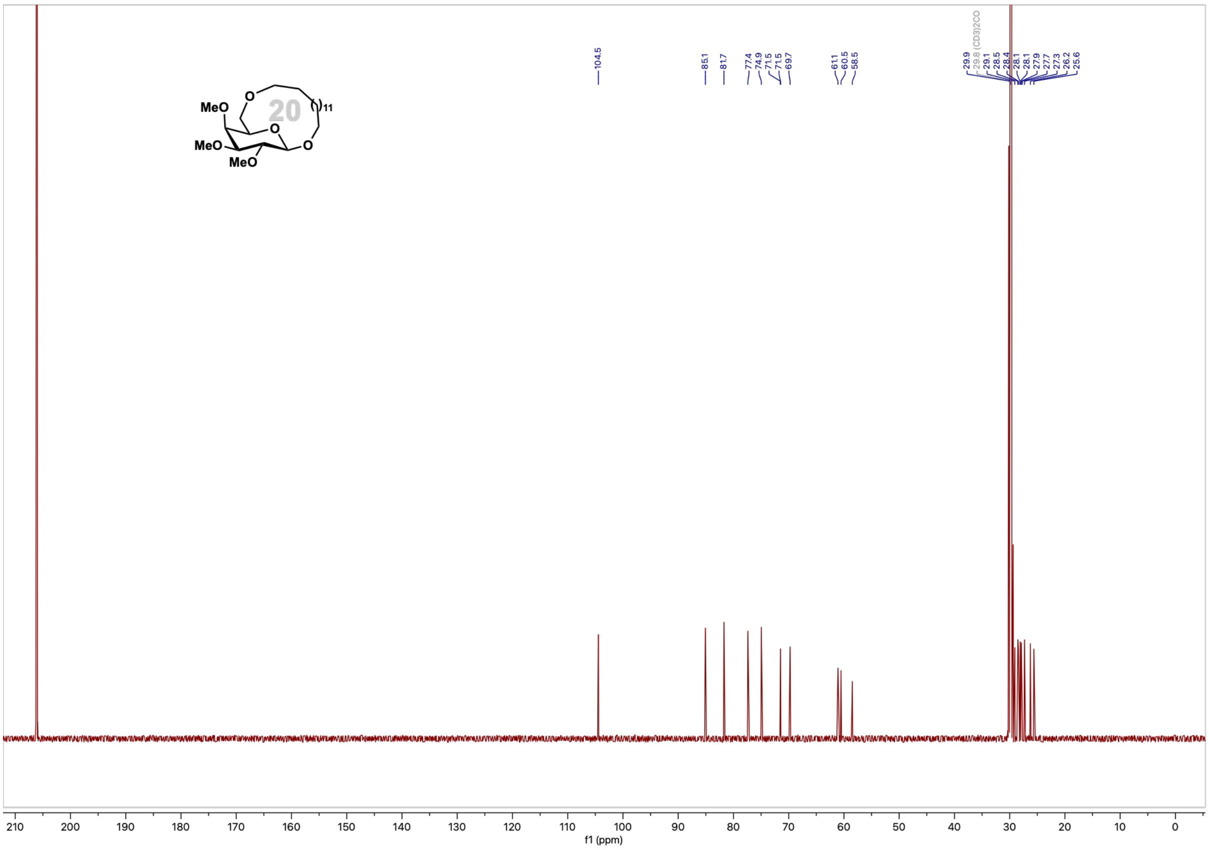

^{1}H NMR (500 MHz, acetone-d_6, 298K) of compound **2j**

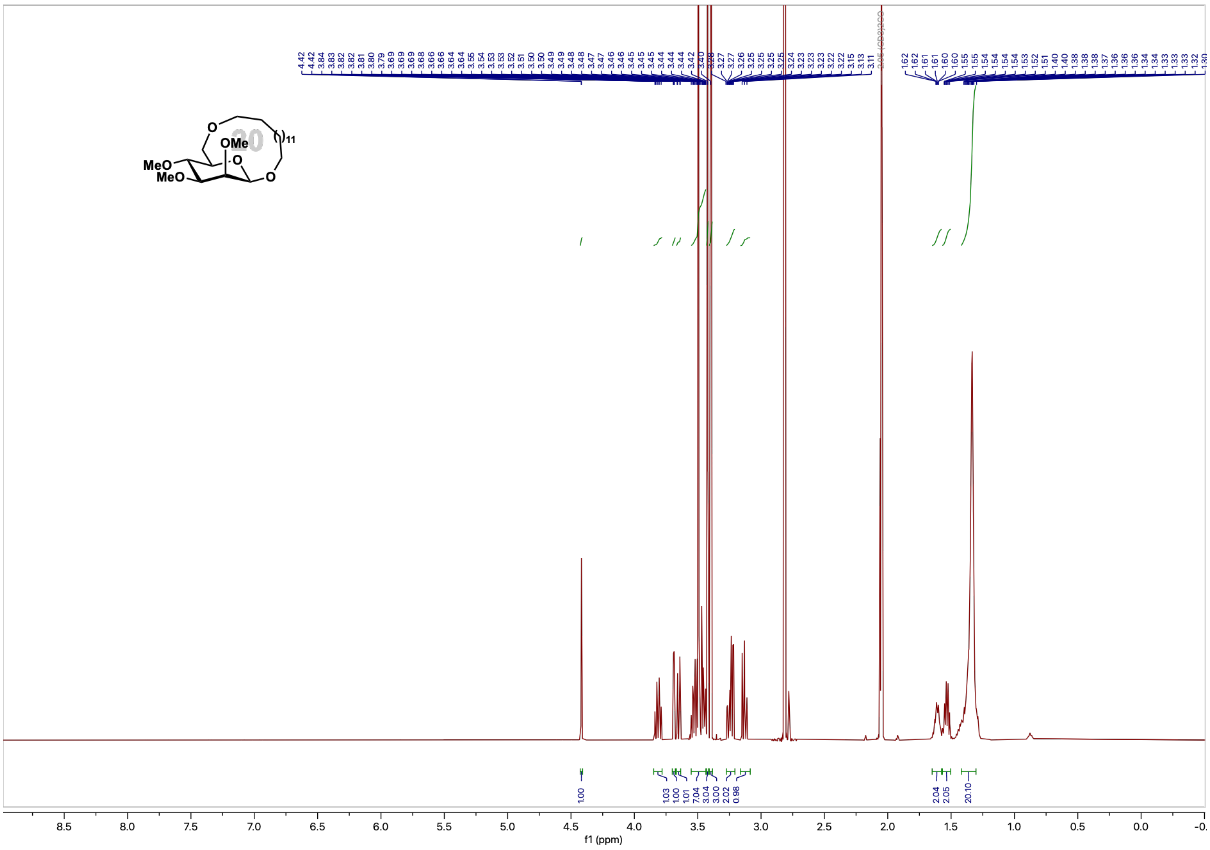

^{13}C NMR (126 MHz, acetone-d_6, 298K) of compound **2j**

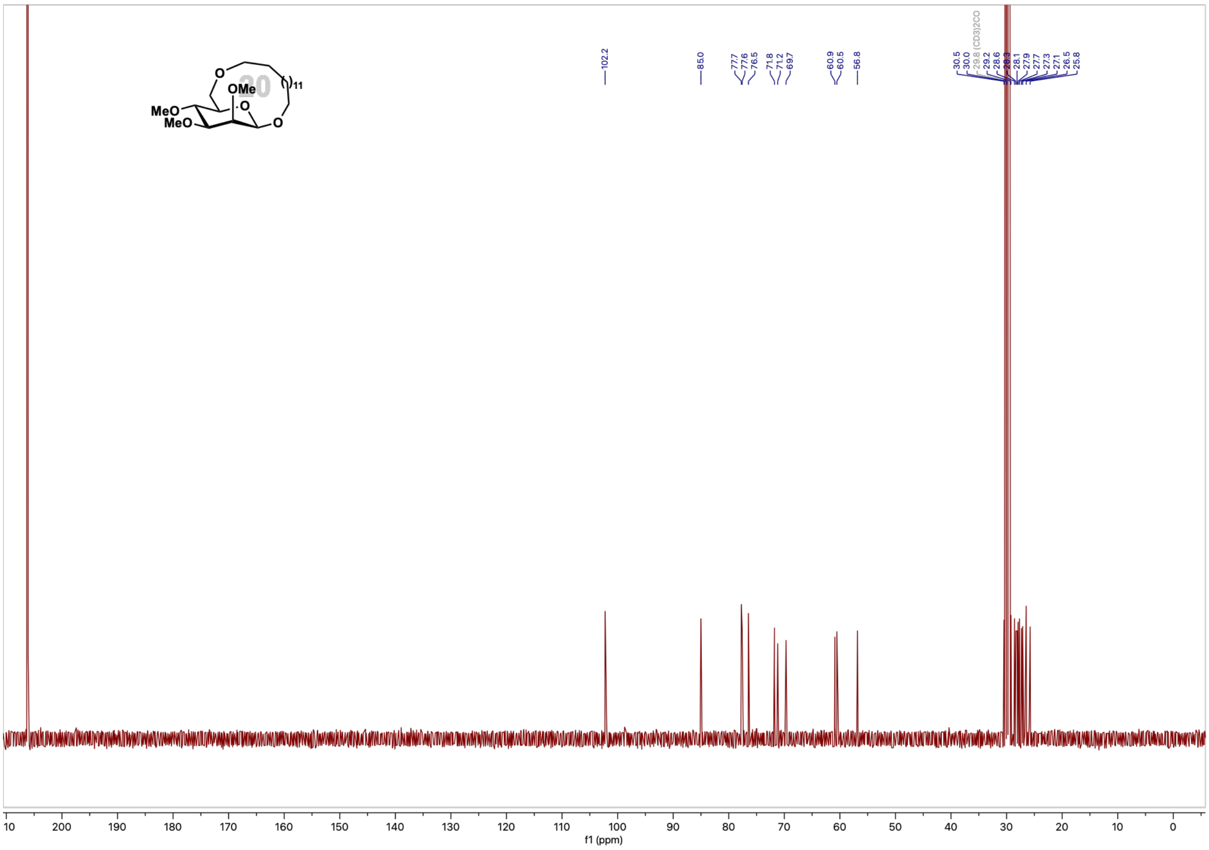

^{1}H NMR (500 MHz, acetone-d_6, 298K) of compound **4**

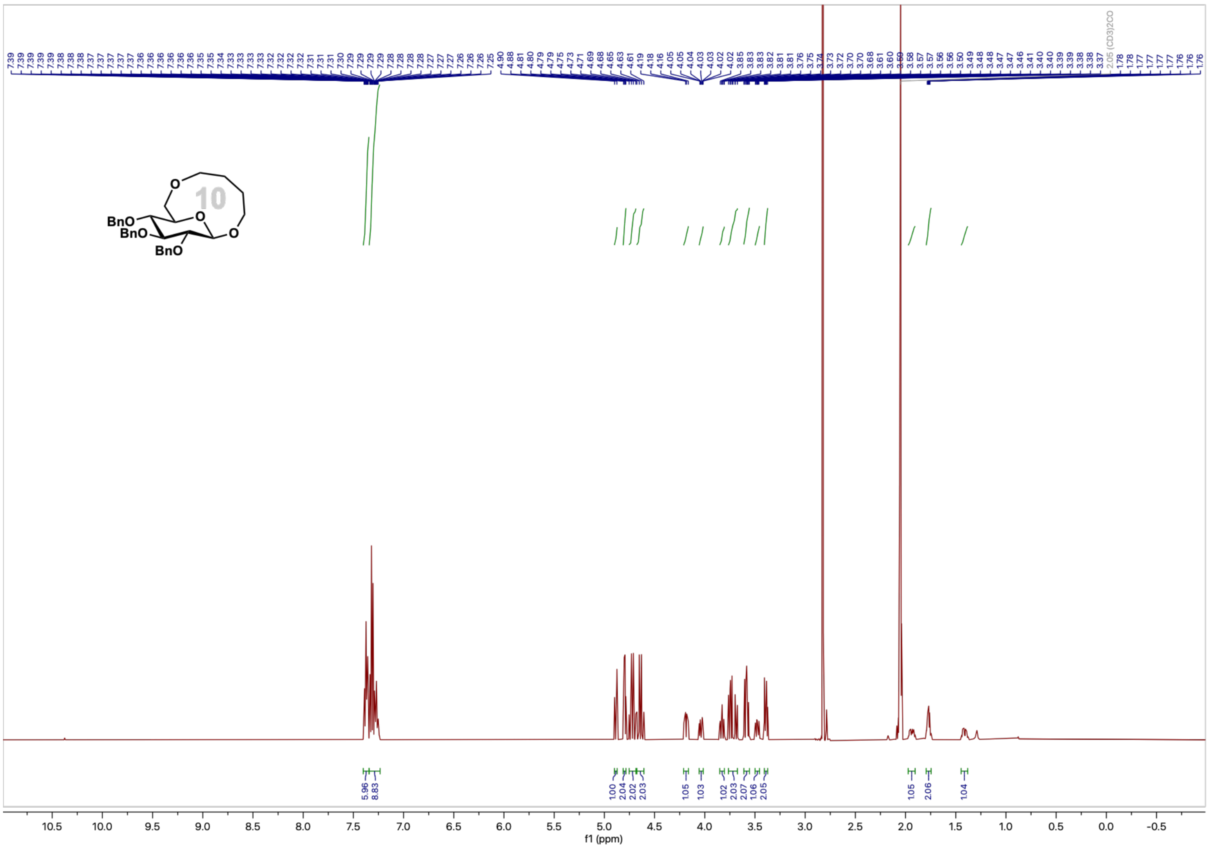

^{13}C NMR (126 MHz, acetone-d_6, 298K) of compound **4**

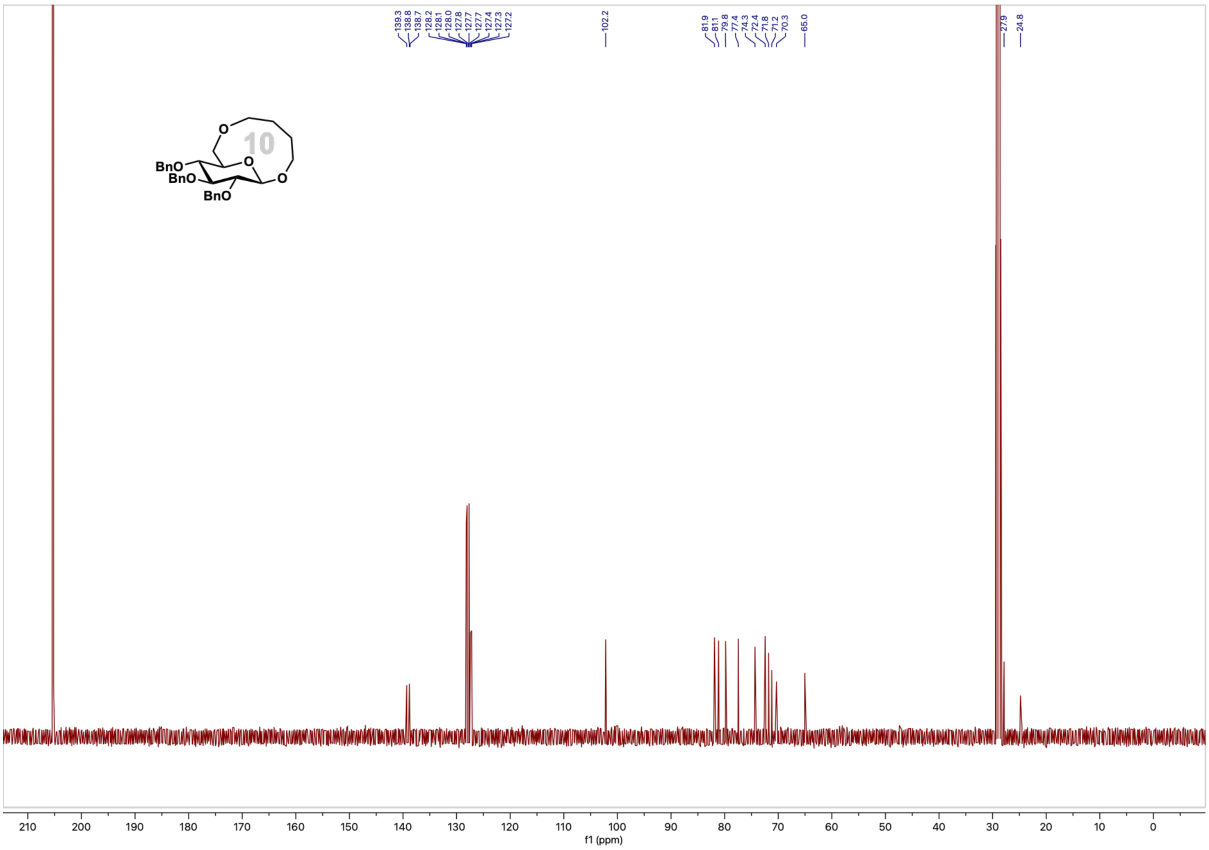

^{1}H NMR (500 MHz, acetone-d_6, 298K) of compound **5**

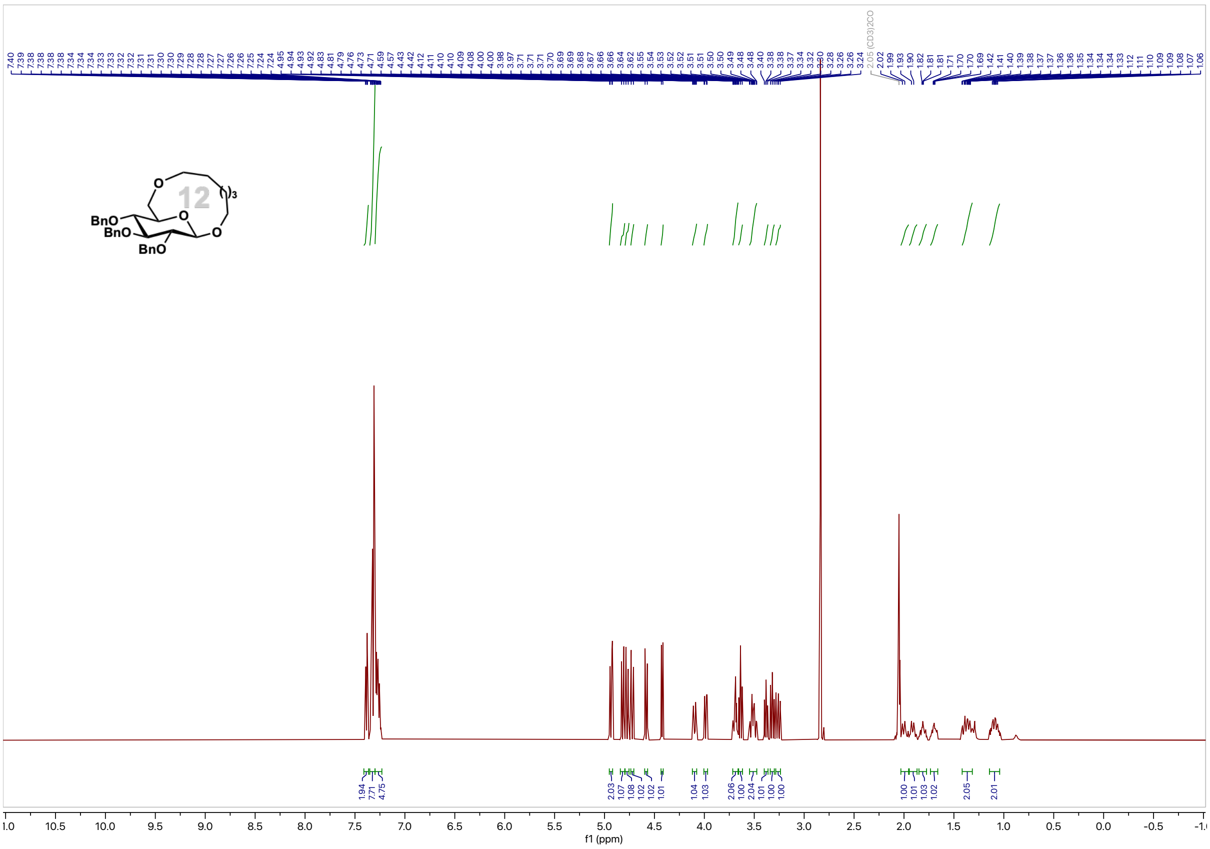

^{13}C NMR (126 MHz, acetone-d_6, 298K) of compound **5**

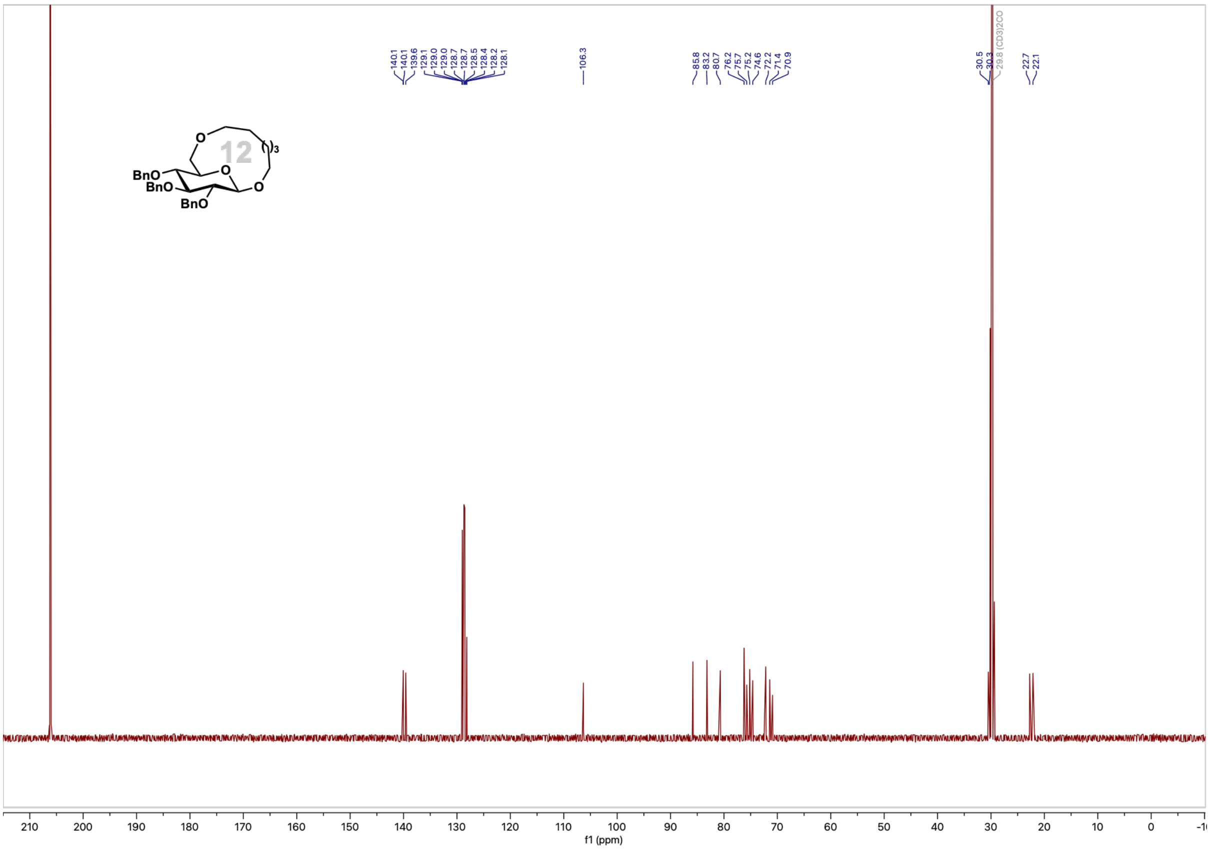

^{1}H NMR (500 MHz, acetone-d_6, 298K) of compound **6**

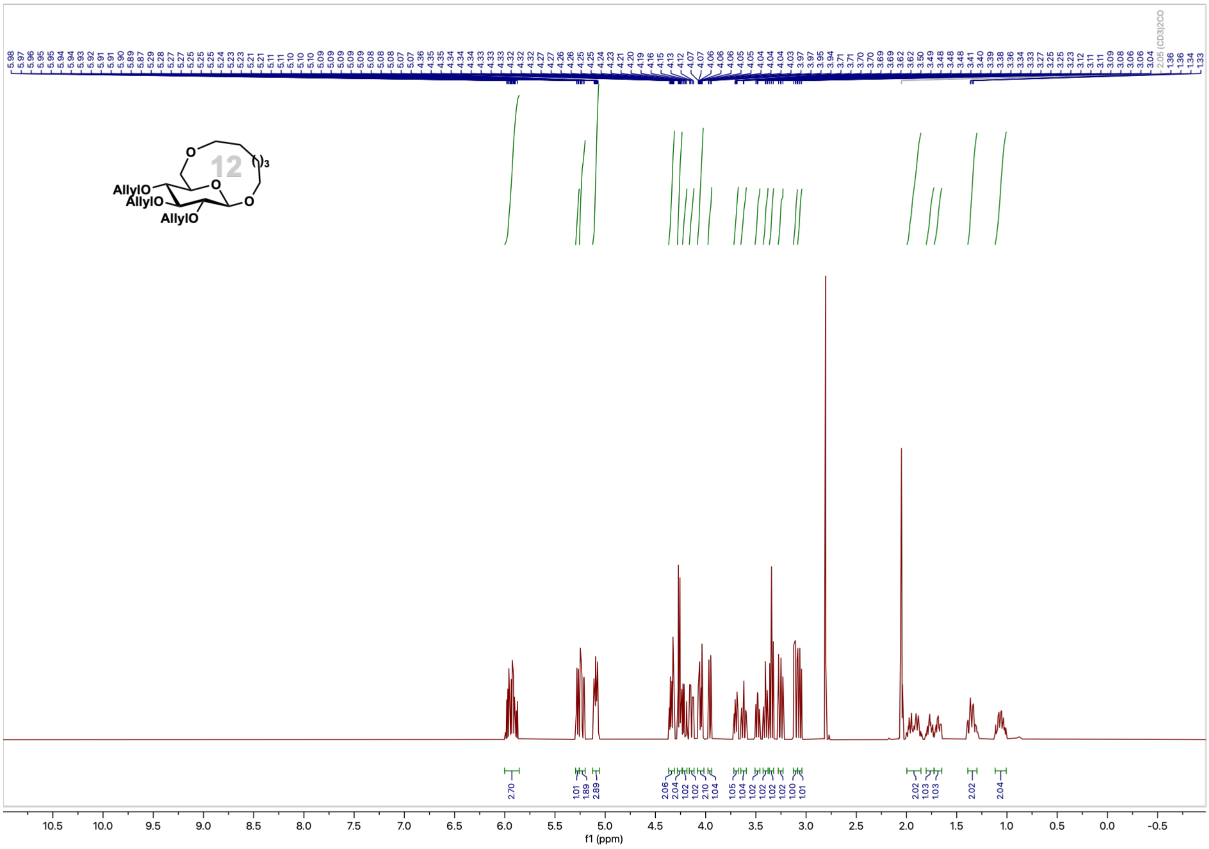

^{13}C NMR (126 MHz, acetone-d_6, 298K) of compound **6**

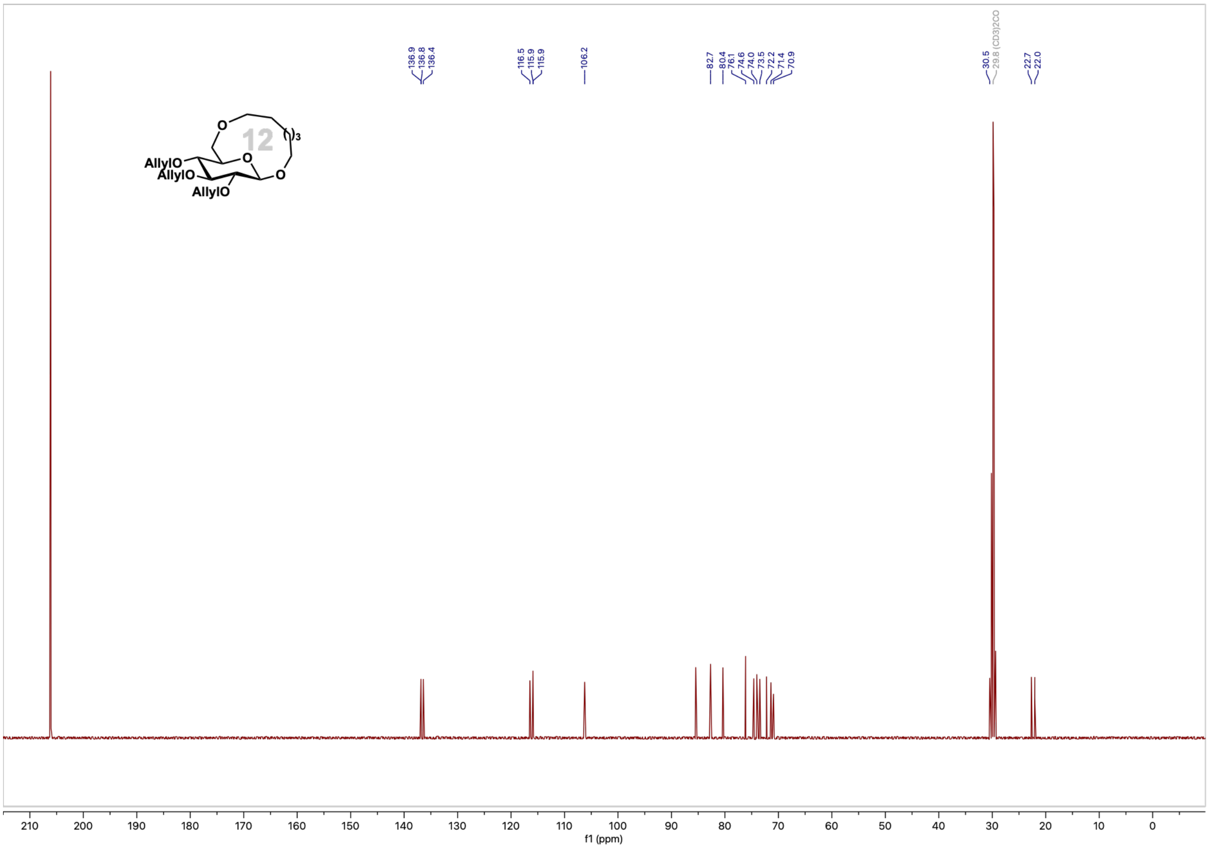

DEPT 135 spectra (126 MHz, acetone-d_6, 298K) of compound **6**

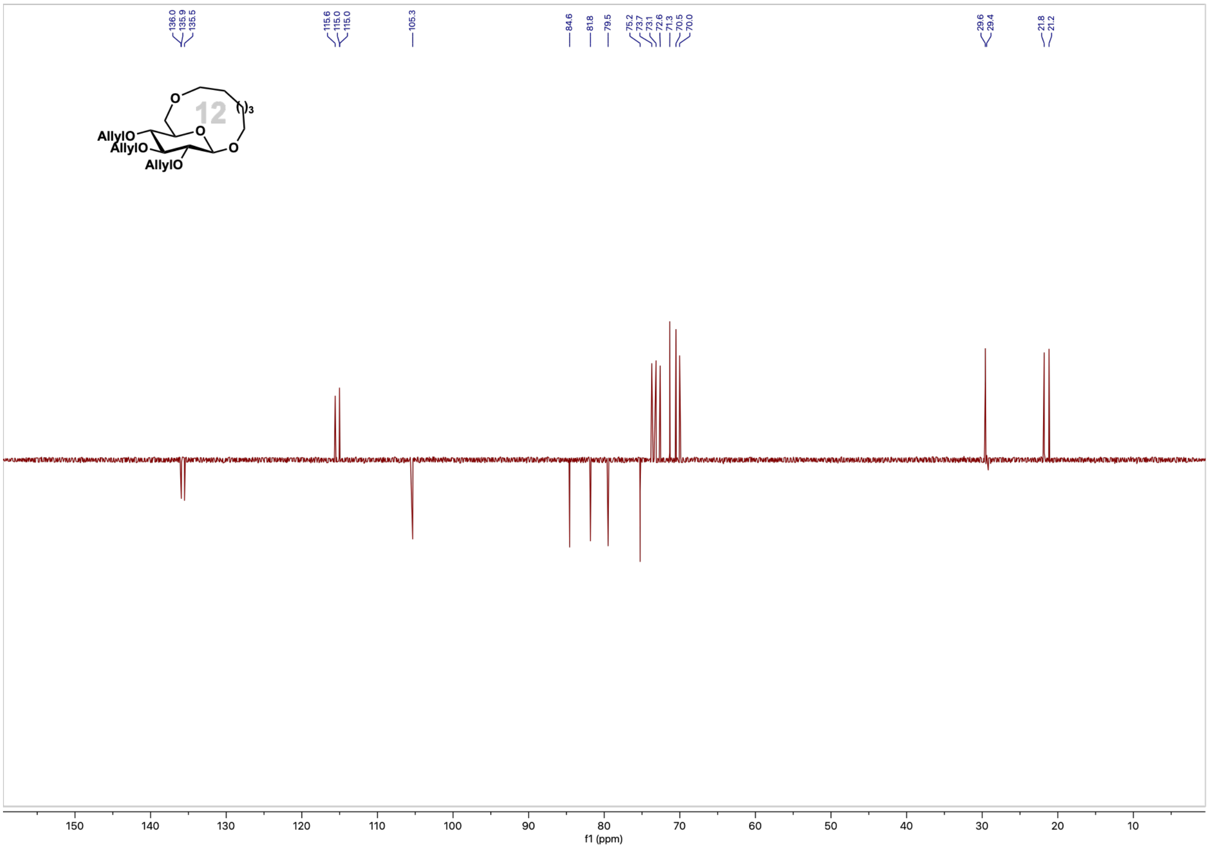

^{1}H NMR (500 MHz, acetone-d_6, 298K) of compound **7**

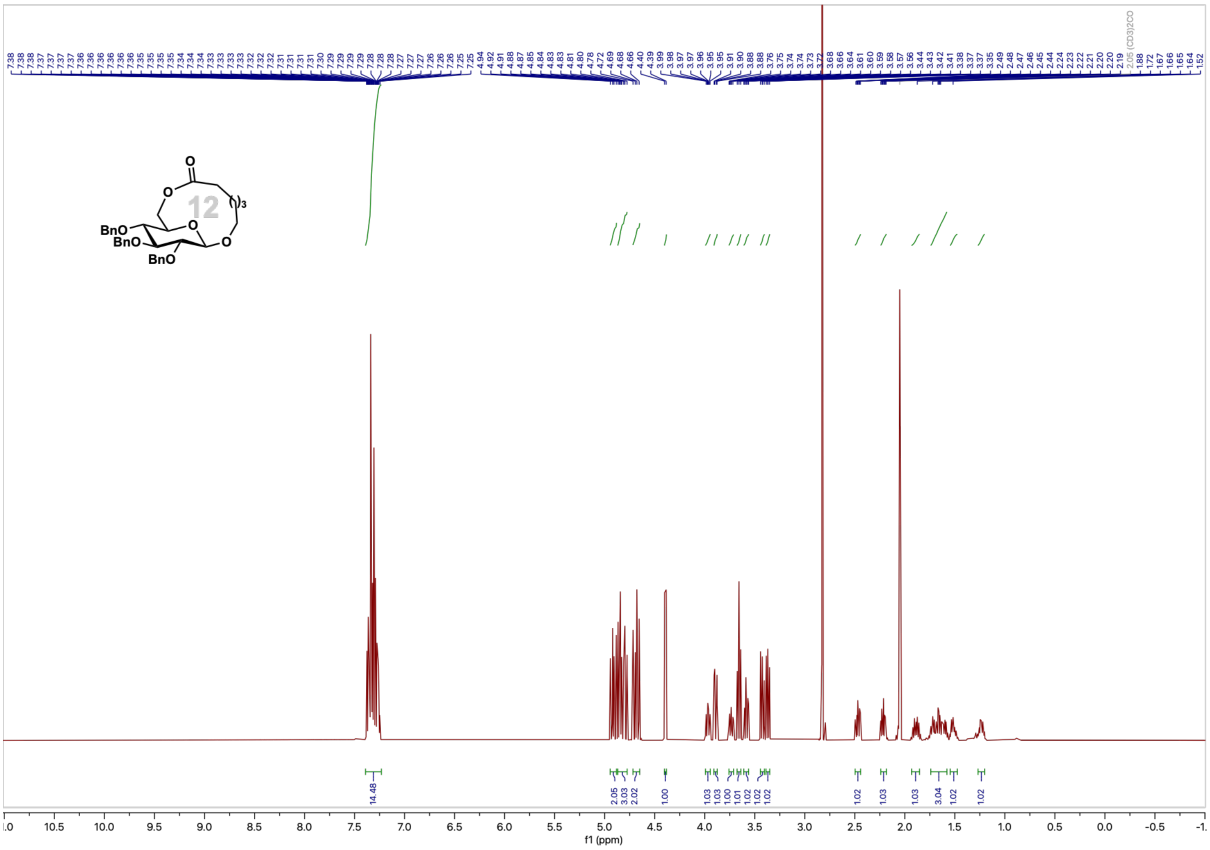

^{13}C NMR (126 MHz, acetone-d_6, 298K) of compound **7**

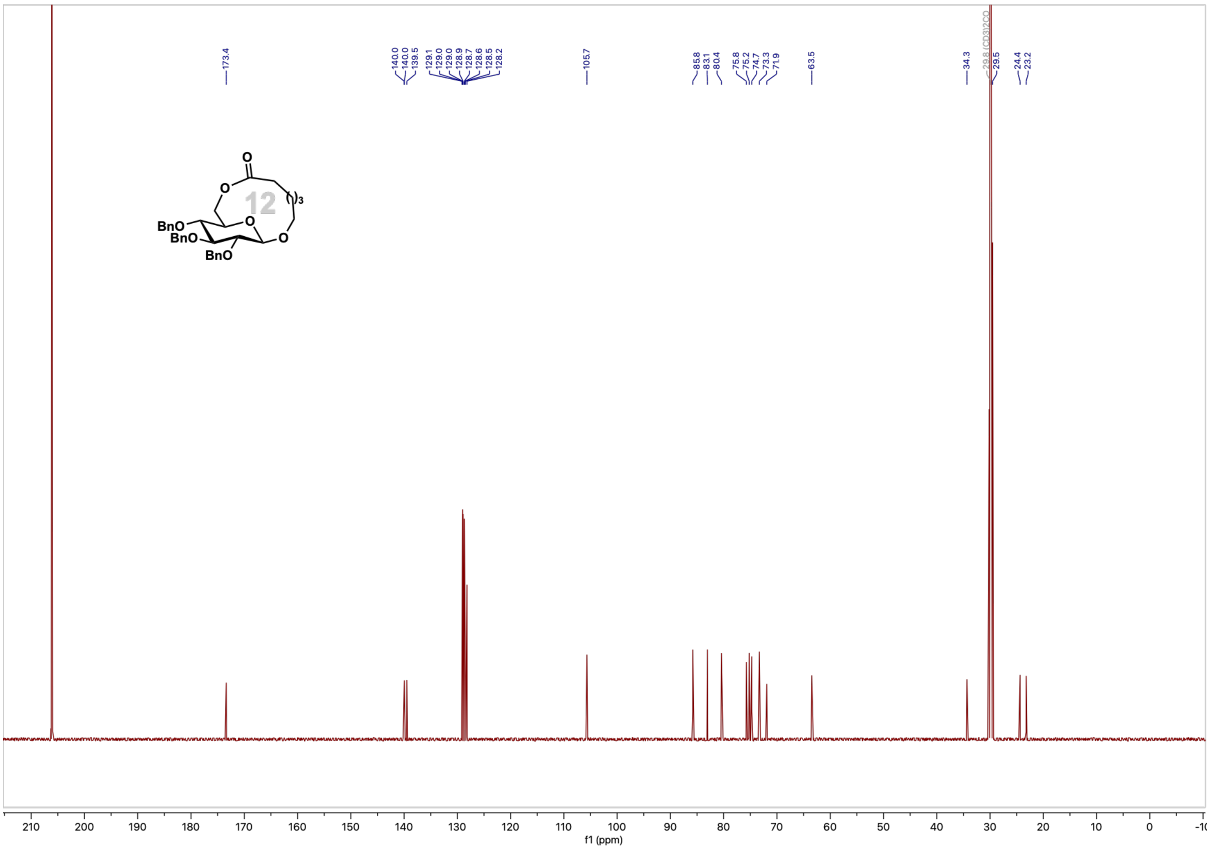

^{1}H NMR (500 MHz, acetone-d_6, 298K) of compound **8**

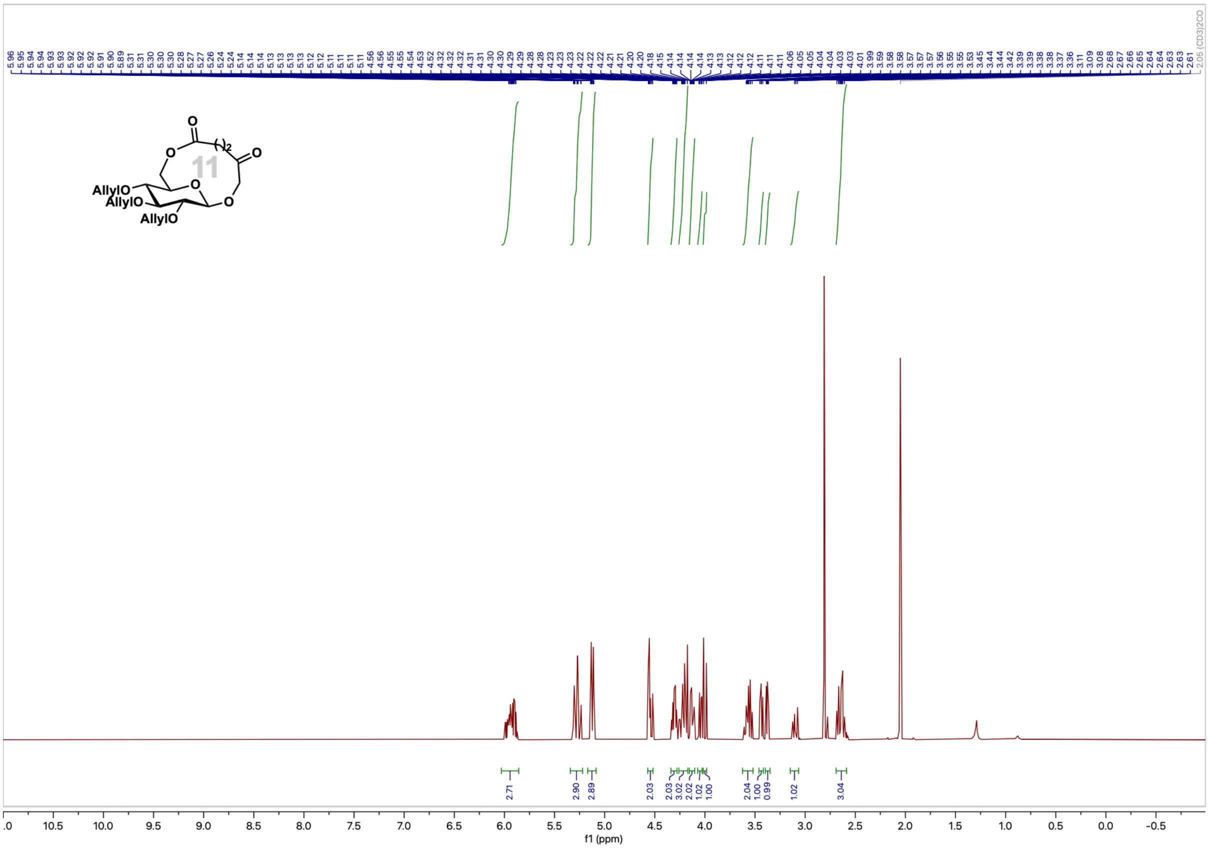

^{13}C NMR (126 MHz, acetone-d_6, 298K) of compound **8**

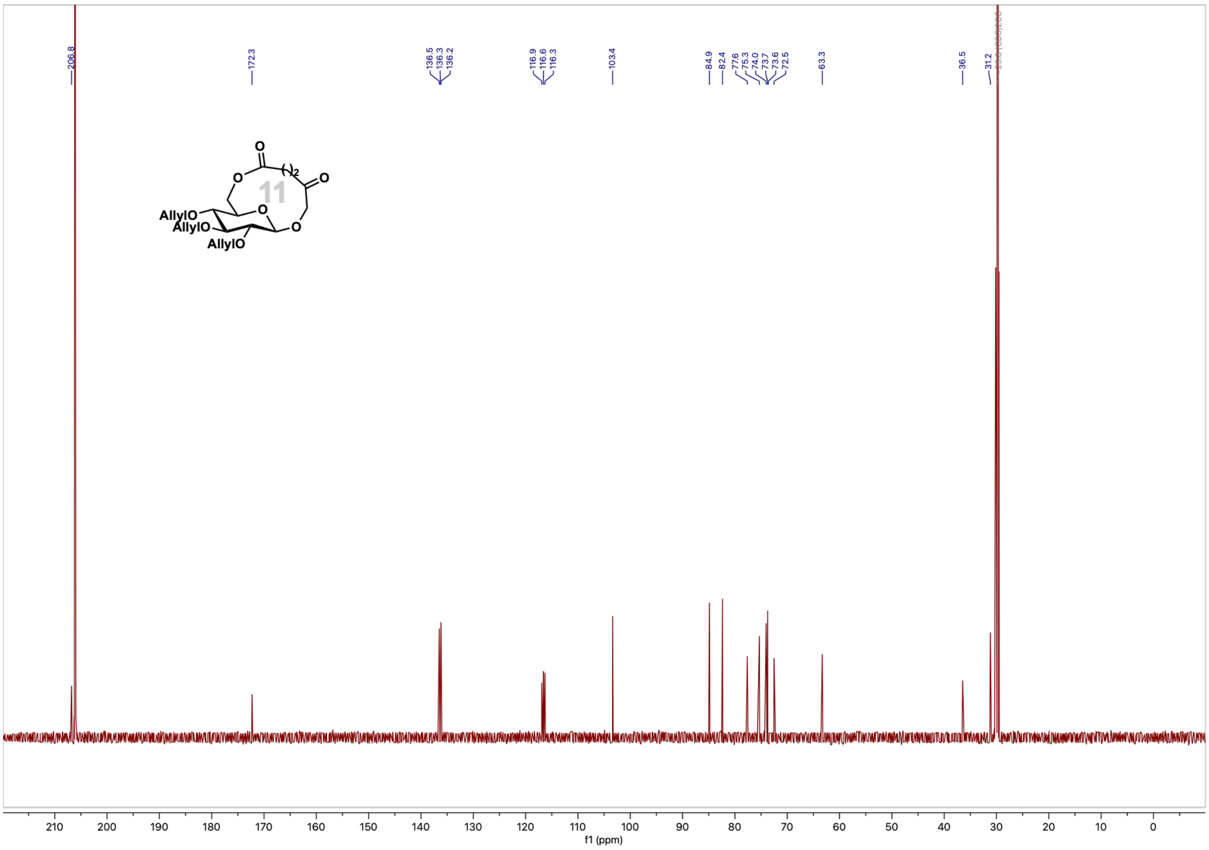

^{1}H NMR (500 MHz, $CDCl_3$, 298K) of compound **S3**

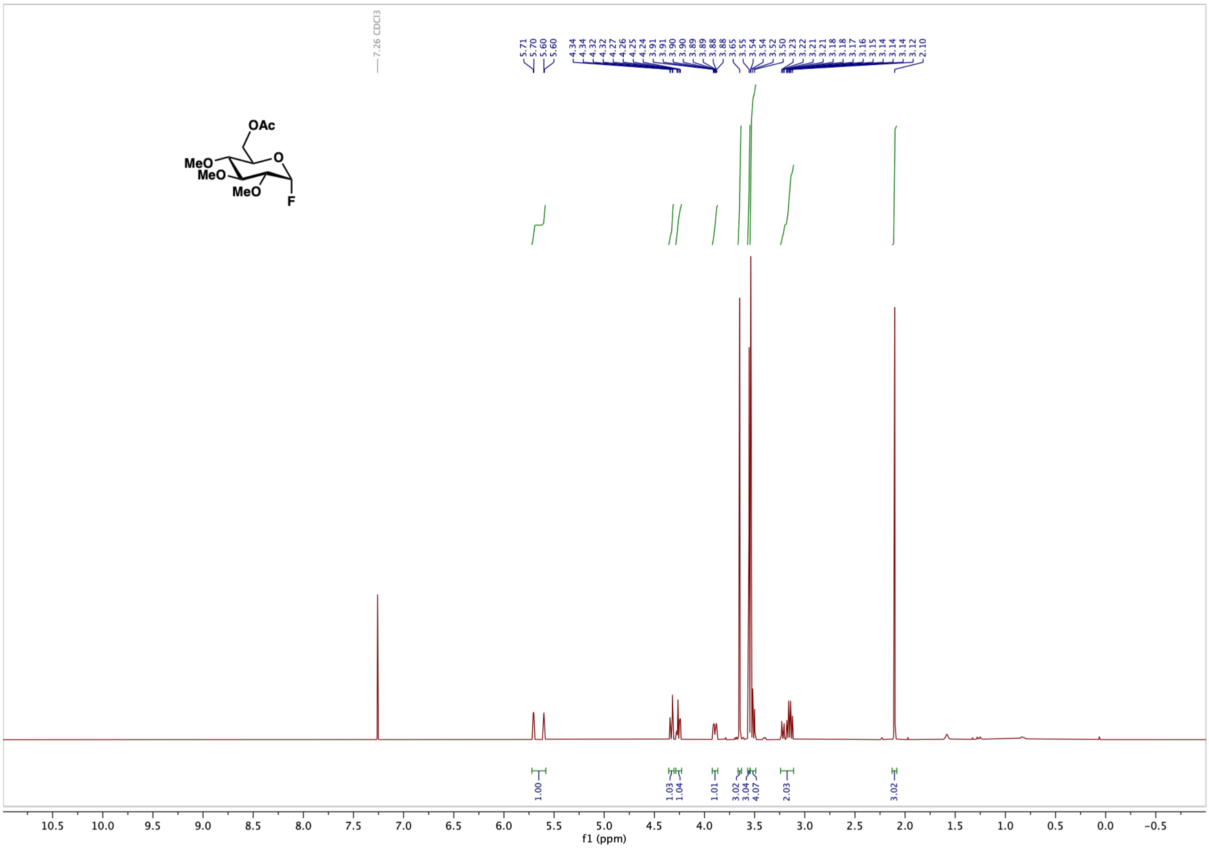

^{19}F NMR (470 MHz, $CDCl_3$, 298K) of compound **S3**

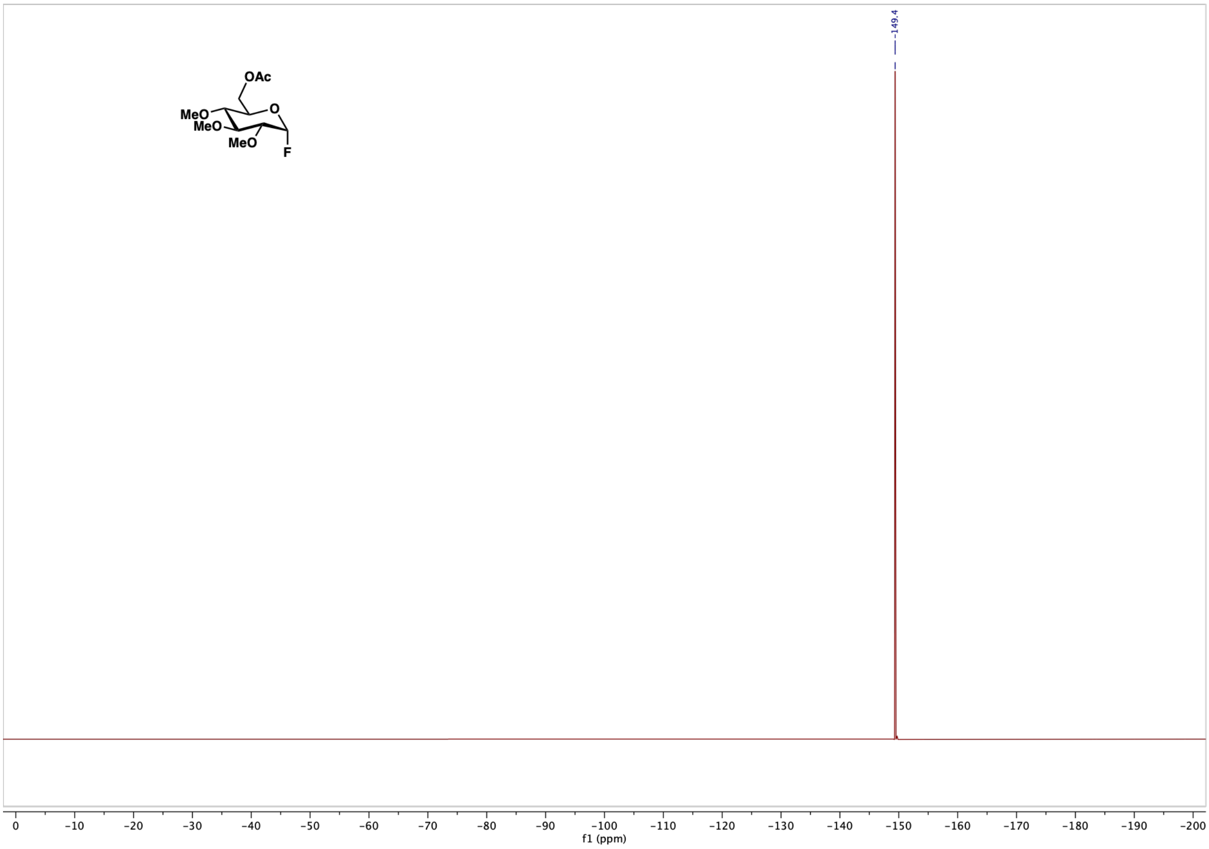

^{13}C NMR (126 MHz, $CDCl_3$, 298K) of compound **S3**

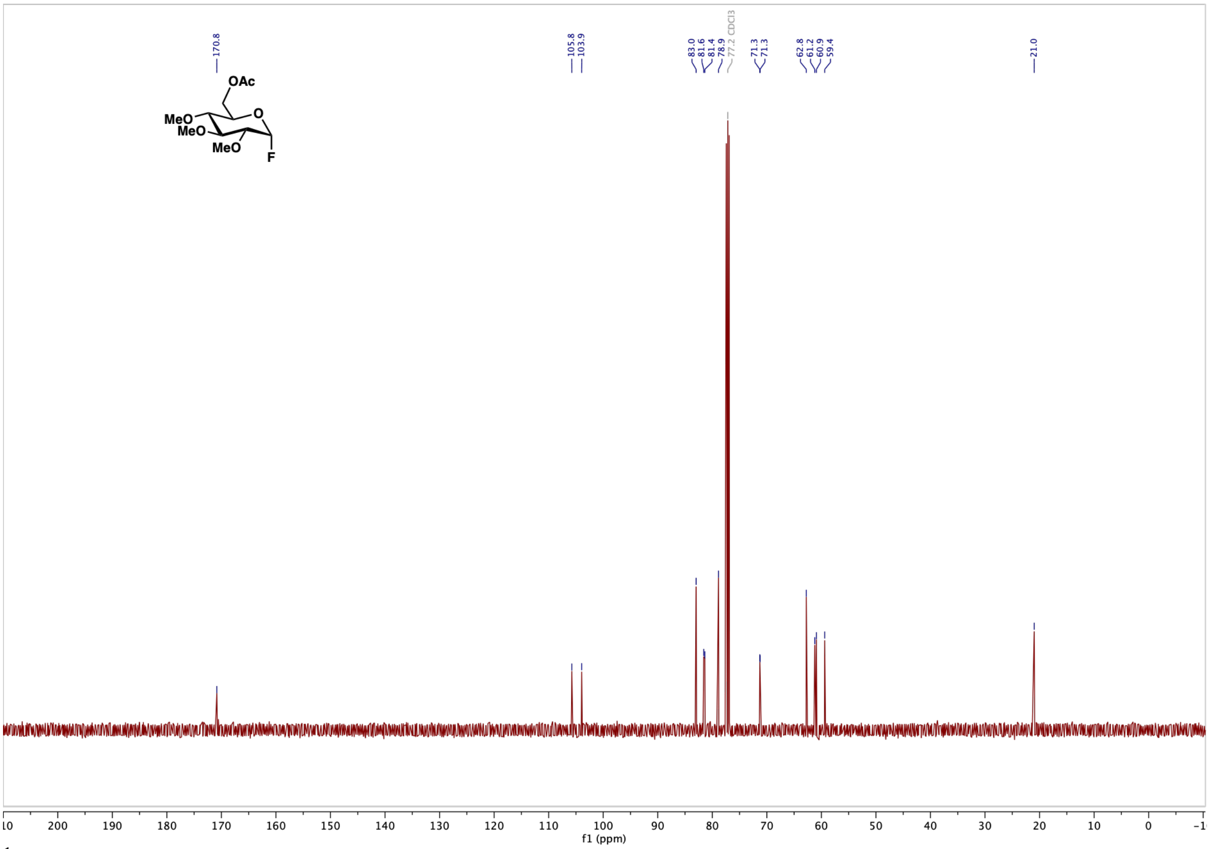

^{1}H NMR (500 MHz, $CDCl_3$, 298K) of compound **S4**

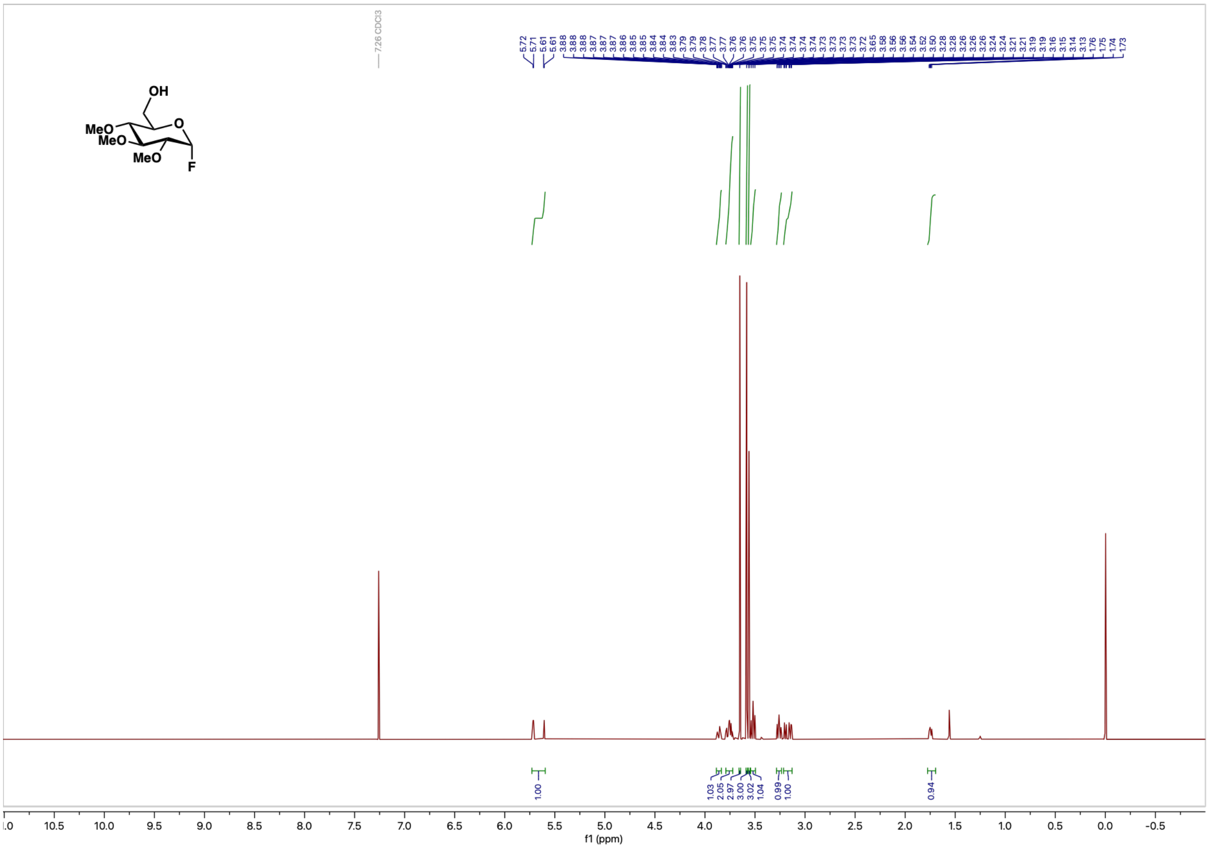

^{19}F NMR (470 MHz, $CDCl_3$, 298K) of compound **S4**

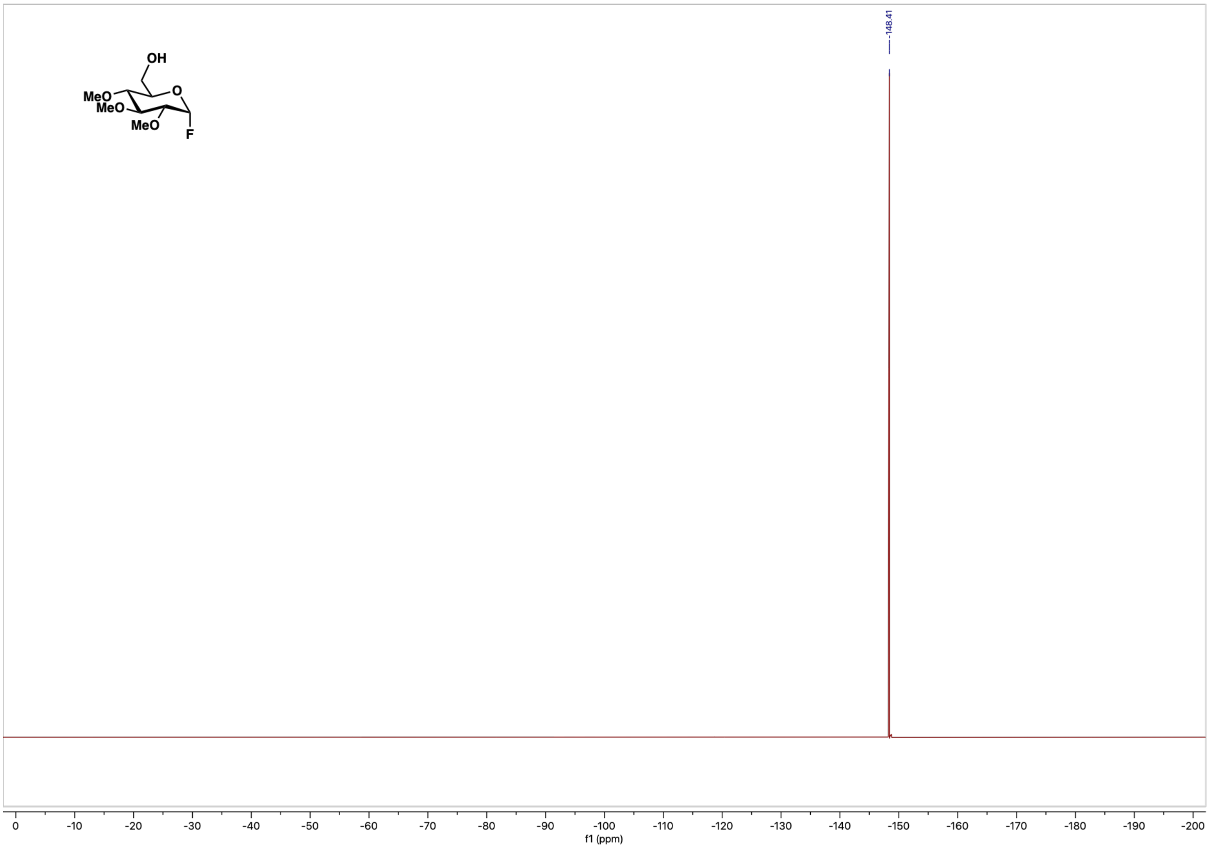

^{13}C NMR (126 MHz, $CDCl_3$, 298K) of compound **S4**

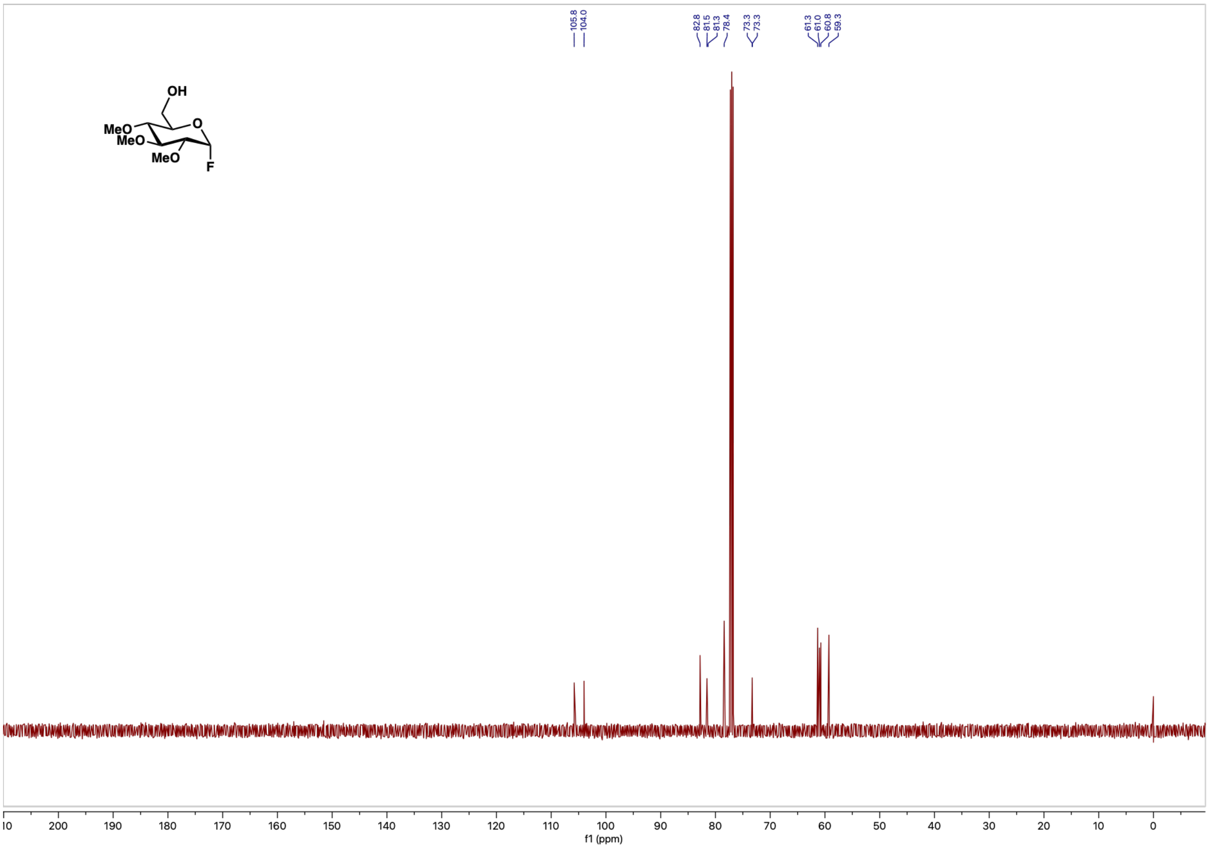

^{1}H NMR (500 MHz, $CDCl_3$, 298K) of compound **S5a**

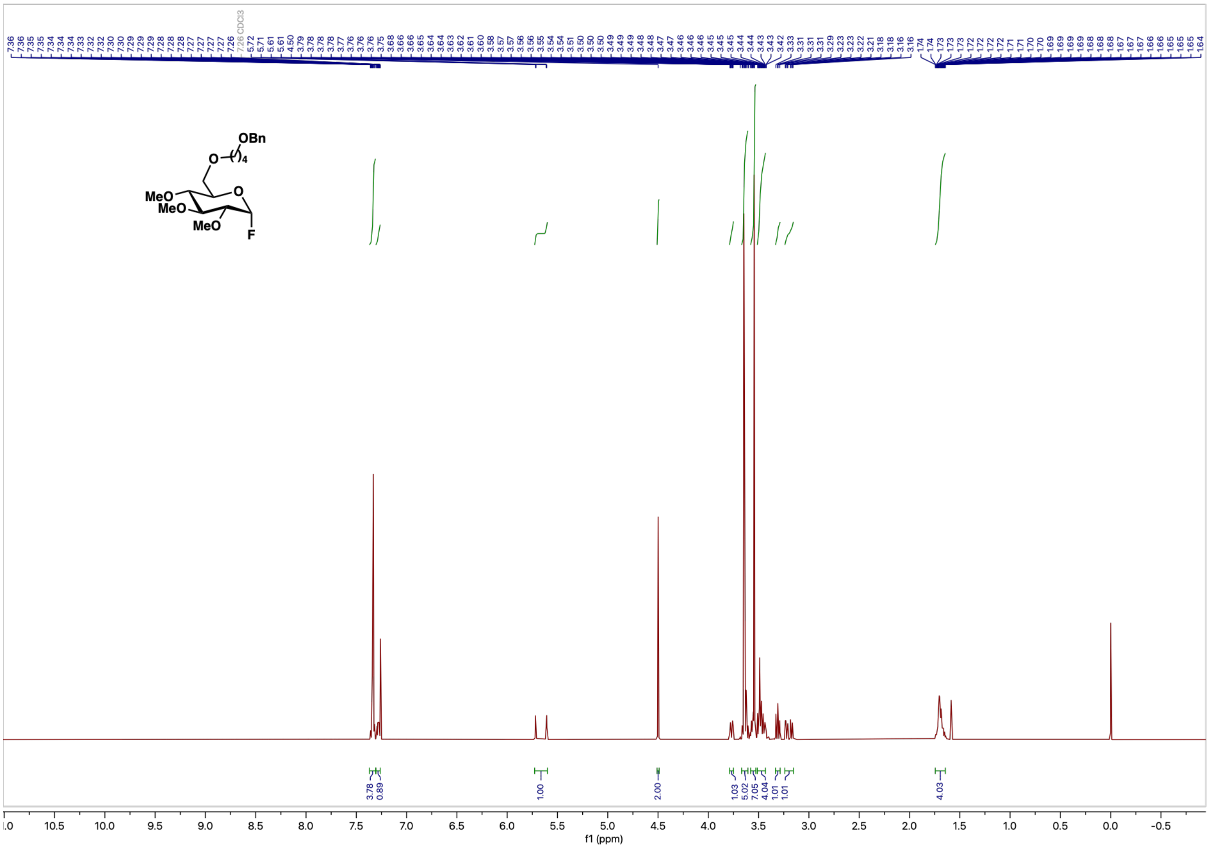

^{19}F NMR (470 MHz, $CDCl_3$, 298K) of compound **S5a**

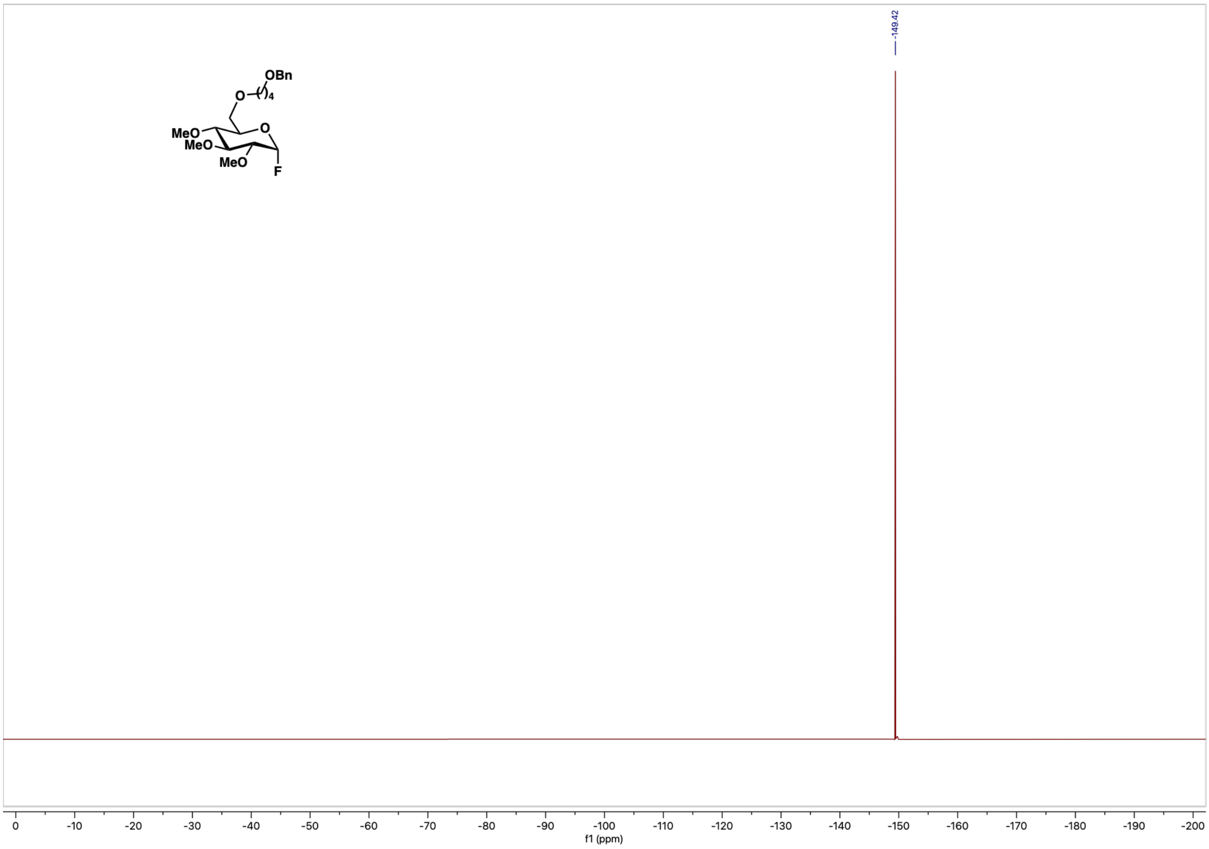

^{13}C NMR (126 MHz, $CDCl_3$, 298K) of compound **S5a**

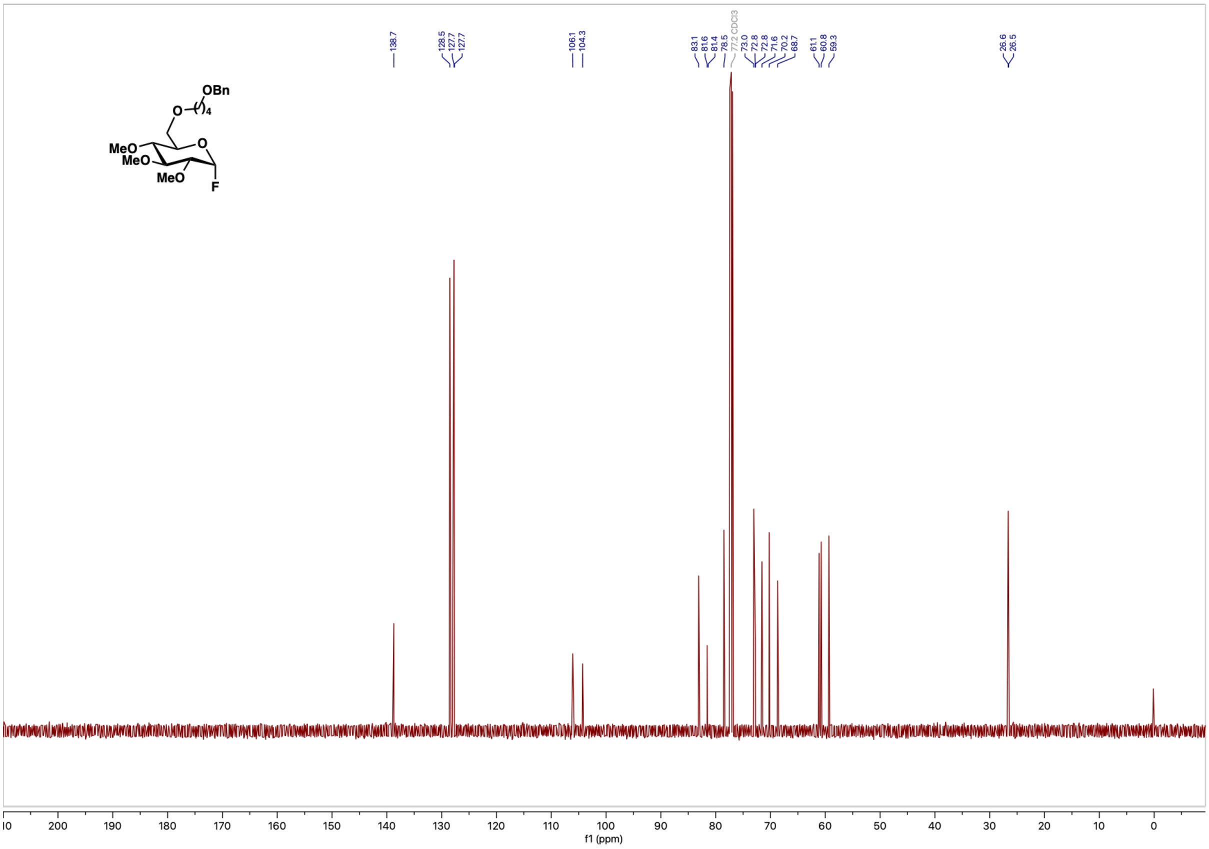

^{1}H NMR (500 MHz, $CDCl_3$, 298K) of compound **1a**

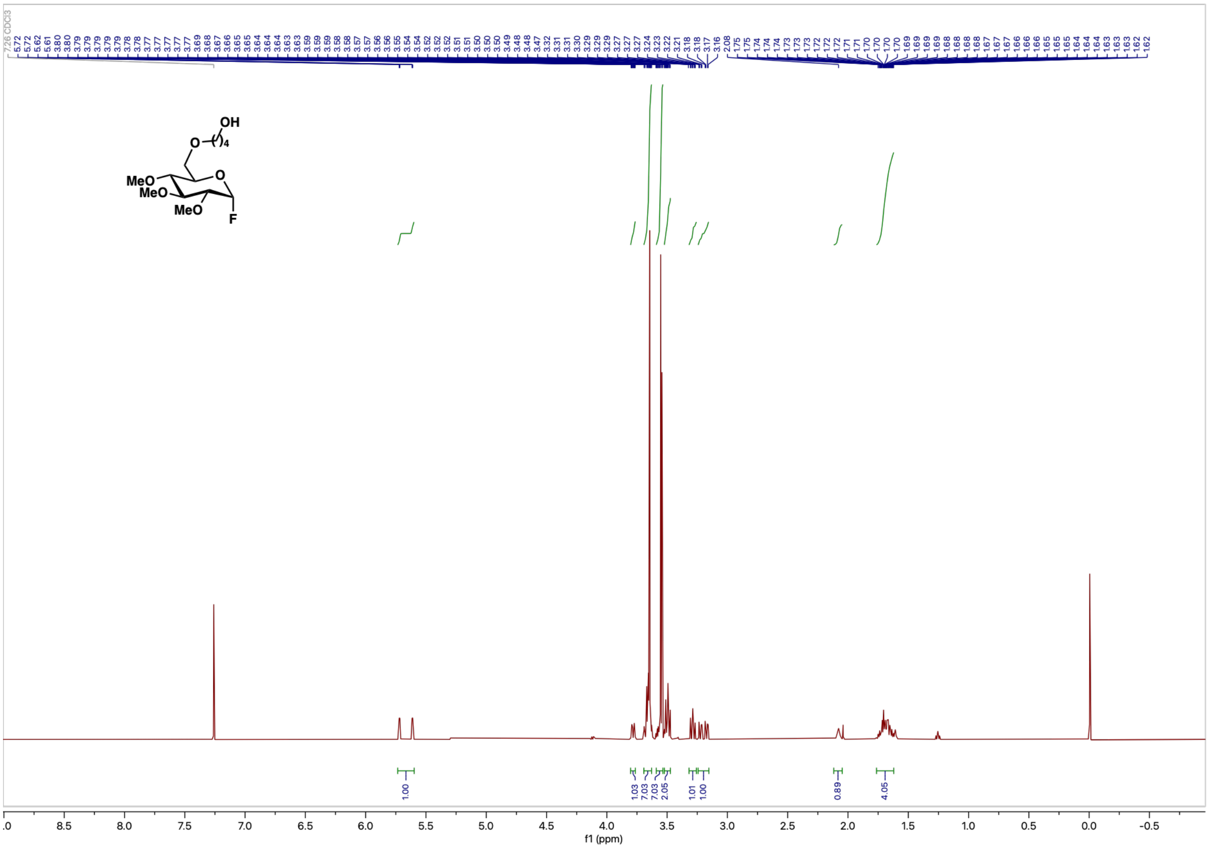

^{19}F NMR (470 MHz, $CDCl_3$, 298K) of compound **1a**

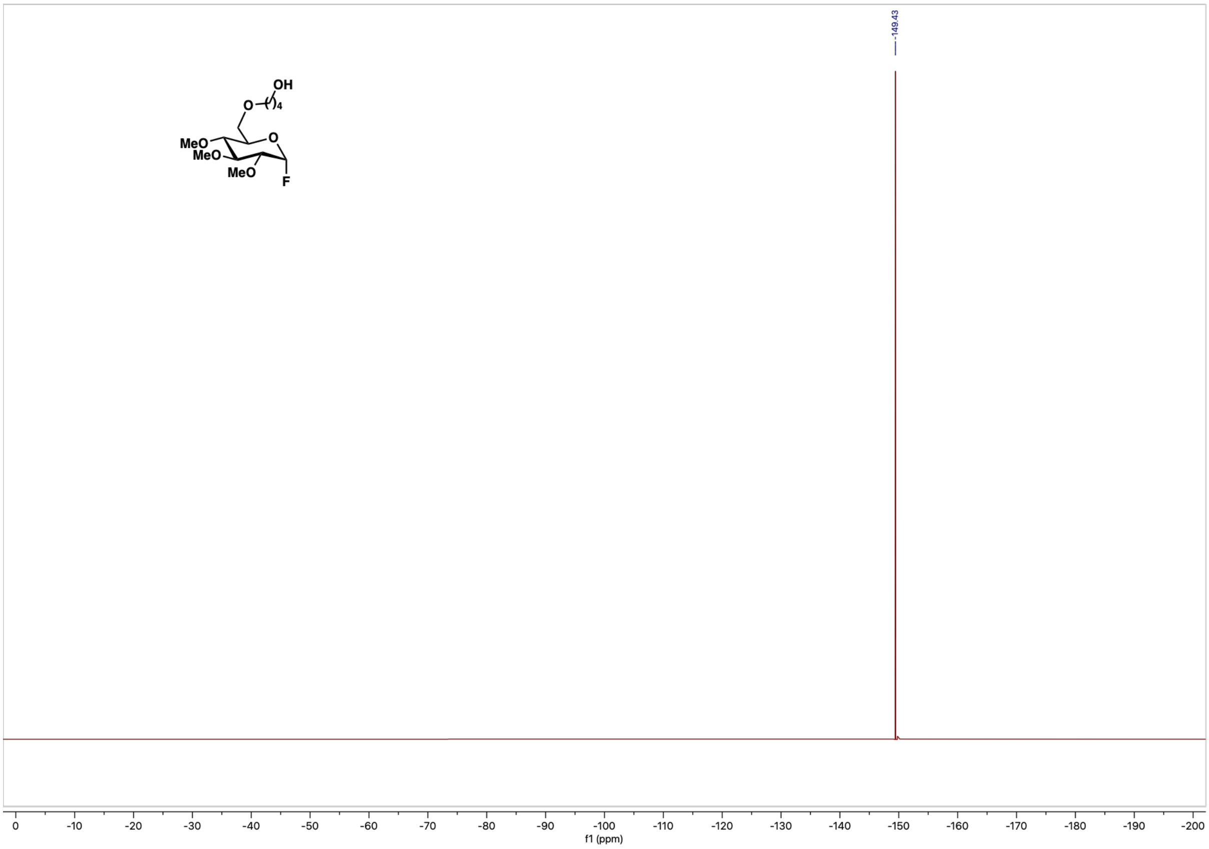

^{13}C NMR (126 MHz, $CDCl_3$, 298K) of compound **1a**

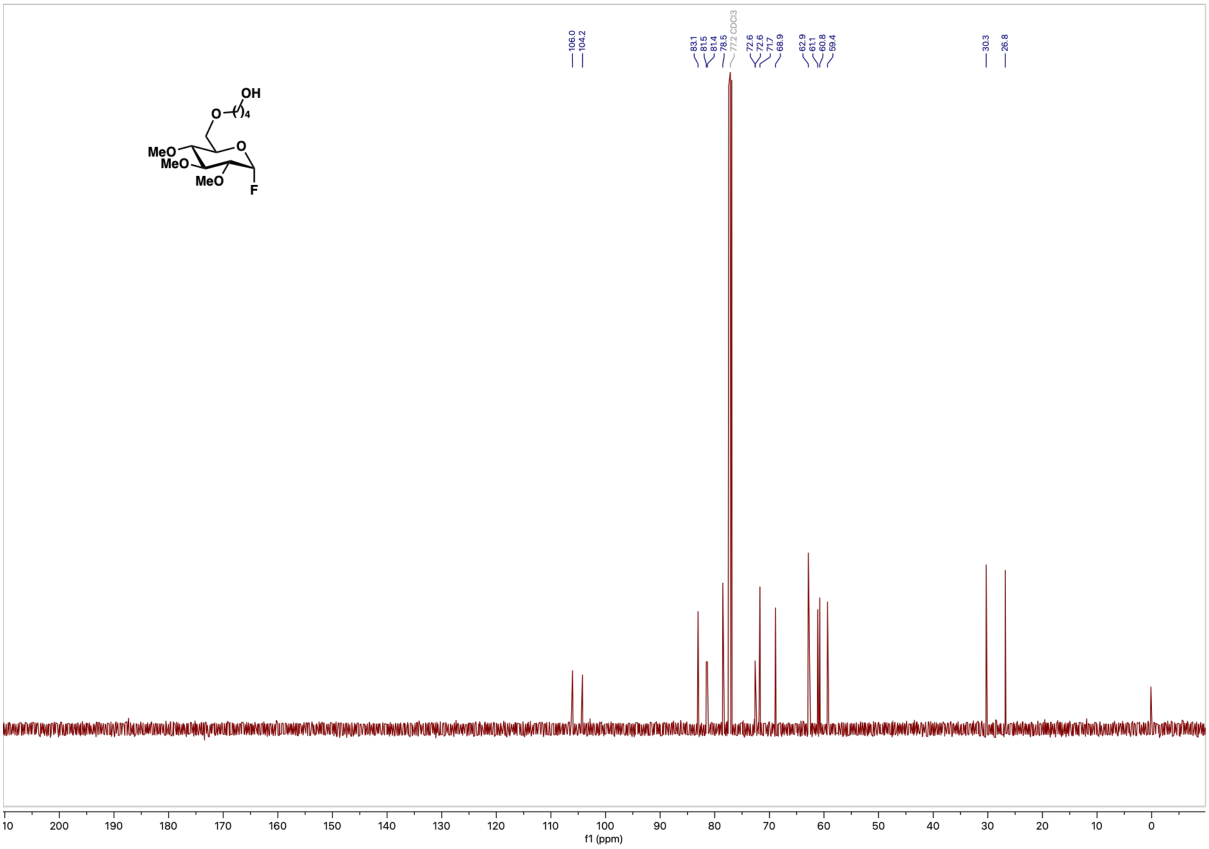

^{1}H NMR (500 MHz, $CDCl_3$, 298K) of compound **S5b**

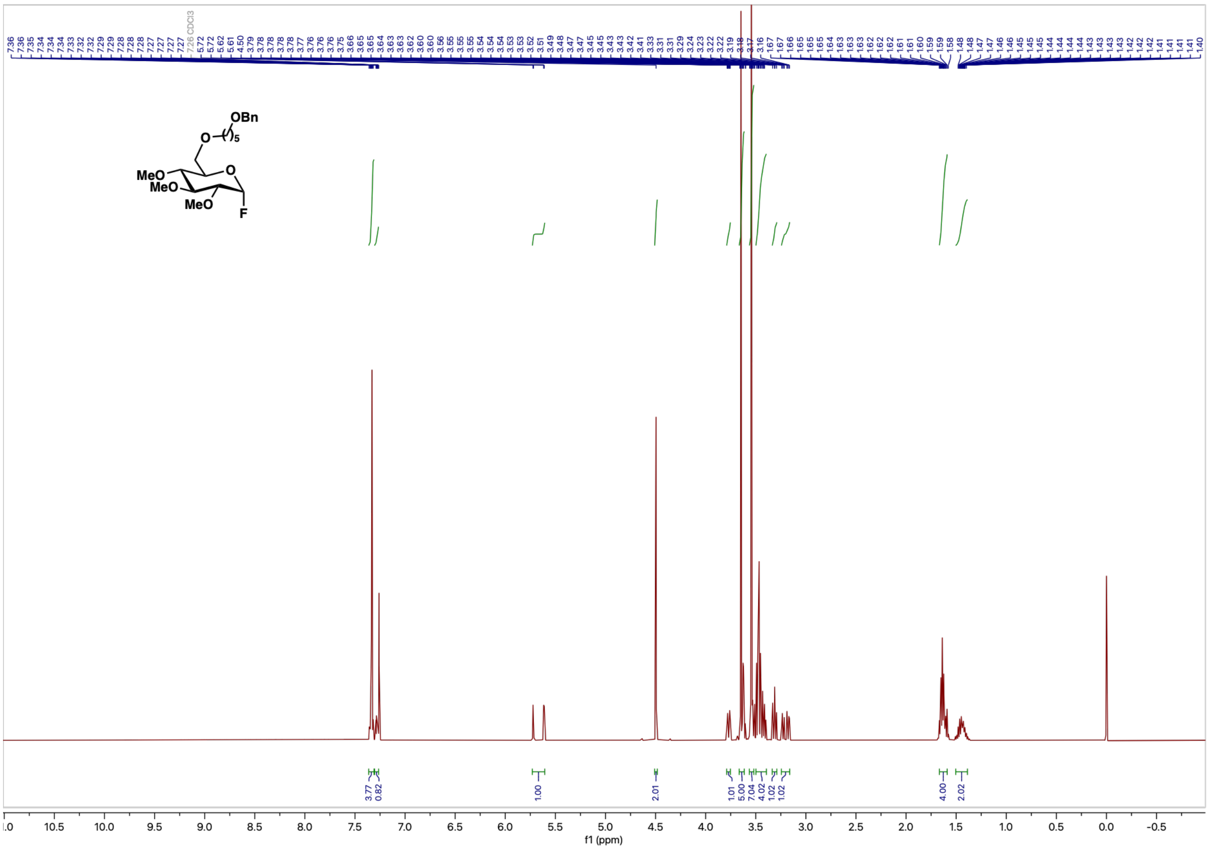

^{19}F NMR (470 MHz, $CDCl_3$, 298K) of compound **S5b**

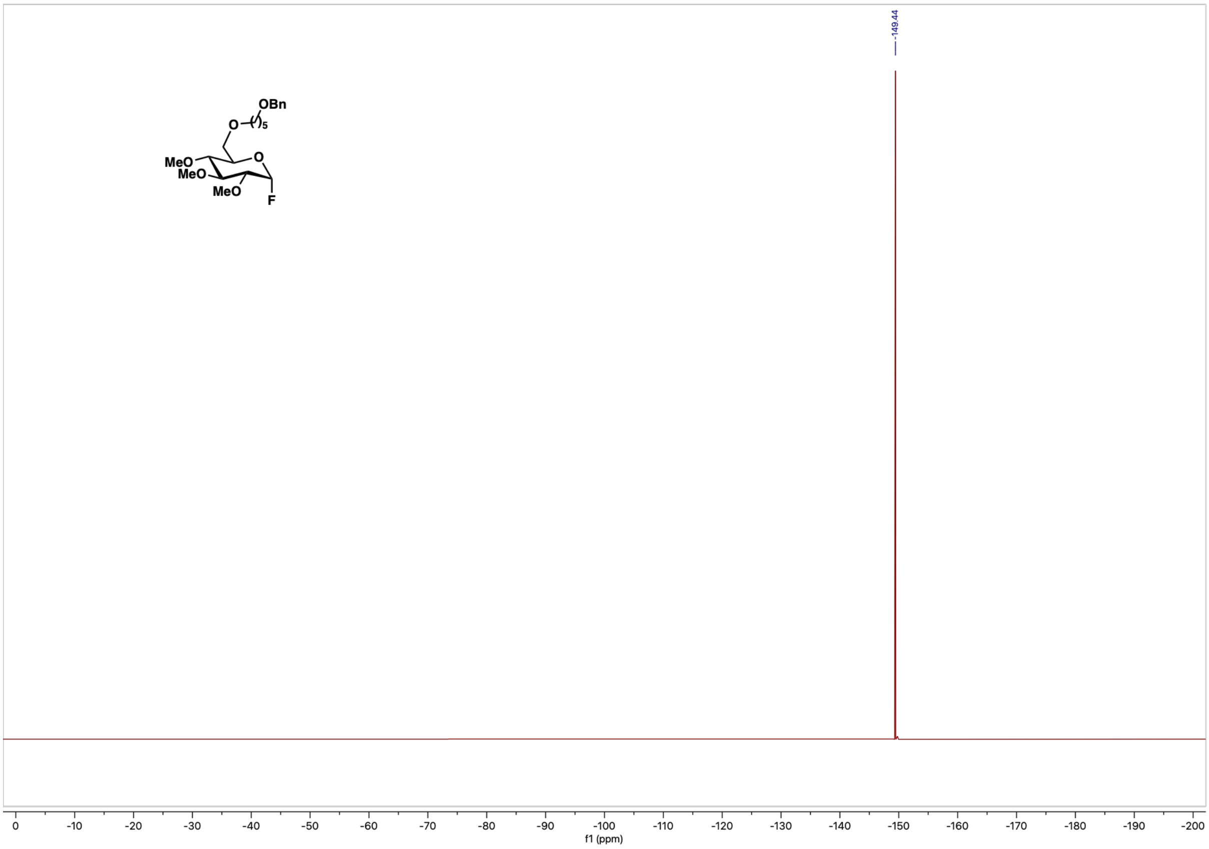

^{13}C NMR (126 MHz, $CDCl_3$, 298K) of compound **S5b**

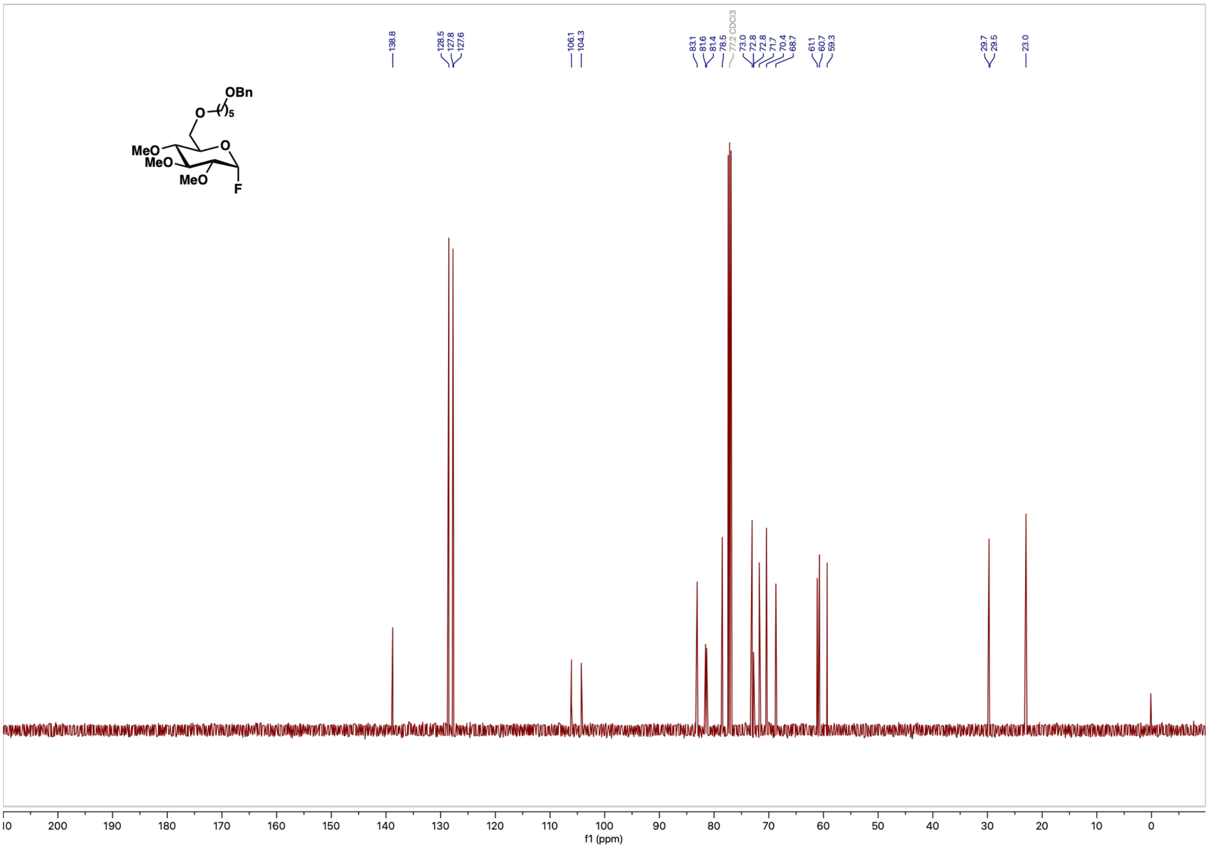

^{1}H NMR (500 MHz, $CDCl_3$, 298K) of compound **1b**

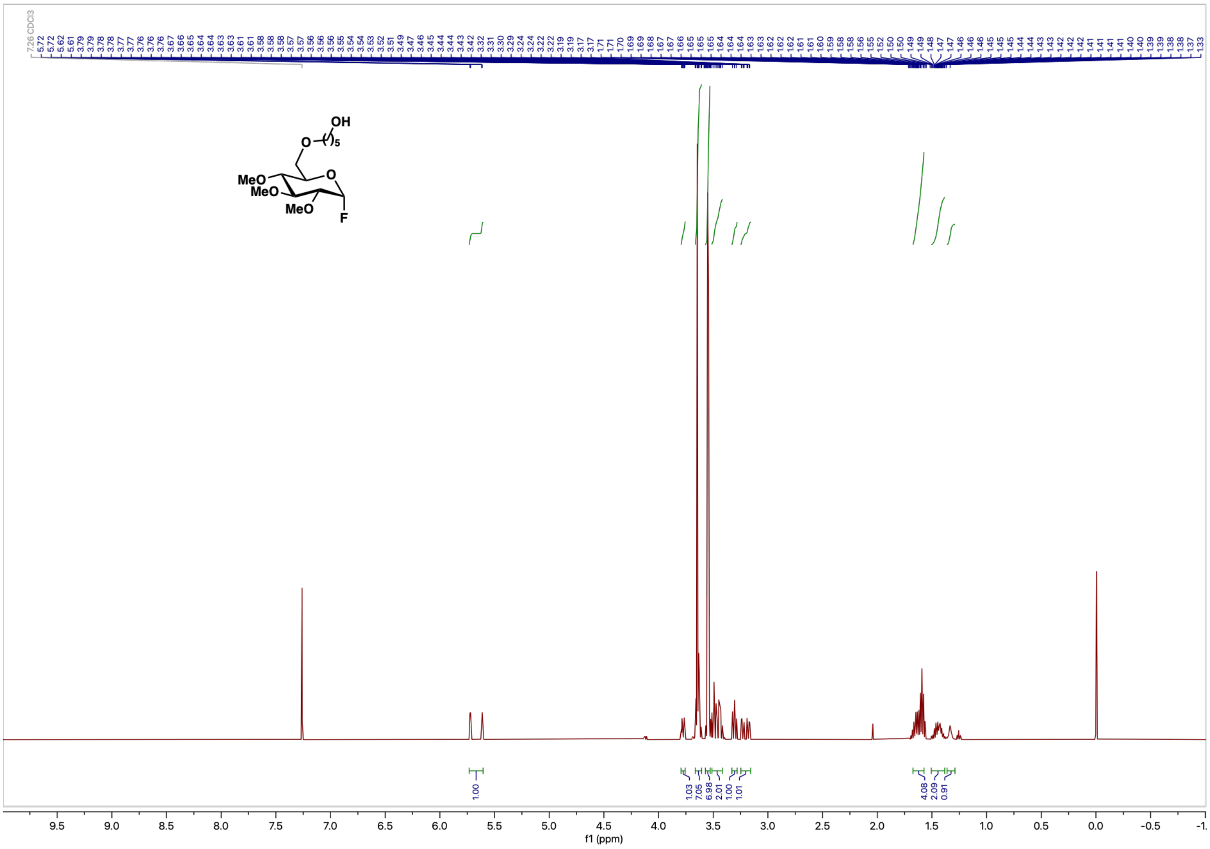

^{19}F NMR (470 MHz, $CDCl_3$, 298K) of compound **1b**

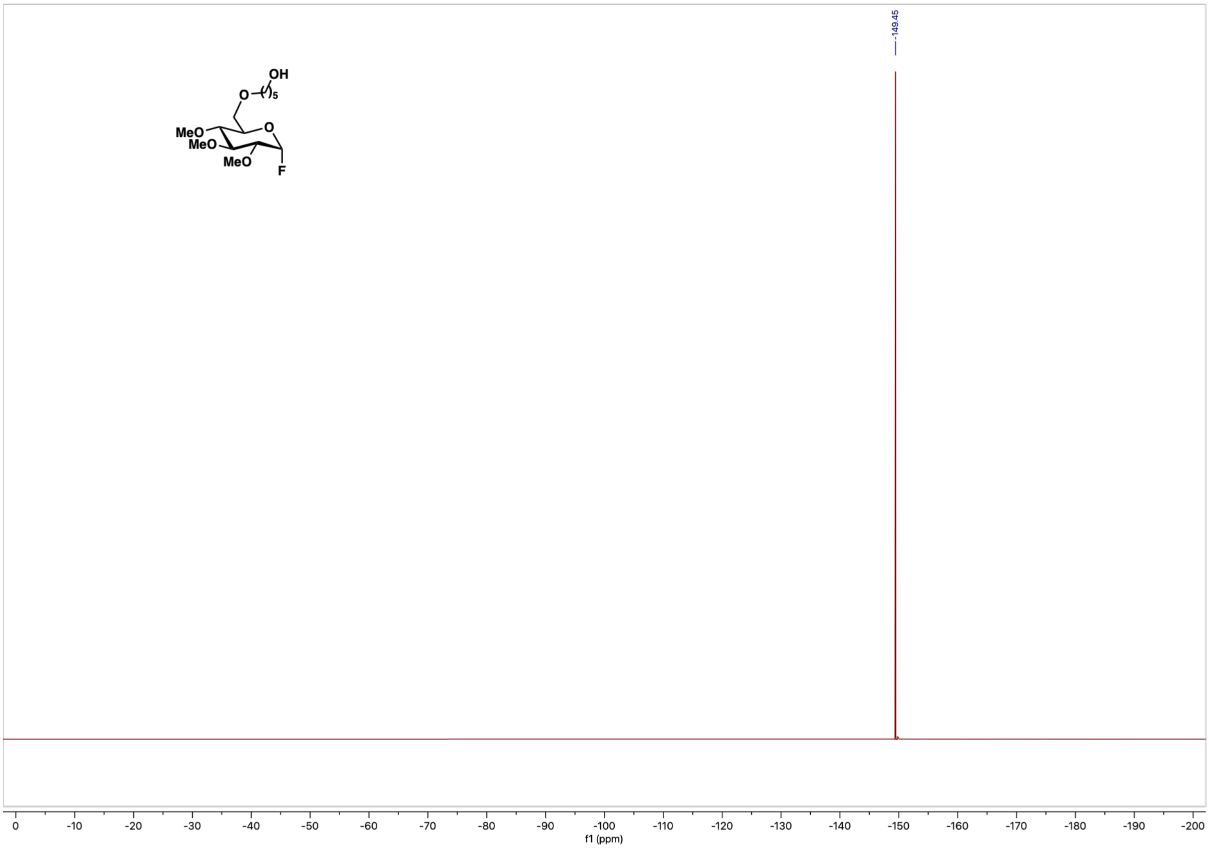

^{13}C NMR (126 MHz, $CDCl_3$, 298K) of compound **1b**

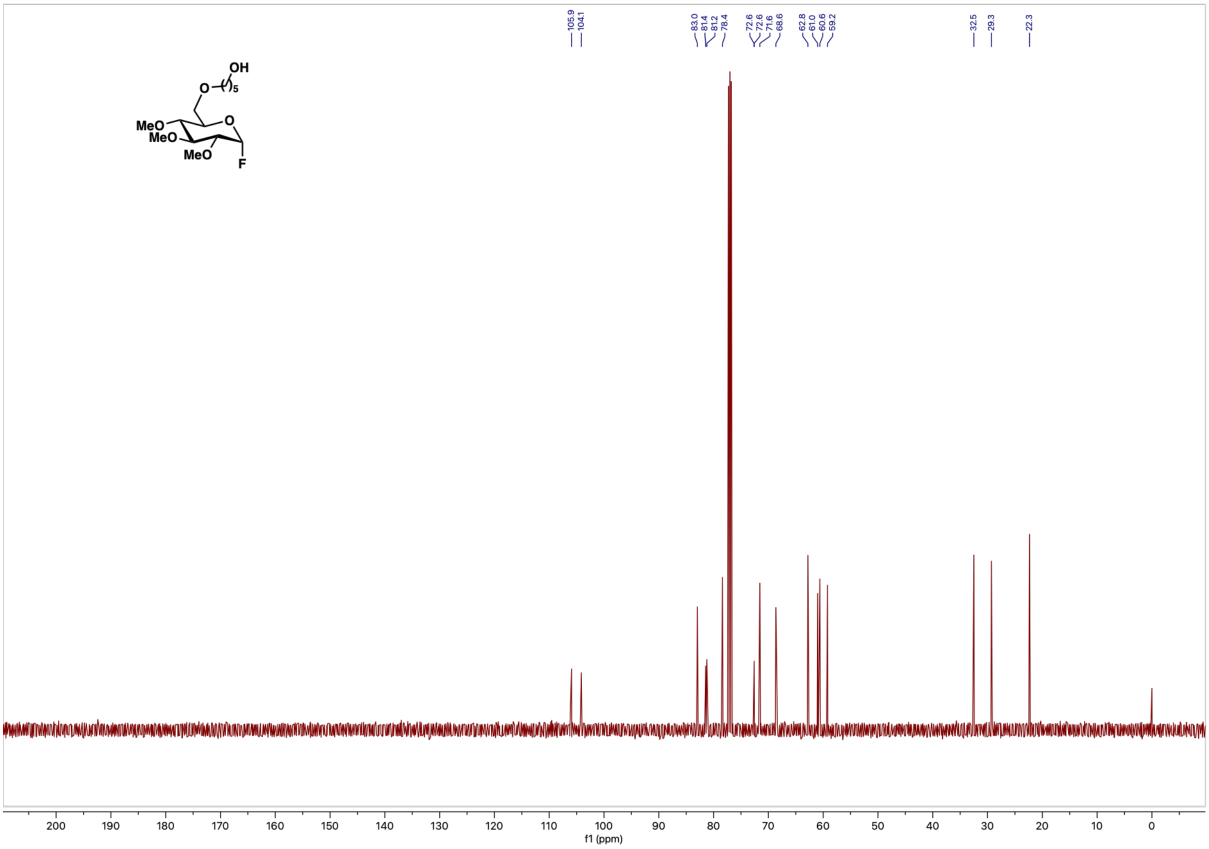

^{1}H NMR (500 MHz, $CDCl_3$, 298K) of compound **S5c**

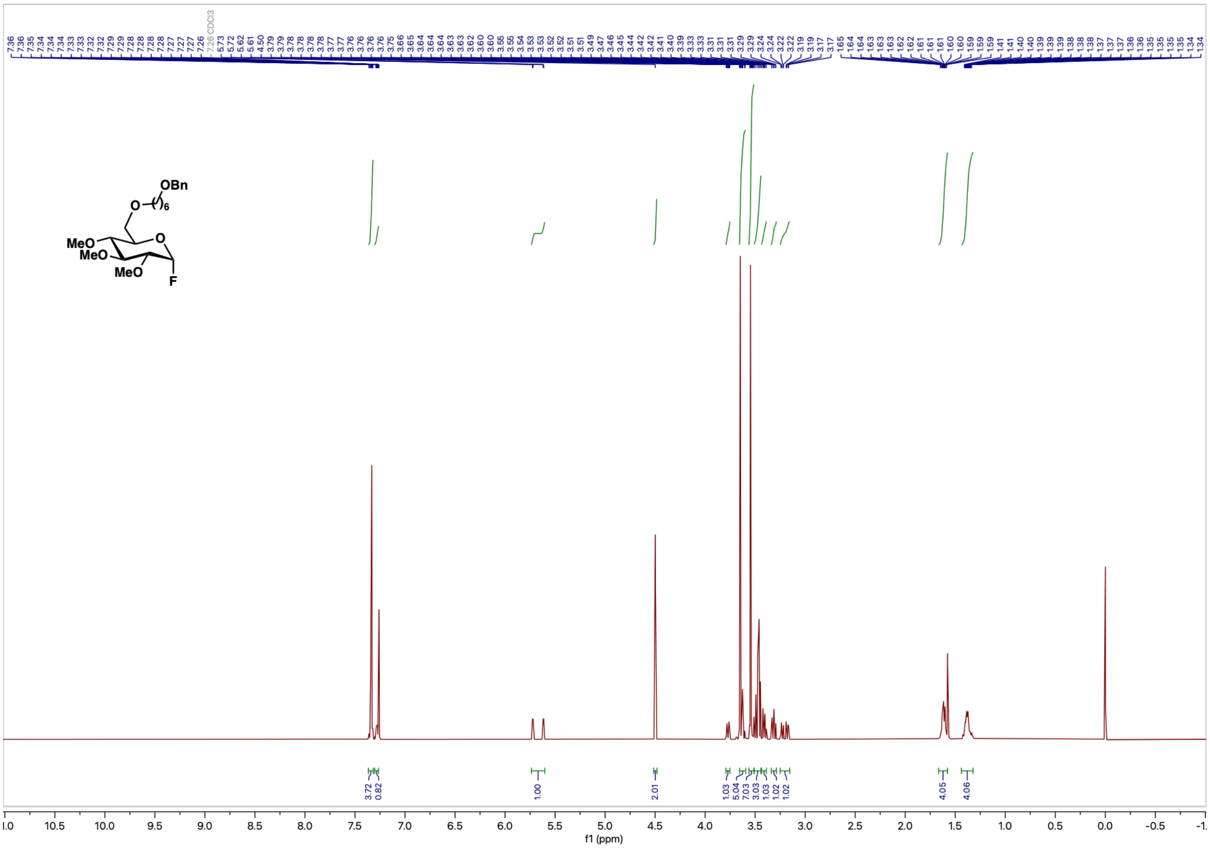

^{19}F NMR (470 MHz, $CDCl_3$, 298K) of compound **S5c**

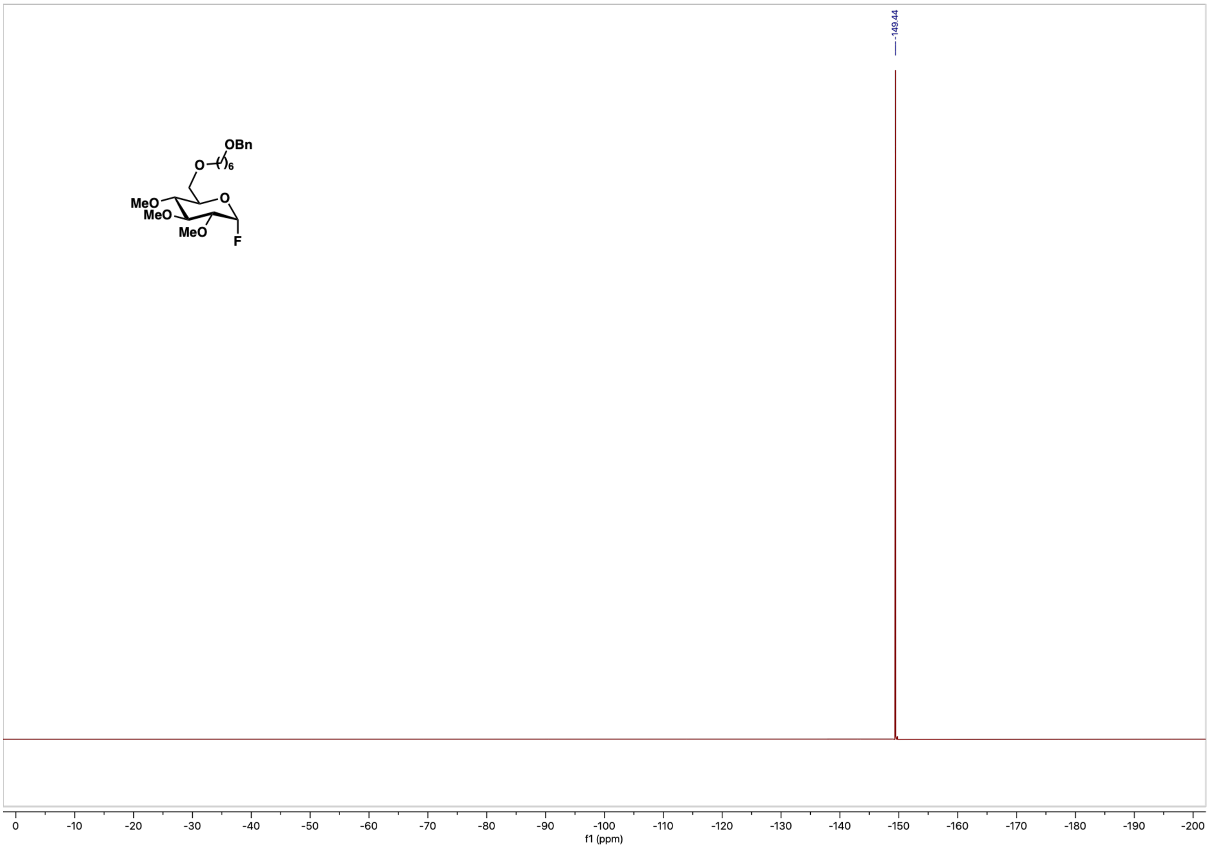

^{13}C NMR (126 MHz, $CDCl_3$, 298K) of compound **S5c**

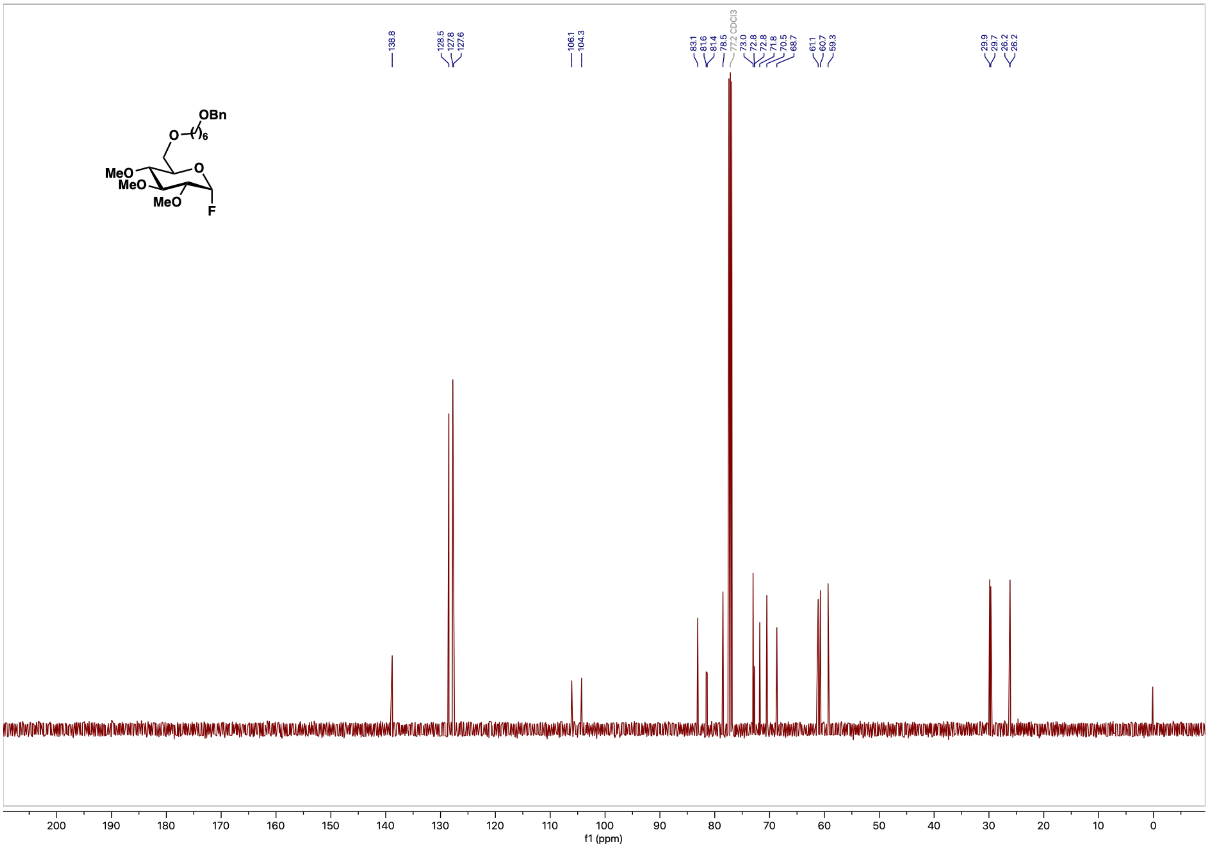

^{1}H NMR (500 MHz, $CDCl_3$, 298K) of compound **1c**

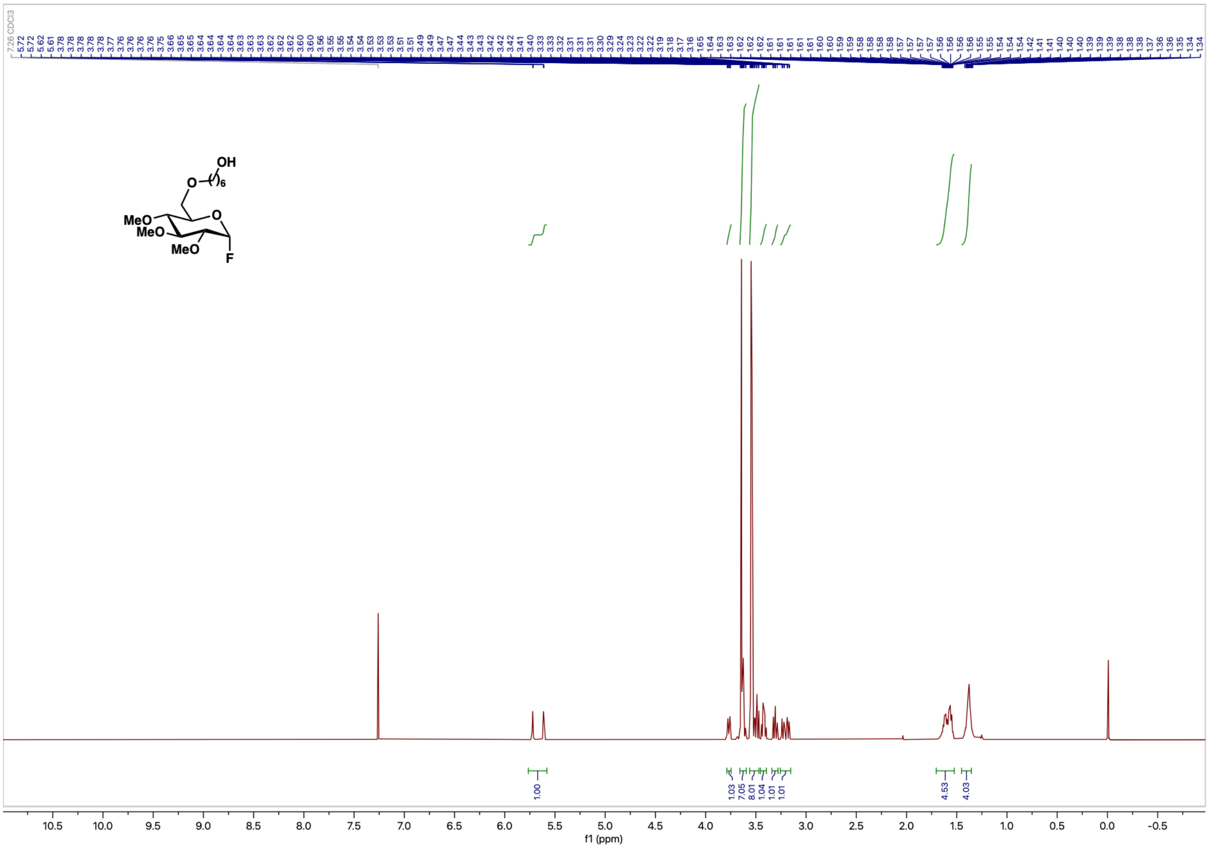

^{19}F NMR (470 MHz, $CDCl_3$, 298K) of compound **1c**

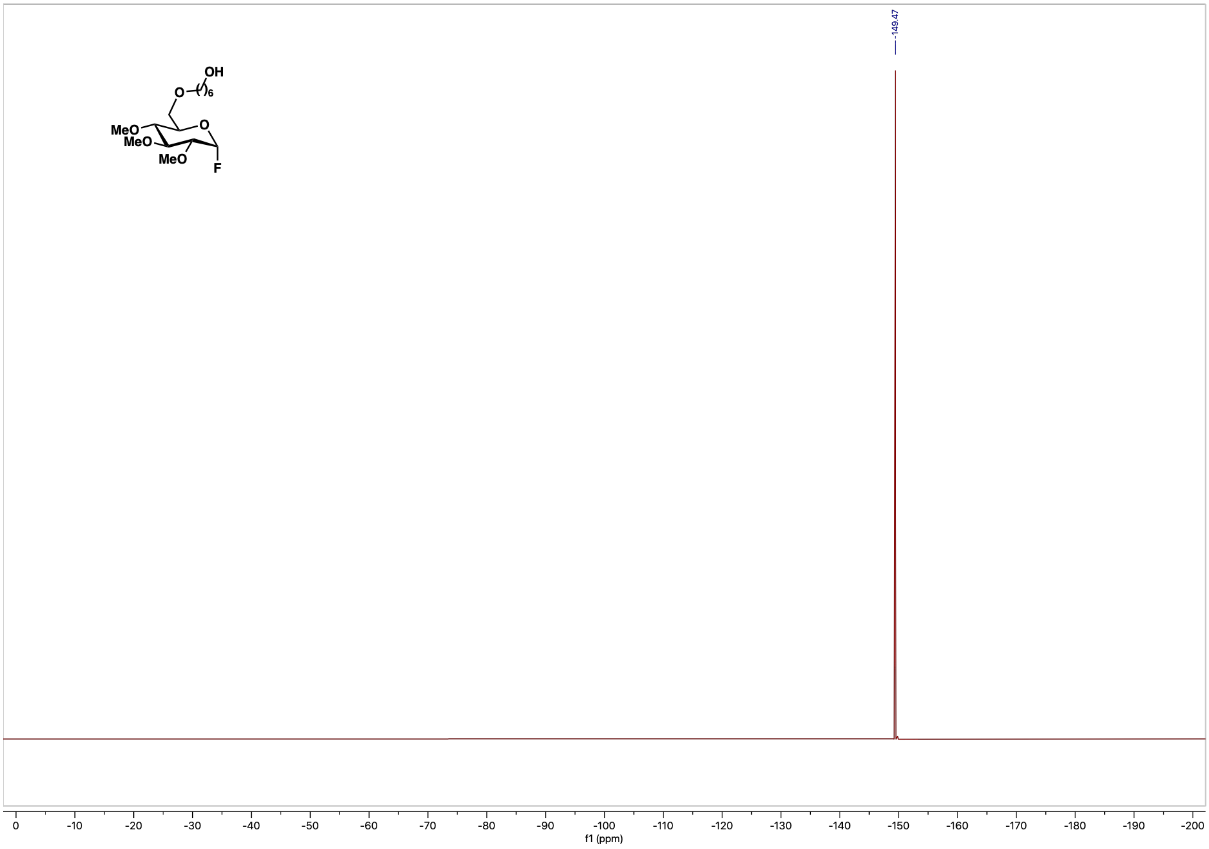

^{13}C NMR (126 MHz, $CDCl_3$, 298K) of compound **1c**

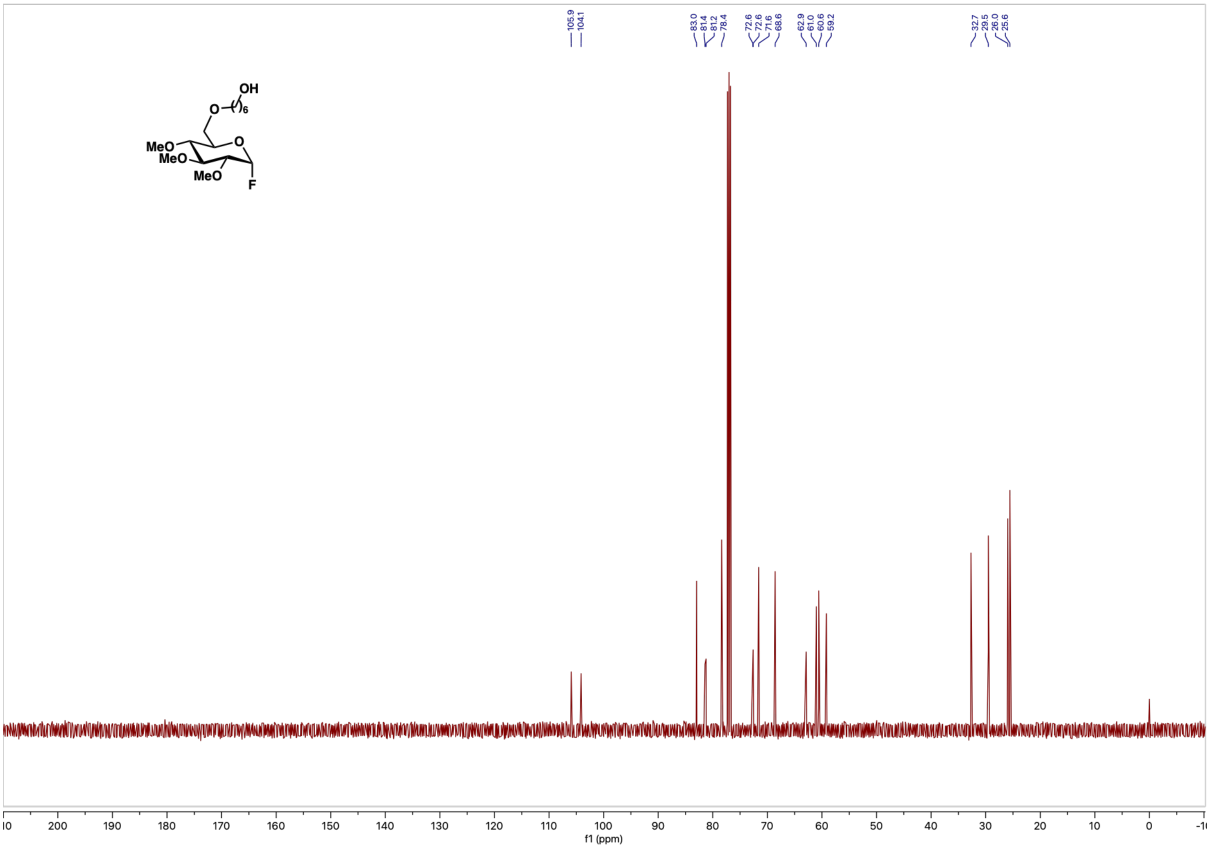

^{1}H NMR (500 MHz, $CDCl_3$, 298K) of compound **S5d**

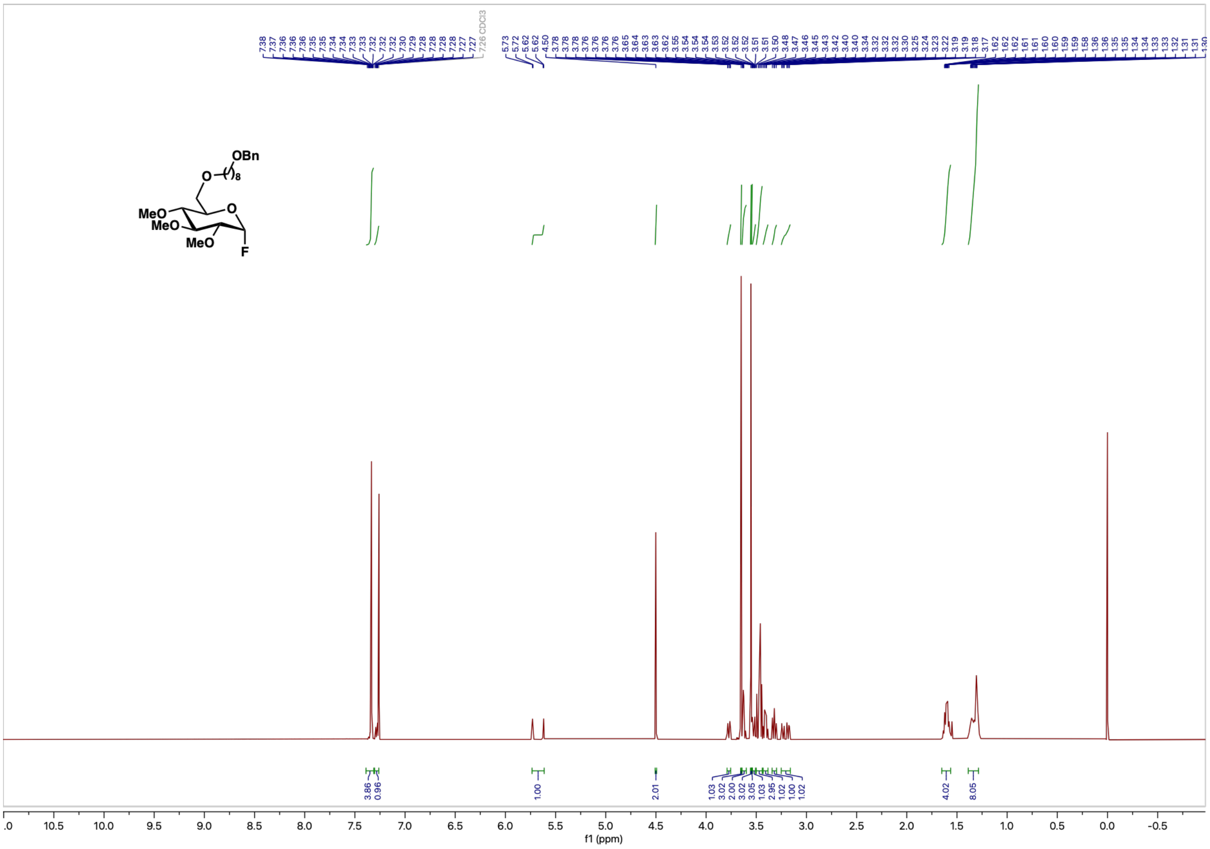

^{19}F NMR (470 MHz, $CDCl_3$, 298K) of compound **S5d**

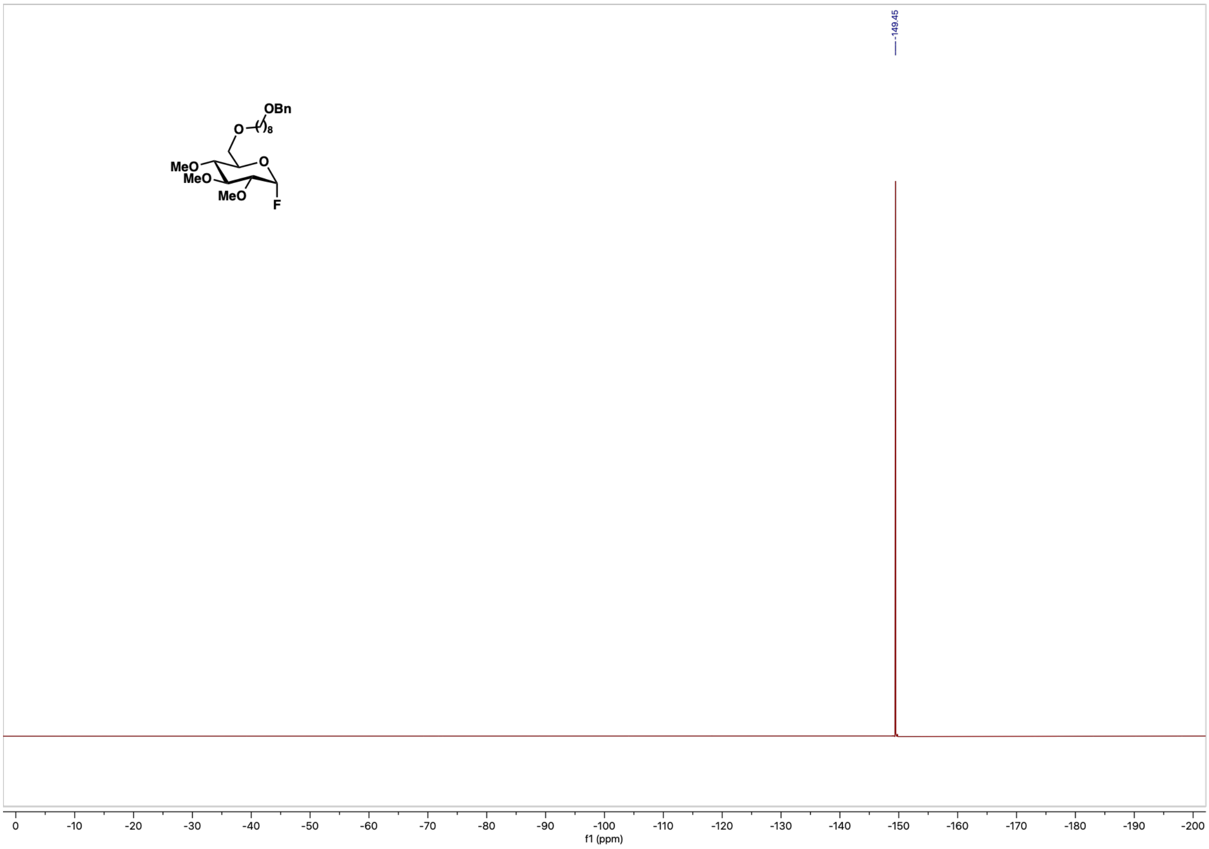

^{13}C NMR (126 MHz, $CDCl_3$, 298K) of compound **S5d**

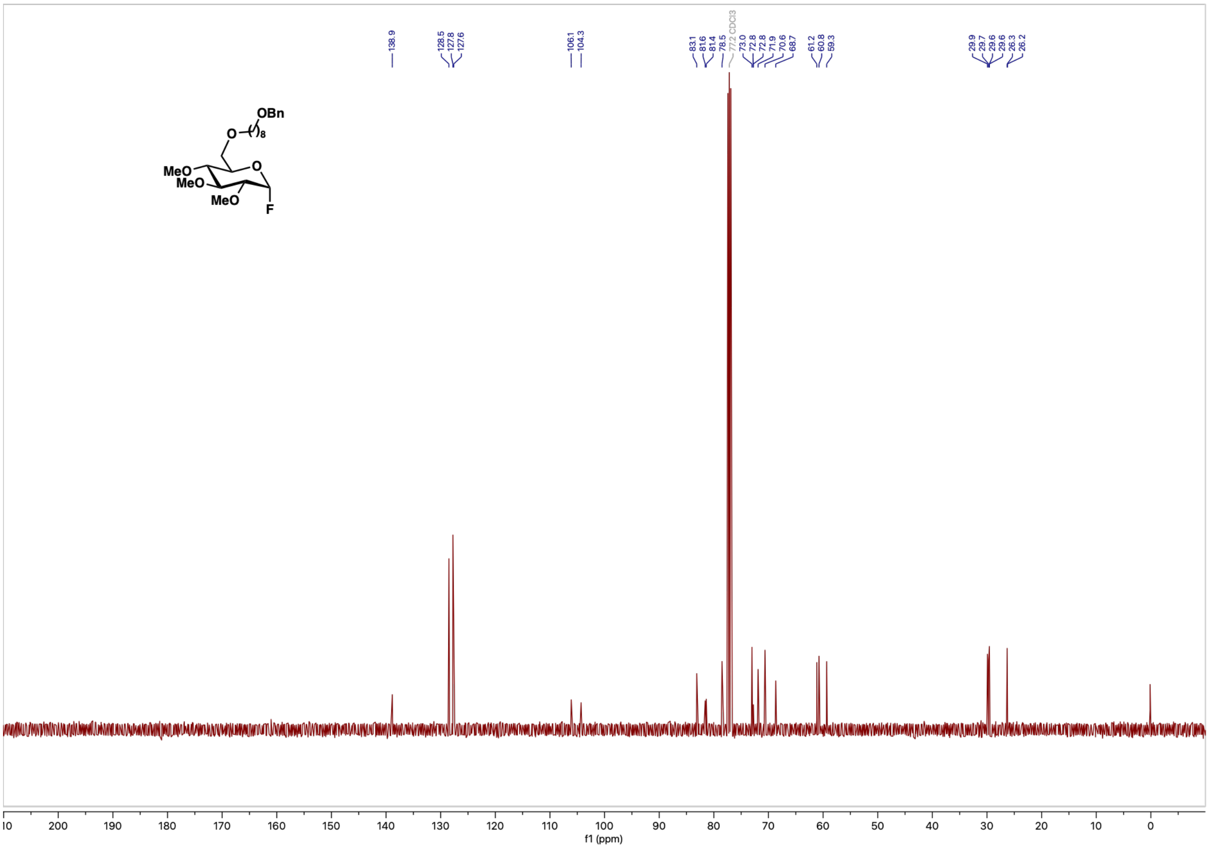

^{1}H NMR (500 MHz, $CDCl_3$, 298K) of compound **1d**

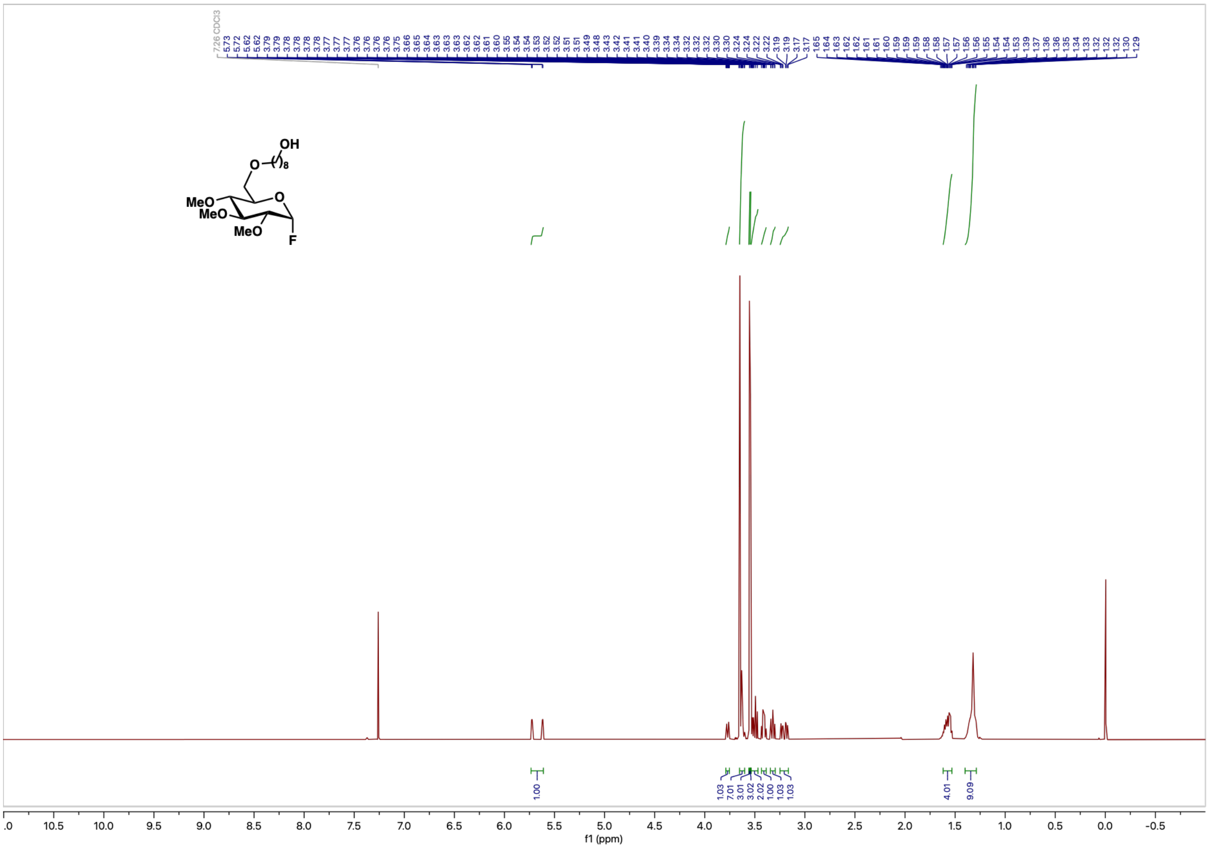

^{19}F NMR (470 MHz, $CDCl_3$, 298K) of compound **1d**

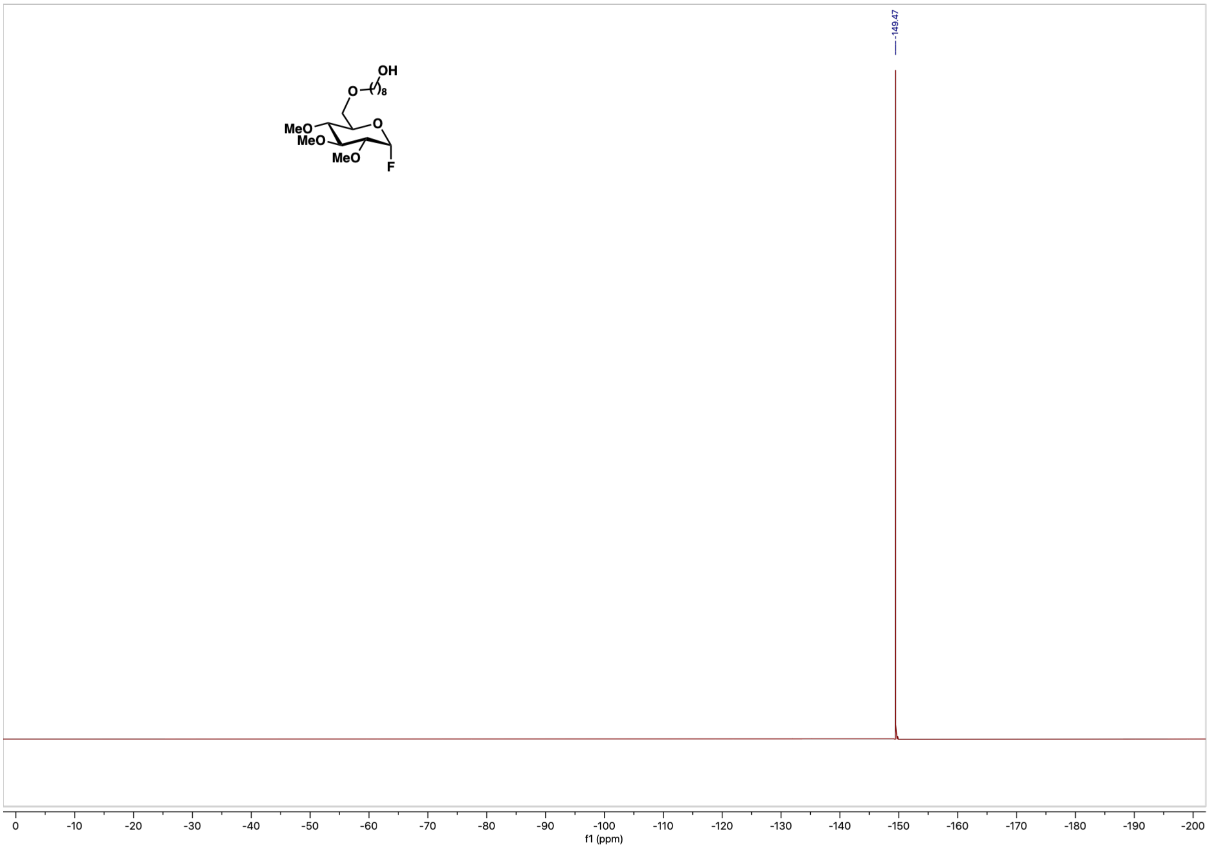

^{13}C NMR (126 MHz, $CDCl_3$, 298K) of compound **1d**

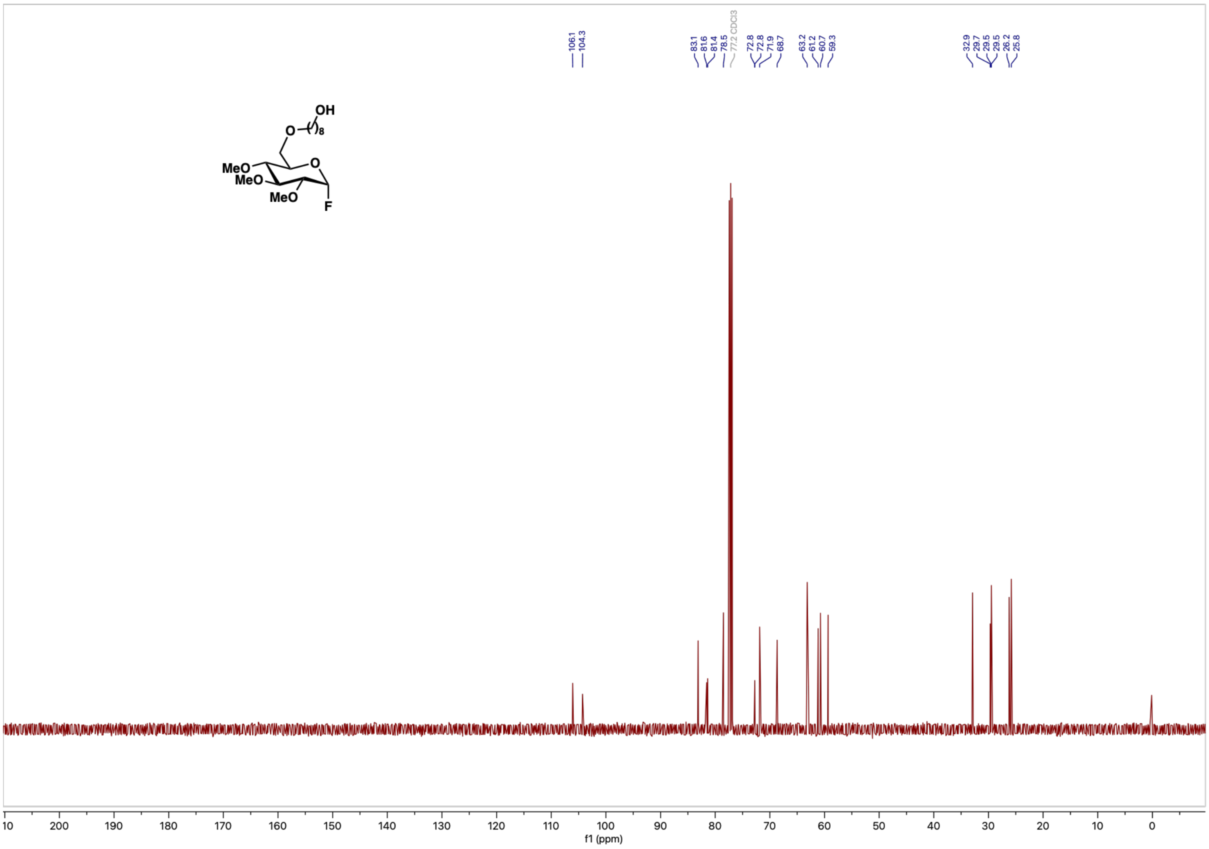

^{1}H NMR (500 MHz, CDCl$_3$, 298K) of compound **S5e**

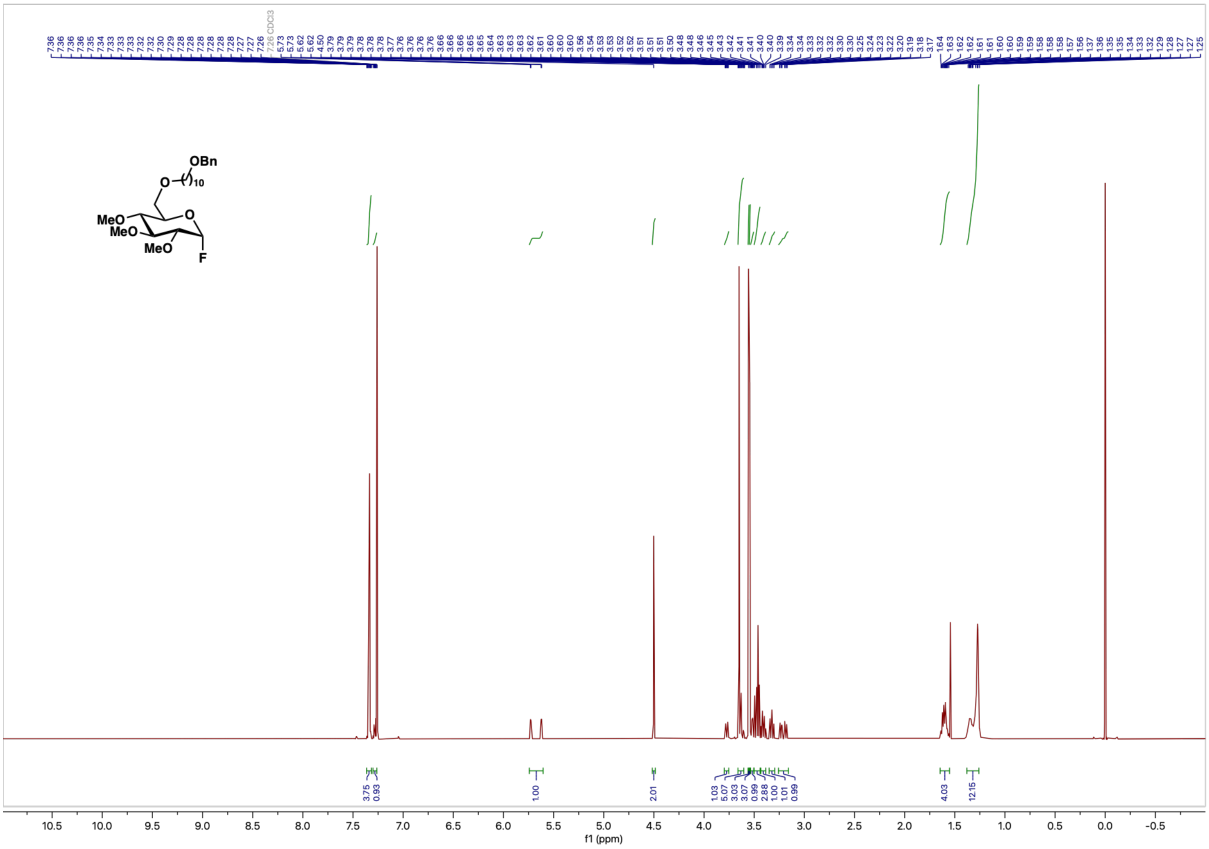

^{19}F NMR (470 MHz, CDCl$_3$, 298K) of compound **S5e**

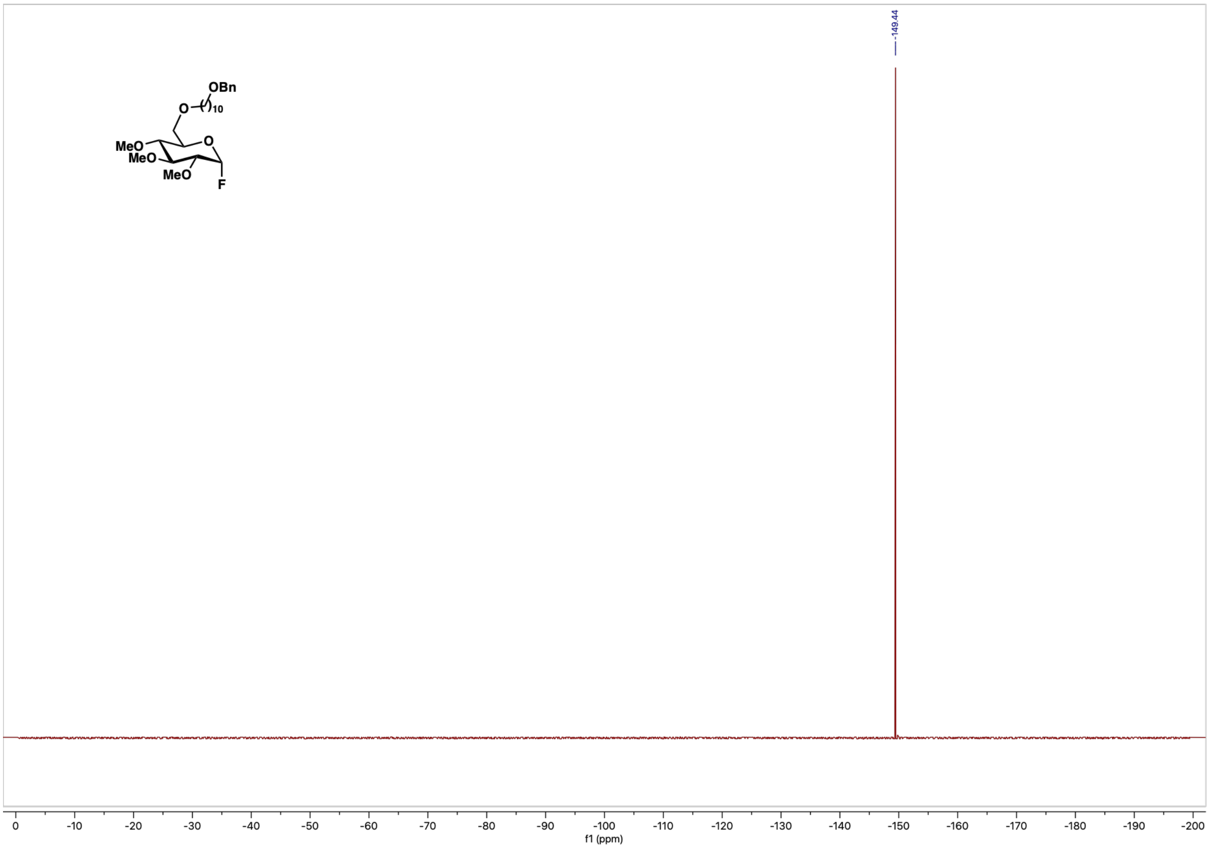

^{13}C NMR (126 MHz, $CDCl_3$, 298K) of compound **S5e**

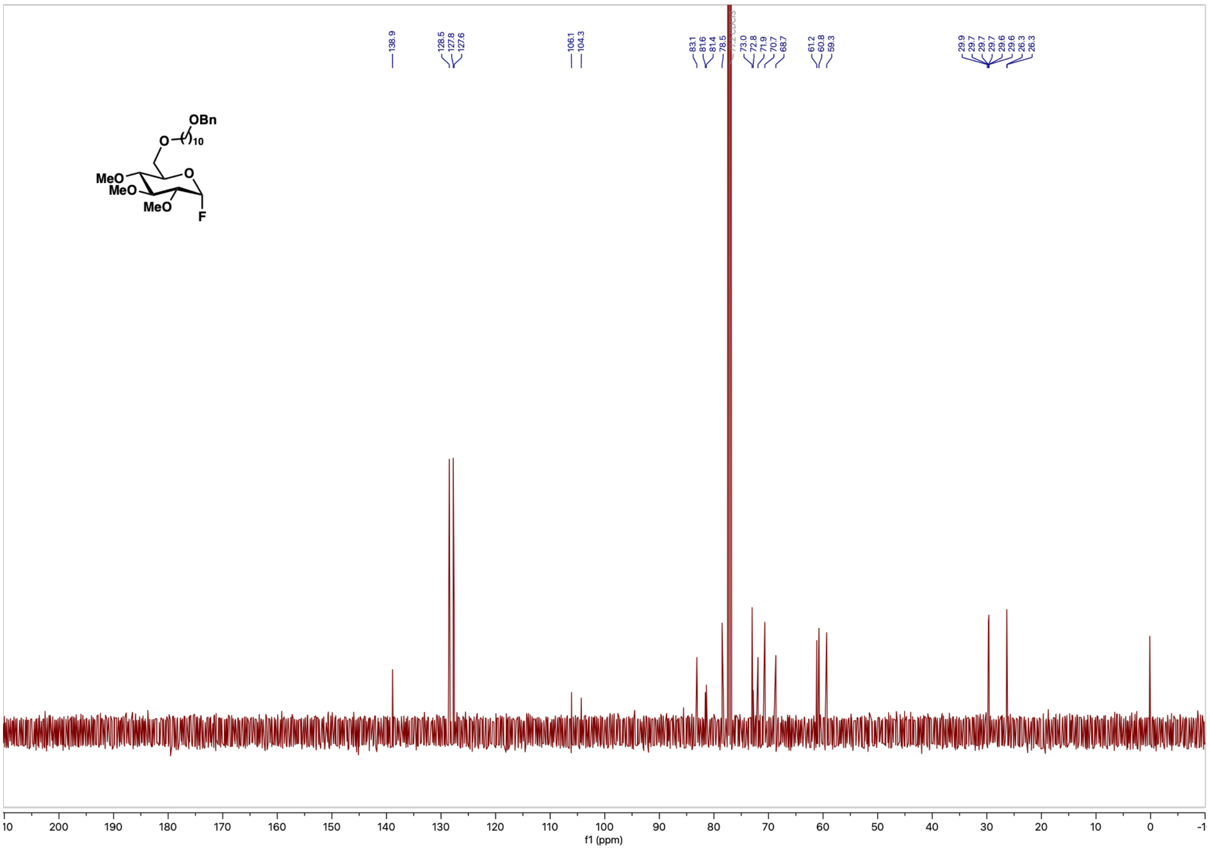

^{1}H NMR (500 MHz, $CDCl_3$, 298K) of compound **1e**

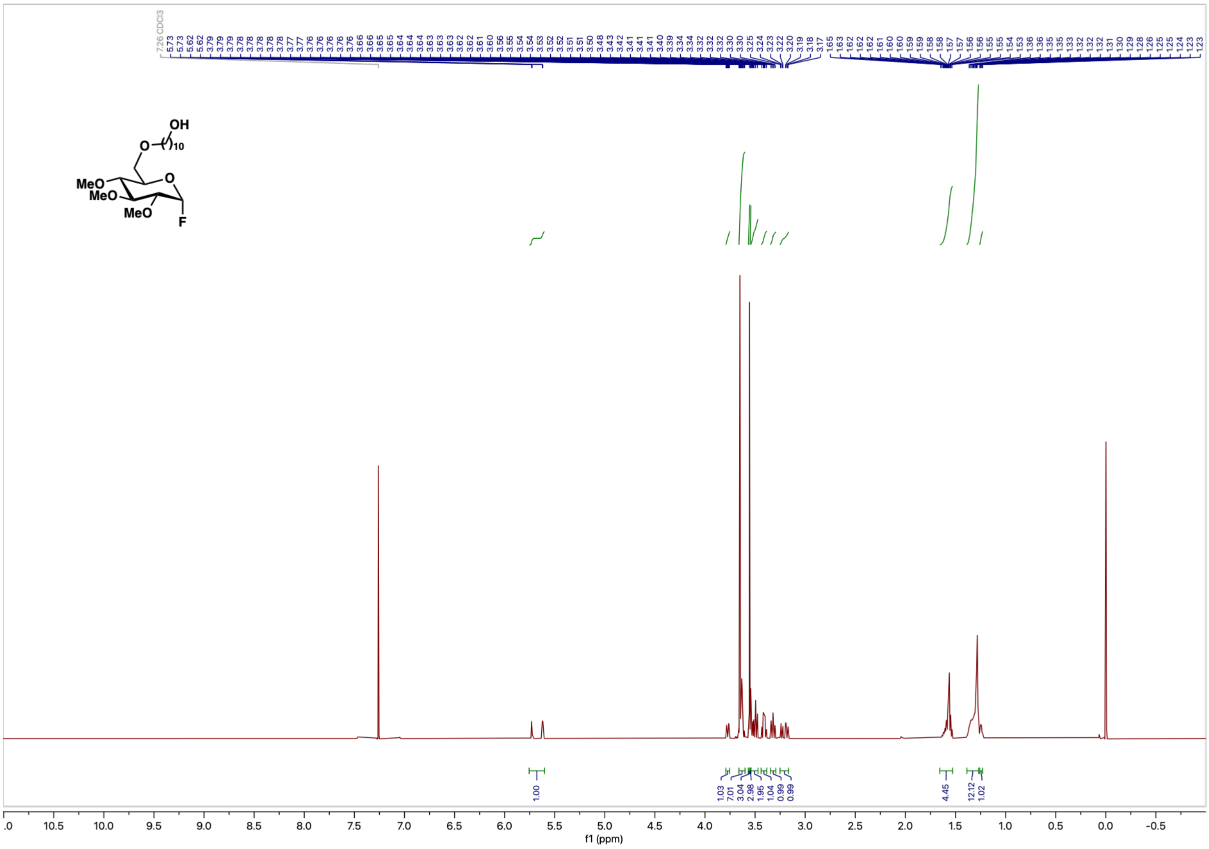

^{19}F NMR (470 MHz, $CDCl_3$, 298K) of compound **1e**

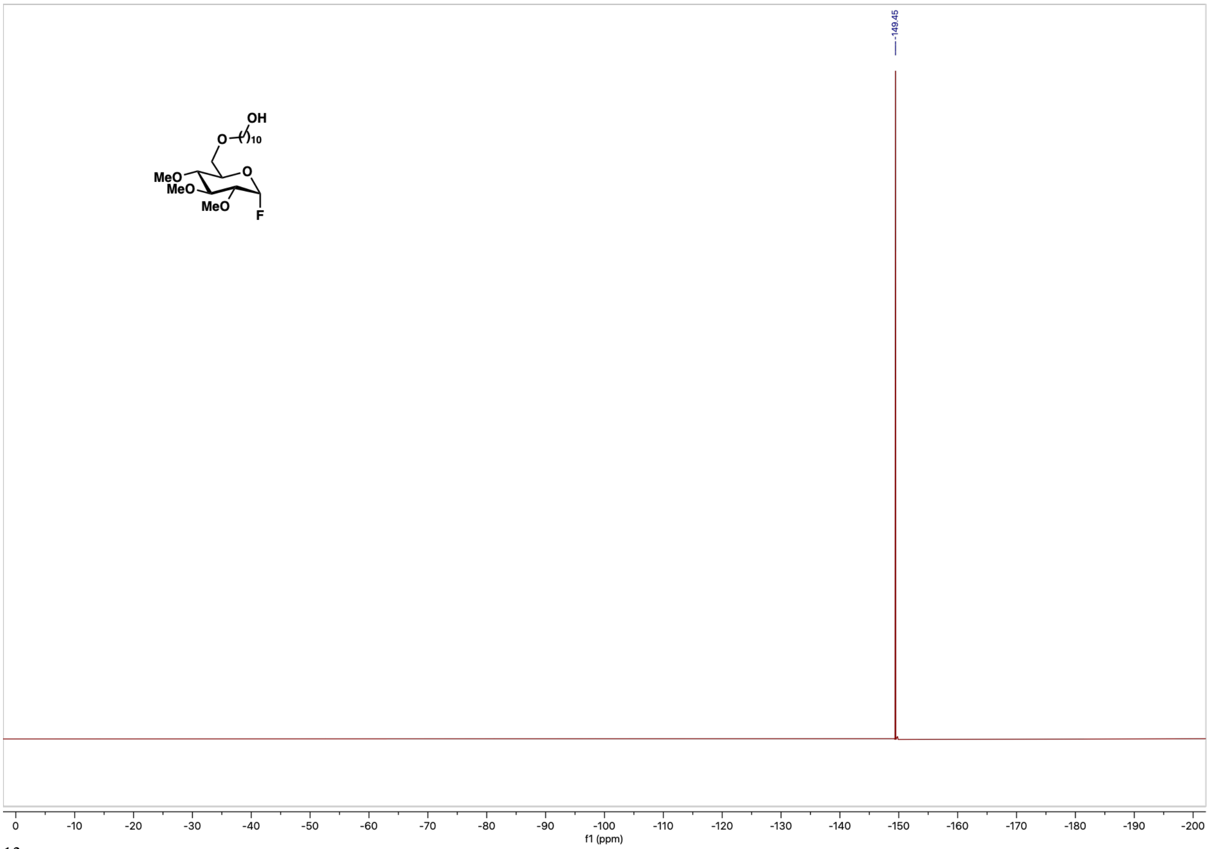

^{13}C NMR (126 MHz, $CDCl_3$, 298K) of compound **1e**

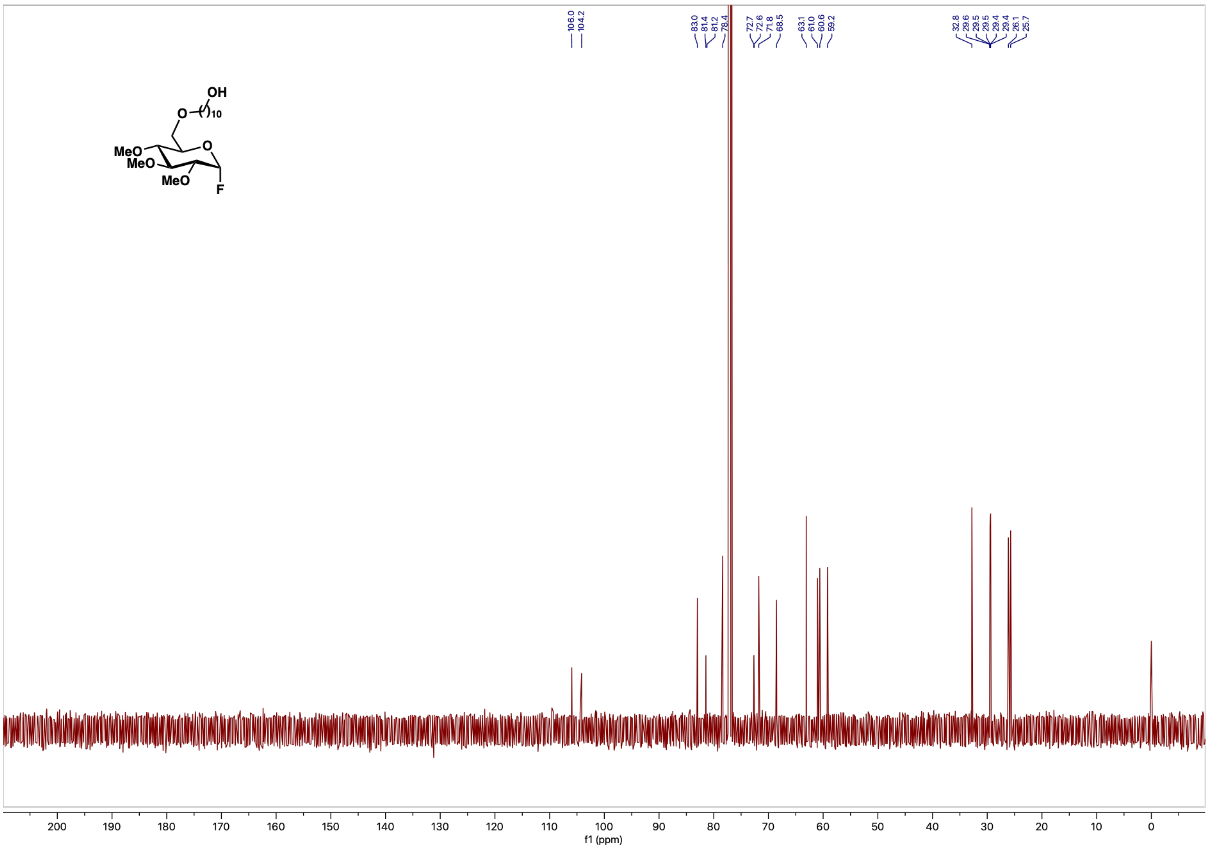

^{1}H NMR (500 MHz, $CDCl_3$, 298K) of compound **S5f**

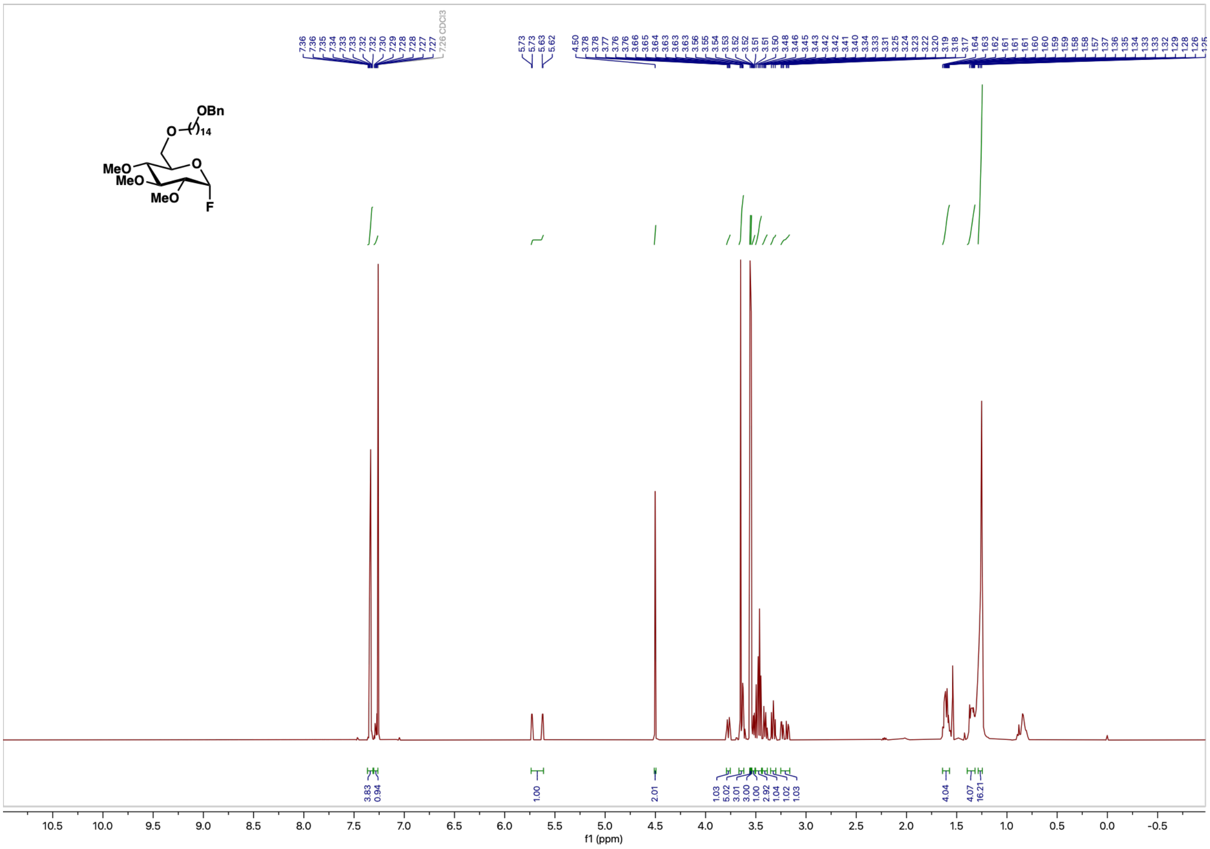

^{19}F NMR (470 MHz, $CDCl_3$, 298K) of compound **S5f**

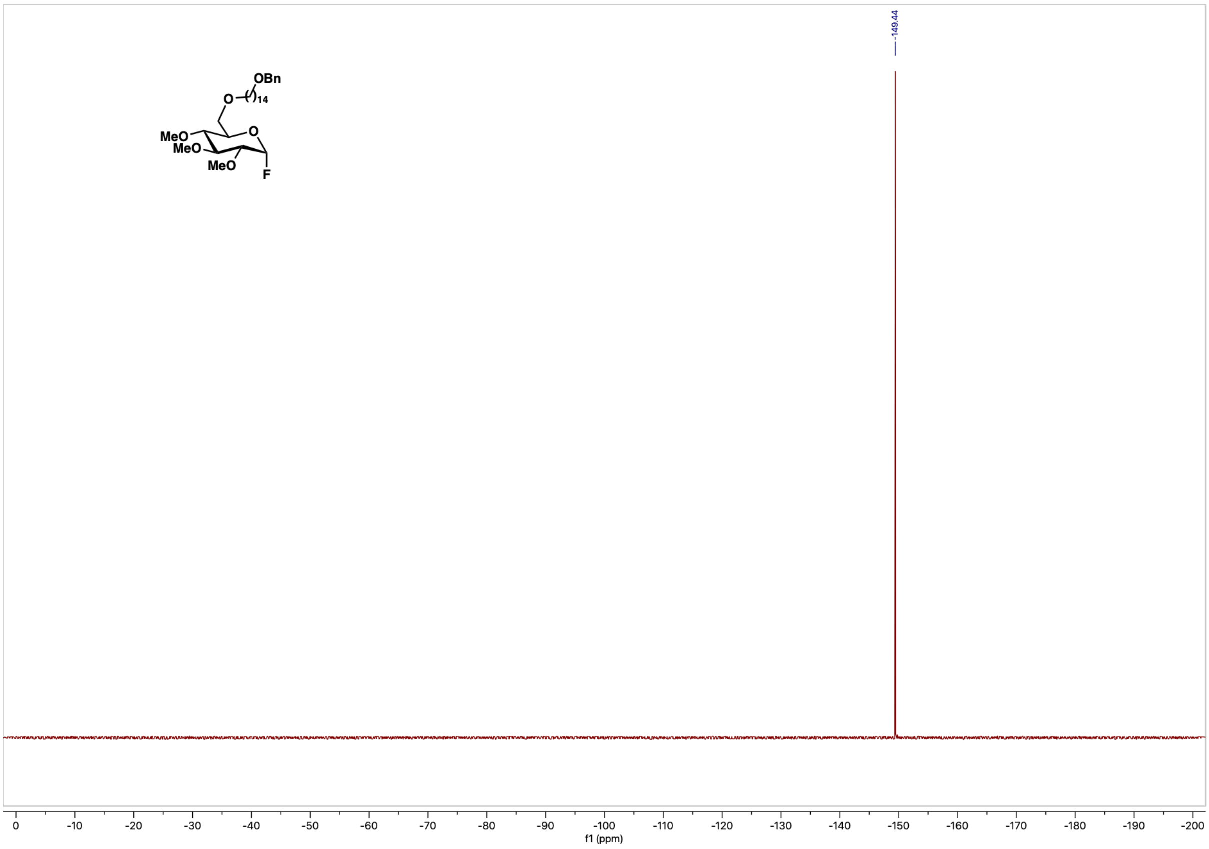

^{13}C NMR (126 MHz, $CDCl_3$, 298K) of compound **S5f**

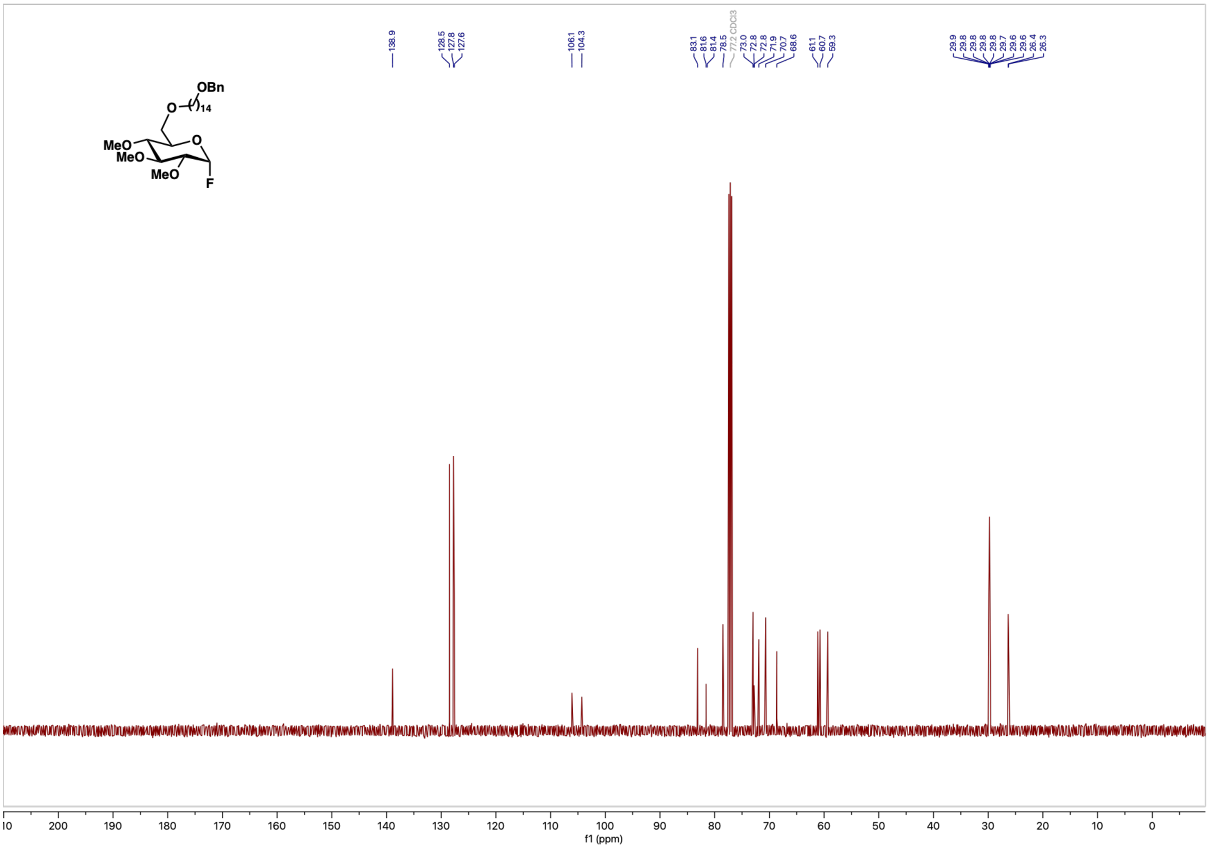

^{1}H NMR (500 MHz, $CDCl_3$, 298K) of compound **1f**

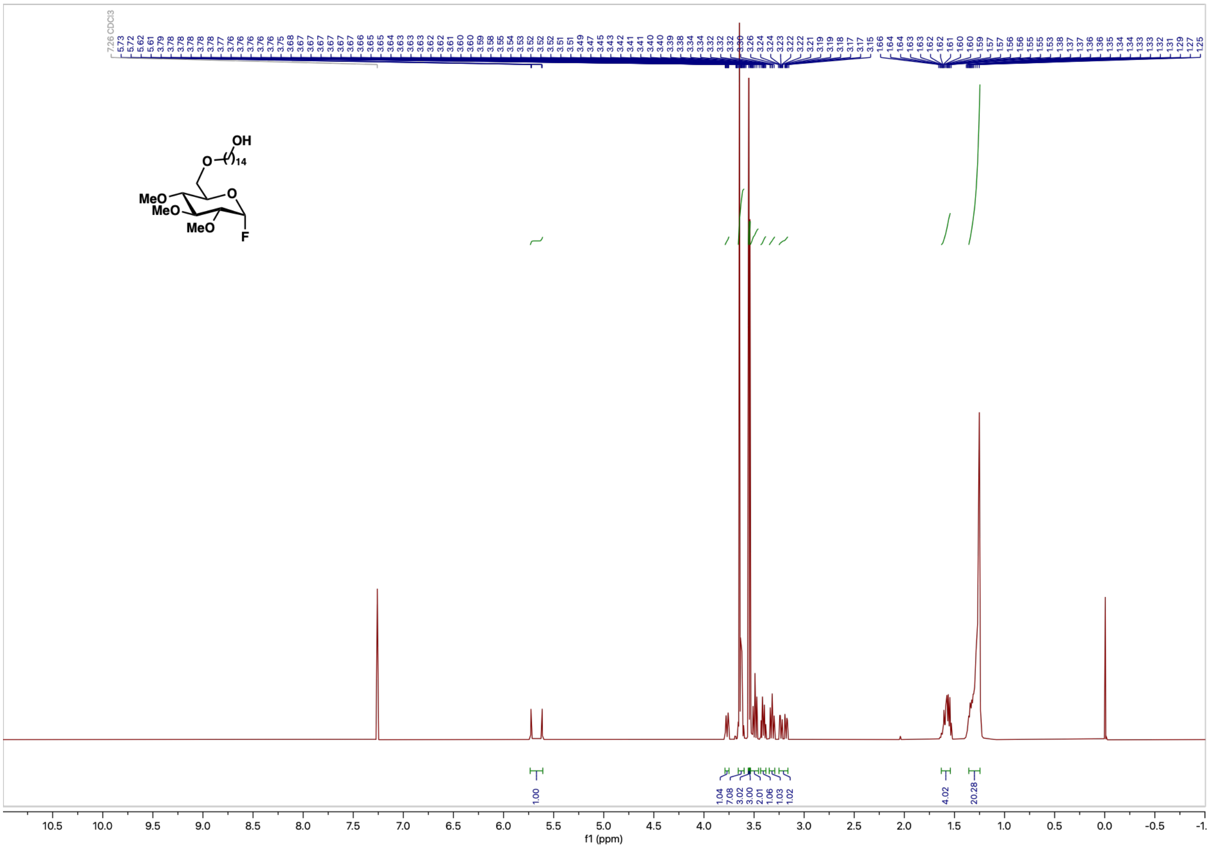

^{19}F NMR (470 MHz, $CDCl_3$, 298K) of compound **1f**

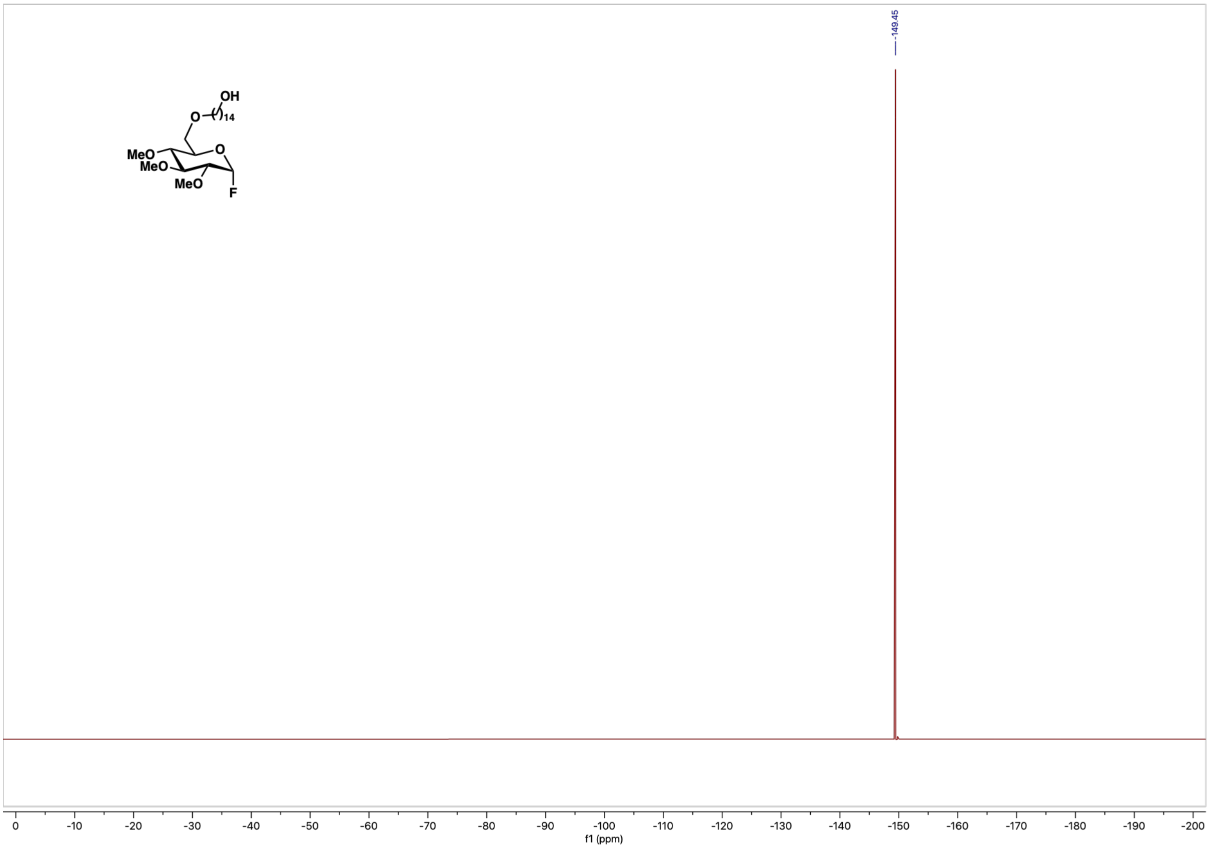

^{13}C NMR (126 MHz, $CDCl_3$, 298K) of compound **1f**

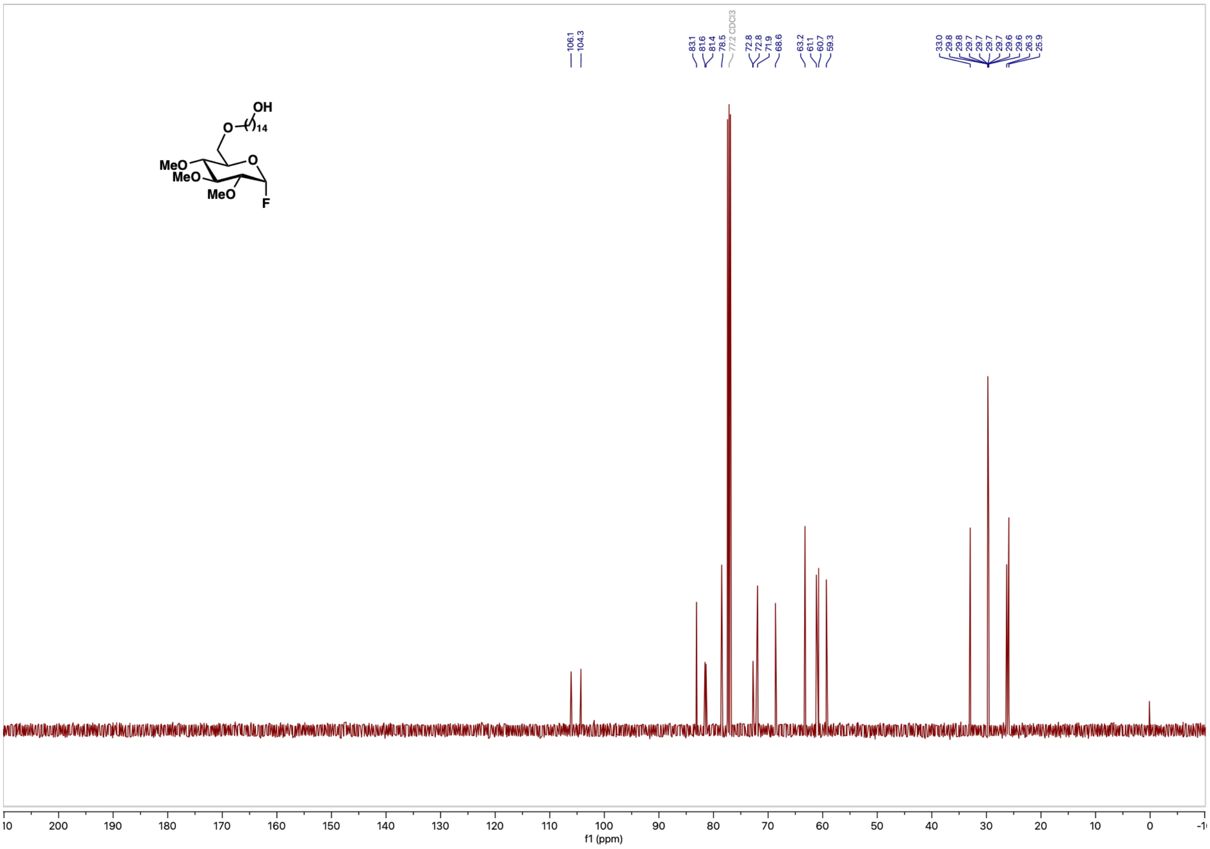

^{1}H NMR (500 MHz, $CDCl_3$, 298K) of compound **R-22b**

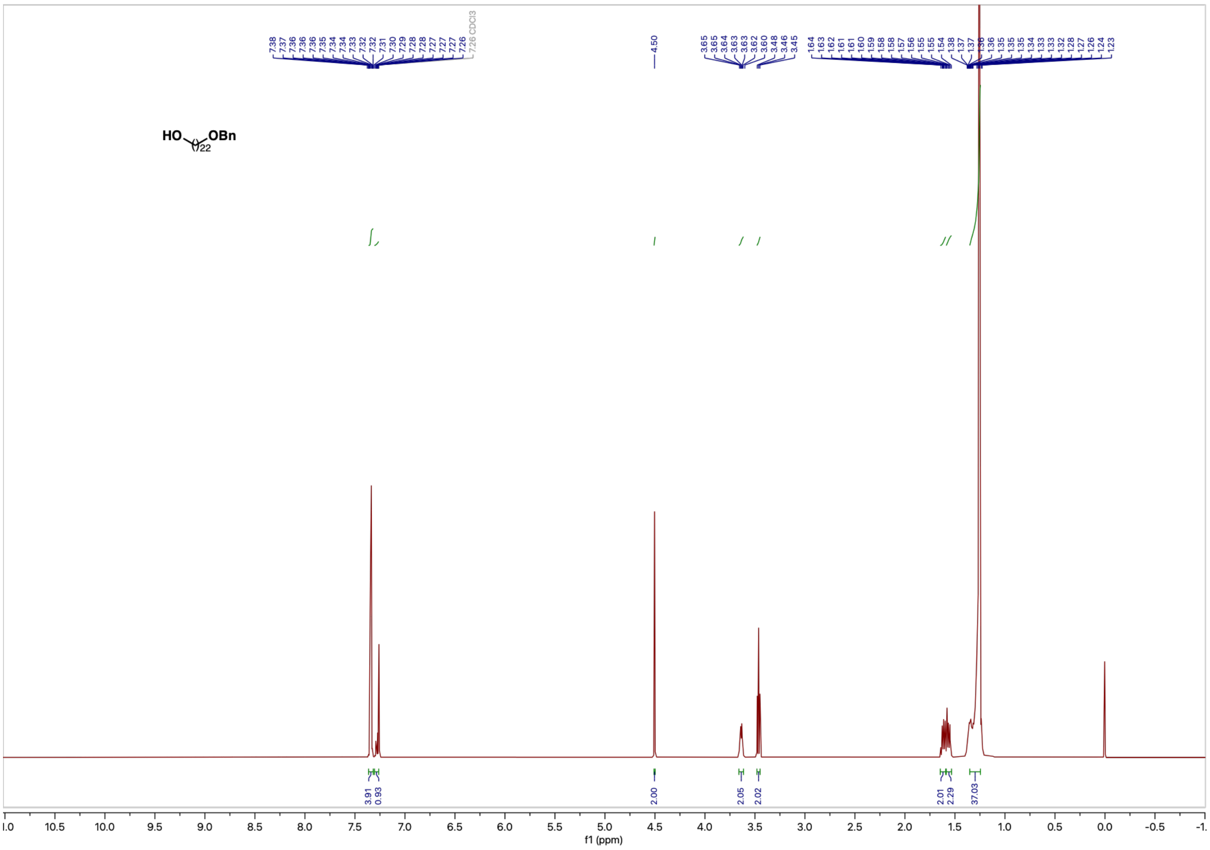

^{13}C NMR (126 MHz, $CDCl_3$, 298K) of compound **R-22b**

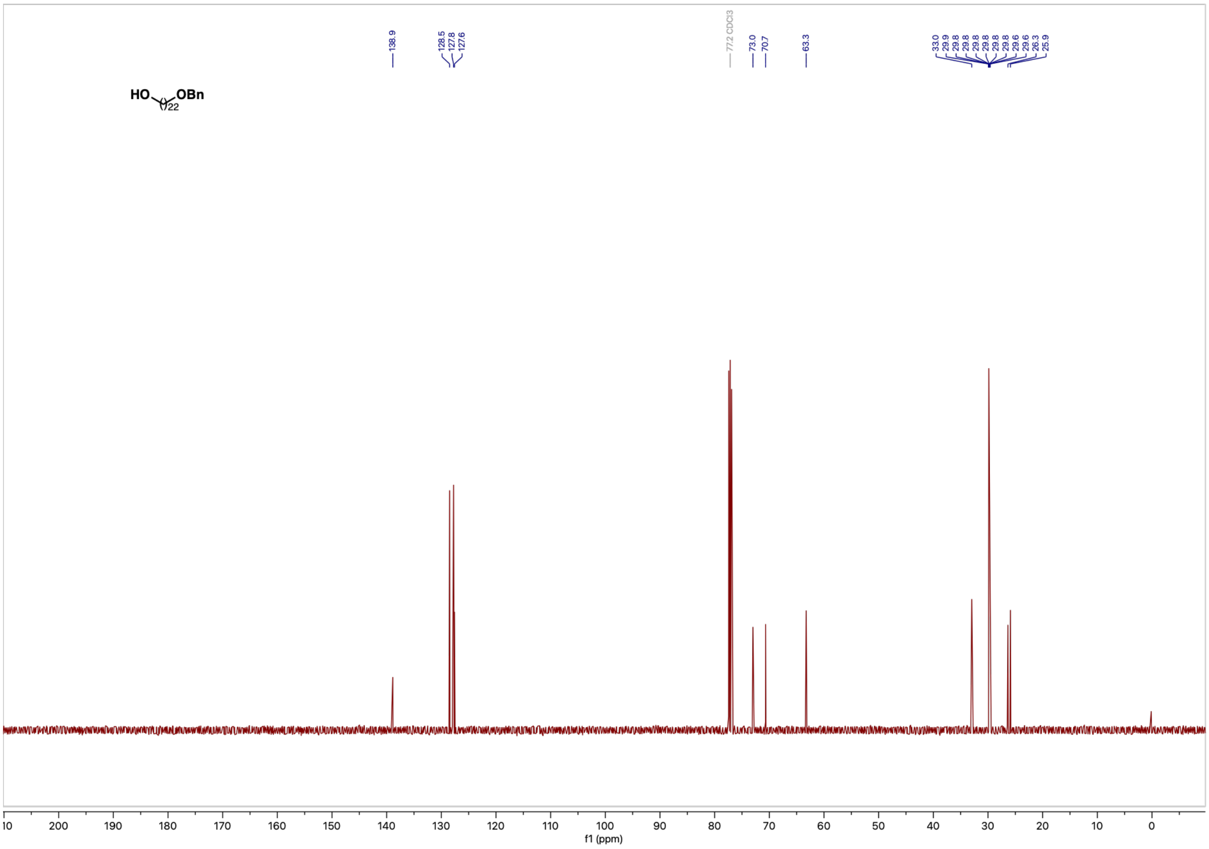

^{1}H NMR (500 MHz, $CDCl_3$, 298K) of compound **R-22c**

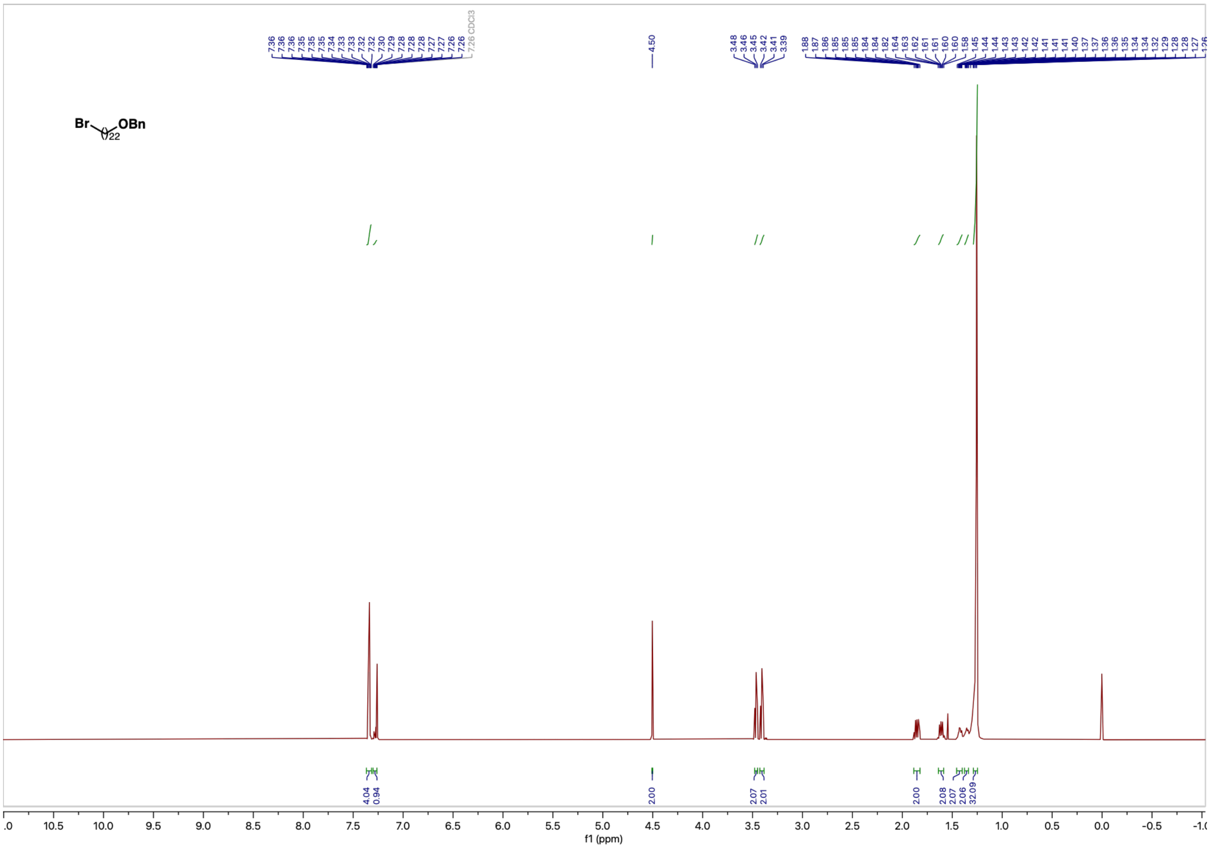

^{13}C NMR (126 MHz, $CDCl_3$, 298K) of compound **R-22c**

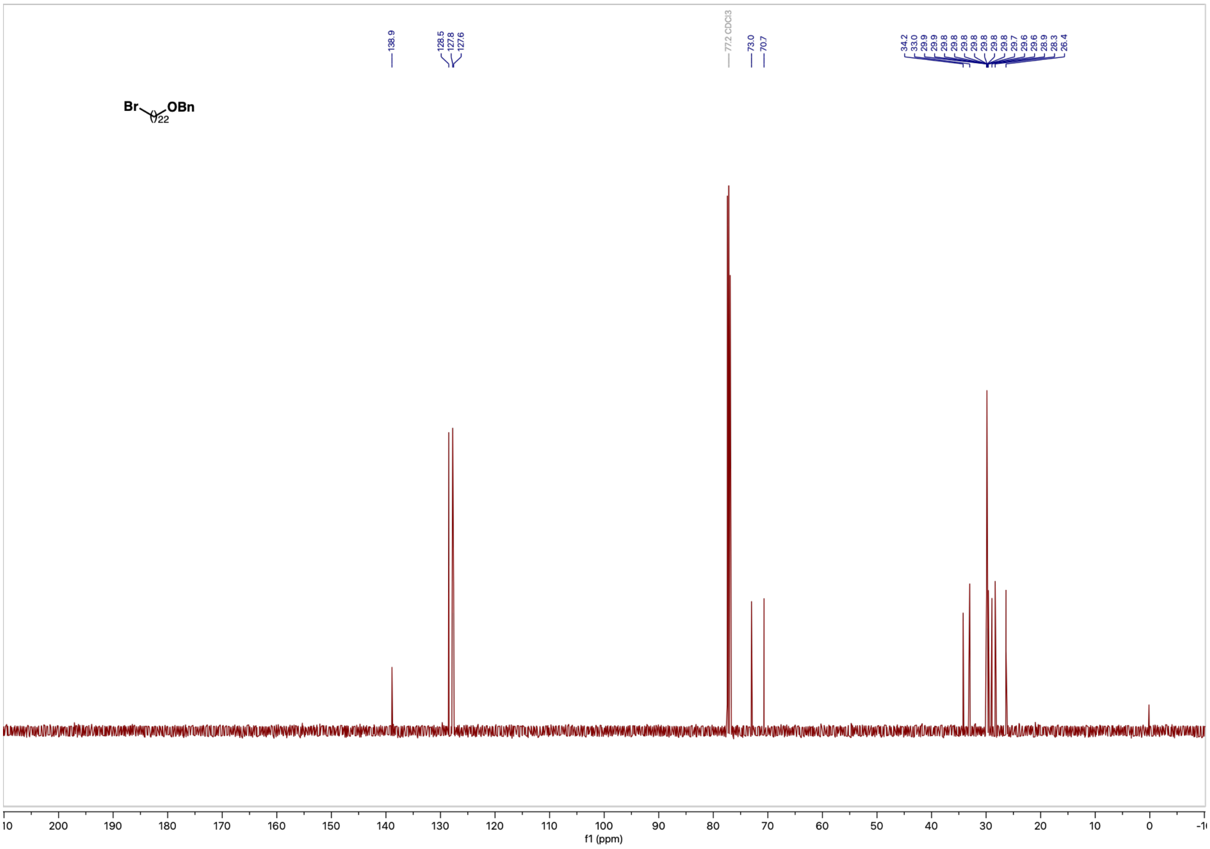

^{1}H NMR (500 MHz, $CDCl_3$, 298K) of compound **S5g**

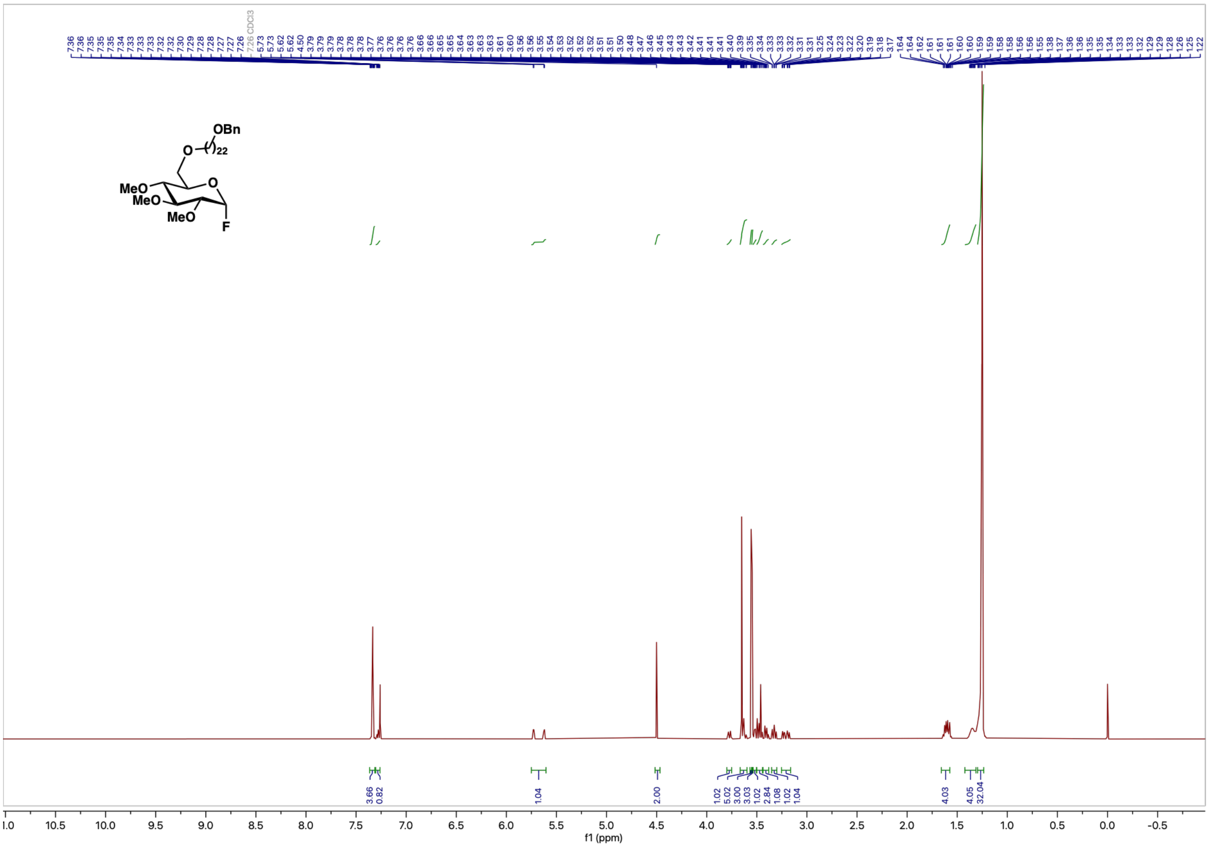

^{19}F NMR (470 MHz, $CDCl_3$, 298K) of compound **S5g**

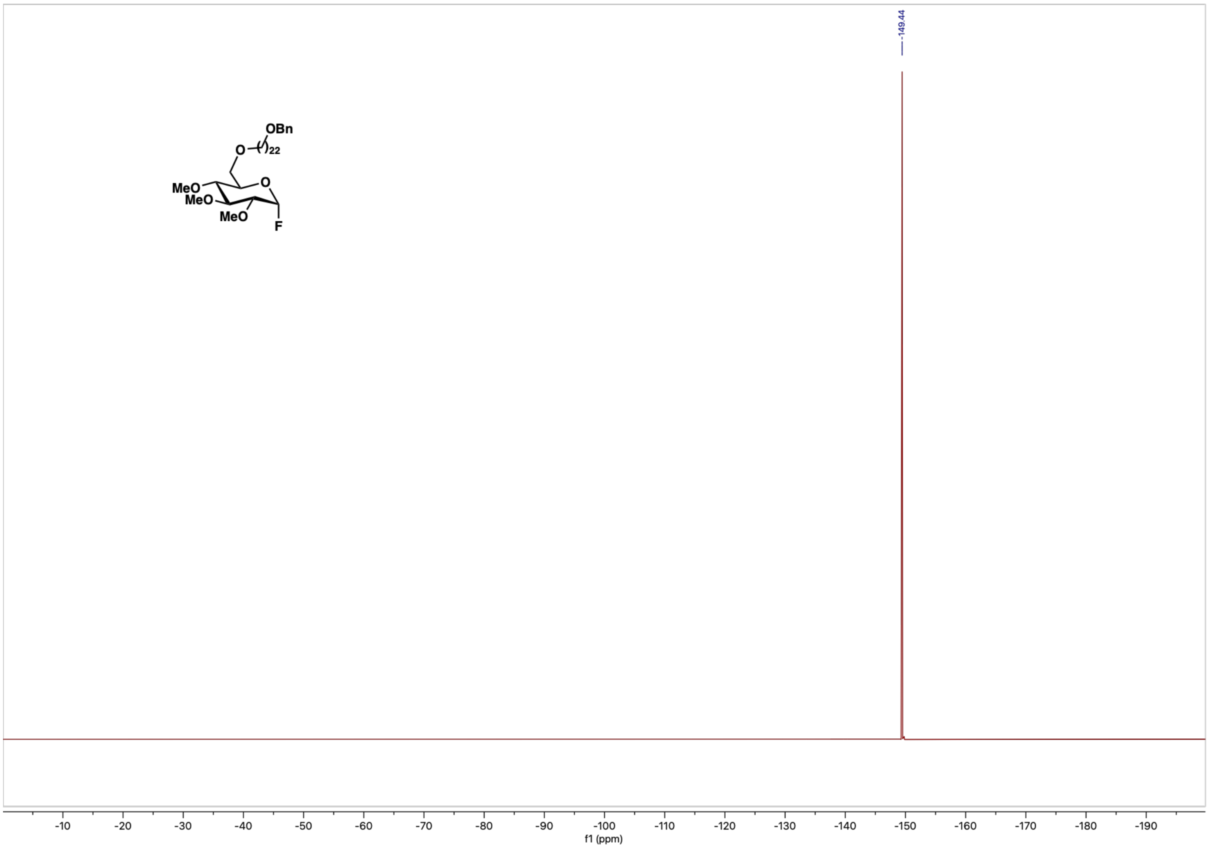

^{13}C NMR (126 MHz, $CDCl_3$, 298K) of compound **S5g**

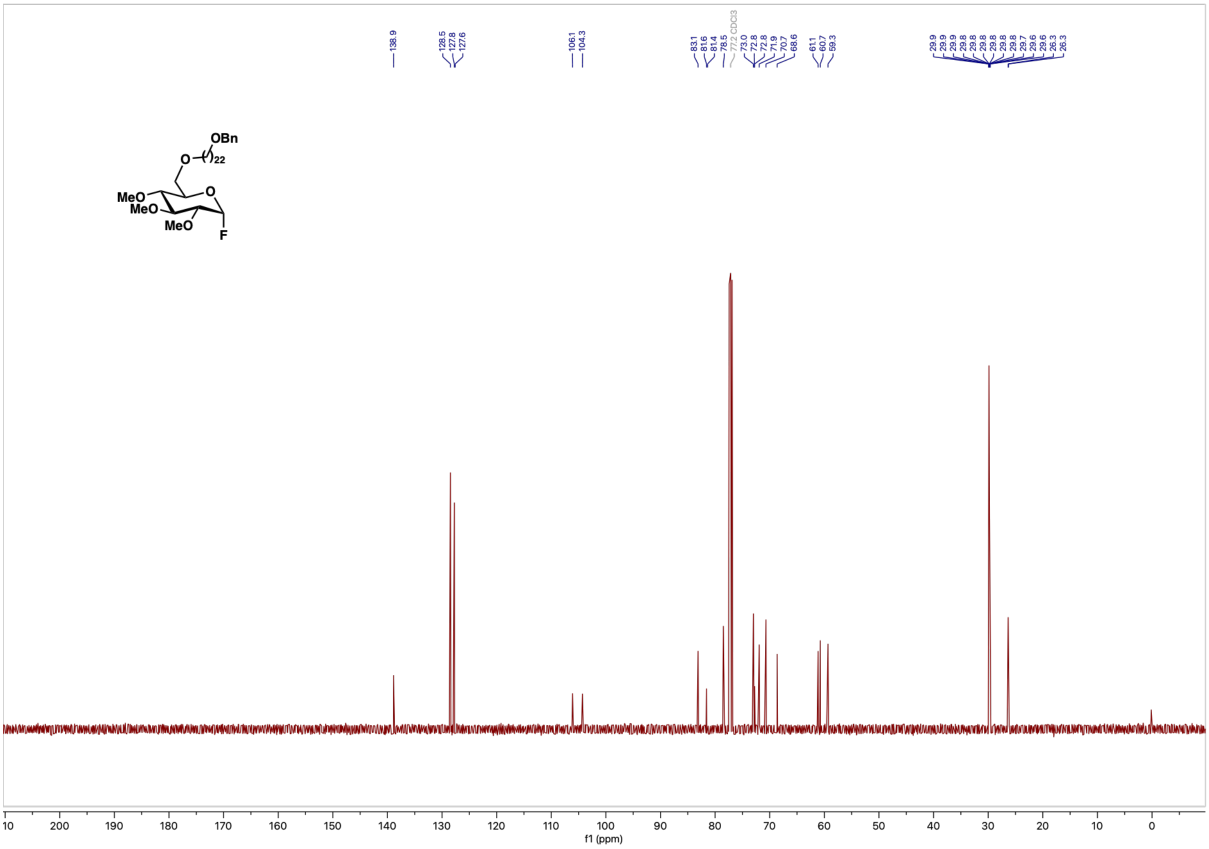

^{1}H NMR (500 MHz, $CDCl_3$, 298K) of compound **1g**

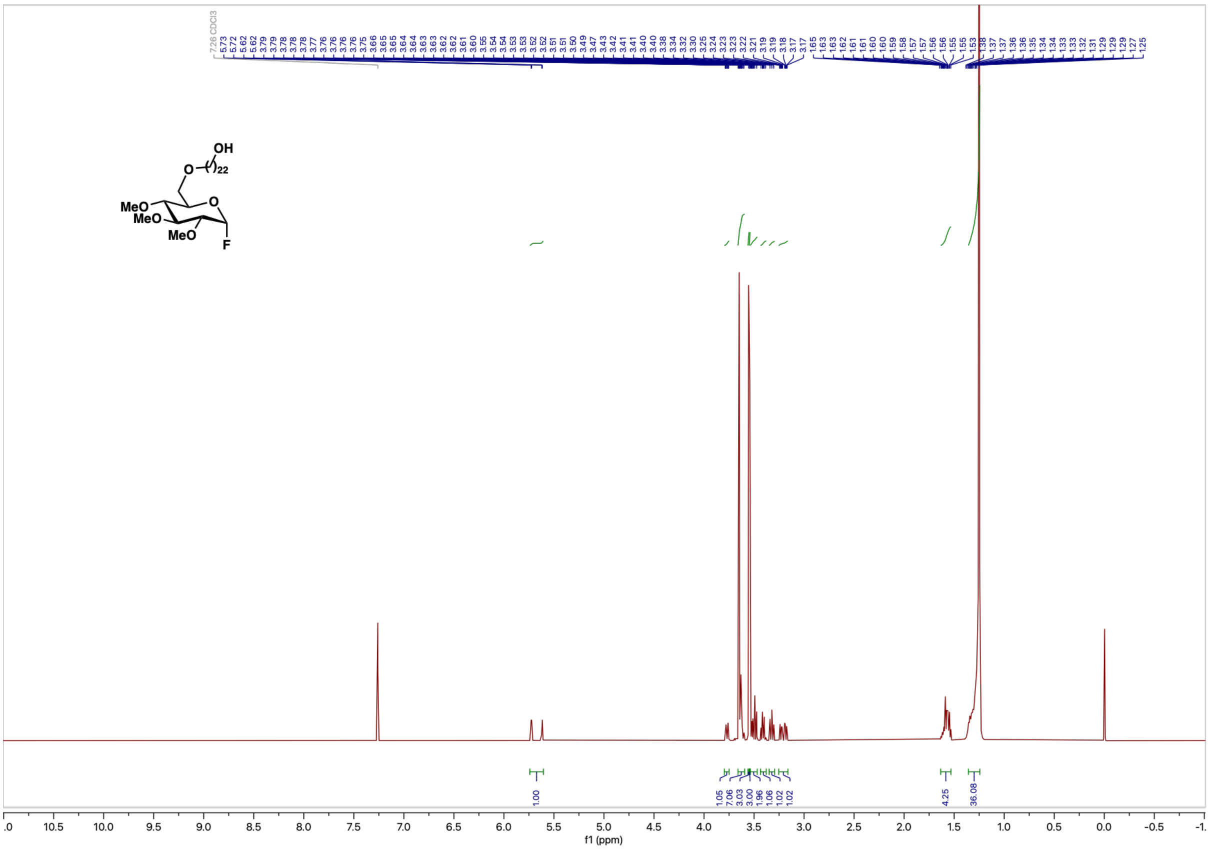

^{19}F NMR (470 MHz, $CDCl_3$, 298K) of compound **1g**

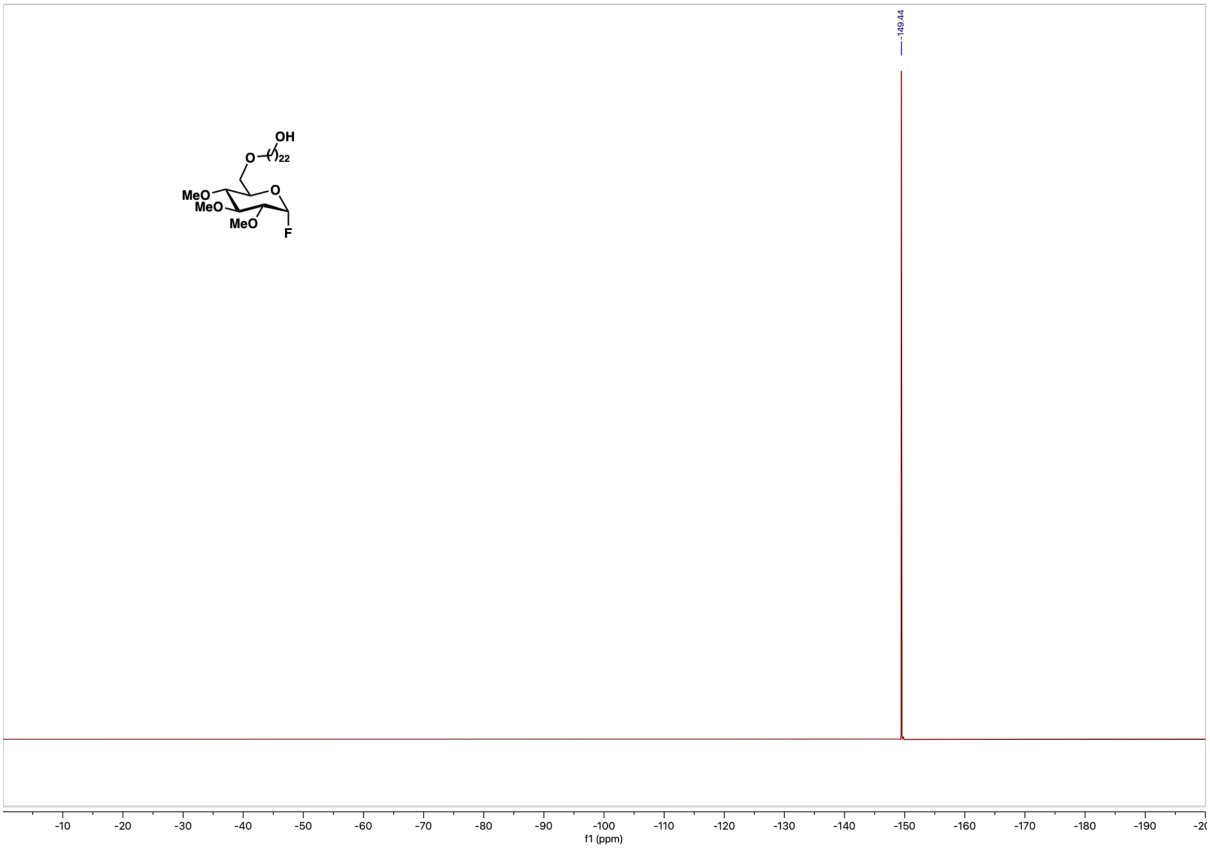

^{13}C NMR (126 MHz, $CDCl_3$, 298K) of compound **1g**

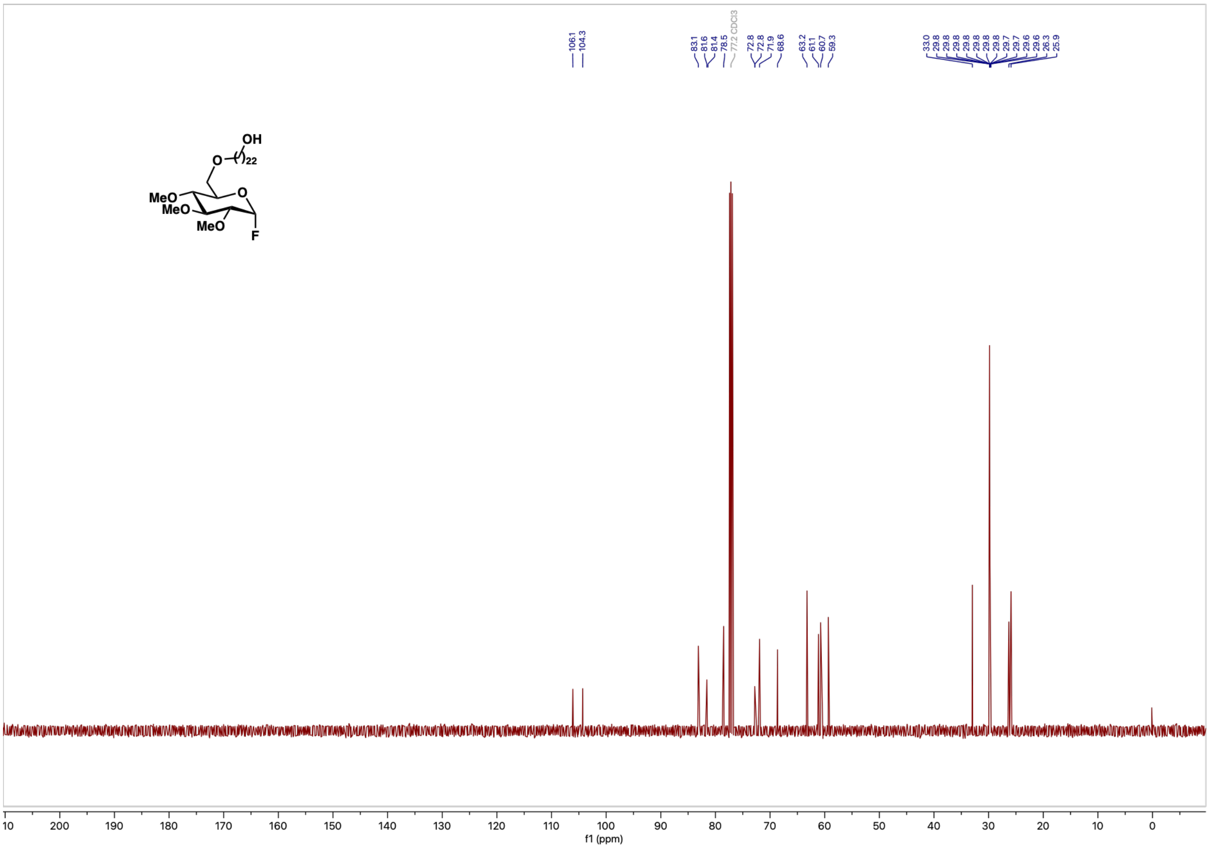

^{1}H NMR (500 MHz, $CDCl_3$, 298K) of compound **S5h**

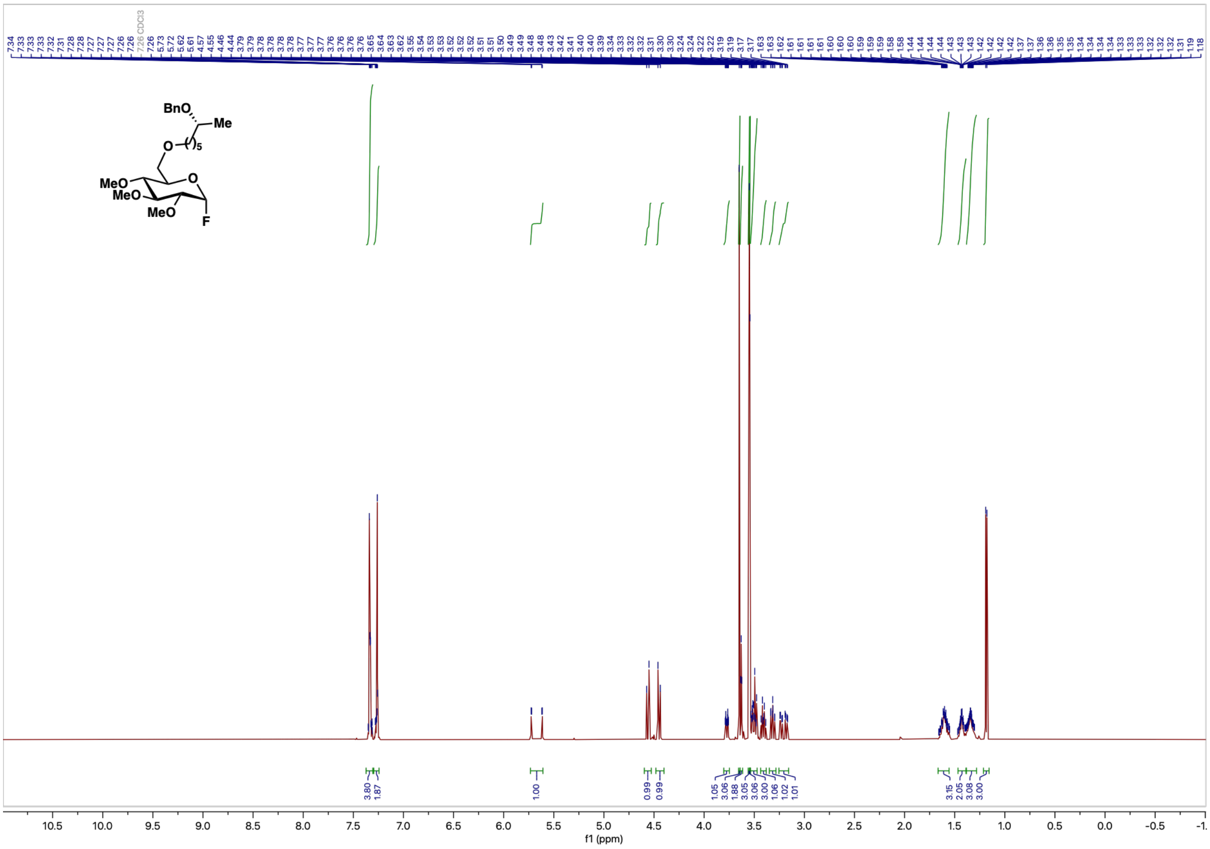

^{19}F NMR (470 MHz, $CDCl_3$, 298K) of compound **S5h**

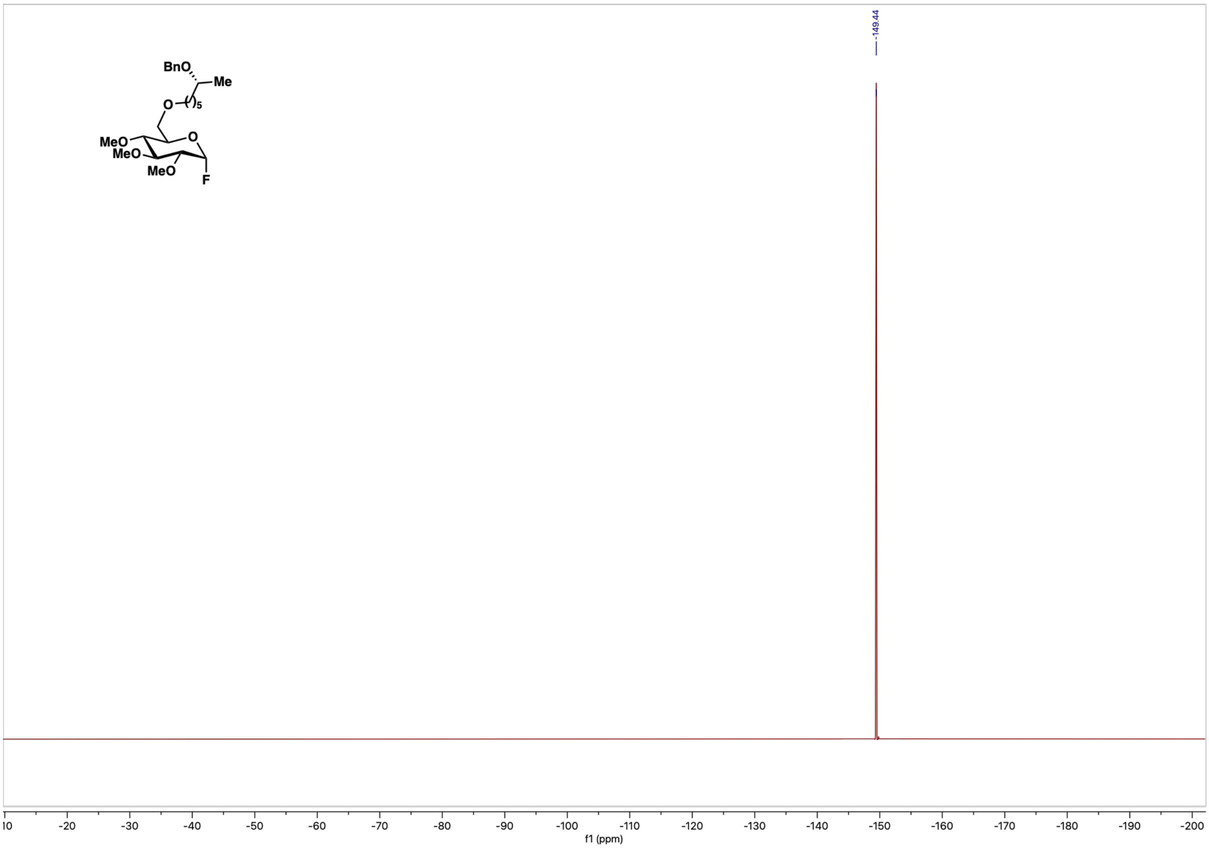

^{13}C NMR (126 MHz, $CDCl_3$, 298K) of compound **S5h**

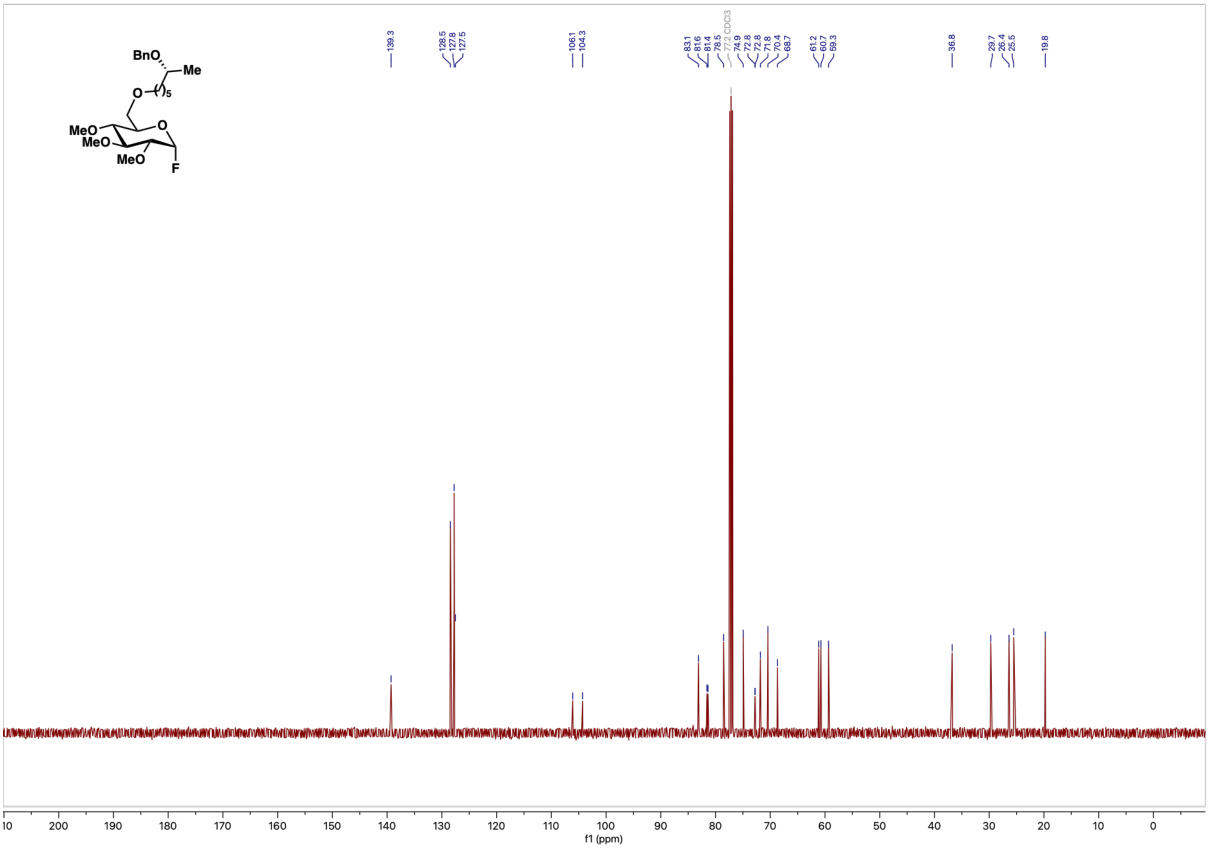

^{1}H NMR (500 MHz, acetone-d_6, 298K) of compound **1h**

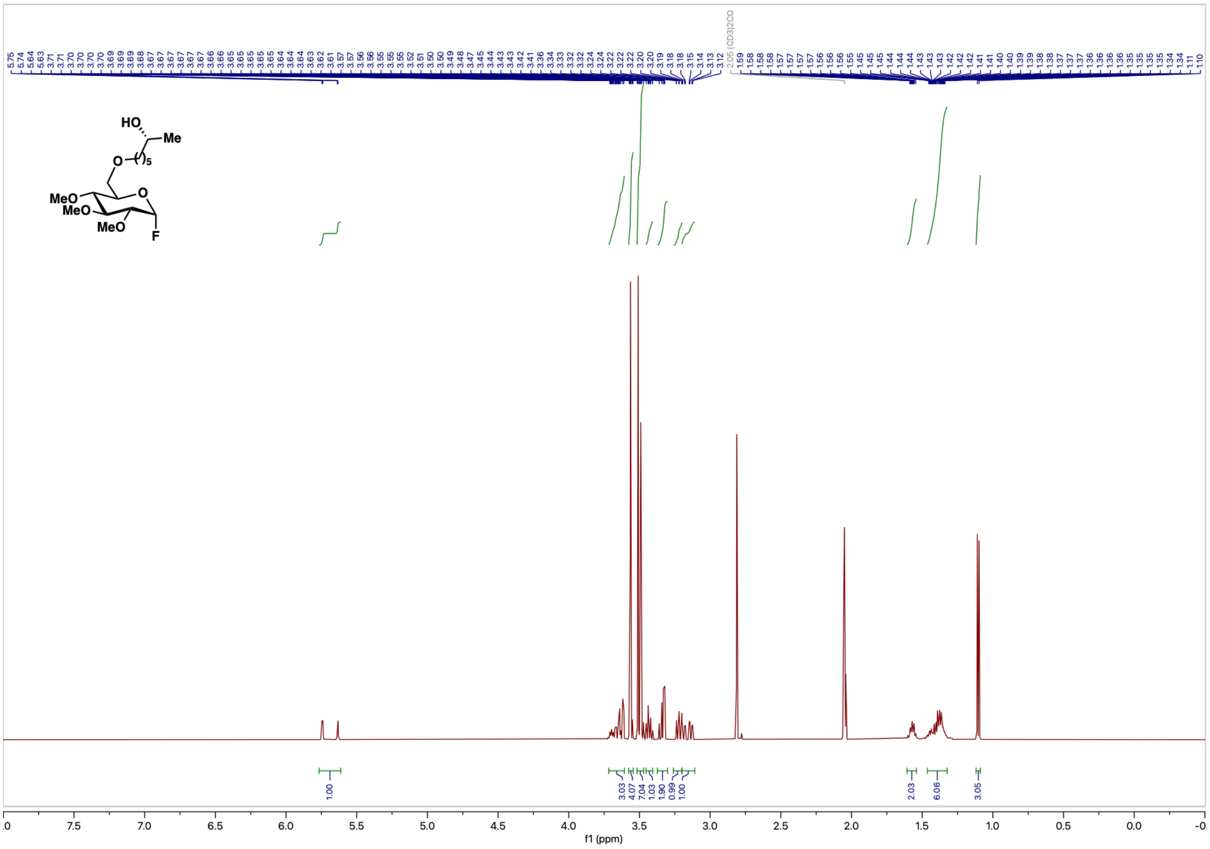

^{19}F NMR (470 MHz, acetone-d_6, 298K) of compound **1h**

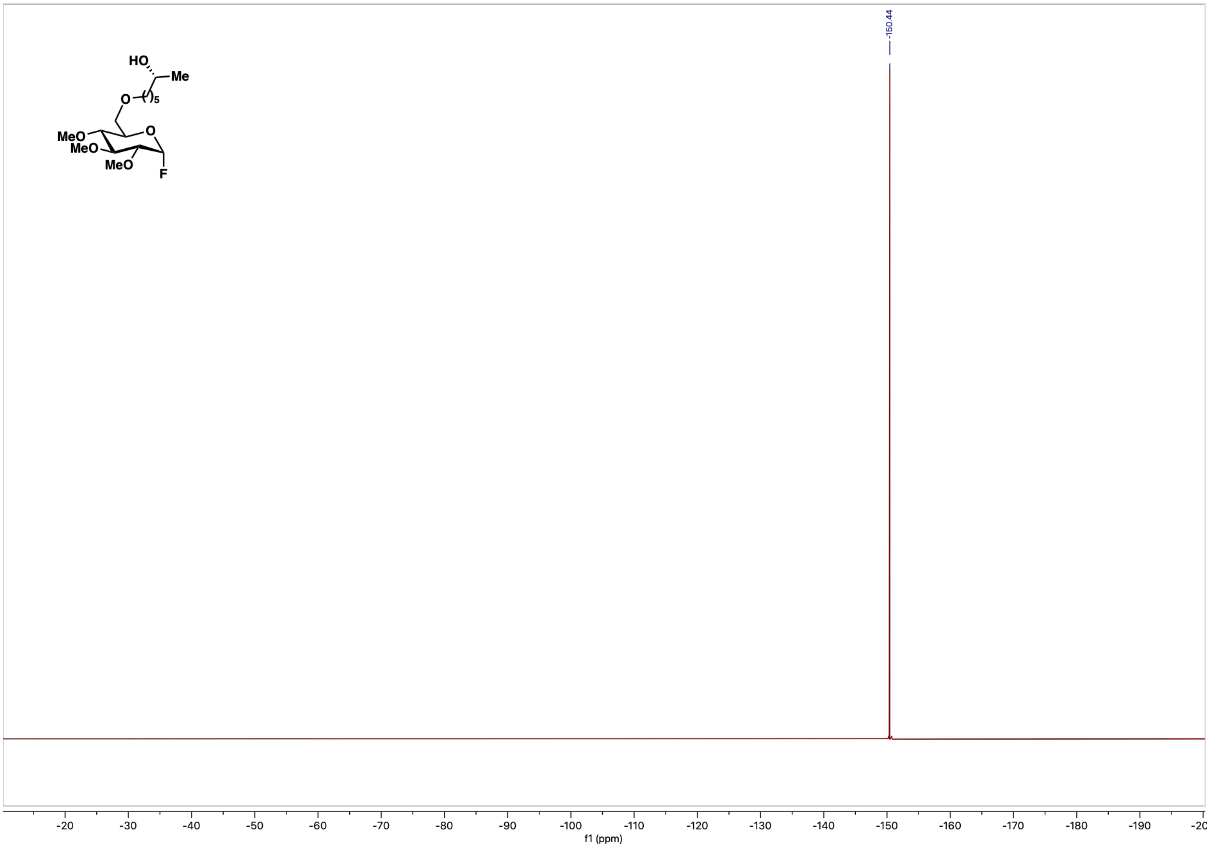

^{13}C NMR (126 MHz, acetone-d_6, 298K) of compound **1h**

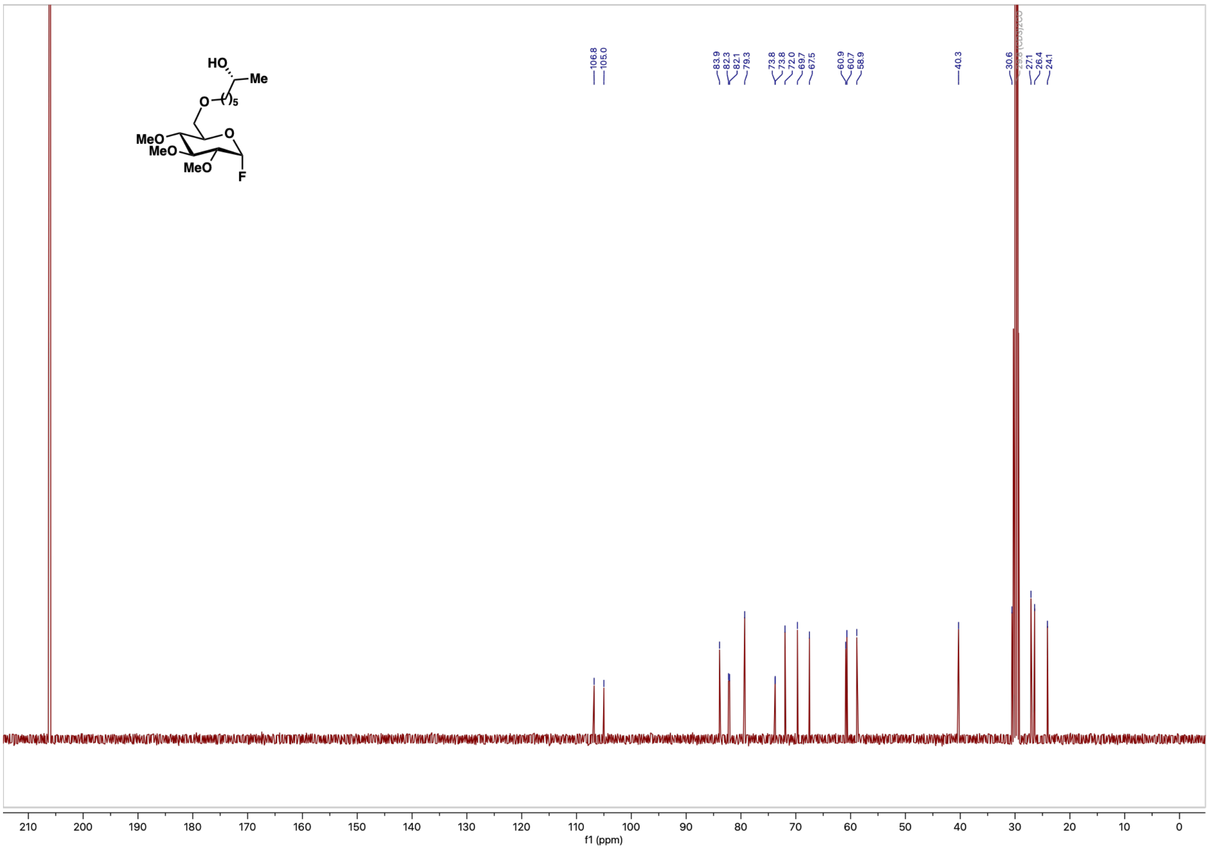

^{1}H NMR (500 MHz, CD_3OD, 298K) of compound **S7a**

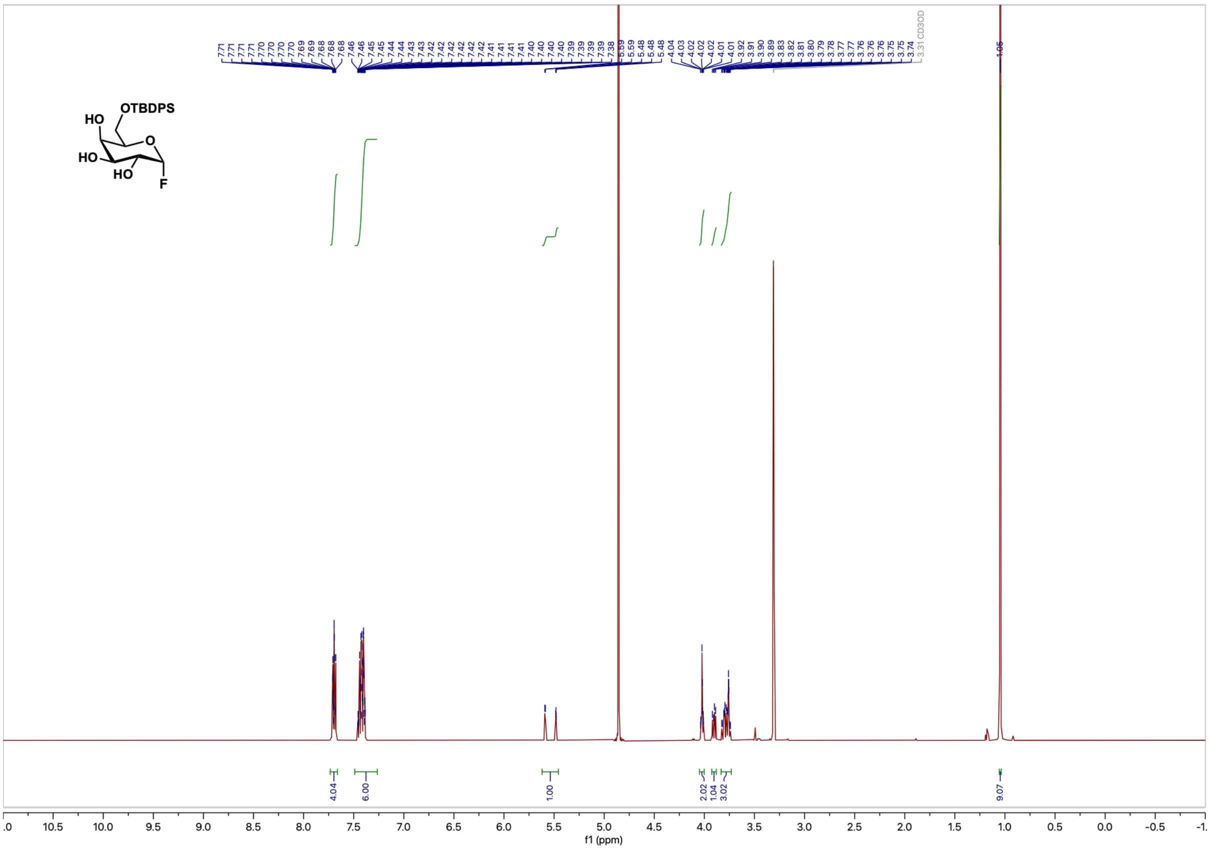

^{19}F NMR (470 MHz, CD_3OD, 298K) of compound **S7a**

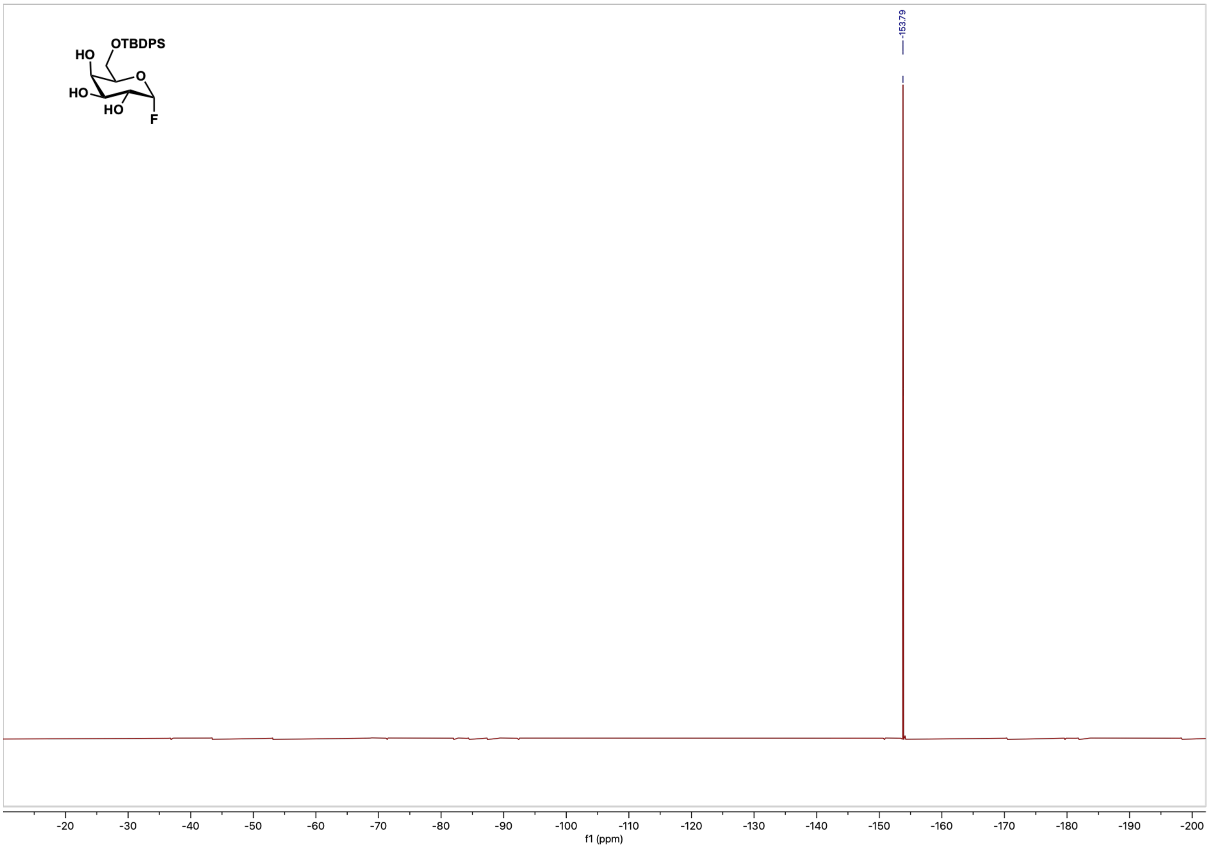

^{13}C NMR (126 MHz, CD_3OD, 298K) of compound **S7a**

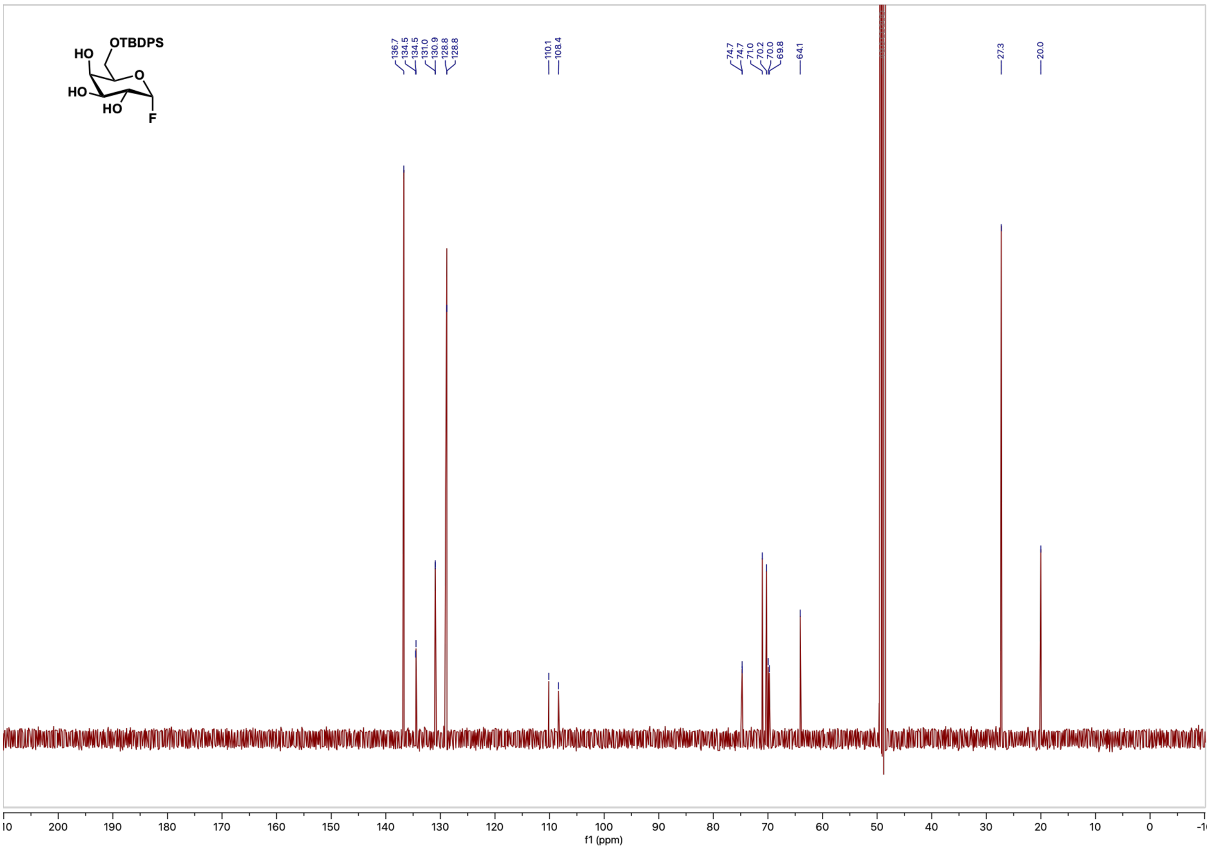

^{1}H NMR (500 MHz, $CDCl_3$, 298K) of compound **S8a**

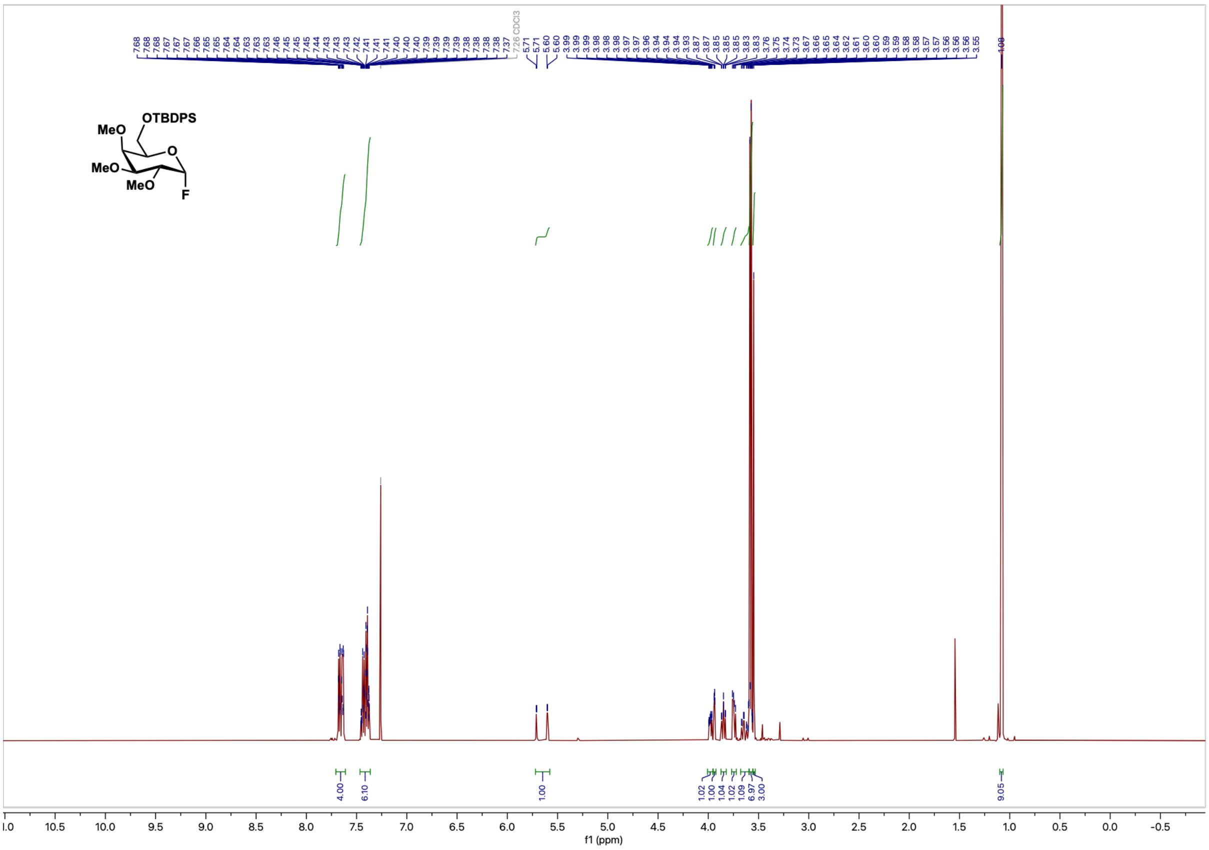

^{19}F NMR (470 MHz, $CDCl_3$, 298K) of compound **S8a**

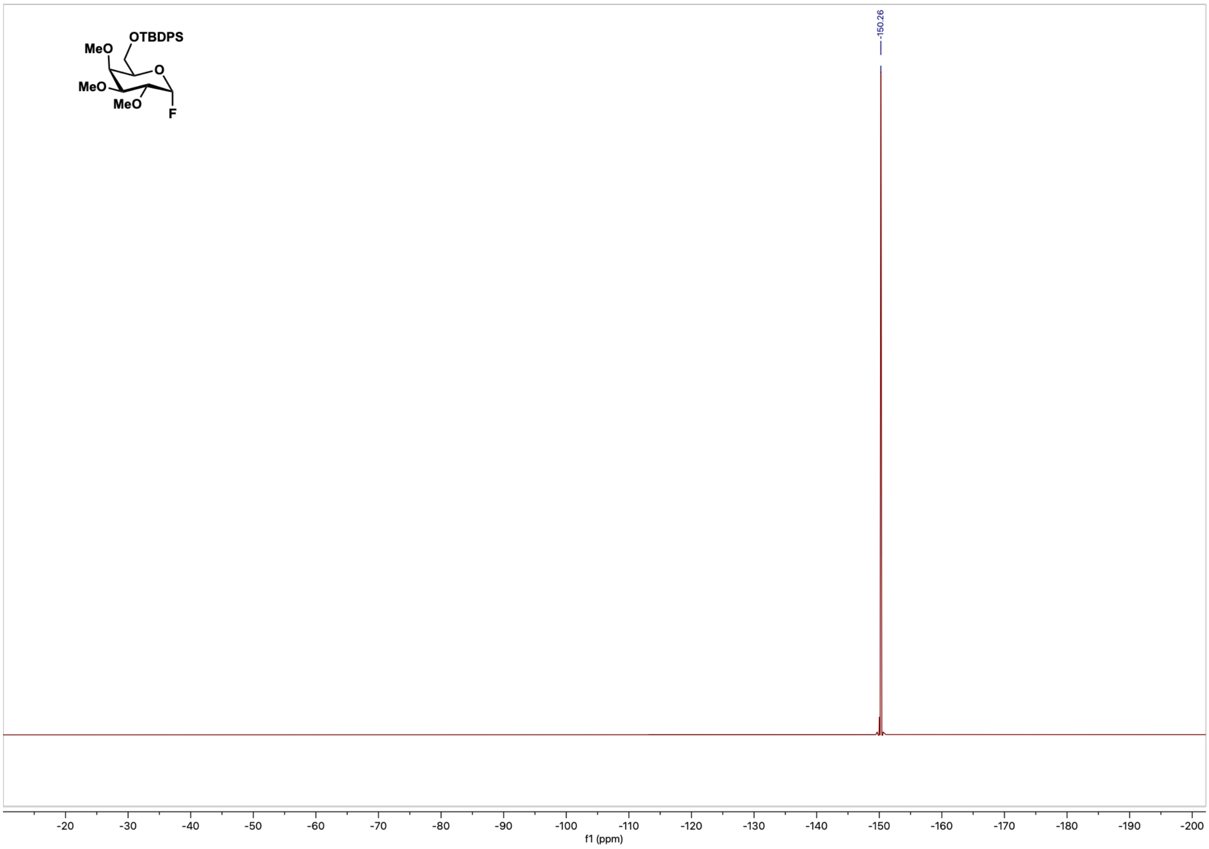

^{13}C NMR (126 MHz, $CDCl_3$, 298K) of compound **S8a**

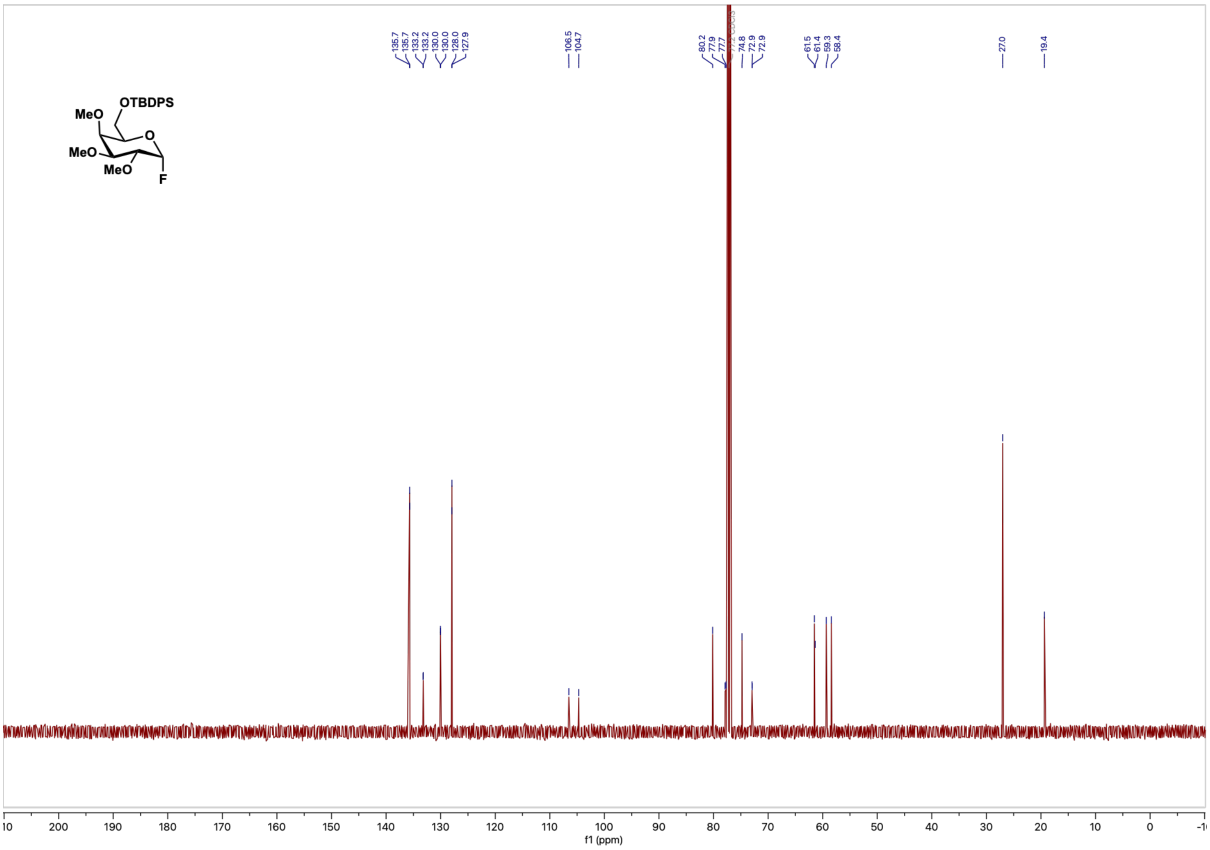

^{1}H NMR (500 MHz, CD_3OD, 298K) of compound **S9a**

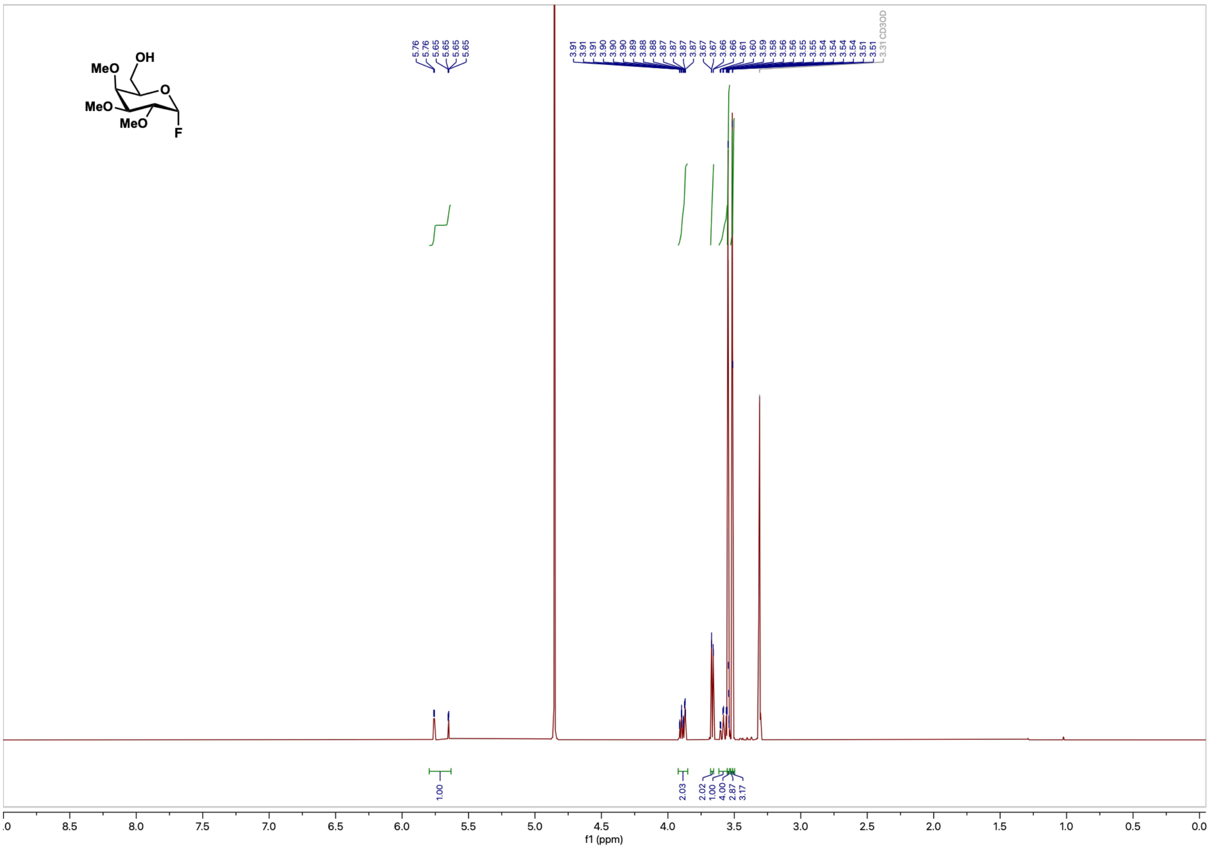

^{19}F NMR (470 MHz, CD_3OD, 298K) of compound **S9a**

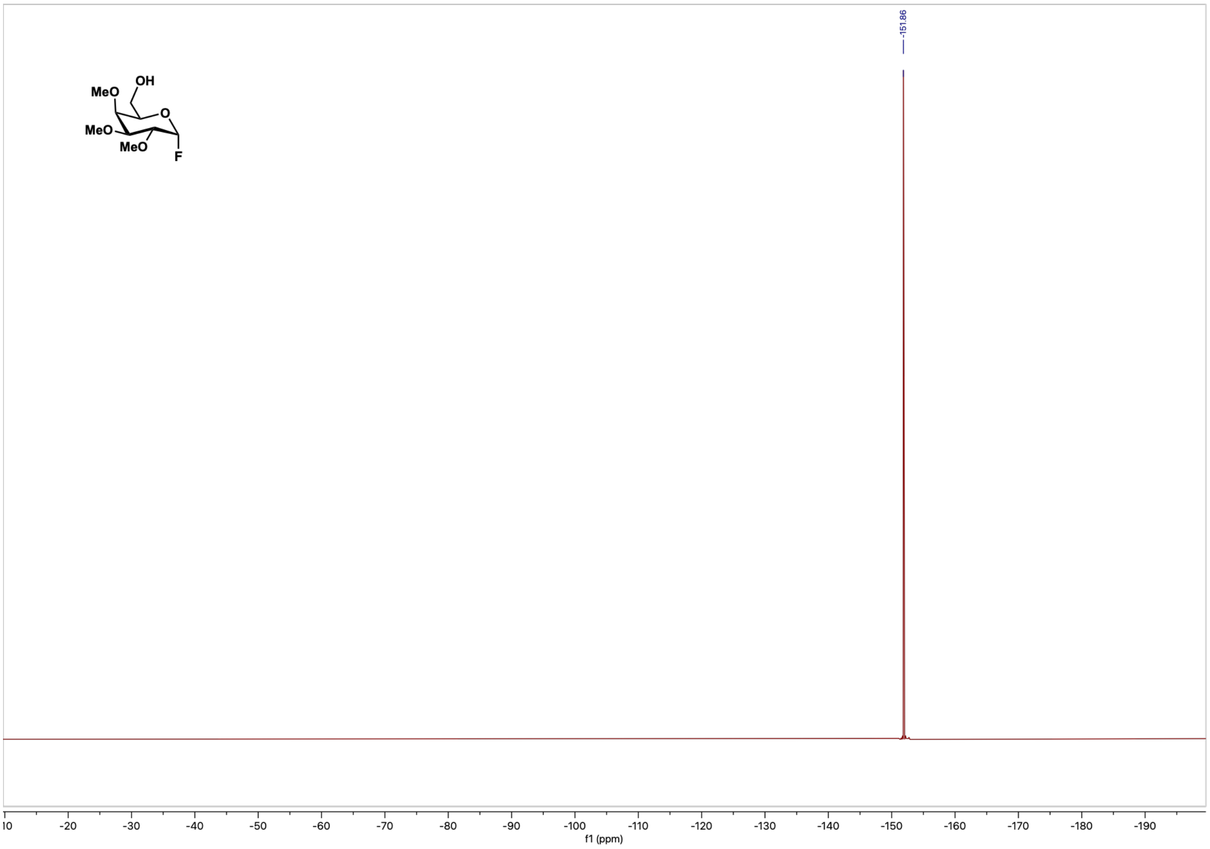

^{13}C NMR (126 MHz, CD_3OD, 298K) of compound **S9a**

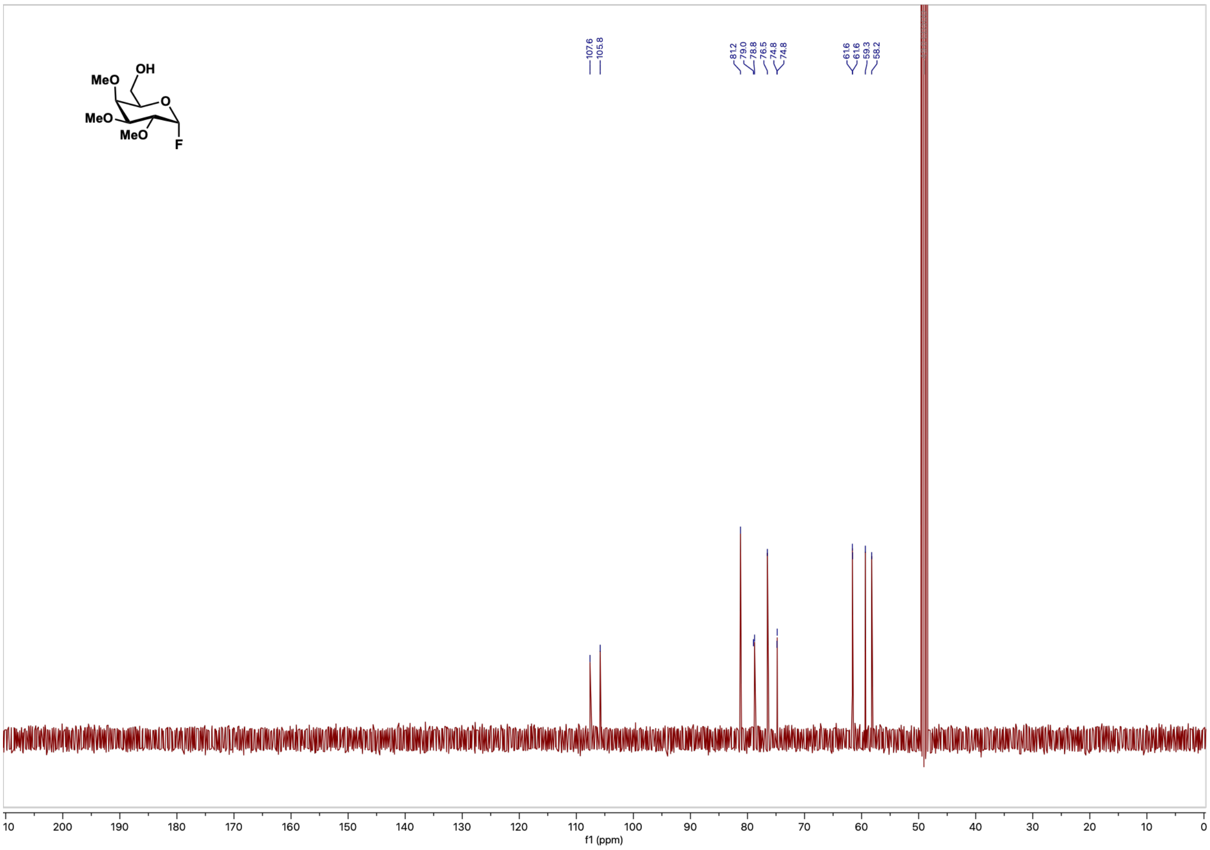

^{1}H NMR (500 MHz, $CDCl_3$, 298K) of compound **S10a**

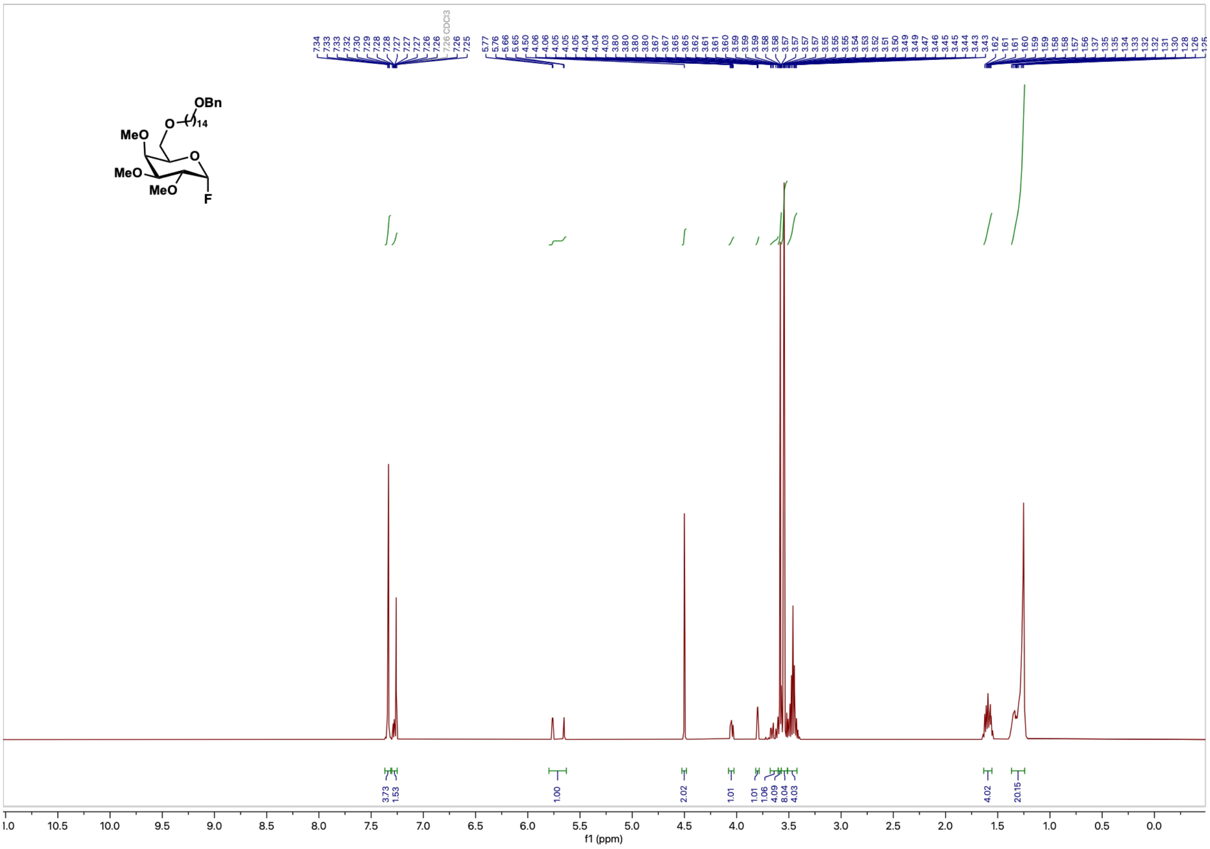

^{19}F NMR (470 MHz, $CDCl_3$, 298K) of compound **S10a**

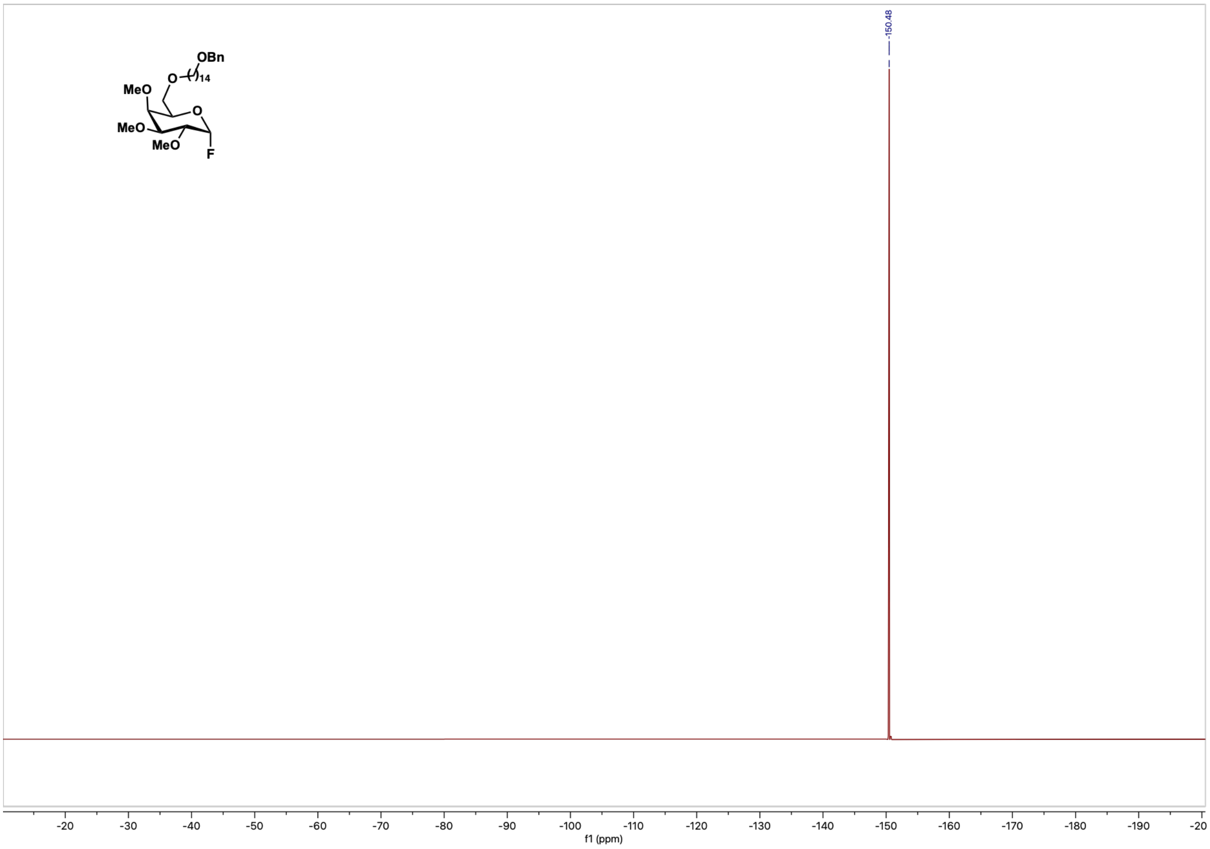

^{13}C NMR (126 MHz, $CDCl_3$, 298K) of compound **S10a**

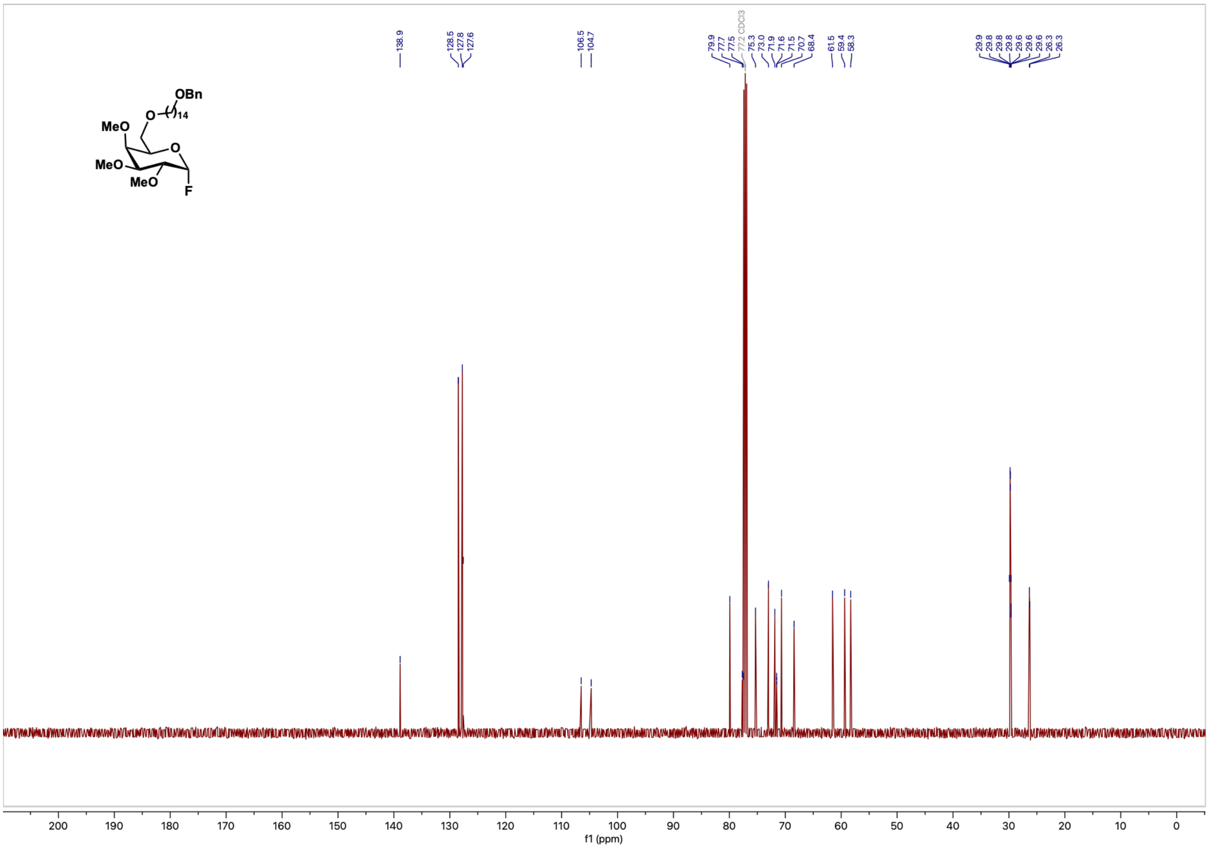

^{1}H NMR (500 MHz, acetone-d_6, 298K) of compound **1i**

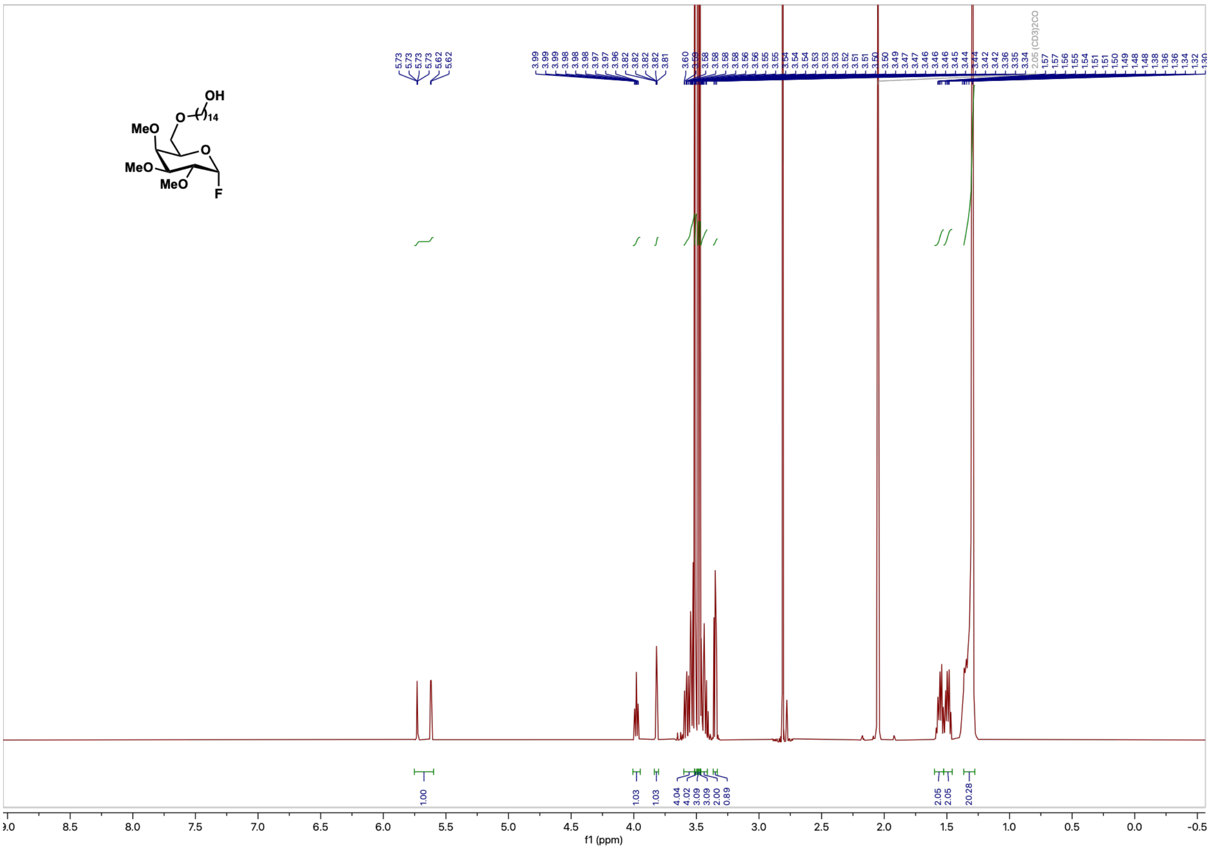

^{19}F NMR (470 MHz, acetone-d_6, 298K) of compound **1i**

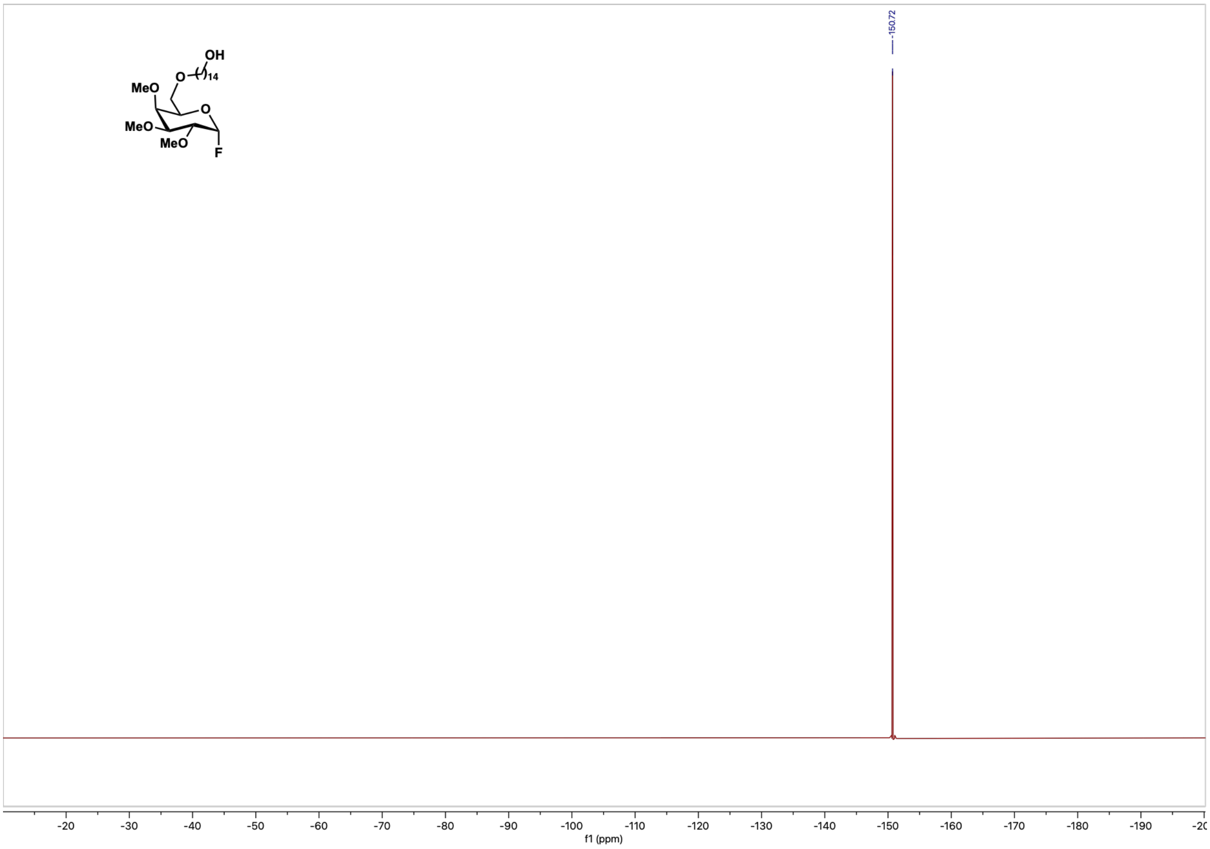

^{13}C NMR (126 MHz, acetone-d_6, 298K) of compound **1i**

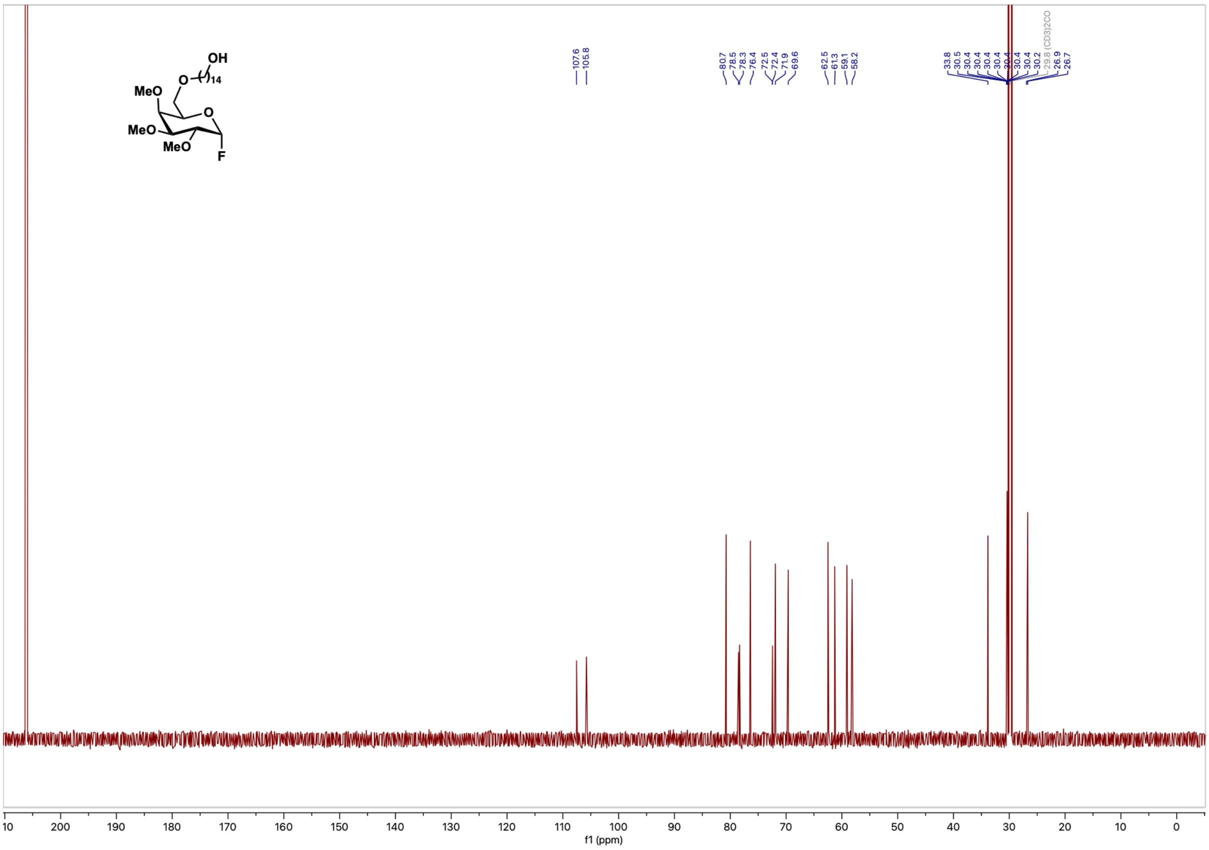

^{1}H NMR (500 MHz, CD_3OD, 298K) of compound **S7b**

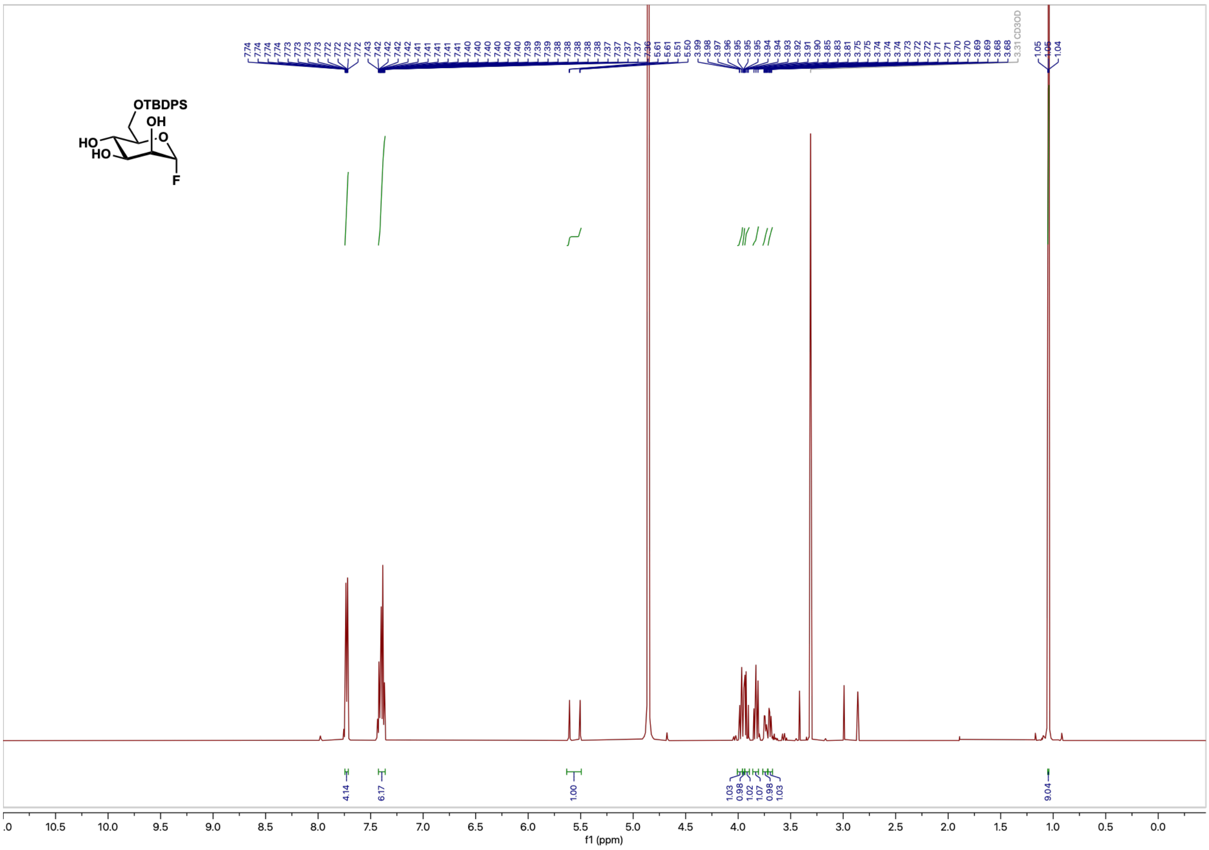

^{19}F NMR (470 MHz, CD_3OD, 298K) of compound **S7b**

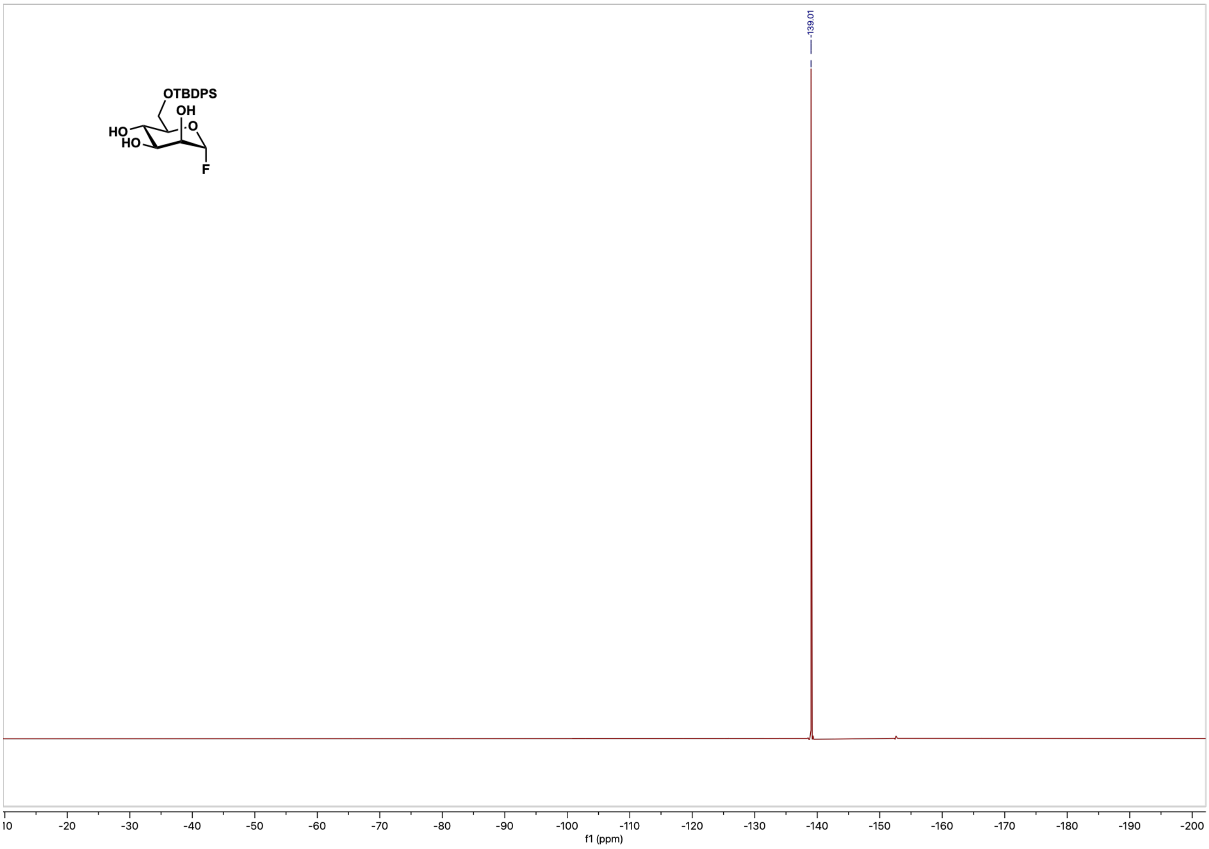

^{13}C NMR (126 MHz, CD_3OD, 298K) of compound **S7b**

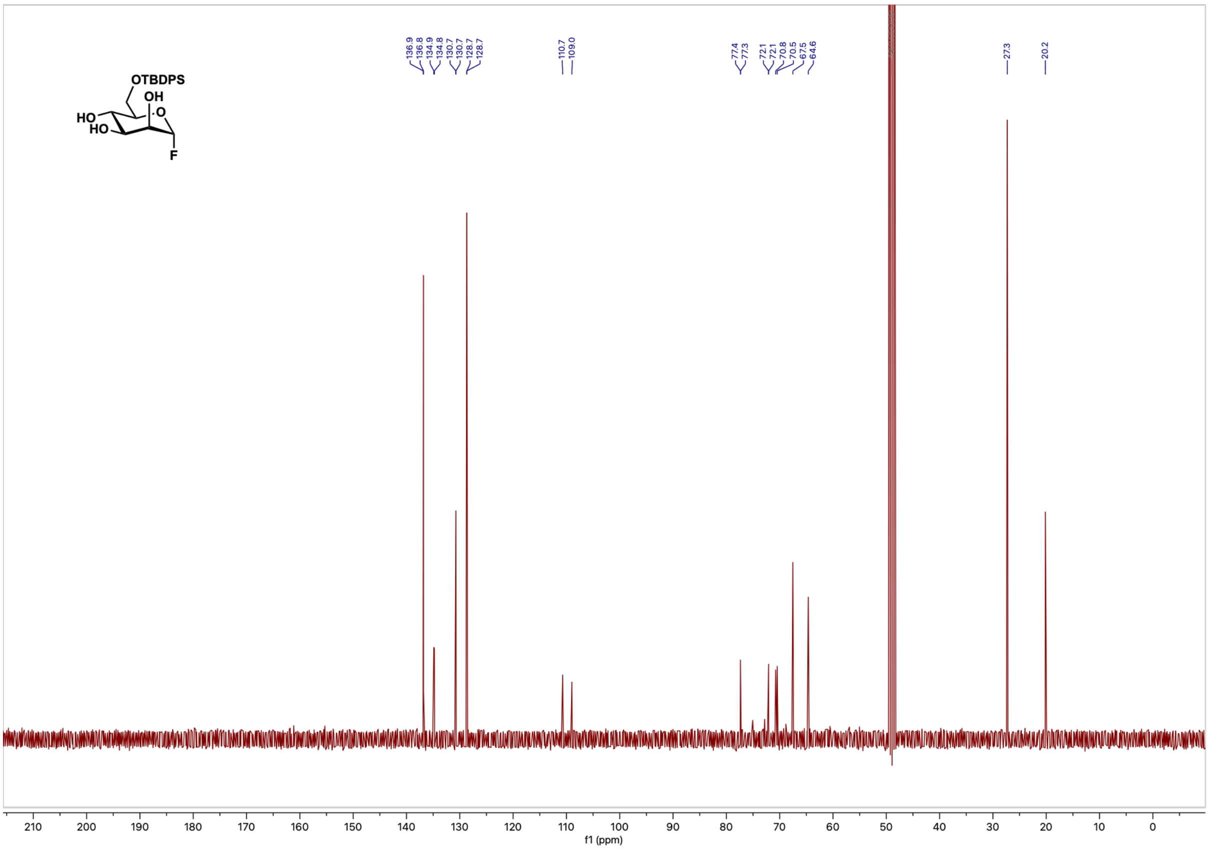

^{1}H NMR (500 MHz, $CDCl_3$, 298K) of compound **S8b**

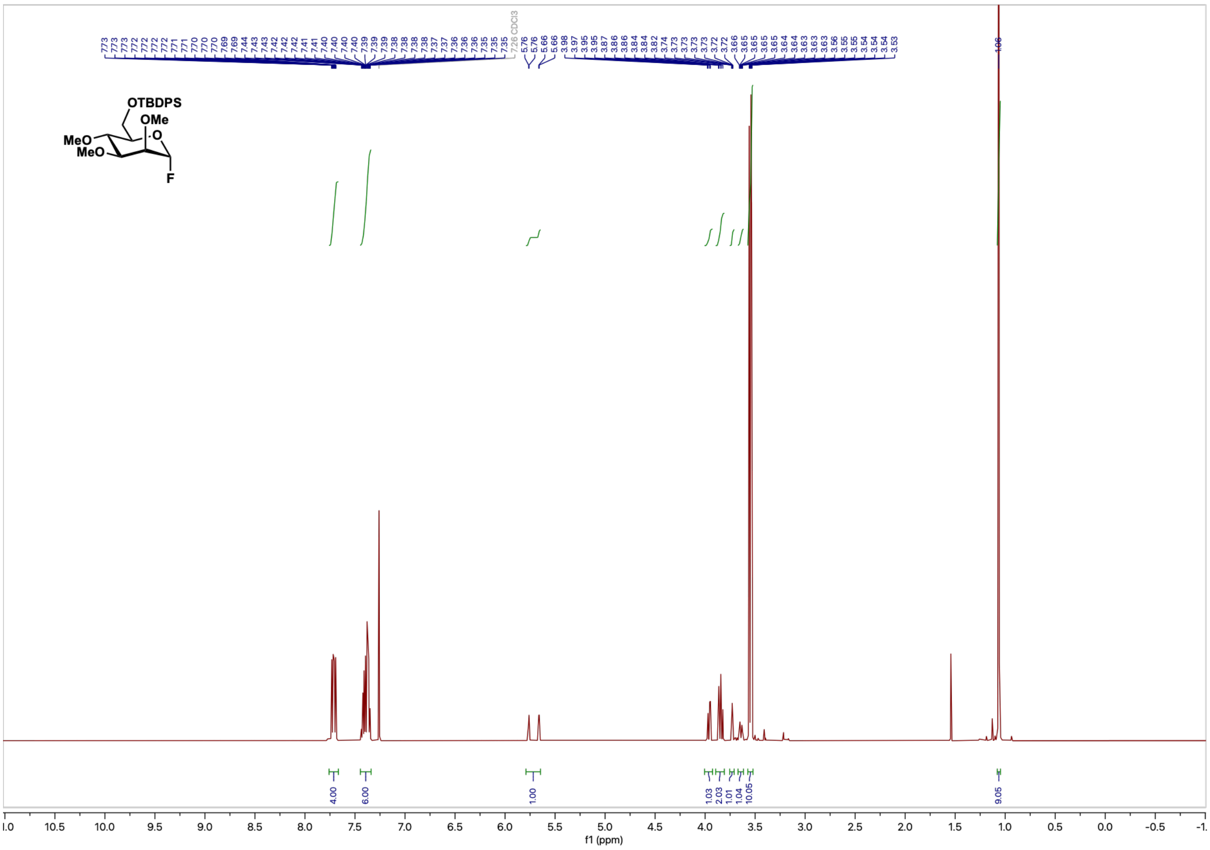

^{19}F NMR (470 MHz, $CDCl_3$, 298K) of compound **S8b**

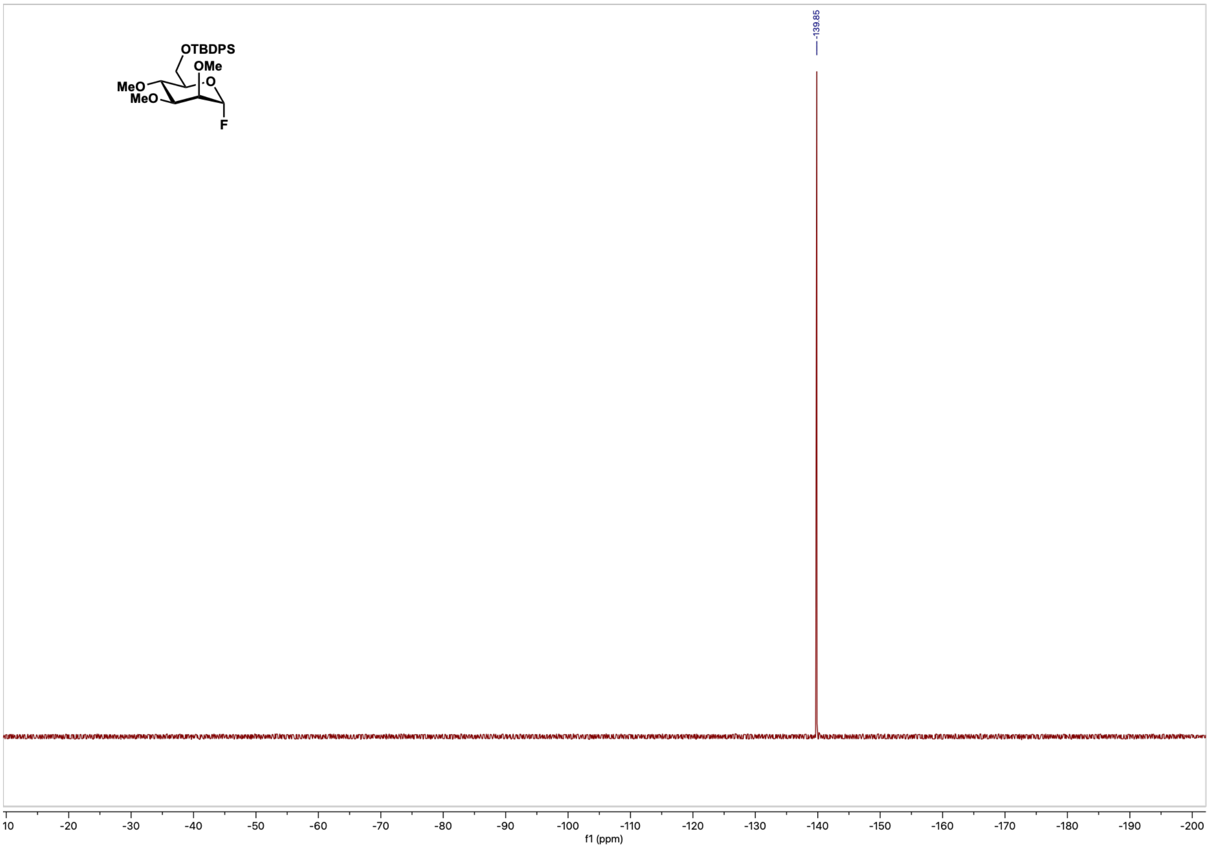

^{13}C NMR (126 MHz, $CDCl_3$, 298K) of compound **S8b**

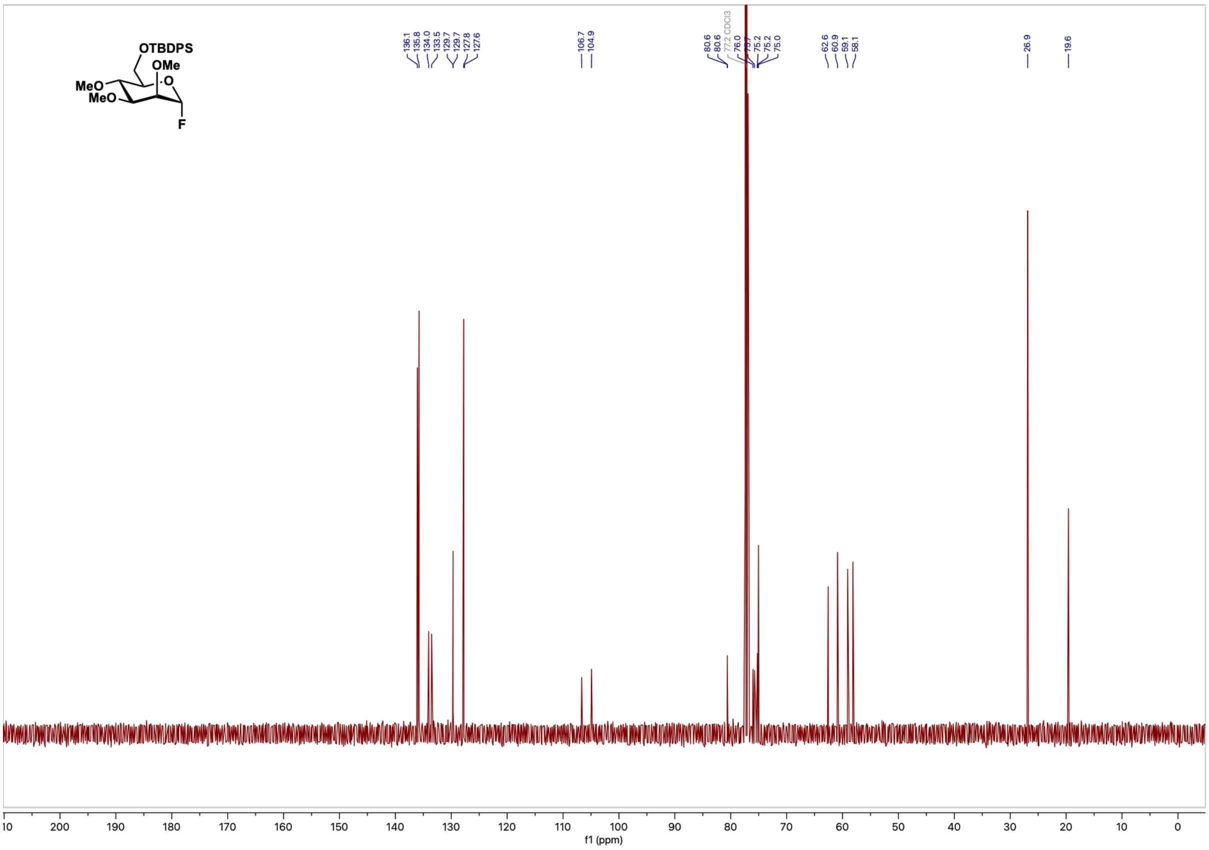

^{1}H NMR (500 MHz, CD_3OD, 298K) of compound **S9b**

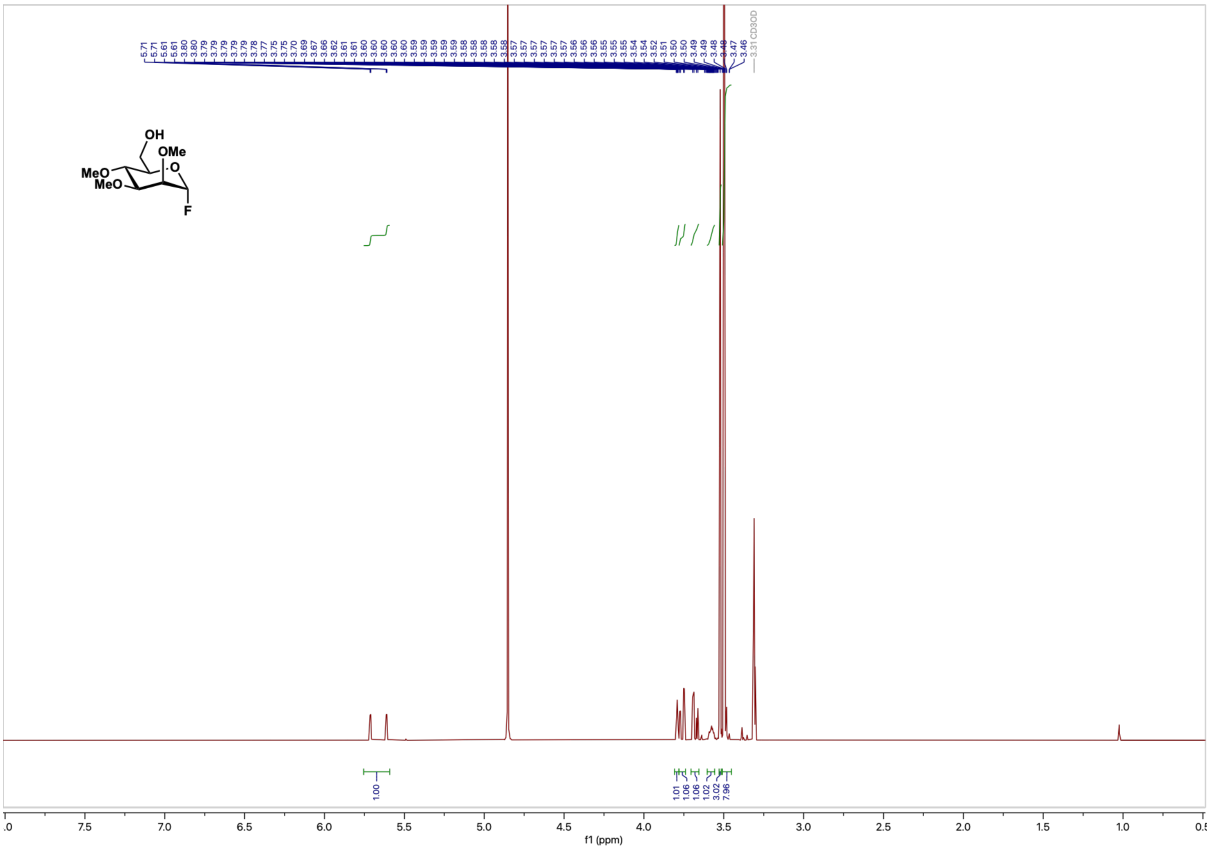

^{19}F NMR (470 MHz, CD_3OD, 298K) of compound **S9b**

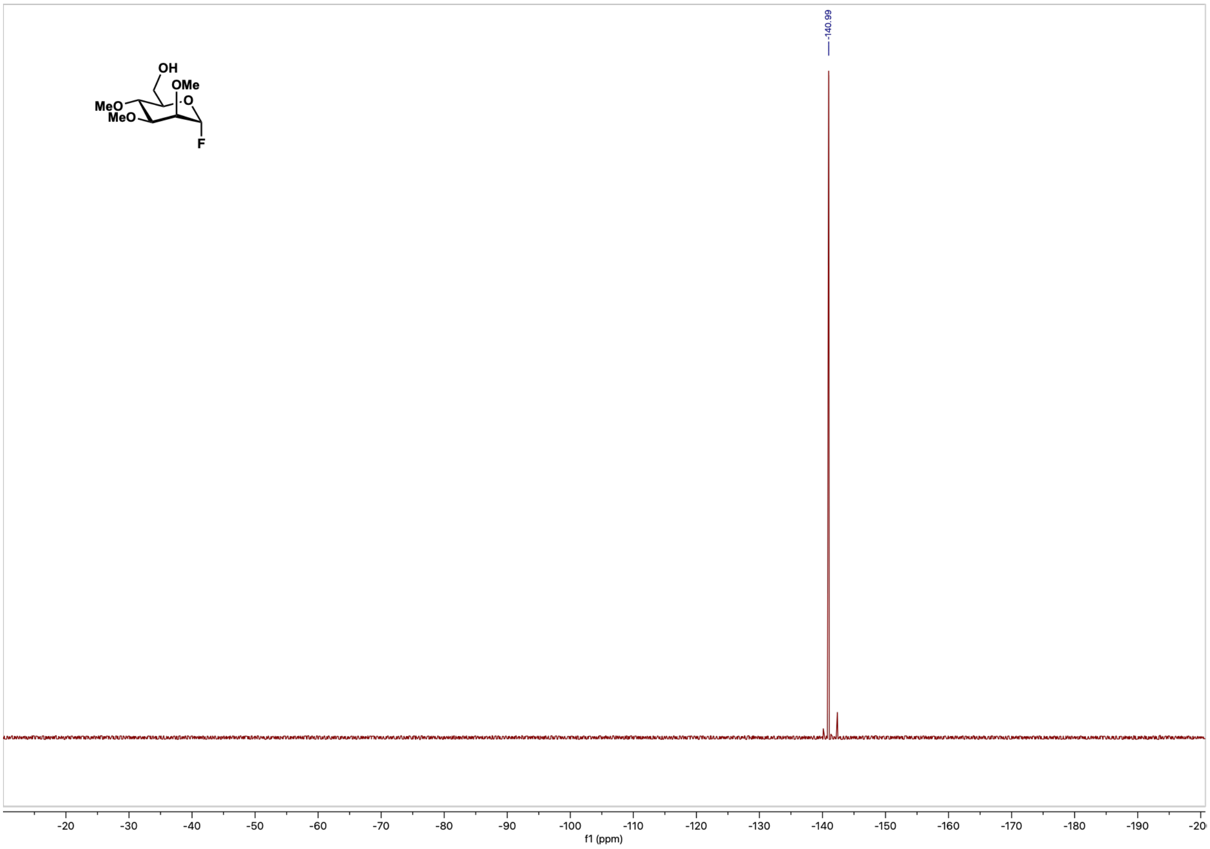

^{13}C NMR (126 MHz, CD_3OD, 298K) of compound **S9b**

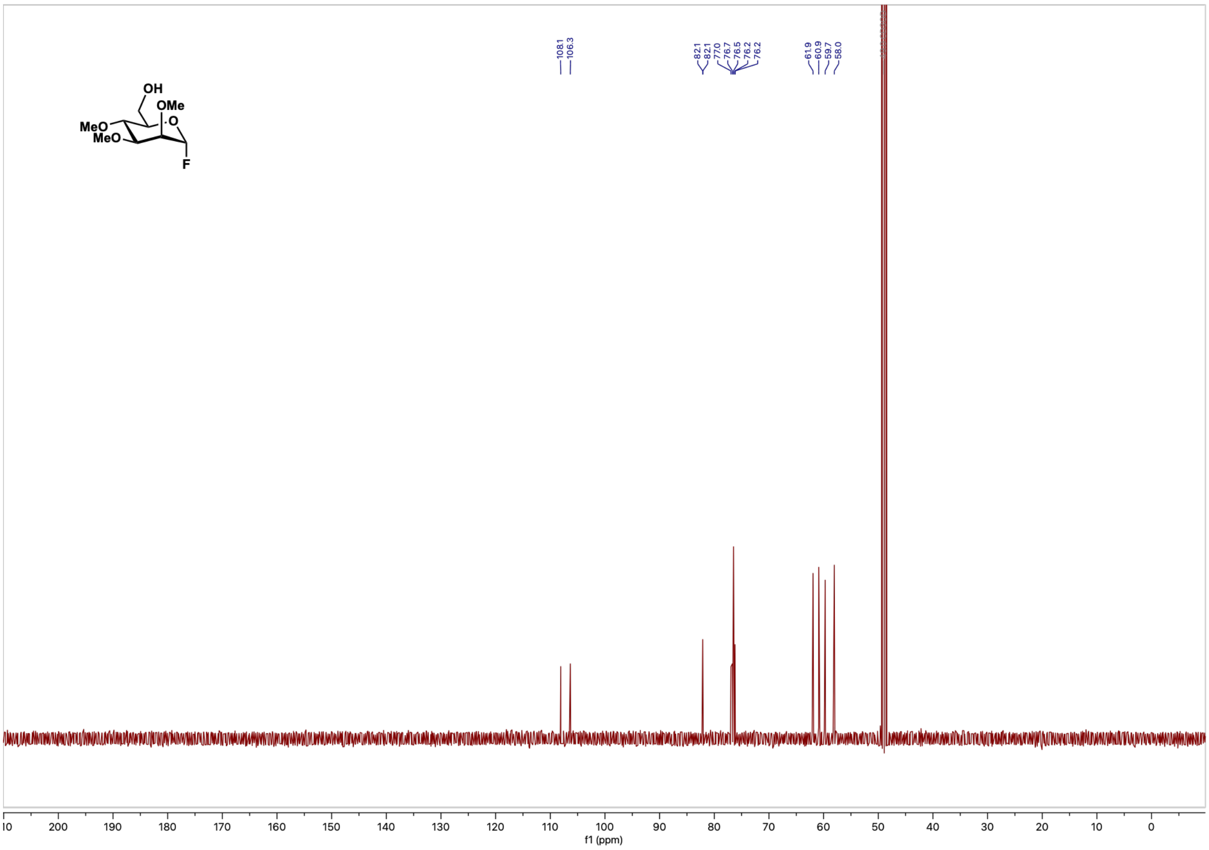

^{1}H NMR (500 MHz, $CDCl_3$, 298K) of compound **S10b**

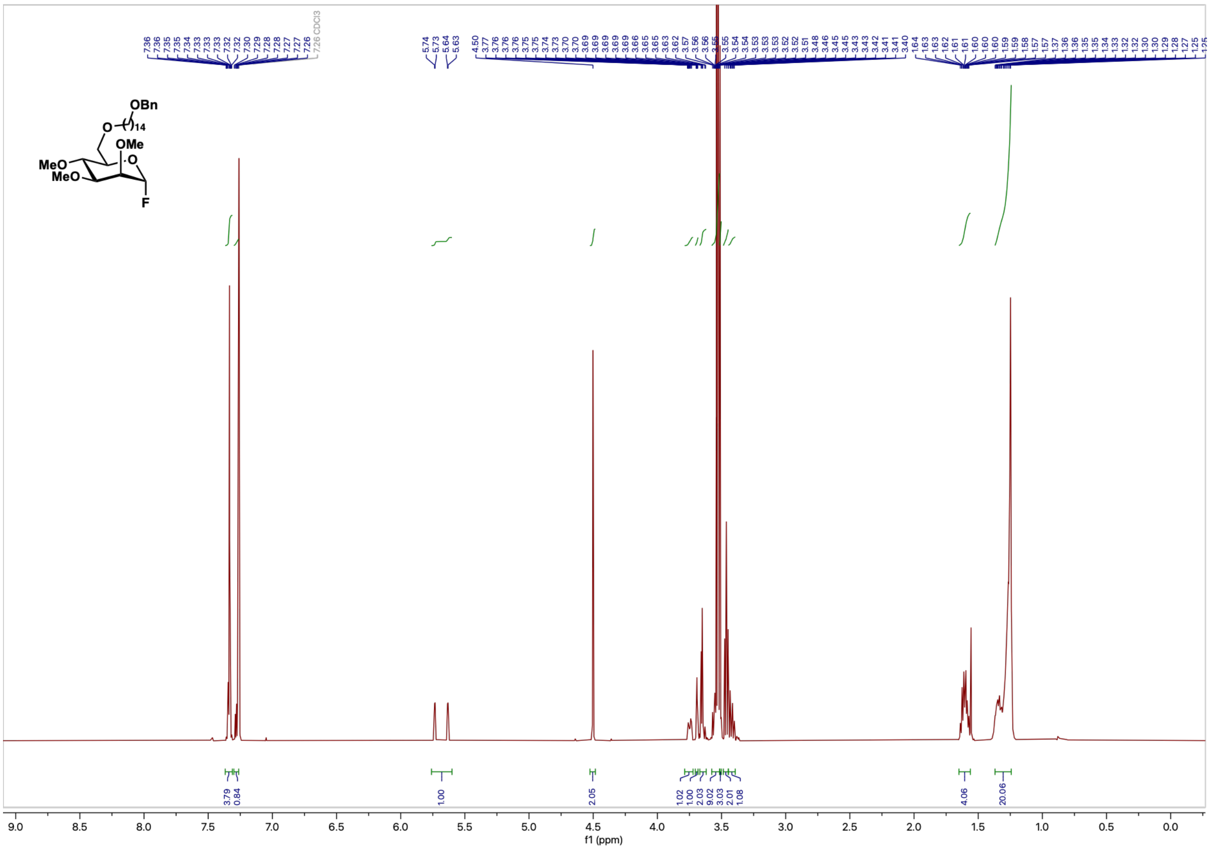

^{19}F NMR (470 MHz, $CDCl_3$, 298K) of compound **S10b**

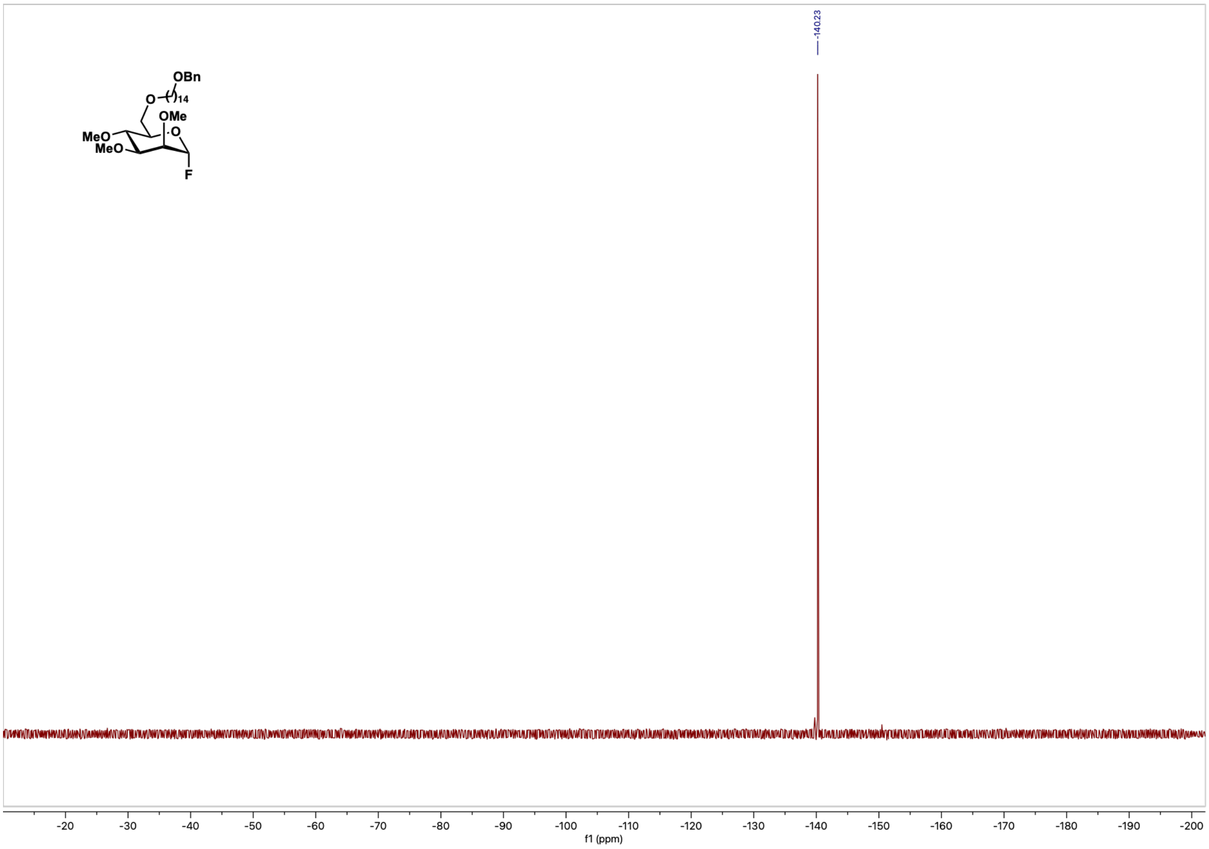

^{13}C NMR (126 MHz, $CDCl_3$, 298K) of compound **S10b**

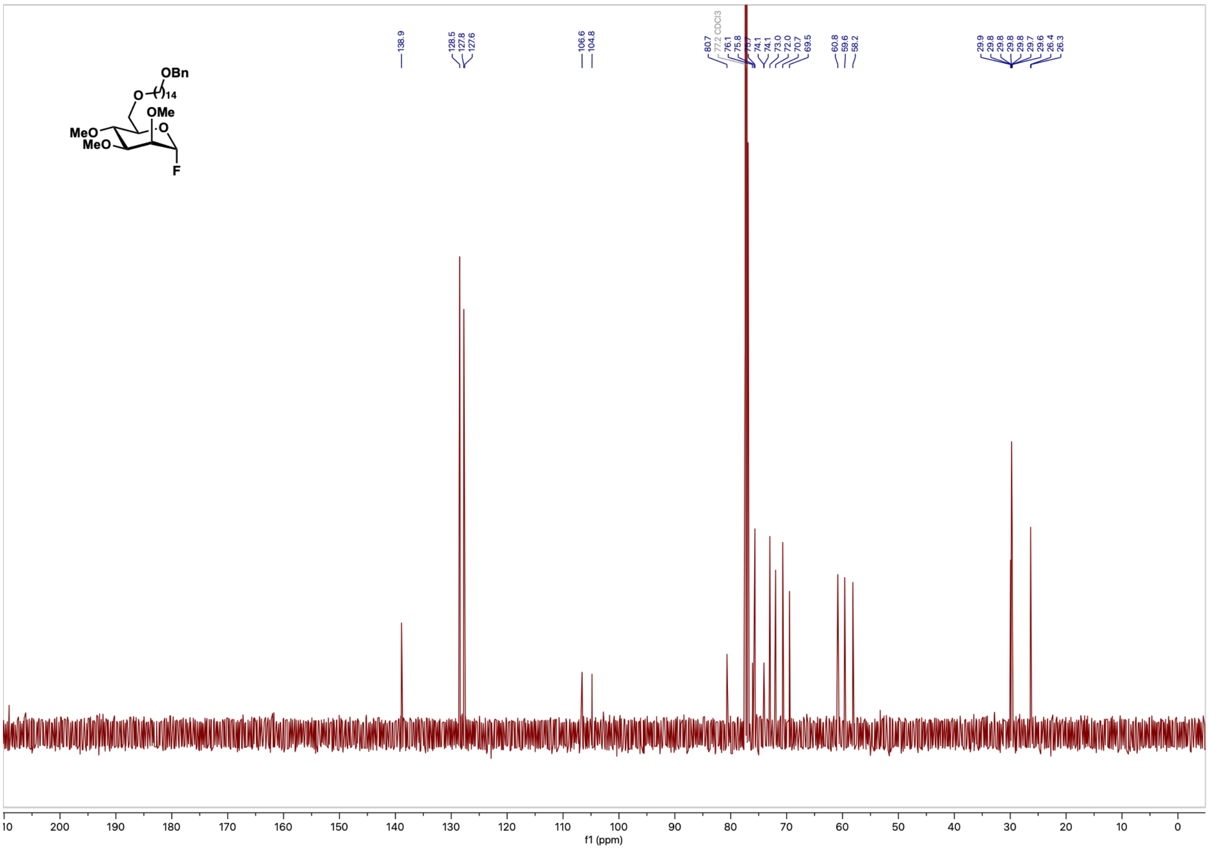

^{1}H NMR (500 MHz, acetone-d_6, 298K) of compound **1j**

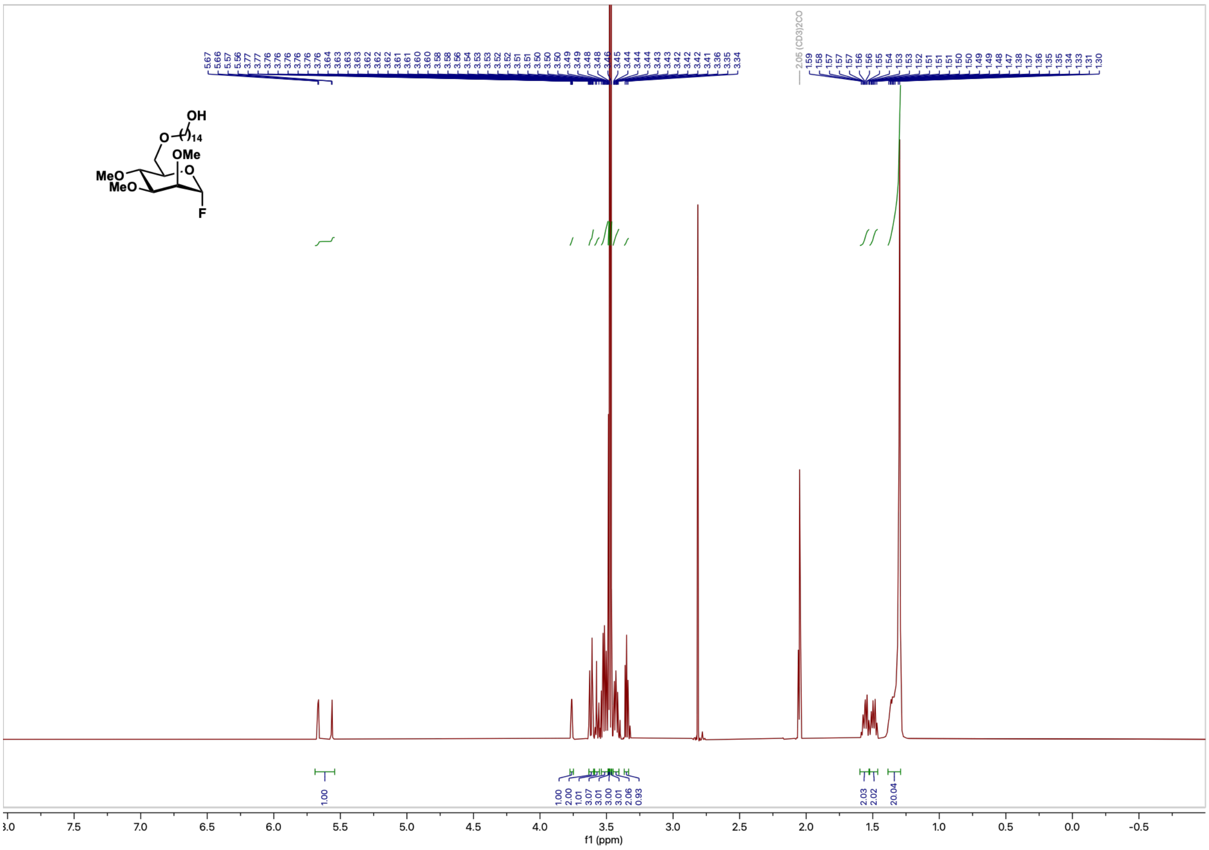

^{19}F NMR (470 MHz, acetone-d_6, 298K) of compound **1j**

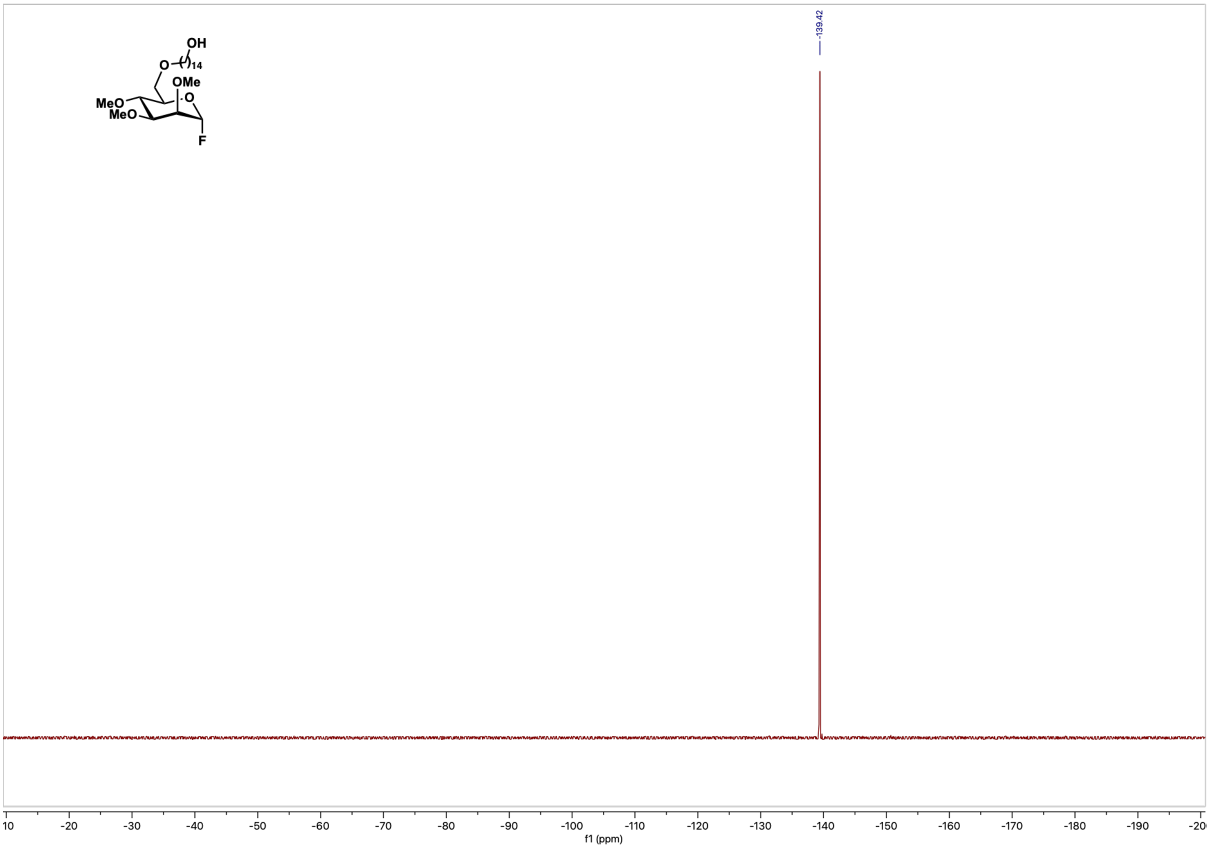

^{13}C NMR (126 MHz, acetone-d_6, 298K) of compound **1j**

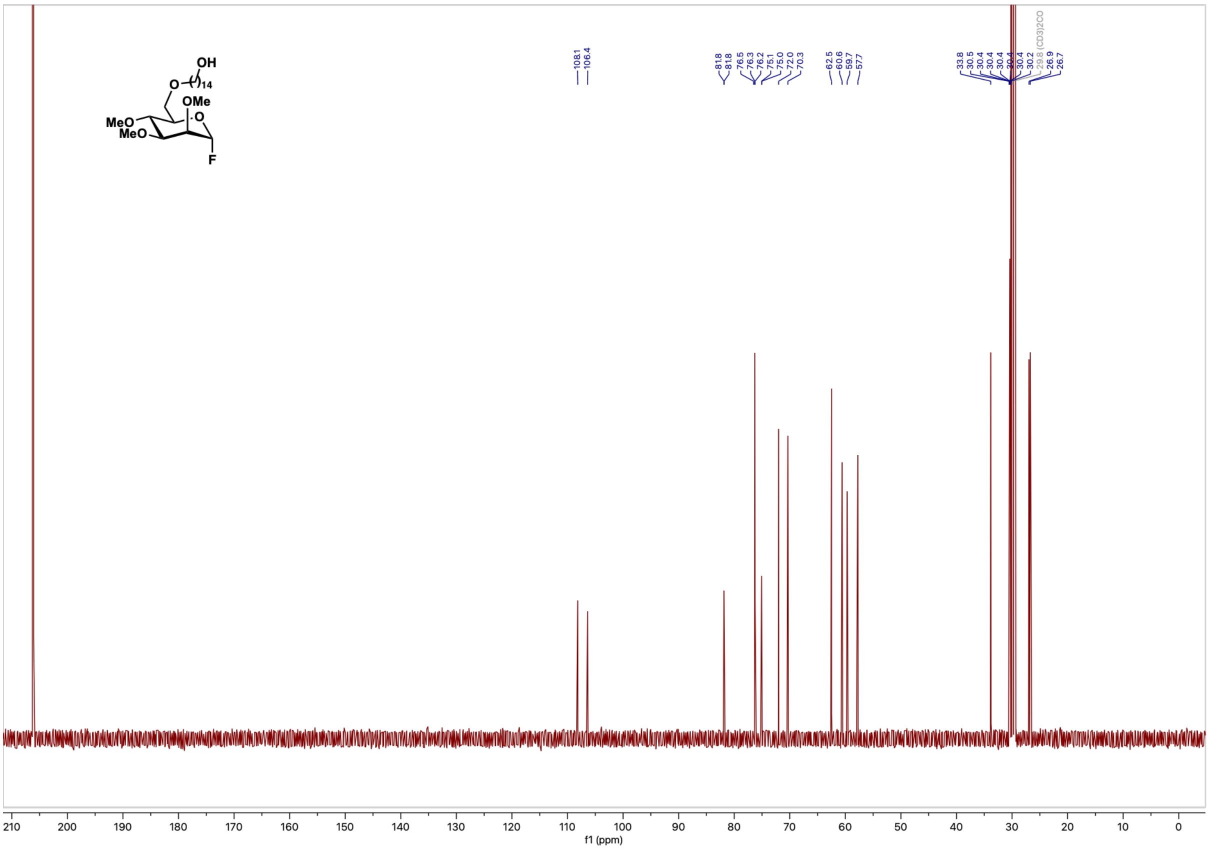

^{1}H NMR (500 MHz, $CDCl_3$, 298K) of compound **S7**

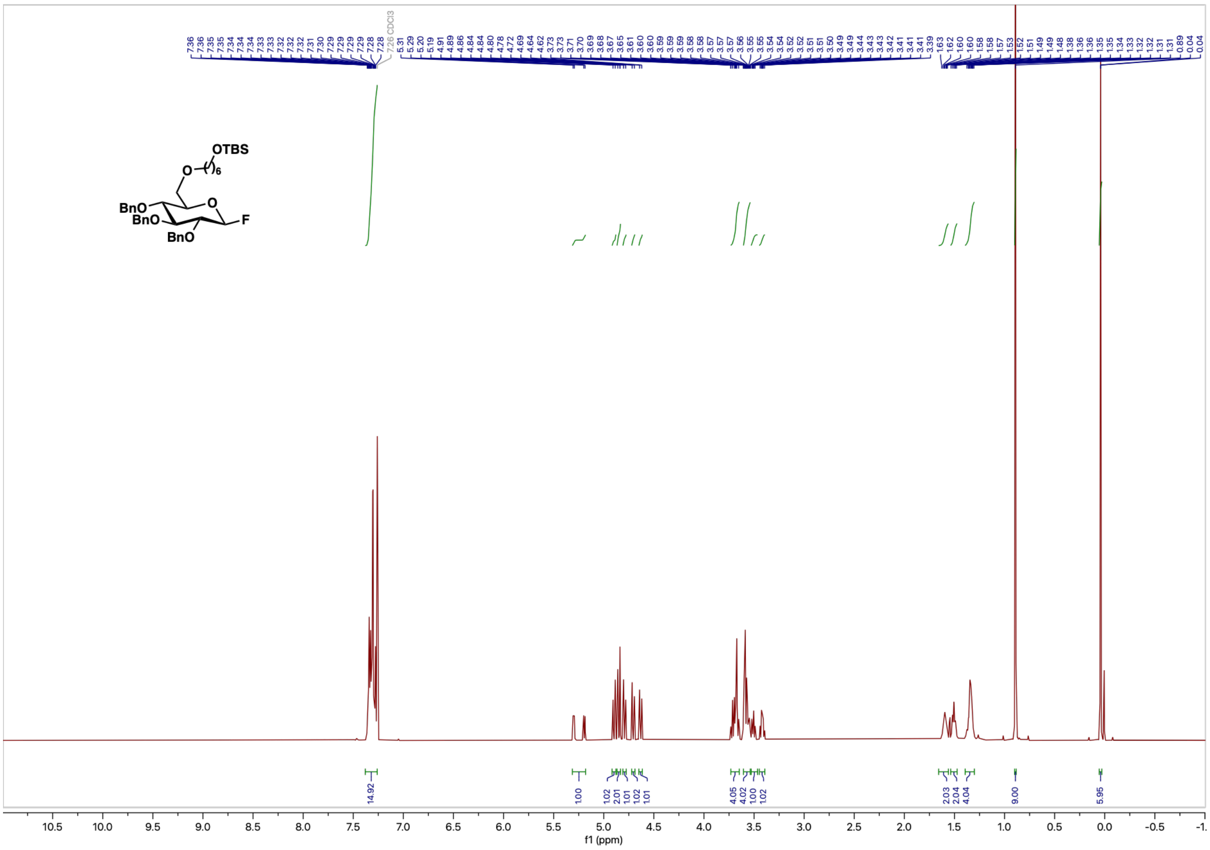

^{19}F NMR (470 MHz, $CDCl_3$, 298K) of compound **S7**

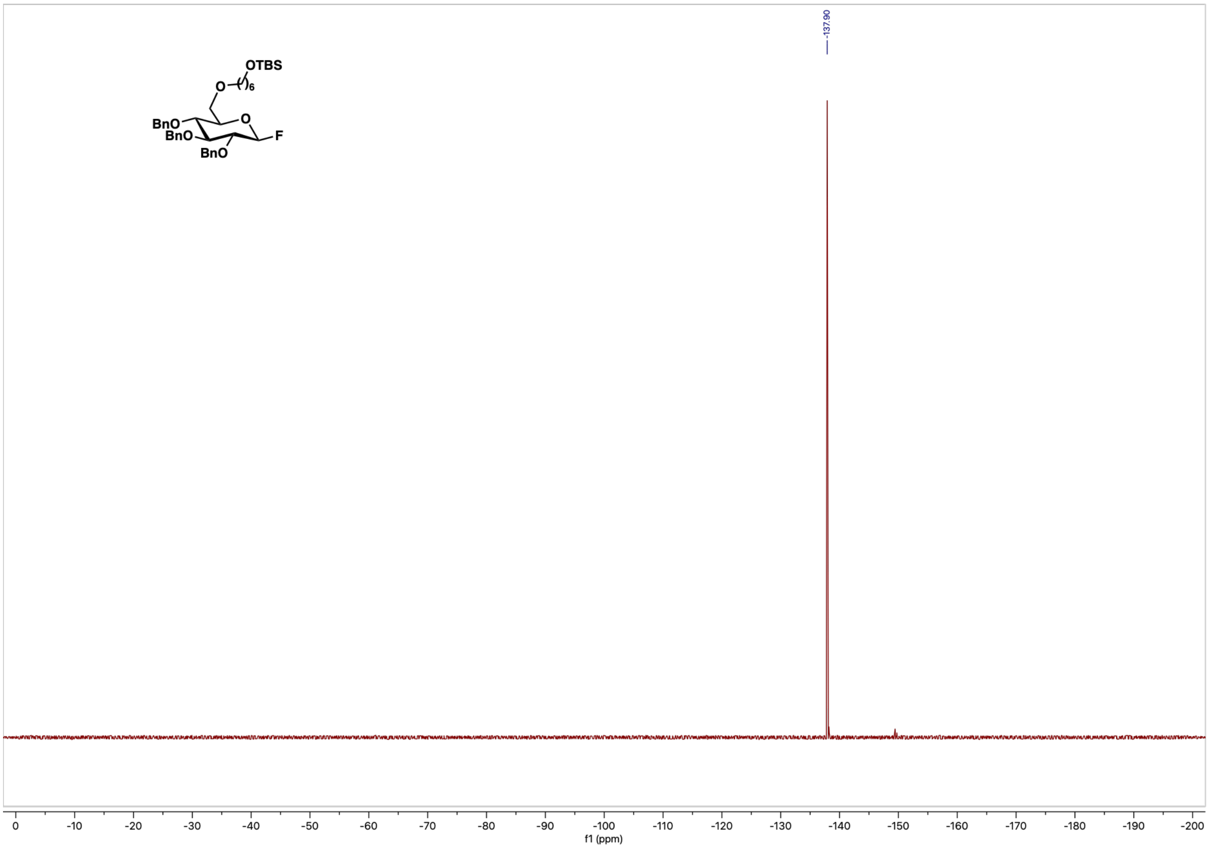

^{13}C NMR (126 MHz, $CDCl_3$, 298K) of compound **S7**

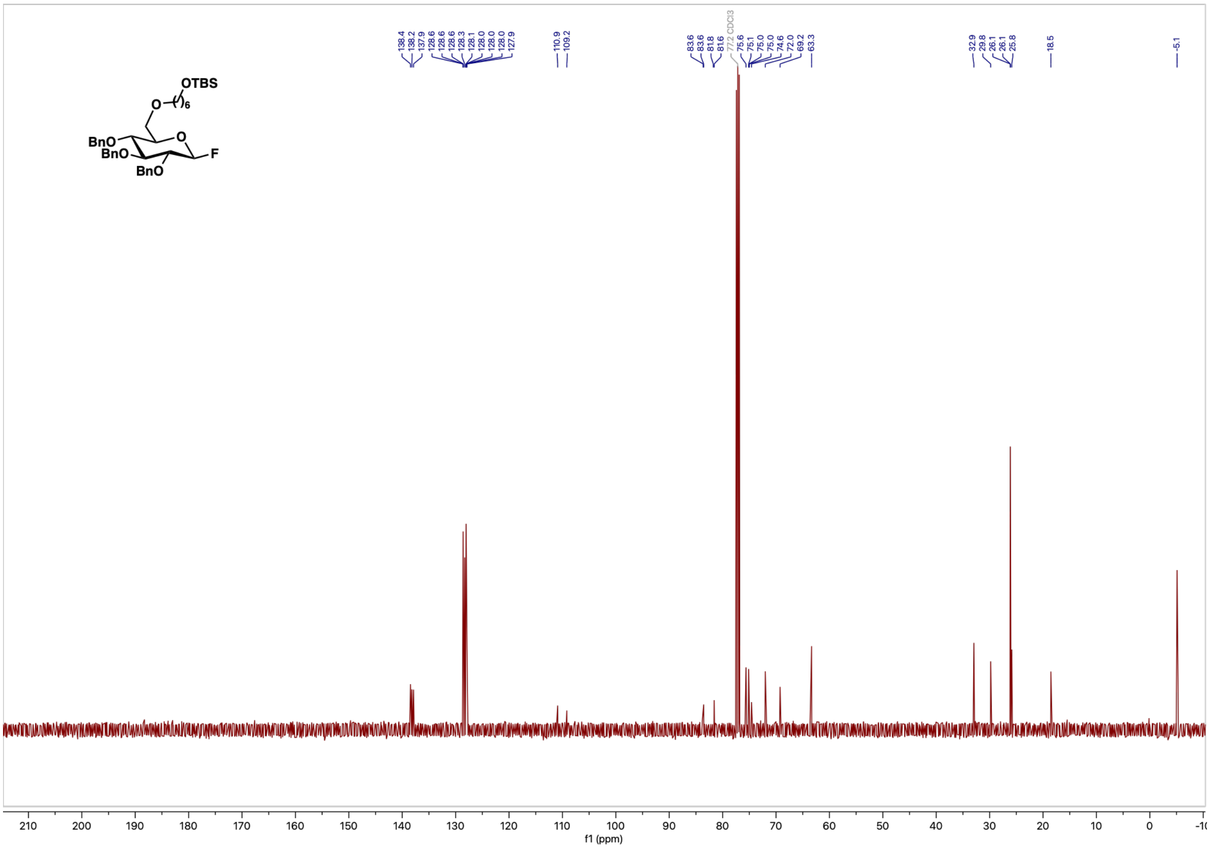

^{1}H NMR (500 MHz, $CDCl_3$, 298K) of compound **S8**

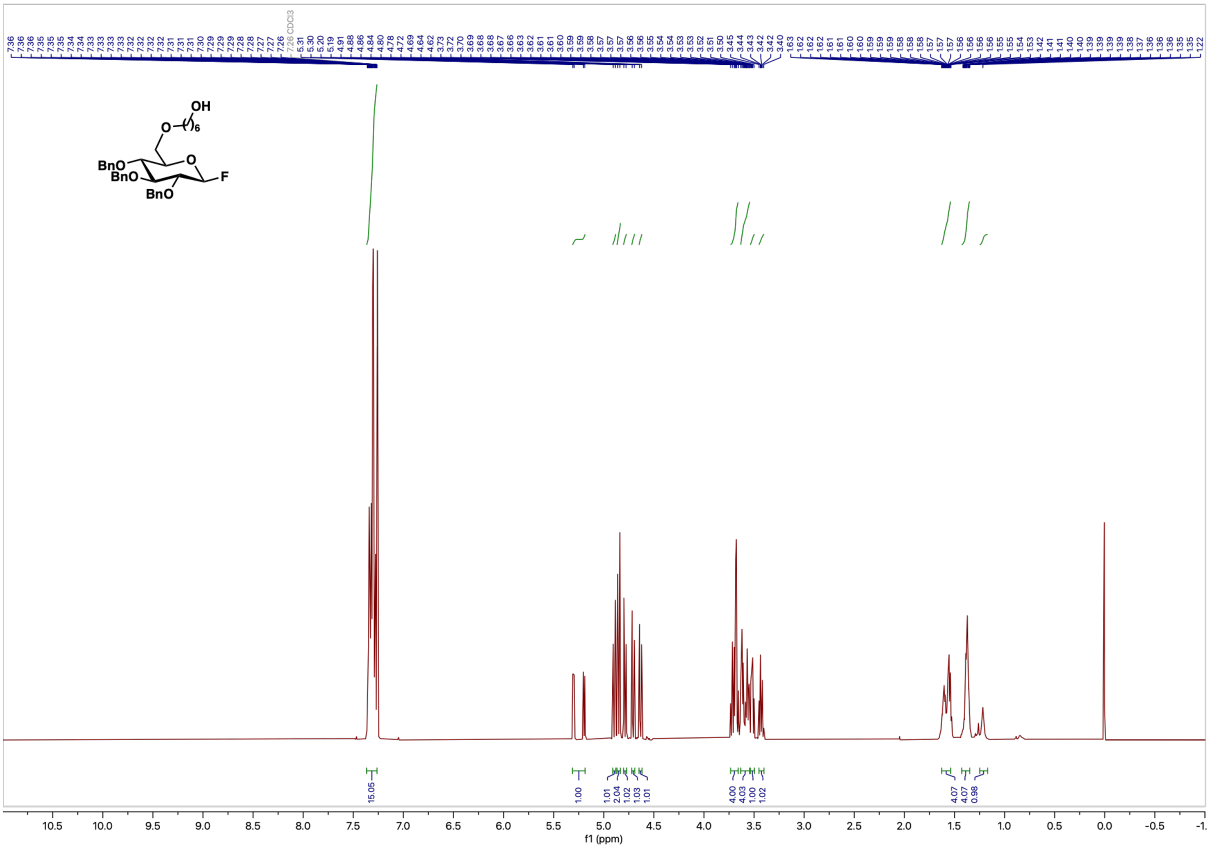

^{19}F NMR (470 MHz, CDCl$_3$, 298K) of compound **S8**

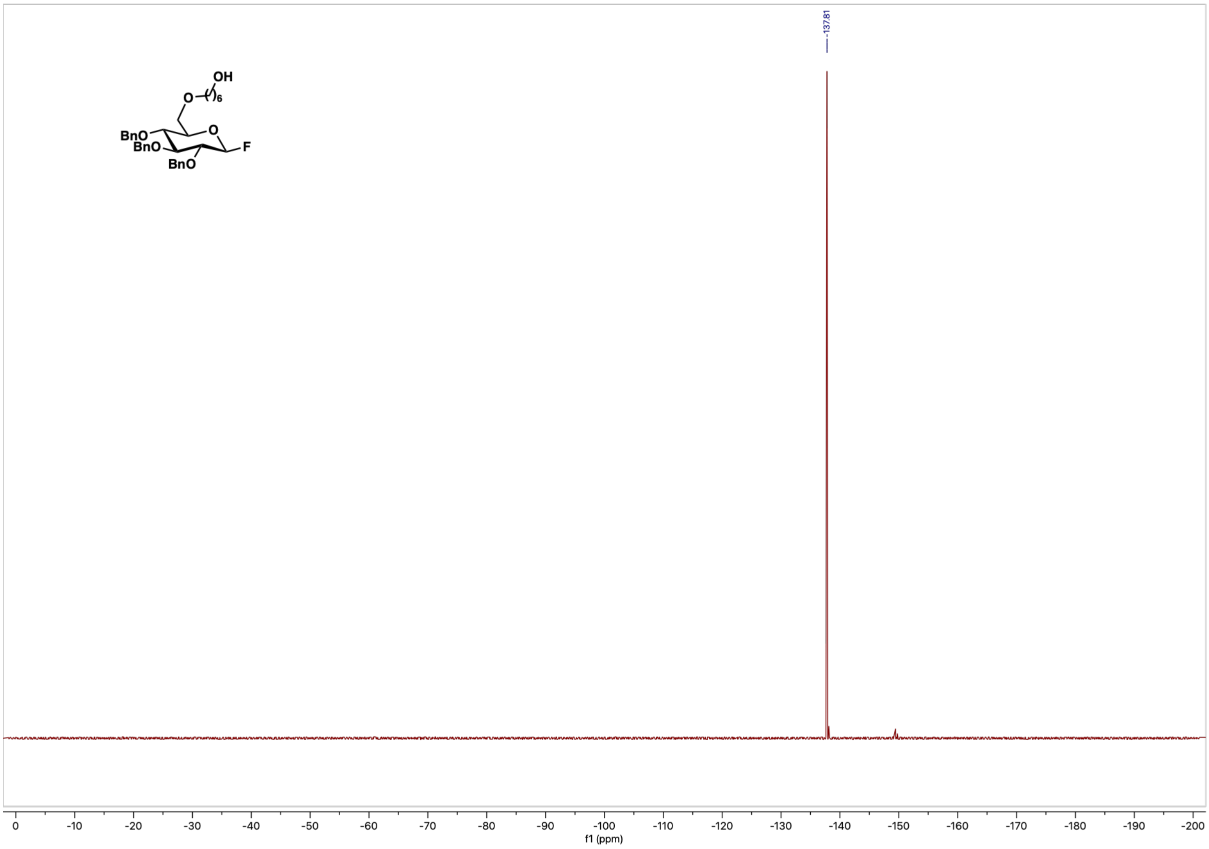

^{13}C NMR (126 MHz, CDCl$_3$, 298K) of compound **S8**

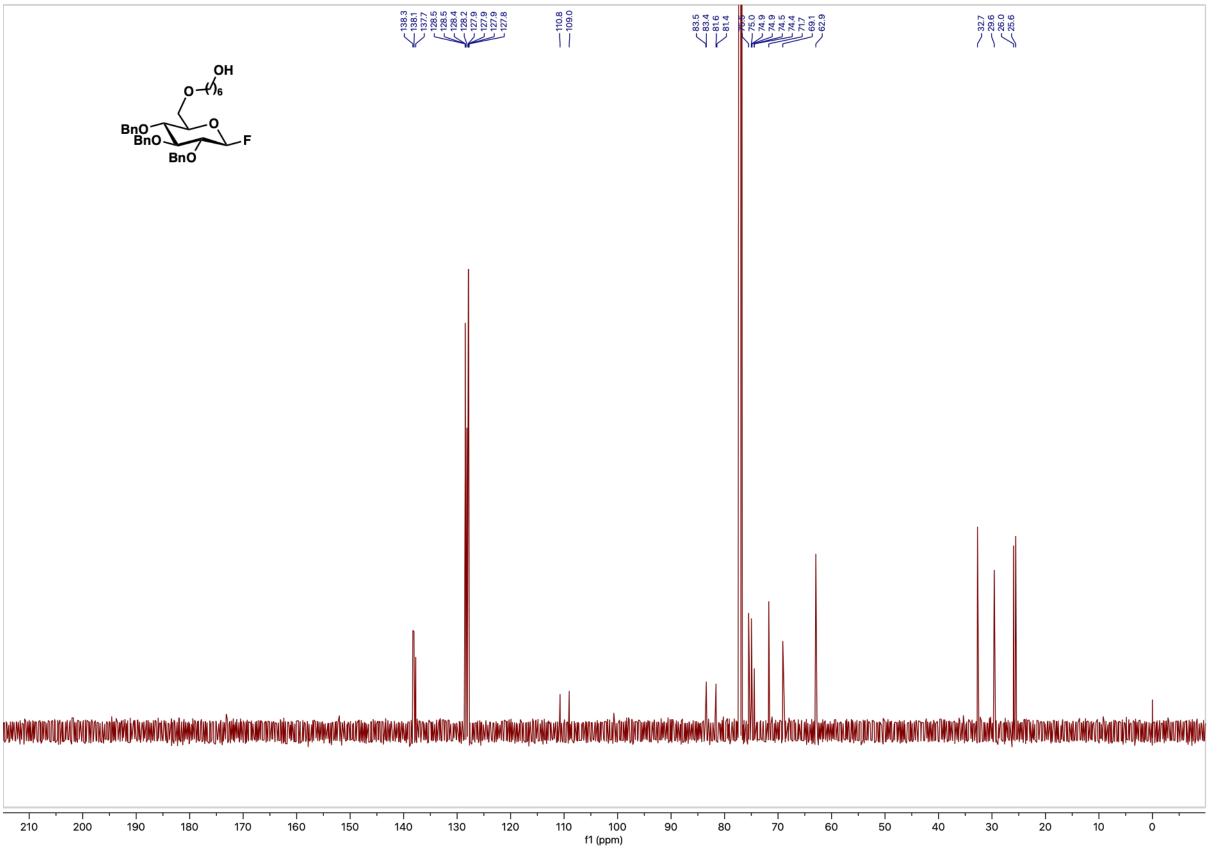

^{1}H NMR (500 MHz, $CDCl_3$, 298K) of compound **S10a**

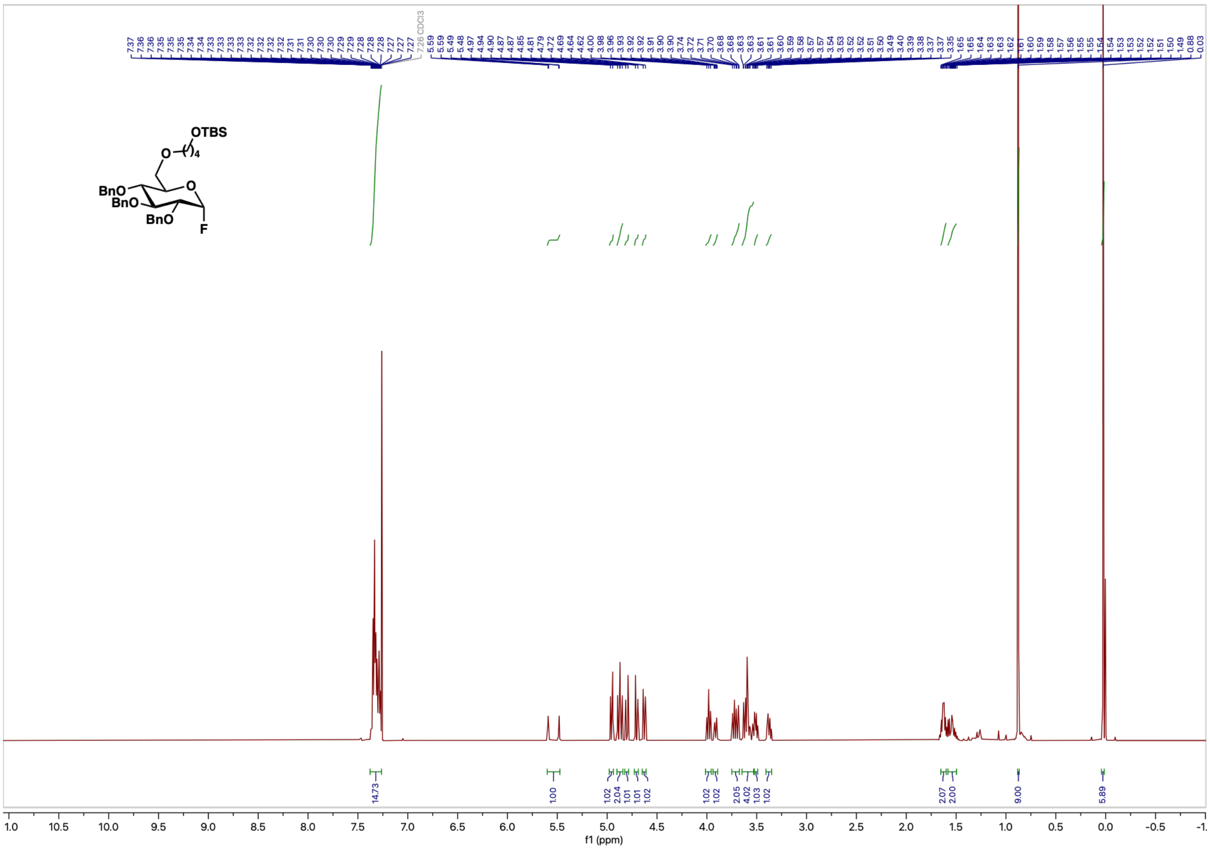

^{19}F NMR (470 MHz, $CDCl_3$, 298K) of compound **S10a**

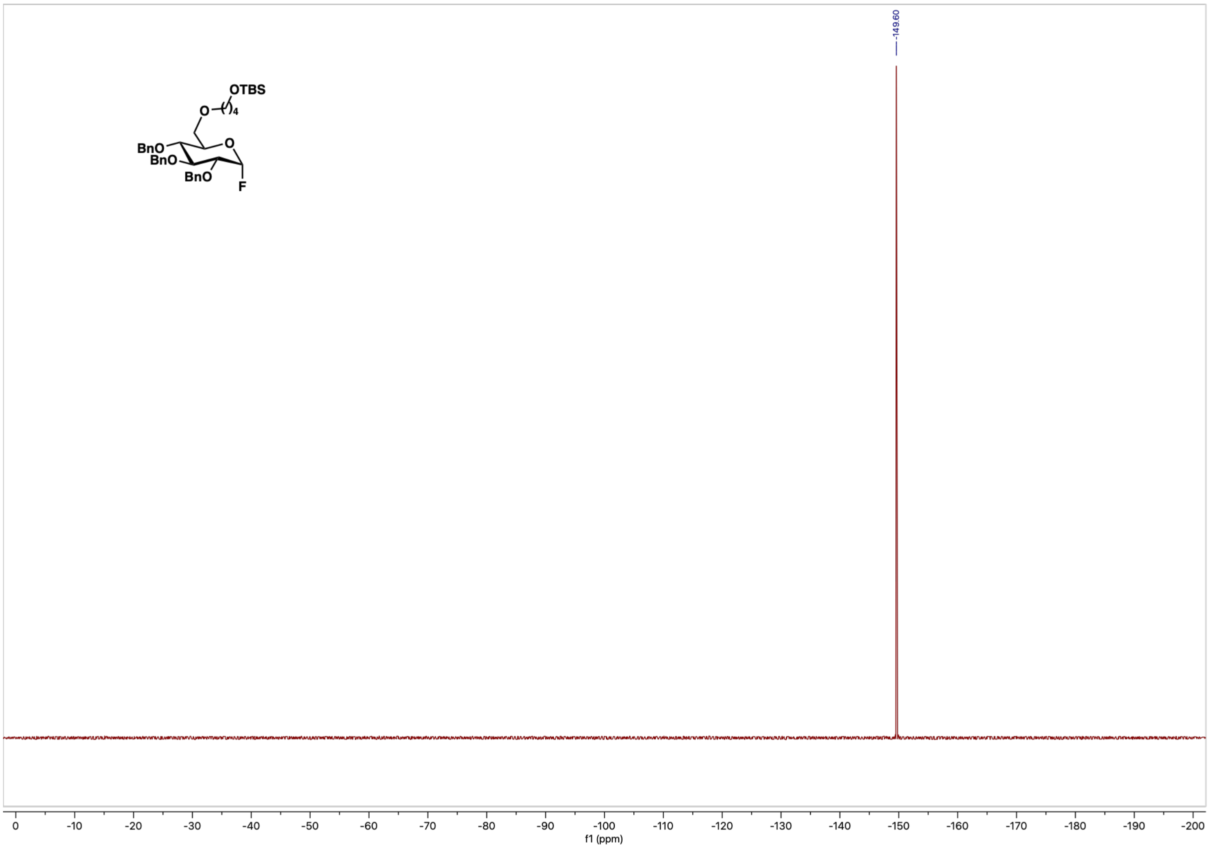

^{13}C NMR (126 MHz, $CDCl_3$, 298K) of compound **S10a**

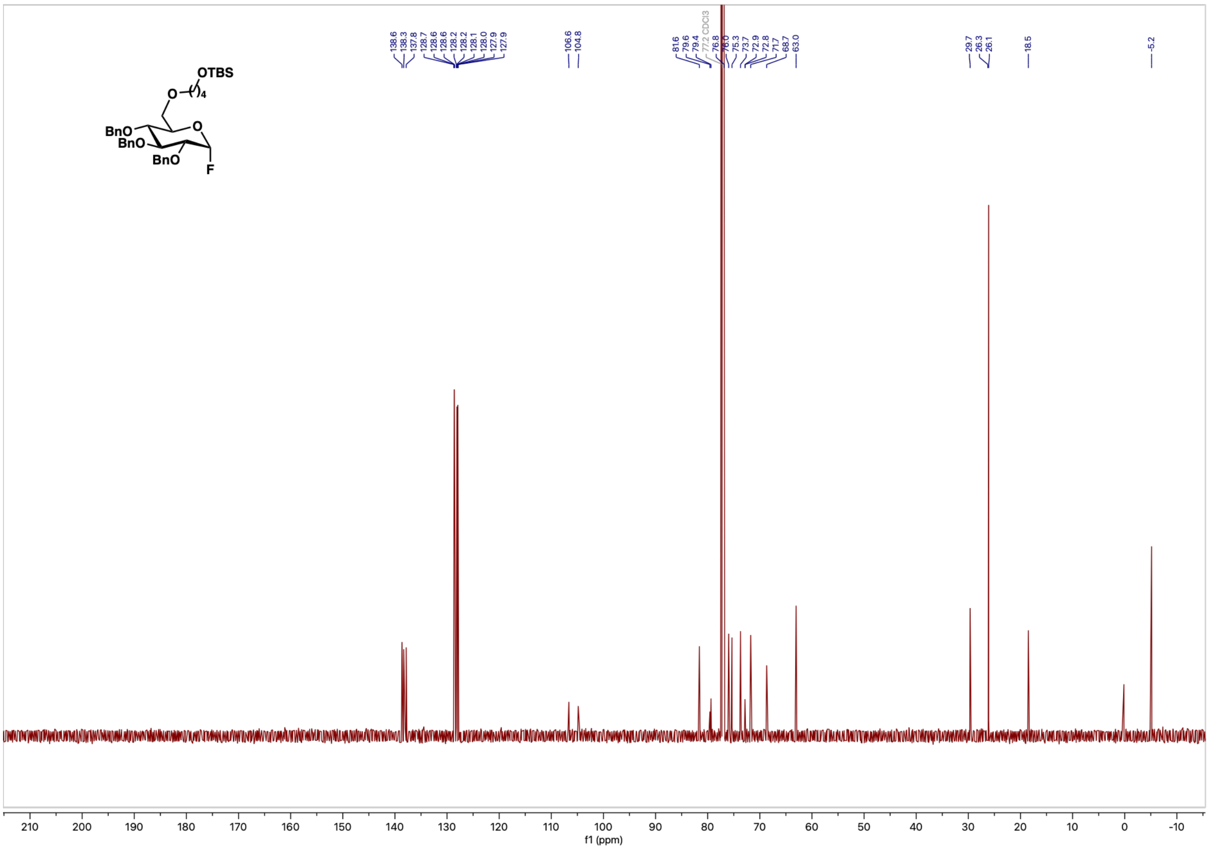

^{1}H NMR (500 MHz, $CDCl_3$, 298K) of compound **S11a**

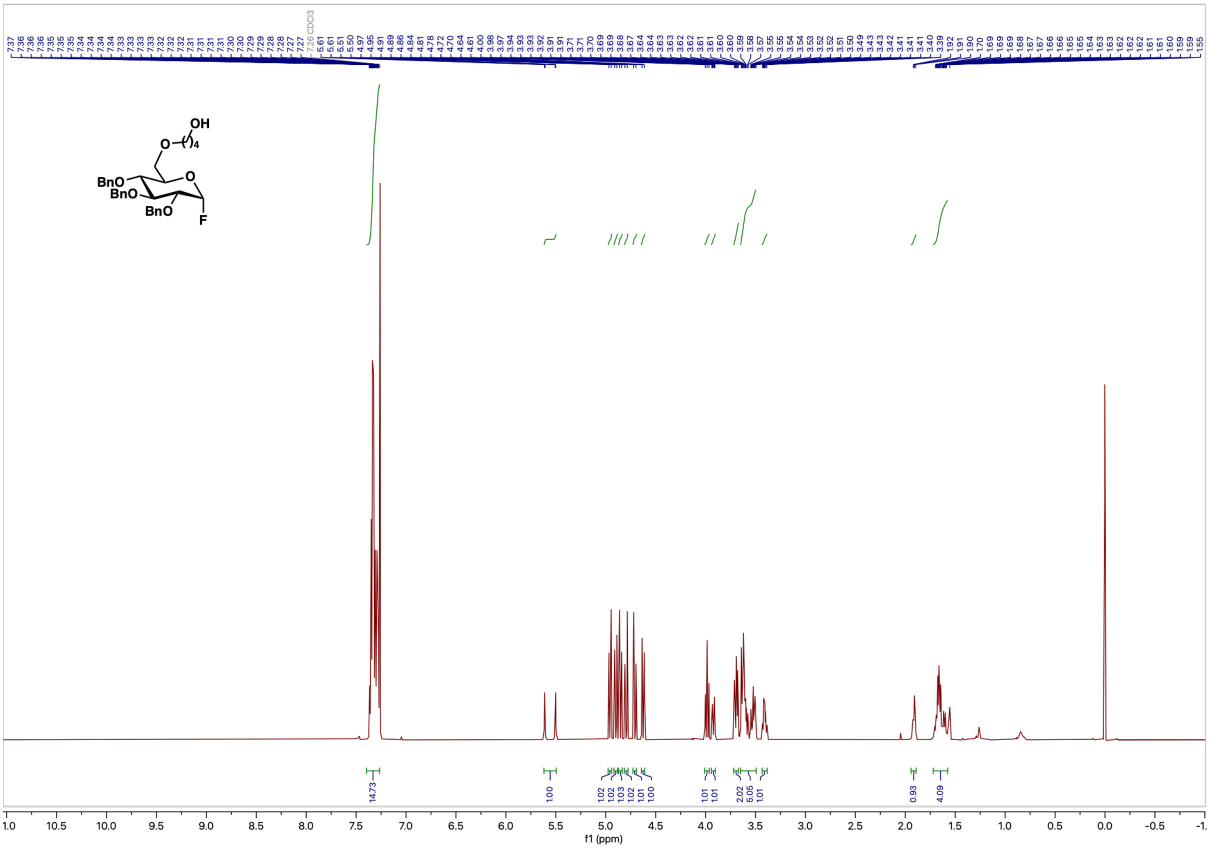

^{19}F NMR (470 MHz, $CDCl_3$, 298K) of compound **S11a**

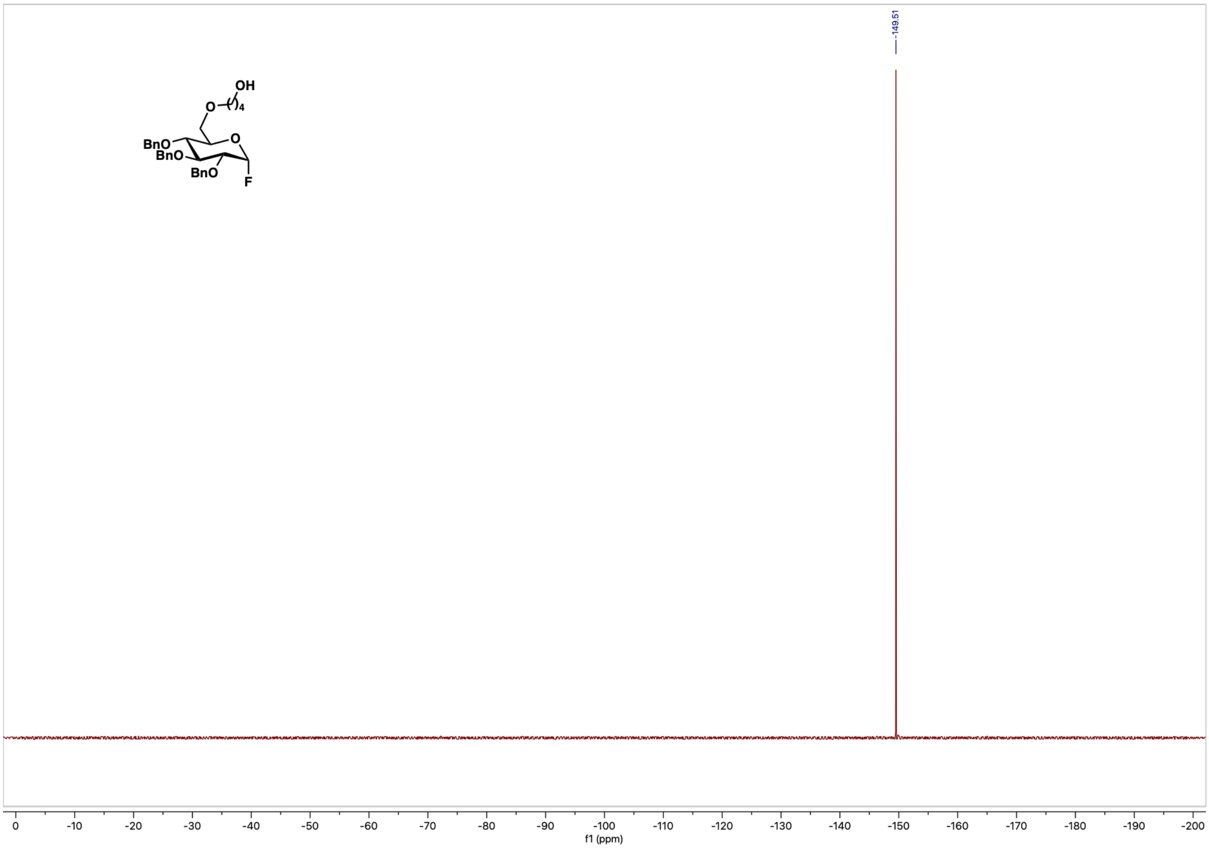

^{13}C NMR (126 MHz, $CDCl_3$, 298K) of compound **S11a**

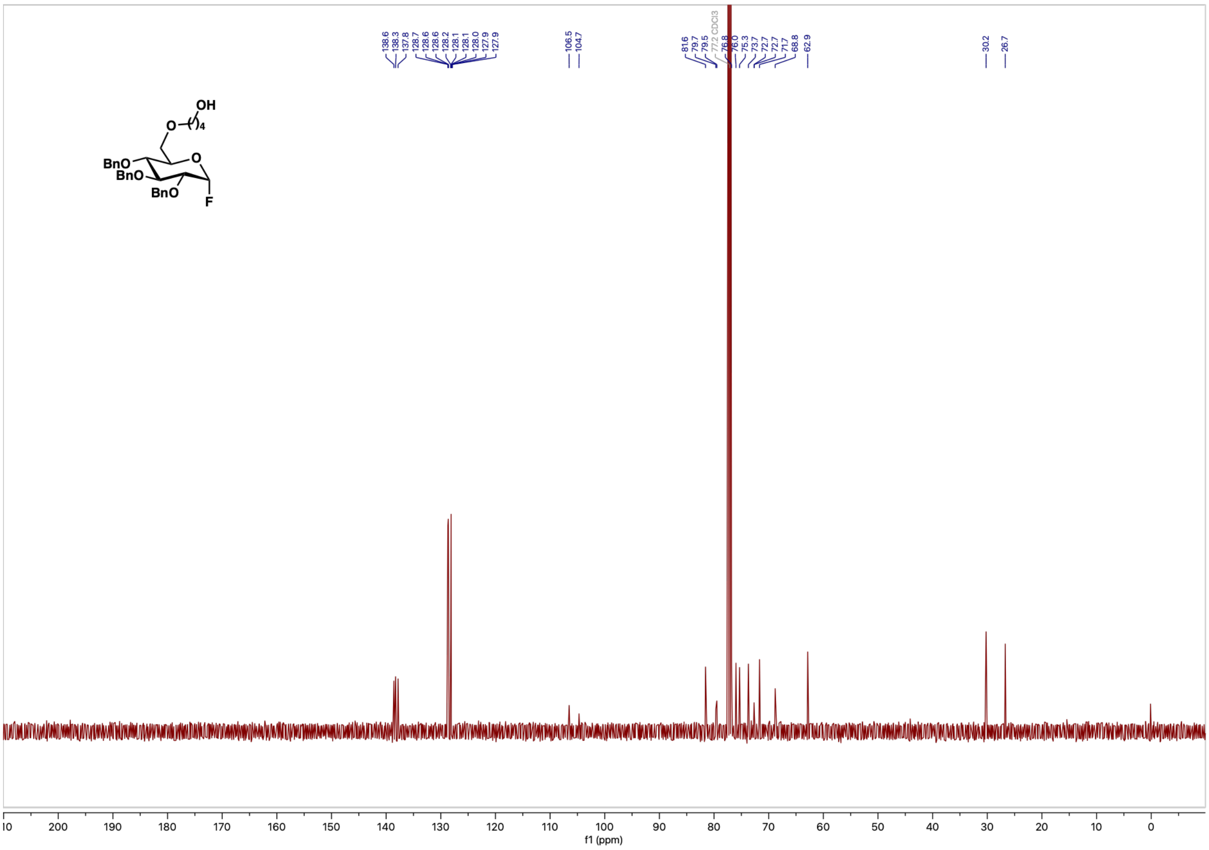

^{1}H NMR (500 MHz, $CDCl_3$, 298K) of compound **S10b**

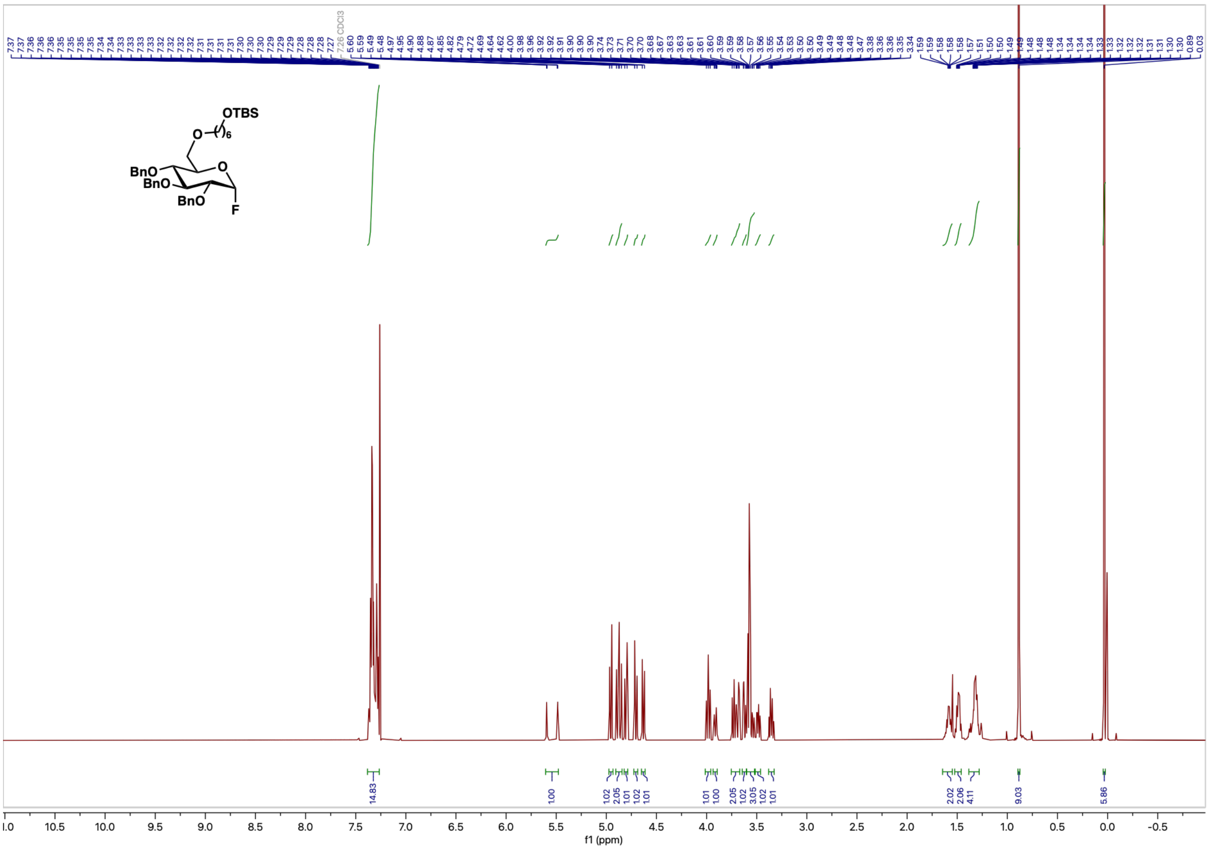

^{19}F NMR (470 MHz, $CDCl_3$, 298K) of compound **S10b**

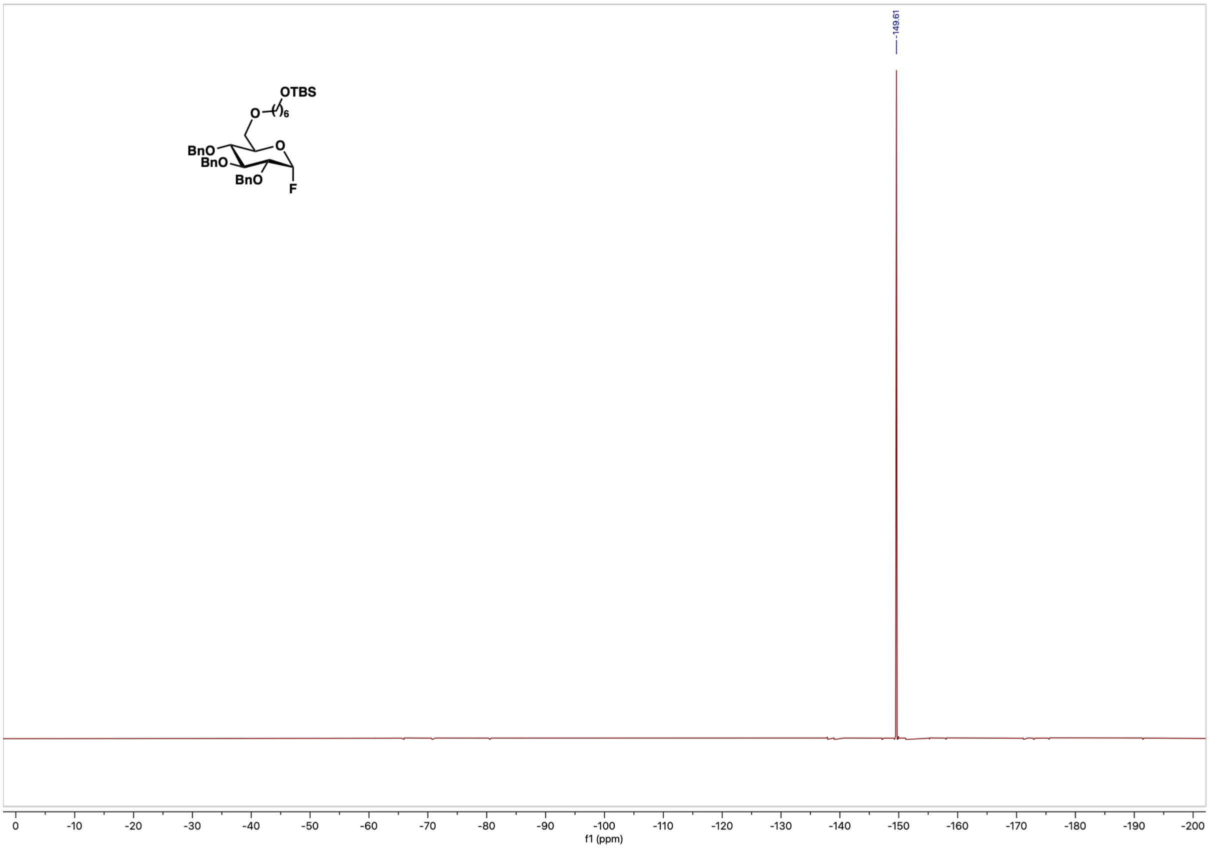

^{13}C NMR (126 MHz, $CDCl_3$, 298K) of compound **S10b**

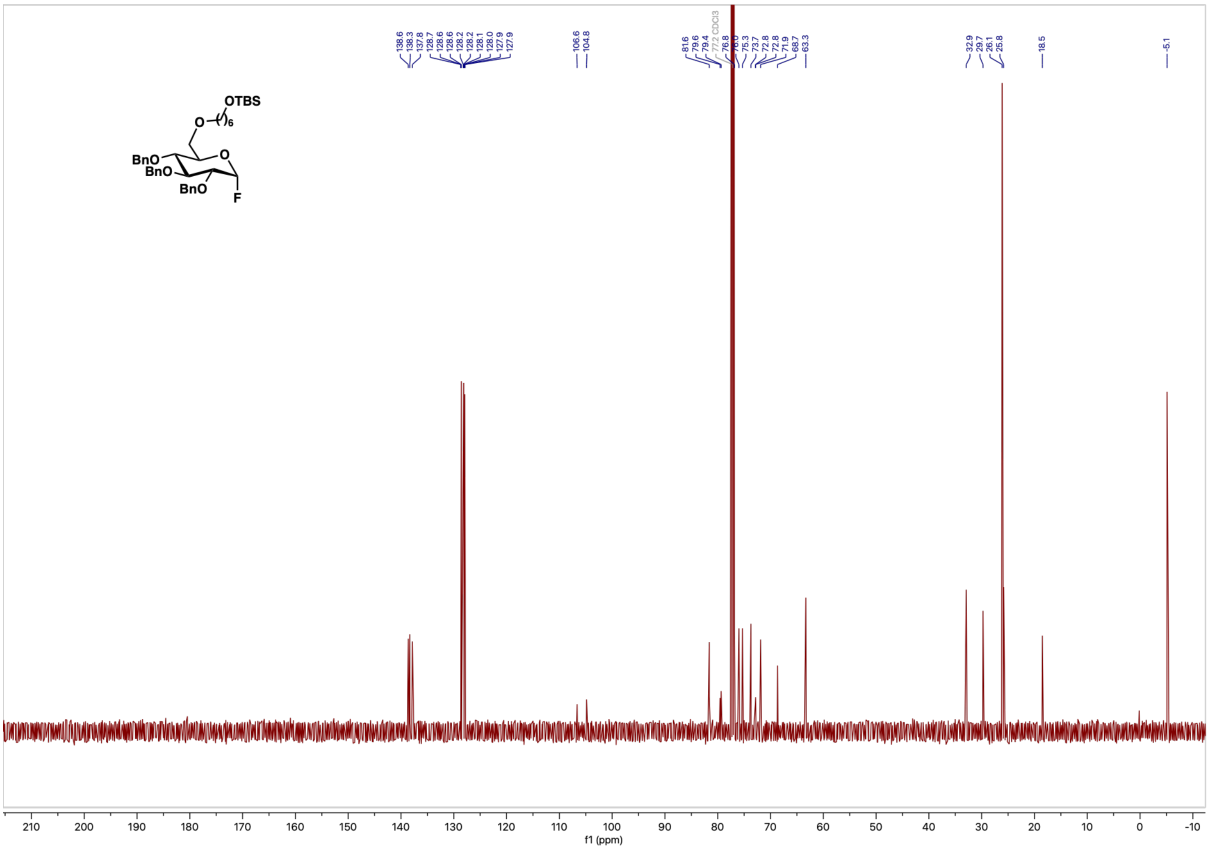

^{1}H NMR (500 MHz, $CDCl_3$, 298K) of compound **S11b**

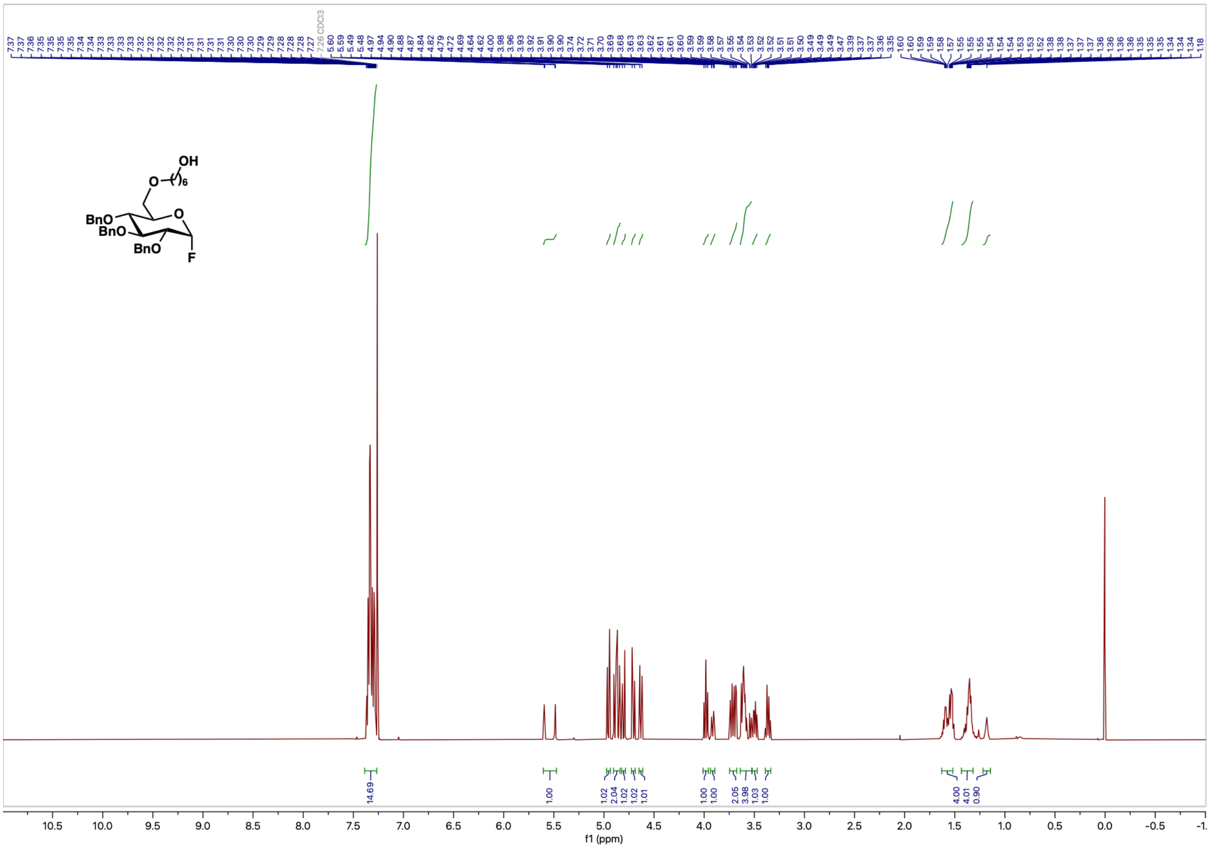

^{19}F NMR (470 MHz, $CDCl_3$, 298K) of compound **S11b**

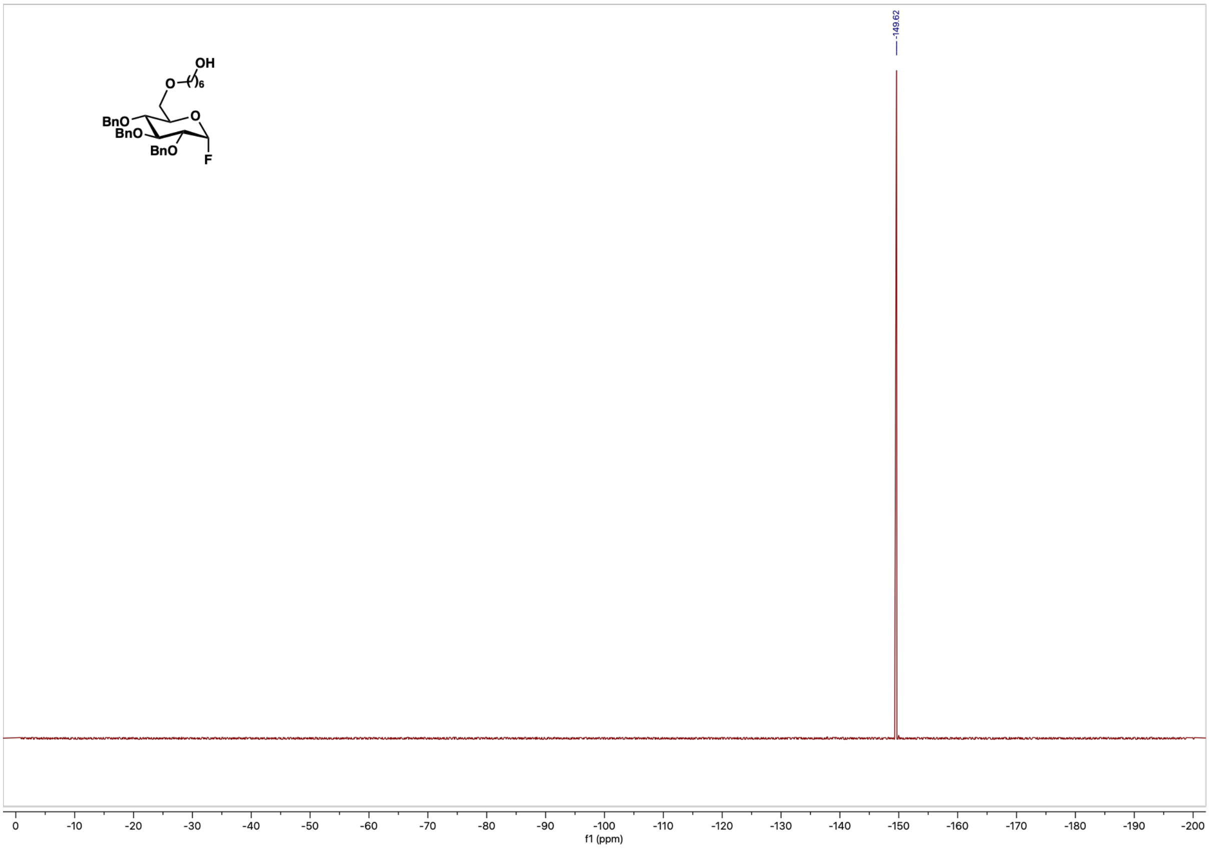

^{13}C NMR (126 MHz, $CDCl_3$, 298K) of compound **S11b**

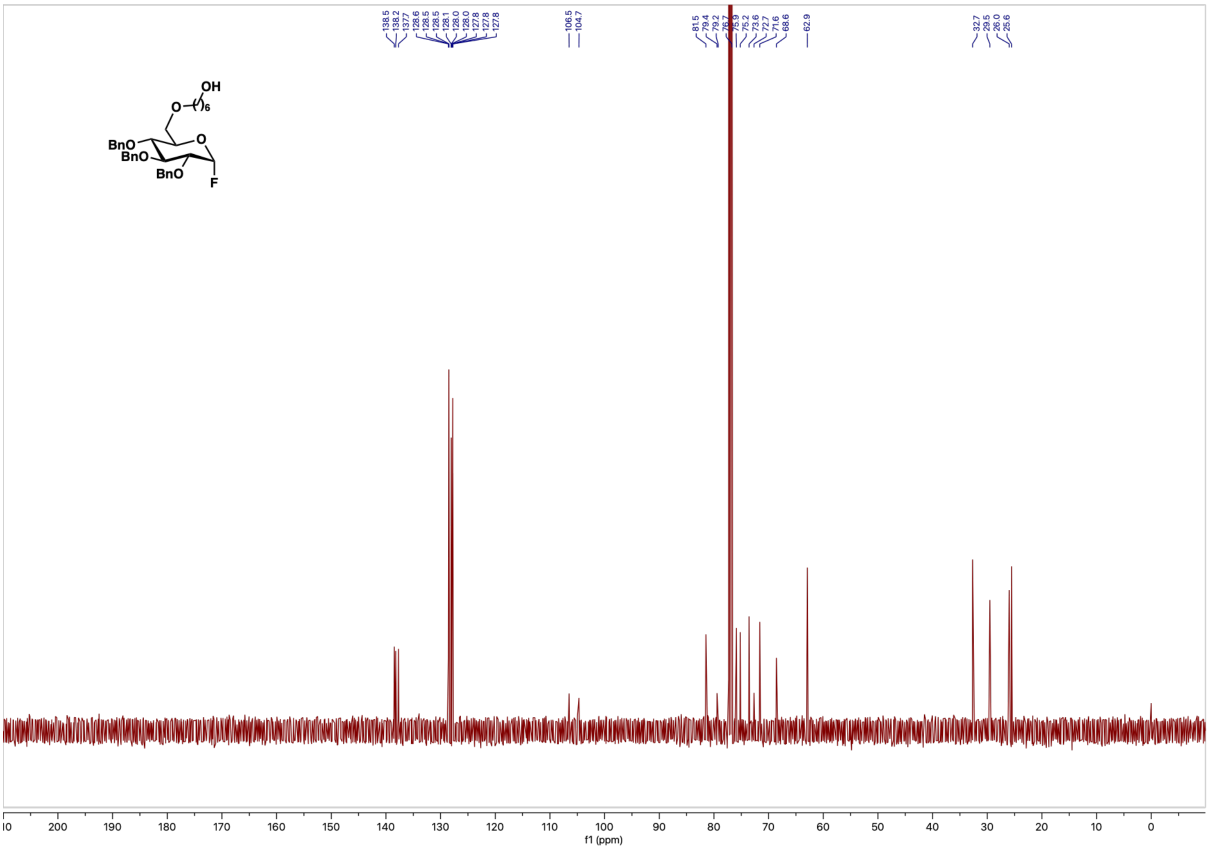

^{1}H NMR (500 MHz, $CDCl_3$, 298K) of compound **S12**

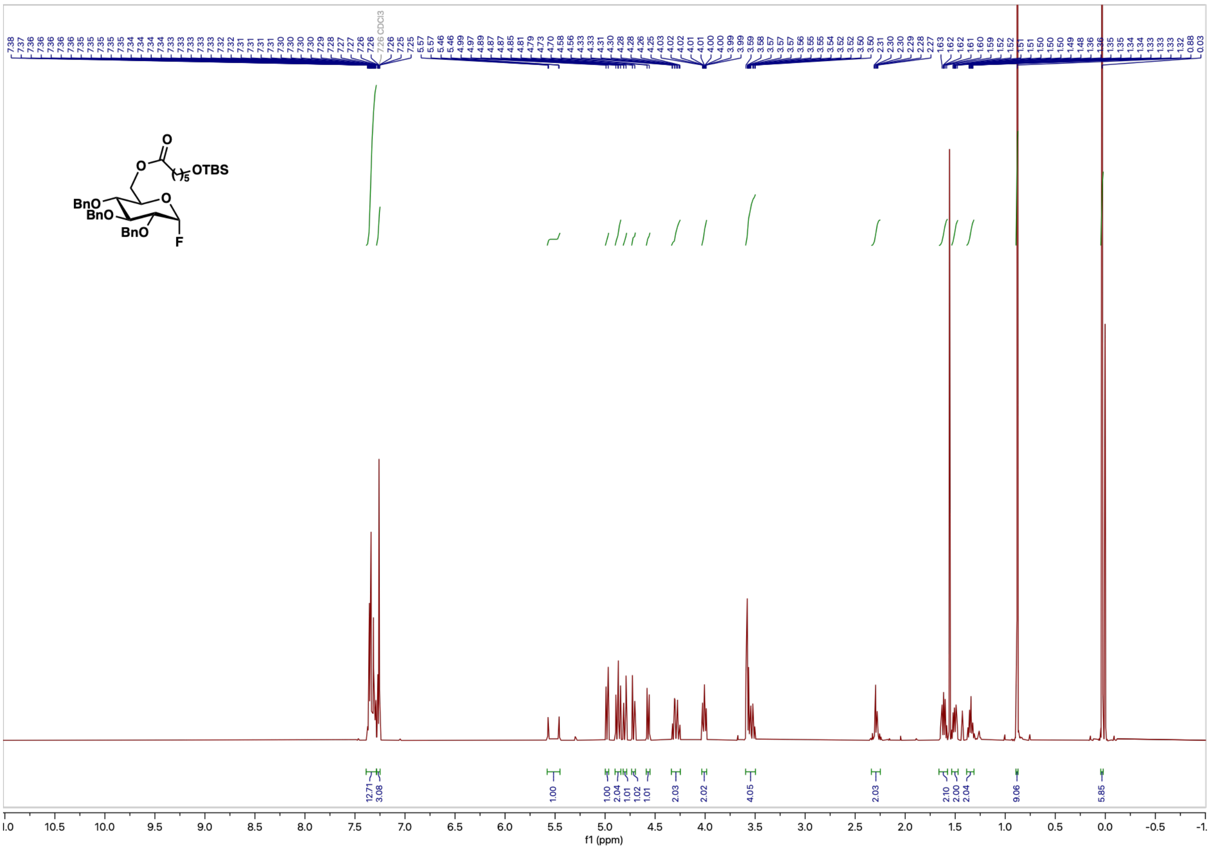

^{19}F NMR (470 MHz, $CDCl_3$, 298K) of compound **S12**

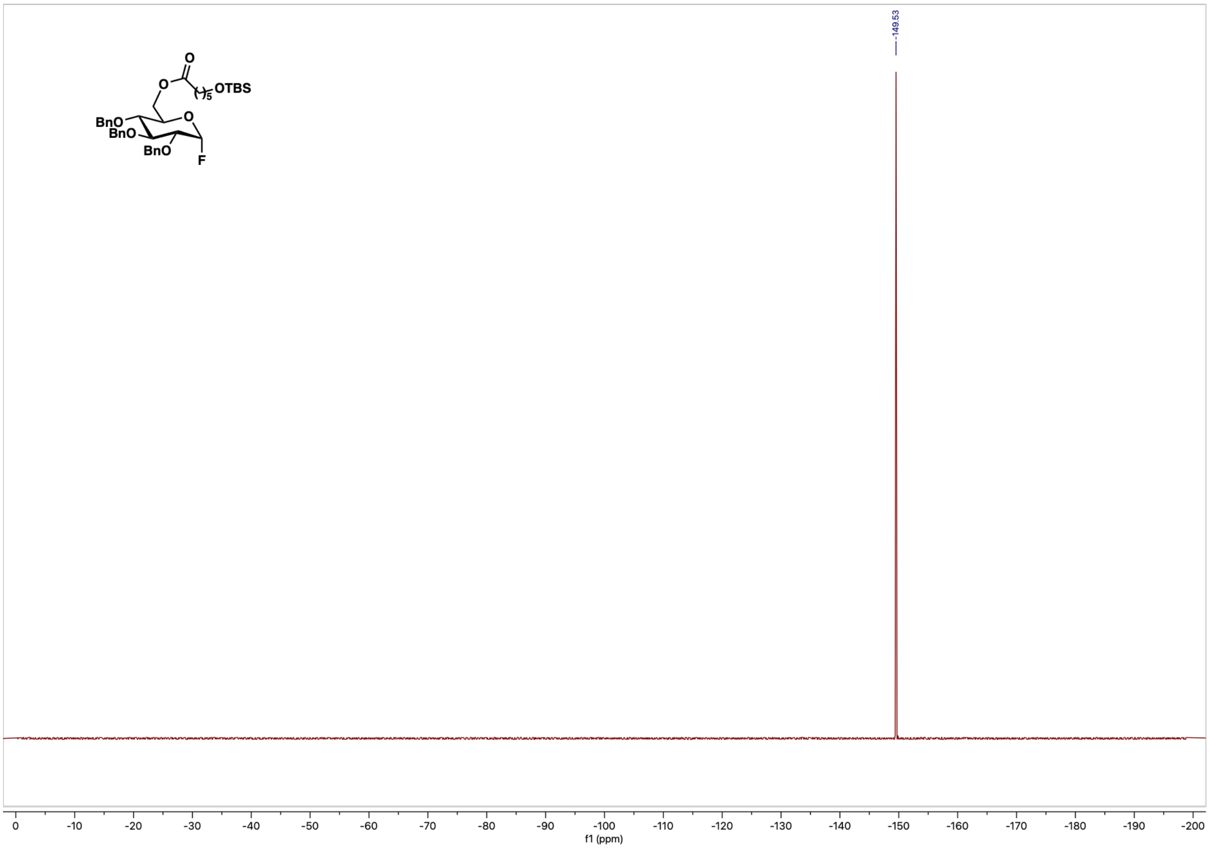

^{13}C NMR (126 MHz, $CDCl_3$, 298K) of compound **S12**

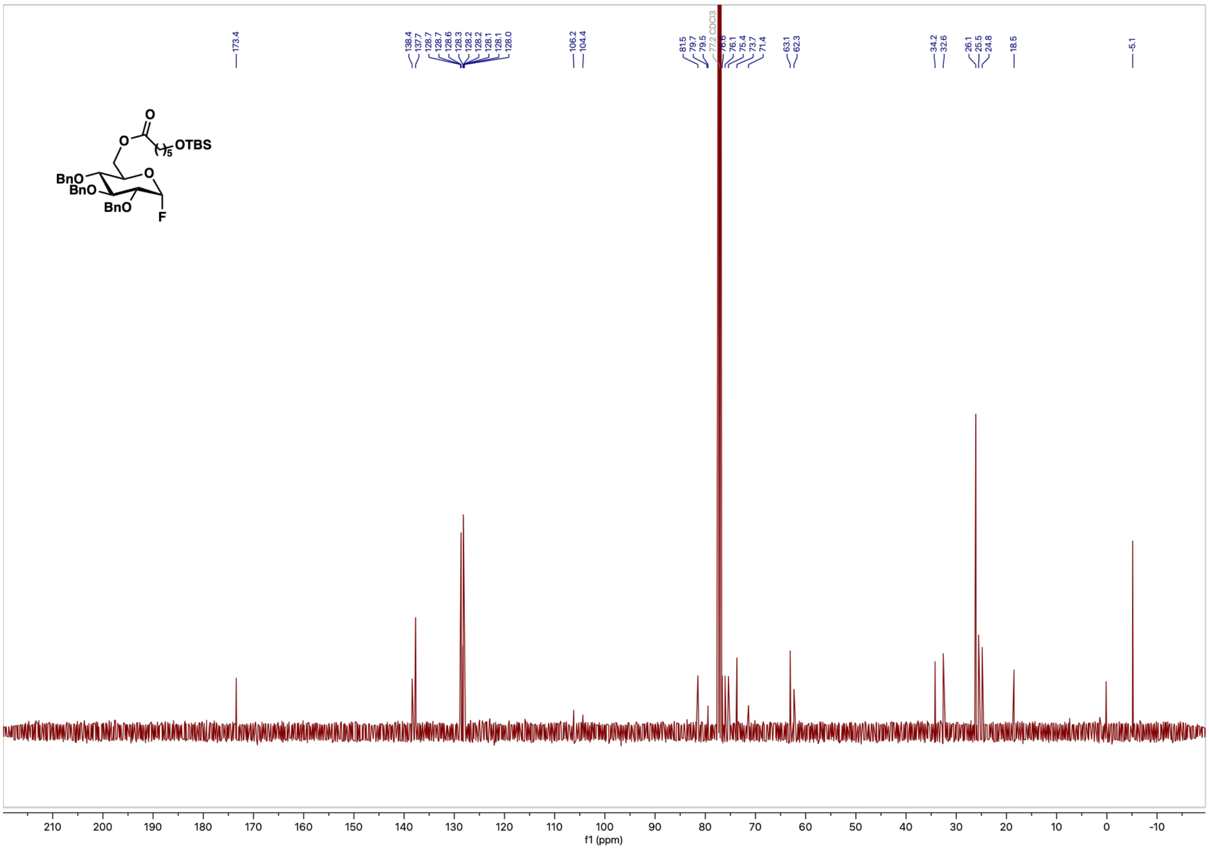

^{1}H NMR (500 MHz, $CDCl_3$, 298K) of compound **S13**

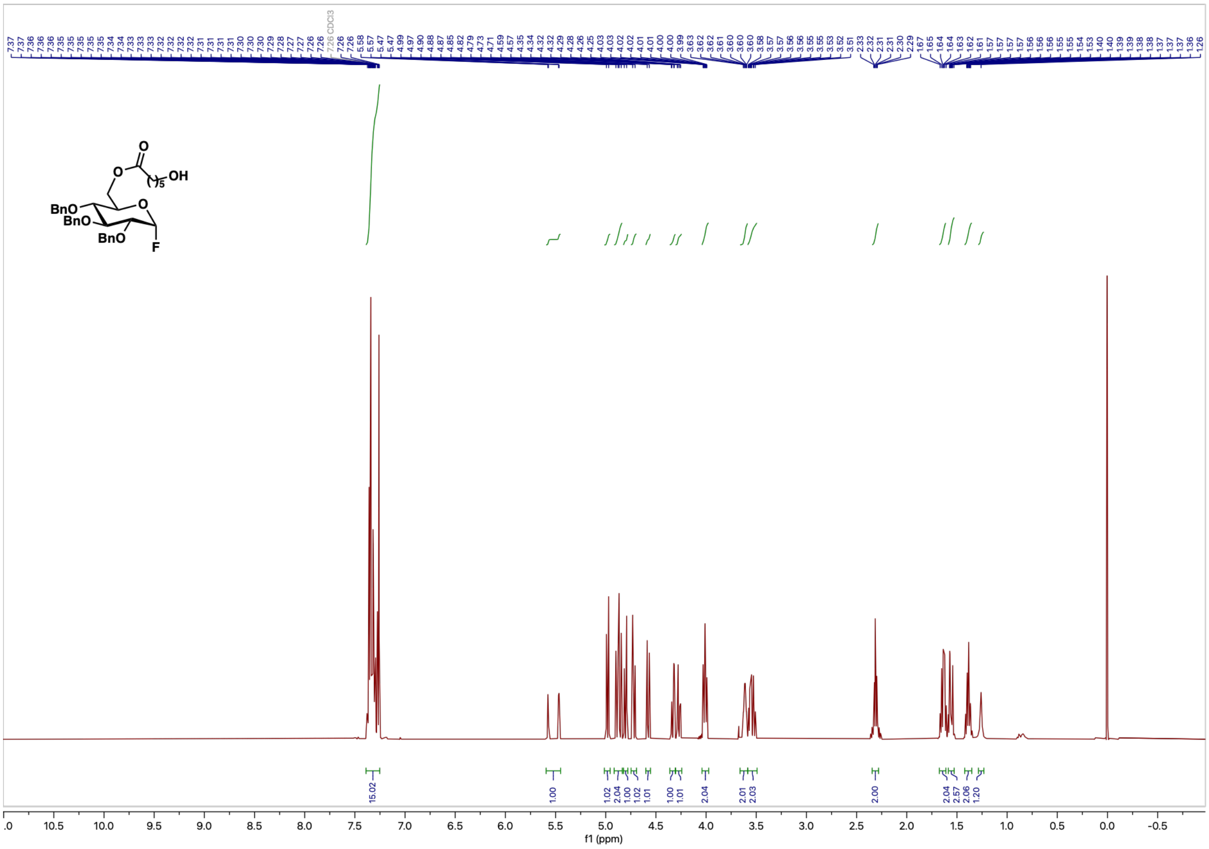

^{19}F NMR (470 MHz, $CDCl_3$, 298K) of compound **S13**

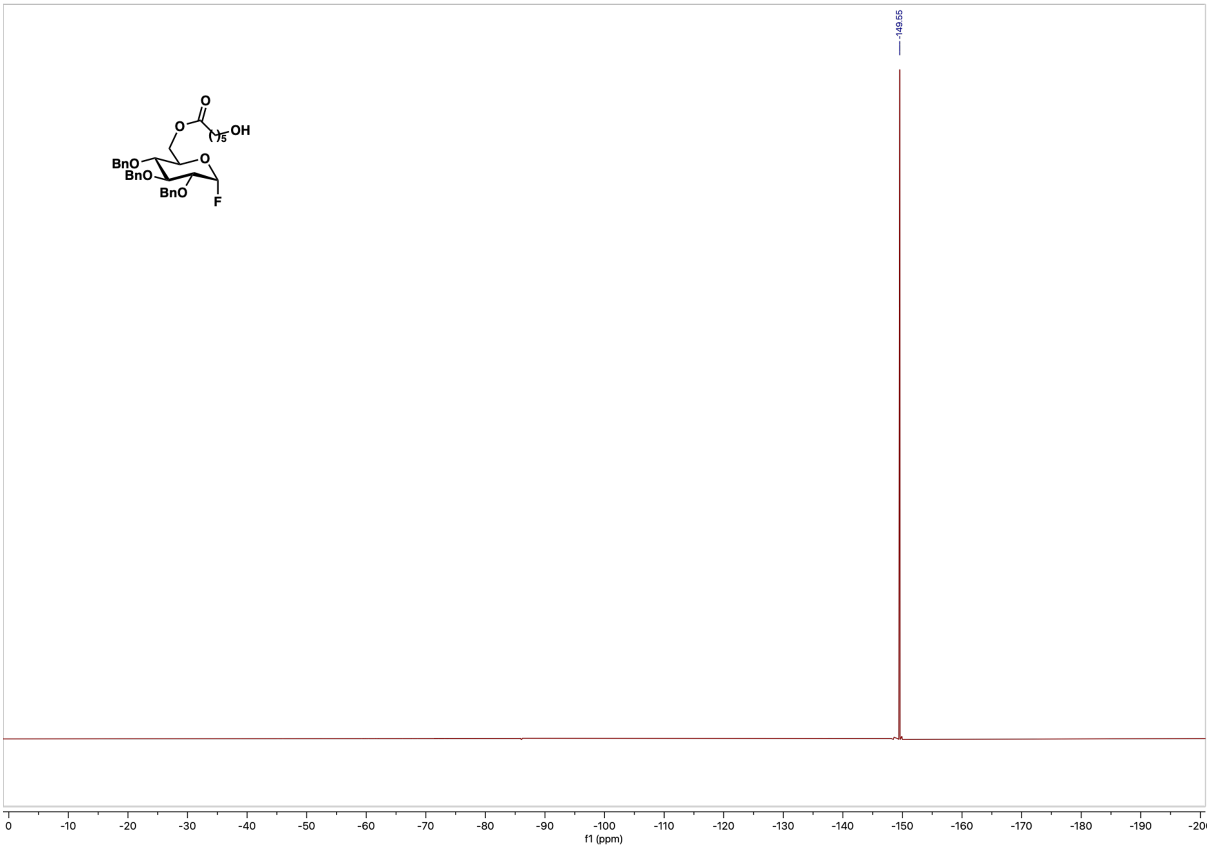

^{13}C NMR (126 MHz, $CDCl_3$, 298K) of compound **S13**

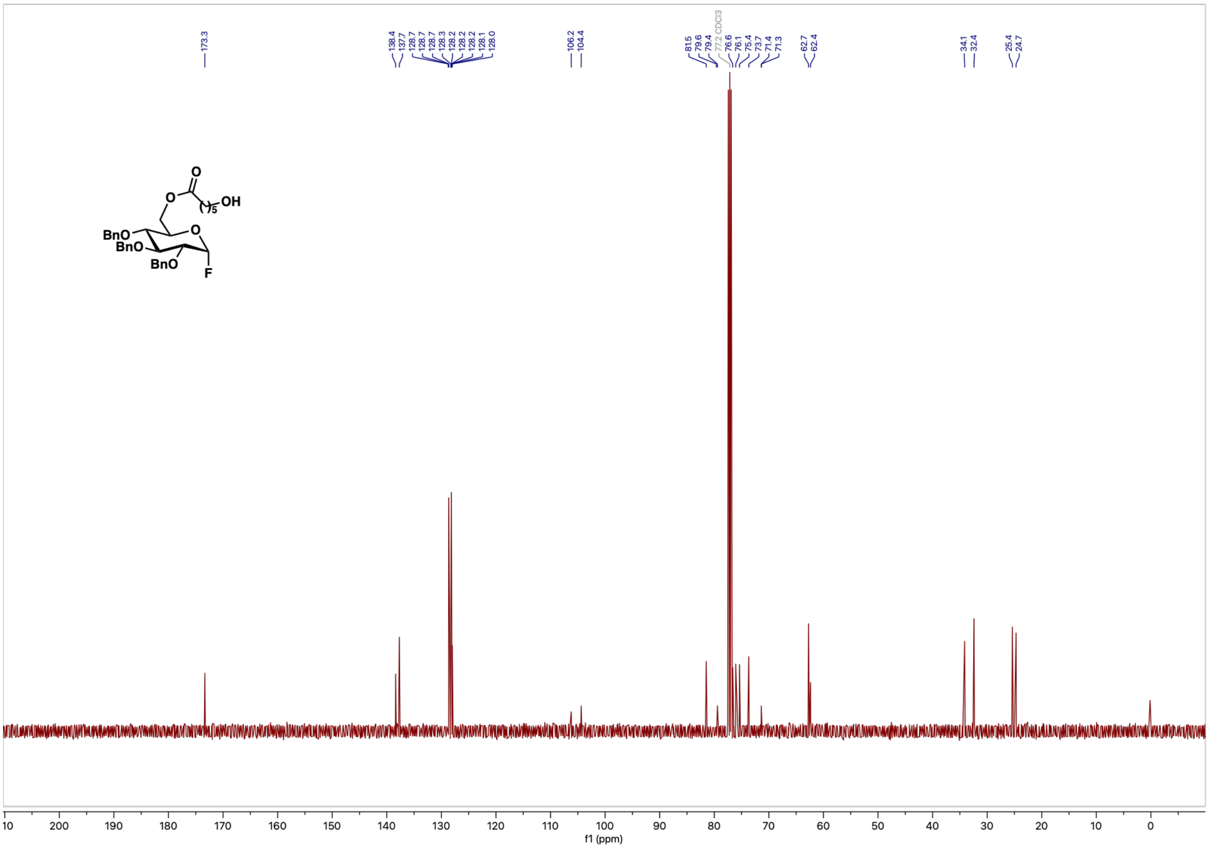

^{1}H NMR (500 MHz, CD_3OD, 298K) of compound **S16**

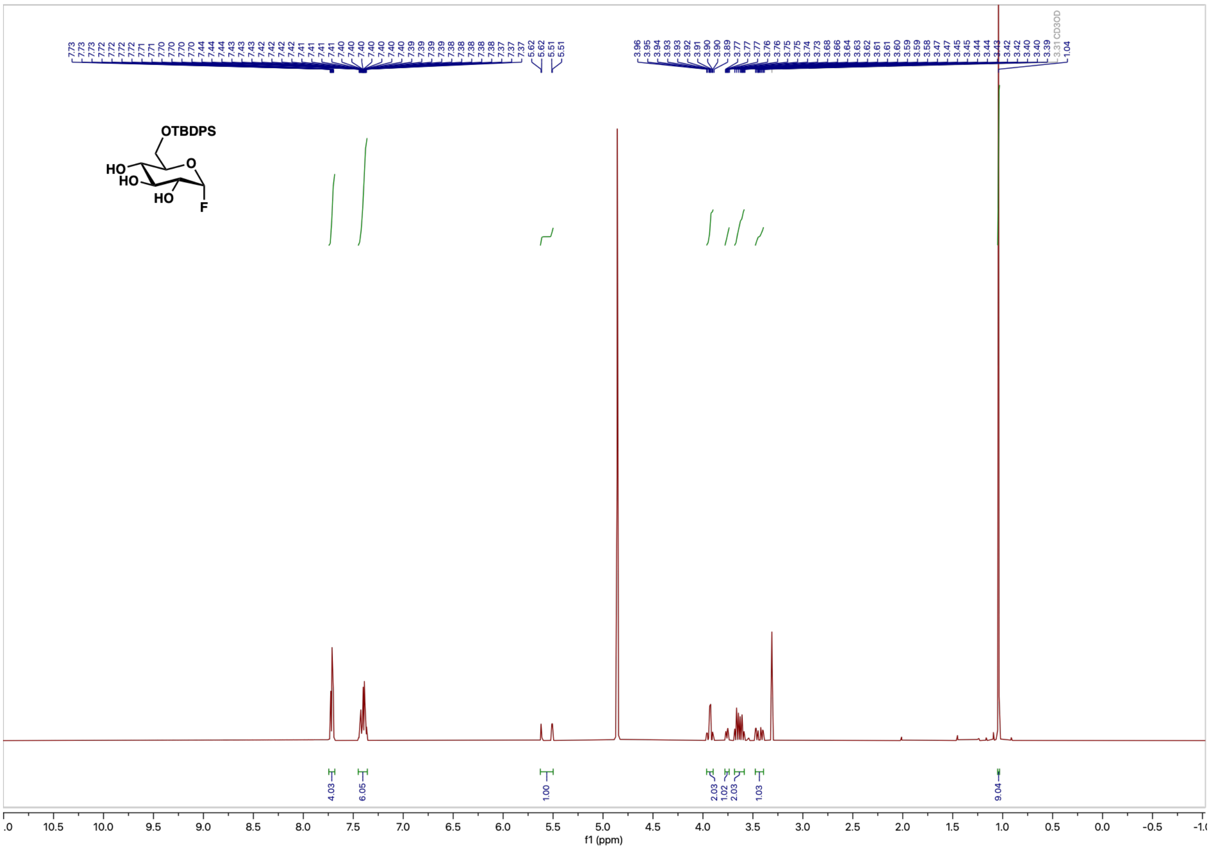

^{19}F NMR (470 MHz, CD_3OD, 298K) of compound **S16**

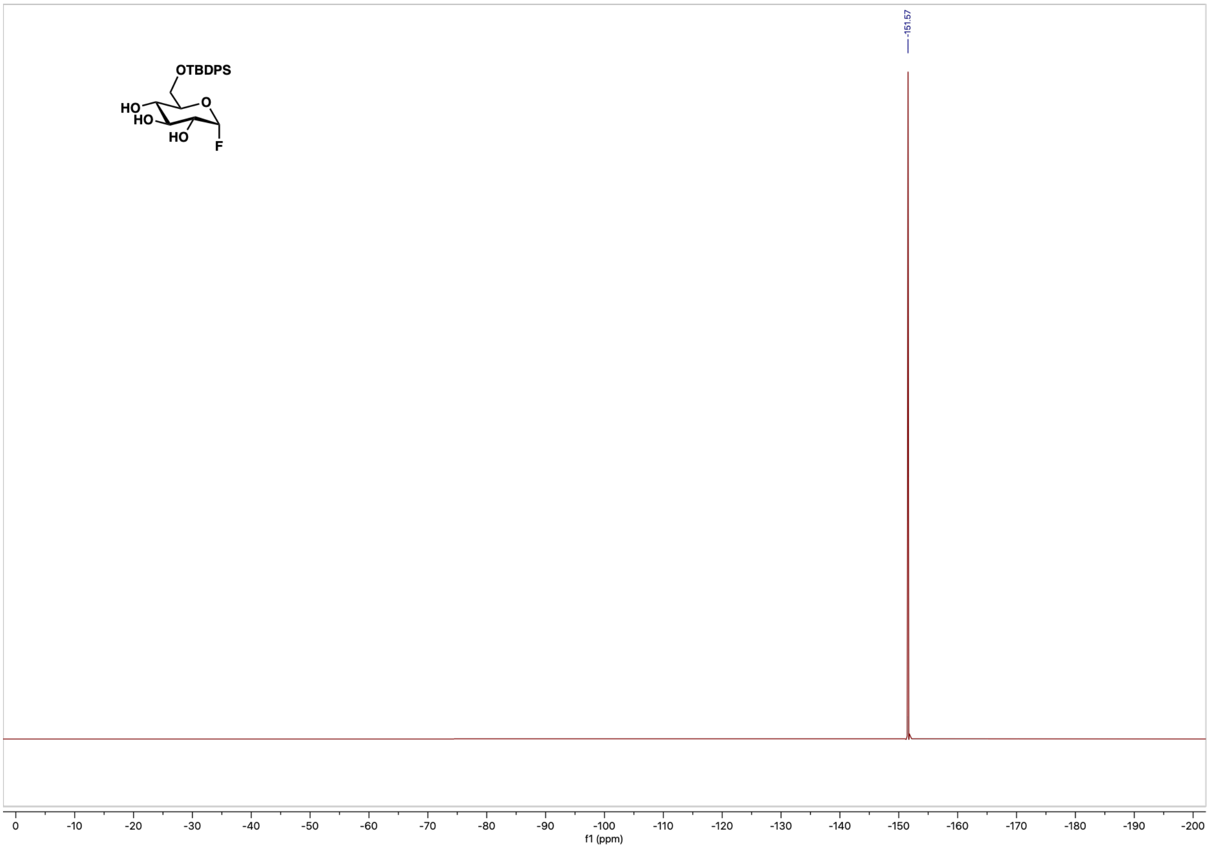

^{13}C NMR (126 MHz, CD_3OD, 298K) of compound **S16**

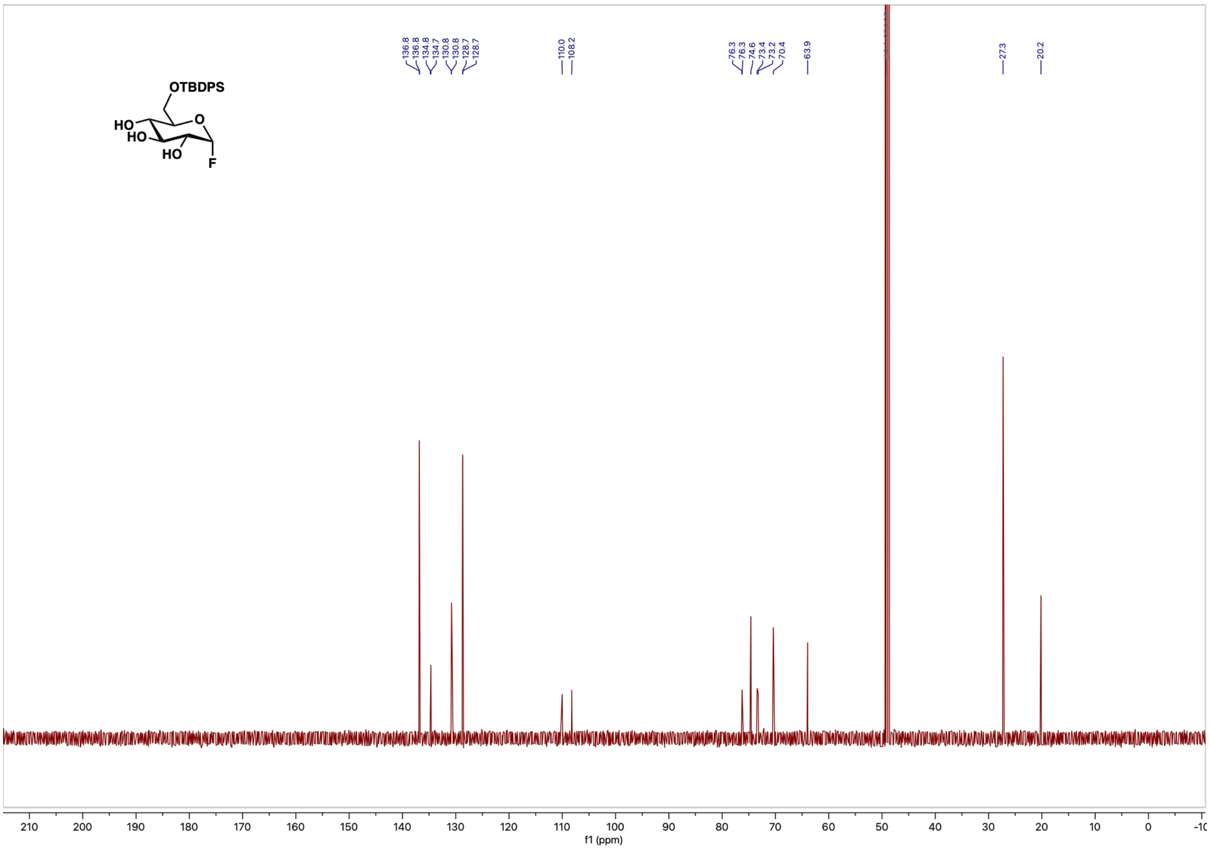

^{1}H NMR (500 MHz, $CDCl_3$, 298K) of compound **S17**

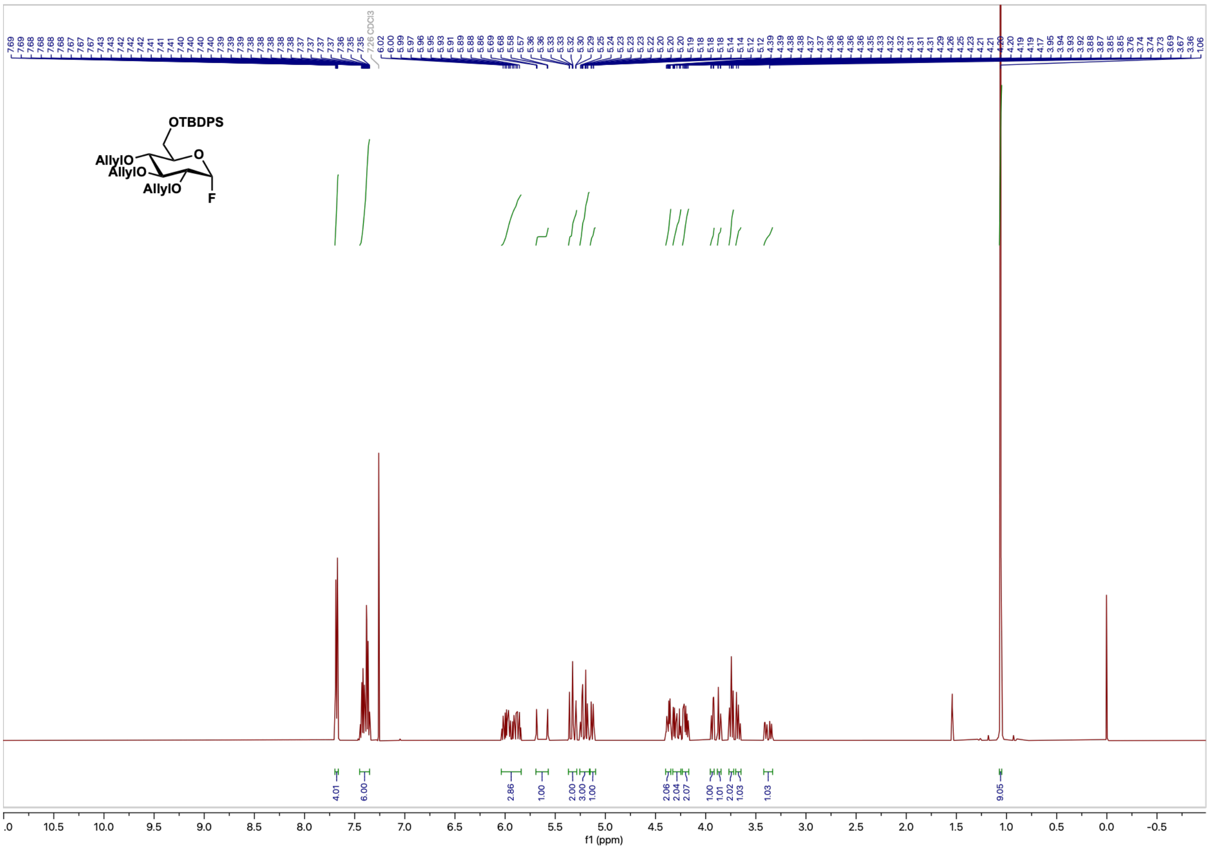

^{19}F NMR (470 MHz, $CDCl_3$, 298K) of compound **S17**

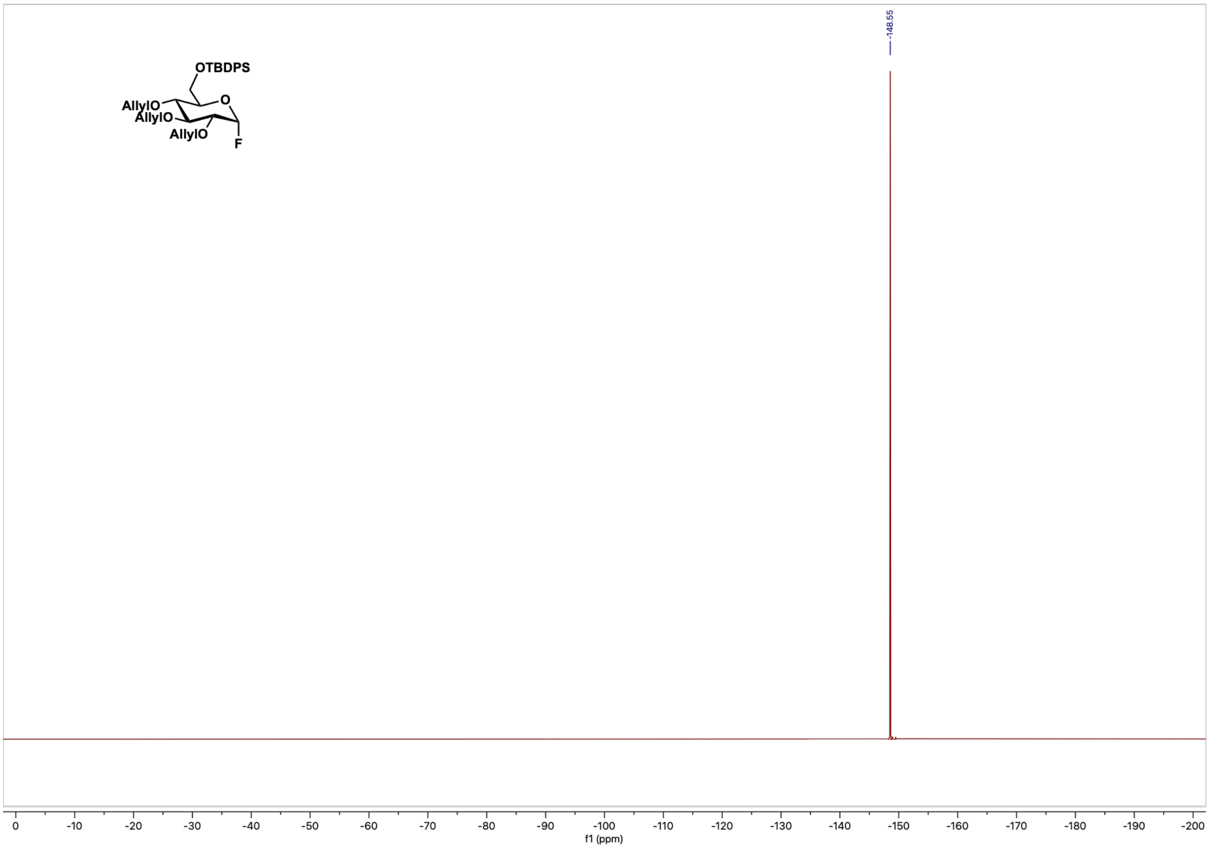

^{13}C NMR (126 MHz, $CDCl_3$, 298K) of compound **S17**

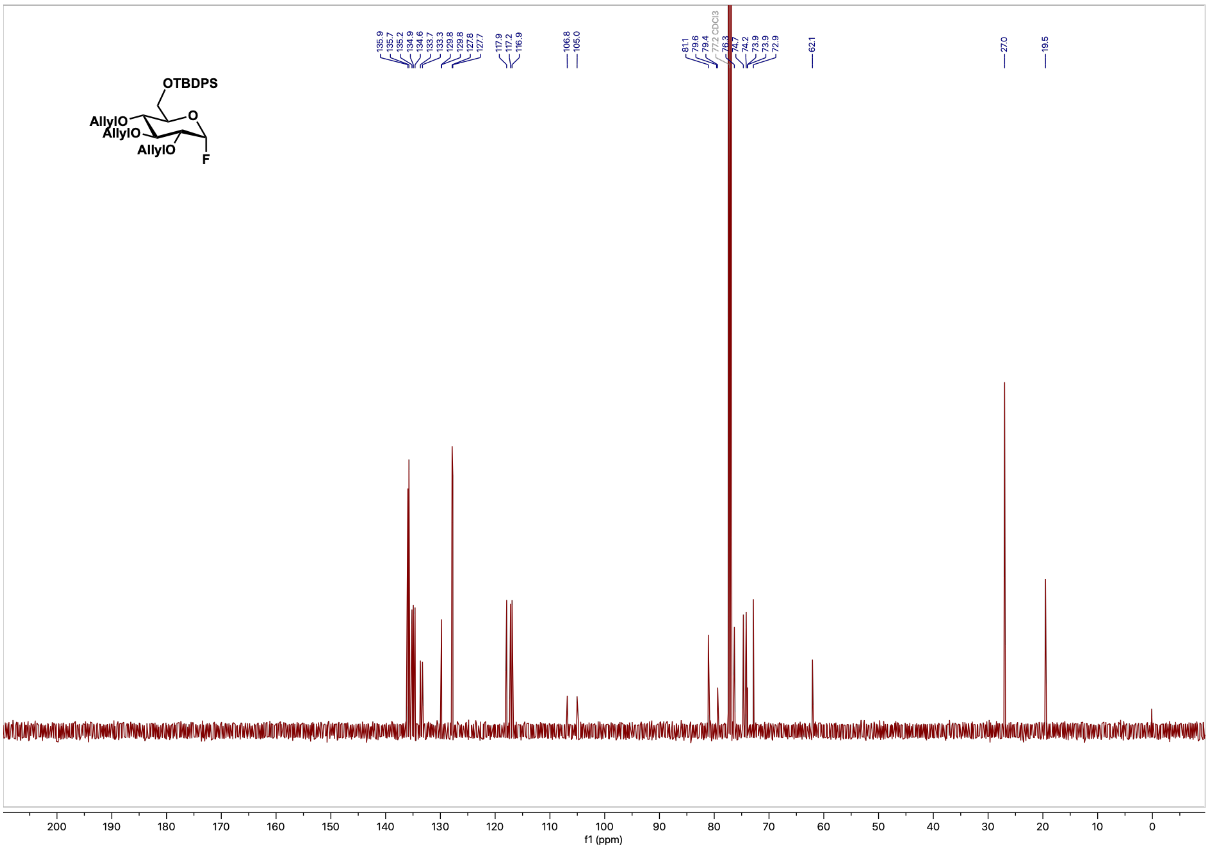

^1H NMR (500 MHz, $CDCl_3$, 298K) of compound **S18**

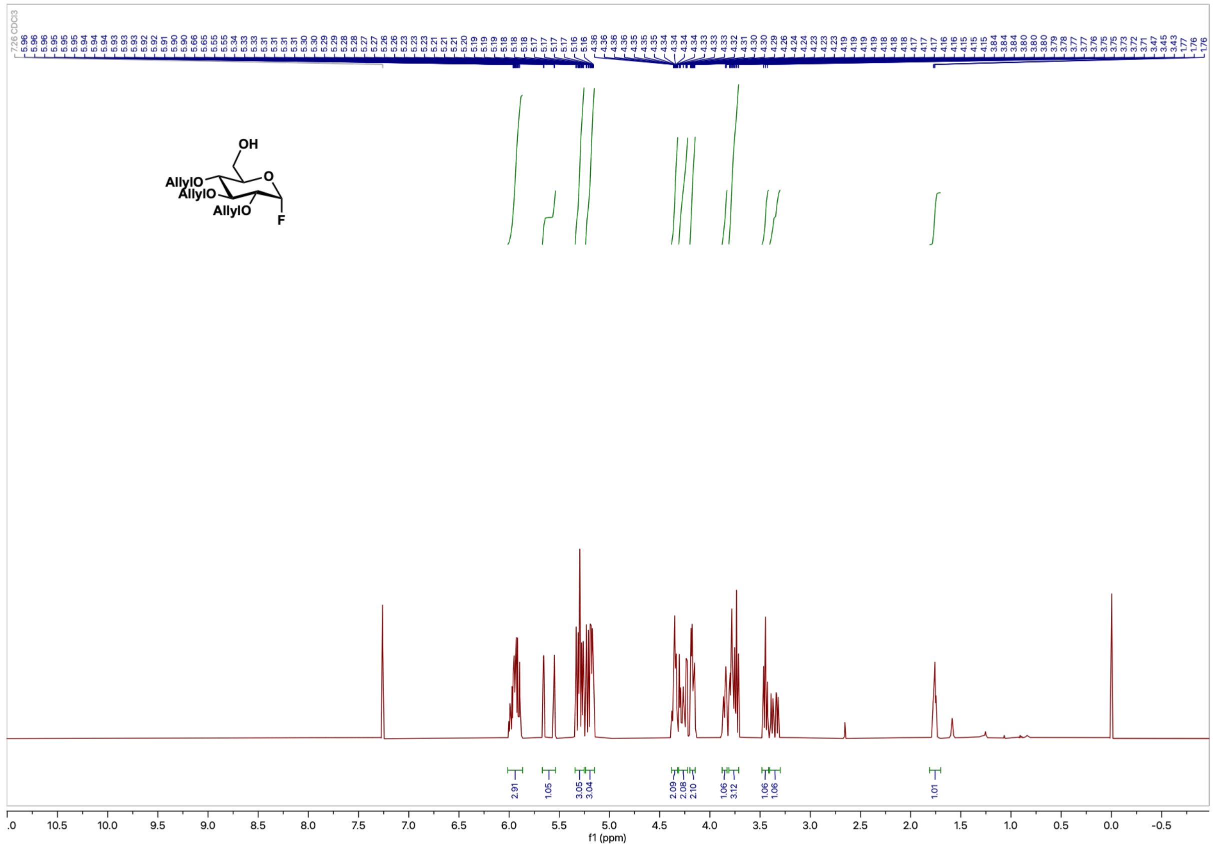

^{19}F NMR (470 MHz, $CDCl_3$, 298K) of compound **S18**

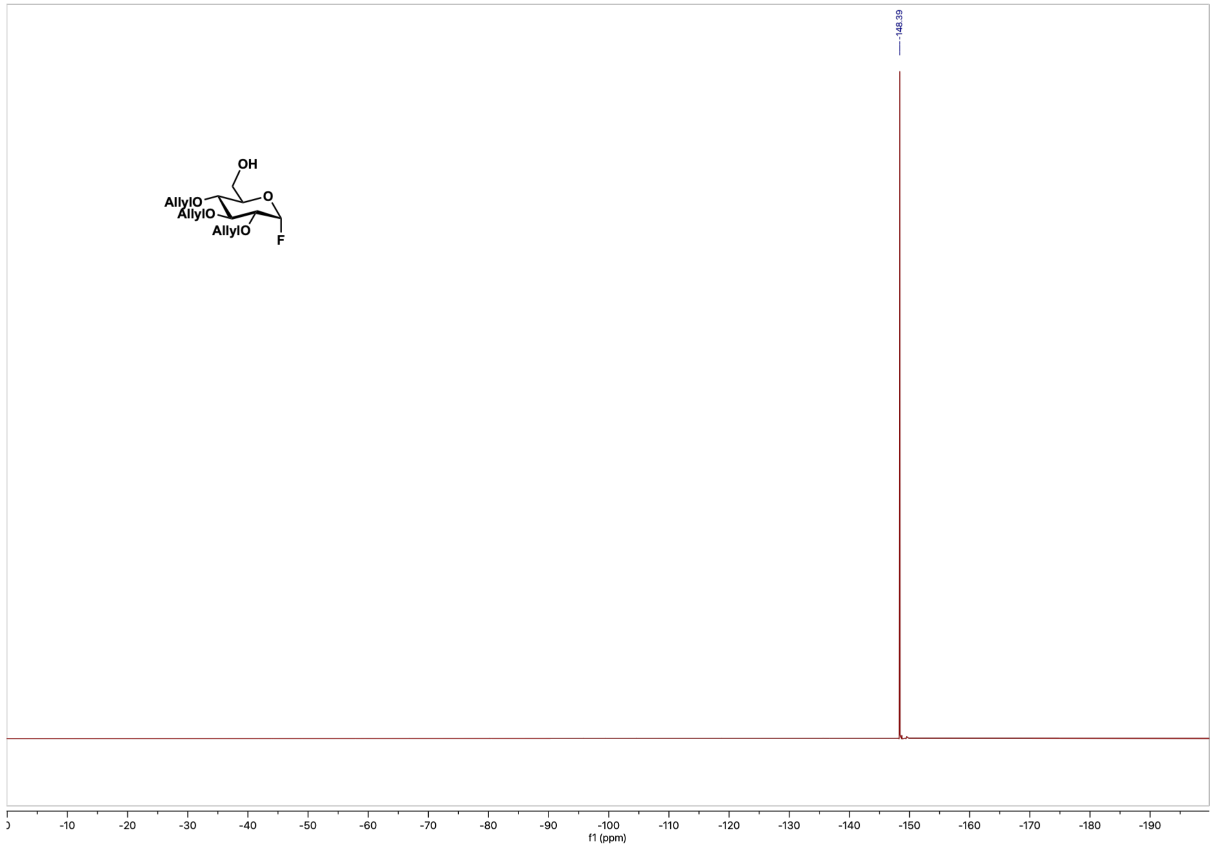

^{13}C NMR (126 MHz, $CDCl_3$, 298K) of compound **S18**

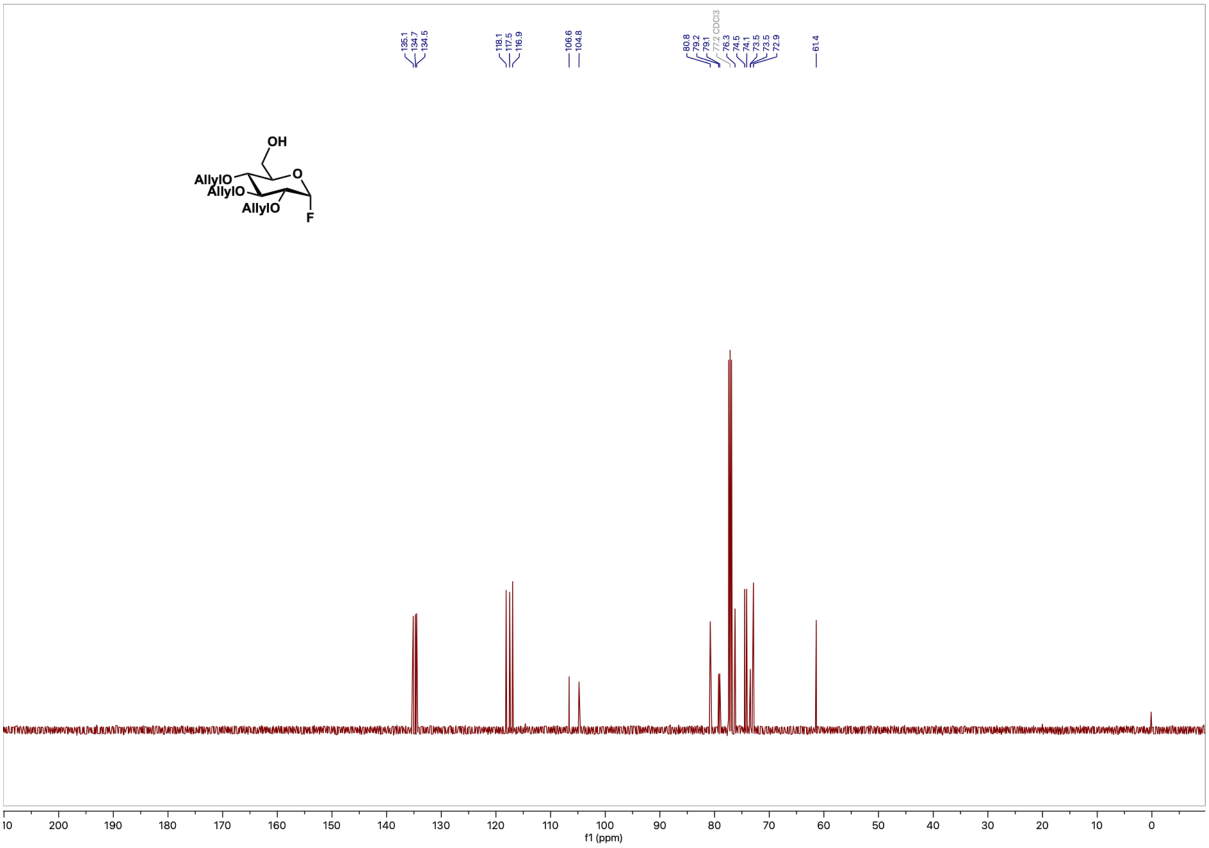

^{1}H NMR (500 MHz, $CDCl_3$, 298K) of compound **S19**

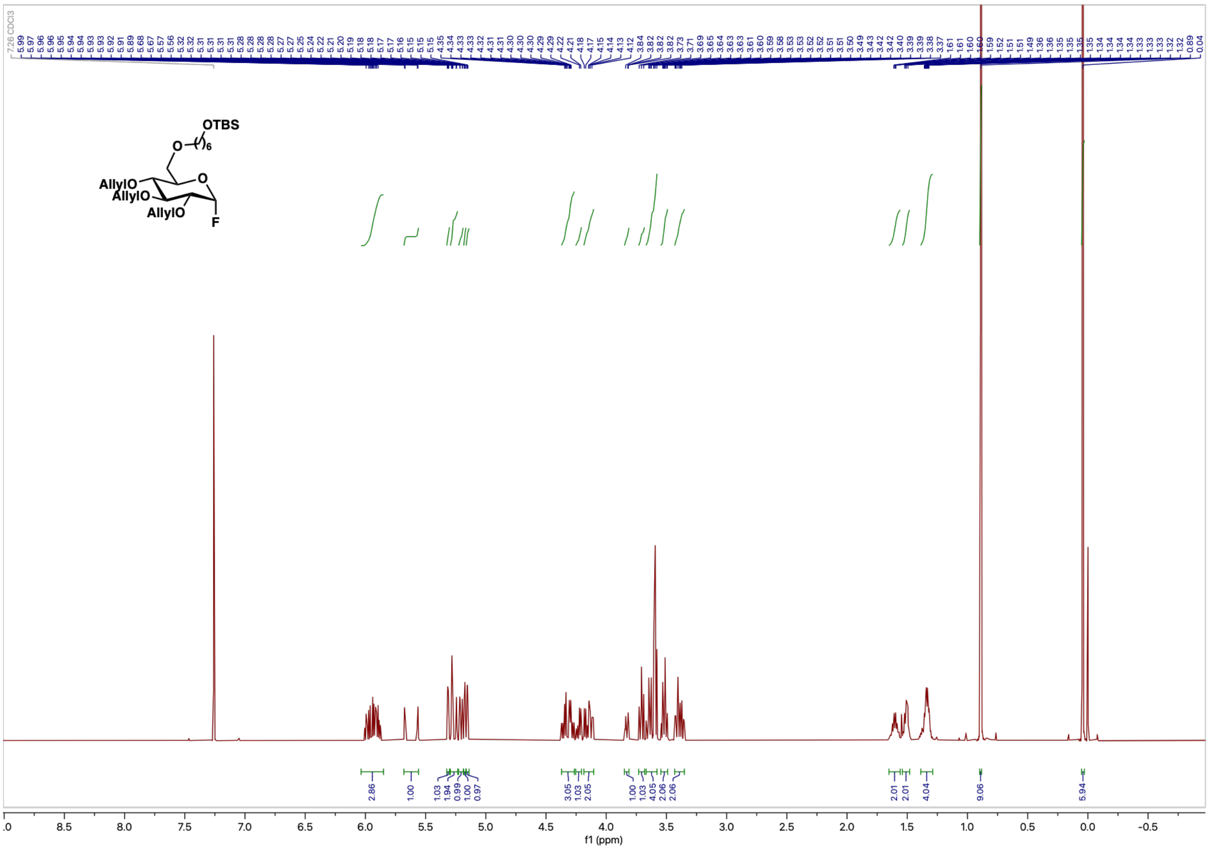

^{19}F NMR (470 MHz, $CDCl_3$, 298K) of compound **S19**

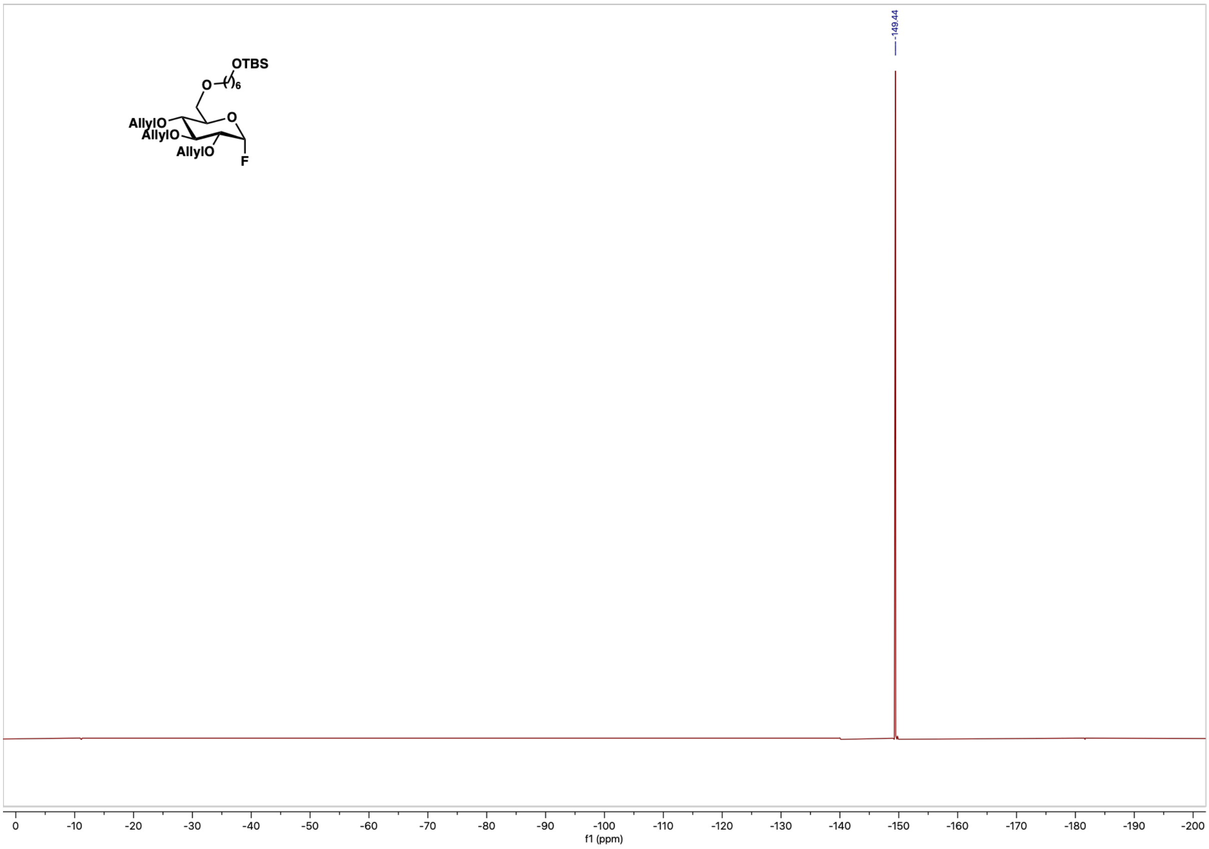

^{13}C NMR (126 MHz, $CDCl_3$, 298K) of compound **S19**

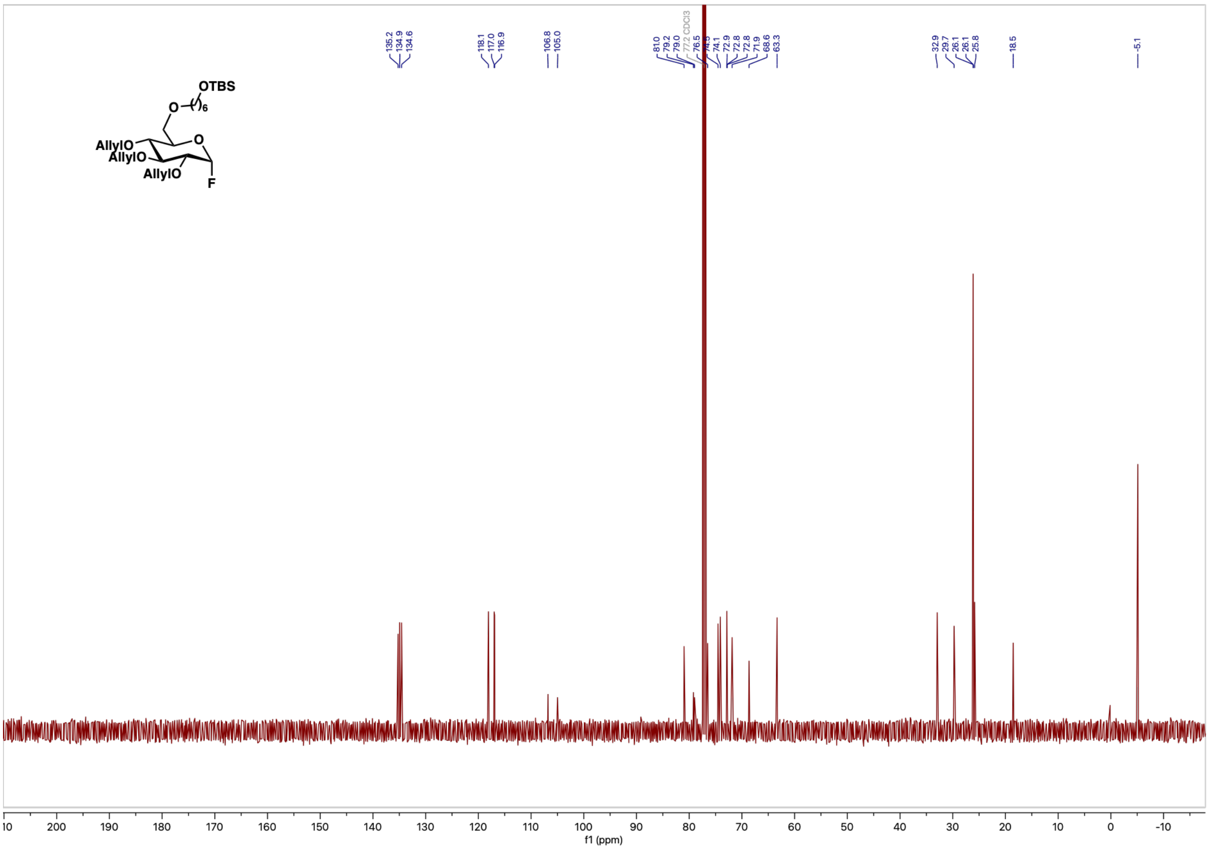

^{1}H NMR (500 MHz, acetone-d_6, 298K) of compound **S20**

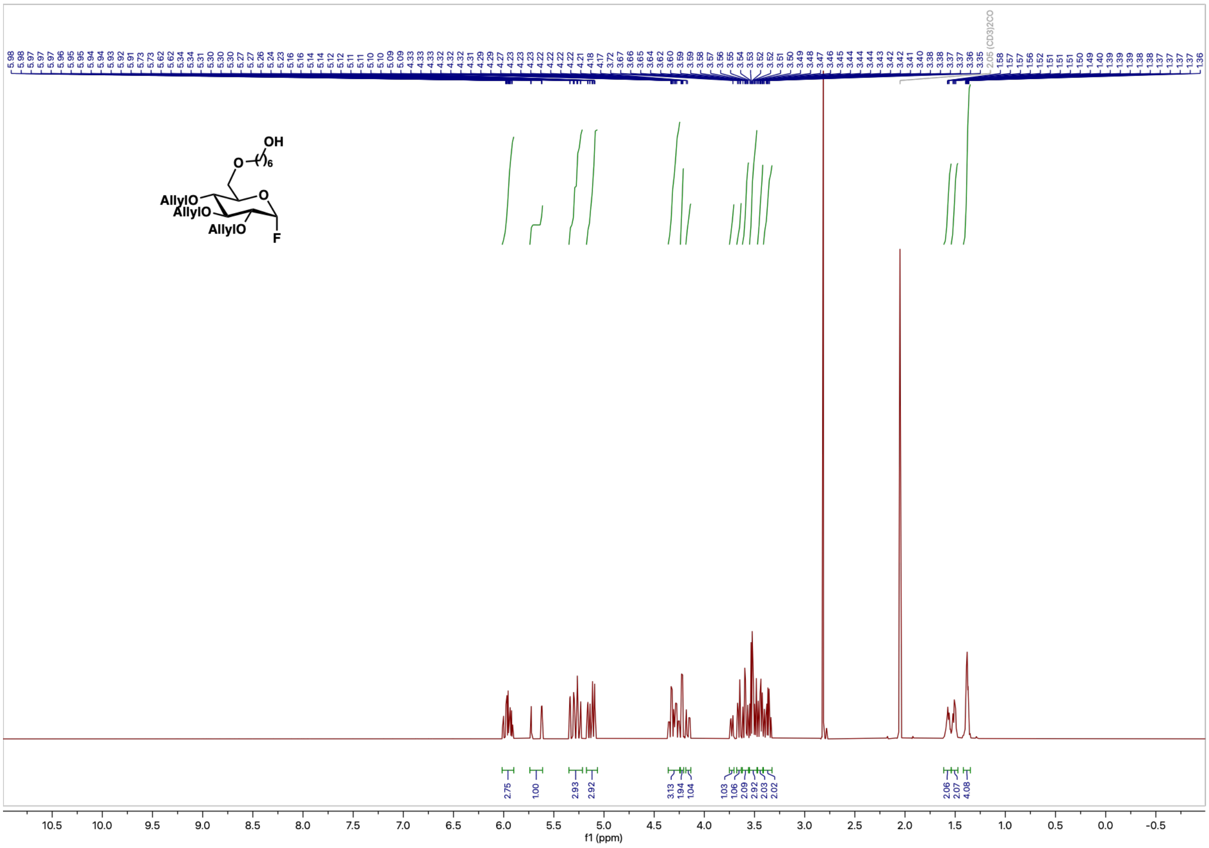

^{19}F NMR (470 MHz, acetone-d_6, 298K) of compound **S20**

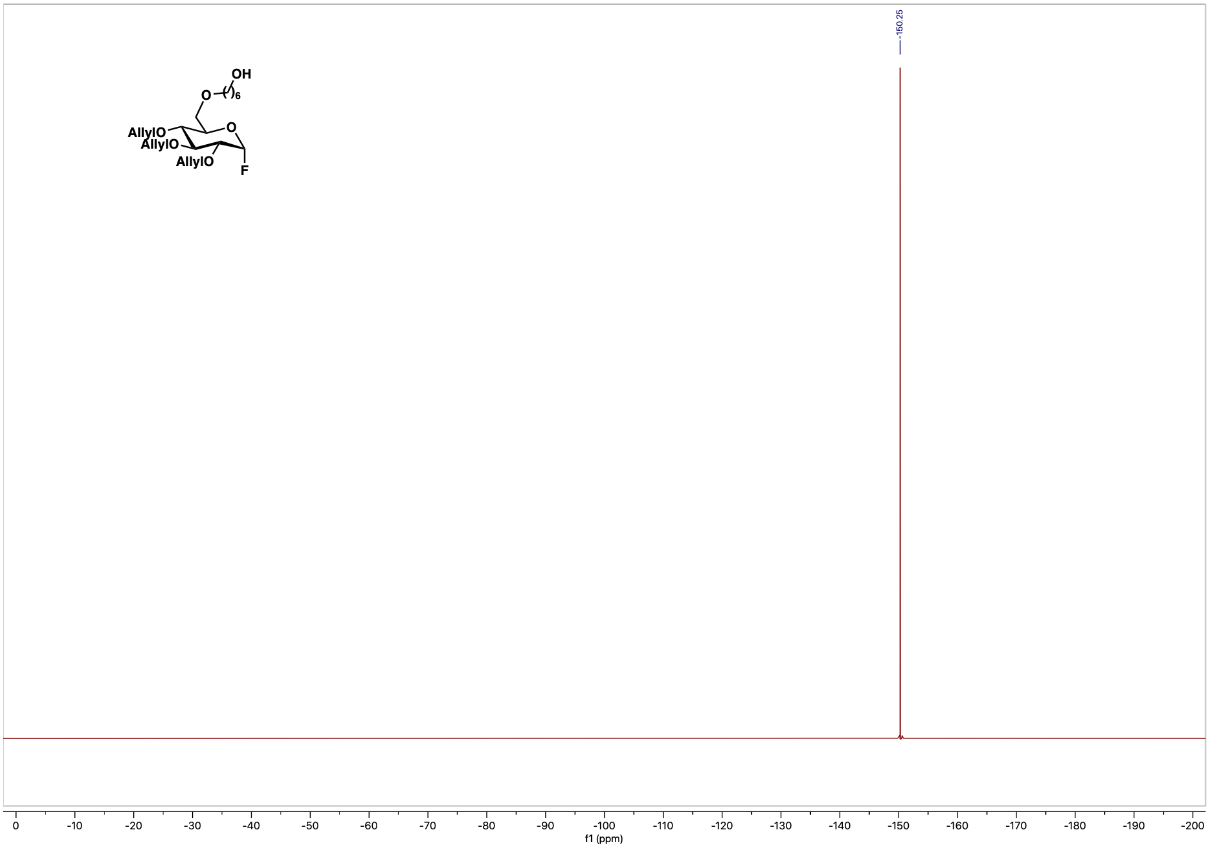

^{13}C NMR (126 MHz, acetone-d_6) of compound **S20**

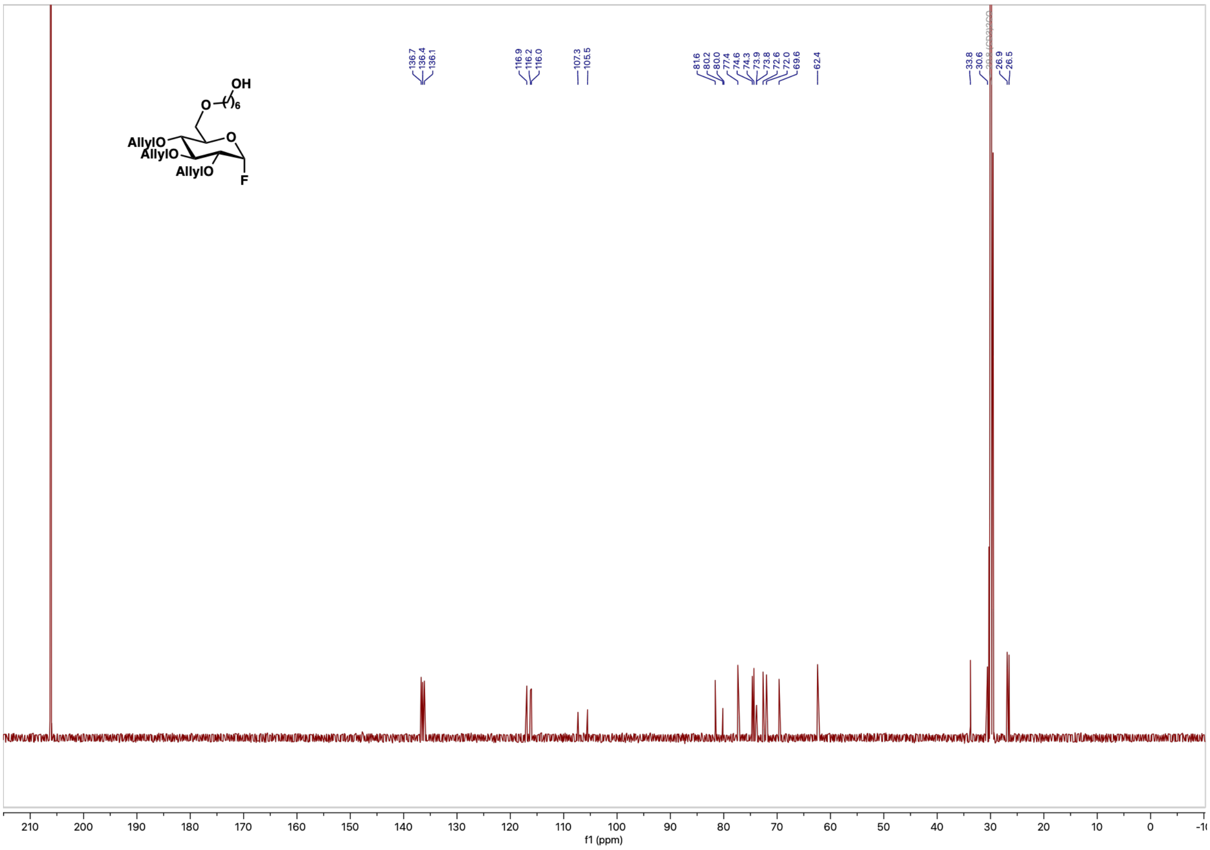

^{1}H NMR (500 MHz, $CDCl_3$, 298K) of compound **S21**

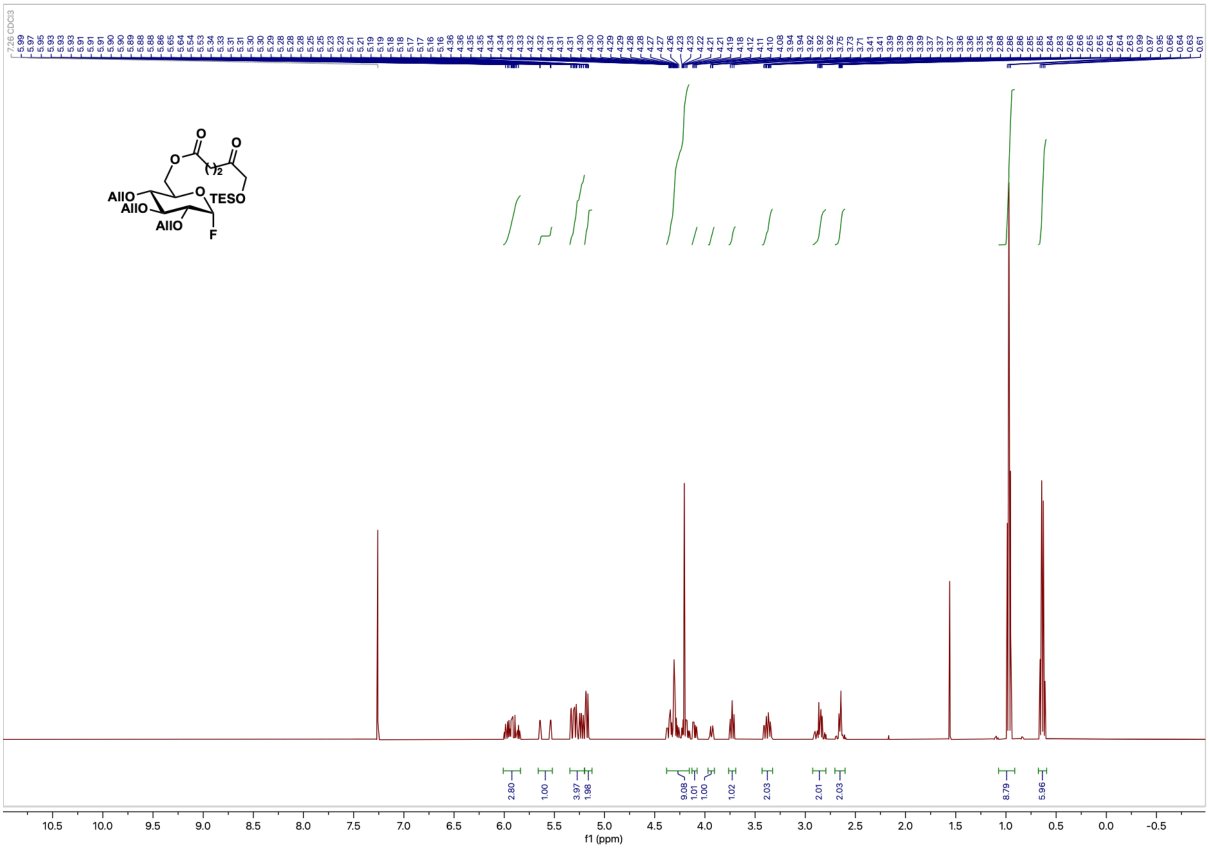

^{19}F NMR (470 MHz, $CDCl_3$, 298K) of compound **S21**

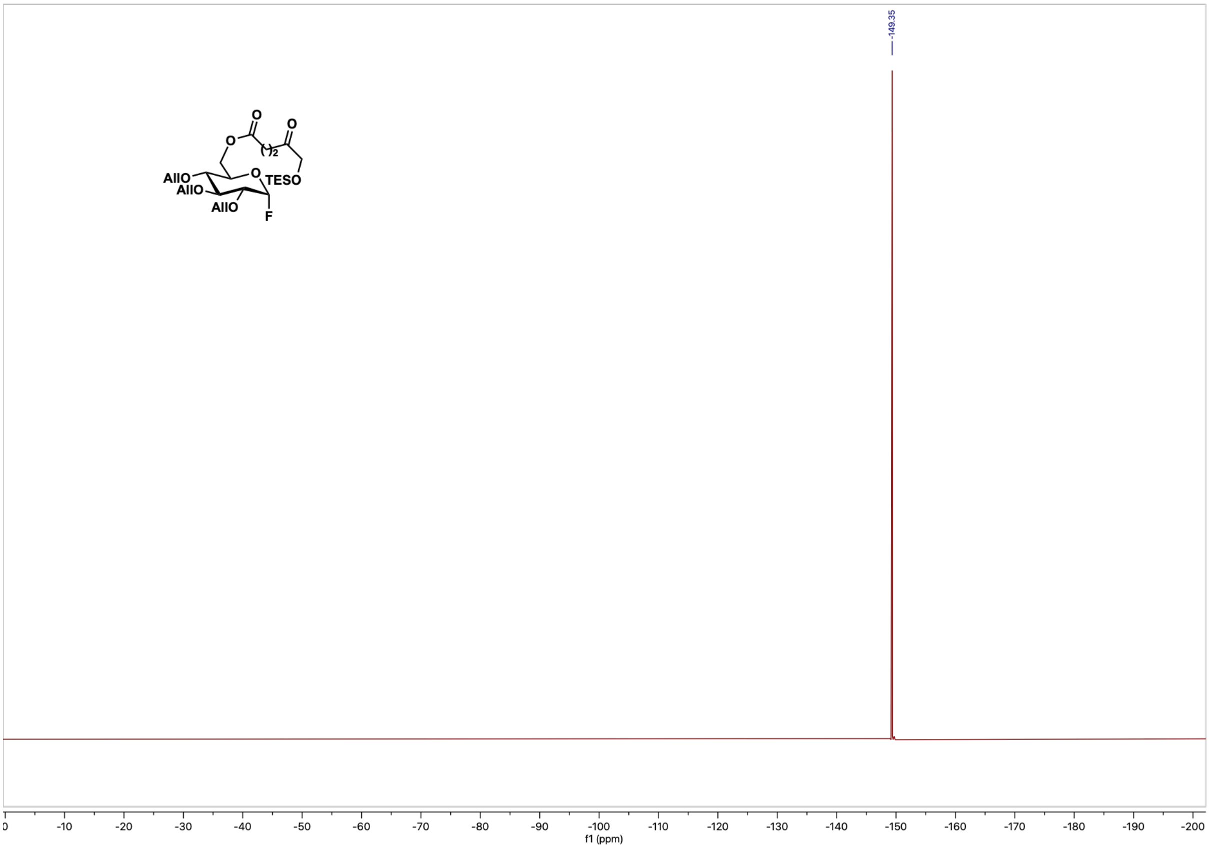

^{13}C NMR (126 MHz, $CDCl_3$, 298K) of compound **S21**

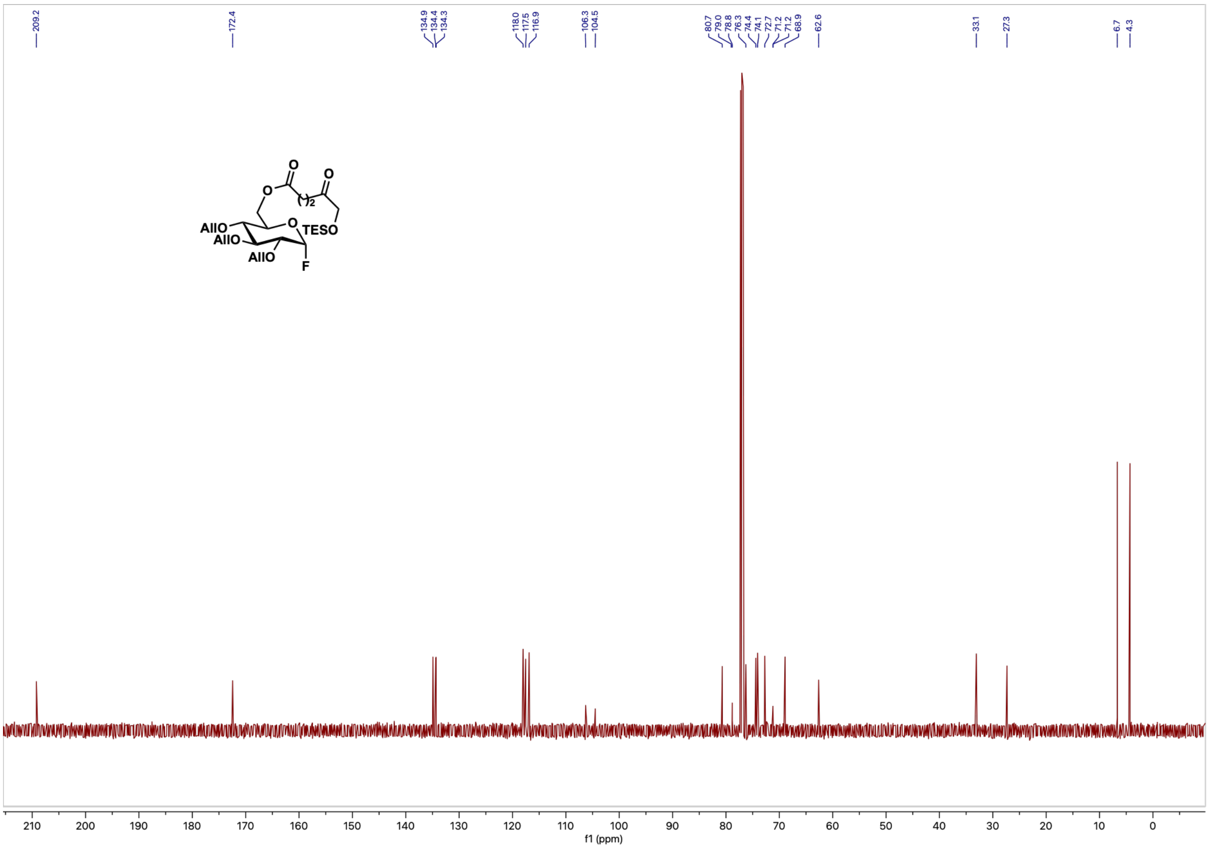

^{1}H NMR (500 MHz, acetone-*d6*, 298K) of compound **S22**

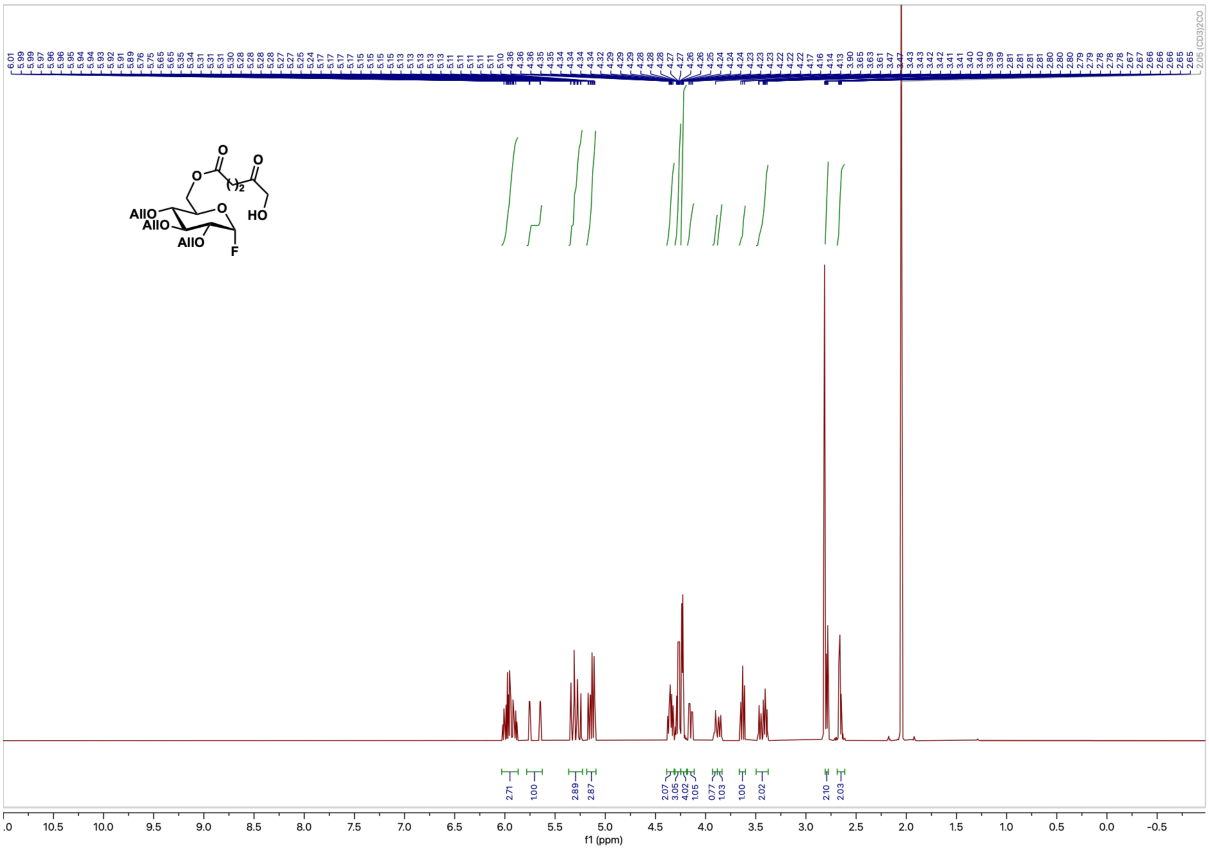

^{19}F NMR (470 MHz, acetone-*d6*, 298K) of compound **S22**

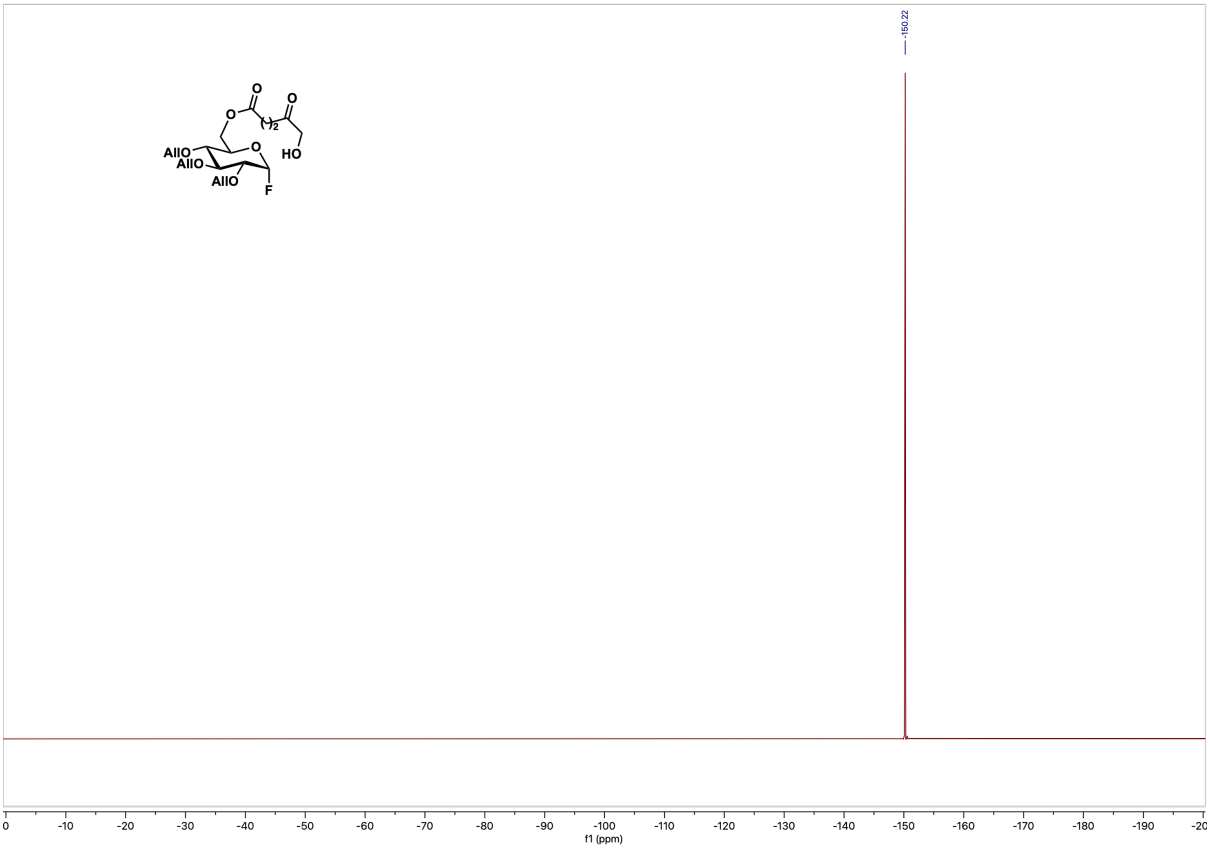

^{13}C NMR (126 MHz, $CDCl_3$, 298K) of compound **S22**

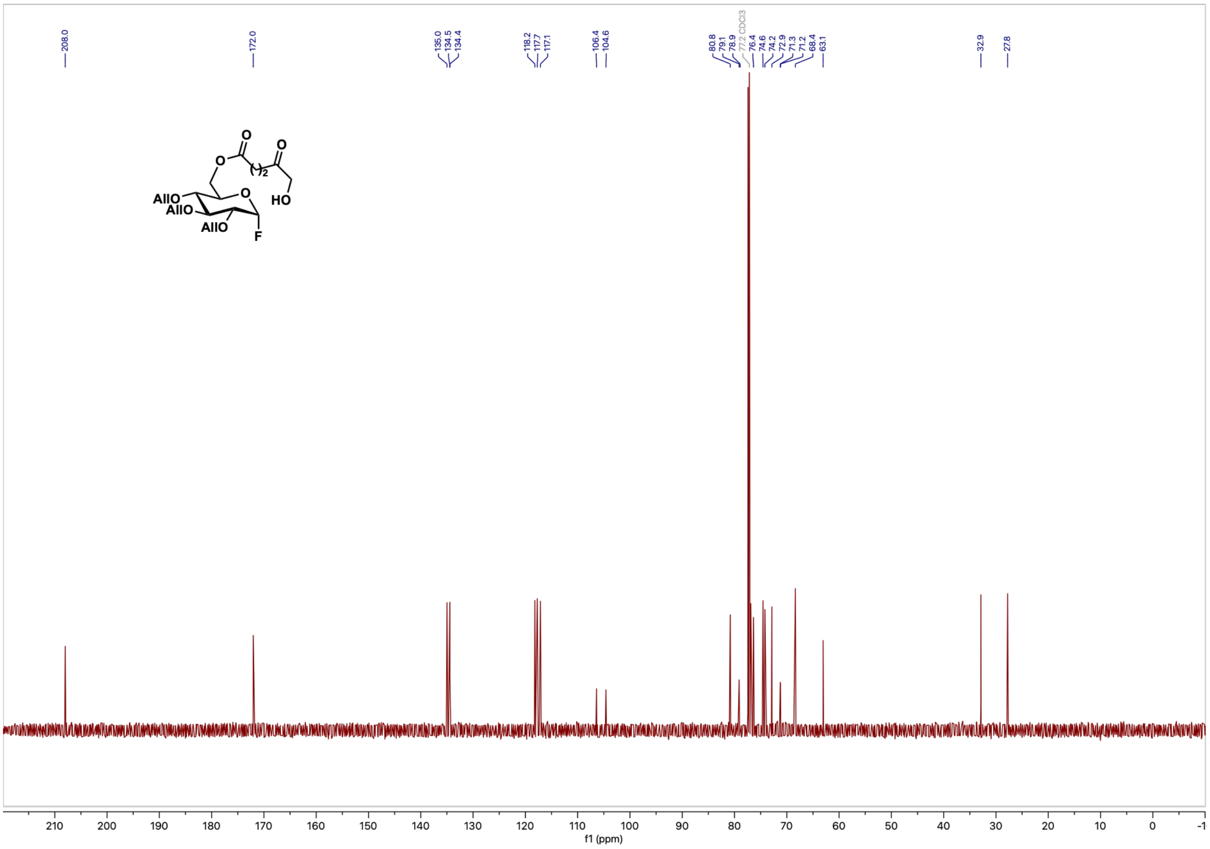

^{1}H NMR (500 MHz, $CDCl_3$, 298K) of compound **S23**

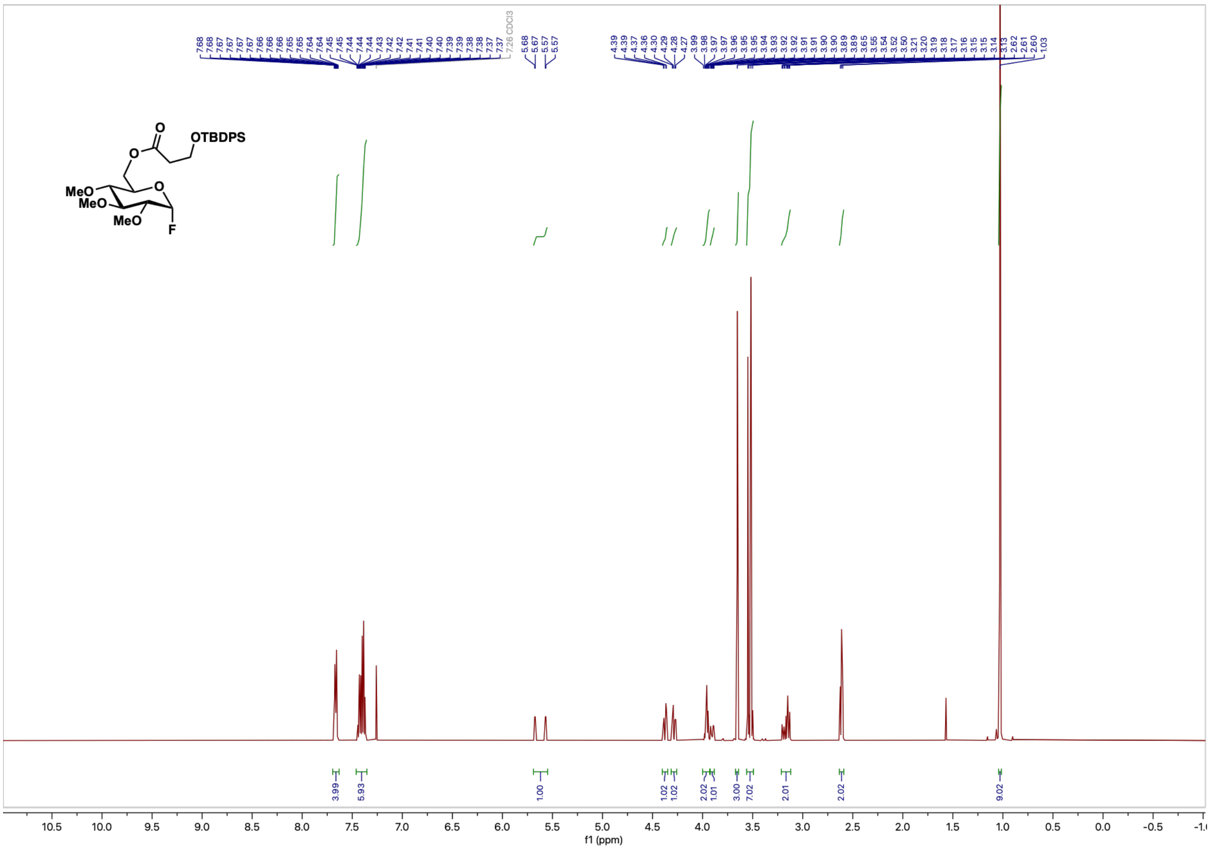

^{19}F NMR (470 MHz, $CDCl_3$, 298K) of compound **S23**

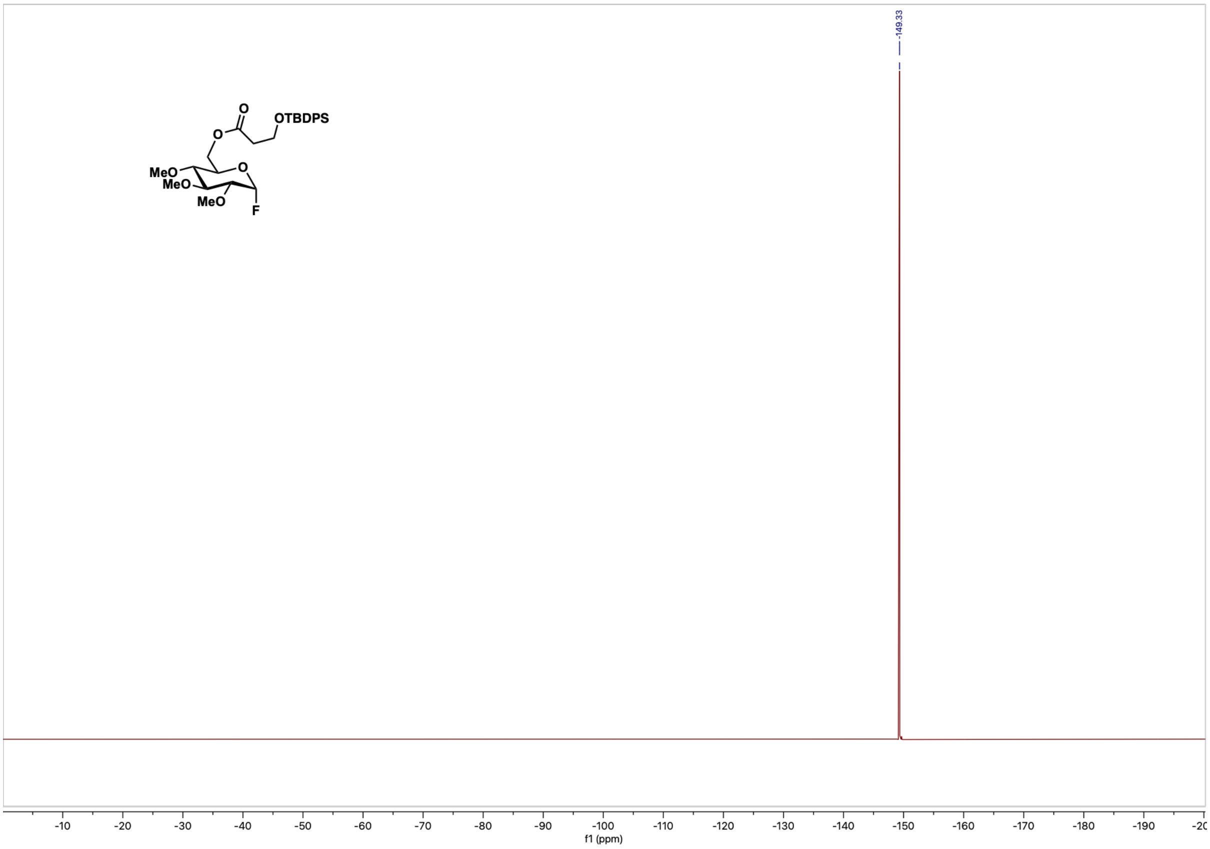

^{1}H NMR (500 MHz, $CDCl_3$, 298K) of compound **S23**

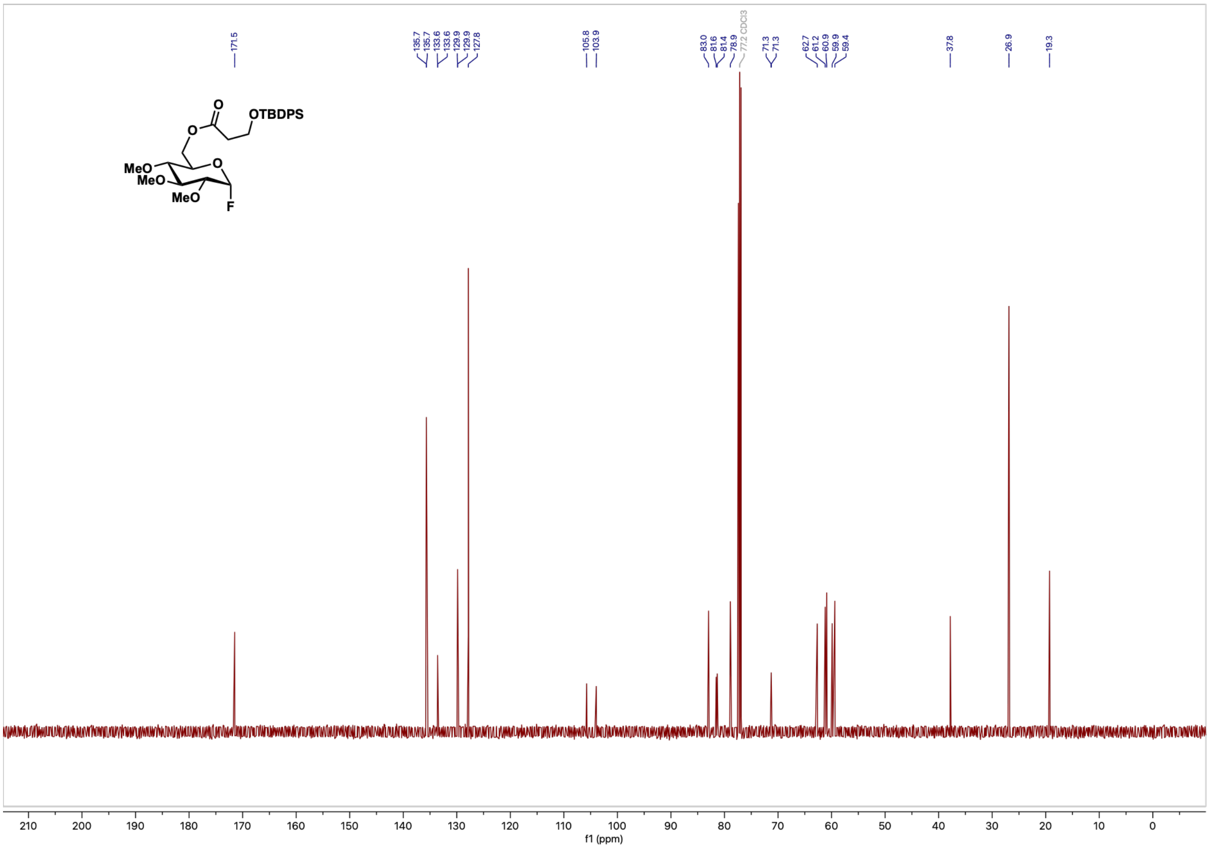

^{1}H NMR (500 MHz, acetone-d_6, 298K) of compound **9**

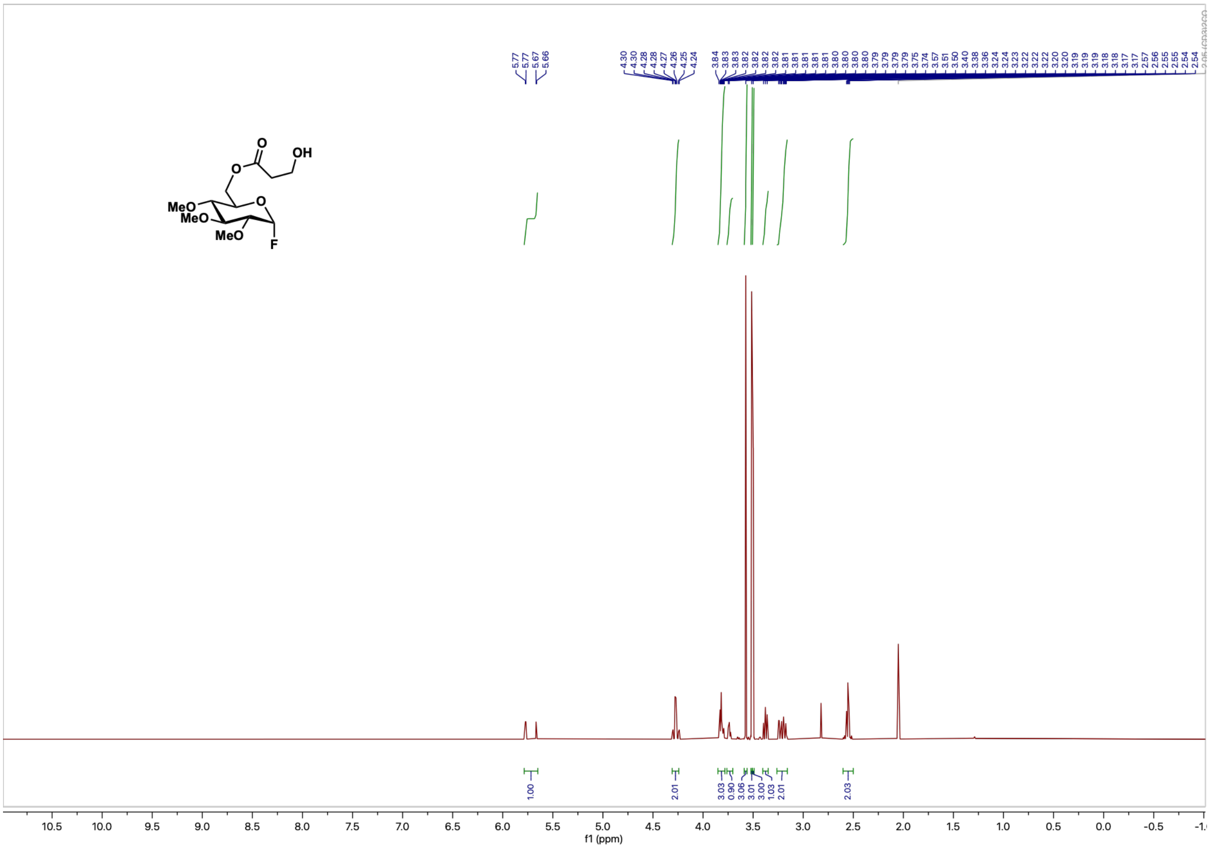

^{19}F NMR (470 MHz, acetone-d_6, 298K) of compound **9**

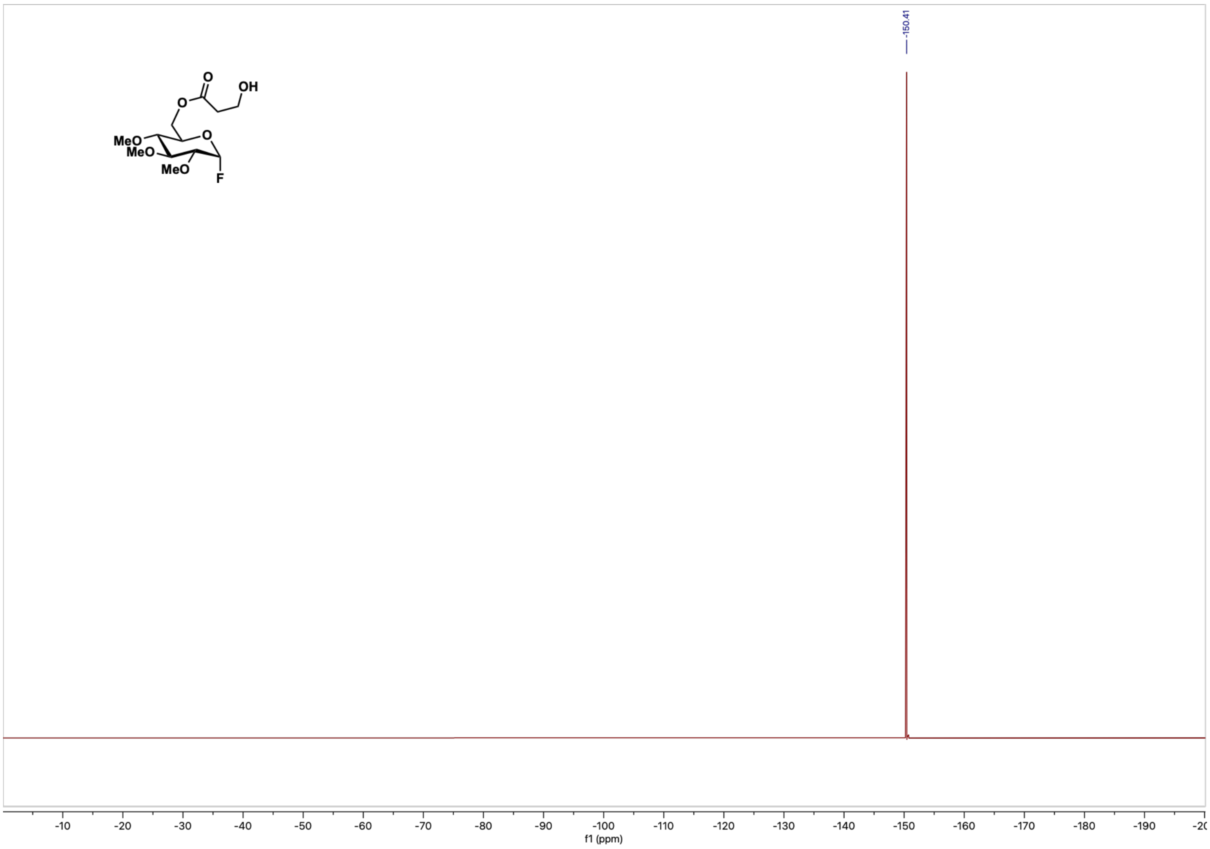

^{13}C NMR (126 MHz, acetone-*d6*, 298K) of compound **9**

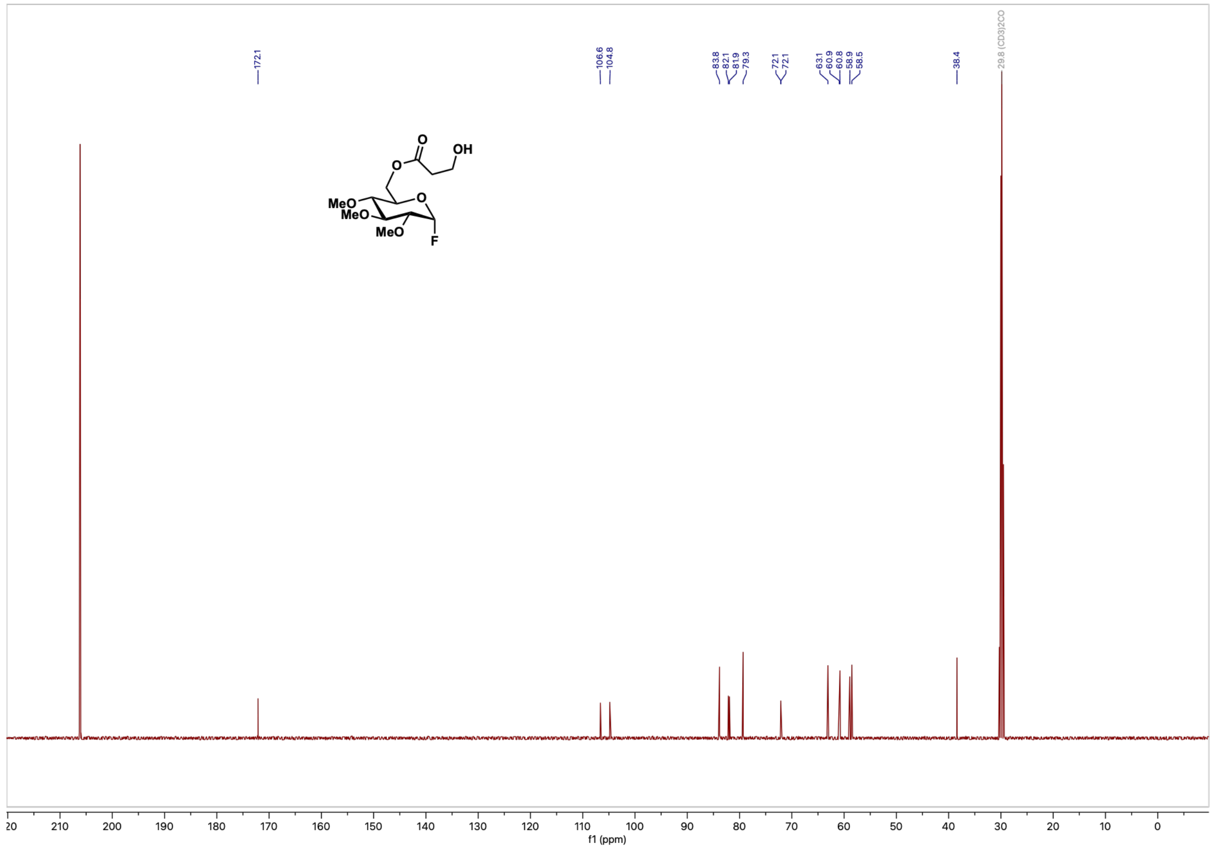

^{1}H NMR (500 MHz, acetone-d_6, 298K) of compound **10**

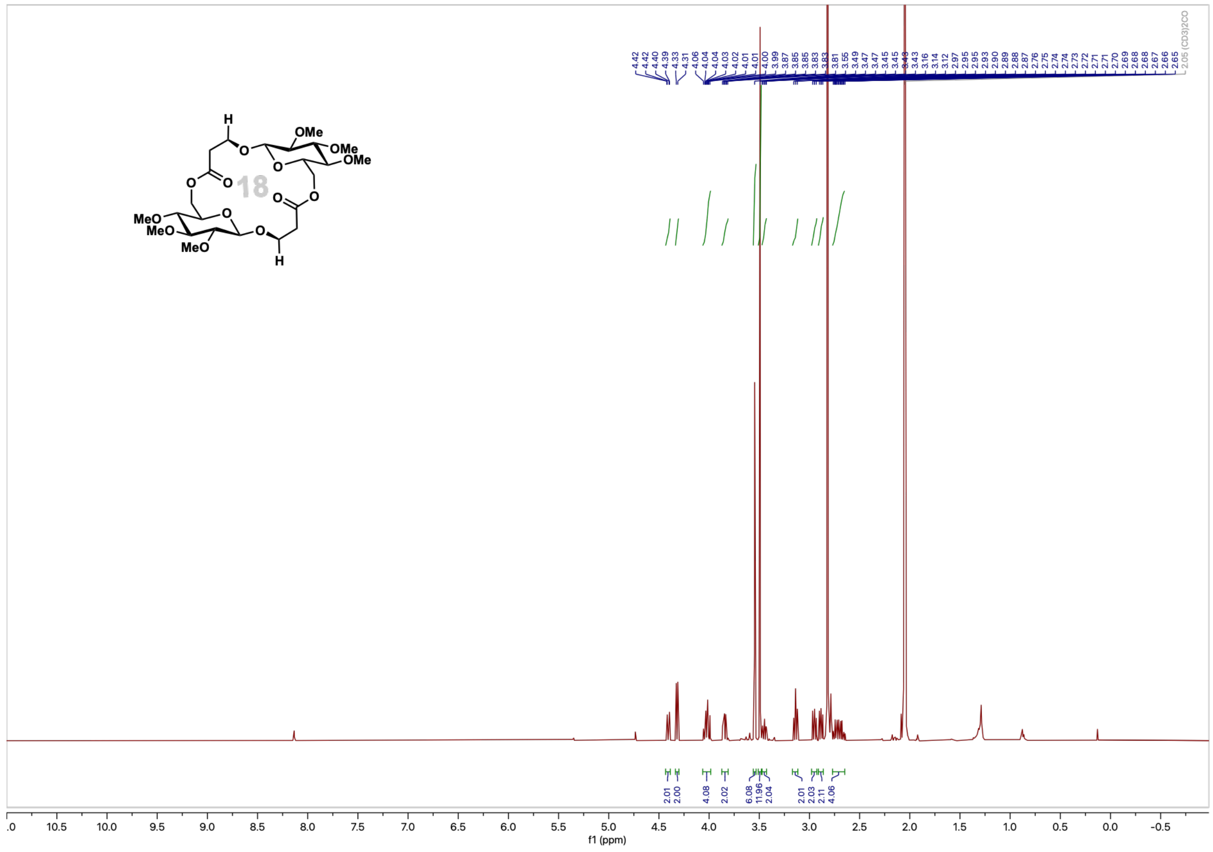

^{13}C NMR (500 MHz, acetone-d_6, 298K) of compound **10**

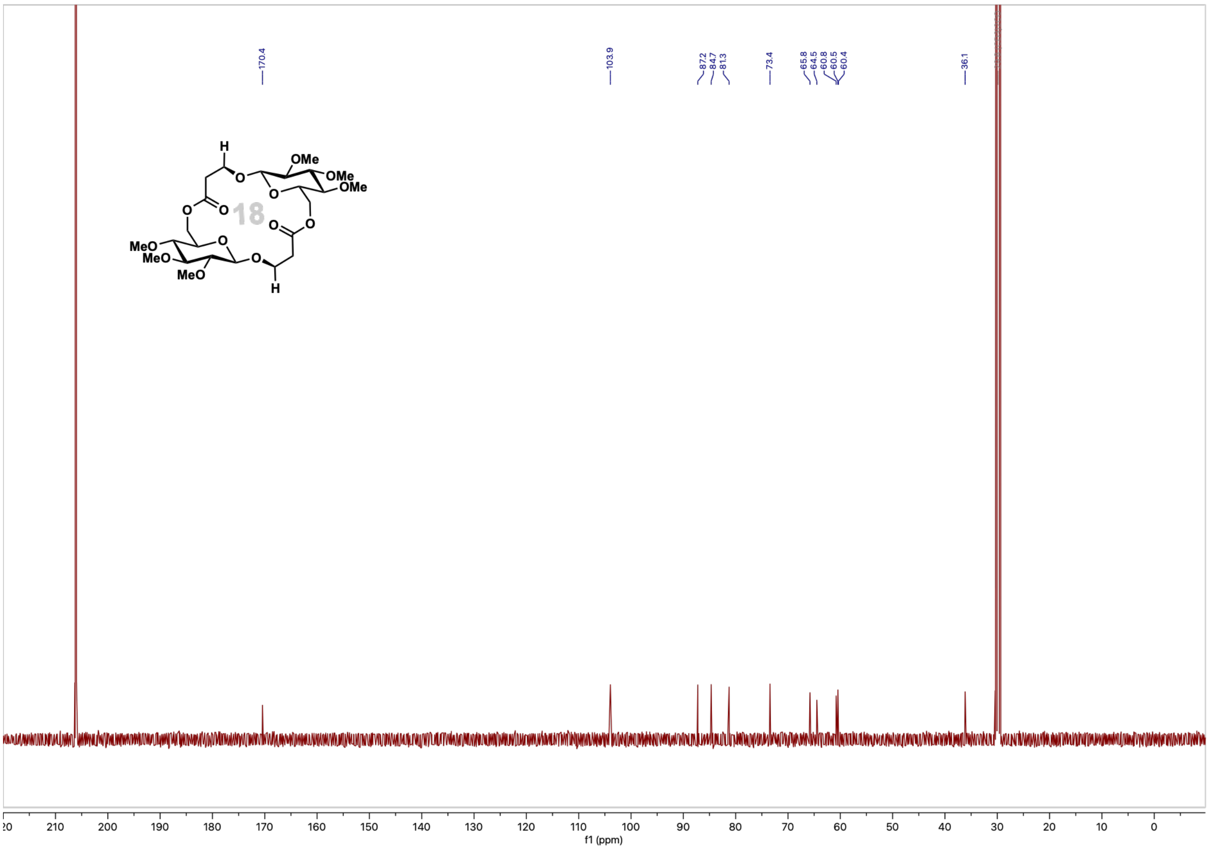

Supplement: Supplementary file 1 [file ja6c03730_si_001.pdf]
